# Supplementary material for: Regio- and enantioselective CuH-catalyzed 1,2- and 1,4-hydrosilylation of 1,3-enynes
Source: Nat Commun. 2023 Aug 19;14:5048. doi: 10.1038/s41467-023-40703-1 (PMC10439940; doi:10.1038/s41467-023-40703-1)
Supplement: Supplementary file 1 — Supplementary Information [file 41467_2023_40703_MOESM1_ESM.pdf]

Supplementary Information

Regio- and Enantioselective CuH-Catalyzed 1,2- and 1,4-Hydrosilylation of 1,3-Enynes

Zi-Lu Wang,[1] Qi Li,[1] Meng-Wei Yang,[1] Zhao-Xin Song,[1] Zhen-Yu Xiao,[1] Wei-Wei Ma,[1] Jin-Bo Zhao,*[2] and Yun-He Xu*[1]

1. Department of Chemistry, University of Science and Technology of China, Hefei 230026, P. R. China

2. Faculty of Chemistry and Life Science, Changchun University of Technology, Changchun 130012, P.R. China.

*Email: zhaojinbo@ccut.edu.cn
*E-mail: xyh0709@ustc.edu.cn

Content

1. General Information....3
2. Experiment Procedures....4
   2.1 Synthesis of 1,3-Enynes....4
   2.2 Synthesis of Products....4
   2.3 Effect of Chlorinated Solvents....8
   2.4 Optimization of asymmetric 1,2-hydrosilylation reaction....8
3. Characterization Data and Spectra of Substrates and Products....10
4. Computational Details.....182
5. References....274

1. General Information

Unless otherwise noted, reagents and solvents were purchased from commercial suppliers (such as Shanghai Titan Scientific Co., Ltd., Energy Chemical Corporation, *J&K* Scientific, Sinopharm Chemical Reagent Corporation *etc*.) and used without further purification, extra dry DCE was purchased from Energy Chemical Corporation, with molecular sieves, water = 17 ppm. ^{1}H NMR, ^{13}C NMR and ^{19}F NMR spectra were recorded at 25 ºC on a Bruker Advance 400M NMR spectrometer ($CDCl_3$). Chemical shifts for ^{1}H NMR spectra were reported as δ in parts per million (ppm) downfield from $SiMe_4$ (δ 0.00) and relative to the signal of $CDCl_3$ residual peak (δ 7.26 singlet). Multiplicities were given as: s (singlet); d (doublet); t (triplet); q (quartet); dd (doublet of doublets); dt (doublet of triplets); m (multiplets) *etc*. Chemical shifts for ^{13}C NMR spectra were reported as δ in parts per million (ppm) downfield from $SiMe_4$ (δ 0.00) and relative to the signal of $CDCl_3$ (δ 77.16 triplet). Coupling constants were reported as *J* value in Hz. High-resolution mass spectral analysis (HRMS) was performed on Waters XEVO G2 Q-TOF using electrospray ionization (ESI) or Thermo Scientific Q Exactive GC Orbitrap using electron ionization (EI). Flash chromatography was performed using 200-300 mesh silica gel with the indicated solvent system. Enantiomeric excesses of chiral compounds were determined by chiral high-performance liquid chromatography analyses which were performed on an Agilent 1260 Infinity equipped with Daicel Chiralpak OJ-3 column. Optical rotations were recorded on an Anton Paar MCP 200 polarimeter at 589 nm in $CHCl_3$. Single crystal X-ray diffraction data was collected on the Rigaku Oxford Diffraction (ROD) SuperNova Diffraction System.

2. Experiment Procedures

2.1 Synthesis of 1,3-Enynes

1,3-Enynes **1a-1n, 1q-1u, 1z** are known compounds.

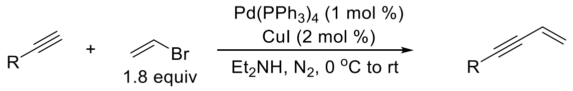

Procedure 1:

CuI (2 mmol %) and Pd(PPh$_3$)$_4$ (1 mmol %) were dissolved in diethylamine (1.50 mL/1.0 mmol alkyne) under nitrogen. The reaction mixture was cooled to 0 °C, then alkynes (1.0 equiv) and vinyl bromide (1.8 equiv, 1.0 M in THF) were added and the resulting mixture was warmed up to room temperature. After complete consumption of the starting material (monitored by TLC), ethyl acetate was added, and the precipitate was removed by filtration. The resultant solution was concentrated, and the crude products were purified by column chromatography to give **1a-1aa**.

2.2 Synthesis of Products

Procedure 2

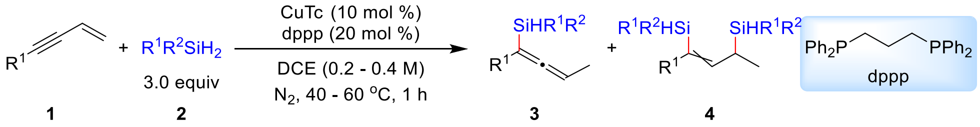

An oven dried 10-mL Schlenk tub equipped with a stirring bar, was charged with CuTc (10 mol %, 0.02 mmol, 3.8 mg), dppp (20 mol %, 0.04 mmol, 16.5 mg), extra dry DCE (0.5 or 1.0 mL), **2** (3.0 equiv, 0.6 mmol) and **1** (0.2 mmol) in sequence. The reaction mixture was stirred at the indicated temperature for 1 h. Then, ethyl acetate was added, and the precipitate was removed by filtration. The resultant solution was concentrated, and the crude product was purified by column chromatography.

Procedure 3

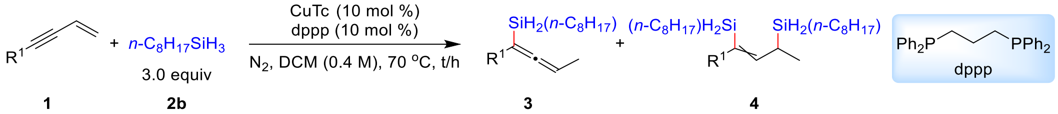

An oven dried 10-mL Schlenk tube equipped with a stirring bar, was added CuTc (10 mol %, 0.02 mmol, 3.8 mg), dppp (10 mol %, 0.02 mmol, 8.2 mg), 0.5 mL extra dry DCM, **2b** (3.0 equiv, 0.6 mmol, 86.5 mg) and **1** (0.2 mmol) in sequence. The reaction mixture was stirred at 70 °C. After completion (monitored by TLC), ethyl acetate was added, and the precipitate was removed by filtration. The resultant solution was concentrated, and the crude product was purified by column chromatography.

Procedure 4

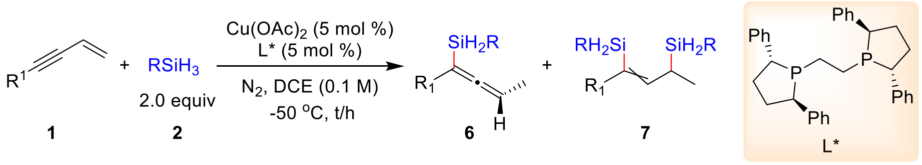

An oven dried 10-mL Schlenk tube equipped with a stirring bar, was added $Cu(OAc)_2$ (5 mol %, 0.01 mmol, 1.8 mg), L* (5 mol %, 0.01 mmol, 5.1 mg) and 2 mL extra dry DCE. The mixture was stirred at 30 °C for 5 minutes, then cooled to -35 °C (about 1 minute) following by adding **2** (2.0 equiv, 0.4 mmol) and **1** (0.2 mmol) to it at the same temperature. After that, the reaction mixture was stirred at -50 °C. Then ethyl acetate was added, and the precipitate was removed by filtration. The resultant solution was concentrated, and the crude product was purified by column chromatography.

Procedure 5

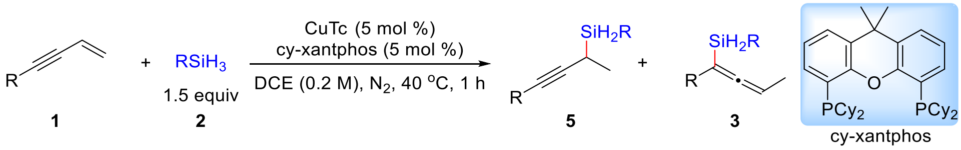

An oven dried 10-mL Schlenk tube equipped with a stirring bar, was added CuTc (5 mol %, 0.01 mmol, 1.9 mg), cy-xantphos (5 mol %, 0.01 mmol, 6.0 mg), 1 mL extra dry DCE, **2** (1.5 equiv, 0.3 mmol) and **1** (0.2 mmol) in sequence. The reaction mixture was stirred at 40 °C. After completion, ethyl acetate was added, and the precipitate was removed by filtration. The resultant solution was concentrated, and the crude product was purified by column chromatography.

Procedure 6

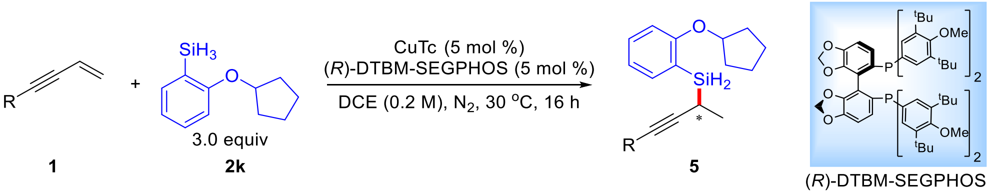

An oven dried 10-mL Schlenk tube equipped with a stirring bar, was added CuTc (5 mol %, 0.01 mmol, 1.9 mg), (*R*)-DTBM-SEGPHOS (5 mol %, 0.01 mmol, 11.8 mg) and 1 mL extra dry DCE. The mixture was stirred at 30 °C for 30 minutes, then **2k** (3.0 equiv, 0.6 mmol, 115.4 mg) and **1** (0.2 mmol) were added. After that, the reaction mixture was stirred at 30 °C. After completion, ethyl acetate was added, and the precipitate was removed by filtration. The resultant solution was concentrated, and the crude product was purified by column chromatography.

Scaled-up experiments and derivatizations

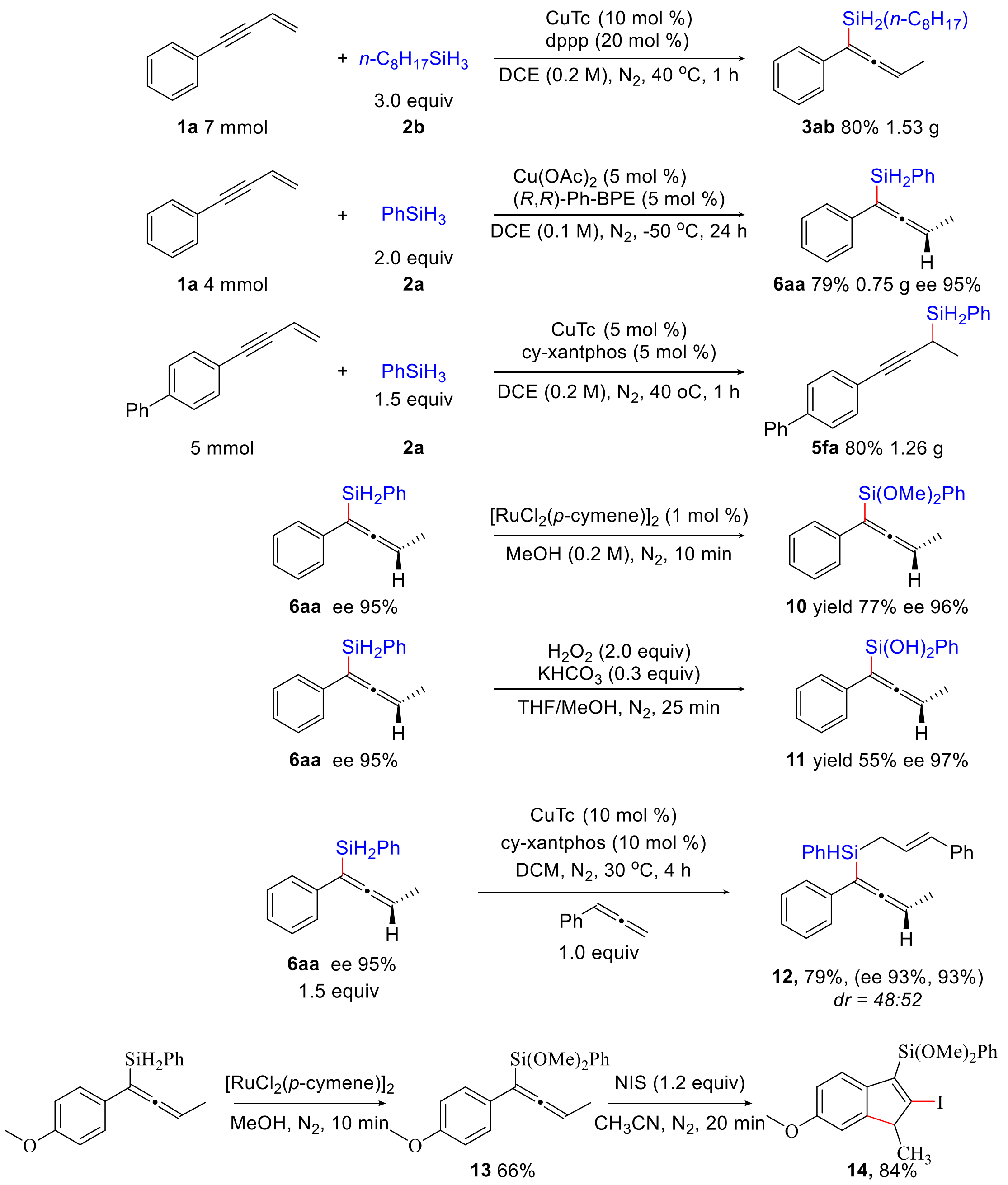

Procedure 7

Synthesis of **3ab**: An oven dried 100-mL Schlenk flask equipped with a stirring bar, was added CuTc (10 mol %, 0.7 mmol, 133.4 mg), dppp (20 mol %, 1.4 mmol, 577.5 mg), 35 mL dry DCE, **2b** (3.0 equiv, 21 mmol, 3.0 g) and **1a** (7 mmol, 897.4 mg) in sequence. The reaction mixture was stirred at 40 °C for 1 h. Then ethyl acetate was added, and the precipitate was removed by filtration. The resultant solution was concentrated, and the crude product was purified by column chromatography to give **3ab** in 80% yield (5.6 mmol, 1.53 g).

Procedure 8

Synthesis of **6aa**: An oven dried 100-mL Schlenk flask equipped with a stirring bar, was added $Cu(OAc)_2$ (5 mol %, 0.2 mmol, 36.0 mg), (*R*,*R*)-Ph-BPE (5 mol %, 0.2 mmol, 101.3 mg) and 40 mL dry DCE. The mixture was stirred at 30 °C for 5 minutes, then

cooled to -35 °C (about 3 minutes) following by adding **2a** (2.0 equiv, 8 mmol, 865.6 mg) and **1a** (4 mmol, 512.2 mg) to it at the same temperature. After that, the reaction mixture was stirred at -50 °C for 24 h. Then ethyl acetate was added, and the precipitate was removed by filtration. The resultant solution was concentrated, and the crude product was purified by column chromatography to give **6aa** in 79% yield (3.16 mmol, 0.75 g) with 95% ee value.

Procedure 9

Synthesis of **5fa**: An oven dried 100-mL Schlenk flask equipped with a stirring bar, was added CuTc (5 mol %, 0.25 mmol, 47.7 mg), cy-xantphos (5 mol %, 0.25 mmol, 144.6 mg), 25 mL dry DCE, **2a** (1.5 equiv, 7.5 mmol, 811.5 mg) and **1a** (5 mmol, 1.02 g) in sequence. The reaction mixture was stirred at 40 °C for 1 h. Then ethyl acetate was added, and the precipitate was removed by filtration. The resultant solution was concentrated, and the crude product was purified by column chromatography to give **5fa** in 80% yield (4.0 mmol, 1.26 g).

Procedure 10

Synthesis of **10**: An oven dried 10-mL Schlenk tube equipped with a stirring bar, was added $[RuCl_2(p\text{-cymene})]_2$ (1 mol %, 0.002 mmol, 1.3 mg), 1 mL dry MeOH, and **6aa** (0.2 mmol, 47.3 mg) in sequence. The reaction mixture was stirred at 30 °C for 10 minutes. After completion, ethyl acetate was added, and the precipitate was removed by filtration. The resultant solution was concentrated, and the crude product was purified by column chromatography to give **10** in 77% yield (0.15 mmol, 45.6 mg) with 96% ee value.

Procedure 11

Synthesis of **11**: An oven dried 10-mL Schlenk tube equipped with a stirring bar, was added $KHCO_3$ (0.3 equiv, 6.0 mg), 4 mL THF/MeOH (1:1) and **6aa** (0.2 mmol, 47.3 mg) in sequence, then 30% H_2O_2 (2.0 equiv, 45.0 mg) was added slowly. The reaction mixture was stirred at 30 °C for 25 minutes. The reaction mixture was diluted with H_2O and extracted with ethyl acetate. The combined organic layer was dried over anhydrous sodium sulfate, filtered and concentrated under reduced pressure. The crude residue was purified by flash chromatography affording **11** in 55% yield (0.11 mmol, 29.5 mg) with 97% ee value.

Procedure 12

Synthesis of **12**: An oven dried 10-mL Schlenk tube equipped with a stirring bar, was added CuTc (10 mol %, 1.9 mg), cy-xantphos (10 mol %, 6.0 mg) and dry DCM (0.5 mL). The mixture was stirred at 30 °C for 30 minutes, then phenyl allene (0.1 mmol) and **6aa** (0.15 mmol) were added. After that, the reaction mixture was stirred at 30 °C for 4 h. After completion, ethyl acetate was added, and the precipitate was removed by filtration. The resultant solution was concentrated, and the crude product was purified by column chromatography.

Procedure 13

Synthesis of **14**: An oven dried 10-mL Schlenk tube equipped with a stirring bar, was added NIS (1.2 equiv, 54 mg), and 2.0 mL CH_3CN (0.5 mL). then **13** (0.2 mmol, 65.3 mg) was added. The reaction mixture was stirred at 30 °C for 20 min. After completion, the reaction mixture was purified by column chromatography directly.

2.3 Effect of Chlorinated Solvents

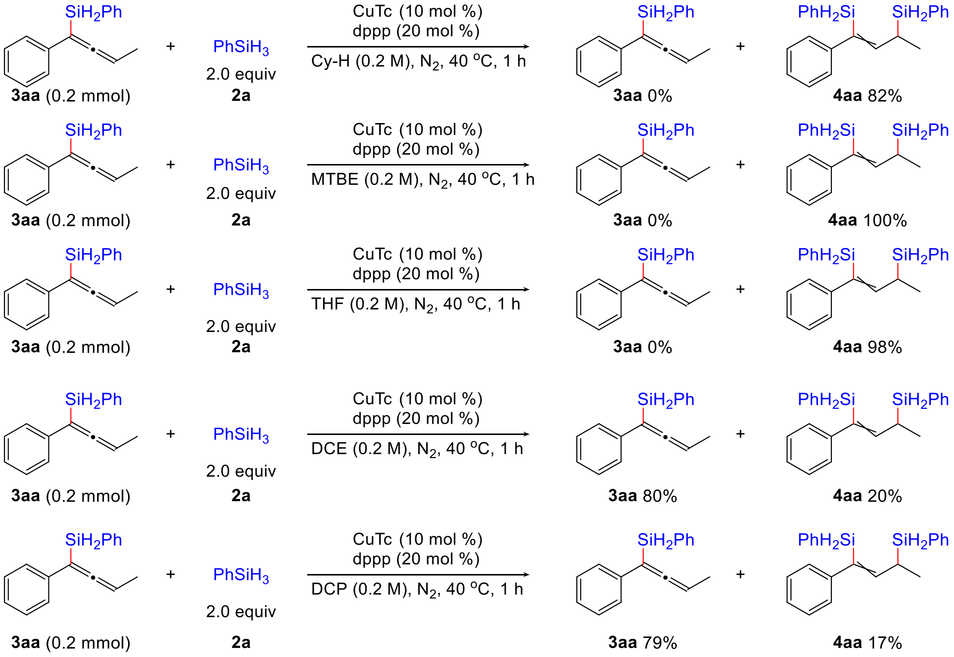

Supplementary Fig 1 Effect of Chlorinated Solvents[a] [a] The mixture of **3aa** (0.2 mmol), **2a** (0.6 mmol), CuTc (10 mol %), and dppp (20 mol %) in solvent (1.0 mL) was stirred at 40 °C for 1 h under nitrogen atmosphere in an oil bath. The yields were determined by crude ^{1}H NMR using 1,1,2,2-tetrachlorethane as an internal standard.

When allenylsilane **3aa** was subjected to the reaction conditions, we found complete conversion of **3aa** to **4aa** using Cy-H, MTBE or THF as solvent, while **3aa** was partially converted to **4aa** using DCE or DCP as solvent (Supplementary Fig 1).

2.4 Optimization of asymmetric 1,2-hydrosilylation reaction

Firstly, different chiral ligand were examined (Supplementary Tab 1, entries 1-14), only (*R*)-DTBM-SEGPHOS (L_8) provided the desired product in moderate yield and enantioselectivity (entry 8). This reaction cannot happened when other chiral phosphine ligands, nitrogen containing ligands or chiral NHC ligands were used. Sadly, whether we change the catalyst precursors or solvents, this reaction also cannot happen (entries 15-31). Besides, the effect of silanes on the enantioselectivity of asymmetric 1,2-hydrosilylation have also been investigated (Supplementary Fig 2).

Supplementary Tab 1. Optimization of Asymmetric 1,2-Hydrosilylation of 1,3-Enynes with Silanes

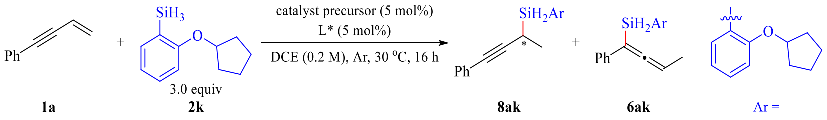

entry	catalyst precursor (5 mol %)	ligand (5 mol %)	solvent (1 mL)	T/°C	t/h	8ak yield %	8ak ee %	6ak yield %	6ak ee %
1	CuTc	L_1	DCE	30	16	6	--	40	--

	2	CuTc	L_2	DCE	30	16	14	--	51	--
	3	CuTc	L_3	DCE	30	16	trace		trace	
	4	CuTc	L_4	DCE	30	16	1	--	10	--
	5	CuTc	L_5	DCE	30	16	0		0	--
	6	CuTc	L_6	DCE	30	16	0		0	--
	7	CuTc	L_7	DCE	30	16	2	--	47	--
Screening of ligands	8	CuTc	L_8	DCE	30	16	77(70)	60	6	--
	9	CuTc	L_9	DCE	30	16	trace		25	
	10	CuTc	L_{10}	DCE	30	16	0		0	
	11	CuTc	L_{11}	DCE	30	16	0		0	
	12	CuTc	L_{12}	DCE	30	16	0		0	
	13	CuTc	L_{13}	DCE	30	16	0		0	
	14	CuTc	L_{14}	DCE	30	16	0		0	
	15	$Cu(OAc)_2$	L_8	DCE	30	16	0		0	
	16	CuOAc	L_8	DCE	30	16	0		0	
	17	$Cu(acac)_2$	L_8	DCE	30	16	0		0	
	18	CuCl	L_8	DCE	30	16	0		0	
	19	CuBr	L_8	DCE	30	16	0		0	
Screening of catalyst precursors	20	CuI	L_8	DCE	30	16	0		0	
	21	CuF_2	L_8	DCE	30	16	0		0	
	22	$CuCl_2$	L_8	DCE	30	16	0		0	
	23	copper(II) acrylate	L_8	DCE	30	16	15	--	8	--
	24	copper(II) methacrylate	L_8	DCE	30	16	12	--	7	--
	25	CuTc	L_8	DCM	30	16	72	59	4	
	26	CuTc	L_8	Toluene	30	16	0		0	
	27	CuTc	L_8	PhCl	30	16	0		0	
Screening of solvents	28	CuTc	L_8	CyH	30	16	0		0	
	29	CuTc	L_8	THF	30	16	0		0	
	30	CuTc	L_8	Et_2O	30	16	0		0	
	31	CuTc	L_8	1,4-dioxane	30	16	0		0	

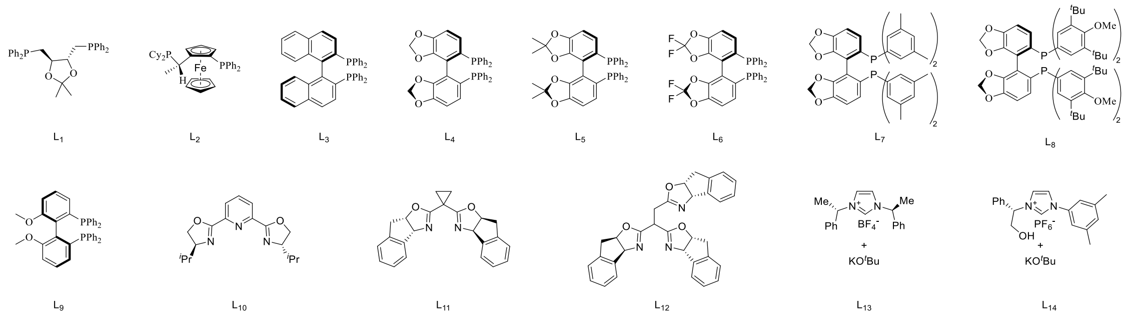

[a] Reaction conditions: catalyst precursor (5 mol %), ligand (5 mol %), **1a** (0.2 mmol) and **2k** were stirred in DCE (1 mL) under nitrogen atmosphere, the yields were determined by crude ^{1}H NMR using 1,1,2,2-tetrachlorethane as an internal standard.

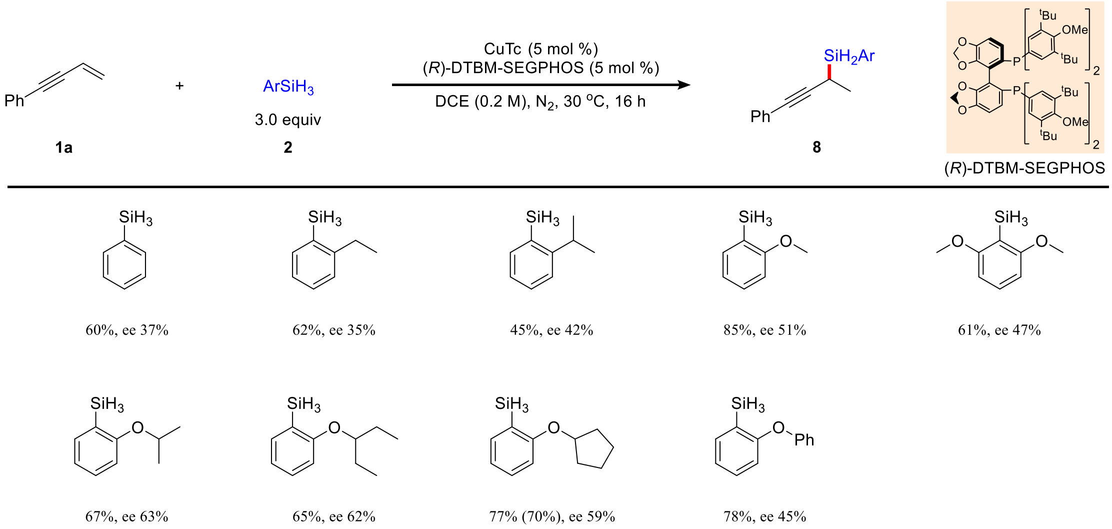

Supplementary Fig 2 Effect of silanes on the enantioselectivity of asymmetric 1,2-hydrosilylation.[a] [a] The mixture of 1,3-enynes **1a** (0.2 mmol), **2** (0.6 mmol), CuTc (5 mol %) and (*R*)-DTBM-SEGPHOS (5 mol %) in DCE (1.0 mL) was stirred at 30 °C under nitrogen atmosphere, the yields were determined by crude ^{1}H NMR using 1,1,2,2-tetrachlorethane as an internal standard.

3. Characterization Data and Spectra of Substrates and Products

3-(But-3-en-1-yn-1-yl)phenyl 4-methylbenzenesulfonate (1o)

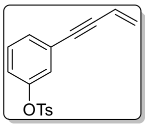

Prepared according to procedure 1 as an orange viscous liquid in 58% yield (3.5 mmol, 1.04 g).

R_f: 0.46 (ethyl acetate : petroleum ether = 1:20).

HRMS (ESI) (m/z): Calcd for $C_{17}H_{15}O_3S$ $[M+H]^+$: 299.0742, found: 299.0745.

^{1}H NMR (400 MHz, $CDCl_3$) δ 7.73 – 7.69 (m, 2H), 7.34 – 7.29 (m, 3H), 7.21 (t, *J* = 8.0 Hz, 1H), 7.11 (dd, *J* = 2.2, 1.5 Hz, 1H), 6.92 (ddd, *J* = 8.2, 2.4, 1.1 Hz, 1H), 5.98 (dd, *J* = 17.6, 11.1 Hz, 1H), 5.74 (dd, *J* = 17.6, 2.0 Hz, 1H), 5.57 (dd, *J* = 11.2, 2.0 Hz, 1H), 2.45 (s, 3H).

^{13}C NMR (101 MHz, $CDCl_3$) δ 149.5, 145.7, 132.3, 130.4, 130.0, 129.6, 128.6, 128.0, 125.5, 124.9, 122.5, 116.9, 89.4, 88.5, 21.9.

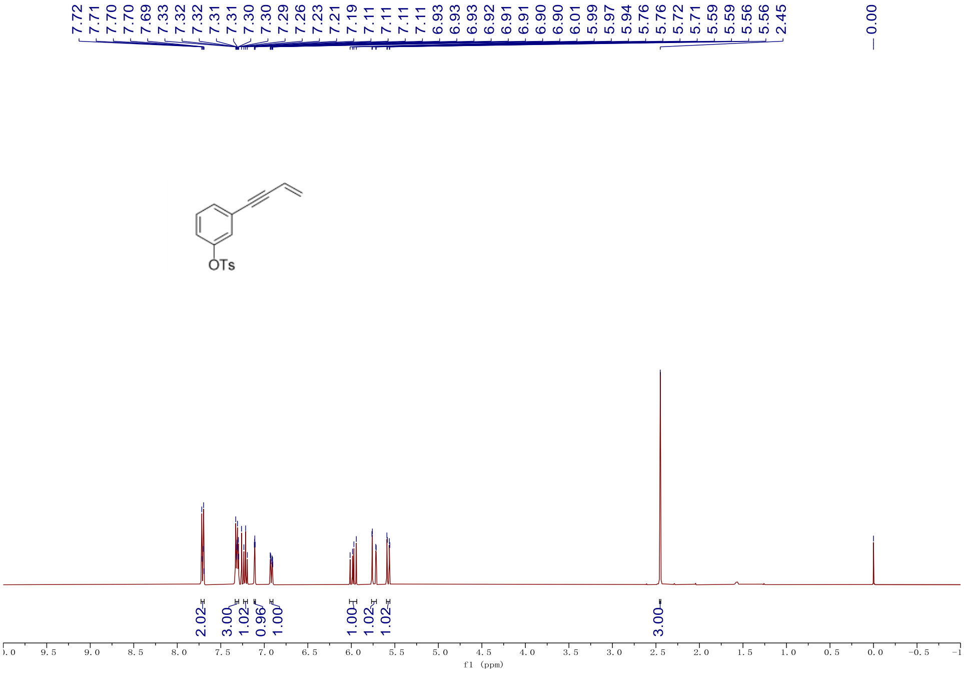

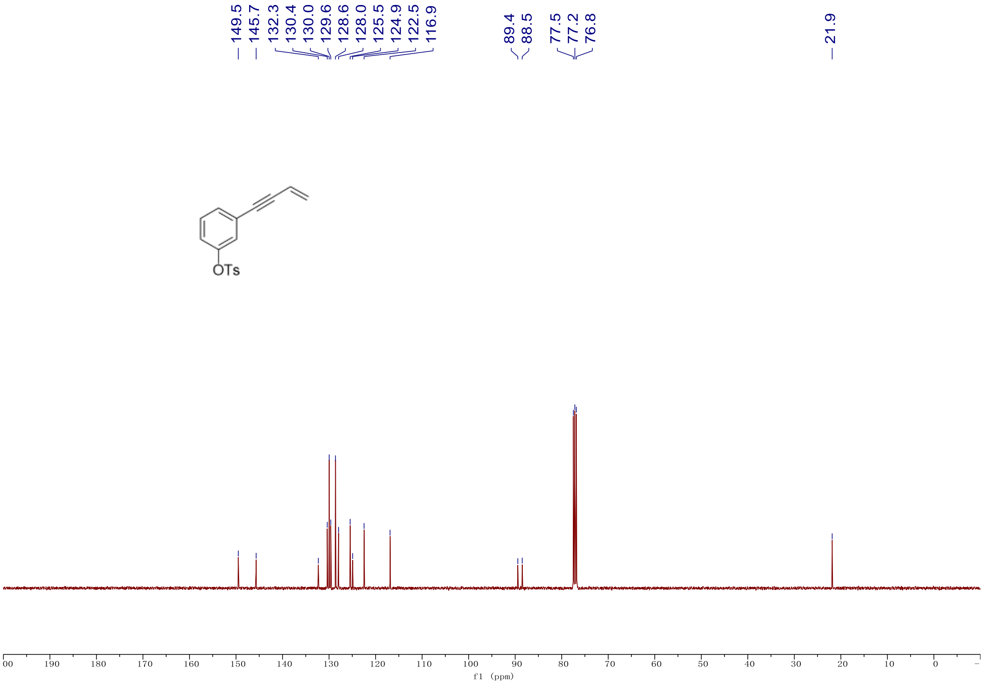

N-(4-(but-3-en-1-yn-1-yl)phenyl)-*N*-methylbenzamide (1p)

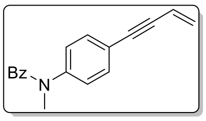

Prepared according to procedure 1 as white solid in 70% yield (3.0 mmol, 783.3 mg,).

R*f*: 0.45 (ethyl acetate : petroleum ether = 1:5)

HRMS (ESI) (m/z): Calcd for $C_{18}H_{16}NO$ $[M+H]^+$: 262.1232, found: 262.1238.

^{1}H NMR (400 MHz, $CDCl_3$) δ 7.31 – 7.27 (m, 4H), 7.26 – 7.23 (m, 1H), 7.21 – 7.15 (m, 2H), 6.99 – 6.95 (m, 2H), 5.98 (dd, *J* = 17.5, 11.2 Hz, 1H), 5.71 (dd, *J* = 17.5, 2.0 Hz, 1H), 5.54 (dd, *J* = 11.2, 2.0 Hz, 1H), 3.49 (s, 3H).

^{13}C NMR (101 MHz, $CDCl_3$) δ 170.7, 144.9, 135.7, 132.5, 130.0, 128.9, 128.0, 127.5, 126.8, 121.3, 117.0, 89.1, 88.9, 38.3.

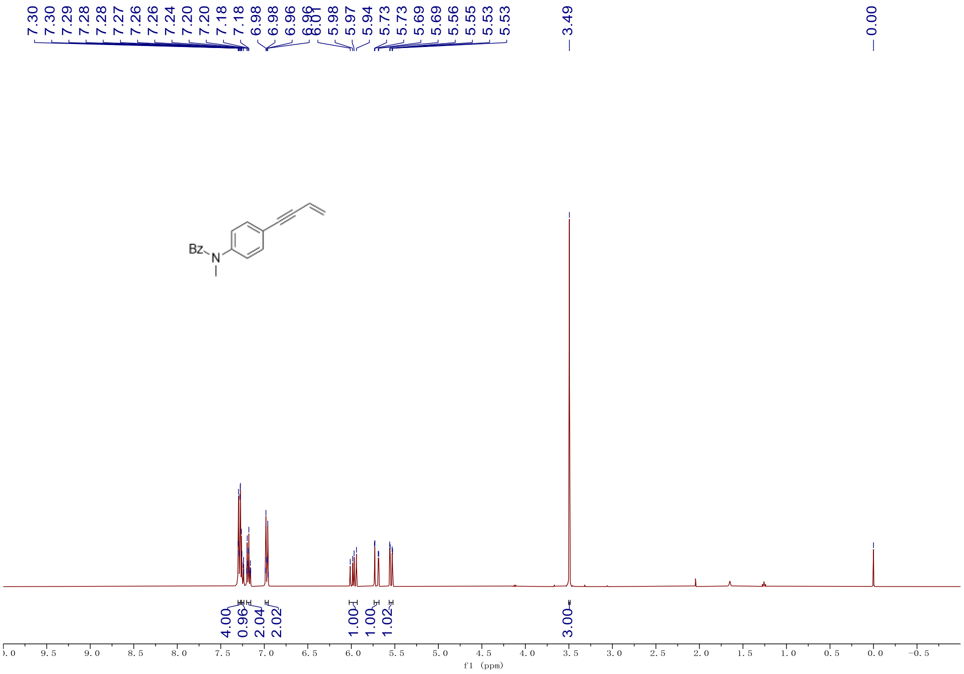

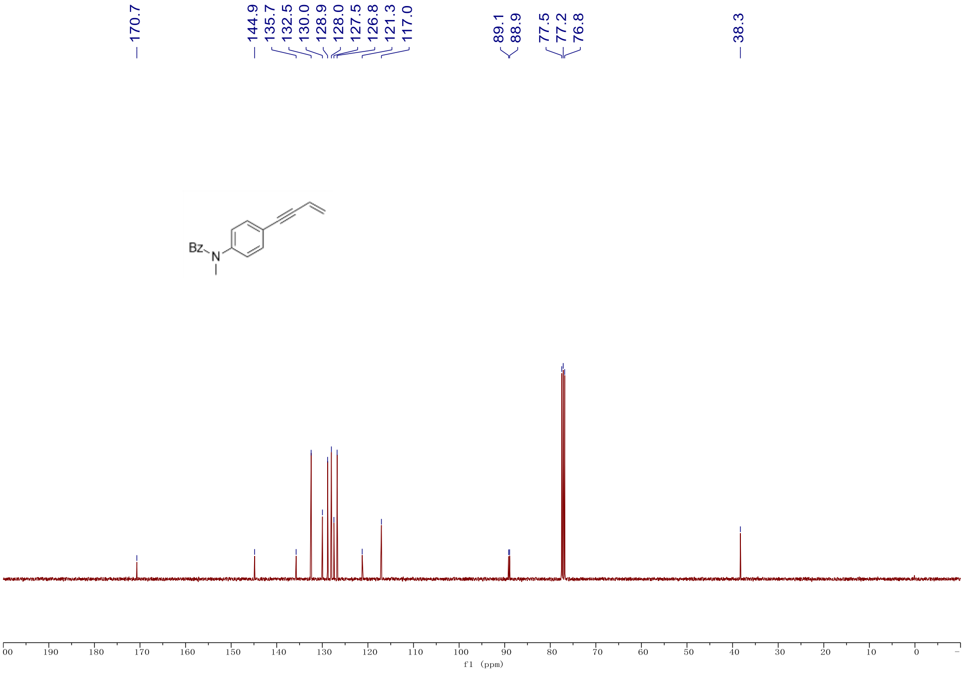

1-(But-3-en-1-yn-1-yl)-4-vinylbenzene (1v)

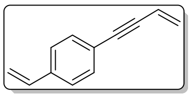

Prepared according to procedure 1 as colorless oil in 65% yield (2.6 mmol, 400.9 mg).

R$_f$: 0.80 (petroleum ether).

HRMS (ESI) (m/z): Calcd for $C_{12}H_{11}$ $[M+H]^+$: 155.0861, found: 155.0858.

^{1}H NMR (400 MHz, $CDCl_3$) δ 7.41 (d, J = 8.2 Hz, 2H), 7.36 (d, J = 8.2 Hz, 2H), 6.70 (dd, J = 17.6, 11.2 Hz, 1H), 6.03 (dd, J = 17.6, 11.2 Hz, 1H), 5.80 – 5.71 (m, 2H), 5.55 (dd, J = 11.2, 2.0 Hz, 1H), 5.29 (d, J = 10.8 Hz, 1H).

^{13}C NMR (101 MHz, $CDCl_3$) δ 137.6, 136.4, 131.9, 127.0, 126.3, 122.5, 117.3, 114.9, 90.2, 88.9.

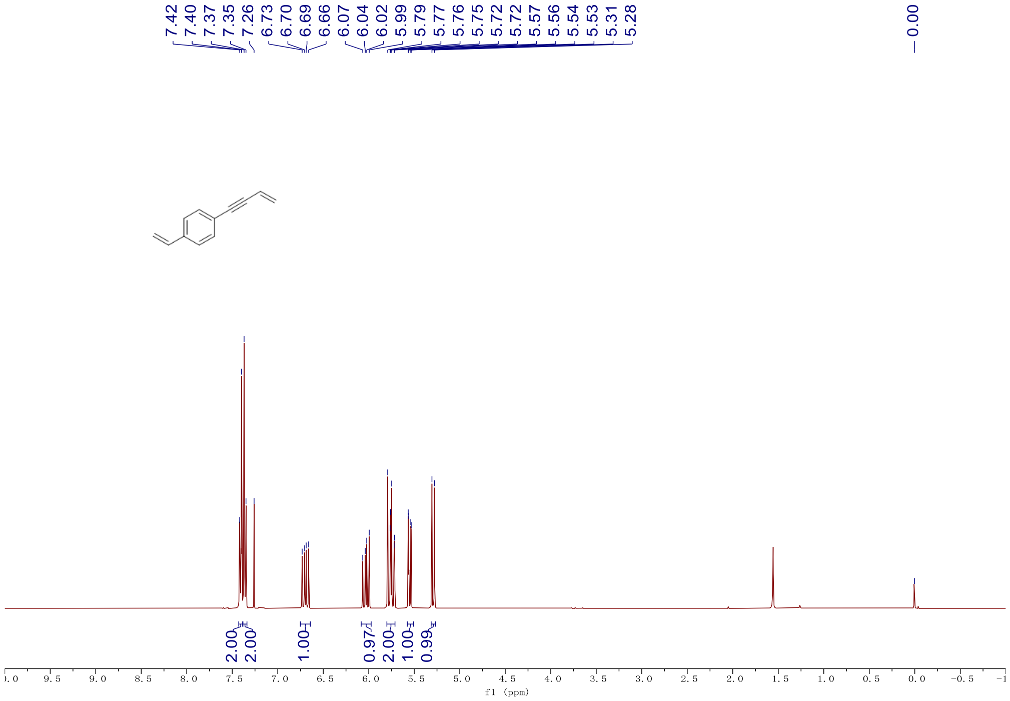

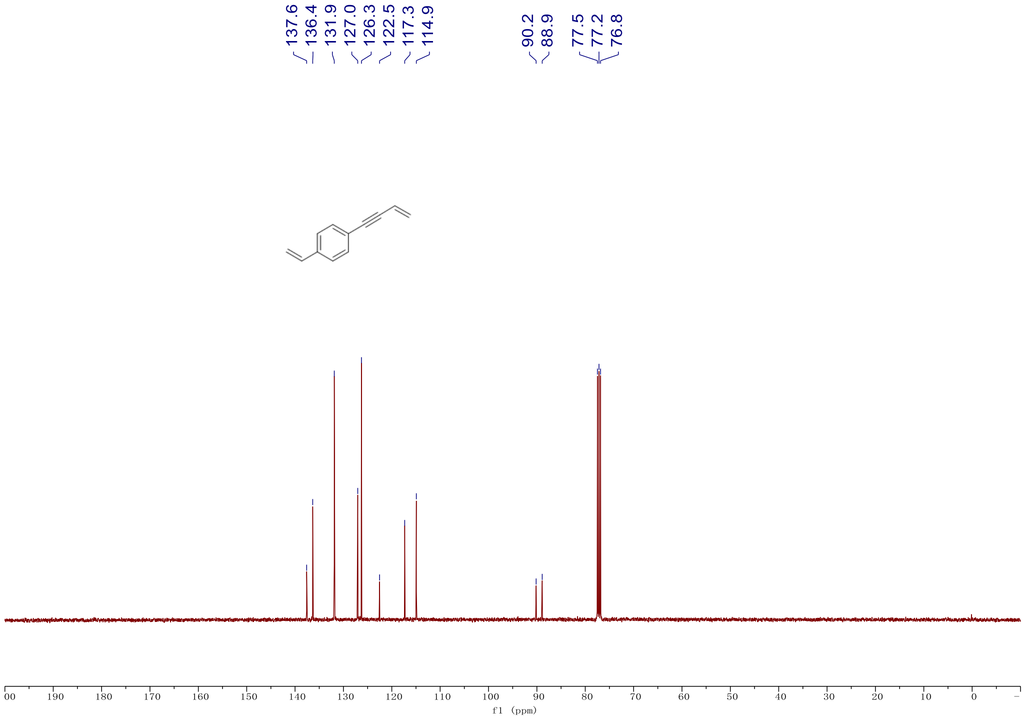

1-(But-3-en-1-yn-1-yl)-4-(prop-1-en-2-yl)benzene (1w)

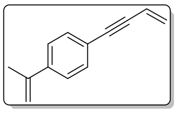

Prepared according to procedure 1 as colorless oil in 55% yield (2.2 mmol, 370.0 mg).

R*f*: 0.81 (petroleum ether).

HRMS (ESI) (m/z): Calcd for $C_{13}H_{13}$ $[M+H]^+$: 169.1017, found: 169.1018.

^{1}H NMR (400 MHz, $CDCl_3$) δ 7.45 – 7.38 (m, 4H), 6.03 (dd, *J* = 17.5, 11.1 Hz, 1H), 5.73 (d, *J* = 17.5 Hz, 1H), 5.54 (dd, *J* = 11.2, 2.1 Hz, 1H), 5.41 (d, *J* = 0.8 Hz, 1H), 5.12 (d, *J* = 1.5 Hz, 1H), 2.14 (s, 3H).

^{13}C NMR (101 MHz, $CDCl_3$) δ 142.6, 141.1, 131.6, 127.0, 125.5, 122.2, 117.4, 113.4, 90.1, 88.7, 21.7.

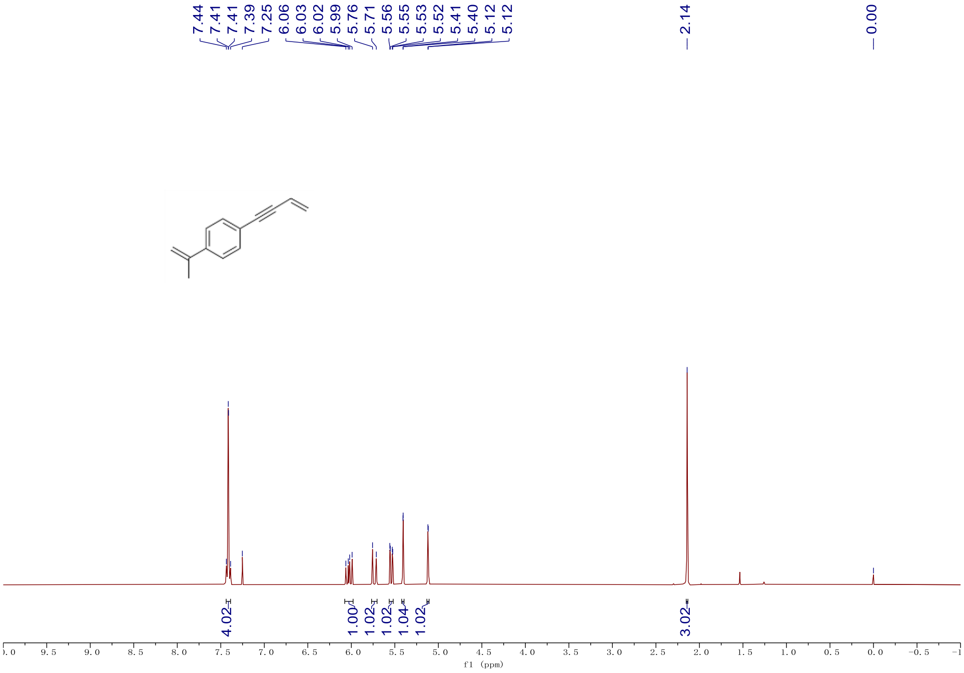

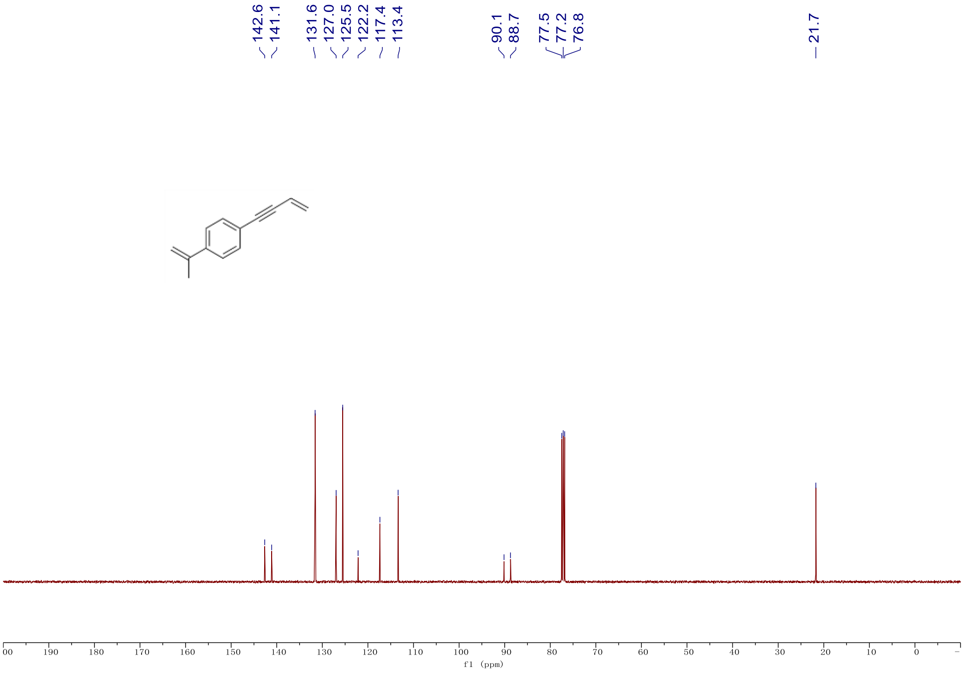

1-(But-3-en-1-yn-1-yl)-4-ethynylbenzene (1x)

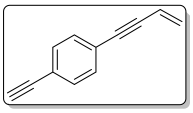

Prepared by the desilylation of **1y** in the presence of K_2CO_3 (2.0 equiv) in MeOH (0.5 M) for 4 h. **1x** was obtained in 65% yield (2.4 mmol, 365.0 mg).

R*f*: 0.80 (petroleum ether).

HRMS (ESI) (m/z): Calcd for $C_{12}H_9$ $[M+H]^+$: 153.0704, found: 153.0718.

^{1}H NMR (400 MHz, $CDCl_3$) δ 7.47 – 7.35 (m, 4H), 6.02 (dd, *J* = 17.5, 11.2 Hz, 1H), 5.75 (dd, *J* = 17.5, 2.0 Hz, 1H), 5.57 (dd, *J* = 11.2 , 2.0 Hz, 1H), 3.16 (s, 1H).

^{13}C NMR (101 MHz, $CDCl_3$) δ 132.2, 131.6, 127.6, 123.8, 122.0, 117.1, 90.2, 89.5, 83.4, 79.0.

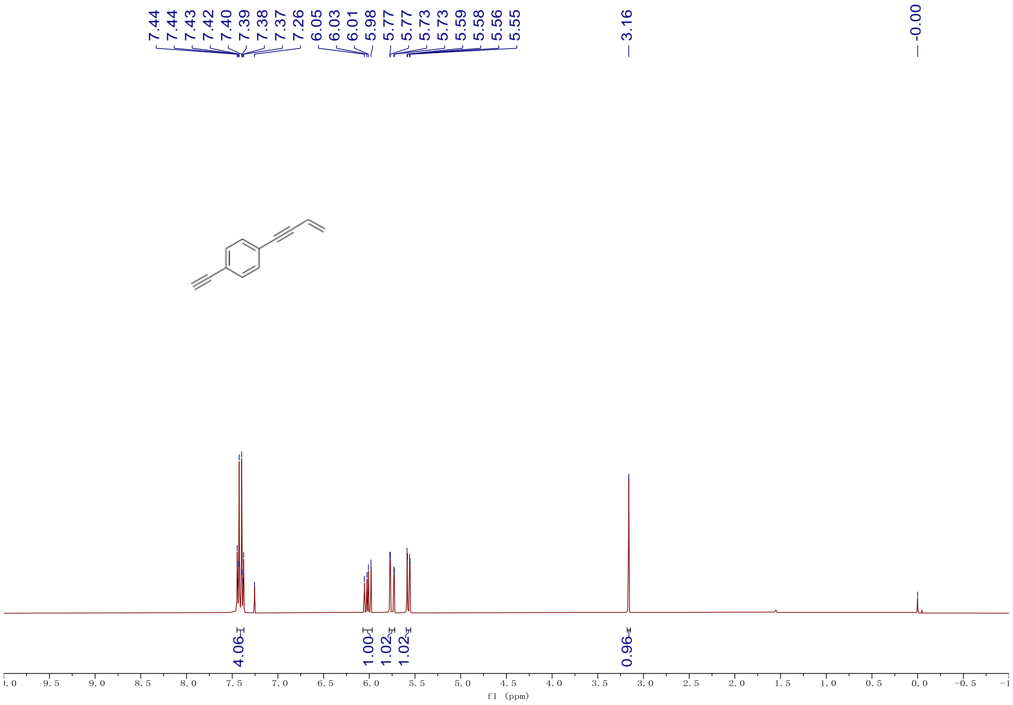

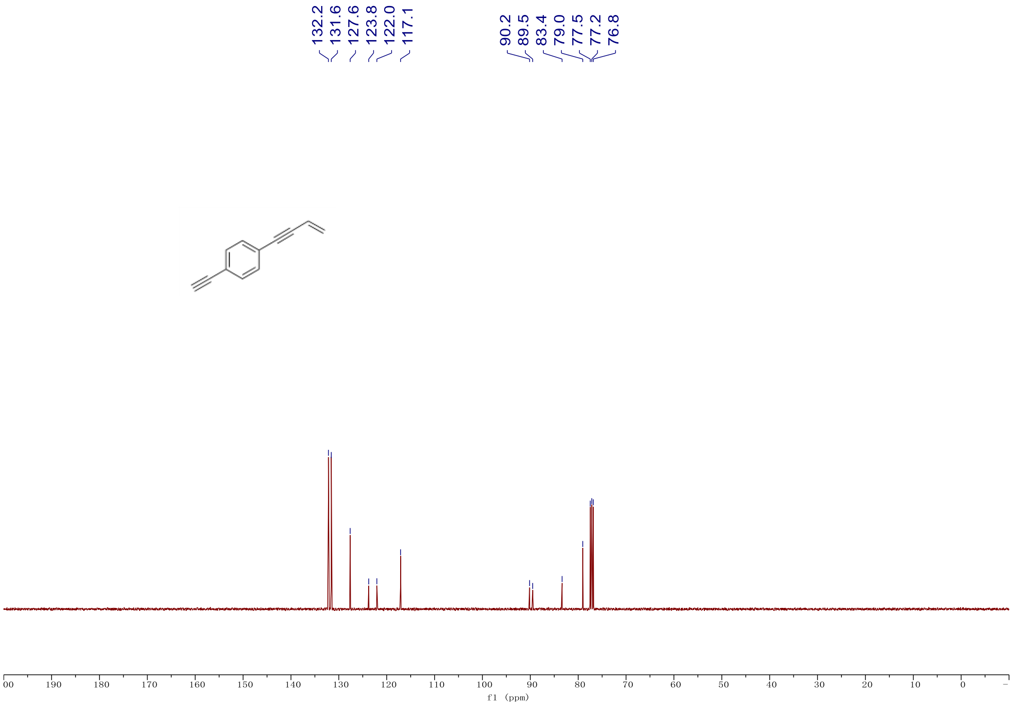

((4-(But-3-en-1-yn-1-yl)phenyl)ethynyl)trimethylsilane (1y)

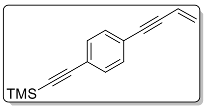

Prepared according to procedure 1 as colorless oil in 62% yield (3.1 mmol, 694.7 mg).

R*f*: 0.75 (petroleum ether).

HRMS (ESI) (m/z): Calcd for $C_{15}H_{17}Si$ $[M+H]^{+}$: 225.1100, found: 225.1095.

^{1}H NMR (400 MHz, $CDCl_3$) δ 7.43 – 7.34 (m, 4H), 6.01 (dd, *J* = 17.5, 11.2 Hz, 1H), 5.74 (dd, *J* = 17.6, 2.0 Hz, 1H), 5.56 (dd, *J* = 11.1, 2.1 Hz, 1H), 0.25 (s, 9H).

^{13}C NMR (101 MHz, $CDCl_3$) δ 132.0, 131.5, 127.5, 123.3, 123.1, 117.1, 104.7, 96.4, 90.1, 89.7, 0.1.

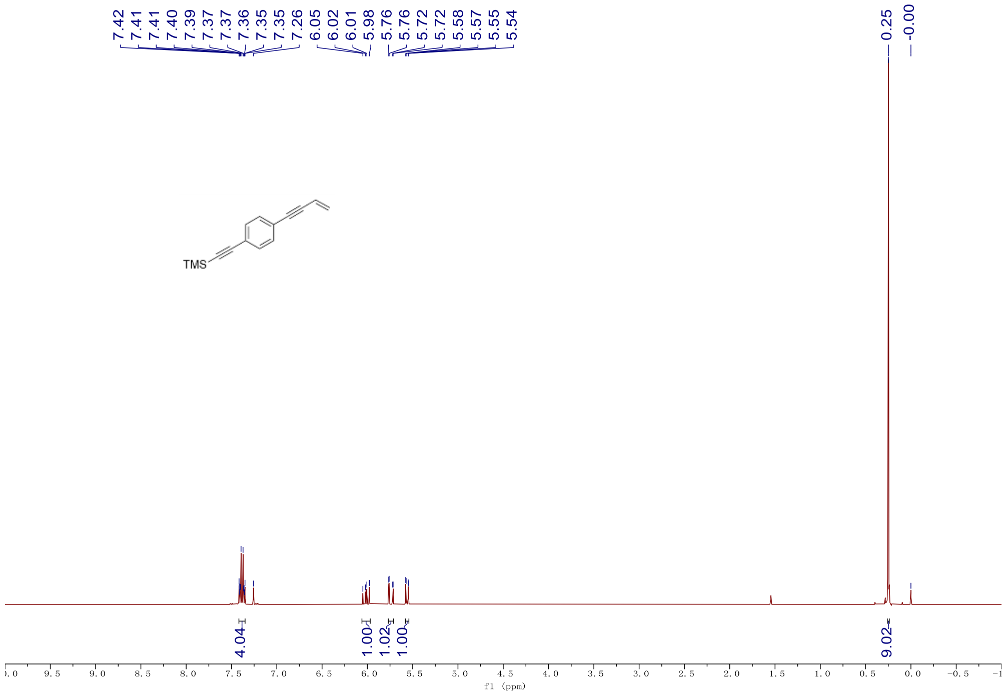

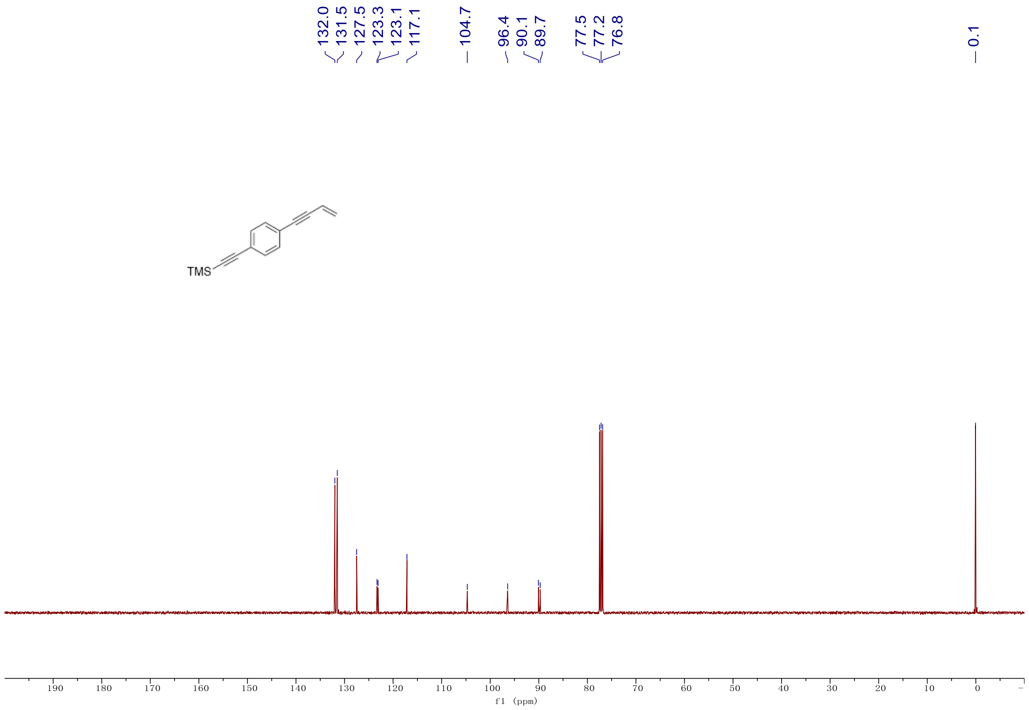

(*E*)-1-(but-3-en-1-yn-1-yl)cyclooct-1-ene (1aa)

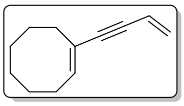

Prepared according to procedure 1 as colorless oil in 75% yield (4.2 mmol, 672.5 mg).

R*f*: 0.80 (petroleum ether).

HRMS (ESI) (m/z): Calcd for $C_{12}H_{17}$ $[M+H]^+$: 161.1330, found: 161.1329.

^{1}H NMR (400 MHz, $CDCl_3$) δ 6.10 (t, *J* = 8.4 Hz, 1H), 5.91 (dd, *J* = 17.6, 11.2 Hz, 1H), 5.58 (dd, *J* = 17.6, 2.0 Hz, 1H), 5.41 (dd, *J* = 11.2, 2.0 Hz, 1H), 2.33 – 2.29 (m, 2H), 2.21 – 2.15 (m, 2H), 1.63 – 1.57 (m, 2H), 1.54 – 1.47 (m, 6H).

^{13}C NMR (101 MHz, $CDCl_3$) δ 138.3, 125.7, 123.9, 117.7, 92.9, 85.3, 30.0, 29.8, 28.6, 27.2, 26.5, 26.0.

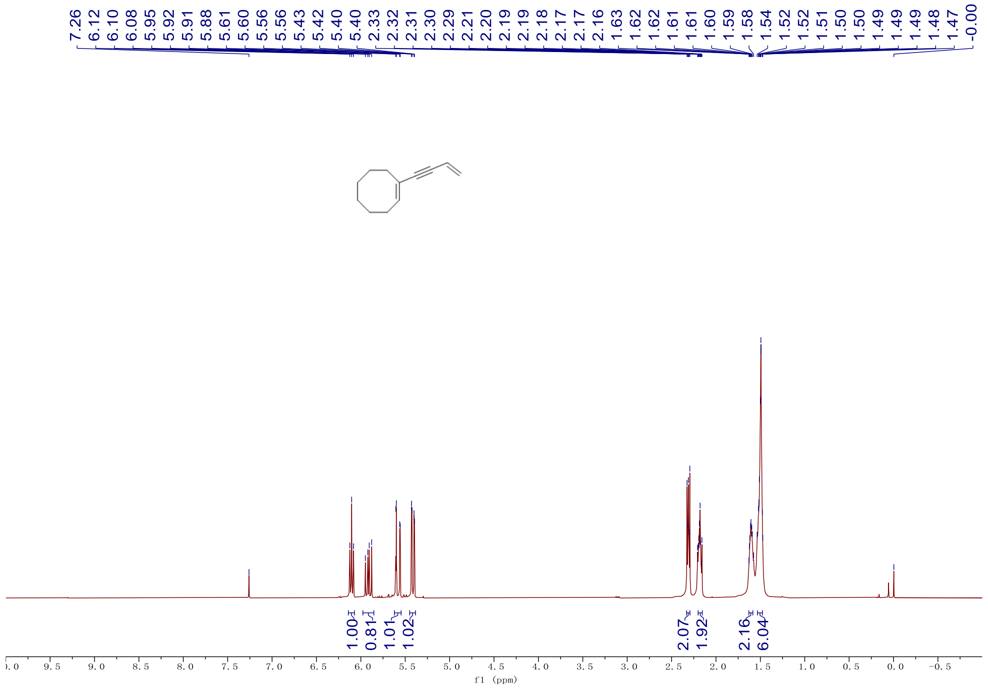

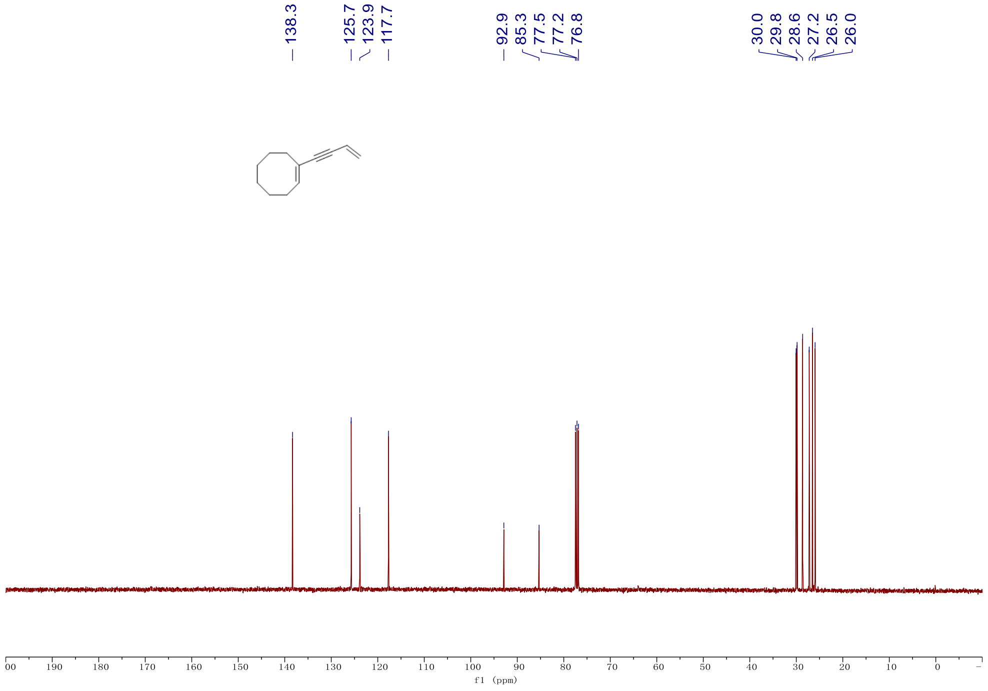

(2-(Cyclopentyloxy)phenyl)silane (2k)

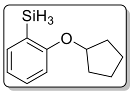

Prepared according to reported literature[1].

HRMS (ESI) (m/z): Calcd for $C_{11}H_{17}OSi$ $[M+H]^+$: 193.1047, found: 193.1066.

^{1}H NMR (400 MHz, $CDCl_3$) δ 7.49 (d, *J* = 7.3 Hz, 1H), 7.37 (t, *J* = 7.8 Hz, 1H), 6.91 (t, *J* = 7.3 Hz, 1H), 6.82 (d, *J* = 8.3 Hz, 1H), 4.84 – 4.79 (m, 1H), 4.07 (s, 3H), 1.90 – 1.79 (m, 6H), 1.67 – 1.59 (m, 2H).

^{13}C NMR (101 MHz, $CDCl_3$) δ 163.0, 138.0, 132.1, 120.5, 118.4, 111.5, 79.4, 33.0, 24.1.

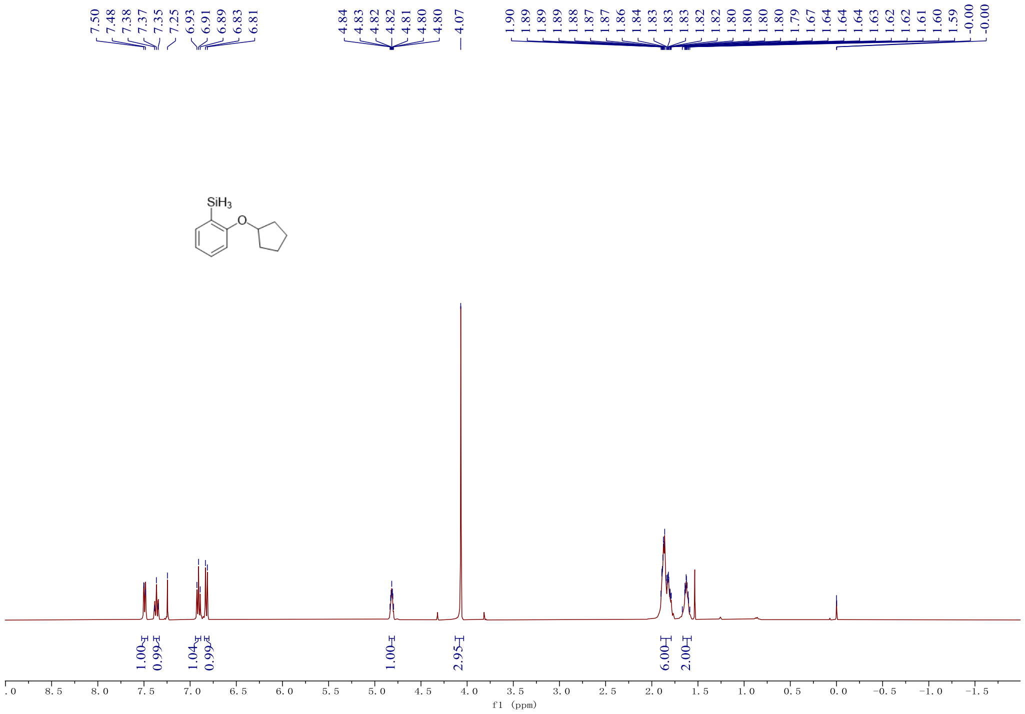

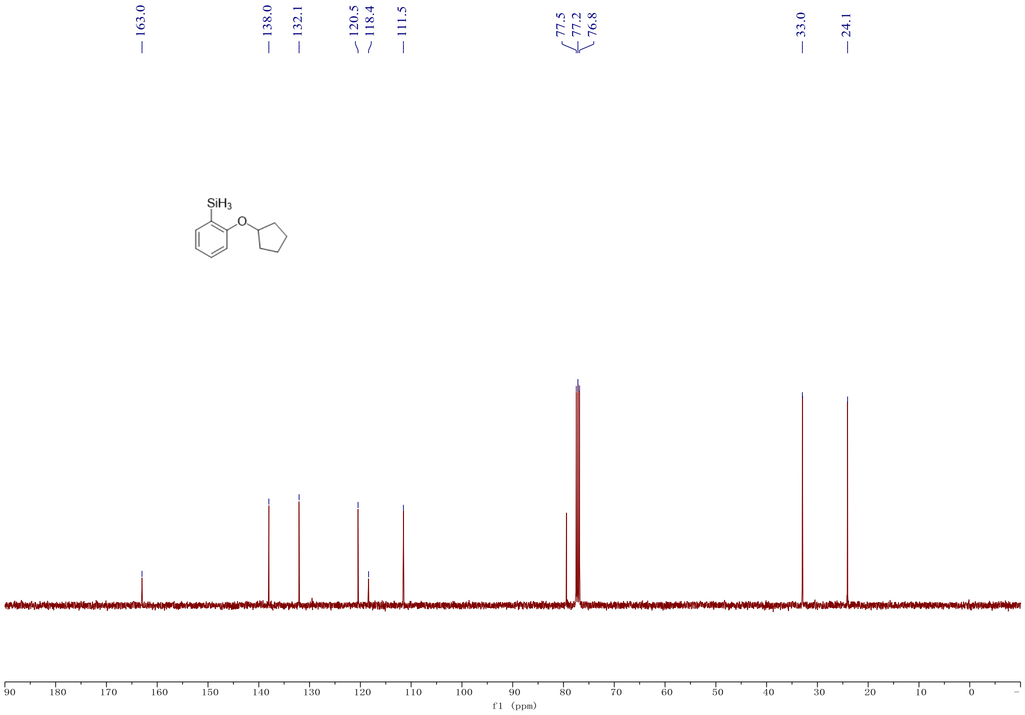

Octyl(1-phenylbuta-1,2-dien-1-yl)silane (3ab)

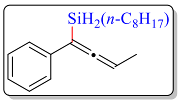

Prepared according to procedure 2 from **1a** (0.2 mmol, 25.6 mg) and **2b** (0.6 mmol, 86.4 mg). The reaction mixture was stirred at 40 °C for 1 h in 1.0 mL dry DCE. The product was isolated in 78% yield (42.5 mg) as colorless oil.

R*f*: 0.80 (petroleum ether).

HRMS (ESI) (m/z): Calcd for $C_{18}H_{29}Si$ $[M+H]^+$: 273.2039, found: 273.2026.

^{1}H NMR (400 MHz, $CDCl_3$) δ 7.38 – 7.35 (m, 2H), 7.33 – 7.28 (m, 2H), 7.21 – 7.17 (m, 1H), 5.22 (q, *J* = 7.2 Hz, 1H), 4.31 – 4.21 (m, 2H), 1.75 (d, *J* = 7.2 Hz, 3H), 1.49 – 1.42 (m, 2H), 1.38 – 1.22 (m, 10H), 0.91 – 0.84 (m, 5H).

^{13}C NMR (101 MHz, $CDCl_3$) δ 210.5, 137.4, 128.7, 127.3, 126.6, 92.9, 82.9, 32.9, 32.0, 29.4, 29.4, 25.2, 22.8, 14.3, 13.4, 9.7.

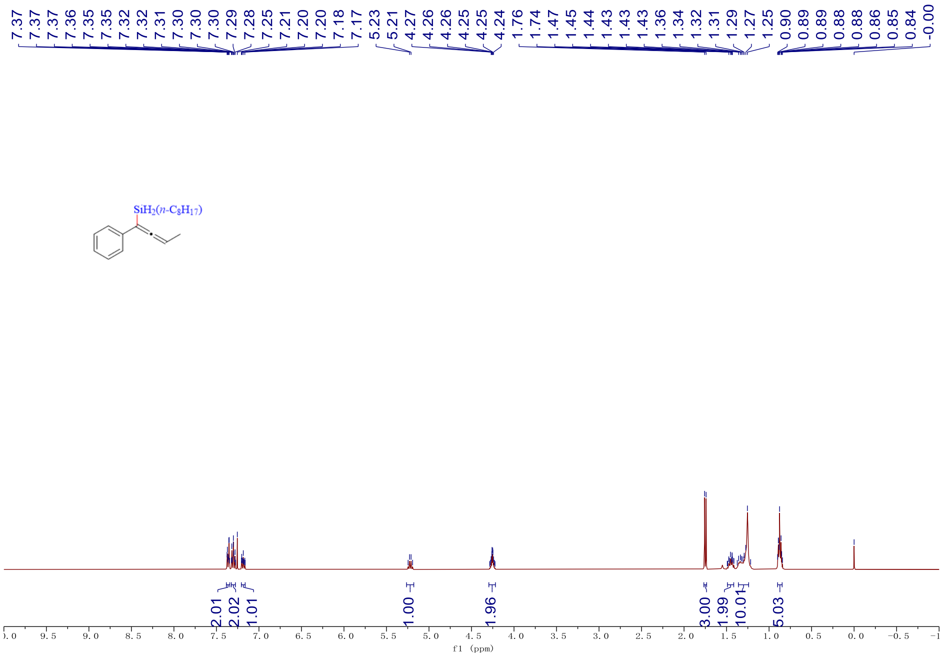

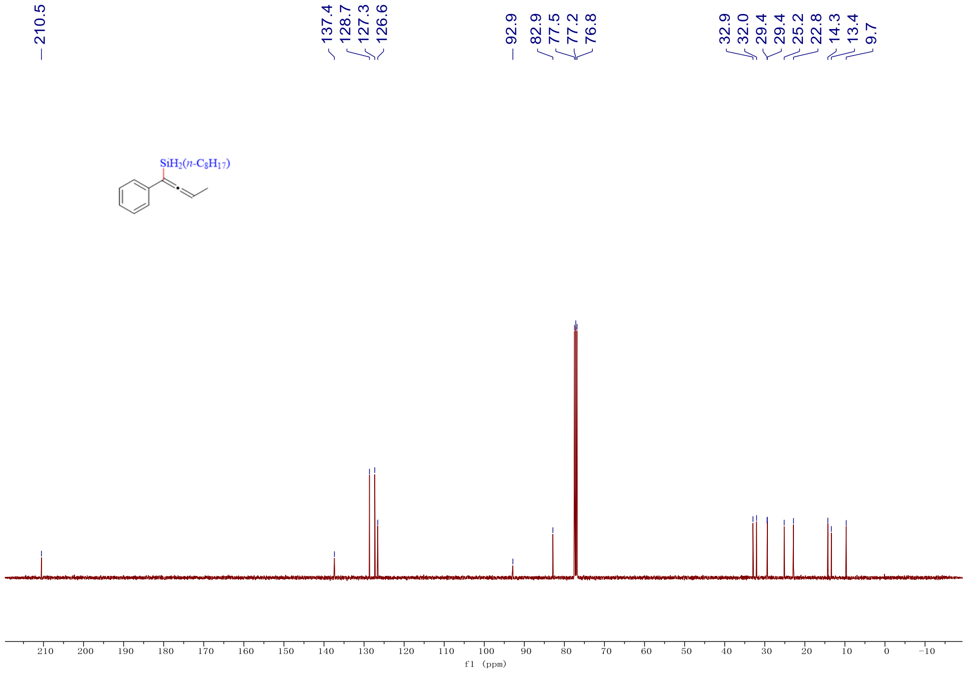

Octyl(1-(*m*-tolyl)buta-1,2-dien-1-yl)silane (3bb)

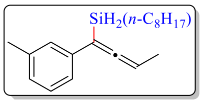

Prepared according to procedure 2 from **1b** (0.2 mmol, 28.4 mg) and **2b** (0.6 mmol, 86.4 mg). The reaction mixture was stirred at 60 °C for 1 h in 0.5 mL dry DCE. The product was isolated in 75% yield (43.0 mg) as colorless oil.

R*f*: 0.85 (petroleum ether).

HRMS (ESI) (m/z): Calcd for $C_{19}H_{31}Si$ $[M+H]^+$: 287.2195, found: 287.2194.

^{1}H NMR (400 MHz, $CDCl_3$) δ 7.22 – 7.13 (m, 3H), 7.00 (d, *J* = 7.2 Hz, 1H), 5.20 (q, *J* = 7.0 Hz, 1H), 4.28 – 4.21 (m, 2H), 2.34 (s, 3H), 1.74 (d, *J* = 7.1 Hz, 3H), 1.50 – 1.40 (m, 2H), 1.38 – 1.20 (m, 10H), 0.90 – 0.83 (m, 5H).

^{13}C NMR (101 MHz, $CDCl_3$) δ 210.4, 138.2, 137.3, 128.5, 128.0, 127.4, 124.4, 92.8, 82.7, 32.9, 32.0, 29.4, 29.4, 25.2, 22.8, 21.6, 14.3, 13.4, 9.7.

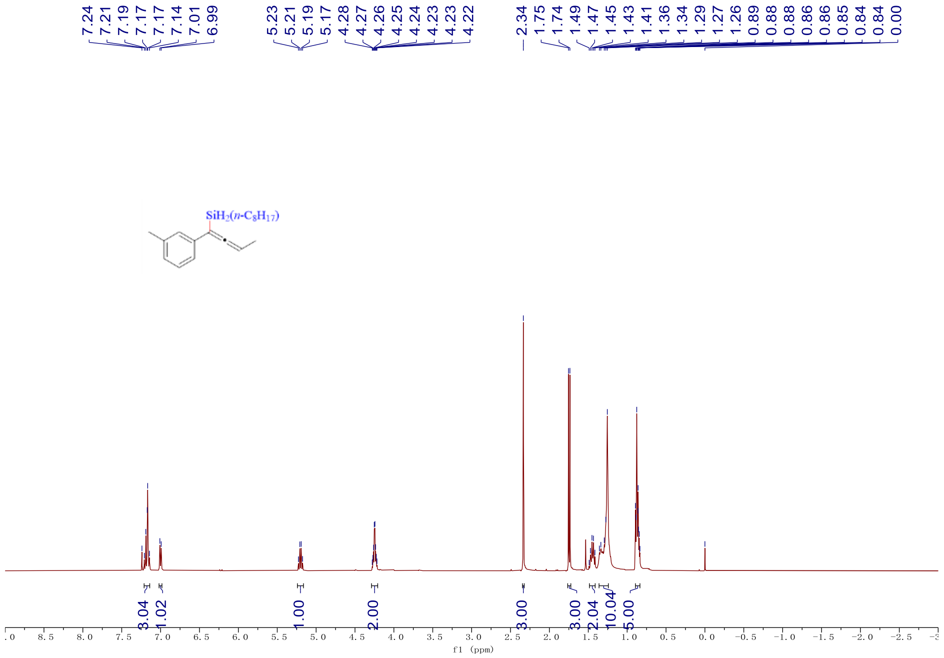

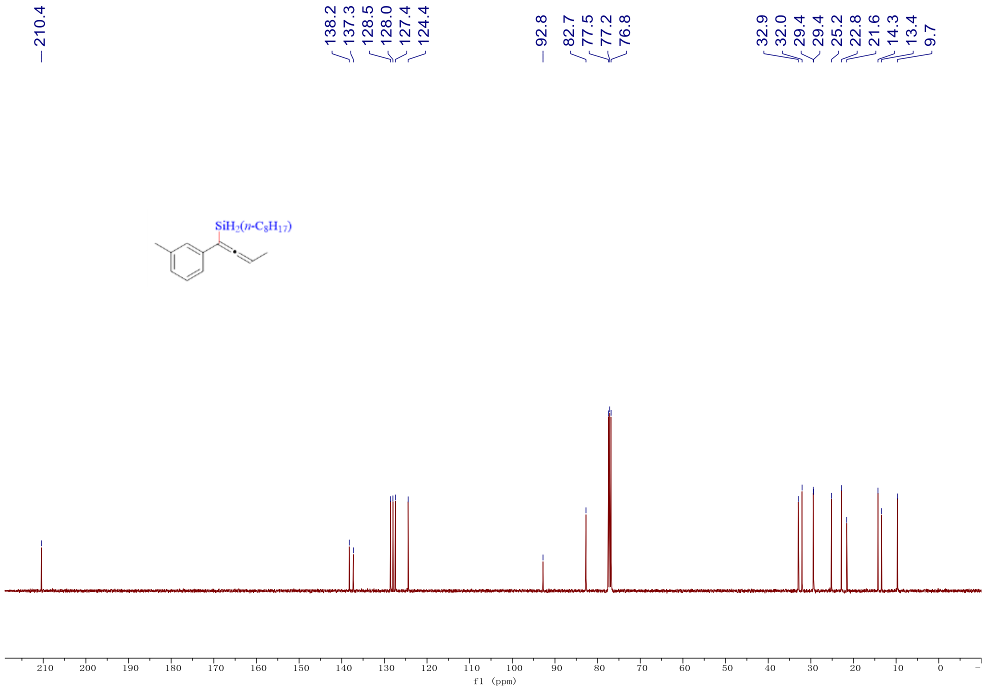

Octyl(1-(*p*-tolyl)buta-1,2-dien-1-yl)silane (3cb)

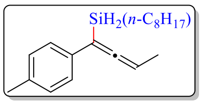

Prepared according to procedure 2 from **1c** (0.2 mmol, 28.4 mg) and **2b** (0.6 mmol, 86.4 mg). The reaction mixture was stirred at 60 °C for 1 h in 0.5 mL dry DCE. The product was isolated in 75% yield (43.2 mg) as colorless oil.

R_f: 0.83 (petroleum ether).

HRMS (ESI) (m/z): Calcd for $C_{19}H_{31}Si$ $[M+H]^+$: 287.2195, found: 287.2190.

^{1}H NMR (400 MHz, $CDCl_3$) δ 7.27 – 7.23 (m, 2H), 7.11 (d, *J* = 7.9 Hz, 2H), 5.19 (q, *J* = 7.1 Hz, 1H), 4.28 – 4.20 (m, 2H), 2.32 (s, 3H), 1.74 (d, *J* = 7.1 Hz, 3H), 1.50 – 1.39 (m, 2H), 1.36 – 1.22 (m, 10H), 0.90 – 0.83 (m, 5H).

^{13}C NMR (101 MHz, $CDCl_3$) δ 210.2, 136.3, 134.3, 129.4, 127.2, 92.5, 82.8, 32.9, 32.1, 29.4, 29.4, 25.2, 22.8, 21.2, 14.3, 13.5, 9.7.

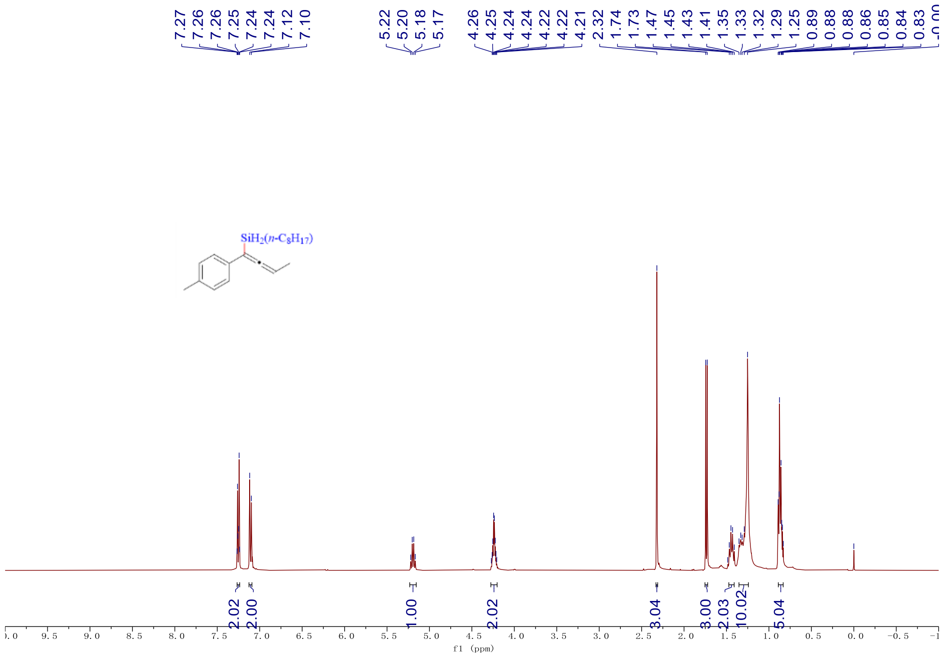

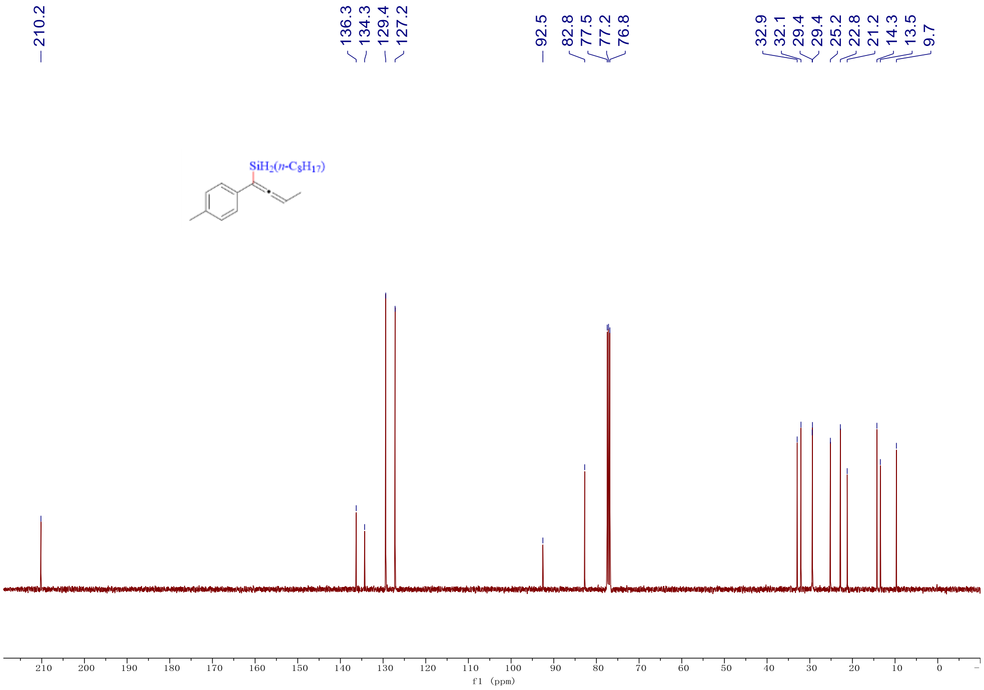

(1-(4-(*Tert*-butyl)phenyl)buta-1,2-dien-1-yl)(octyl)silane (3db)

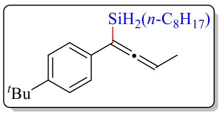

Prepared according to procedure 2 from **1d** (0.2 mmol, 36.9 mg) and **2b** (0.6 mmol, 86.4 mg). The reaction mixture was stirred at 60 °C for 1 h in 0.5 mL dry DCE. The product was isolated in 76% yield (50.0 mg) as colorless oil.

R*f*: 0.75 (petroleum ether).

HRMS (ESI) (m/z): Calcd for $C_{22}H_{37}Si$ $[M+H]^+$: 329.2665, found: 329.2666.

^{1}H NMR (400 MHz, $CDCl_3$) δ 7.35 – 7.28 (m, 4H), 5.20 (q, *J* = 7.1 Hz, 1H), 4.29 – 4.21 (m, 2H), 1.73 (d, *J* = 7.1 Hz, 3H), 1.50 – 1.41 (m, 2H), 1.39 – 1.22 (m, 19H), 0.90 – 0.84 (m, 5H).

^{13}C NMR (101 MHz, $CDCl_3$) δ 210.4, 149.6, 134.3, 127.0, 125.6, 92.4, 82.8, 34.6, 33.0, 32.1, 31.5, 29.4, 29.4, 25.2, 22.8, 14.3, 13.5, 9.7.

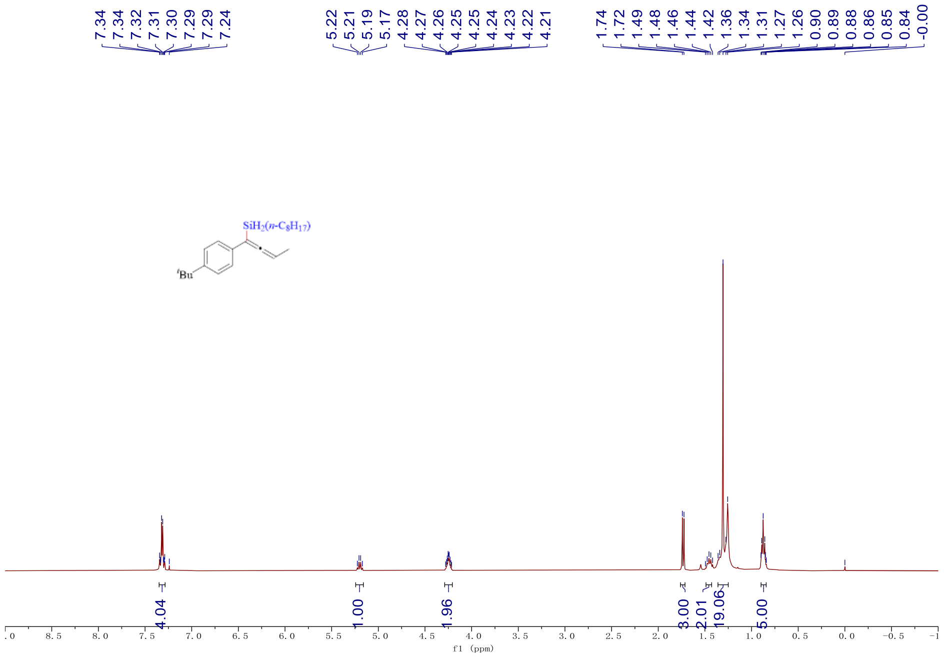

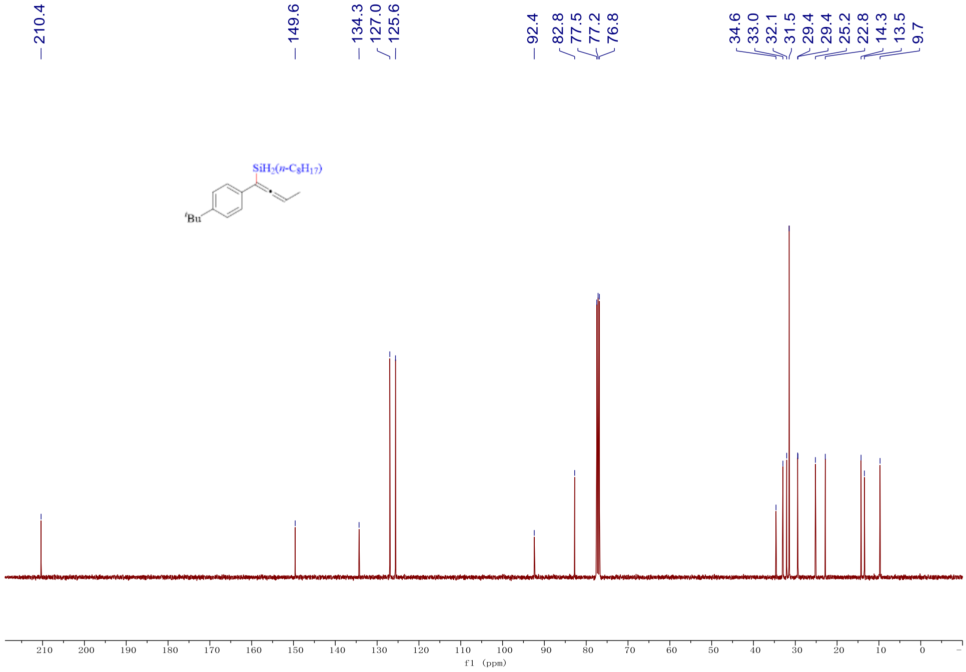

(1-([1,1'-Biphenyl]-4-yl)buta-1,2-dien-1-yl)(octyl)silane (3eb)

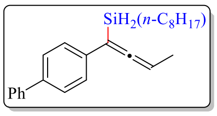

Prepared according to procedure 2 from **1e** (0.2 mmol, 40.9 mg) and **2b** (0.6 mmol, 86.4 mg). The reaction mixture was stirred at 40 °C for 1 h in 0.5 mL dry DCE. The product was isolated in 82% yield (57.2 mg) as colorless oil.

R*f*: 0.77 (petroleum ether).

HRMS (ESI) (m/z): Calcd for $C_{24}H_{33}Si$ $[M+H]^+$: 349.2352, found: 349.2365.

^{1}H NMR (400 MHz, $CDCl_3$) δ 7.62 – 7.51 (m, 4H), 7.47 – 7.38 (m, 4H), 7.37 – 7.28 (m, 1H), 5.26 (q, *J* = 7.1 Hz, 1H), 4.28 (m, 2H), 1.77 (d, *J* = 7.1 Hz, 3H), 1.47 (m, 2H), 1.41 – 1.22 (m, 10H), 0.93 – 0.85 (m, 5H).

^{13}C NMR (101 MHz, $CDCl_3$) δ 210.7, 141.0, 139.5, 136.5, 128.9, 127.7, 127.4, 127.3, 127.1, 92.5, 83.1, 33.0, 32.1, 29.4, 29.4, 25.2, 22.8, 14.3, 13.4, 9.7.

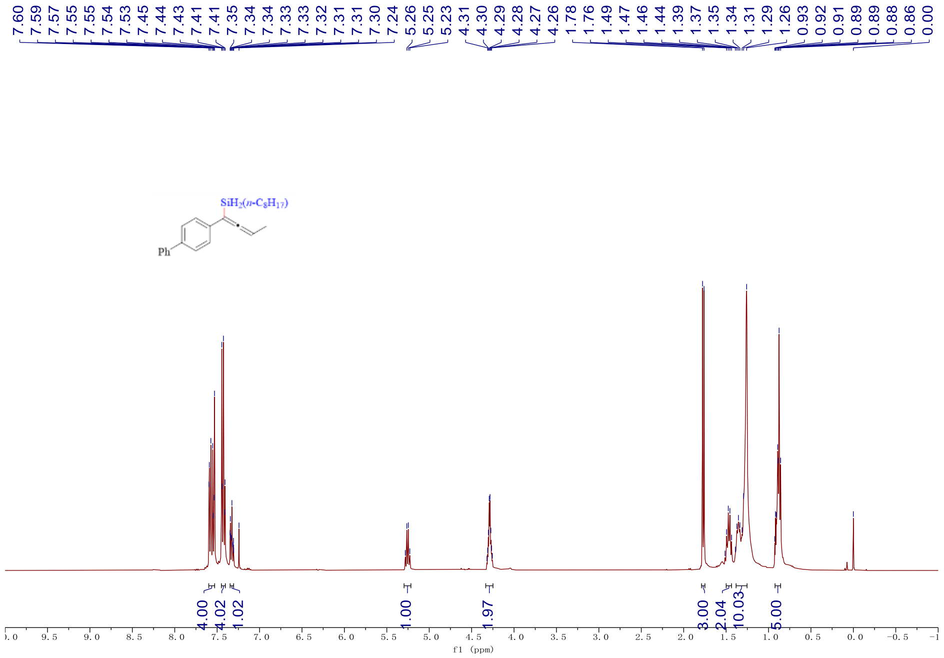

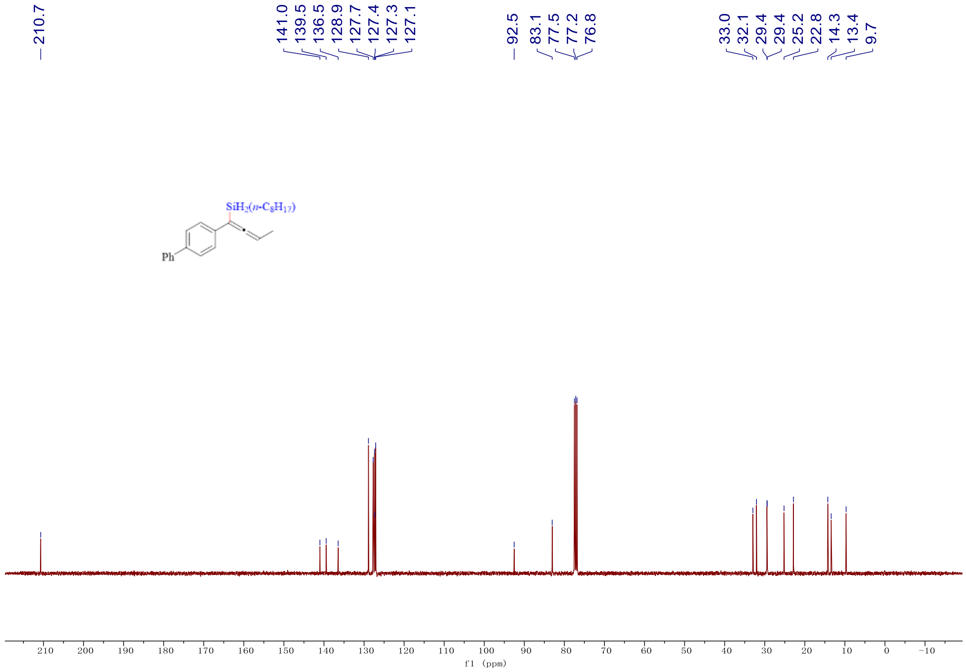

(1-(Naphthalen-2-yl)buta-1,2-dien-1-yl)(octyl)silane (3fb)

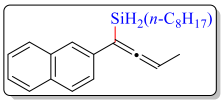

Prepared according to procedure 2 from **1f** (0.2 mmol, 35.6 mg) and **2b** (0.6 mmol, 86.4 mg). The reaction mixture was stirred at 60 °C for 1 h in 1.0 mL dry DCE. The product was isolated in 77% yield (49.8 mg) as colorless oil.

R*f*: 0.70 (petroleum ether).

HRMS (ESI) (m/z): Calcd for $C_{22}H_{31}Si$ $[M+H]^+$: 323.2195, found: 323.2190.

^{1}H NMR (400 MHz, $CDCl_3$) δ 7.81 – 7.74 (m, 3H), 7.72 (s, 1H), 7.56 (dd, *J* = 8.5, 1.8 Hz, 1H), 7.47 – 7.39 (m, 2H), 5.30 (q, *J* = 7.0 Hz, 1H), 4.40 – 4.32 (m, 2H), 1.80 (d, *J* = 7.1 Hz, 3H), 1.53 – 1.43 (m, 2H), 1.40 – 1.22 (m, 10H), 0.96 – 0.89 (m, 2H), 0.87 (t, *J* = 6.8 Hz, 3H).

^{13}C NMR (101 MHz, $CDCl_3$) δ 211.1, 134.8, 133.8, 132.4, 128.2, 128.0, 127.7, 126.2, 125.9, 125.7, 125.6, 93.1, 83.3, 32.9, 32.0, 29.4, 29.4, 25.2, 22.8, 14.3, 13.5, 9.7.

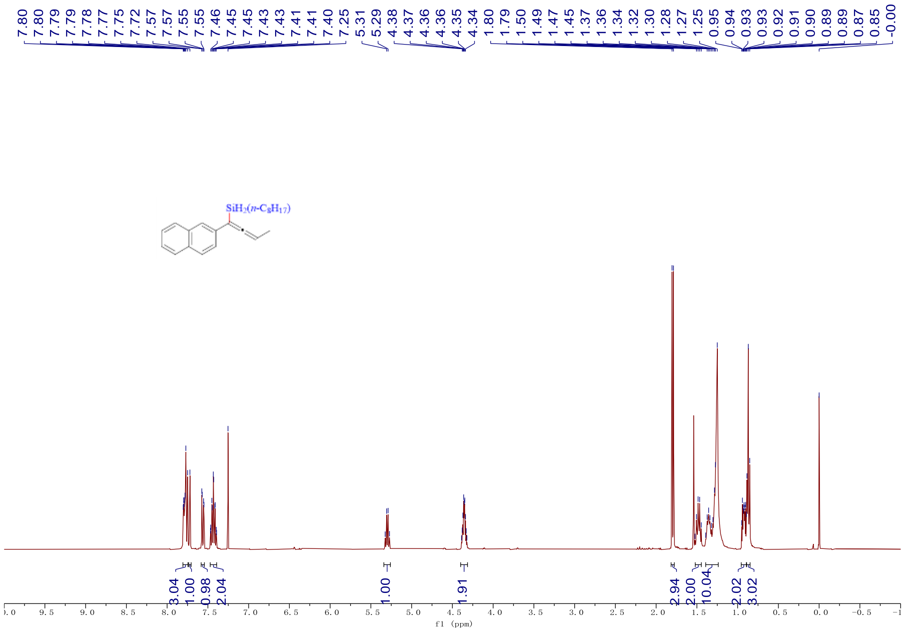

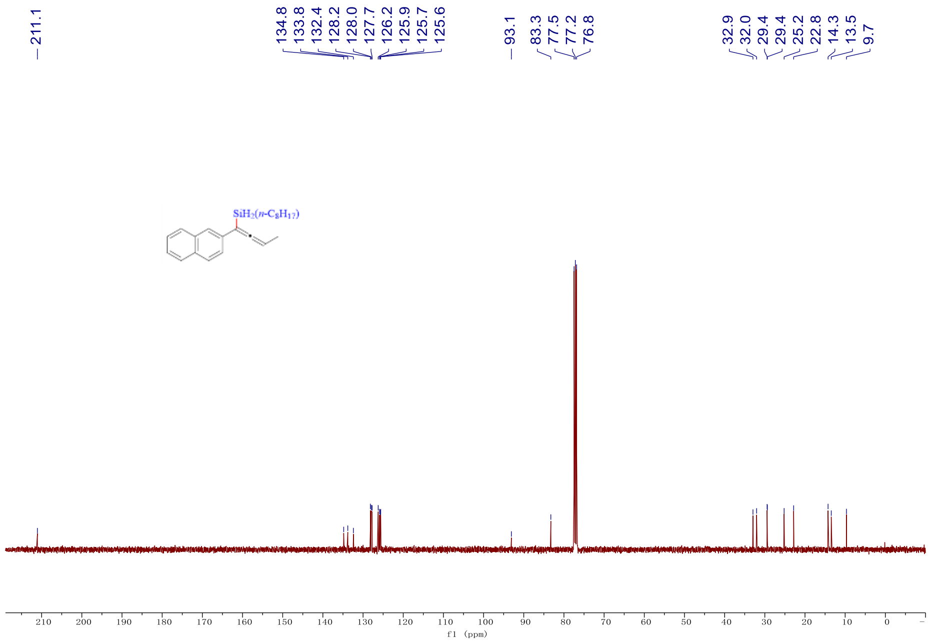

(1-(4-Fluorophenyl)buta-1,2-dien-1-yl)(octyl)silane (3gb)

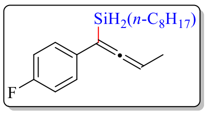

Prepared according to procedure 2 from **1g** (0.2 mmol, 29.2 mg) and **2b** (0.6 mmol, 86.4 mg). The reaction mixture was stirred at 40 °C for 1 h in 0.5 mL dry DCE. The product was isolated in 75% yield (43.7 mg) as colorless oil.

R*f*: 0.90 (petroleum ether).

HRMS (ESI) (m/z): Calcd for $C_{18}H_{28}FSi$ $[M+H]^+$: 291.1944, found: 291.1938.

^{1}H NMR (400 MHz, $CDCl_3$) δ 7.34 – 7.28 (m, 2H), 7.02 – 6.96 (m, 2H), 5.22 (q, *J* = 7.2 Hz, 1H), 4.27 – 4.20 (m, 2H), 1.75 (d, *J* = 7.1 Hz, 3H), 1.49 – 1.40 (m, 2H), 1.38 – 1.22 (m, 10H), 0.90 – 0.83 (m, 5H).

^{13}C NMR (101 MHz, $CDCl_3$) δ 210.3, 161.8 (d, *J* = 245.5 Hz), 133.3 (d, *J* = 3.0 Hz), 128.7 (d, *J* = 8.0 Hz), 115.5 (d, *J* = 21.4 Hz), 92.0, 83.1, 32.9, 32.0, 29.4, 29.3, 25.1, 22.8, 14.3, 13.4, 9.6.

^{19}F NMR (376 MHz, $CDCl_3$) δ -116.4.

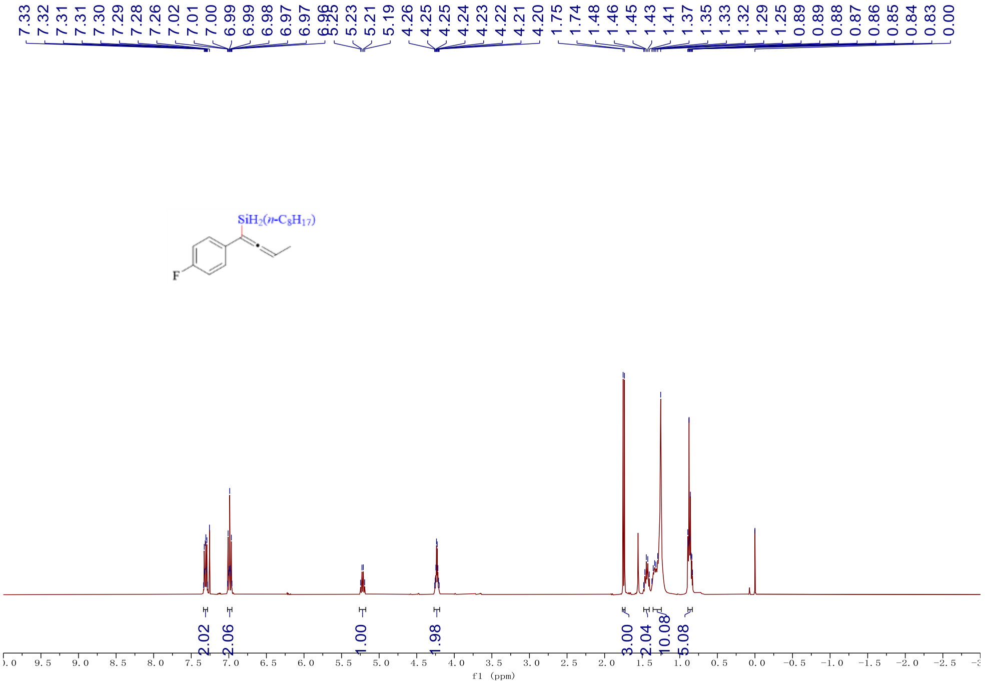

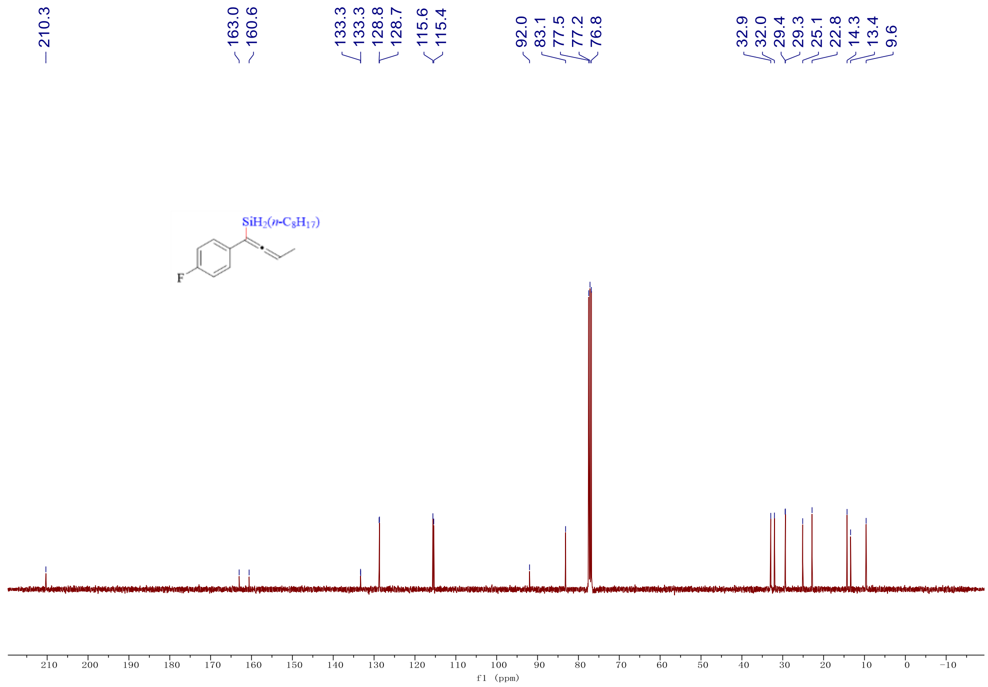
210.3
163.0
160.6
133.3
133.3
128.8
128.7
115.6
115.4
92.0
83.1
77.5
77.2
76.8
32.9
32.0
29.4
29.3
25.1
22.8
14.3
13.4
9.6
SiH2(n-C8H17)
F
f1 (ppm)

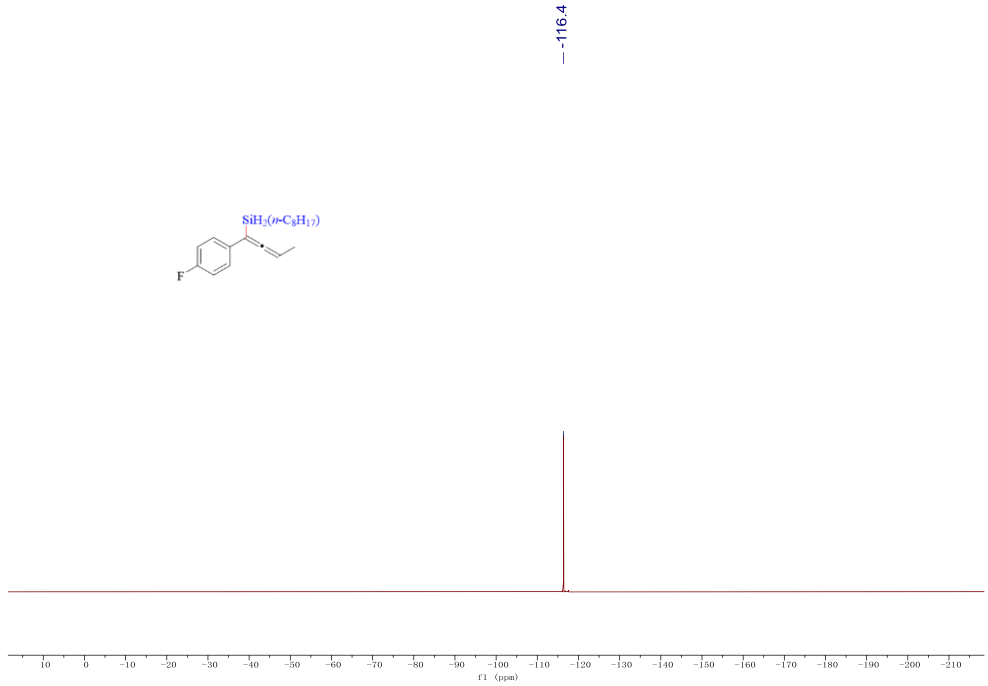
-116.4
SiH2(n-C8H17)
F
f1 (ppm)

(1-(2-Fluorophenyl)buta-1,2-dien-1-yl)(octyl)silane (3hb)

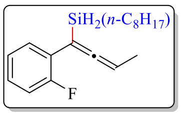

Prepared according to procedure 2 from **1h** (0.2 mmol, 29.2 mg) and **2b** (0.6 mmol, 86.4 mg). The reaction mixture was stirred at 60 °C for 1 h in 0.5 mL dry DCE. The product was isolated in 84% yield (48.9 mg) as colorless oil.

R*f*: 0.80 (petroleum ether).

HRMS (ESI) (m/z): Calcd for $C_{18}H_{28}FSi$ $[M+H]^+$: 291.1944, found: 291.1959.

^{1}H NMR (400 MHz, $CDCl_3$) δ 7.31 (td, *J* = 7.6, 1.5 Hz, 1H), 7.19 – 7.13 (m, 1H), 7.10 – 6.98 (m, 2H), 5.17 (q, *J* = 7.2 Hz, 1H), 4.20 (dt, *J* = 7.7, 3.5 Hz, 2H), 1.75 (d, *J* = 7.2 Hz, 3H), 1.48 – 1.38 (m, 2H), 1.35 – 1.22 (m, 10H), 0.91 – 0.83 (m, 5H).

^{13}C NMR (101 MHz, $CDCl_3$) δ 211.6, 160.2 (d, *J* = 247.1 Hz), 130.1 (d, *J* = 3.9 Hz), 128.1 (d, *J* = 8.1 Hz), 125.3 (d, *J* = 13.7 Hz), 124.3 (d, *J* = 3.4 Hz), 115.8 (d, *J* = 22.3 Hz), 87.9, 81.9, 32.9, 32.0, 29.4, 29.4, 25.0, 22.8, 14.3, 13.2, 10.1.

^{19}F NMR (376 MHz, $CDCl_3$) δ -113.9.

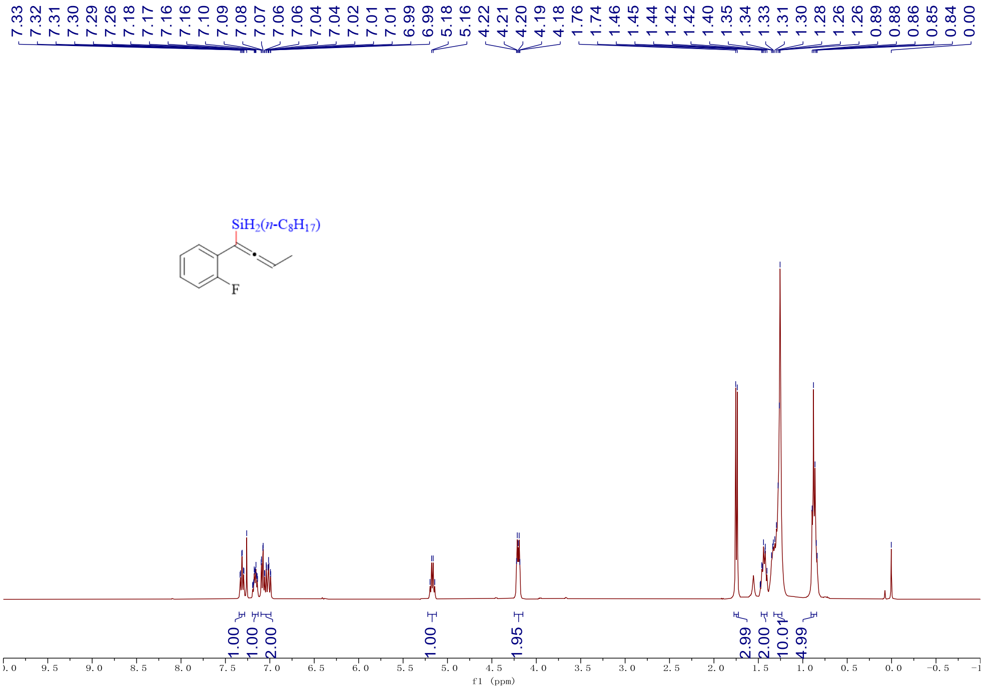

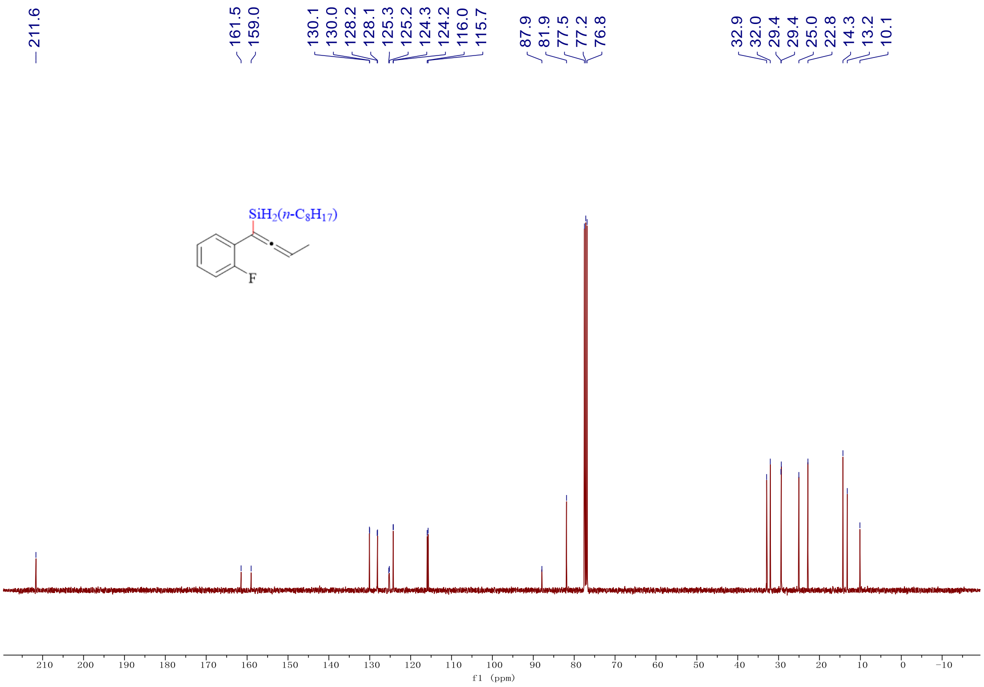

211.6
161.5
159.0
130.1
130.0
128.2
128.1
125.3
125.2
124.3
124.2
116.0
115.7
87.9
81.9
77.5
77.2
76.8
32.9
32.0
29.4
29.4
25.0
22.8
14.3
13.2
10.1
SiH2(n-C8H17)
F
f1 (ppm)

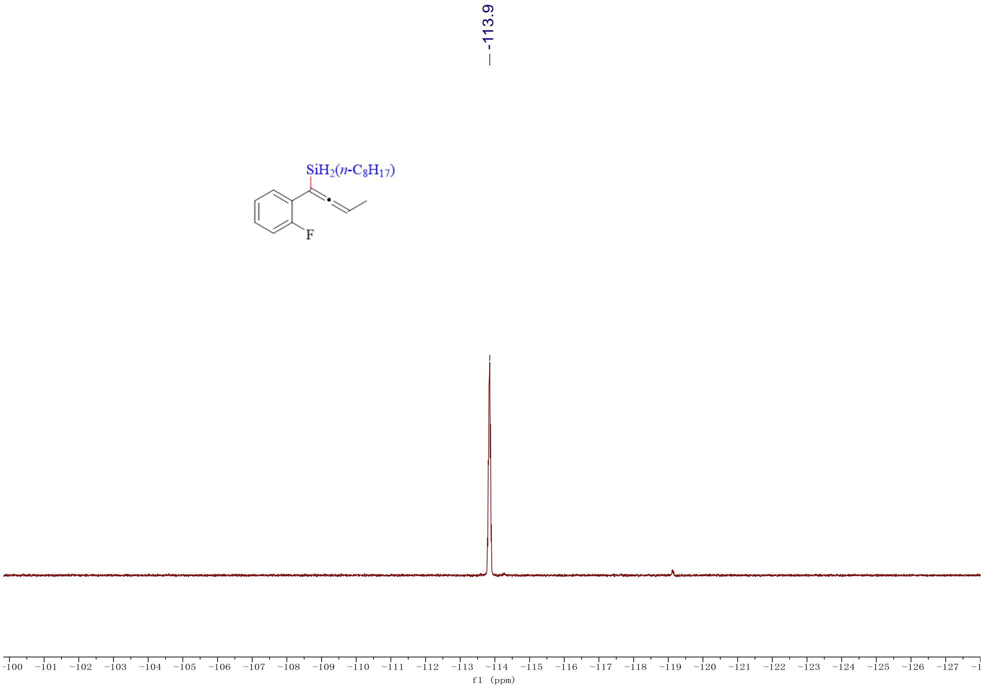

-113.9
SiH2(n-C8H17)
F
f1 (ppm)

(1-(4-Chlorophenyl)buta-1,2-dien-1-yl)(octyl)silane (3ib)

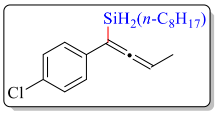

Prepared according to procedure 2 from **1i** (0.2 mmol, 32.5 mg) and **2b** (0.6 mmol, 86.4 mg). The reaction mixture was stirred at 40 °C for 1 h in 1.0 mL dry DCE. The product was isolated in 80% yield (49.1 mg) as colorless oil.

R*f*: 0.88 (petroleum ether).

HRMS (ESI) (m/z): Calcd for $C_{18}H_{28}ClSi$ $[M+H]^+$: 307.1649, found: 307.1656.

^{1}H NMR (400 MHz, $CDCl_3$) δ 7.30 – 7.23 (m, 4H), 5.23 (q, *J* = 7.1 Hz, 1H), 4.27 – 4.19 (m, 2H), 1.75 (d, *J* = 7.1 Hz, 3H), 1.48 – 1.39 (m, 2H), 1.38 – 1.21 (m, 10H), 0.90 – 0.82 (m, 5H).

^{13}C NMR (101 MHz, $CDCl_3$) δ 210.6, 136.0, 132.3, 128.7, 128.5, 92.0, 83.3, 32.9, 32.0, 29.4, 29.3, 25.1, 22.8, 14.3, 13.3, 9.5.

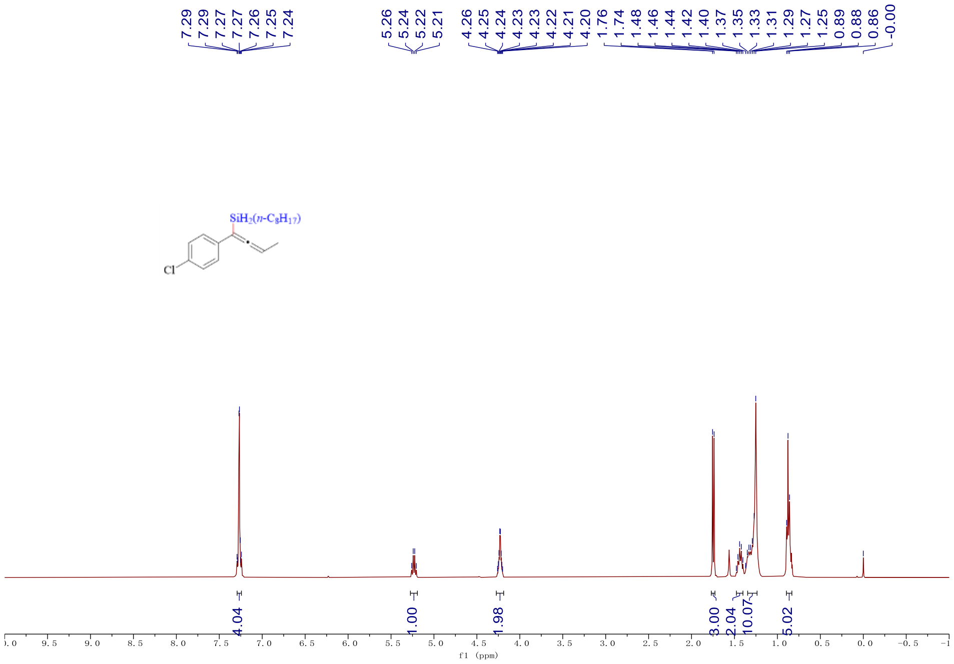

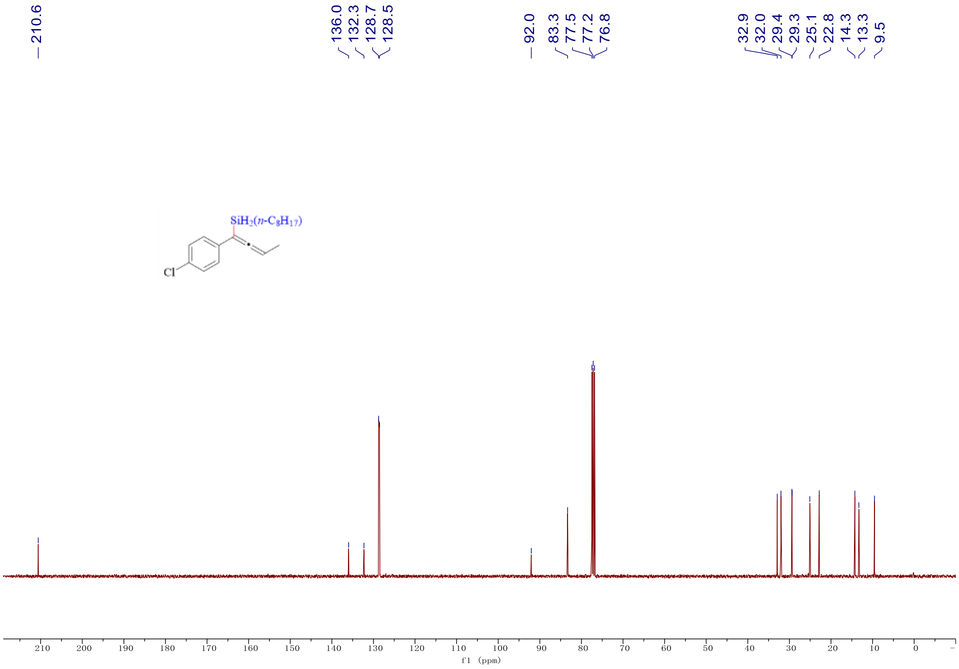

(1-(2-Chlorophenyl)buta-1,2-dien-1-yl)(octyl)silane (3jb)

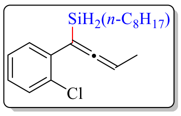

Prepared according to procedure 2 from **1j** (0.2 mmol, 32.5 mg) and **2b** (0.6 mmol, 86.4 mg). The reaction mixture was stirred at 60 °C for 1 h in 0.5 mL dry DCE. The product was isolated in 84% yield (51.8 mg) as colorless oil.

R*f*: 0.74 (petroleum ether).

HRMS (ESI) (m/z): Calcd for $C_{18}H_{28}ClSi$ $[M+H]^+$: 307.1649, found: 307.1644

^{1}H NMR (400 MHz, $CDCl_3$) δ 7.35 (dd, *J* = 7.8, 1.4 Hz, 1H), 7.24 – 7.17 (m, 2H), 7.16 – 7.10 (m, 1H), 5.07 (q, *J* = 7.1 Hz, 1H), 4.21 – 4.14 (m, 2H), 1.73 (d, *J* = 7.1 Hz, 3H), 1.47 – 1.38 (m, 2H), 1.36 – 1.21 (m, 10H), 0.91 – 0.79 (m, 5H).

^{13}C NMR (101 MHz, $CDCl_3$) δ 210.6, 137.0, 132.9, 130.3, 129.9, 127.7, 126.9, 91.4, 81.2, 32.9, 32.0, 29.4, 29.3, 25.0, 22.8, 14.3, 13.0, 10.2.

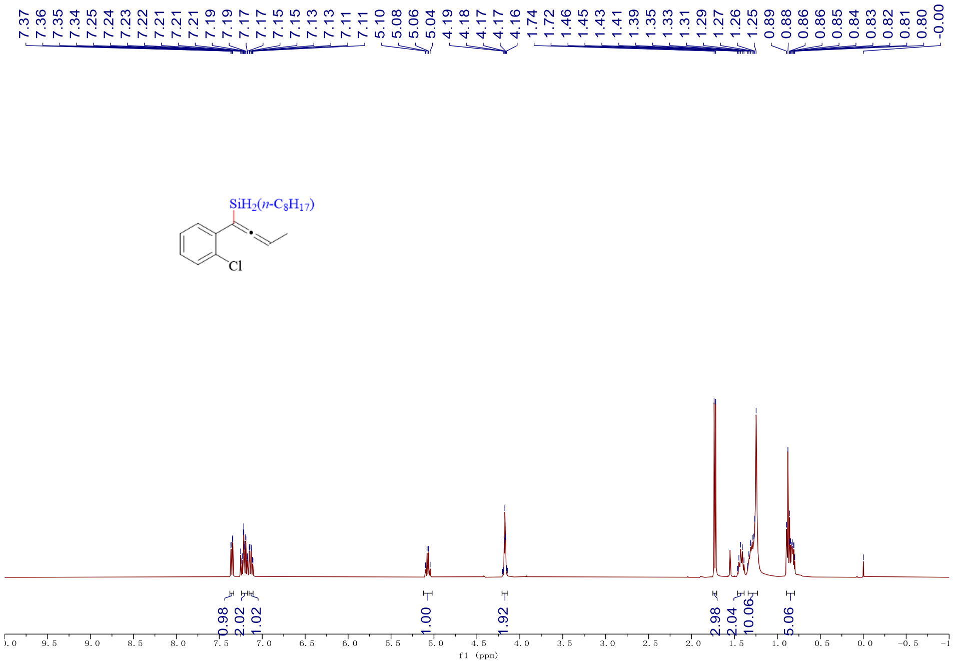

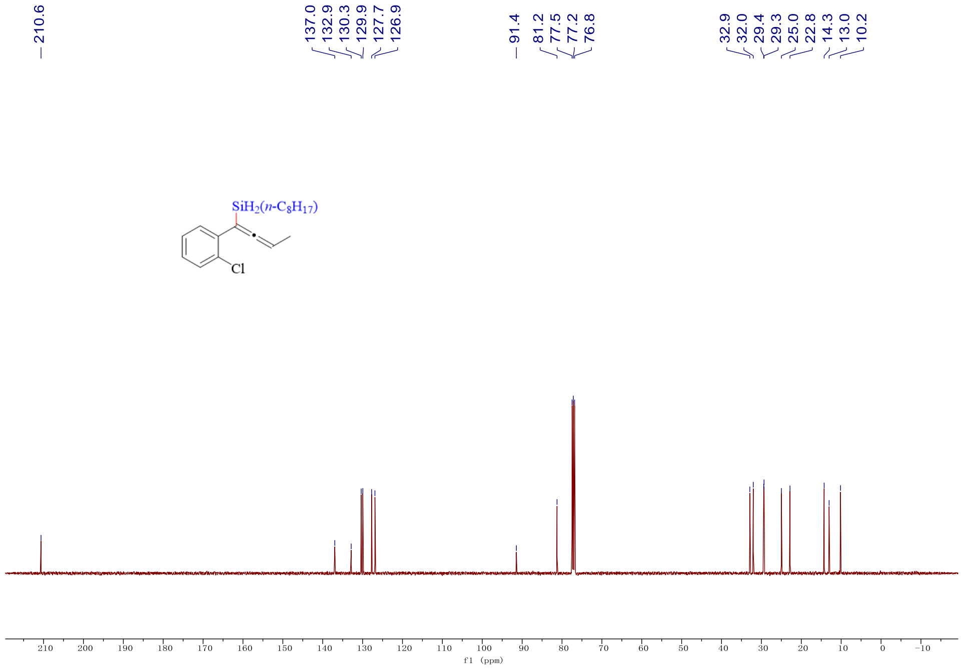

(1-(4-Bromophenyl)buta-1,2-dien-1-yl)(octyl)silane (3kb)

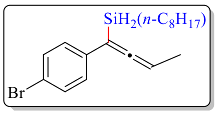

Prepared according to procedure 2 from **1k** (0.2 mmol, 41.4 mg) and **2b** (0.6 mmol, 86.4 mg). The reaction mixture was stirred at 60 °C for 1 h in 0.5 mL dry DCE. The product was isolated in 80% yield (56.2 mg) as colorless oil.

R*f*: 0.88 (petroleum ether).

HRMS (EI) (m/z): Calcd for $C_{18}H_{27}BrSi$ $[M]^+$: 350.1065, found: 350.1065.

^{1}H NMR (400 MHz, $CDCl_3$) δ 7.43 – 7.38 (m, 2H), 7.24 – 7.19 (m, 2H), 5.22 (q, *J* = 7.0 Hz, 1H), 4.27 – 4.18 (m, 2H), 1.75 (d, *J* = 7.1 Hz, 3H), 1.49 – 1.38 (m, 2H), 1.37 – 1.21 (m, 10H), 0.92 – 0.81 (m, 5H).

^{13}C NMR (101 MHz, $CDCl_3$) δ 210.6, 136.5, 131.7, 128.9, 120.4, 92.1, 83.4, 32.9, 32.0, 29.4, 29.3, 25.1, 22.8, 14.3, 13.3, 9.5.

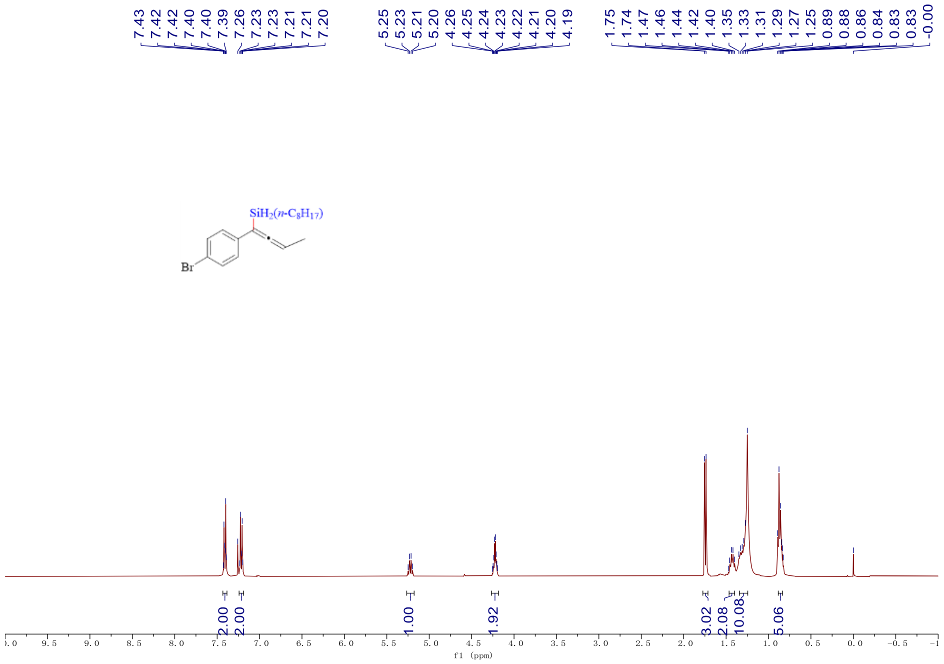

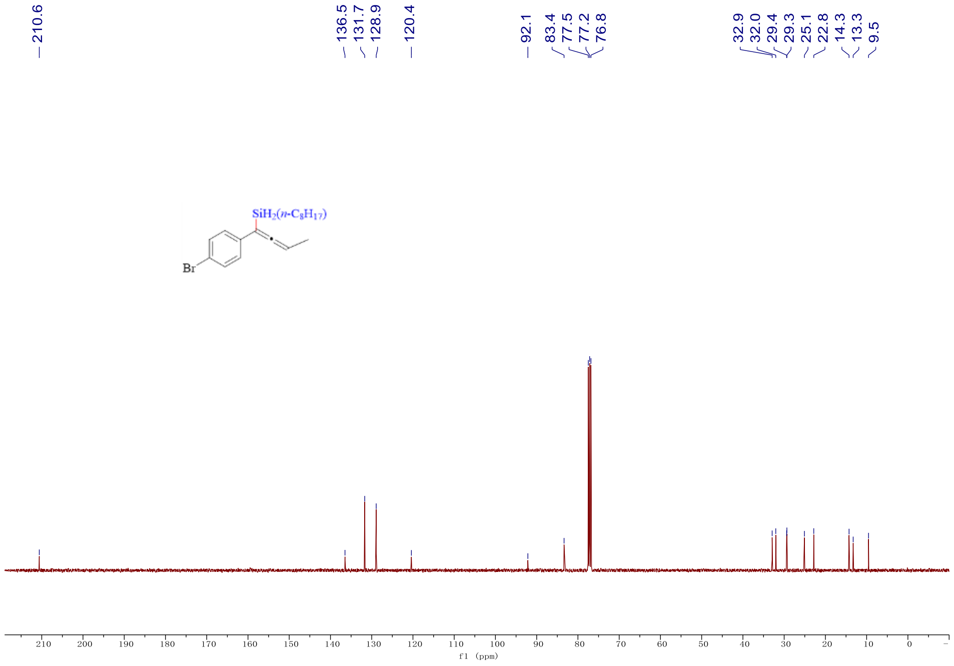

(1-(4-Methoxyphenyl)buta-1,2-dien-1-yl)(octyl)silane (3lb)

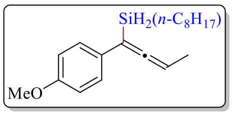

Prepared according to procedure 2 from **1l** (0.2 mmol, 31.6 mg) and **2b** (0.6 mmol, 86.4 mg). The reaction mixture was stirred at 60 °C for 1 h in 0.5 mL dry DCE. The product was isolated in 72% yield (43.6 mg) as colorless oil.

R*f*: 0.30 (petroleum ether).

HRMS (ESI) (m/z): Calcd for $C_{19}H_{31}OSi$ $[M+H]^+$: 303.2144, found: 303.2146.

^{1}H NMR (400 MHz, $CDCl_3$) δ 7.29 (d, *J* = 8.7 Hz, 2H), 6.85 (d, *J* = 8.8 Hz, 2H), 5.20 (q, *J* = 7.1 Hz, 1H), 4.27 – 4.20 (m, 2H), 3.79 (s, 3H), 1.74 (d, *J* = 7.1 Hz, 3H), 1.49 – 1.40 (m, 2H), 1.37 – 1.22 (m, 10H), 0.90 – 0.85 (m, 5H).

^{13}C NMR (101 MHz, $CDCl_3$) δ 209.8, 158.5, 129.5, 128.3, 114.1, 92.1, 82.9, 55.4, 33.0, 32.0, 29.4, 29.4, 25.2, 22.8, 14.3, 13.6, 9.7.

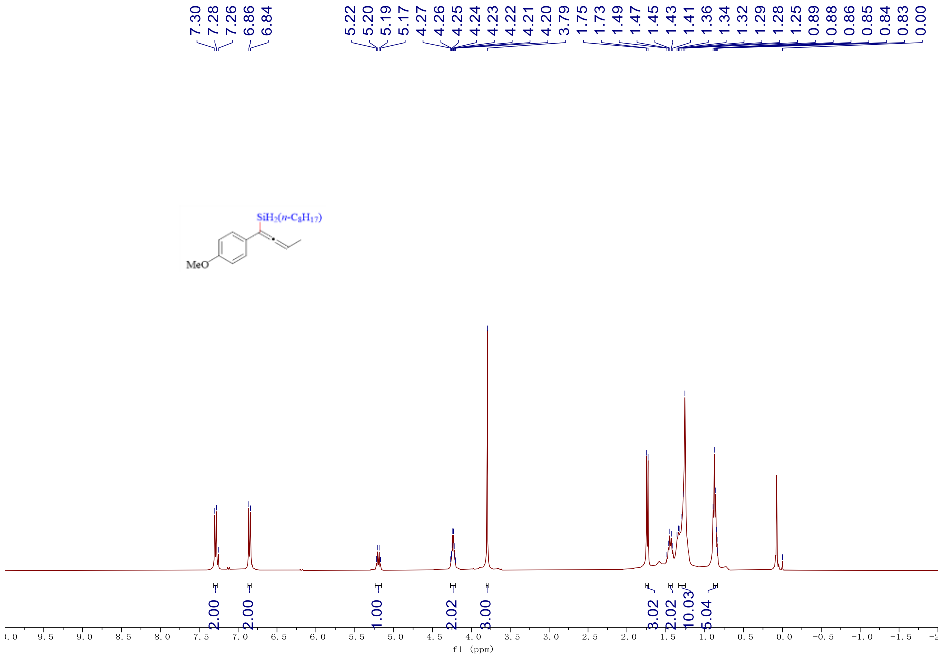

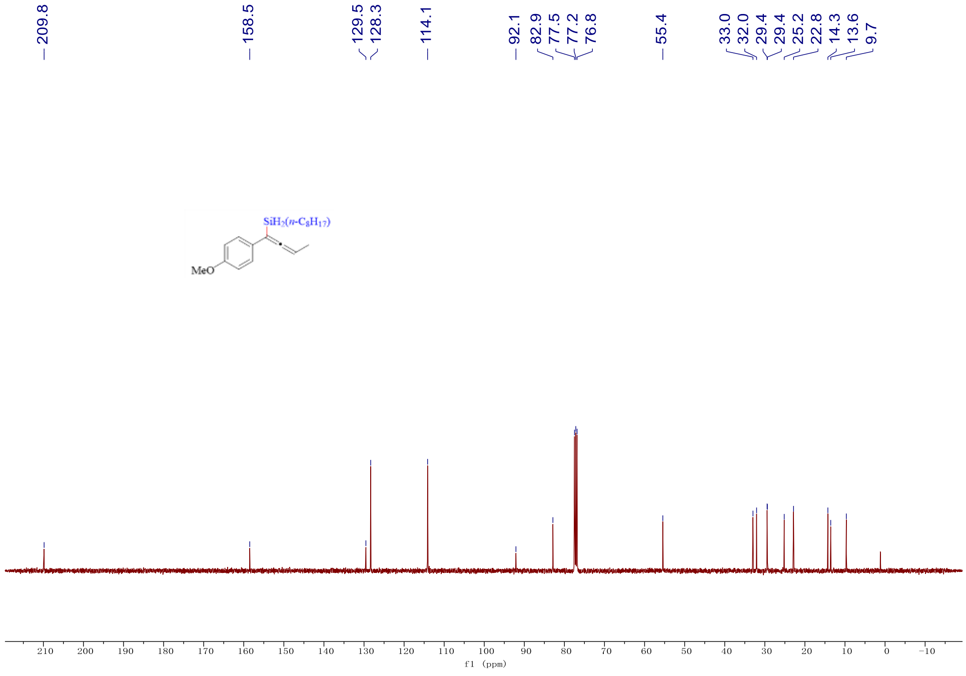

(1-(Benzo[*d*][1,3]dioxol-5-yl)buta-1,2-dien-1-yl)(octyl)silane (3mb)

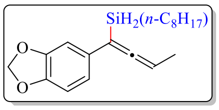

Prepared according to procedure 2 from **1m** (0.2 mmol, 34.4 mg) and **2b** (0.6 mmol, 86.4 mg). The reaction mixture was stirred at 60 °C for 1 h in 0.5 mL dry DCE. The product was isolated in 72% yield (45.6 mg) as colorless oil.

R*f*: 0.30 (ethyl acetate : petroleum ether = 1:100).

HRMS (ESI) (m/z): Calcd for $C_{19}H_{29}O_2Si$ $[M+H]^+$: 317.1937, found: 317.1936.

^{1}H NMR (400 MHz, $CDCl_3$) δ 6.90 (d, *J* = 1.7 Hz, 1H), 6.80 (dd, *J* = 8.0, 1.7 Hz, 1H), 6.75 (d, *J* = 8.0 Hz, 1H), 5.93 (s, 2H), 5.21 (q, *J* = 7.2 Hz, 1H), 4.27 – 4.17 (m, 2H), 1.73 (d, *J* = 7.1 Hz, 3H), 1.50 – 1.39 (m, 2H), 1.38 – 1.22 (m, 10H), 0.92 – 0.82 (m, 5H).

^{13}C NMR (101 MHz, $CDCl_3$) δ 209.9, 148.1, 146.5, 131.4, 120.6, 108.3, 107.7, 101.1, 92.6, 83.2, 32.9, 32.0, 29.4, 29.4, 25.1, 22.8, 14.3, 13.5, 9.6.

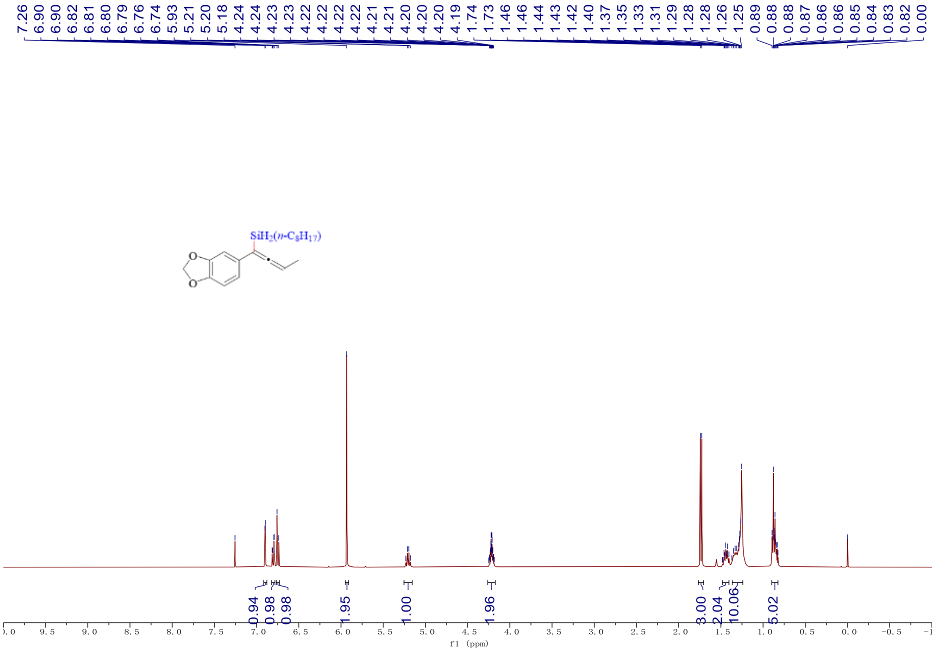

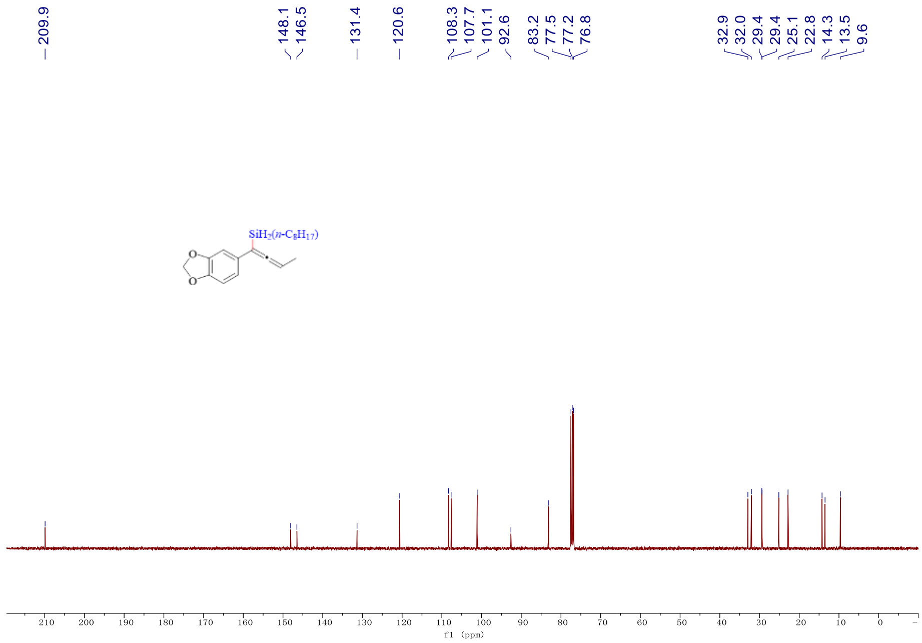

Octyl(1-(4-(trifluoromethoxy)phenyl)buta-1,2-dien-1-yl)silane (3nb)

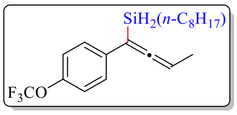

Prepared according to procedure 2 from **1n** (0.2 mmol, 42.4 mg) and **2b** (0.6 mmol, 86.4 mg). The reaction mixture was stirred at 40 °C for 1 h in 1.0 mL dry DCE. The product was isolated in 74% yield (53.0 mg) as colorless oil.

R*f*: 0.81 (petroleum ether).

HRMS (EI) (m/z): Calcd for $C_{19}H_{27}F_3OSi$ $[M]^+$: 356.1783, found: 356.1770.

^{1}H NMR (400 MHz, $CDCl_3$) δ 7.39 – 7.34 (m, 2H), 7.18 – 7.12 (m, 2H), 5.25 (q, *J* = 7.1 Hz, 1H), 4.29 – 4.20 (m, 2H), 1.75 (d, *J* = 7.2 Hz, 3H), 1.50 – 1.40 (m, 2H), 1.38 – 1.23 (m, 10H), 0.91 – 0.83 (m, 5H).

^{13}C NMR (101 MHz, $CDCl_3$) δ 210.8, 147.9, 136.3, 128.5, 121.2, 120.6 (q, *J* = 256.8 Hz), 91.9, 83.3, 32.9, 32.0, 29.4, 29.3, 25.1, 22.8, 14.2, 13.3, 9.6.

^{19}F NMR (376 MHz, $CDCl_3$) δ -57.9.

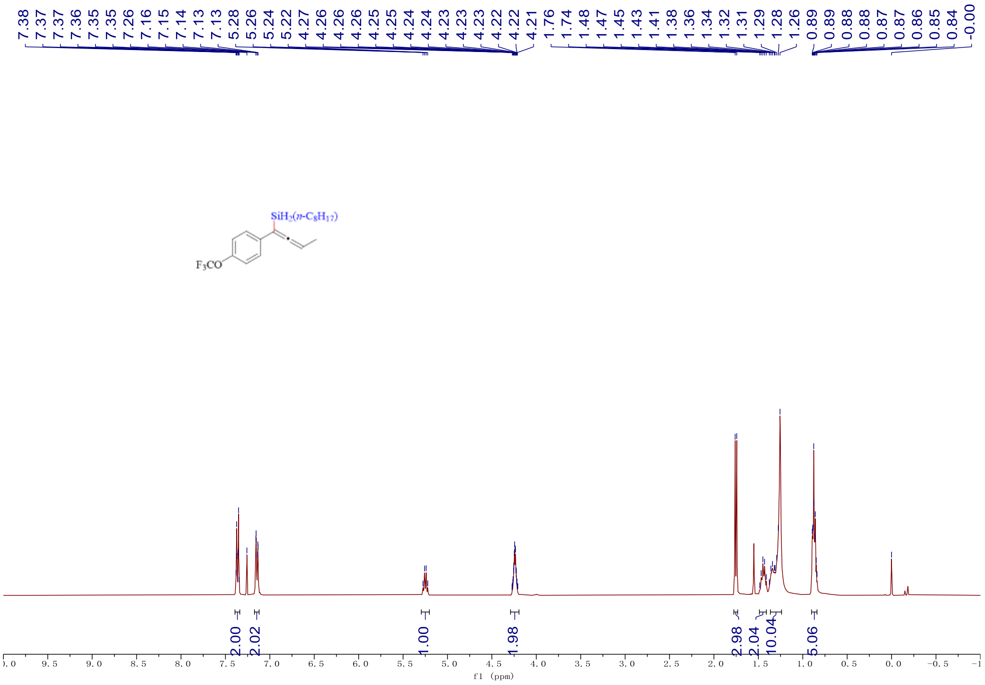

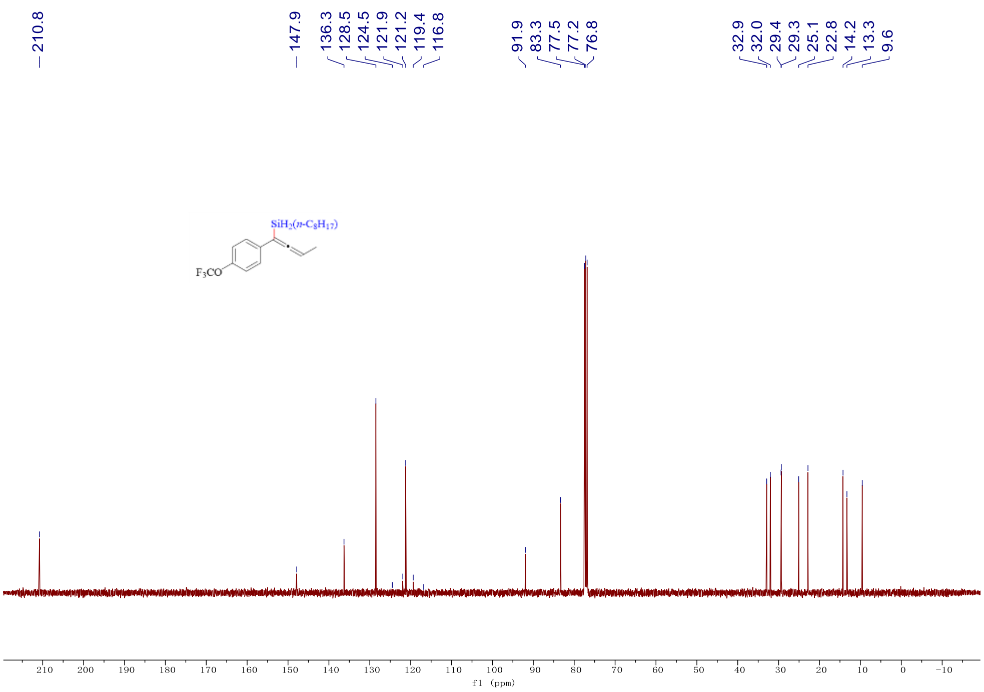
210.8
147.9
136.3
128.5
124.5
121.9
121.2
119.4
116.8
91.9
83.3
77.5
77.2
76.8
32.9
32.0
29.4
29.3
25.1
22.8
14.2
13.3
9.6
SiH2(n-C8H17)
F3CO
f1 (ppm)

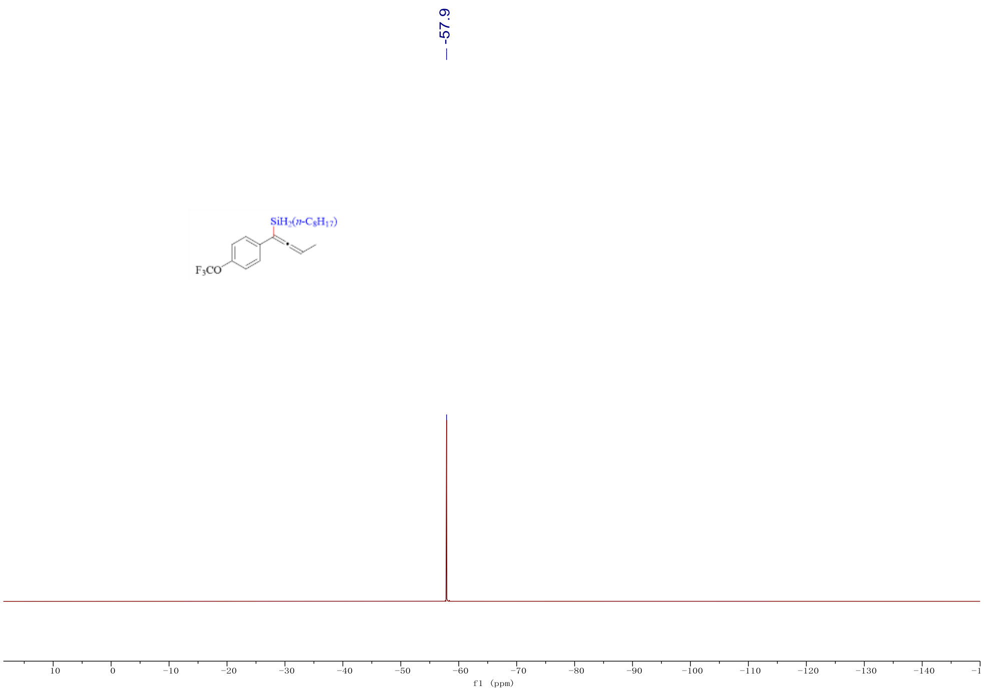
-57.9
SiH2(n-C8H17)
F3CO
f1 (ppm)

3-(1-(Octylsilyl)buta-1,2-dien-1-yl)phenyl 4-methylbenzenesulfonate (3ob)

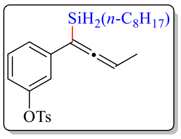

Prepared according to procedure 2 from **1o** (0.2 mmol, 59.7 mg) and **2b** (0.6 mmol, 86.4 mg). The reaction mixture was stirred at 60 °C for 1 h in 0.5 mL dry DCE. The product was isolated in 80% yield (70.8 mg) as colorless oil.

R$_f$: 0.44 (ethyl acetate : petroleum ether = 1:20).

HRMS (ESI) (m/z): Calcd for $C_{25}H_{35}O_3SSi$ $[M+H]^+$: 443.2076, found: 443.2083.

^{1}H NMR (400 MHz, $CDCl_3$) δ 7.74 – 7.69 (m, 2H), 7.34 – 7.29 (m, 2H), 7.23 – 7.19 (m, 2H), 6.88 – 6.81 (m, 2H), 5.19 (q, J = 7.1 Hz, 1H), 4.15 – 4.08 (m, 2H), 2.44 (s, 3H), 1.70 (d, J = 7.2 Hz, 3H), 1.45 – 1.36 (m, 2H), 1.35 – 1.23 (m, 10H), 0.88 (t, J = 6.7 Hz, 3H), 0.83 – 0.77 (m, 2H).

^{13}C NMR (101 MHz, $CDCl_3$) δ 210.8, 150.1, 145.3, 139.5, 132.6, 129.8, 129.6, 128.7, 125.9, 121.0, 120.4, 92.1, 83.5, 32.9, 32.0, 29.4, 29.3, 25.0, 22.8, 21.8, 14.3, 13.1, 9.4.

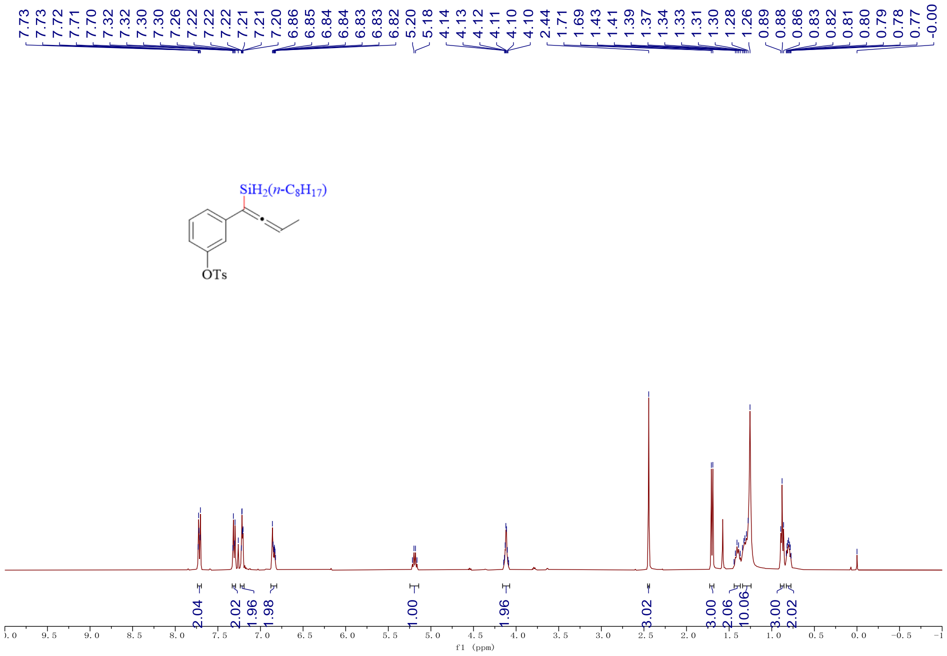

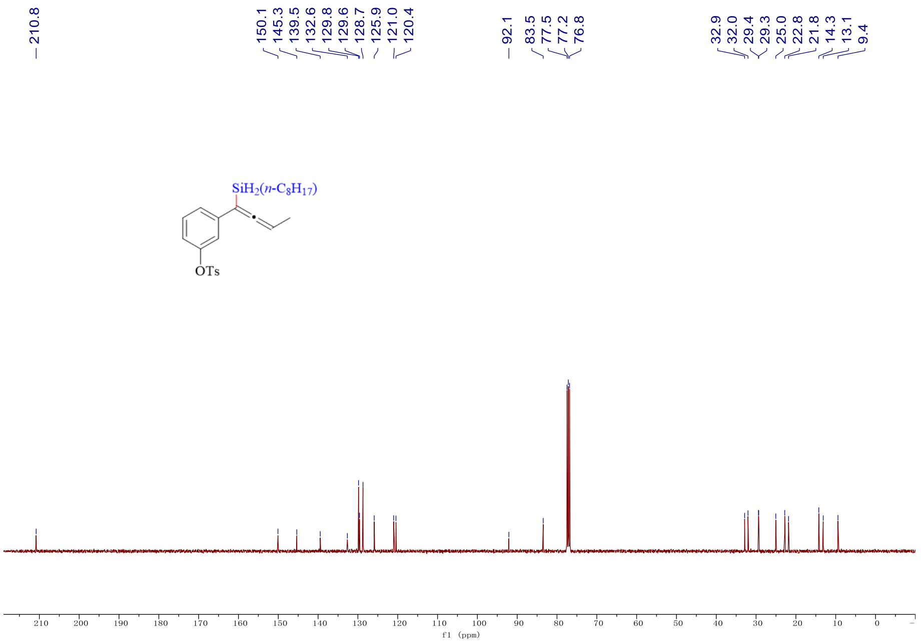

N-methyl-*N*-(4-(1-(octylsilyl)buta-1,2-dien-1-yl)phenyl)benzamide (3pb)

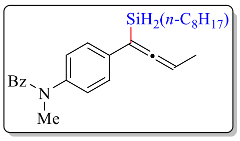

Prepared according to procedure 2 from **1p** (0.2 mmol, 52.3 mg) and **2b** (0.6 mmol, 86.4 mg). The reaction mixture was stirred at 60 °C for 1 h in 1.0 mL dry DCE. The product was isolated in 82% yield (66.5 mg) as yellow oil.

R$_f$: 0.50 (ethyl acetate : petroleum ether = 1:5).

HRMS (ESI) (m/z): Calcd for $C_{26}H_{36}NOSi$ $[M+H]^+$: 406.2566, found: 406.2567.

^{1}H NMR (400 MHz, $CDCl_3$) δ 7.32 (d, *J* = 7.4 Hz, 2H), 7.25 – 7.15 (m, 5H), 6.96 (d, *J* = 8.1 Hz, 2H), 5.21 (q, *J* = 7.1 Hz, 1H), 4.25 – 4.14 (m, 2H), 3.48 (s, 3H), 1.72 (d, *J* = 7.1 Hz, 3H), 1.46 – 1.37 (m, 2H), 1.36 – 1.21 (m, 10H), 0.92 – 0.78 (m, 5H).

^{13}C NMR (101 MHz, $CDCl_3$) δ 210.6, 170.8, 143.3, 136.0, 135.6, 129.8, 128.9, 127.9, 127.0, 92.1, 83.3, 38.6, 32.9, 32.0, 29.4, 29.3, 25.1, 22.8, 14.3, 13.3, 9.5.

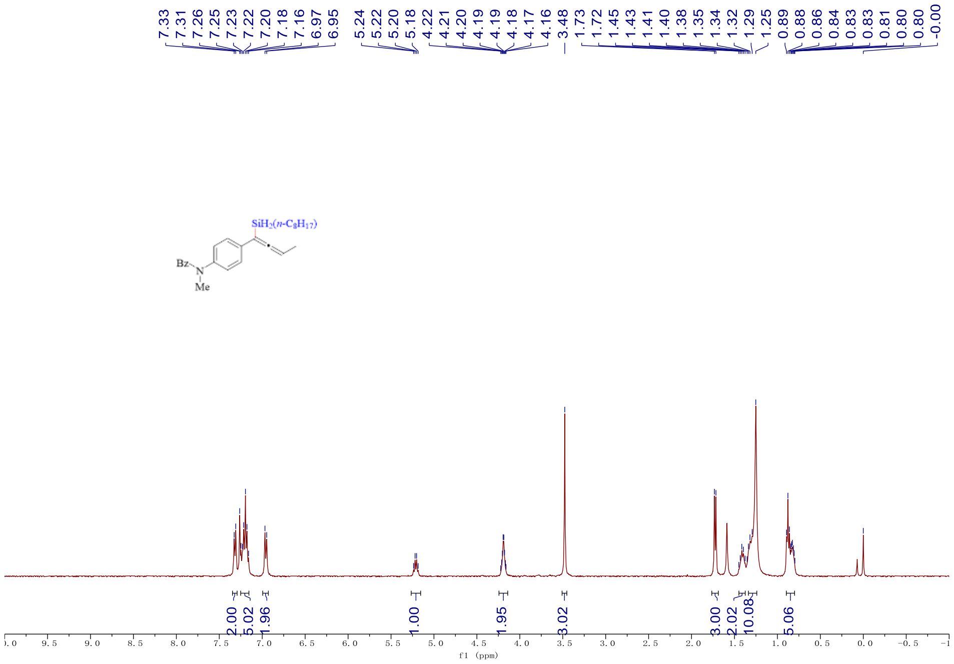

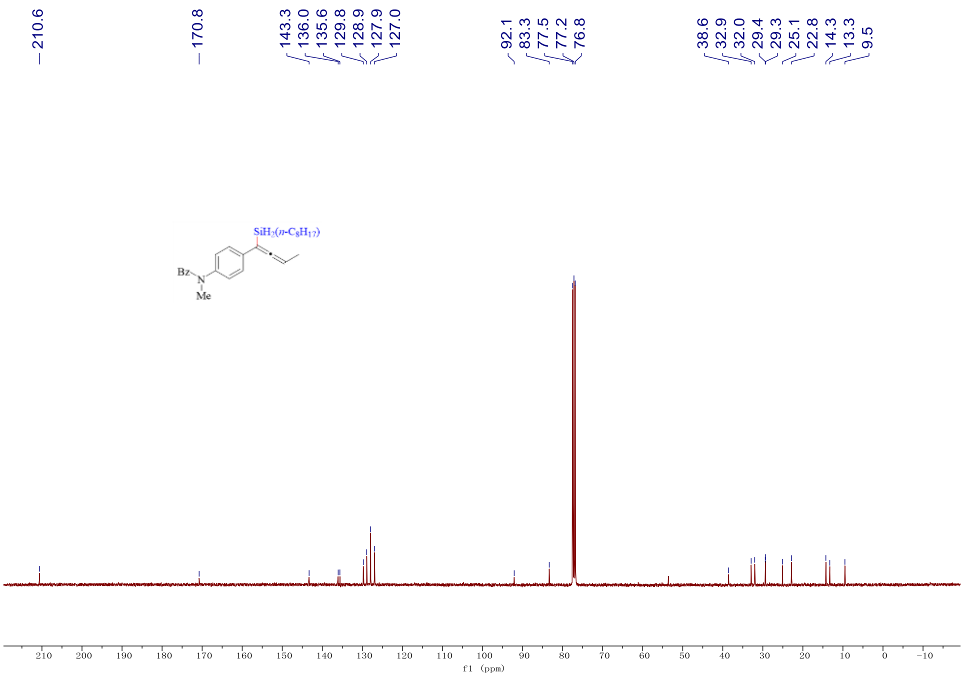

4-(1-(Octylsilyl)buta-1,2-dien-1-yl)-N,N-diphenylaniline (3qb)

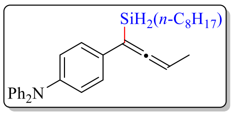

Prepared according to procedure 2 from **1q** (0.2 mmol, 59.1 mg) and **2b** (0.6 mmol, 86.4 mg). The reaction mixture was stirred at 60 °C for 1 h in 0.5 mL dry DCE. The product was isolated in 69% yield (60.7 mg) as yellow oil.

R*f*: 0.48 (ethyl acetate : petroleum ether = 1:100).

HRMS (ESI) (m/z): Calcd for $C_{30}H_{38}NSi$ $[M+H]^+$: 440.2774, found: 440.2752.

^{1}H NMR (400 MHz, $CDCl_3$) δ 7.25 – 7.20 (m, 6H), 7.10 – 7.06 (m, 4H), 7.03 – 6.97 (m, 4H), 5.21 (q, *J* = 7.1 Hz, 1H), 4.27 – 4.20 (m, 2H), 1.74 (d, *J* = 7.1 Hz, 3H), 1.50 – 1.41 (m, 2H), 1.39 – 1.22 (m, 10H), 0.91 – 0.84 (m, 5H).

^{13}C NMR (101 MHz, $CDCl_3$) δ 210.1, 147.9, 146.4, 131.4, 129.3, 128.1, 124.3, 124.3, 122.8, 92.3, 83.1, 33.0, 32.0, 29.4, 29.4, 25.2, 22.8, 14.3, 13.5, 9.7.

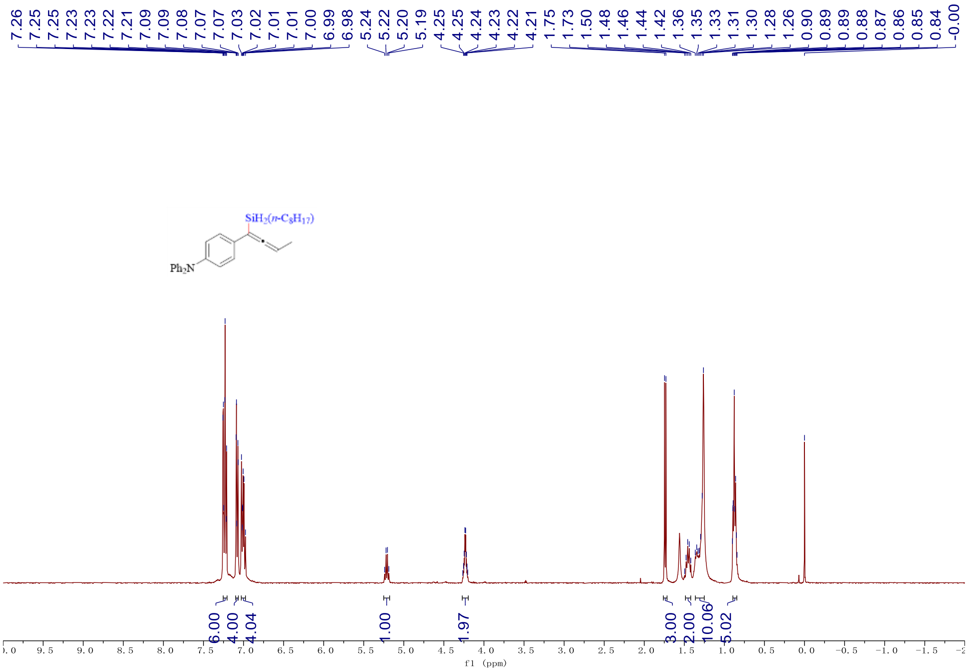

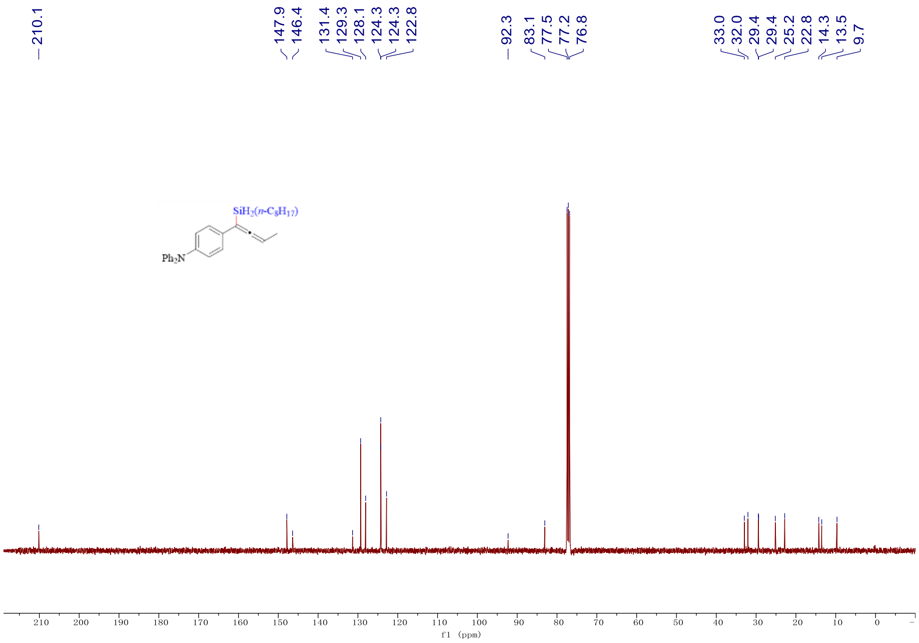

Octyl(1-(4-(trifluoromethyl)phenyl)buta-1,2-dien-1-yl)silane (3rb)

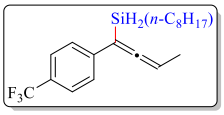

Prepared according to procedure 2 from **1r** (0.2 mmol, 39.2 mg) and **2b** (0.6 mmol, 86.4 mg). The reaction mixture was stirred at 40 °C for 1 h in 1.0 mL dry DCE. The product was isolated in 81% yield (55.3 mg) as colorless oil.

R*f*: 0.72 (petroleum ether).

HRMS (EI) (m/z): Calcd for $C_{19}H_{27}F_3Si$ $[M]^+$: 340.1834, found: 340.1836.

^{1}H NMR (400 MHz, $CDCl_3$) δ 7.54 (d, *J* = 8.0 Hz, 2H), 7.45 (d, *J* = 8.0 Hz, 2H), 5.33 – 5.24 (q, *J* = 7.2 Hz, 1H), 4.26 (m, 2H), 1.77 (d, *J* = 7.2 Hz, 3H), 1.50 – 1.40 (m, 2H), 1.38 – 1.23 (m, 10H), 0.91 – 0.84 (m, 5H).

^{13}C NMR (101 MHz, $CDCl_3$) δ 211.4, 141.5, 128.5 (q, *J* = 32.3 Hz), 127.5, 125.5 (q, *J* = 3.8 Hz), 124.4 (q, *J* = 271.6 Hz), 92.4, 83.5, 32.9, 32.0, 29.4, 29.3, 25.1, 22.8, 14.2, 13.2, 9.5.

^{19}F NMR (376 MHz, $CDCl_3$) δ -62.4.

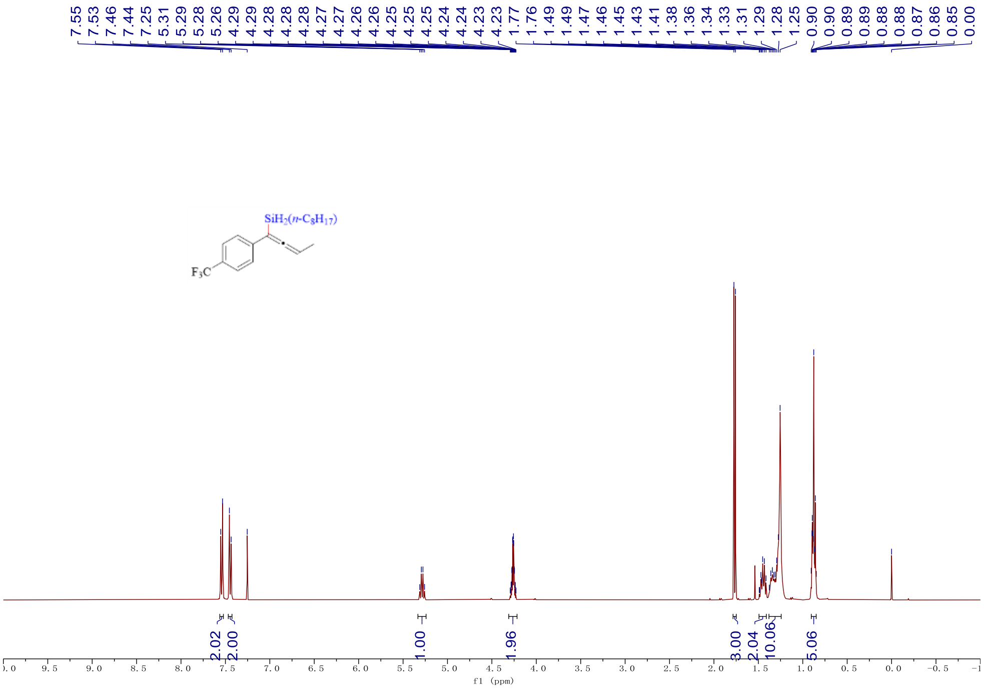

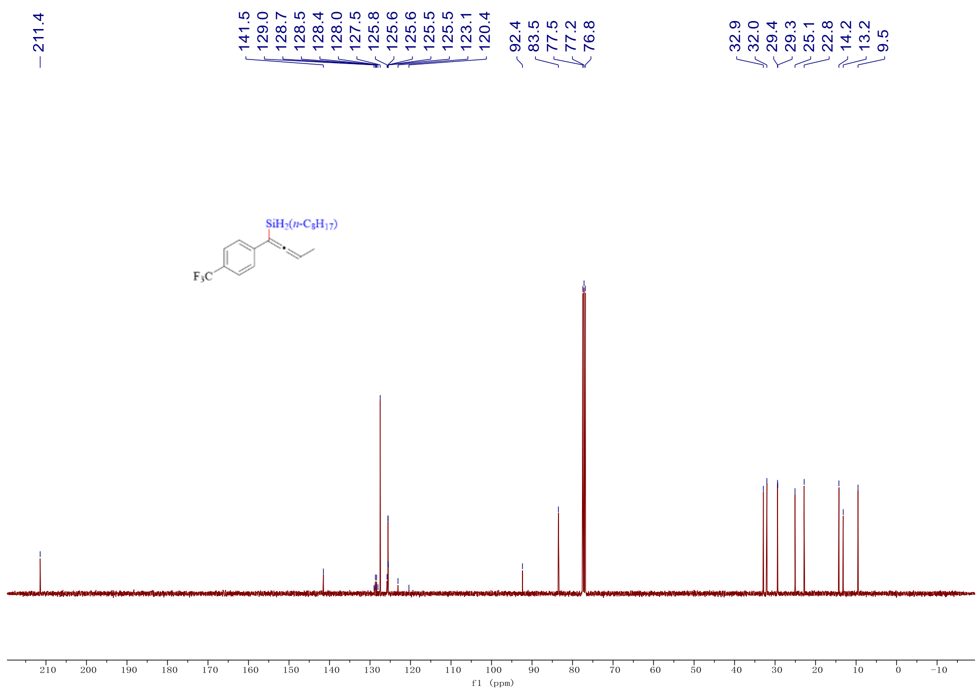
211.4
141.5
129.0
128.7
128.5
128.4
128.0
127.5
125.8
125.6
125.6
125.5
125.5
123.1
120.4
92.4
83.5
77.5
77.2
76.8
32.9
32.0
29.4
29.3
25.1
22.8
14.2
13.2
9.5
SiH2(n-C8H17)
F3C
f1 (ppm)

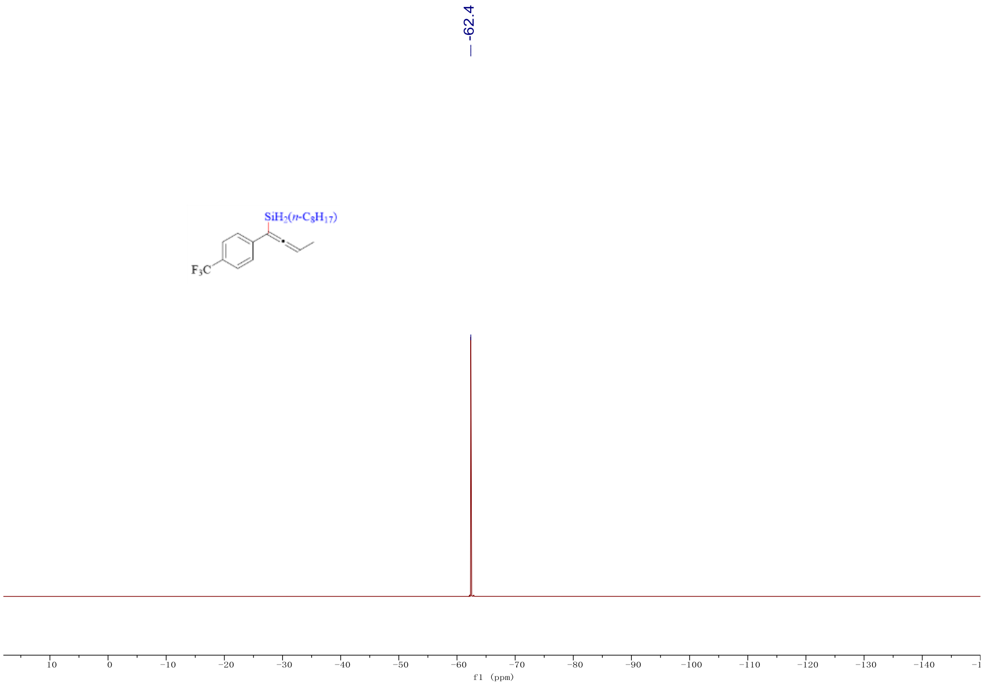
-62.4
SiH2(n-C8H17)
F3C
f1 (ppm)

Methyl 4-(1-(octylsilyl)buta-1,2-dien-1-yl)benzoate (3sb)

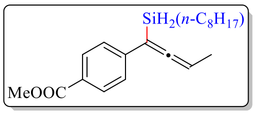

Prepared according to procedure 2 from **1s** (0.2 mmol, 37.2 mg) and **2b** (0.6 mmol, 86.4 mg). The reaction mixture was stirred at 40 °C for 1 h in 0.5 mL dry DCE. The product was isolated in 73% yield (48.3 mg) as colorless oil.

R*f*: 0.50 (ethyl acetate : petroleum ether = 1:50).

HRMS (ESI) (m/z): Calcd for $C_{20}H_{31}O_2Si$ $[M+H]^+$: 331.2093, found: 331.2091.

^{1}H NMR (400 MHz, $CDCl_3$) δ 7.96 (dt, *J* = 8.6, 2.0 Hz, 2H), 7.41 (dt, *J* = 8.3, 1.8 Hz, 2H), 5.28 (q, *J* = 7.2 Hz, 1H), 4.31 – 4.23 (m, 2H), 3.91 (s, 3H), 1.77 (d, *J* = 7.1 Hz, 3H), 1.49 – 1.40 (m, 2H), 1.38 – 1.23 (m, 10H), 0.91 – 0.84 (m, 5H).

^{13}C NMR (101 MHz, $CDCl_3$) δ 211.5, 167.1, 142.6, 130.0, 128.1, 127.2, 92.7, 83.4, 52.2, 32.9, 32.0, 29.4, 29.3, 25.1, 22.8, 14.2, 13.2, 9.5.

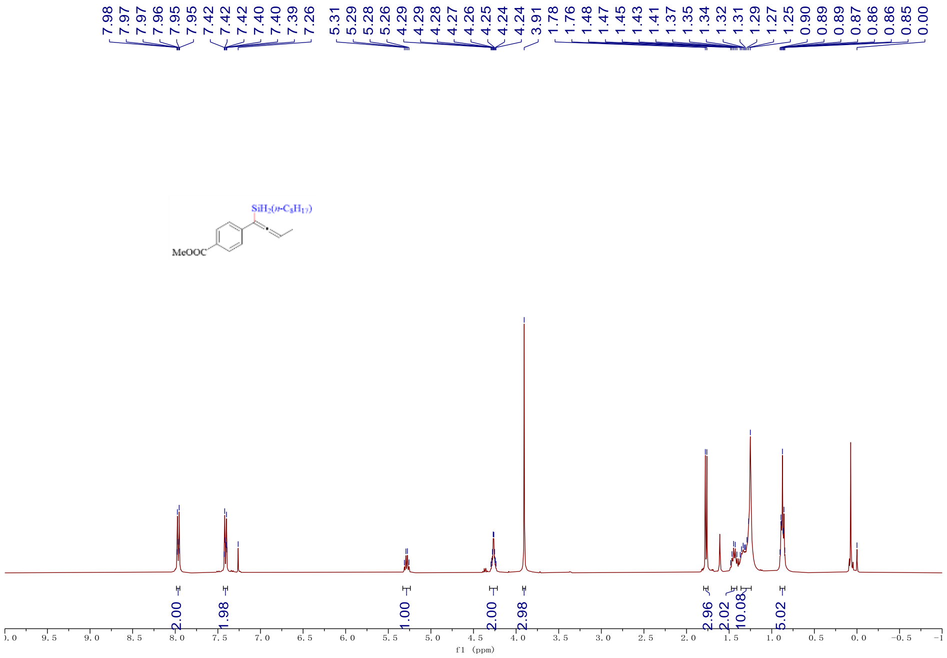

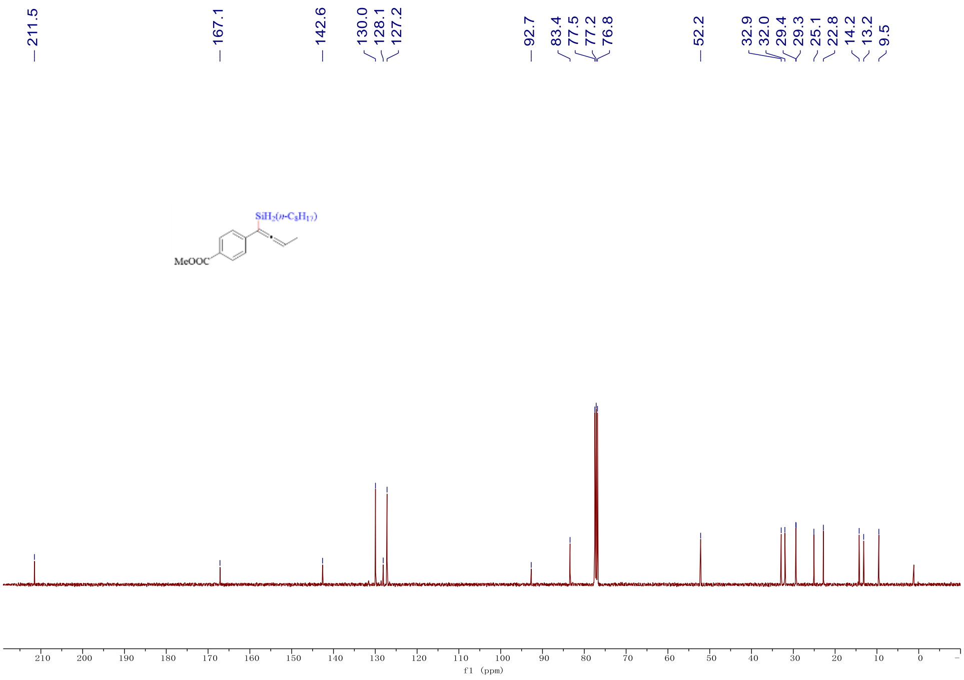

Octyl(1-(thiophen-3-yl)buta-1,2-dien-1-yl)silane (3tb)

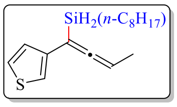

Prepared according to procedure 2 from **1t** (0.2 mmol, 26.8 mg) and **2b** (0.6 mmol, 86.4 mg). The reaction mixture was stirred at 60 °C for 1 h in 0.5 mL dry DCE. The product was isolated in 80% yield (44.7 mg) as colorless oil.

R*f*: 0.70 (petroleum ether).

HRMS (ESI) (m/z): Calcd for $C_{16}H_{26}SSiNa$ $[M+Na]^+$: 301.1422, found: 301.1416.

^{1}H NMR (400 MHz, $CDCl_3$) δ 7.27 – 7.25 (m, 1H), 7.12 (dd, *J* = 5.0, 1.3 Hz, 1H), 7.09 (d, *J* = 2.8 Hz, 1H), 5.18 (q, *J* = 7.1 Hz, 1H), 4.25 – 4.18 (m, 2H), 1.73 (d, *J* = 7.1 Hz, 3H), 1.50 – 1.41 (m, 2H), 1.38 – 1.20 (m, 10H), 0.92 – 0.83 (m, 5H).

^{13}C NMR (101 MHz, $CDCl_3$) δ 210.3, 138.2, 127.3, 125.5, 120.3, 87.8, 82.6, 32.9, 32.0, 29.4, 29.4, 25.1, 22.8, 14.3, 13.5, 9.5.

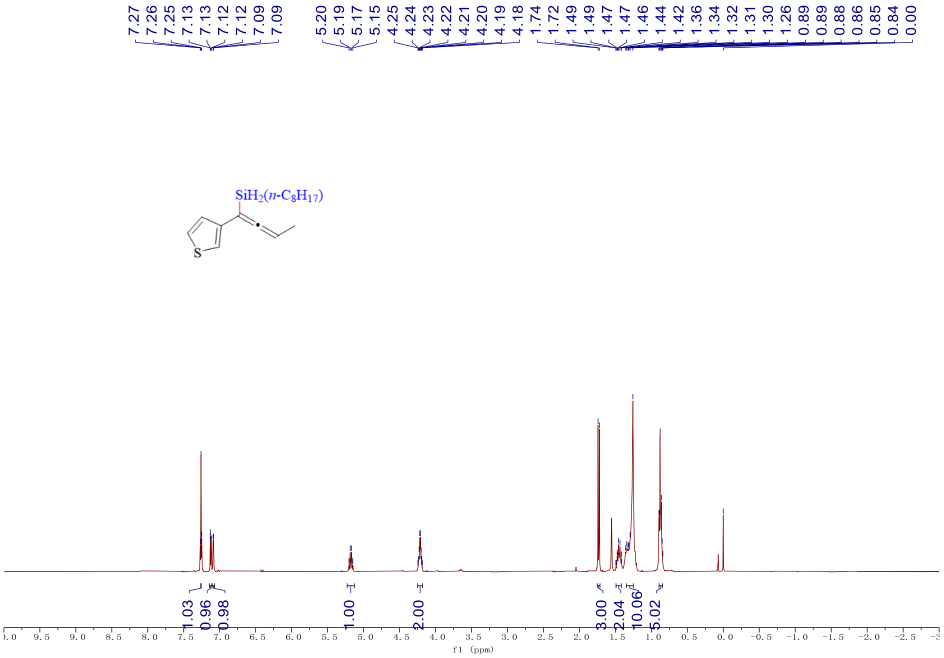

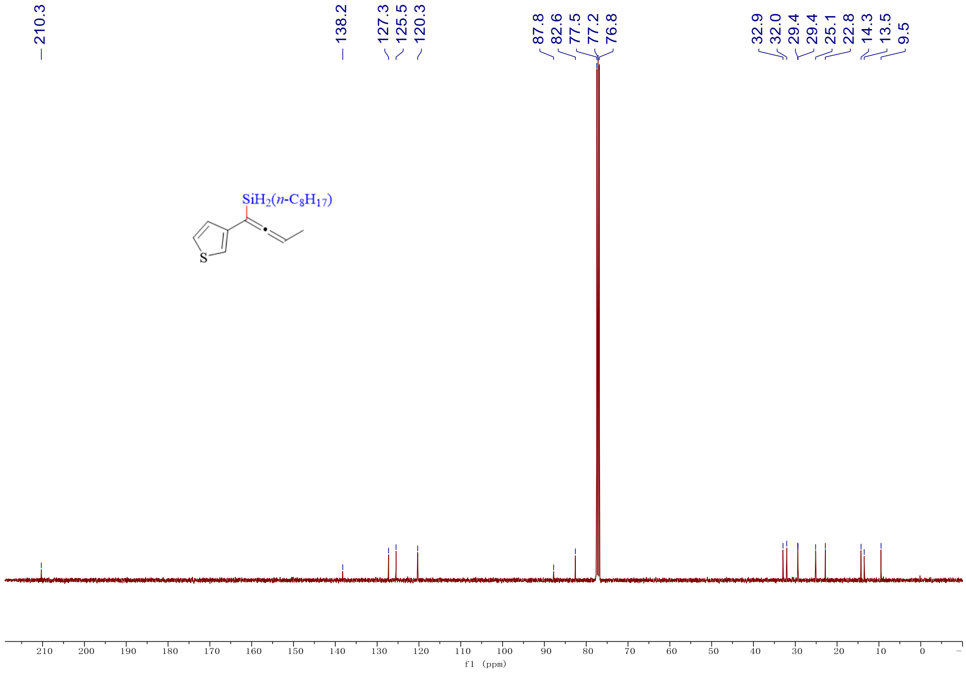

3-(1-(Octylsilyl)buta-1,2-dien-1-yl)-1-tosyl-1*H*-indole (3ub)

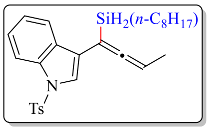

Prepared according to procedure 2 from **1u** (0.2 mmol, 64.3 mg) and **2b** (0.6 mmol, 86.4 mg). The reaction mixture was stirred at 60 °C for 1 h in 0.5 mL dry DCE. The product was isolated in 79% yield (73.7 mg) as colorless oil.

R*f*: 0.35 (ethyl acetate : petroleum ether = 1:20).

HRMS (ESI) (m/z): Calcd for $C_{27}H_{36}NO_2SSi$ $[M+H]^+$: 466.2236, found: 466.2239.

^{1}H NMR (400 MHz, $CDCl_3$) δ 7.97 (d, *J* = 8.2 Hz, 1H), 7.92 (d, *J* = 7.7 Hz, 1H), 7.75 (d, *J* = 8.4 Hz, 2H), 7.48 (s, 1H), 7.34 – 7.29 (m, 1H), 7.25 – 7.18 (m, 3H), 5.25 (q, *J* = 7.1 Hz, 1H), 4.30 – 4.21 (m, 2H), 2.33 (s, 3H), 1.80 (d, *J* = 7.1 Hz, 3H), 1.50 – 1.41 (m, 2H), 1.38 – 1.22 (m, 10H), 0.92 – 0.85 (m, 5H).

^{13}C NMR (101 MHz, $CDCl_3$) δ 210.7, 145.0, 135.5, 135.2, 130.3, 130.0, 127.0, 125.1, 123.5, 123.4, 121.2, 118.5, 113.7, 84.2, 82.8, 33.0, 32.0, 29.4, 29.4, 25.1, 22.8, 21.7, 14.3, 13.9, 9.4.

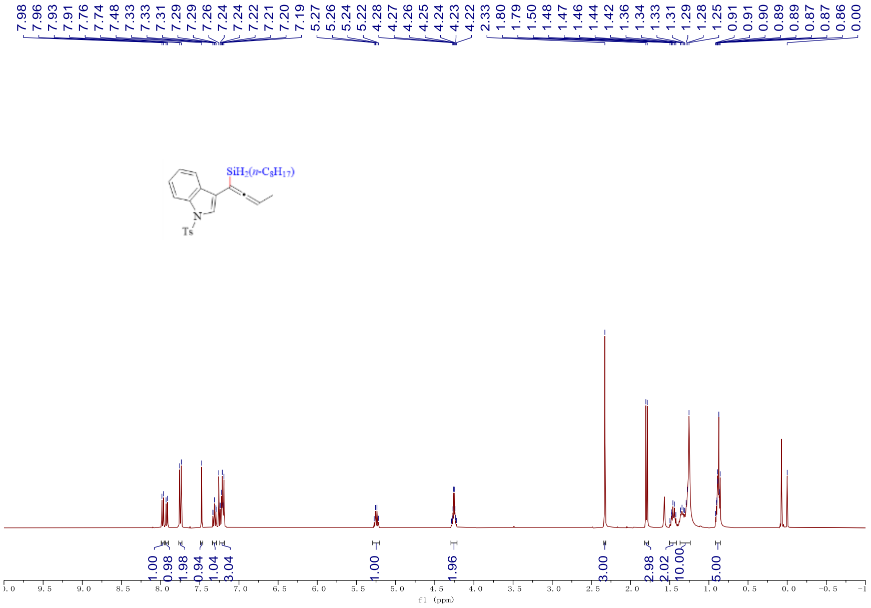

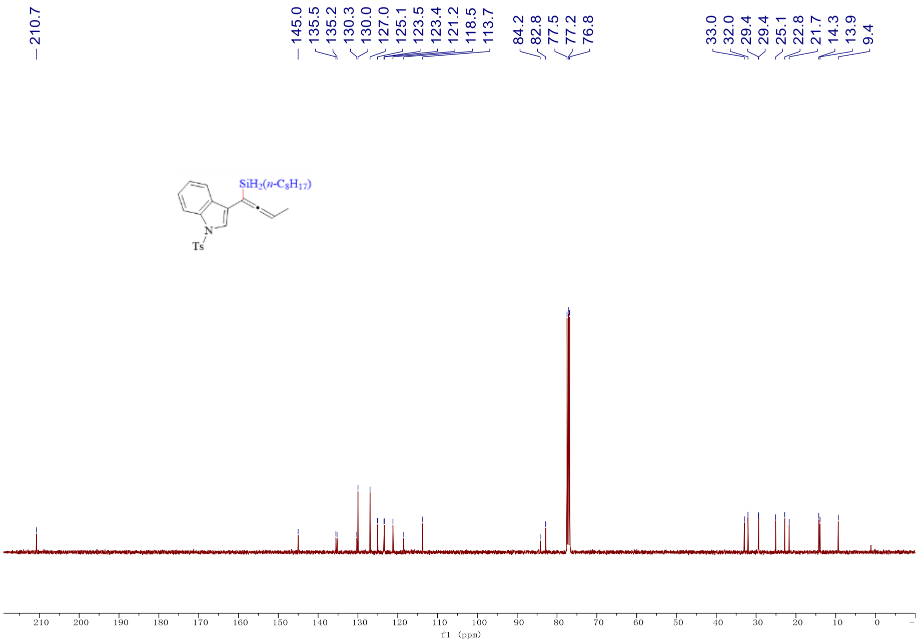

Octyl(1-(4-vinylphenyl)buta-1,2-dien-1-yl)silane (3vb)

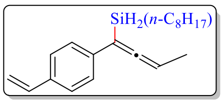

Prepared according to procedure 2 from **1v** (0.2 mmol, 30.8 mg) and **2b** (0.6 mmol, 86.4 mg). The reaction mixture was stirred at 60 °C for 1 h in 0.5 mL dry DCE. The product was isolated in 78% yield (46.6 mg) as colorless oil.

R*f*: 0.85 (petroleum ether).

HRMS (ESI) (m/z): Calcd for $C_{20}H_{31}Si$ $[M+H]^+$: 299.2195, found: 299.2198.

^{1}H NMR (400 MHz, $CDCl_3$) δ 7.37 – 7.30 (m, 4H), 6.69 (dd, *J* = 17.6, 10.9 Hz, 1H), 5.72 (dd, *J* = 17.6, 1.0 Hz, 1H), 5.27 – 5.19 (m, 2H), 4.29 – 4.21 (m, 2H), 1.75 (d, *J* = 7.1 Hz, 3H), 1.50 – 1.40 (m, 2H), 1.38 – 1.22 (m, 10H), 0.91 – 0.83 (m, 5H).

^{13}C NMR (101 MHz, $CDCl_3$) δ 210.6, 136.9, 136.6, 135.9, 127.4, 126.5, 113.5, 92.6, 83.0, 32.9, 32.0, 29.4, 29.4, 25.1, 22.8, 14.3, 13.4, 9.6.

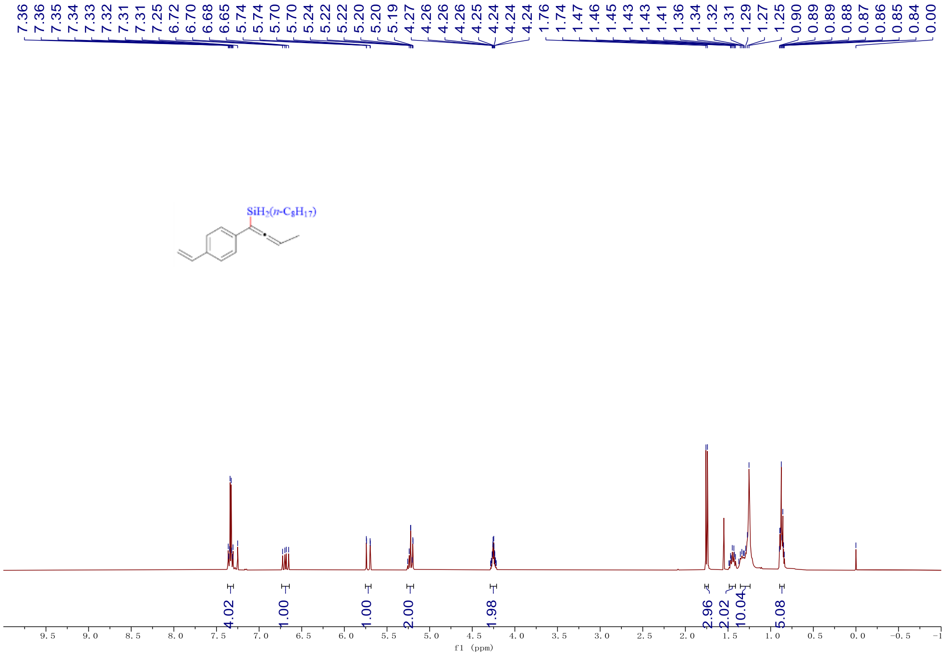

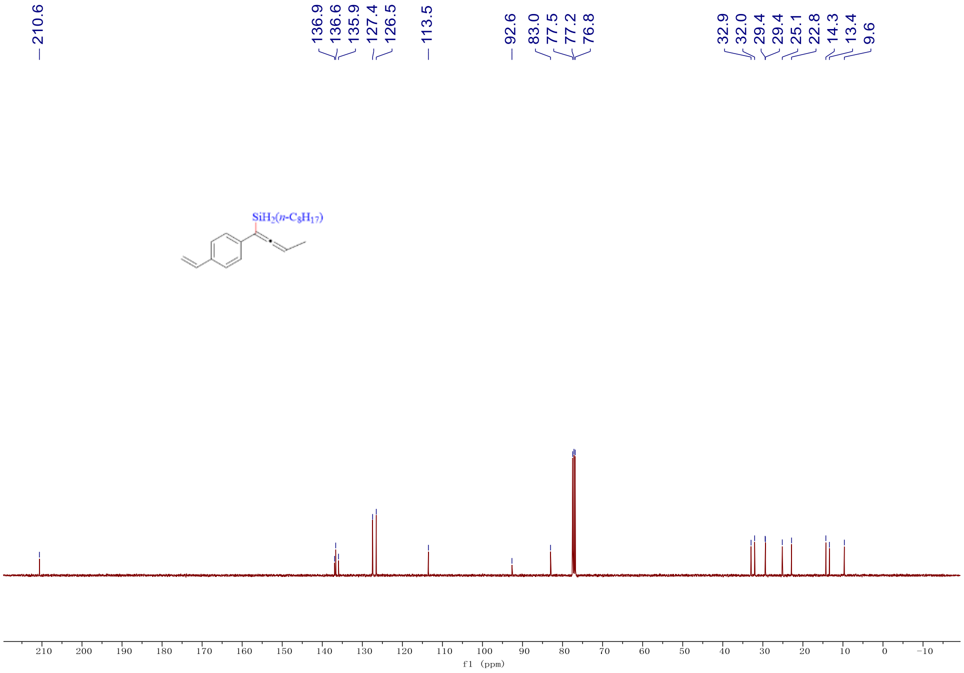

Octyl(1-(4-(prop-1-en-2-yl)phenyl)buta-1,2-dien-1-yl)silane (3wb)

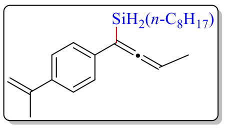

Prepared according to procedure 2 from **1w** (0.2 mmol, 33.6 mg) and **2b** (0.6 mmol, 86.4 mg). The reaction mixture was stirred at 60 °C for 1 h in 0.5 mL dry DCE. The product was isolated in 71% yield (44.4 mg) as colorless oil.

R*f*: 0.83 (petroleum ether).

HRMS (ESI) (m/z): Calcd for $C_{21}H_{33}Si$ $[M+H]^+$: 313.2352, found: 313.2347.

^{1}H NMR (400 MHz, $CDCl_3$) δ 7.45 – 7.38 (m, 2H), 7.36 – 7.28 (m, 2H), 5.36 (dd, *J* = 1.5, 0.7 Hz, 1H), 5.23 (q, *J* = 7.1 Hz, 1H), 5.05 (p, *J* = 1.5 Hz, 1H), 4.25 (m, 2H), 2.16 – 2.11 (m, 3H), 1.75 (d, *J* = 7.1 Hz, 3H), 1.52 – 1.39 (m, 2H), 1.40 – 1.23 (m, 10H), 0.90 – 0.84 (m, 5H).

^{13}C NMR (101 MHz, $CDCl_3$) δ 210.6, 143.0, 139.4, 136.5, 127.1, 125.8, 112.2, 92.5, 82.9, 32.9, 32.0, 29.4, 29.4, 25.2, 22.8, 21.9, 14.3, 13.4, 9.7.

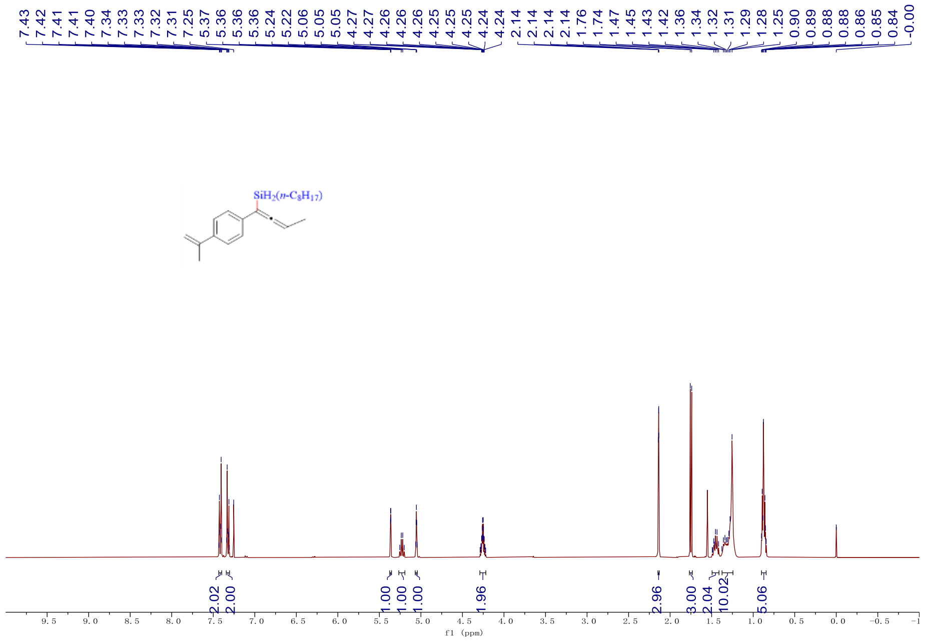

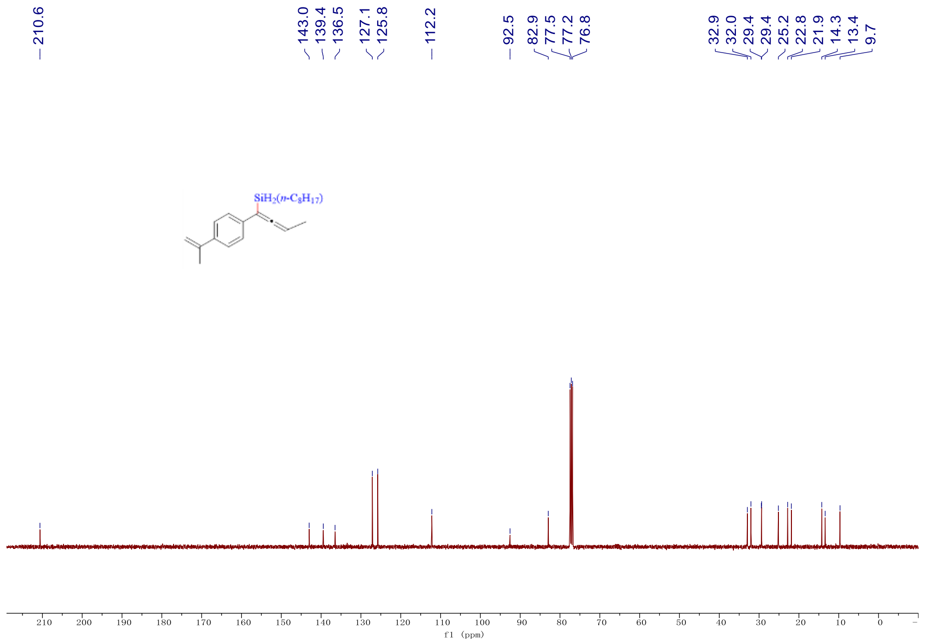

(1-(4-Ethynylphenyl)buta-1,2-dien-1-yl)(octyl)silane (3xb)

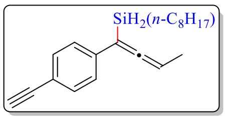

Prepared according to procedure 2 from **1x** (0.2 mmol, 30.4 mg) and **2b** (0.6 mmol, 86.4 mg). The reaction mixture was stirred at 40 °C for 3 h in 0.5 mL dry DCE. The product was isolated in 64% yield (38.0 mg) as pale-yellow oil.

R*f*: 0.53 (petroleum ether).

HRMS (ESI) (m/z): Calcd for $C_{20}H_{28}NaSi$ $[M+Na]^+$: 319.1858, found: 319.1871.

^{1}H NMR (400 MHz, $CDCl_3$) δ 7.44 – 7.40 (m, 2H), 7.33 – 7.29 (m, 2H), 5.25 (q, *J* = 7.1 Hz, 1H), 4.28 – 4.20 (m, 2H), 3.07 (s, 1H), 1.75 (d, *J* = 7.1 Hz, 3H), 1.49 – 1.40 (m, 2H), 1.37 – 1.23 (m, 10H), 0.91 – 0.82 (m, 5H).

^{13}C NMR (101 MHz, $CDCl_3$) δ 211.0, 138.2, 132.4, 127.2, 120.1, 92.7, 83.9, 83.3, 77.4, 32.9, 32.0, 29.4, 29.3, 25.1, 22.8, 14.2, 13.3, 9.6.

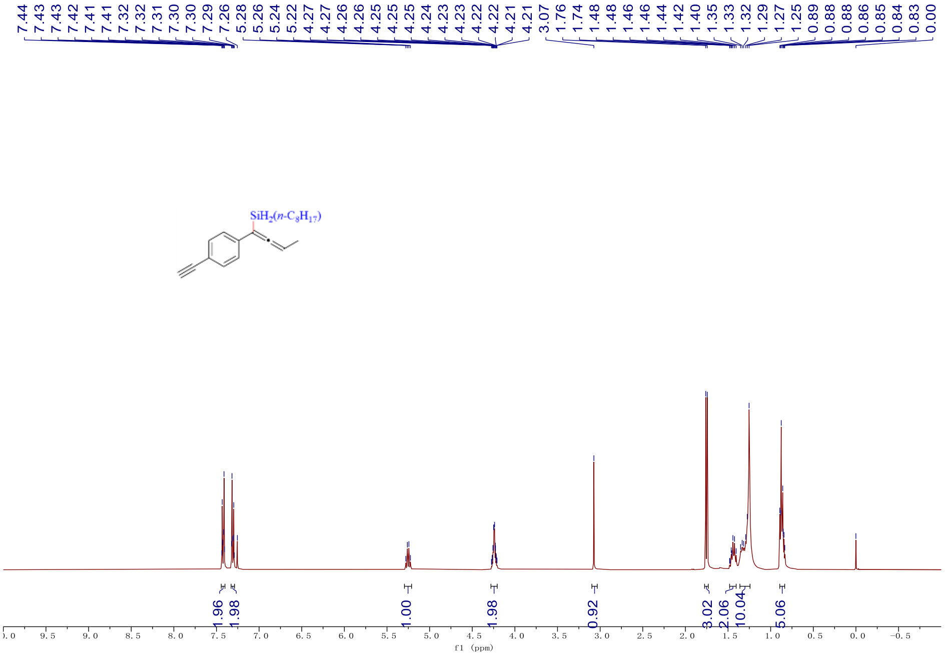

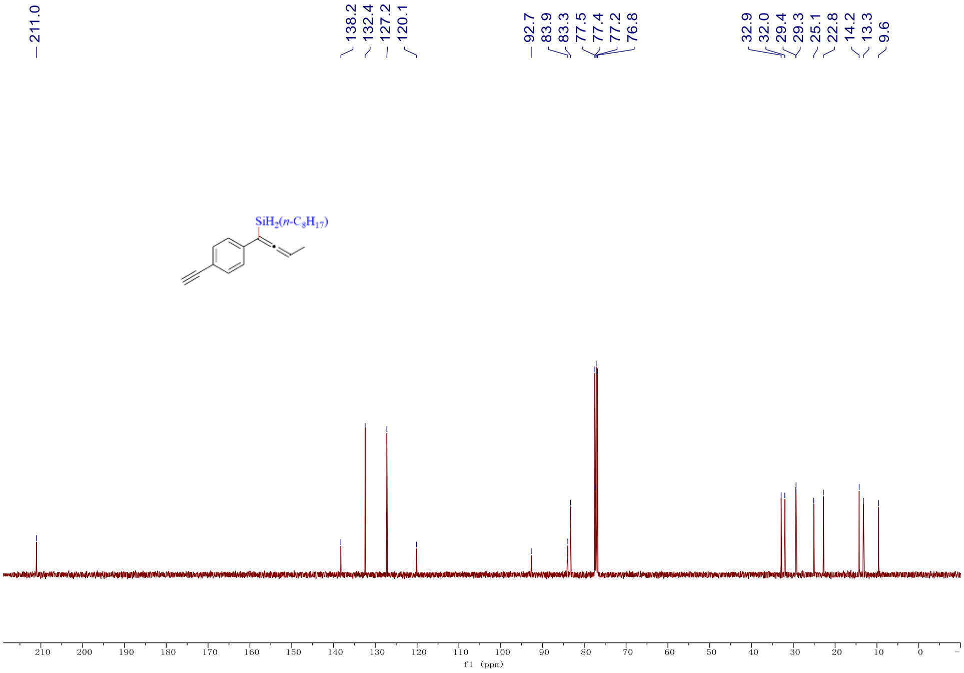

Trimethyl((4-(1-(octylsilyl)buta-1,2-dien-1-yl)phenyl)ethynyl)silane (3yb)

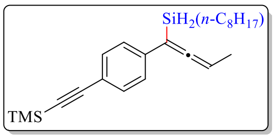

Prepared according to procedure 2 from **1y** (0.2 mmol, 44.9 mg) and **2b** (0.6 mmol, 86.4 mg). The reaction mixture was stirred at 40 °C for 1 h in 1.0 mL dry DCE. The product was isolated in 70% yield (51.6 mg) as colorless oil.

R*f*: 0.70 (petroleum ether).

HRMS (ESI) (m/z): Calcd for $C_{23}H_{37}Si_2$ $[M+H]^+$: 369.2434, found: 369.2432.

^{1}H NMR (400 MHz, $CDCl_3$) δ 7.41 – 7.36 (m, 2H), 7.30 – 7.26 (m, 2H), 5.24 (q, *J* = 7.1 Hz, 1H), 4.28 – 4.20 (m, 2H), 1.75 (d, *J* = 7.1 Hz, 3H), 1.49 – 1.38 (m, 2H), 1.37 – 1.21 (m, 10H), 0.93 – 0.80 (m, 5H), 0.25 (s, 9H).

^{13}C NMR (101 MHz, $CDCl_3$) δ 210.9, 137.8, 132.3, 127.1, 121.1, 105.4, 94.4, 92.7, 83.3, 32.9, 32.0, 29.4, 29.3, 25.1, 22.8, 14.3, 13.3, 9.6, 0.2.

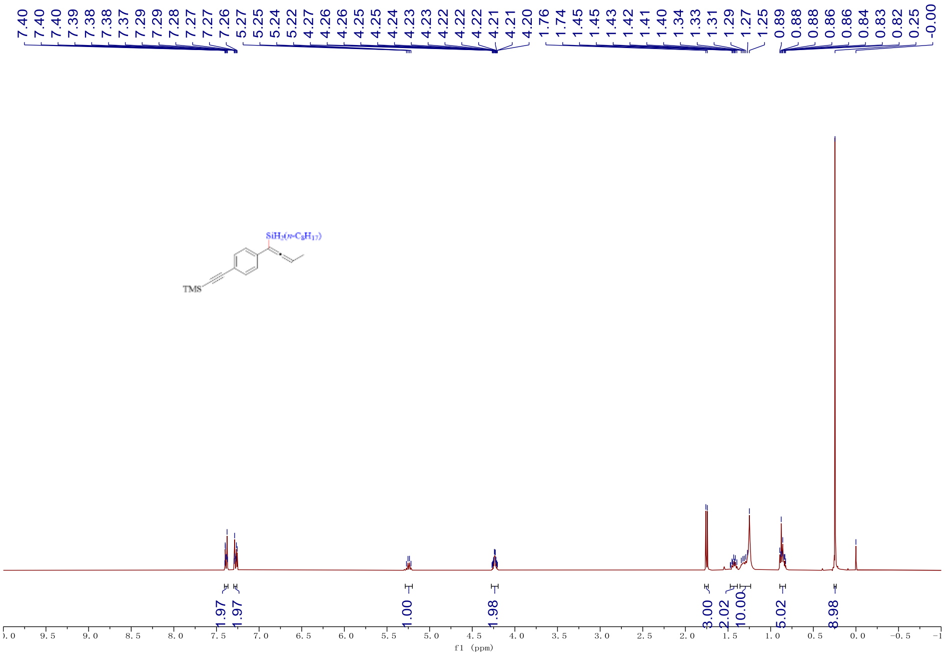

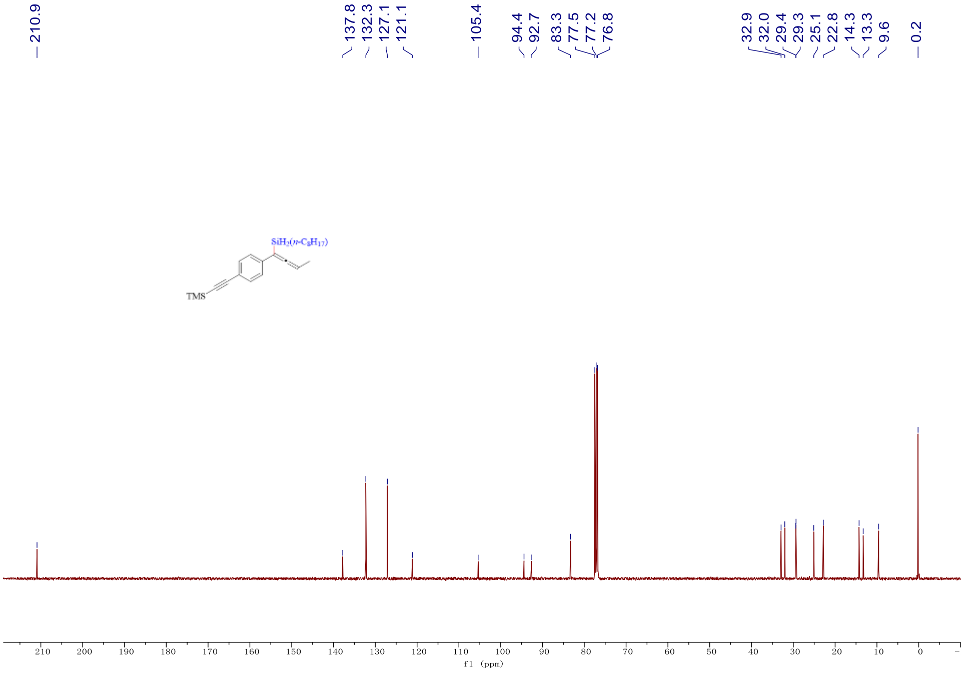

(1-(Cyclohex-1-en-1-yl)buta-1,2-dien-1-yl)(octyl)silane (3zb)

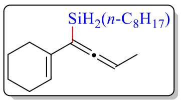

Prepared according to procedure 3 from **1z** (0.2 mmol, 26.4 mg) and **2b** (0.6 mmol, 86.4 mg). The reaction mixture was stirred at 70 °C for 1 h in 0.5 mL dry DCM. The product was isolated in 68% yield (37.6 mg) as colorless oil.

R*f*: 0.75 (petroleum ether).

HRMS (ESI) (m/z): Calcd for $C_{18}H_{33}Si$ $[M+H]^+$: 277.2352, found: 277.2344.

^{1}H NMR (400 MHz, $CDCl_3$) δ 5.70 (t, *J* = 3.4 Hz, 1H), 5.04 (q, *J* = 7.1 Hz, 1H), 4.11 – 4.04 (m, 2H), 2.19 – 2.02 (m, 4H), 1.66 (d, *J* = 7.0 Hz, 3H), 1.64 – 1.54 (m, 4H), 1.47 – 1.37 (m, 2H), 1.36 – 1.24 (m, 10H), 0.88 (t, *J* = 6.7 Hz, 3H), 0.82 – 0.76 (m, 2H).

^{13}C NMR (101 MHz, $CDCl_3$) δ 209.4, 134.4, 125.7, 95.0, 82.7, 33.0, 32.1, 29.5, 29.4, 27.7, 26.2, 25.3, 23.1, 22.8, 22.5, 14.3, 13.9, 9.6.

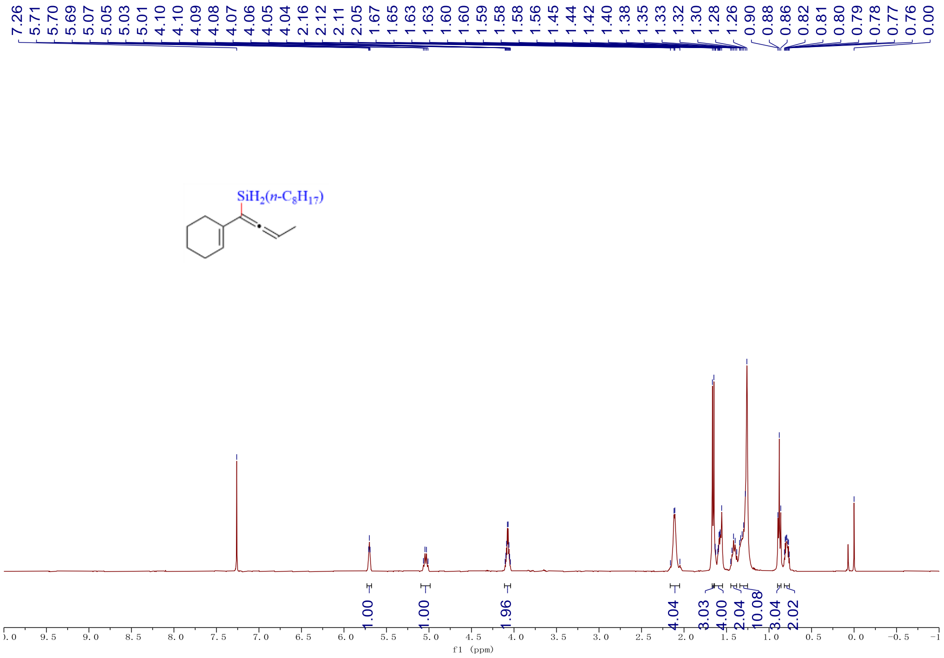

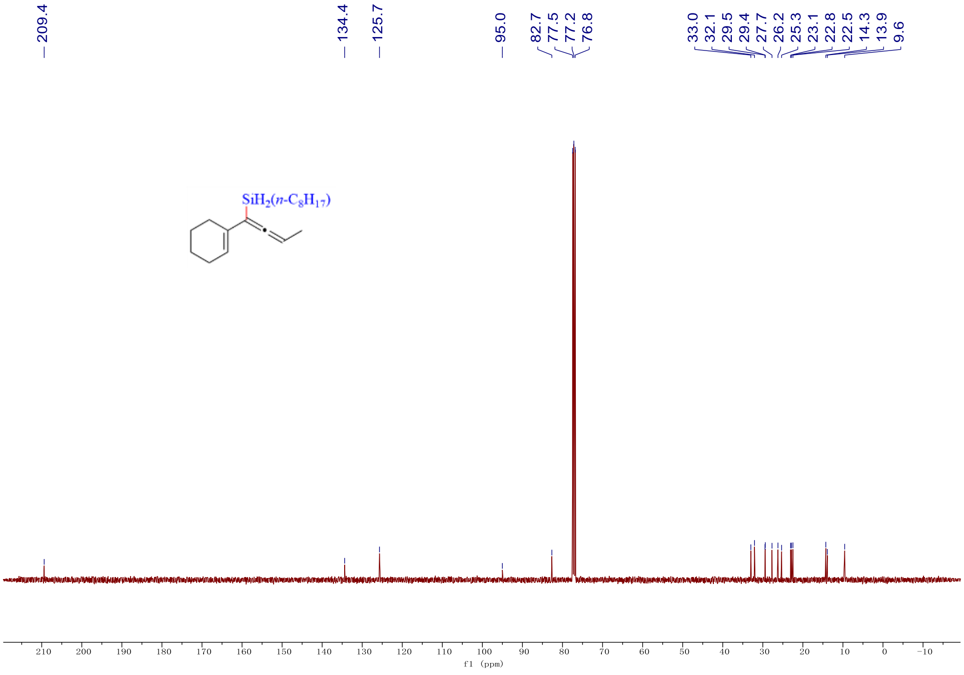

(*E*)-(1-(cyclooct-1-en-1-yl)buta-1,2-dien-1-yl)(octyl)silane (3aab)

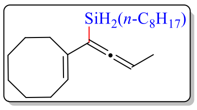

Prepared according to procedure 3 from **1aa** (0.2 mmol, 32.1 mg) and **2b** (0.6 mmol, 86.4 mg). The reaction mixture was stirred at 70 °C for 4 h in 0.5 mL dry DCM. The product was isolated in 69% yield (42.1 mg) as colorless oil.

R*f*: 0.80 (petroleum ether).

HRMS (ESI) (m/z): Calcd for $C_{20}H_{37}Si$ $[M+H]^+$: 305.2665, found: 305.2658.

^{1}H NMR (400 MHz, $CDCl_3$) δ 5.64 (t, *J* = 8.1 Hz, 1H), 5.02 (q, *J* = 7.0 Hz, 1H), 4.13 – 4.05 (m, 2H), 2.42 – 2.33 (m, 2H), 2.23 – 2.16 (m, 2H), 1.66 (d, *J* = 7.0 Hz, 3H), 1.53 – 1.38 (m, 10H), 1.36 – 1.24 (m, 10H), 0.88 (t, *J* = 6.8 Hz, 3H), 0.83 – 0.76 (m, 2H).

^{13}C NMR (101 MHz, $CDCl_3$) δ 209.7, 137.6, 128.5, 94.6, 82.2, 33.0, 32.1, 30.6, 29.5, 29.4, 28.9, 27.8, 27.7, 27.1, 26.3, 25.3, 22.8, 14.3, 13.8, 9.7.

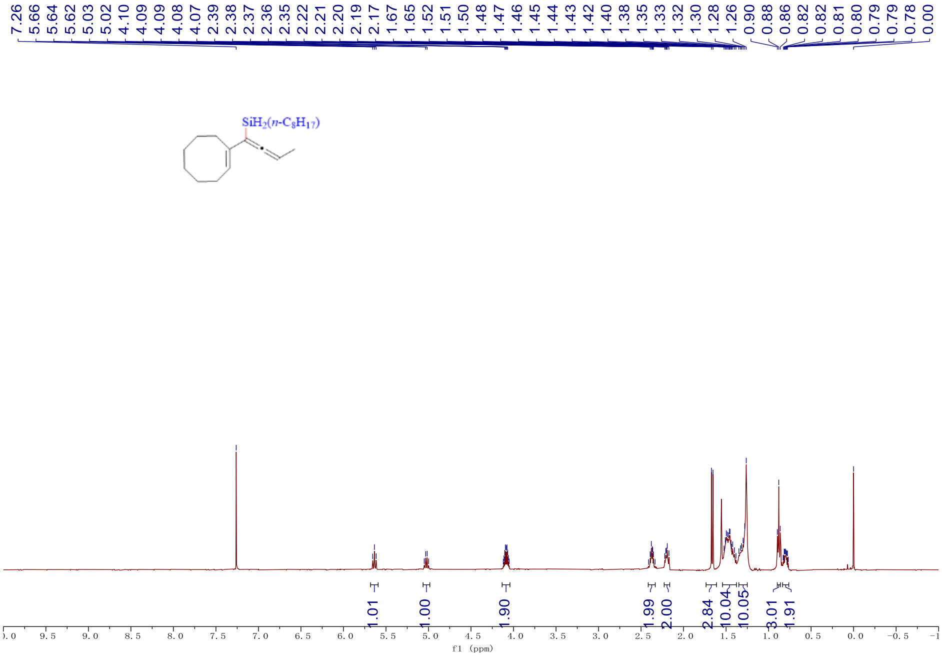

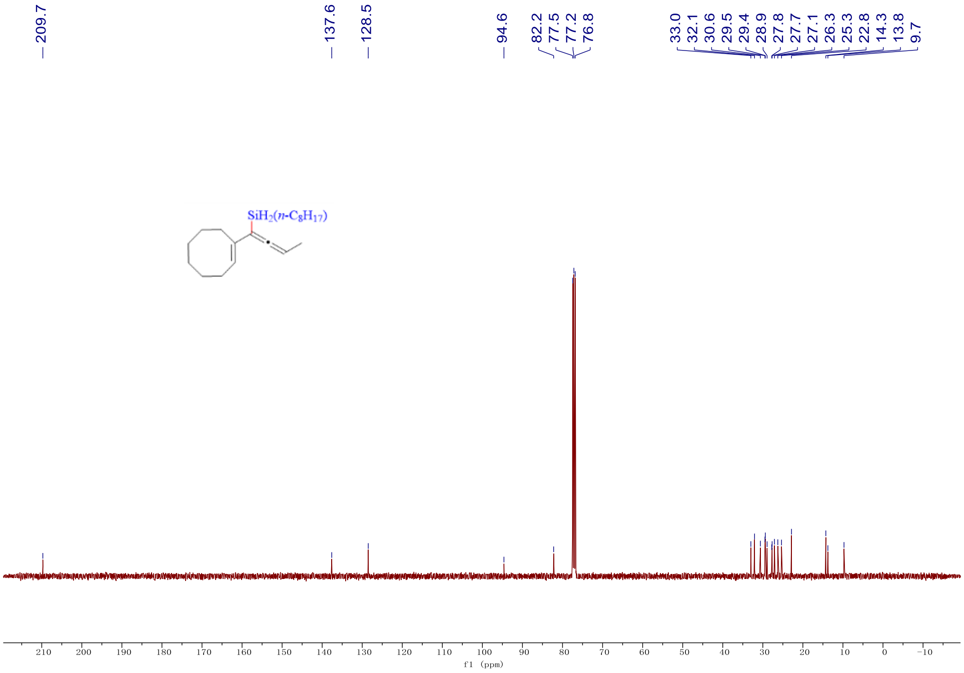

(3,5-Dimethylphenyl)(1-phenylbuta-1,2-dien-1-yl)silane (3ac)

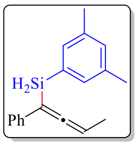

Prepared according to procedure 2 from **1a** (0.2 mmol, 25.6 mg) and **2c** (0.6 mmol, 81.8 mg). The reaction mixture was stirred at 40 °C for 1 h in 1.0 mL dry DCE. The product was isolated in 61% yield (32.4 mg) as colorless oil.

R*f*: 0.60 (petroleum ether).

HRMS (ESI) (m/z): Calcd for $C_{18}H_{21}Si$ $[M+H]^+$: 265.1413, found: 265.1408.

^{1}H NMR (400 MHz, $CDCl_3$) δ 7.38 (dd, *J* = 8.3, 1.0 Hz, 2H), 7.30 – 7.23 (m, 4H), 7.19 – 7.14 (m, 1H), 7.03 (s, 1H), 5.22 (q, *J* = 7.1 Hz, 1H), 4.86 (d, *J* = 6.8 Hz, 1H), 4.83 (d, *J* = 6.8 Hz, 1H), 2.30 (s, 6H), 1.73 (d, *J* = 7.1 Hz, 3H).

^{13}C NMR (101 MHz, $CDCl_3$) δ 211.9, 137.5, 137.0, 133.3, 131.8, 131.1, 128.7, 127.5, 126.6, 92.7, 83.2, 21.4, 13.3.

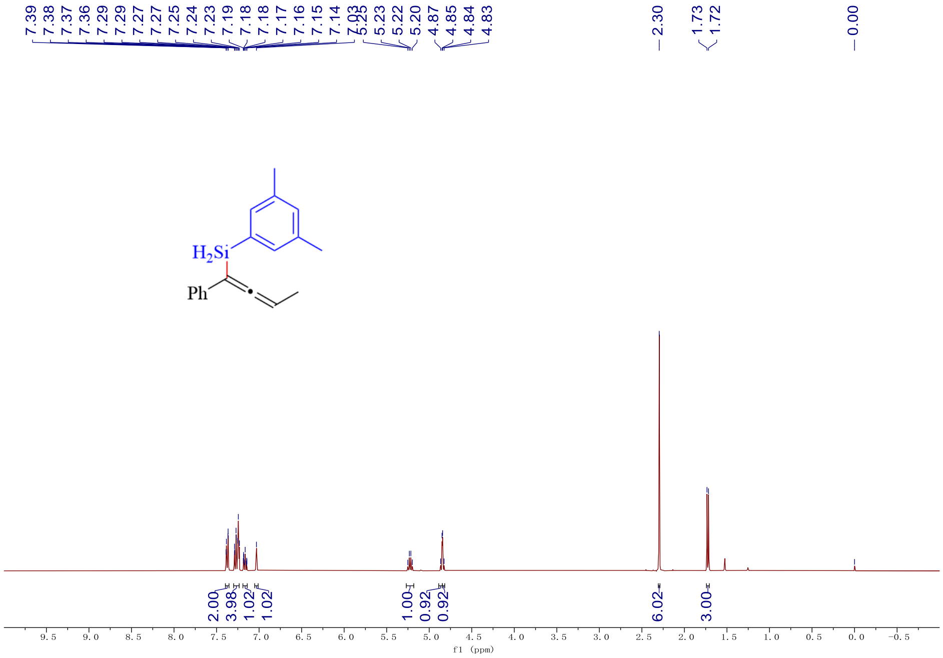

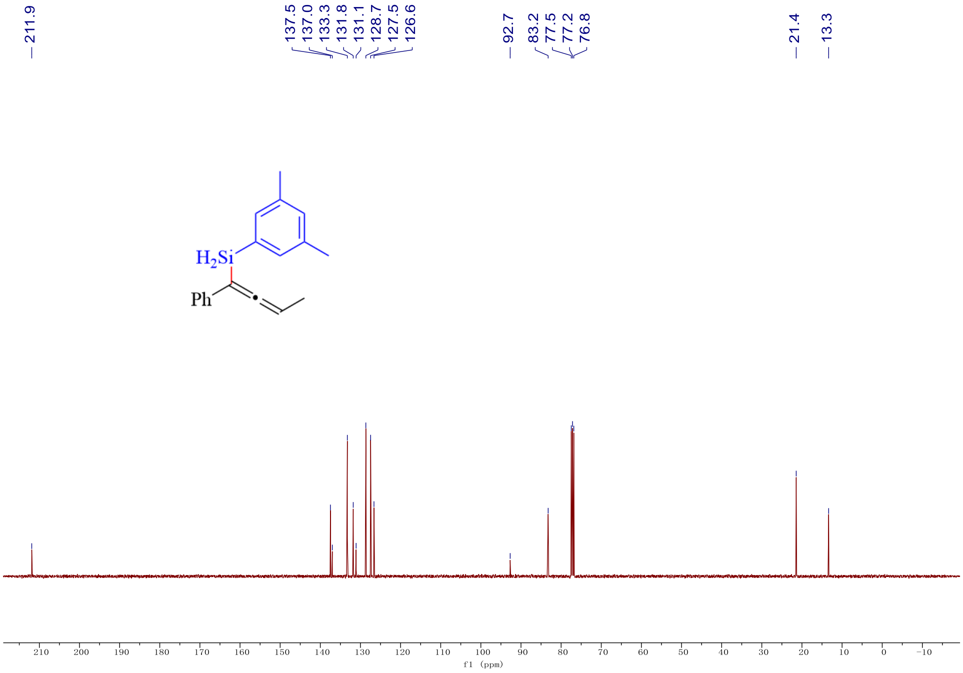

(4-Fluorophenyl)(1-phenylbuta-1,2-dien-1-yl)silane (3ad)

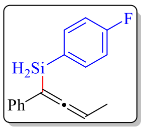

Prepared according to procedure 2 from **1a** (0.2 mmol, 25.6 mg) and **2d** (0.6 mmol, 75.7 mg). The reaction mixture was stirred at 50 °C for 1 h in 1.0 mL dry DCE. The product was isolated in 71% yield (36.1 mg) as colorless oil.

R_f: 0.58 (petroleum ether).

HRMS (EI) (m/z): Calcd for $C_{16}H_{15}FSi$ $[M]^+$: 254.0927, found: 254.0932.

^{1}H NMR (400 MHz, $CDCl_3$) δ 7.64 – 7.55 (m, 2H), 7.39 – 7.32 (m, 2H), 7.32 – 7.22 (m, 2H), 7.22 – 7.13 (m, 1H), 7.05 (tt, *J* = 9.1, 2.3 Hz, 2H), 5.22 (q, *J* = 7.1 Hz, 1H), 4.90 (d, *J* = 6.8 Hz, 1H), 4.87 (d, *J* = 6.8 Hz, 1H), 1.70 (d, *J* = 7.1 Hz, 3H).

^{13}C NMR (101 MHz, $CDCl_3$) δ 212.0, 164.4 (d, *J* = 249.4 Hz), 137.6 (d, *J* = 7.7 Hz), 136.7, 128.7, 127.4, 126.9 (d, *J* = 3.7 Hz), 126.8, 115.5 (d, *J* = 19.9 Hz), 92.5, 83.5, 13.3.

^{19}F NMR (376 MHz, $CDCl_3$) δ -110.4.

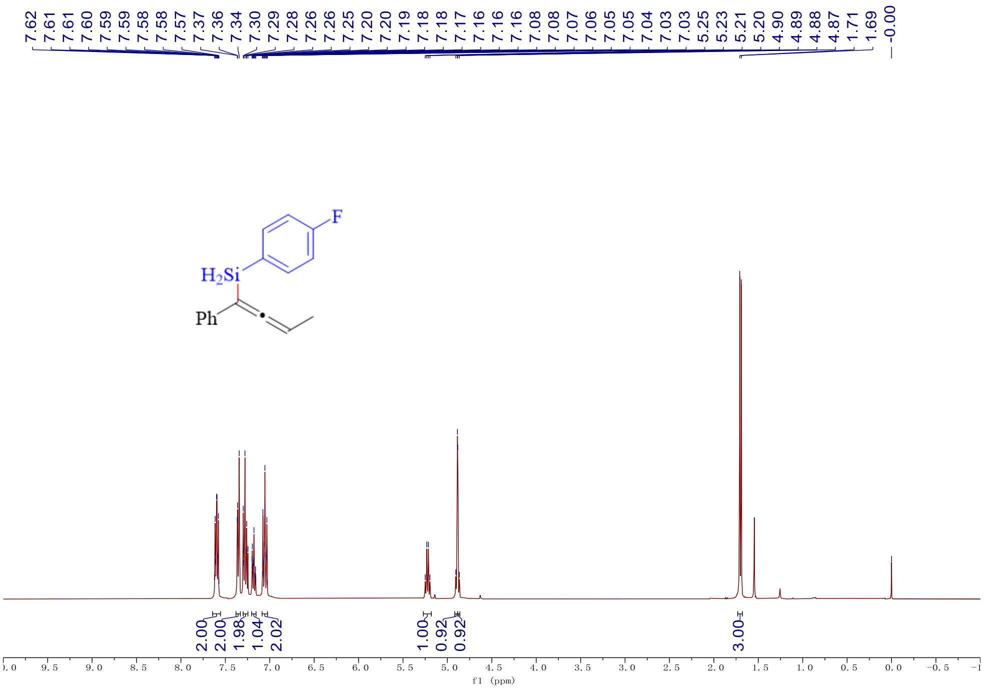

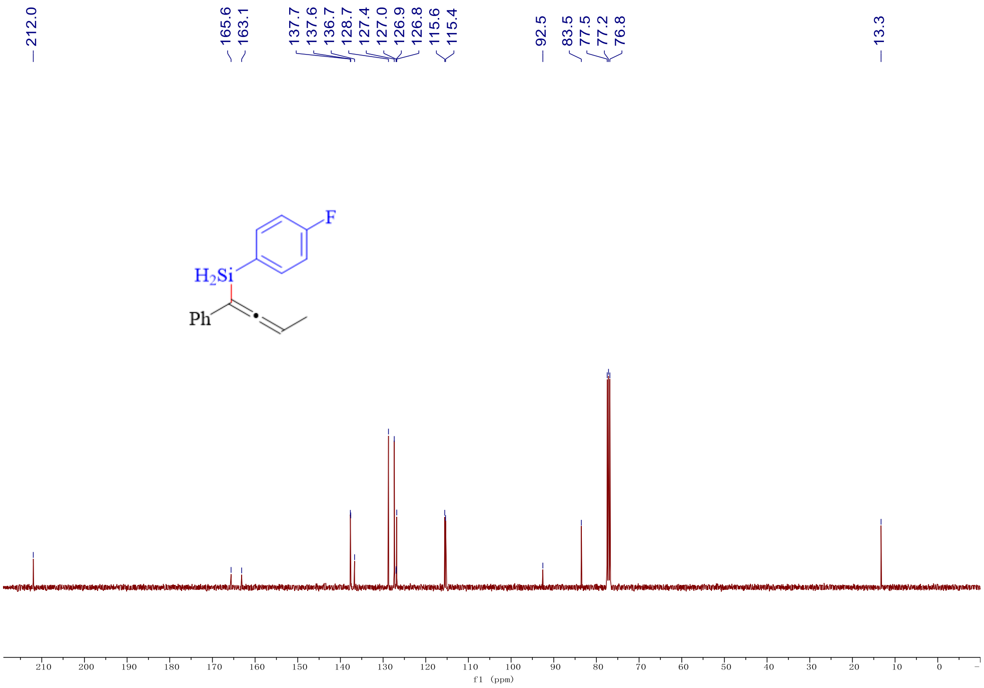
212.0
165.6
163.1
137.7
137.6
136.7
128.7
127.4
127.0
126.9
126.8
115.6
115.4
92.5
83.5
77.5
77.2
76.8
13.3
F
H_2Si
Ph
f1 (ppm)

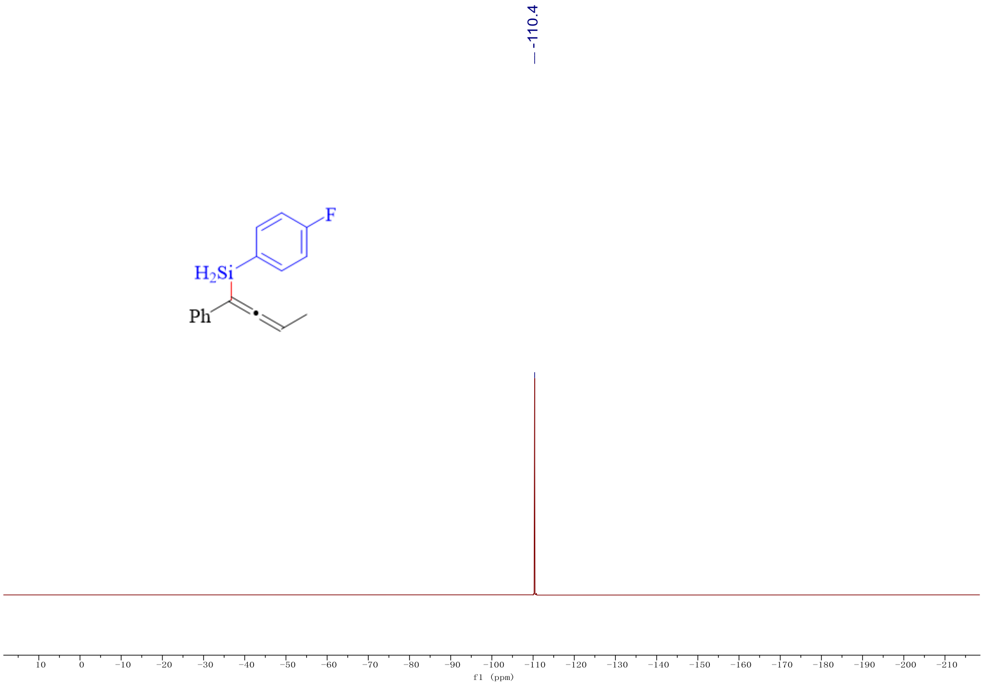
-110.4
F
H_2Si
Ph
f1 (ppm)

(4-Chlorophenyl)(1-phenylbuta-1,2-dien-1-yl)silane (3ae)

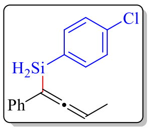

Prepared according to procedure 2 from **1a** (0.2 mmol, 25.6 mg) and **2e** (0.6 mmol, 85.6 mg). The reaction mixture was stirred at 60 °C for 1 h in 1.0 mL dry DCE. The product was isolated in 71% yield (38.5 mg) as colorless oil.

R*f*: 0.65 (petroleum ether).

HRMS (EI) (m/z): Calcd for $C_{16}H_{15}ClSi$ $[M]^+$: 270.0632, found: 270.0636.

^{1}H NMR (400 MHz, $CDCl_3$) δ 7.56 – 7.52 (m, 2H), 7.36 – 7.31 (m, 4H), 7.30 – 7.25 (m, 2H), 7.21 – 7.15 (m, 1H), 5.23 (q, *J* = 7.1 Hz, 1H), 4.88 (d, *J* = 6.8 Hz, 1H), 4.86 (d, *J* = 6.8 Hz, 1H), 1.70 (d, *J* = 7.2 Hz, 3H).

^{13}C NMR (101 MHz, $CDCl_3$) δ 212.0, 136.9, 136.6, 136.5, 129.8, 128.8, 128.4, 127.4, 126.8, 92.3, 83.6, 13.3.

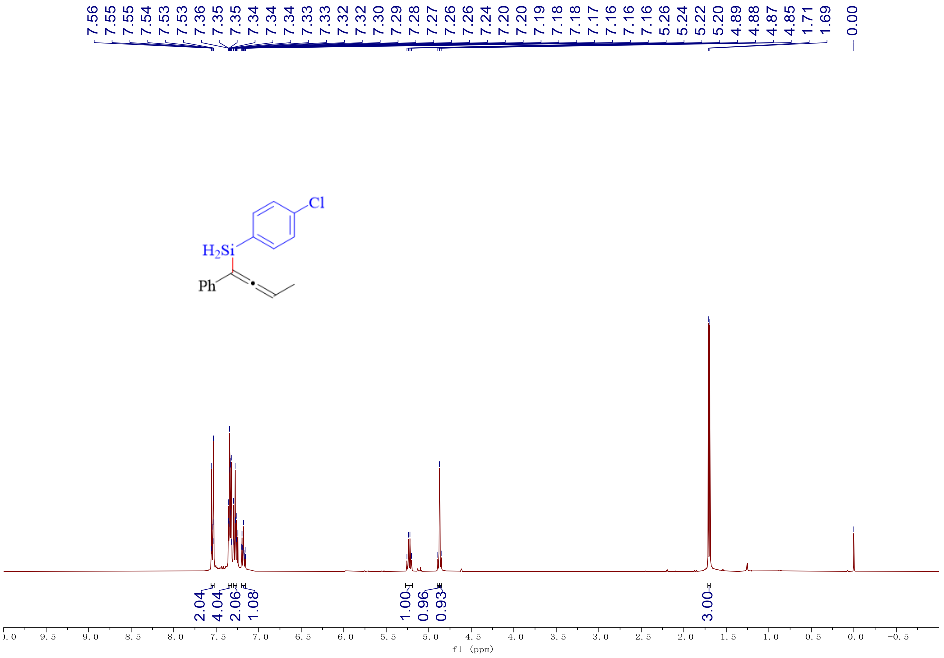

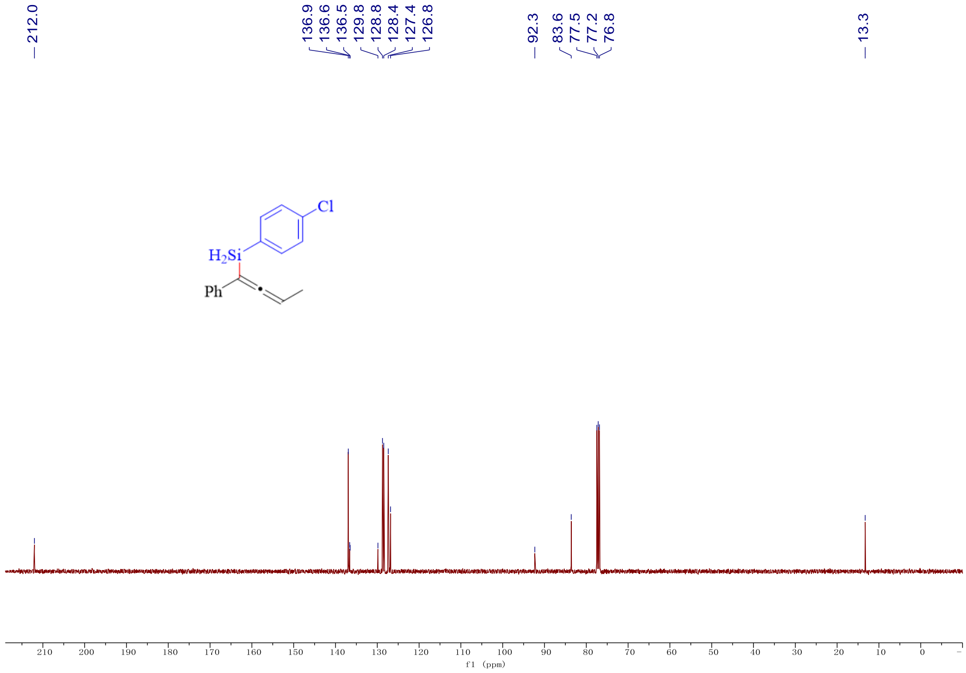

(1-Phenylbuta-1,2-dien-1-yl)(4-(trifluoromethyl)phenyl)silane (3af)

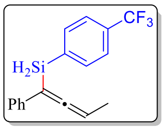

Prepared according to procedure 2 from **1a** (0.2 mmol, 25.6 mg) and **2f** (0.6 mmol, 105.7 mg). The reaction mixture was stirred at 60 °C for 1 h in 1.0 mL dry DCE. The product was isolated in 62% yield (37.7 mg) as colorless oil.

R*f*: 0.60 (petroleum ether).

HRMS (EI) (m/z): Calcd for $C_{17}H_{15}F_3Si$ $[M]^+$: 304.0895, found: 304.0900.

^{1}H NMR (400 MHz, $CDCl_3$) δ 7.77 – 7.71 (m, 2H), 7.60 (dd, *J* = 7.5, 0.8 Hz, 2H), 7.39 – 7.33 (m, 2H), 7.32 – 7.26 (m, 2H), 7.22 – 7.16 (m, 1H), 5.29 (q, *J* = 7.2 Hz, 1H), 4.93 (d, *J* = 6.8 Hz, 1H), 4.91 (d, *J* = 6.8 Hz, 1H), 1.70 (d, *J* = 7.1 Hz, 3H).

^{13}C NMR (101 MHz, $CDCl_3$) δ 212.2, 136.7, 136.4, 135.9, 131.9 (q, *J* = 32.2 Hz), 128.8, 127.3, 126.9, 124.7 (q, *J* = 3.7 Hz), 124.2 (q, *J* = 272.3 Hz), 92.0, 83.8, 13.2.

^{19}F NMR (376 MHz, $CDCl_3$) δ -63.0.

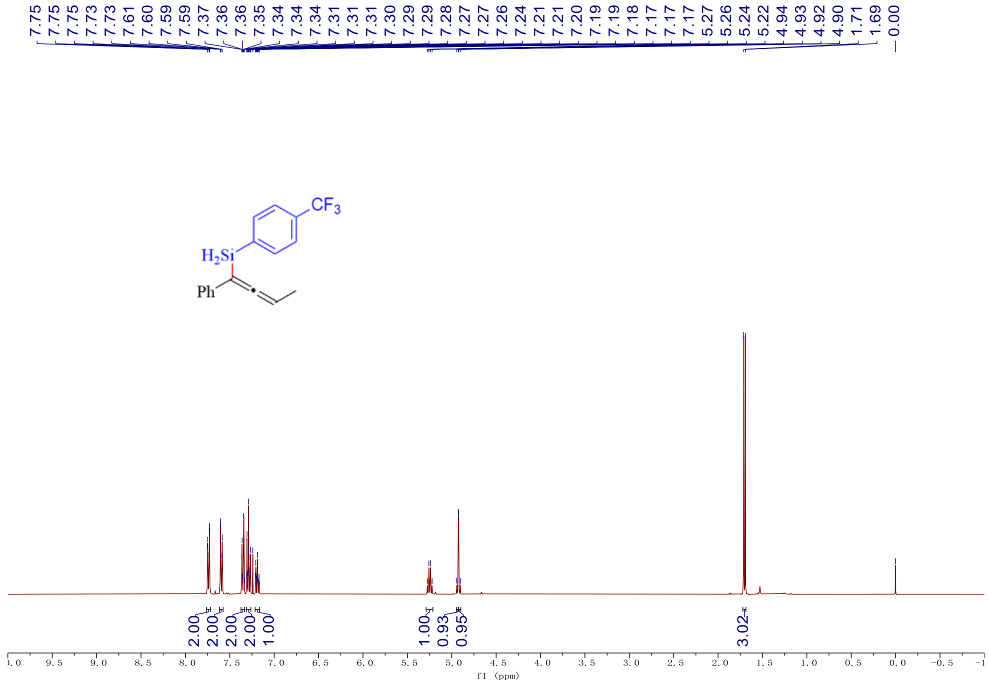

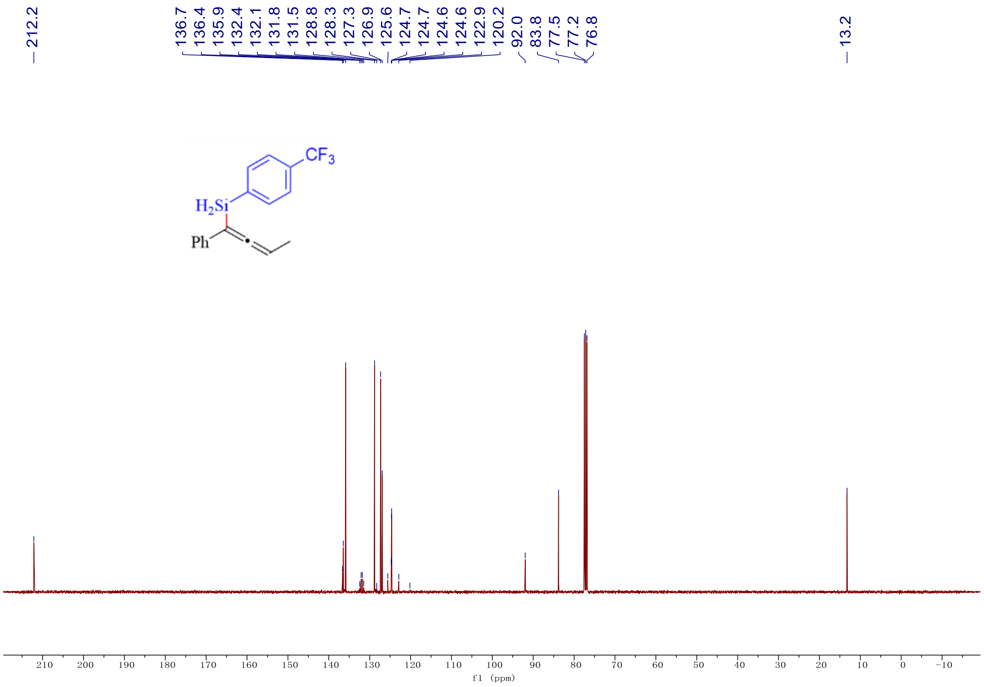
212.2
136.7
136.4
135.9
132.4
132.1
131.8
131.5
128.8
128.3
127.3
126.9
125.6
124.7
124.7
124.6
124.6
122.9
120.2
92.0
83.8
77.5
77.2
76.8
13.2
CF3
H2Si
Ph
f1 (ppm)

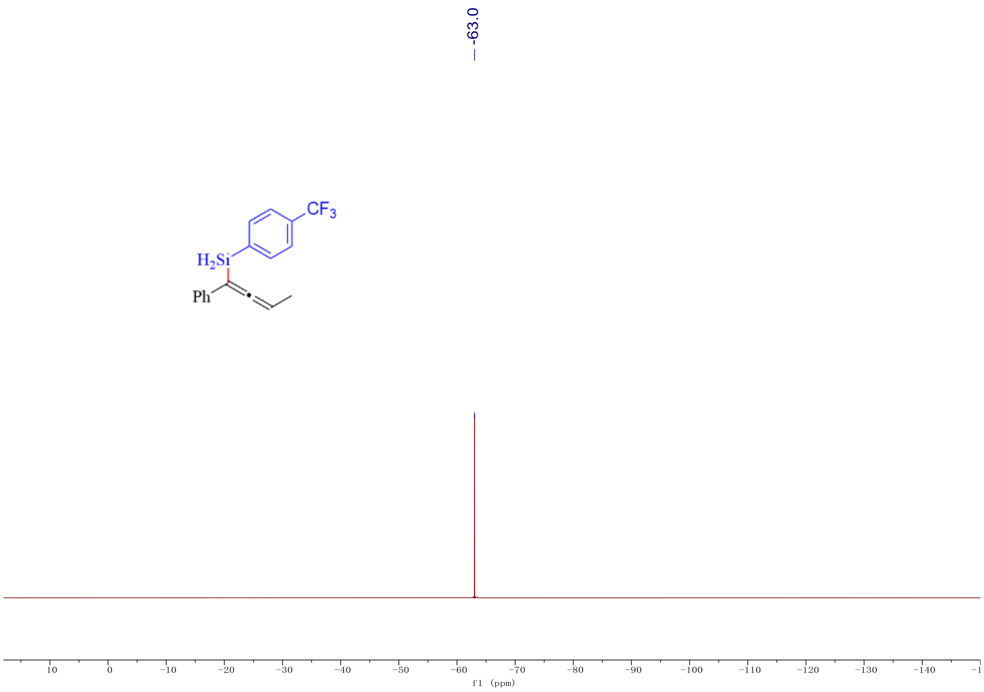
-63.0
CF3
H2Si
Ph
f1 (ppm)

(4-Methoxyphenyl)(1-phenylbuta-1,2-dien-1-yl)silane (3ag)

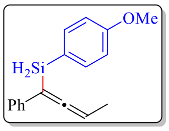

Prepared according to procedure 2 from **1a** (0.2 mmol, 25.6 mg) and **2g** (0.6 mmol, 82.9 mg). The reaction mixture was stirred at 60 °C for 1 h in 1.0 mL dry DCE. The product was isolated in 78% yield (41.7 mg) as colorless oil.

R*f*: 0.40 (ethyl acetate : petroleum ether = 1:100).

HRMS (EI) (m/z): Calcd for $C_{17}H_{18}Si$ $[M]^+$: 266.1127, found: 267.1127.

^{1}H NMR (400 MHz, $CDCl_3$) δ 7.57 – 7.53 (m, 2H), 7.39 – 7.34 (m, 2H), 7.30 – 7.24 (m, 2H), 7.20 – 7.14 (m, 1H), 6.93 – 6.88 (m, 2H), 5.22 (q, *J* = 7.1 Hz, 1H), 4.88 (d, *J* = 6.8 Hz, 1H), 4.86 (d, *J* = 6.8 Hz, 1H), 3.81 (s, 3H), 1.71 (d, *J* = 7.1 Hz, 3H).

^{13}C NMR (101 MHz, $CDCl_3$) δ 211.9, 161.2, 137.1, 137.0, 128.7, 127.4, 126.6, 122.0, 114.0, 92.9, 83.2, 55.2, 13.4.

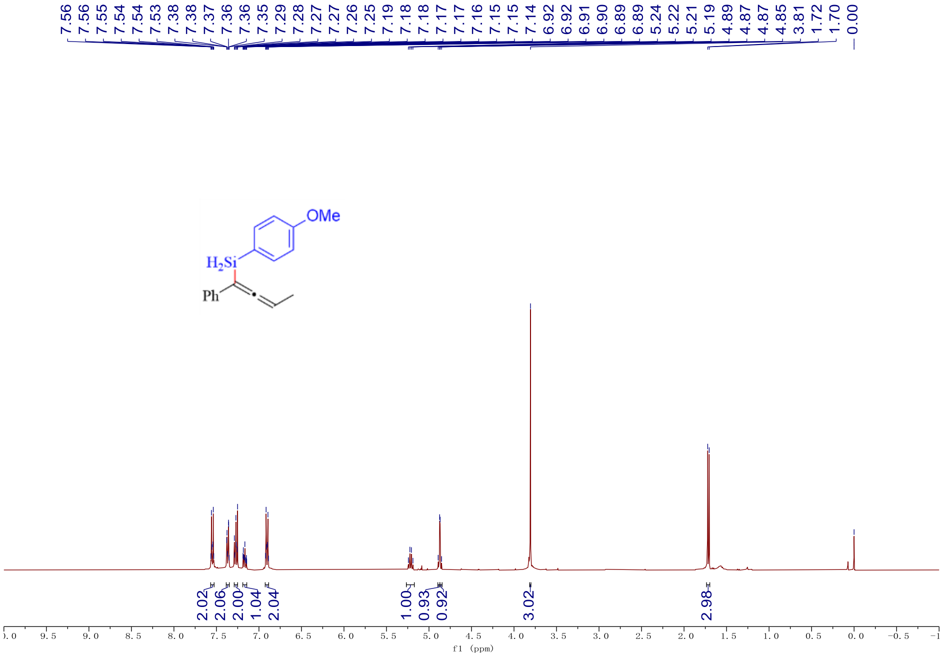

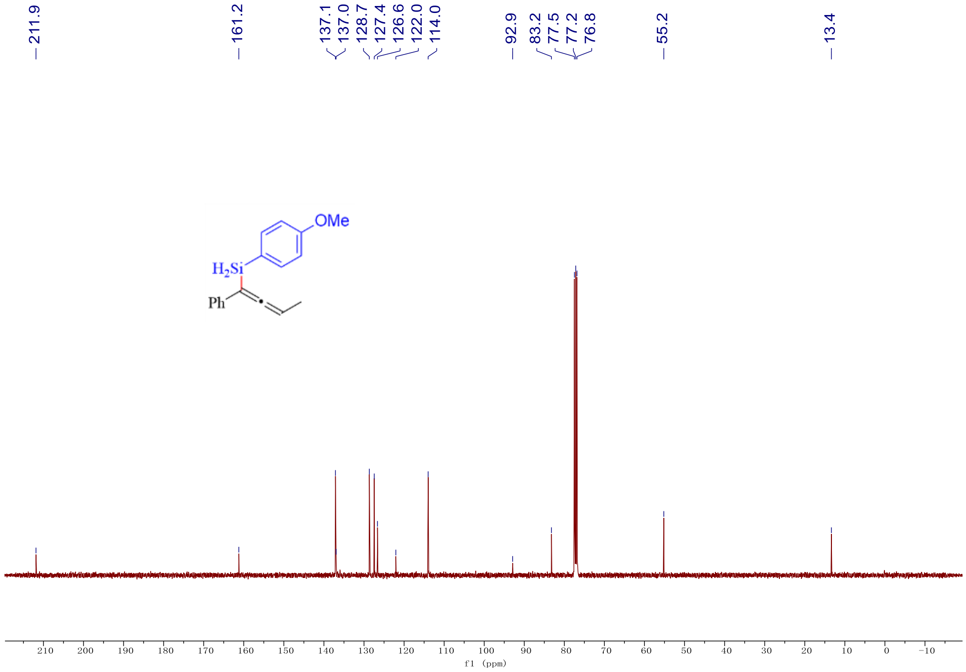

_N,N_-dimethyl-4-((1-phenylbuta-1,2-dien-1-yl)silyl)aniline (3ah)

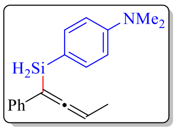

Prepared according to procedure 2 from **1a** (0.2 mmol, 25.6 mg) and **2h** (0.6 mmol, 90.8 mg). The reaction mixture was stirred at 60 °C for 1 h in 0.5 mL dry DCE. The product was isolated in 77% yield (43.1 mg) as pale-yellow oil.

R*f*: 0.53 (ethyl acetate : petroleum ether = 1:50).

HRMS (ESI) (m/z): Calcd for $C_{18}H_{22}NSi$ $[M+H]^+$: 280.1522, found: 280.1523.

^{1}H NMR (400 MHz, $CDCl_3$) δ 7.51 – 7.45 (m, 2H), 7.40 – 7.36 (m, 2H), 7.29 – 7.23 (m, 2H), 7.15 (t, *J* = 7.3 Hz, 1H), 6.73 – 6.68 (m, 2H), 5.21 (q, *J* = 7.1 Hz, 1H), 4.87 (d, *J* = 6.8 Hz, 1H), 4.85 (d, *J* = 6.8 Hz, 1H), 2.95 (s, 6H), 1.73 (d, *J* = 7.1 Hz, 3H).

^{13}C NMR (101 MHz, $CDCl_3$) δ 211.7, 151.7, 137.2, 136.7, 128.6, 127.5, 126.5, 115.7, 112.1, 93.3, 82.9, 40.2, 13.4.

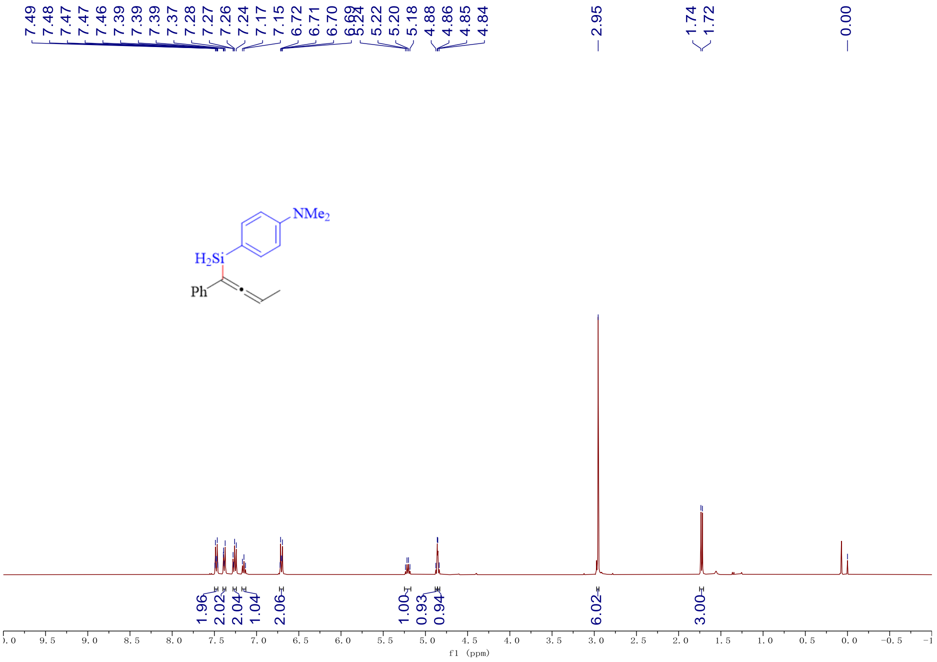

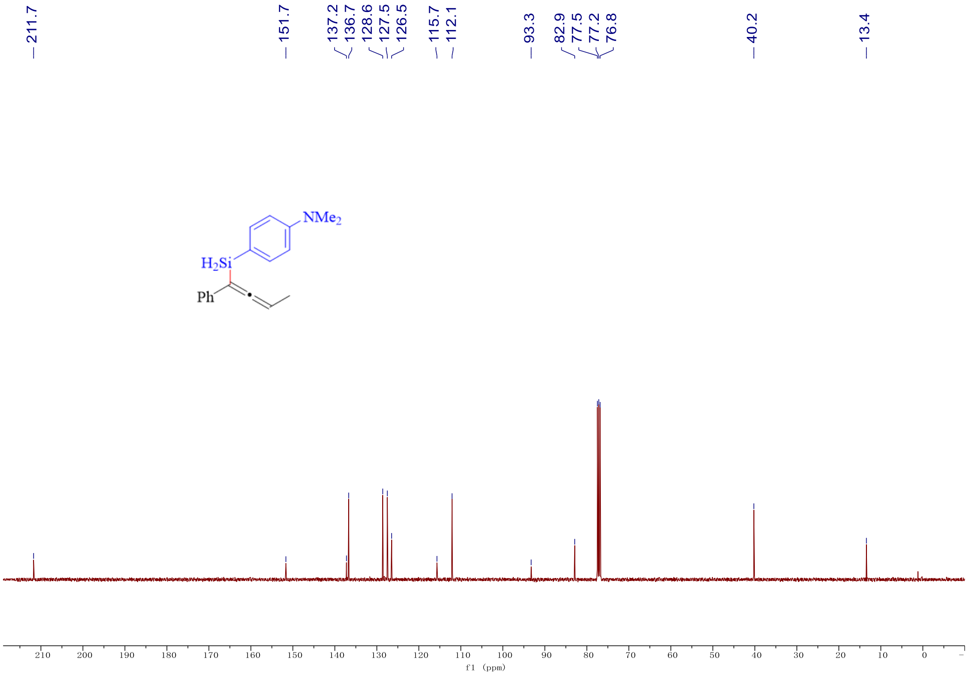

(1-([1,1'-biphenyl]-4-yl)buta-1,2-dien-1-yl)(naphthalen-2-yl)silane (3ei)

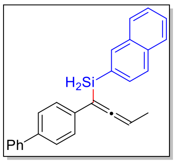

Prepared according to procedure 2 from **1e** (0.2 mmol, 25.6 mg) and **2i** (0.6 mmol, 90.8 mg). The reaction mixture was stirred at 40 °C for 1 h in 1.0 mL dry DCE. The product was isolated in 61% yield (44.2 mg) as white solid.

R*f*: 0.32 (petroleum ether).

HRMS (EI) (m/z): Calcd for $C_{26}H_{22}Si$ $[M]^+$: 362.1491, found:362.1481.

^{1}H NMR (400 MHz, $CDCl_3$) δ 8.21 (s, 1H), 7.88 – 7.81 (m, 3H), 7.70 (d, *J* = 8.4 Hz, 1H), 7.60 – 7.54 (m, 2H), 7.56 – 7.45 (m, 6H), 7.42 (dd, *J* = 8.4, 6.9 Hz, 2H), 7.37 – 7.29 (m, 1H), 5.29 (q, *J* = 7.2 Hz, 1H), 5.07 (d, *J* = 6.8 Hz, 1H), 5.05 (d, *J* = 6.8 Hz, 1H), 1.75 (d, *J* = 7.2 Hz, 3H).

^{13}C NMR (101 MHz, $CDCl_3$) δ 212.2, 140.9, 139.6, 136.9, 135.9, 134.2, 133.1, 131.2, 128.9, 128.9, 128.3, 127.9, 127.8, 127.4, 127.3, 127.1, 126.9, 126.2, 92.2, 83.6, 13.4.

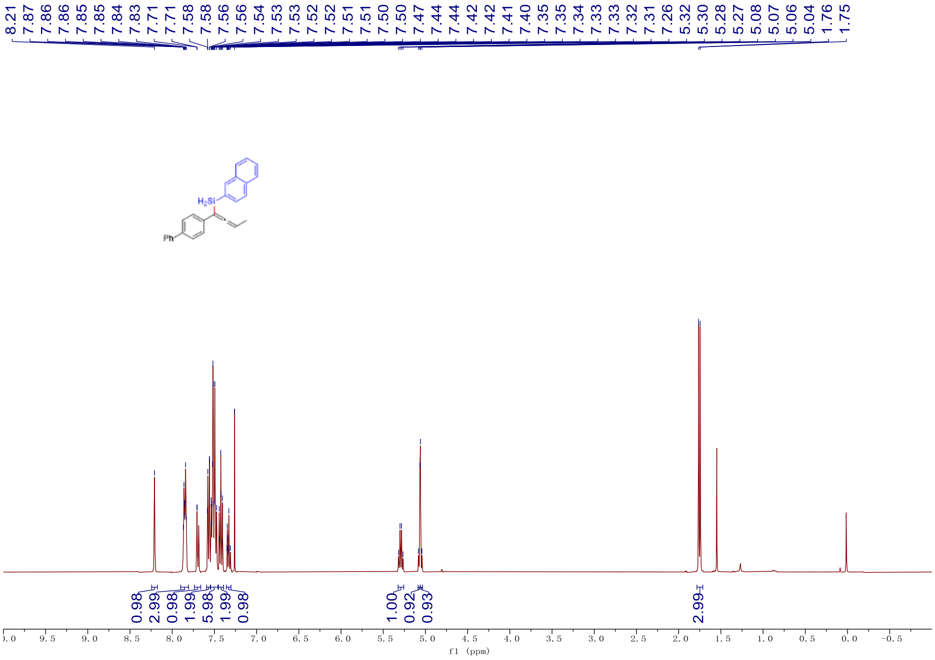

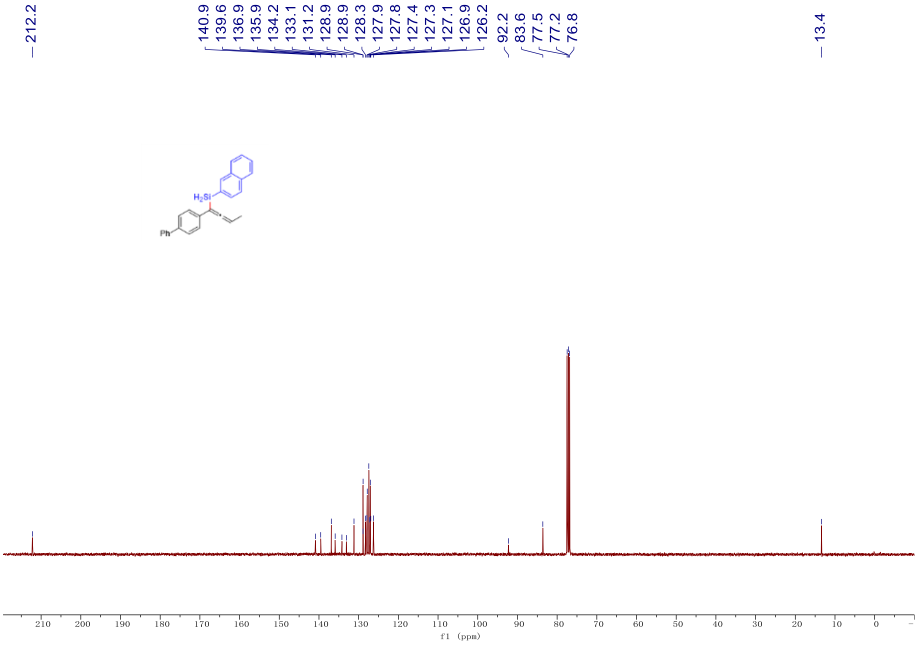

Methyl(phenyl)(1-phenylbuta-1,2-dien-1-yl)silane (3aj)

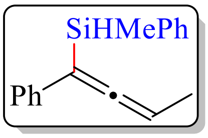

Prepared according to procedure 2 from **1a** (0.2 mmol, 25.6 mg) and **2j** (0.6 mmol, 73.3 mg). The reaction mixture was stirred at 40 °C for 1 h in 1.0 mL dry DCE. The product was isolated in 55% yield with 64:36 *dr* value (27.6 mg) as colorless oil.

R*f*: 0.52 (petroleum ether).

HRMS (ESI) (m/z): Calcd for $C_{17}H_{19}Si$ $[M+H]^+$: 251.1256, found: 251.1251.

^{1}H NMR (400 MHz, $CDCl_3$) δ 7.61 – 7.57 (m, 2H), 7.38 – 7.31 (m, 5H), 7.27 – 7.22 (m, 2H), 7.17 – 7.12 (m, 1H), 5.27 – 5.19 (m, 1H), 4.97 (p, *J* = 3.7 Hz, 1H), 1.73 (d, *J* = 7.2 Hz, 3H), 0.50 (s, 3H).

^{13}C NMR (101 MHz, $CDCl_3$) δ 211.0, 210.9, 137.3, 137.3, 135.6, 134.7, 134.7, 129.6, 129.6, 128.6, 128.1, 127.7, 127.7, 126.5, 95.9, 95.9, 83.4, 13.4, -4.4, -4.4.

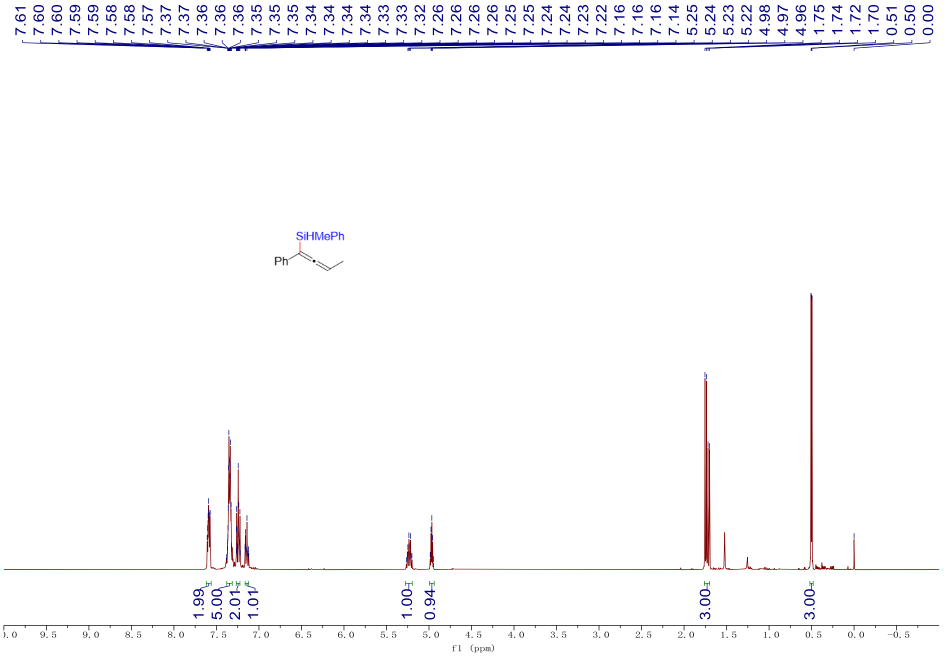

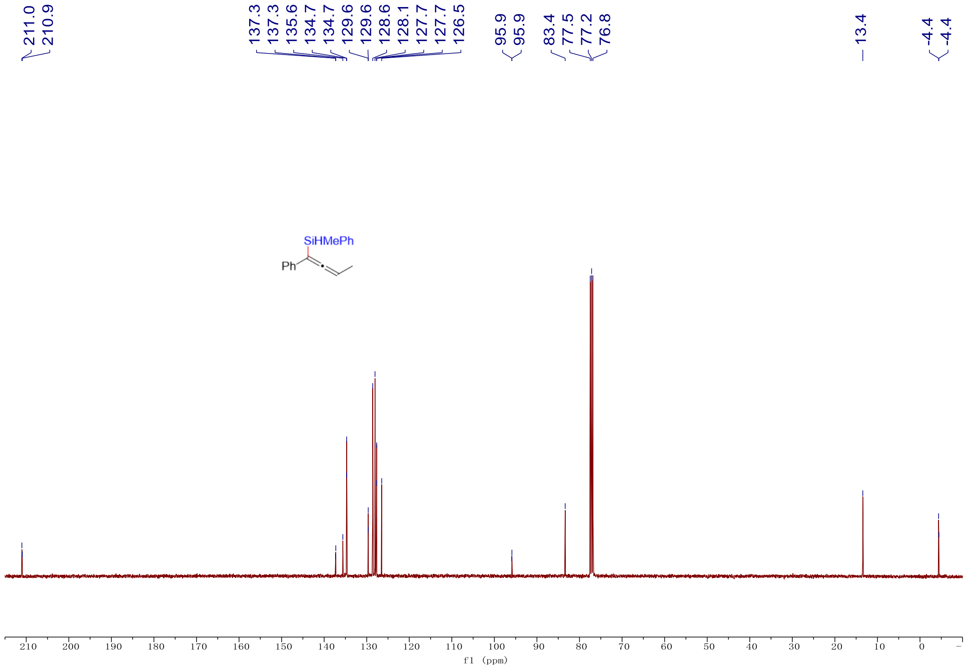

(*R*)-Phenyl(1-phenylbuta-1,2-dien-1-yl)silane (6aa)

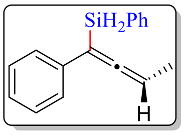

Prepared according to procedure 4 from **1a** (0.2 mmol, 25.6 mg) and **2a** (0.4 mmol, 43.3 mg). The product was isolated in 72% yield with 96% ee value (34.0 mg) as colorless oil.

R_f: 0.50 (petroleum ether).

HRMS (ESI) (m/z): Calcd for $C_{16}H_{17}Si$ $[M+H]^+$: 237.1100, found: 237.1110.

^{1}H NMR (400 MHz, $CDCl_3$) δ 7.66 – 7.62 (m, 2H), 7.42 – 7.34 (m, 5H), 7.31 – 7.26 (m, 2H), 7.21 – 7.16 (m, 1H), 5.24 (q, *J* = 7.2 Hz, 1H), 4.92 (d, *J* = 6.8 Hz, 1H), 4.90 (d, *J* = 6.8 Hz, 1H), 1.72 (d, *J* = 7.2 Hz, 3H).

^{13}C NMR (101 MHz, $CDCl_3$) δ 212.0, 136.9, 135.6, 131.6, 130.1, 128.7, 128.2, 127.5, 126.7, 92.6, 83.4, 13.3.

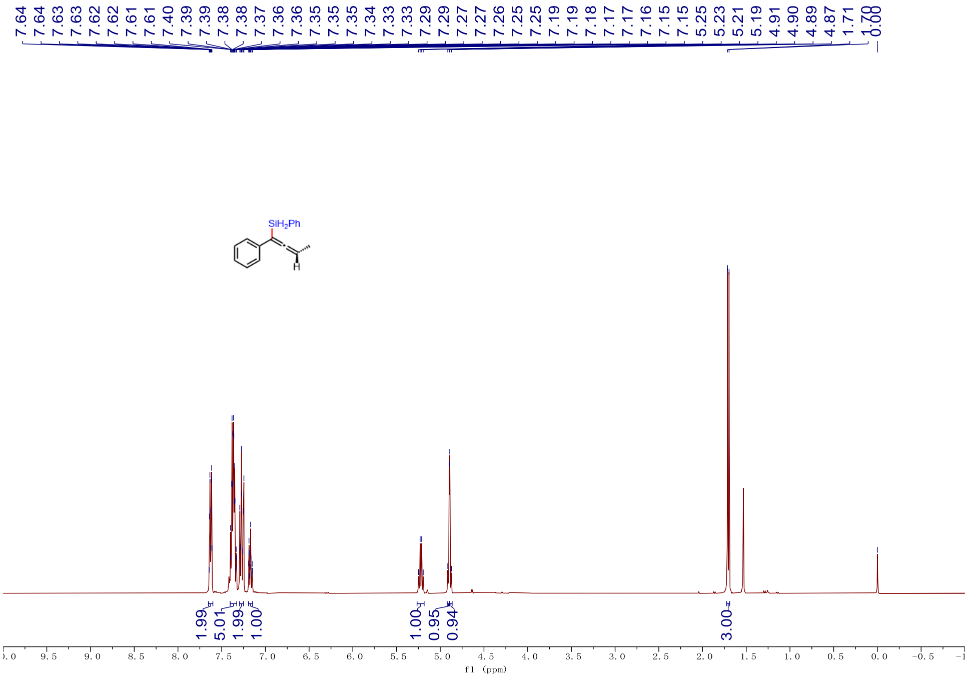

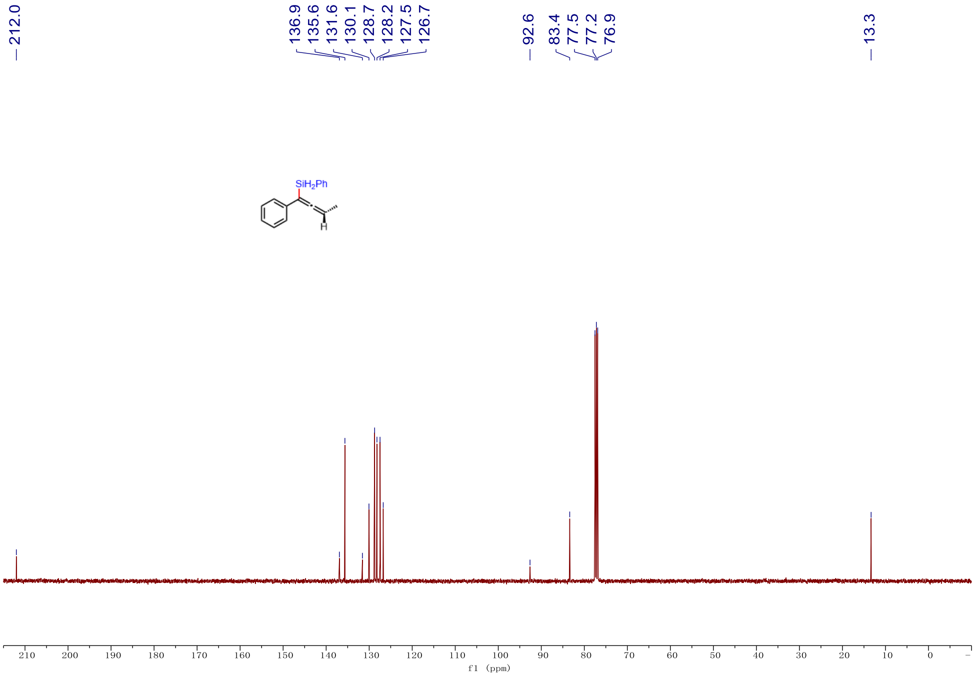

$[\alpha]_D^{20}$ +28.9° (c 1.75, ethyl acetate).

The enantiomeric excess of **6aa** was determined by chiral HPLC analysis on Chiralcel OJ-3 column.

Conditions: hexane : isopropanol = 98:2, flow rate = 0.5 mL/min, UV-Vis detection at λ = 254 nm.

t_{R1} = 13.7 min (major), t_{R2} = 14.9 min (minor)

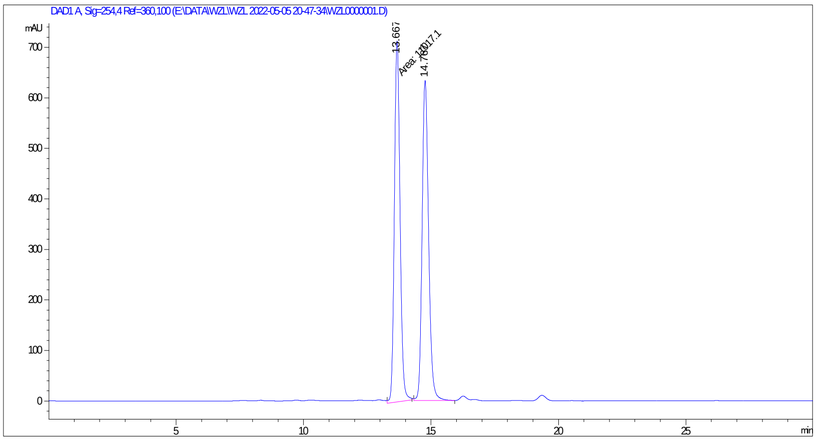

Signal 1: DAD1 A, Sig=254,4 Ref=360,100

Peak #	RetTime [min]	Type	Width [min]	Area [mAU*s]	Height [mAU]	Area %
1	13.667	MM	0.2570	1.10171e4	714.50537	49.5855
2	14.767	VB	0.2723	1.12013e4	633.37927	50.4145

Totals : 2.22185e4 1347.88464

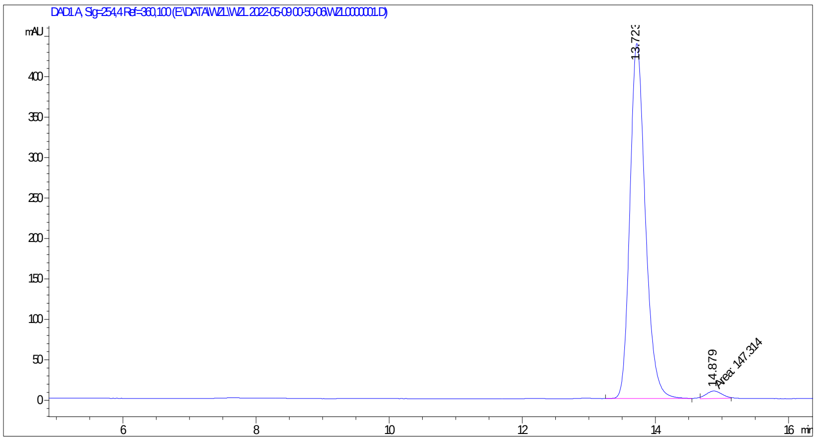

Signal 1: DAD1 A, Sig=254,4 Ref=360,100

Peak #	RetTime [min]	Type	Width [min]	Area [mAU*s]	Height [mAU]	Area %
1	13.723	BB	0.2459	7066.60254	438.78287	97.9579
2	14.879	MM	0.2686	147.31433	9.13920	2.0421

Totals : 7213.91687 447.92207

(*R*)-Phenyl(1-(*m*-tolyl)buta-1,2-dien-1-yl)silane (6ba)

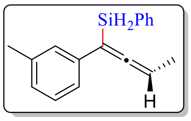

Prepared according to procedure 4 from **1b** (0.2 mmol, 28.4 mg) and **2a** (0.4 mmol, 43.3 mg). The product was isolated in 70% yield with 97% ee value (35.0 mg) as colorless oil.

R*f*: 0.52 (petroleum ether).

HRMS (ESI) (m/z): Calcd for $C_{17}H_{19}Si$ $[M+H]^+$: 251.1256, found: 251.1281.

^{1}H NMR (400 MHz, $CDCl_3$) δ 7.65 – 7.61 (m, 2H), 7.42 – 7.32 (m, 3H), 7.21 – 7.14 (m, 3H), 6.99 (q, *J* = 4.6, 4.0 Hz, 1H), 5.20 (q, *J* = 7.1 Hz, 1H), 4.90 (d, *J* = 6.6 Hz, 1H), 4.88 (d, *J* = 6.6 Hz, 1H), 2.31 (s, 3H), 1.70 (d, *J* = 7.1 Hz, 3H).

^{13}C NMR (101 MHz, $CDCl_3$) δ 211.9, 138.3, 136.7, 135.6, 131.6, 130.0, 128.6, 128.1, 128.1, 127.5, 124.5, 92.5, 83.2, 21.6, 13.3.

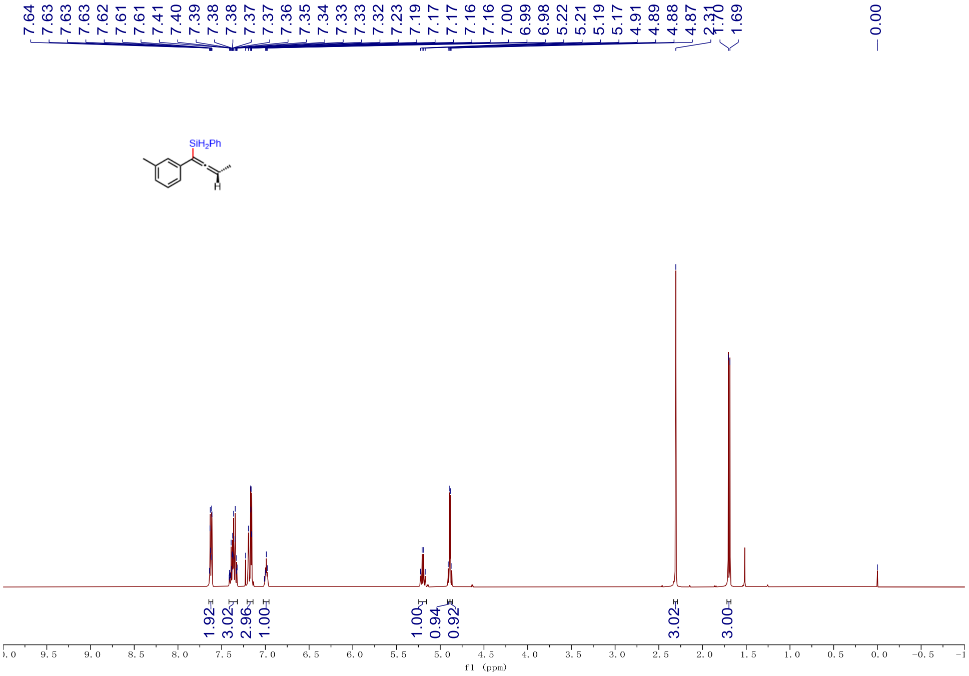

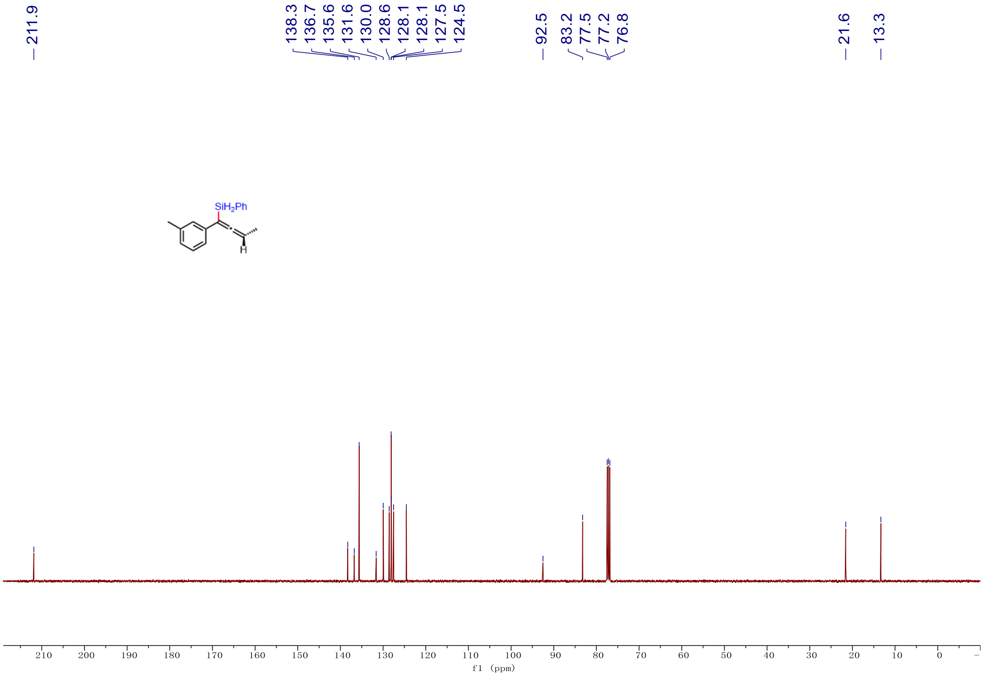

$[\alpha]_D^{20}$ +63.6° (*c* 1.75, ethyl acetate).
The enantiomeric excess of **6ba** was determined by chiral HPLC analysis on Chiralcel OJ-3 column.
Conditions: hexane : isopropanol = 100:1, flow rate = 0.5 mL/min, UV-Vis detection at λ = 254 nm.
t_{R1} = 13.6 min (major), t_{R2} = 14.6 min (minor).

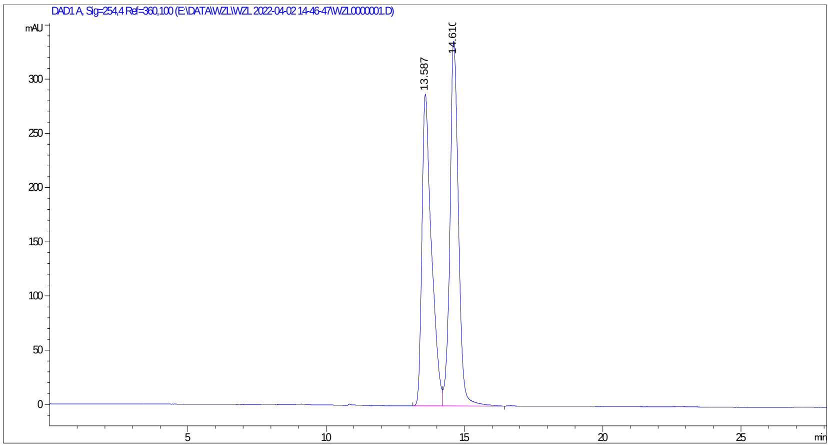

Signal 1: DAD1 A, Sig=254,4 Ref=360,100

Peak #	RetTime [min]	Type	Width [min]	Area [mAU*s]	Height [mAU]	Area %
1	13.587	BV	0.3429	6876.37939	287.61166	48.8602
2	14.610	VB	0.3257	7197.19482	336.33850	51.1398

Totals : 1.40736e4 623.95016

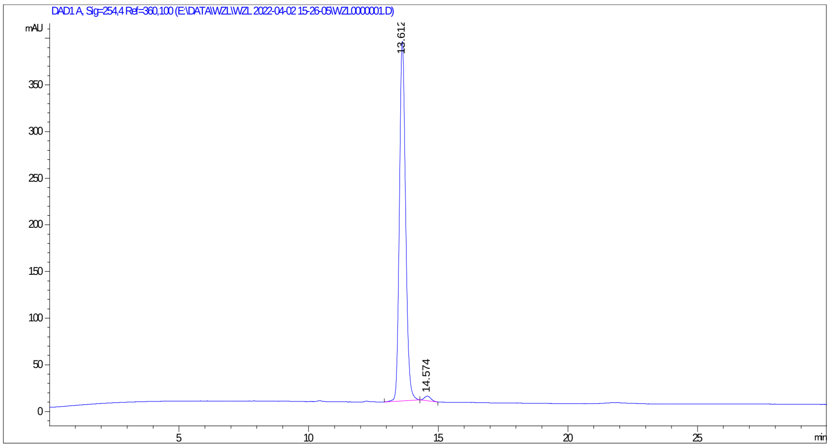

Signal 1: DAD1 A, Sig=254,4 Ref=360,100

Peak #	RetTime [min]	Type	Width [min]	Area [mAU*s]	Height [mAU]	Area %
1	13.612	BB	0.2574	6390.83984	385.48569	98.7099
2	14.574	BB	0.2684	83.52762	4.91169	1.2901

Totals : 6474.36746 390.39738

(*R*)-Phenyl(1-(*p*-tolyl)buta-1,2-dien-1-yl)silane (6ca)

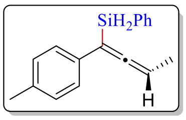

Prepared according to procedure 4 from **1c** (0.2 mmol, 28.4 mg) and **2a** (0.4 mmol, 43.3 mg). The product was isolated in 85% yield with 93% ee value (42.7 mg) as colorless oil.

R*f*: 0.49 (petroleum ether).

HRMS (ESI) (m/z): Calcd for $C_{17}H_{19}Si$ $[M+H]^+$: 251.1256, found: 251.1273.

^{1}H NMR (400 MHz, $CDCl_3$) δ 7.64 – 7.60 (m, 2H), 7.42 – 7.32 (m, 3H), 7.29 – 7.24 (m, 2H), 7.11 – 7.06 (m, 2H), 5.20 (q, *J* = 7.2 Hz, 1H), 4.89 (d, *J* = 6.8 Hz, 1H), 4.87 (d, *J* = 6.8 Hz, 1H), 2.30 (s, 3H), 1.69 (d, *J* = 7.2 Hz, 3H).

^{13}C NMR (101 MHz, $CDCl_3$) δ 211.6, 136.4, 135.6, 133.8, 131.6, 130.0, 129.4, 128.1, 127.3, 92.2, 83.2, 21.2, 13.4.

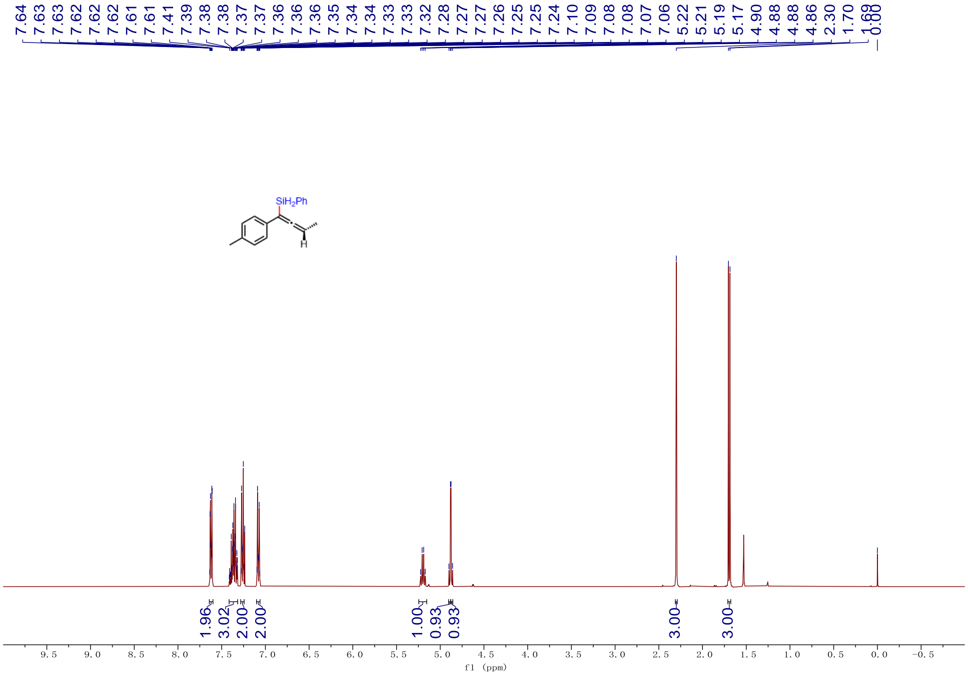

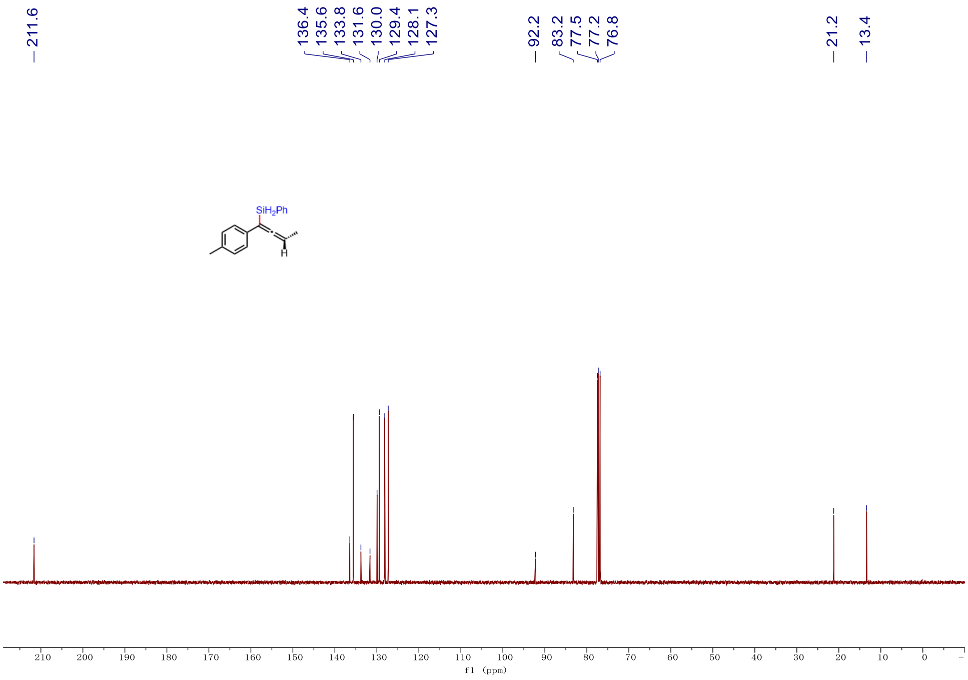

$[\alpha]_D^{20}$ +44.4° (*c* 2.1, ethyl acetate).

The enantiomeric excess of **6ca** was determined by chiral HPLC analysis on Chiralcel OJ-3 column.

Conditions: hexane : isopropanol = 99:1, flow rate = 0.5 mL/min, UV-Vis detection at λ = 254 nm.

t_{R1} = 14.0 min (minor), t_{R2} = 14.9 min (major)

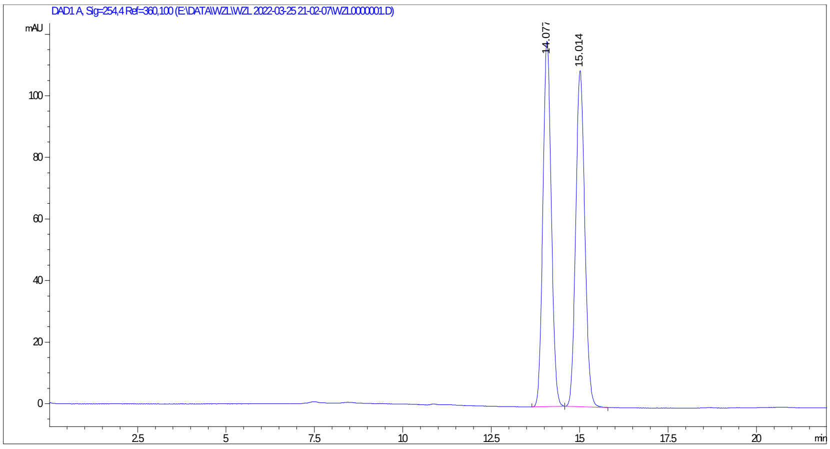

Signal 1: DAD1 A, Sig=254,4 Ref=360,100

Peak #	RetTime [min]	Type	Width [min]	Area [mAU*s]	Height [mAU]	Area %
1	14.077	BB	0.2564	1956.84131	118.67892	50.1198
2	15.014	BB	0.2781	1947.48865	109.15919	49.8802
Totals :				3904.32996	227.83811	

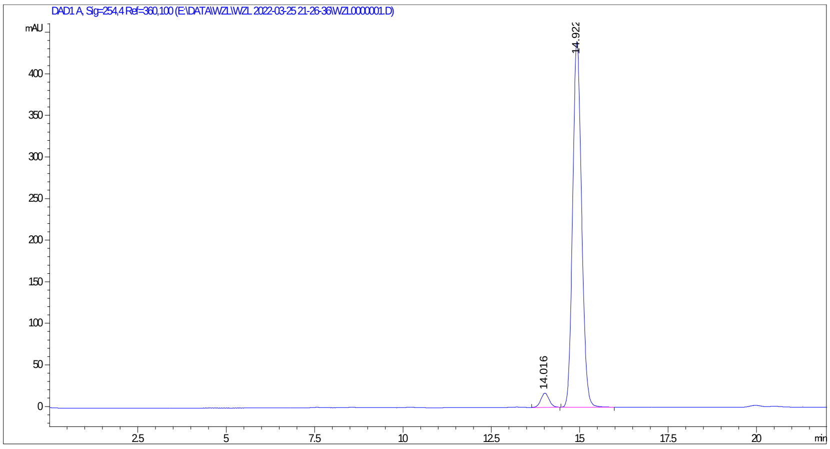

Signal 1: DAD1 A, Sig=254,4 Ref=360,100

Peak #	RetTime [min]	Type	Width [min]	Area [mAU*s]	Height [mAU]	Area %
1	14.016	BB	0.2563	276.44012	16.94394	3.4526
2	14.922	BB	0.2731	7730.24609	439.74554	96.5474
Totals :				8006.68622	456.68948	

(*R*)-(1-(4-(*Tert*-butyl)phenyl)buta-1,2-dien-1-yl)(phenyl)silane (6da)

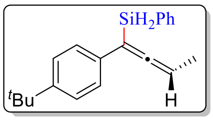

Prepared according to procedure 4 from **1d** (0.2 mmol, 36.9 mg) and **2a** (0.4 mmol, 43.3 mg). The product was isolated in 70% yield with 98% ee value (41.0 mg) as colorless oil.

R*f*: 0.46 (petroleum ether).

HRMS (ESI) (m/z): Calcd for $C_{20}H_{25}Si$ $[M+H]^+$: 293.1726, found: 293.1758.

^{1}H NMR (400 MHz, $CDCl_3$) δ 7.66 – 7.62 (m, 2H), 7.43 – 7.33 (m, 3H), 7.31 (s, 4H), 5.21 (q, *J* = 7.2 Hz, 1H), 4.89 (d, *J* = 6.8 Hz, 1H), 4.87 (d, *J* = 6.8 Hz, 1H), 1.70 (d, *J* = 7.1 Hz, 3H), 1.29 (s, 9H).

^{13}C NMR (101 MHz, $CDCl_3$) δ 211.8, 149.7, 135.6, 133.8, 131.7, 130.0, 128.1, 127.1, 125.7, 92.1, 83.2, 34.6, 31.4, 13.4.

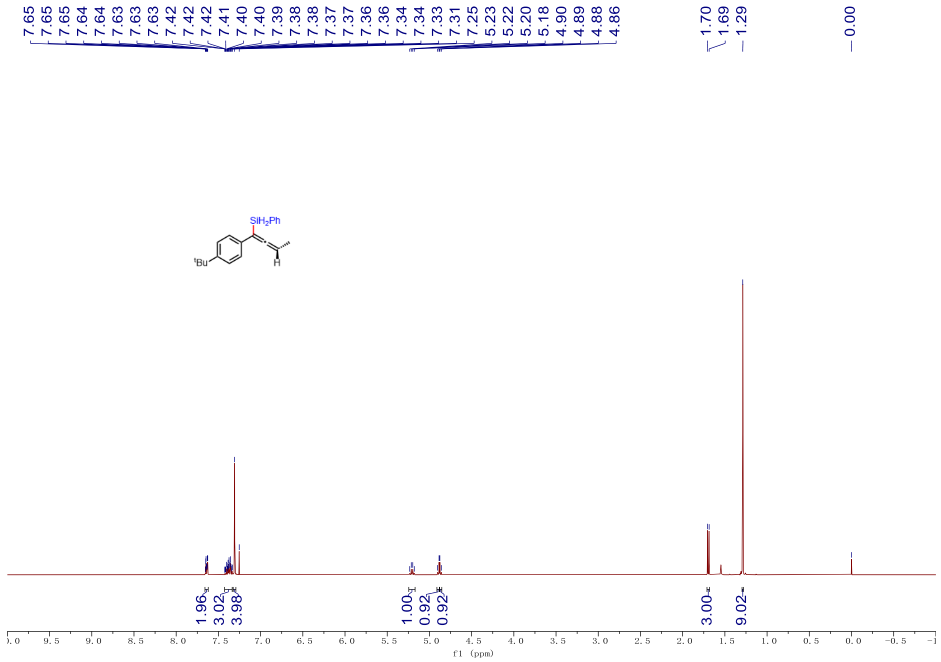

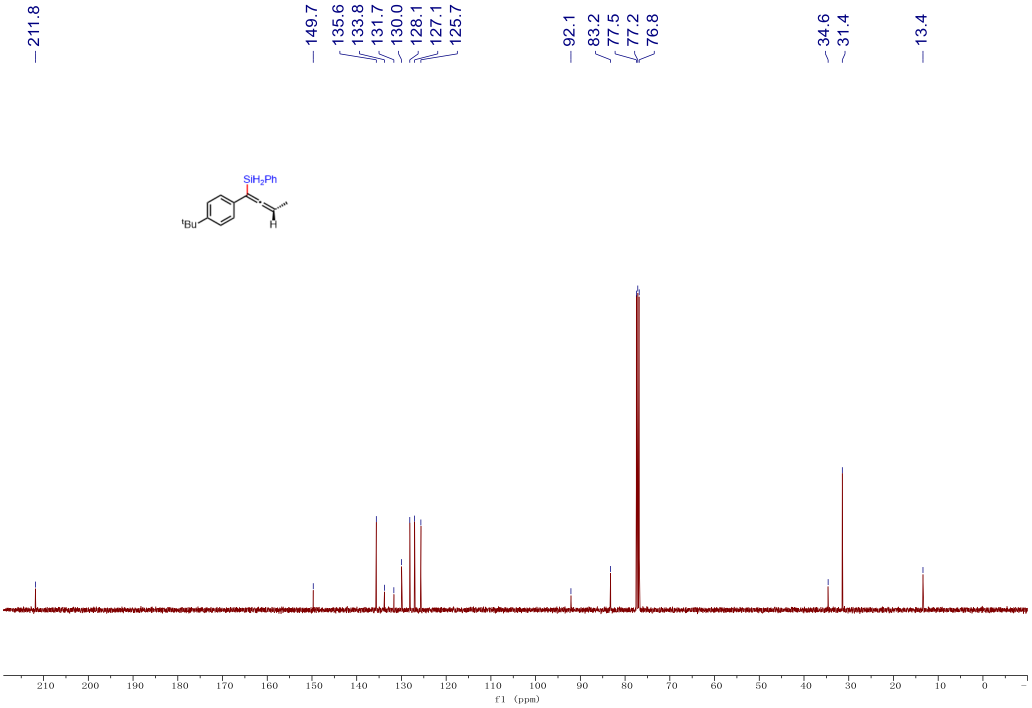

$[\alpha]_D^{20}$ +91° (c 2.0, ethyl acetate).

The enantiomeric excess of **6da** was determined by chiral HPLC analysis on Chiralcel OJ-3 column.

Conditions: hexane : isopropanol = 200:1, flow rate = 0.5 mL/min, UV-Vis detection at λ = 254 nm.

t_{R1} = 15.7 min (minor), t_{R2} = 17.4 min (major)

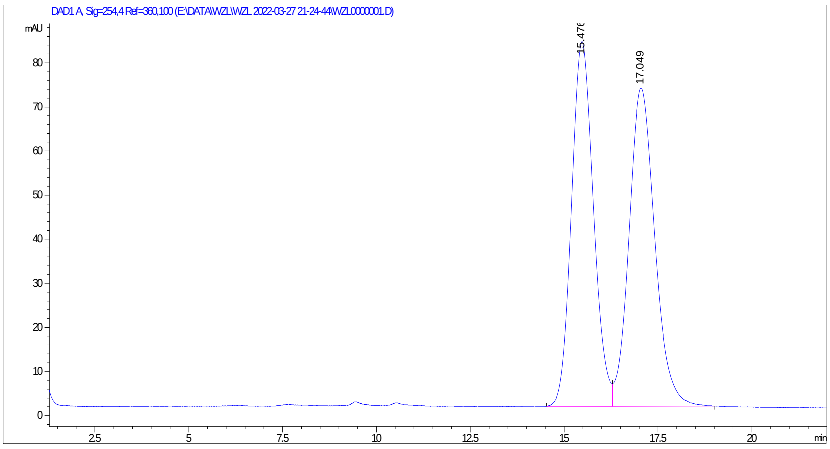

Signal 1: DAD1 A, Sig=254,4 Ref=360,100

Peak #	RetTime [min]	Type	Width [min]	Area [mAU*s]	Height [mAU]	Area %
1	15.476	BV	0.6314	3370.64966	82.71425	49.4376
2	17.049	VB	0.7293	3447.34277	72.16938	50.5624
Totals :				6817.99243	154.88363	

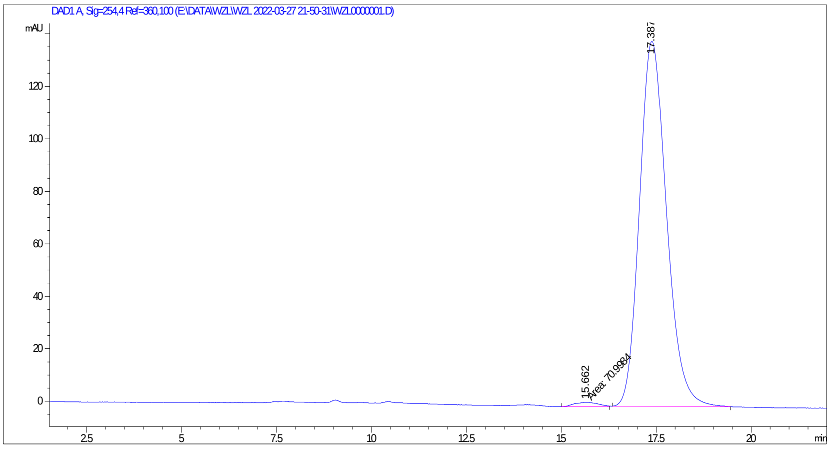

Signal 1: DAD1 A, Sig=254,4 Ref=360,100

Peak #	RetTime [min]	Type	Width [min]	Area [mAU*s]	Height [mAU]	Area %
1	15.662	MM	0.7365	70.99844	1.60659	1.0247
2	17.387	BB	0.7615	6857.96631	139.03304	98.9753
Totals :				6928.96474	140.63963	

(*R*)-(1-([1,1'-Biphenyl]-4-yl)buta-1,2-dien-1-yl)(phenyl)silane (6ea)

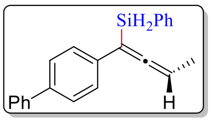

Prepared according to procedure 4 from **1e** (0.2 mmol, 40.9 mg) and **2a** (0.6 mmol, 64.9 mg). The reaction mixture was stirred at -50 °C for 24 h in 2.0 mL dry DCE. The product was isolated in 68% yield with 93% ee value (42.6 mg) as colorless oil.

R*f*: 0.40 (petroleum ether).

HRMS (ESI) (m/z): Calcd for $C_{22}H_{21}Si$ $[M+H]^+$: 313.1413, found: 313.1443.

^{1}H NMR (400 MHz, $CDCl_3$) δ 7.65 (dd, *J* = 7.6, 1.5 Hz, 2H), 7.58 – 7.49 (m, 4H), 7.46 – 7.28 (m, 8H), 5.26 (q, *J* = 7.1 Hz, 1H), 4.94 (d, *J* = 6.8 Hz, 1H), 4.91 (d, *J* = 6.8 Hz, 1H), 1.73 (d, *J* = 7.1 Hz, 3H).

^{13}C NMR (101 MHz, $CDCl_3$) δ 212.1, 140.9, 139.5, 135.9, 135.6, 131.5, 130.1, 128.9, 128.2, 127.8, 127.4, 127.3, 127.1, 92.2, 83.5, 13.3.

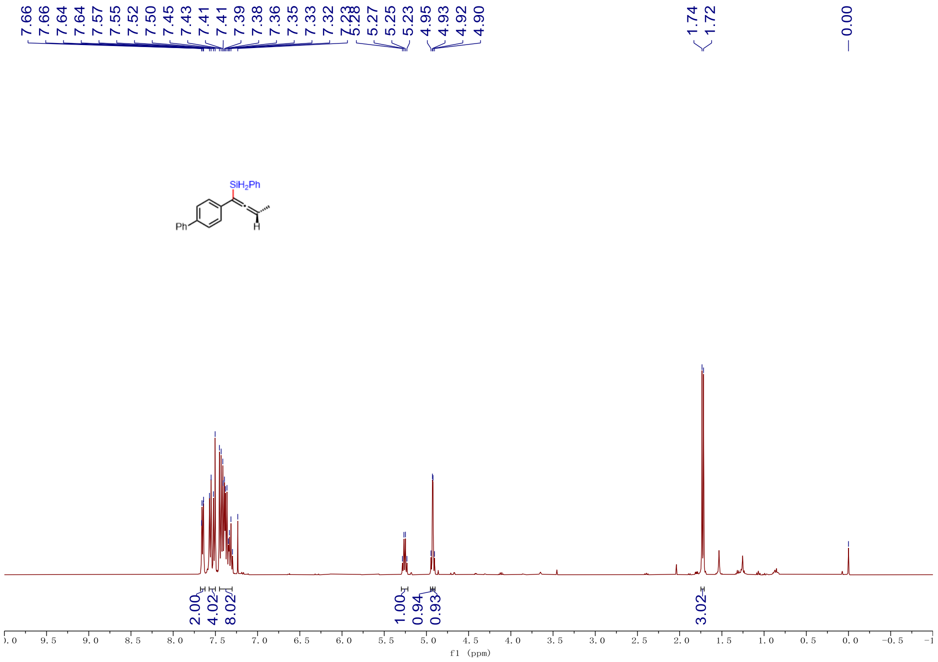

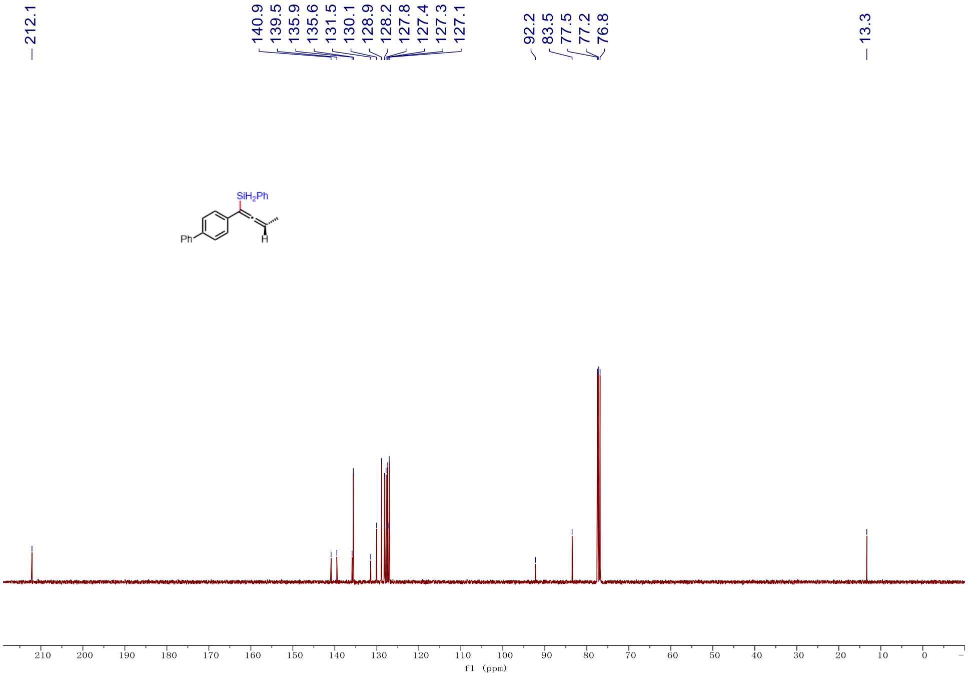

$[\alpha]_D^{20}$ +82.9° (*c* 1.5, ethyl acetate).

The enantiomeric excess of **6ea** was determined by chiral HPLC analysis on Chiralcel OJ-3 column.

Conditions: hexane : isopropanol = 99:1, flow rate = 0.5 mL/min, UV-Vis detection at λ = 220 nm.

t_{R1} = 39.6 min (minor), t_{R2} = 44.8 min (major).

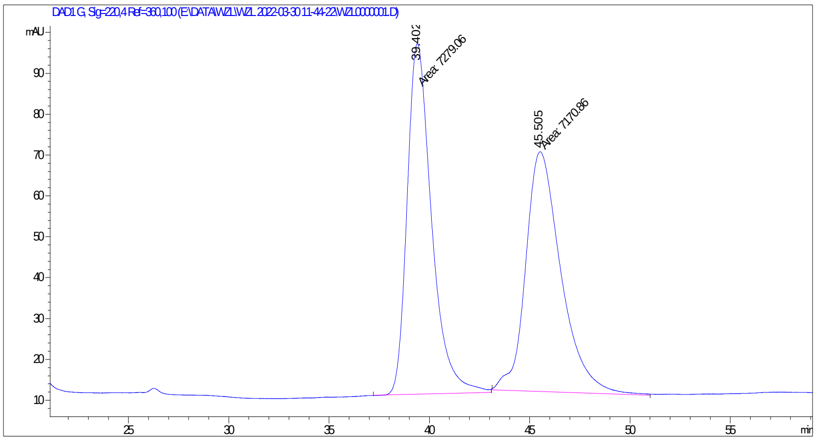

Signal 7: DAD1 G, Sig=220,4 Ref=360,100

Peak #	RetTime [min]	Type	Width [min]	Area [mAU*s]	Height [mAU]	Area %
1	39.402	MM	1.4140	7279.06104	85.79681	50.3744
2	45.505	MM	2.0395	7170.85986	58.59877	49.6256

Totals : 1.44499e4 144.39559

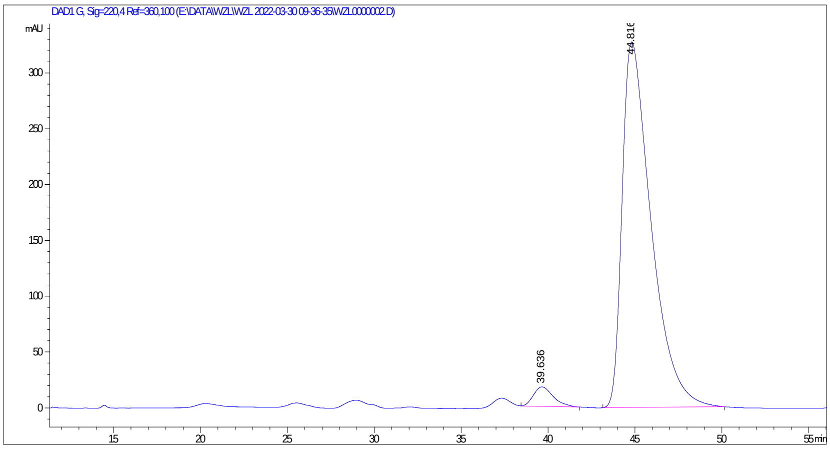

Signal 7: DAD1 G, Sig=220,4 Ref=360,100

Peak #	RetTime [min]	Type	Width [min]	Area [mAU*s]	Height [mAU]	Area %
1	39.636	BB	0.9364	1380.43750	17.42213	3.5119
2	44.816	BB	1.6412	3.79269e4	327.46713	96.4881

Totals : 3.93074e4 344.88927

(*R*)-(1-(Naphthalen-2-yl)buta-1,2-dien-1-yl)(phenyl)silane (6fa)

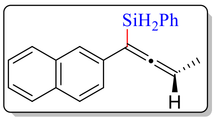

Prepared according to procedure 4 from **1f** (0.2 mmol, 35.6 mg) and **2a** (0.6 mmol, 64.9 mg). The reaction mixture was stirred at -50 °C for 24 h in 2.0 mL dry DCE. The product was isolated in 53% yield with 90% ee value (30.5 mg) as colorless oil.

R*f*: 0.40 (petroleum ether).

HRMS (ESI) (m/z): Calcd for $C_{20}H_{19}Si$ $[M+H]^+$: 287.1256, found: 287.1260.

^{1}H NMR (400 MHz, $CDCl_3$) δ 7.79 – 7.71 (m, 4H), 7.69 – 7.64 (m, 2H), 7.58 (dd, *J* = 8.4, 1.9 Hz, 1H), 7.45 – 7.33 (m, 5H), 5.29 (q, *J* = 7.1 Hz, 1H), 5.00 (d, *J* = 6.8 Hz, 1H), 4.98 (d, *J* = 6.8 Hz, 1H), 1.75 (d, *J* = 7.1 Hz, 3H).

^{13}C NMR (101 MHz, $CDCl_3$) δ 212.5, 135.6, 134.3, 133.7, 132.4, 131.5, 130.1, 128.2, 128.2, 128.0, 127.7, 126.2, 125.9, 125.9, 125.8, 92.8, 83.7, 13.4.

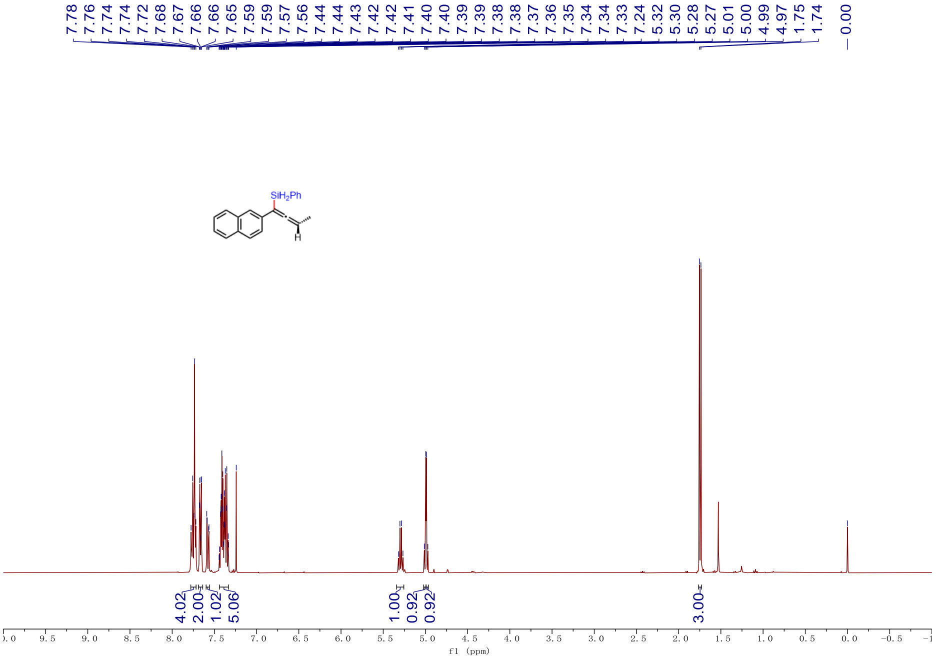

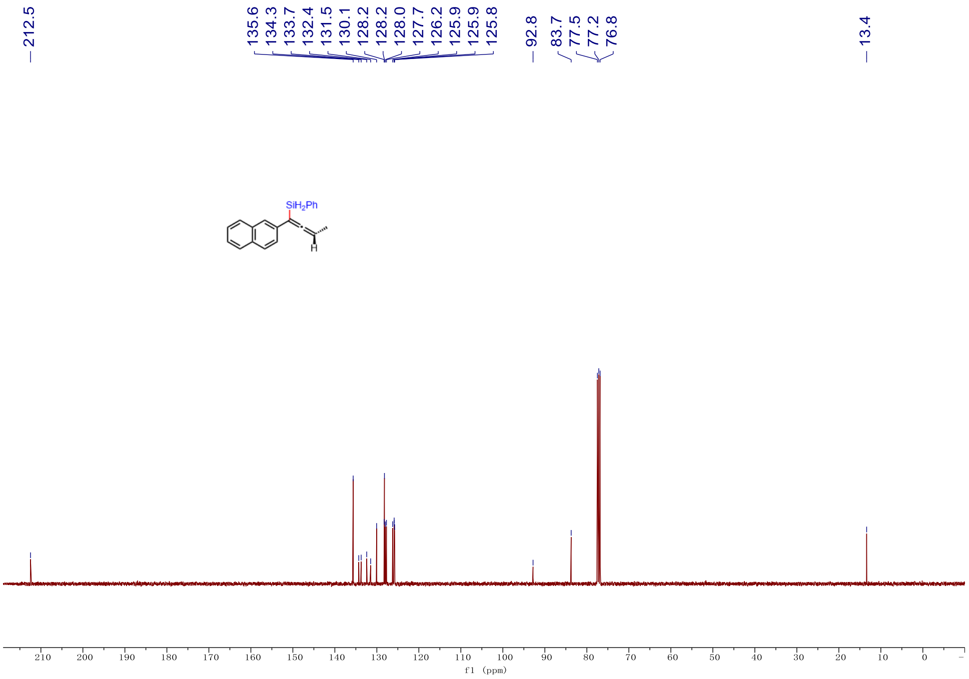

$[\alpha]_D^{20}$ +64° (*c* 1.5, ethyl acetate).

The enantiomeric excess of **6fa** was determined by chiral HPLC analysis on Chiralcel OJ-3 column.

Conditions: hexane : isopropanol = 98:2, flow rate = 0.5 mL/min, UV-Vis detection at λ = 254 nm.

t_{R1} = 18.1 min (minor), t_{R2} = 20.7 min (major).

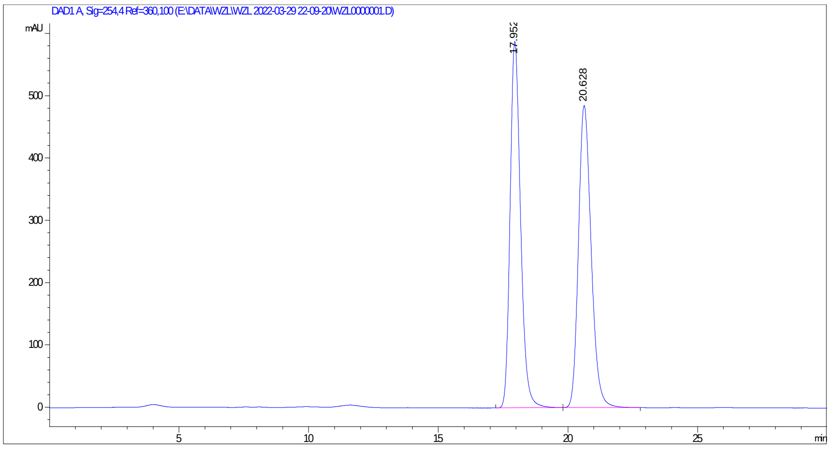

Signal 1: DAD1 A, Sig=254,4 Ref=360,100

Peak #	RetTime [min]	Type	Width [min]	Area [mAU*s]	Height [mAU]	Area %
1	17.952	BB	0.4310	1.64819e4	587.99988	49.9963
2	20.628	BB	0.5245	1.64844e4	485.03809	50.0037

Totals : 3.29662e4 1073.03796

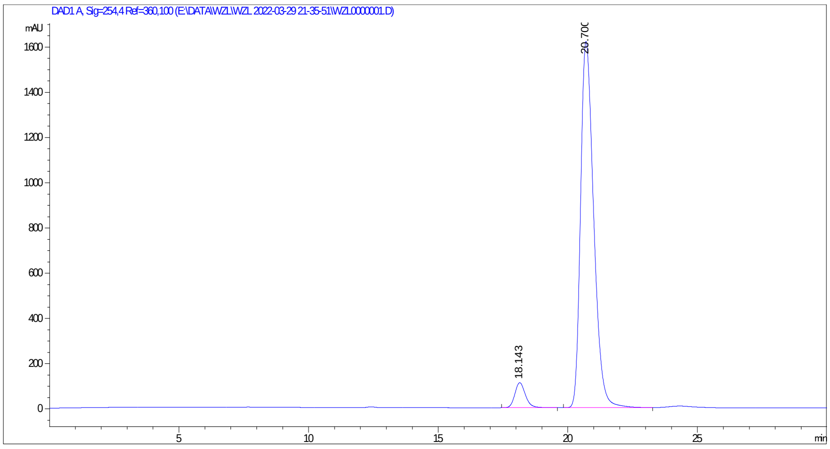

Signal 1: DAD1 A, Sig=254,4 Ref=360,100

Peak #	RetTime [min]	Type	Width [min]	Area [mAU*s]	Height [mAU]	Area %
1	18.143	BB	0.4364	3133.11694	110.63470	5.2348
2	20.700	BB	0.5426	5.67181e4	1619.27747	94.7652

Totals : 5.98513e4 1729.91217

(*R*)-(1-(4-Fluorophenyl)buta-1,2-dien-1-yl)(phenyl)silane (6ga)

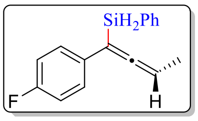

Prepared according to procedure 4 from **1g** (0.2 mmol, 29.2 mg) and **2a** (0.4 mmol, 43.3 mg). The product was isolated in 62% yield with 97% ee value (31.6 mg) as colorless oil.

R_f: 0.48 (petroleum ether).

HRMS (ESI) (m/z): Calcd for $C_{16}H_{16}FSi$ $[M+H]^+$: 255.1005, found: 255.1026.

^{1}H NMR (400 MHz, $CDCl_3$) δ 7.65 – 7.60 (m, 2H), 7.44 – 7.30 (m, 5H), 7.00 – 6.94 (m, 2H), 5.23 (q, *J* = 7.2 Hz, 1H), 4.89 (d, *J* = 6.8 Hz, 1H), 4.87 (d, *J* = 6.8 Hz, 1H), 1.71 (d, *J* = 7.0 Hz, 3H).

^{13}C NMR (101 MHz, $CDCl_3$) δ 211.8, 161.8 (d, *J* = 246.0 Hz), 135.6, 132.8 (d, *J* = 3.3 Hz), 131.3, 130.2, 128.9 (d, *J* = 8.0 Hz), 128.3, 115.6 (d, *J* = 21.6 Hz), 91.7, 83.6, 13.4.

^{19}F NMR (376 MHz, $CDCl_3$) δ -116.1

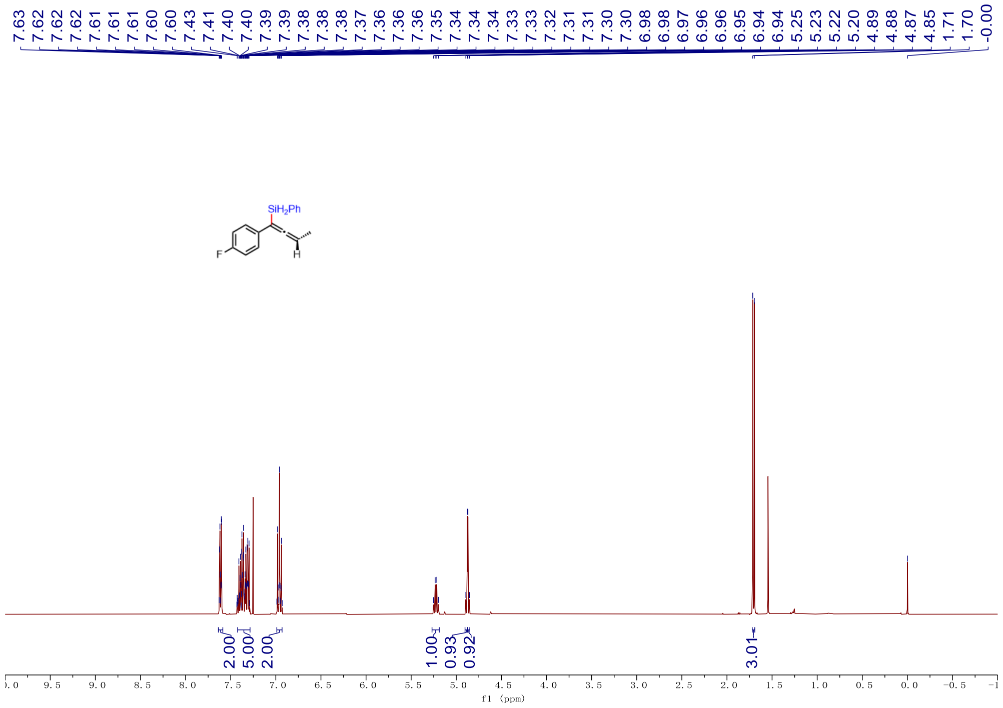

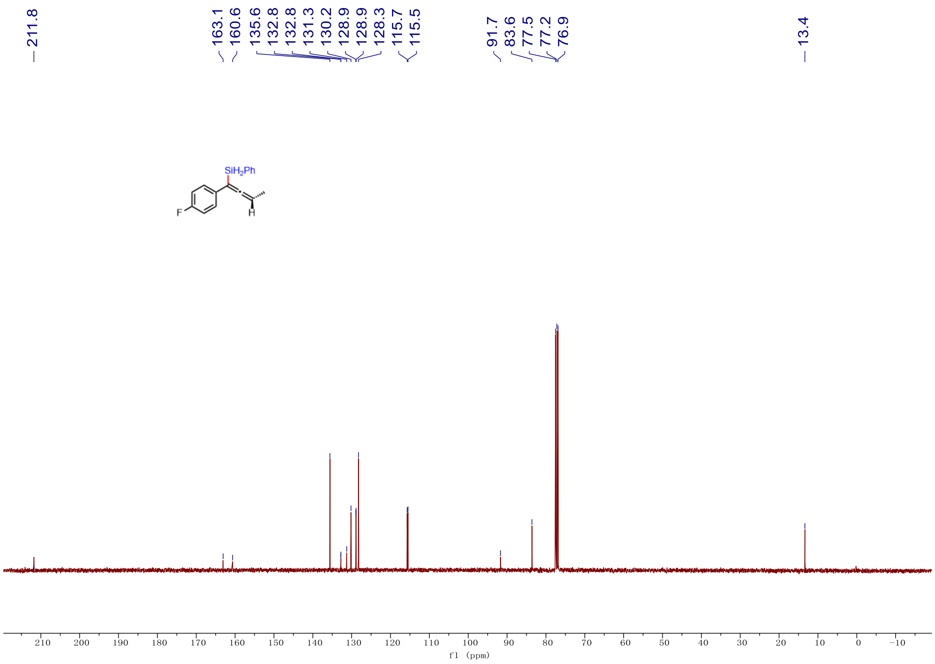
211.8
163.1
160.6
135.6
132.8
132.8
131.3
130.2
128.9
128.9
128.3
115.7
115.5
91.7
83.6
77.5
77.2
76.9
13.4
SiH2Ph
F
H
f1 (ppm)

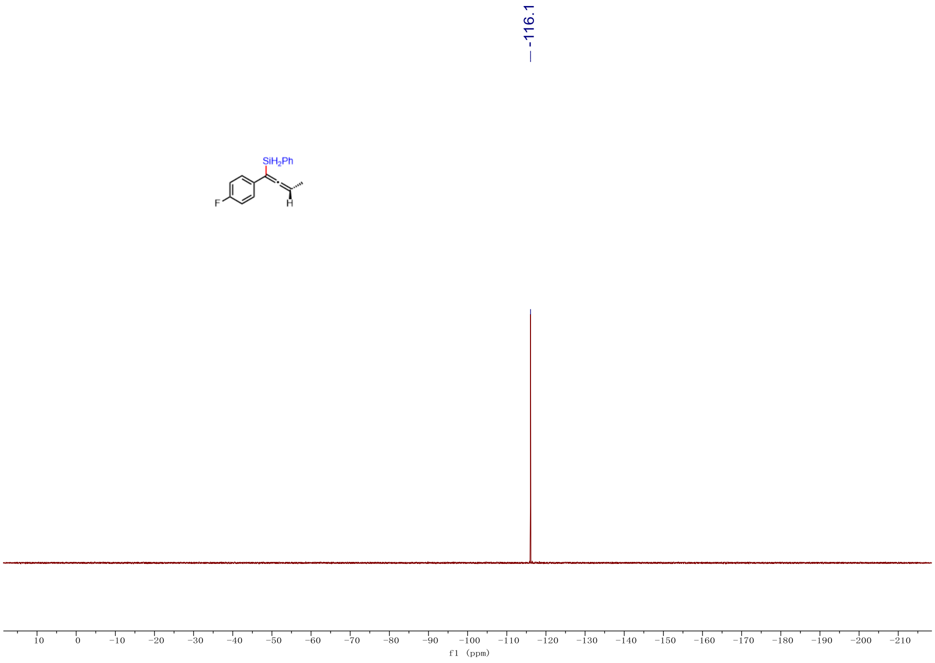
-116.1
SiH2Ph
F
H
f1 (ppm)

$[\alpha]_D^{20}$ +49.3° (c 1.55, ethyl acetate).
The enantiomeric excess of **6ga** was determined by chiral HPLC analysis on Chiralcel OJ-3 column.
Conditions: hexane : isopropanol = 98:2, flow rate = 0.5 mL/min, UV-Vis detection at λ = 254 nm.
t_{R1} = 12.4 min (minor), t_{R2} = 13.3 min (major)

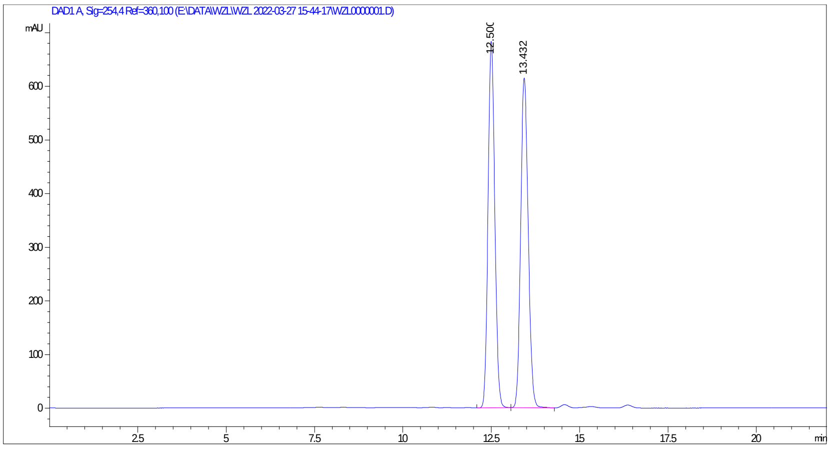

Signal 1: DAD1 A, Sig=254,4 Ref=360,100

Peak #	RetTime [min]	Type	Width [min]	Area [mAU*s]	Height [mAU]	Area %
1	12.500	BB	0.2068	9061.07031	683.05792	49.9754
2	13.432	BB	0.2302	9070.00195	614.58234	50.0246

Totals : 1.81311e4 1297.64026

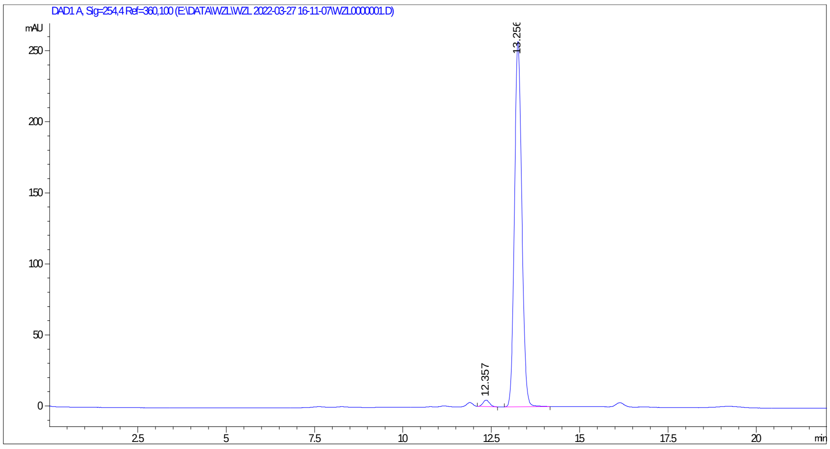

Signal 1: DAD1 A, Sig=254,4 Ref=360,100

Peak #	RetTime [min]	Type	Width [min]	Area [mAU*s]	Height [mAU]	Area %
1	12.357	BB	0.2034	58.58433	4.57416	1.5494
2	13.256	BB	0.2249	3722.63208	257.08878	98.4506

Totals : 3781.21641 261.66294

(*R*)-(1-(4-Chlorophenyl)buta-1,2-dien-1-yl)(phenyl)silane (6ha)

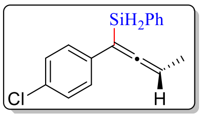

Prepared according to procedure 4 from **1h** (0.2 mmol, 32.5 mg) and **2a** (0.6 mmol, 64.9 mg). The reaction mixture was stirred at -50 °C for 24 h in 2.0 mL dry DCE. The product was isolated in 62% yield with 94% ee value (33.7 mg) as colorless oil.

R$_f$: 0.50 (petroleum ether).

HRMS (ESI) (m/z): Calcd for $C_{16}H_{15}ClNaSi$ $[M+Na]^+$: 293.0529, found: 293.0553.

^{1}H NMR (400 MHz, $CDCl_3$) δ 7.63 – 7.59 (m, 2H), 7.44 – 7.33 (m, 3H), 7.30 – 7.26 (m, 2H), 7.25 – 7.20 (m, 2H), 5.24 (q, *J* = 7.1 Hz, 1H), 4.88 (d, *J* = 6.8 Hz, 1H), 4.86 (d, *J* = 6.8 Hz, 1H), 1.71 (d, *J* = 7.1 Hz, 3H).

^{13}C NMR (101 MHz, $CDCl_3$) δ 212.0, 135.5, 135.4, 132.4, 131.1, 130.2, 128.8, 128.6, 128.2, 91.8, 83.7, 13.2.

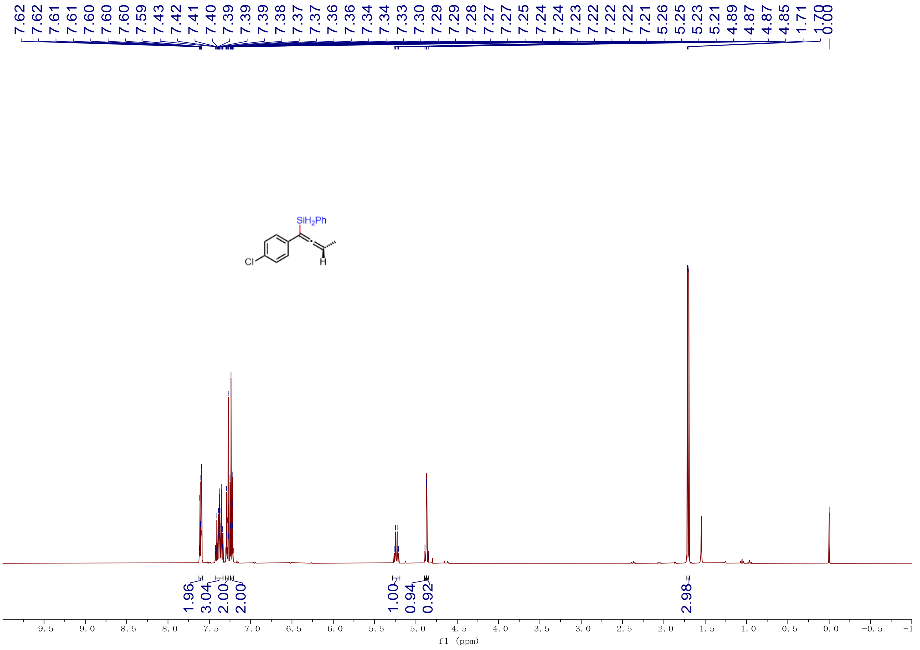

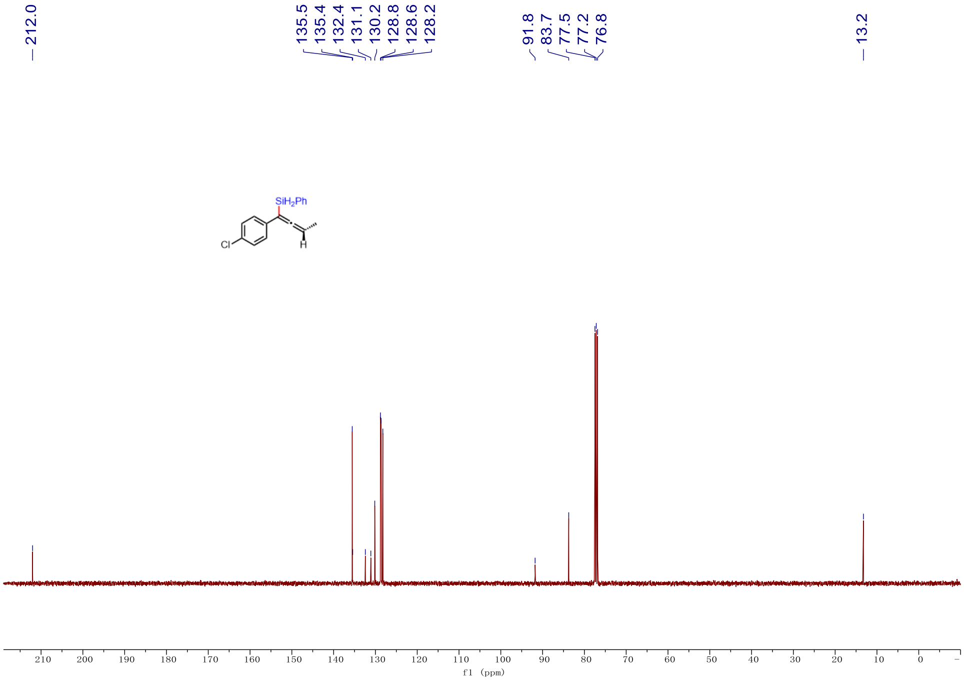

$[\alpha]_D^{20}$ +53.9° (*c* 1.65, ethyl acetate).

The enantiomeric excess of **6ha** was determined by chiral HPLC analysis on Chiralcel OJ-3 column.

Conditions: hexane : isopropanol = 99:1, flow rate = 0.5 mL/min, UV-Vis detection at λ = 270 nm.

t_{R1} = 11.8 min (minor), t_{R2} = 12.7 min (major).

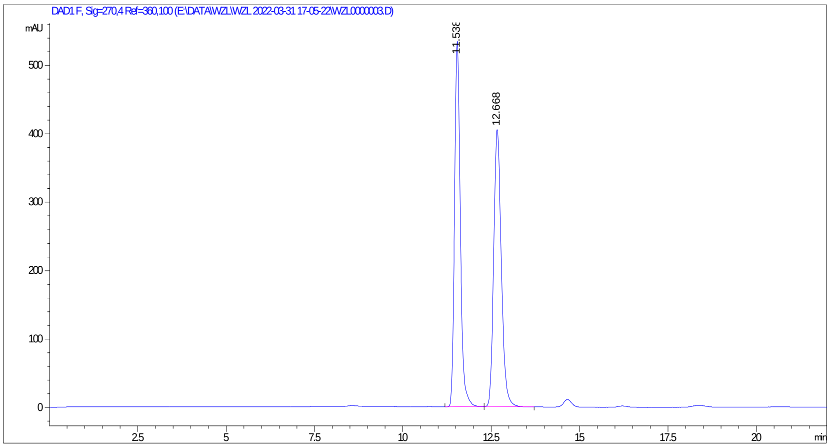

Signal 6: DAD1 F, Sig=270,4 Ref=360,100

Peak #	RetTime [min]	Type	Width [min]	Area [mAU*s]	Height [mAU]	Area %
1	11.538	BB	0.1749	6081.52246	534.02417	50.0782
2	12.668	BB	0.2306	6062.52441	405.18558	49.9218
Totals :				1.21440e4	939.20975	

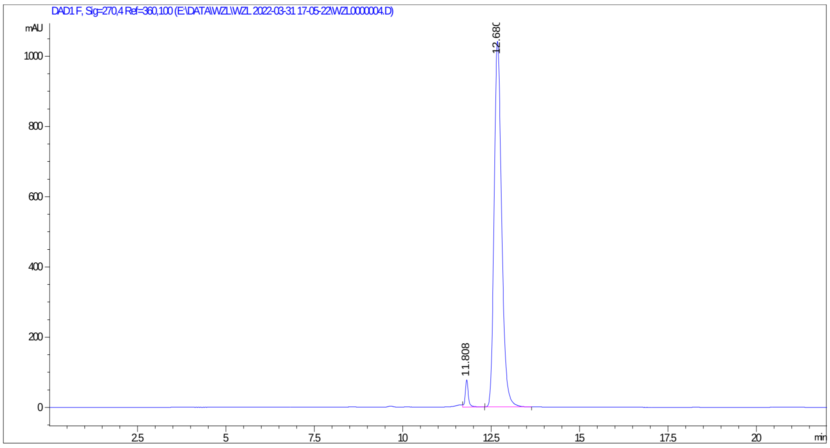

Signal 6: DAD1 F, Sig=270,4 Ref=360,100

Peak #	RetTime [min]	Type	Width [min]	Area [mAU*s]	Height [mAU]	Area %
1	11.808	VB	0.0898	462.21323	77.26671	2.9189
2	12.680	BB	0.2283	1.53728e4	1040.80212	97.0811
Totals :				1.58350e4	1118.06883	

(*R*)-(1-(4-Bromophenyl)buta-1,2-dien-1-yl)(phenyl)silane (6ia)

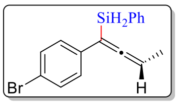

Prepared according to procedure 4 from **1i** (0.2 mmol, 41.4 mg) and **2a** (0.6 mmol, 64.9 mg). The reaction mixture was stirred at -50 °C for 24 h in 2.0 mL dry DCE. The product was isolated in 59% yield with 91% ee value (37.2 mg) as colorless oil.

R*f*: 0.53 (petroleum ether).

HRMS (EI) (m/z): Calcd for $C_{16}H_{15}BrSi$ $[M]^+$: 314.0126, found: 314.0128.

^{1}H NMR (400 MHz, $CDCl_3$) δ 7.61 (dt, *J* = 6.4, 1.6 Hz, 2H), 7.42 – 7.34 (m, 5H), 7.23 (tt, *J* = 8.4, 2.0 Hz, 2H), 5.24 (q, *J* = 7.2 Hz, 1H), 4.89 (d, *J* = 6.8 Hz, 1H), 4.87 (d, *J* = 6.8 Hz, 1H), 1.72 (d, *J* = 7.2 Hz, 3H).

^{13}C NMR (101 MHz, $CDCl_3$) δ 212.0, 135.9, 135.5, 131.7, 131.1, 130.2, 129.0, 128.2, 120.5, 91.9, 83.8, 13.2.

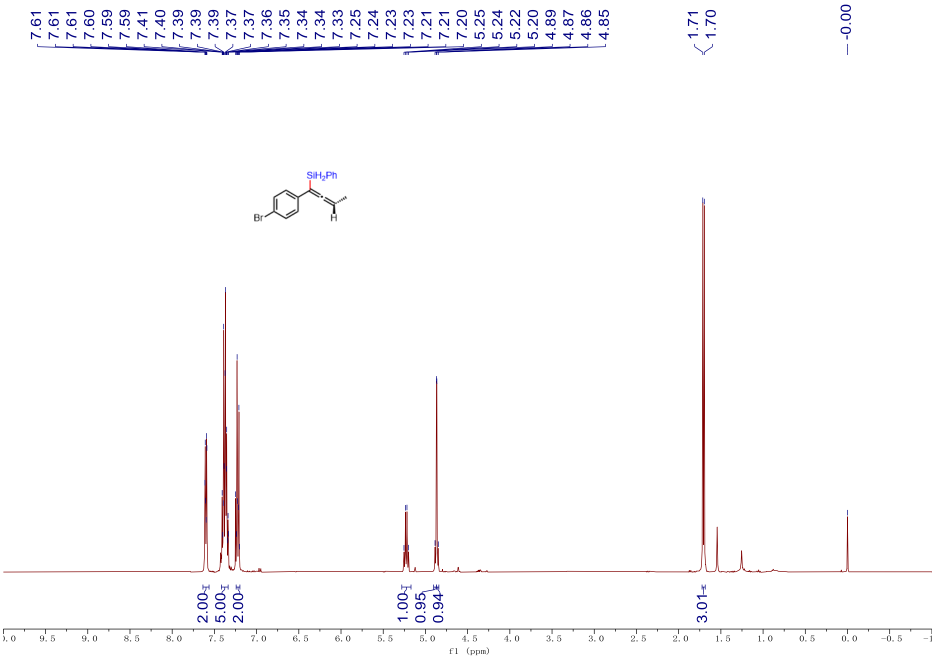

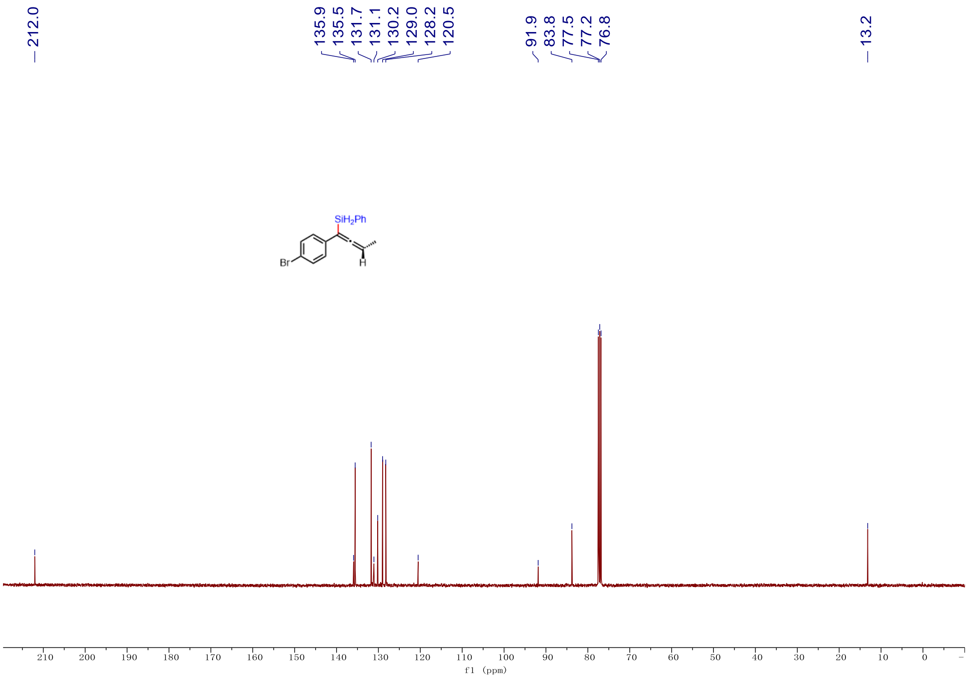

$[\alpha]_D^{20}$ +39.9° (c 1.85, ethyl acetate).

The enantiomeric excess of **6ia** was determined by chiral HPLC analysis on Chiralcel OJ-3 column.

Conditions: hexane : isopropanol = 99:1, flow rate = 0.5 mL/min, UV-Vis detection at λ = 254 nm.

t_{R1} = 12.1 min (minor), t_{R2} = 13.1 min (major).

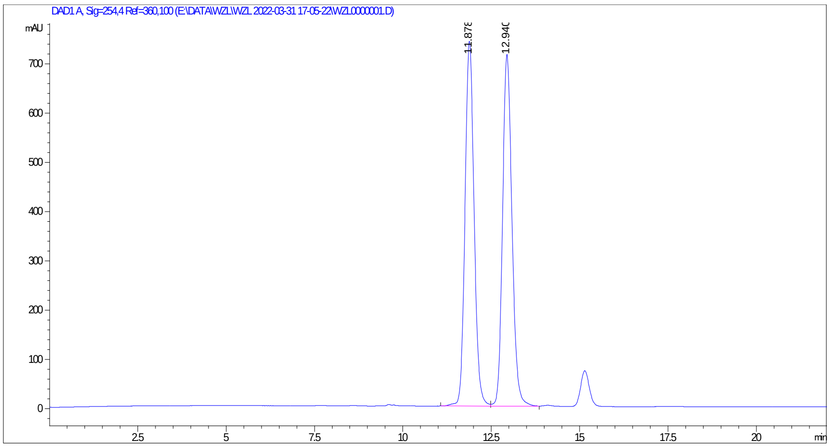

Signal 1: DAD1 A, Sig=254,4 Ref=360,100

Peak #	RetTime [min]	Type	Width [min]	Area [mAU*s]	Height [mAU]	Area %
1	11.878	BV	0.2759	1.31978e4	740.46906	49.9926
2	12.940	VB	0.2858	1.32017e4	713.79303	50.0074

Totals : 2.63995e4 1454.26208

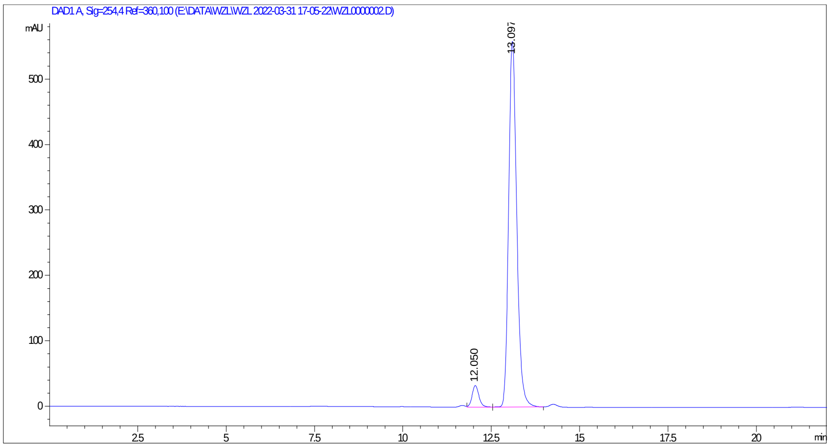

Signal 1: DAD1 A, Sig=254,4 Ref=360,100

Peak #	RetTime [min]	Type	Width [min]	Area [mAU*s]	Height [mAU]	Area %
1	12.050	VB	0.2046	438.61230	33.09156	4.6830
2	13.097	BB	0.2464	8927.38672	558.78113	95.3170

Totals : 9365.99902 591.87269

(*R*)-(1-(4-Methoxyphenyl)buta-1,2-dien-1-yl)(phenyl)silane (6ja)

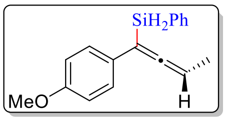

Prepared according to procedure 4 from **1j** (0.2 mmol, 31.6 mg) and **2a** (0.4 mmol, 44.3 mg). The product was isolated in 75% yield with 99% ee value (40.1 mg) as colorless oil.

R*f*: 0.25 (ethyl acetate : petroleum ether = 1:100).

HRMS (ESI) (m/z): Calcd for $C_{17}H_{19}OSi$ $[M+H]^+$: 267.1205, found: 267.1207.

^{1}H NMR (400 MHz, $CDCl_3$) δ 7.64 – 7.60 (m, 2H), 7.42 – 7.32 (m, 3H), 7.32 – 7.27 (m, 2H), 6.84 – 6.79 (m, 2H), 5.20 (q, *J* = 7.1 Hz, 1H), 4.89 (d, *J* = 6.8 Hz, 1H), 4.86 (d, *J* = 6.8 Hz, 1H), 3.77 (s, 3H), 1.70 (d, *J* = 7.1 Hz, 3H).

^{13}C NMR (101 MHz, $CDCl_3$) δ 211.3, 158.6, 135.6, 131.6, 130.0, 129.0, 128.5, 128.1, 114.2, 91.8, 83.3, 55.4, 13.5.

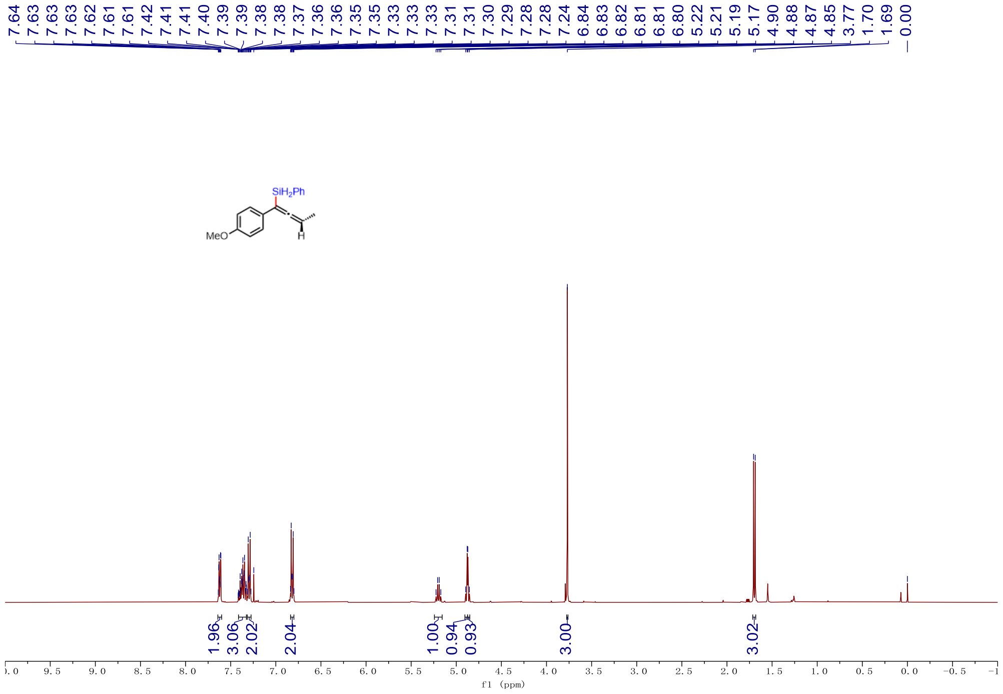

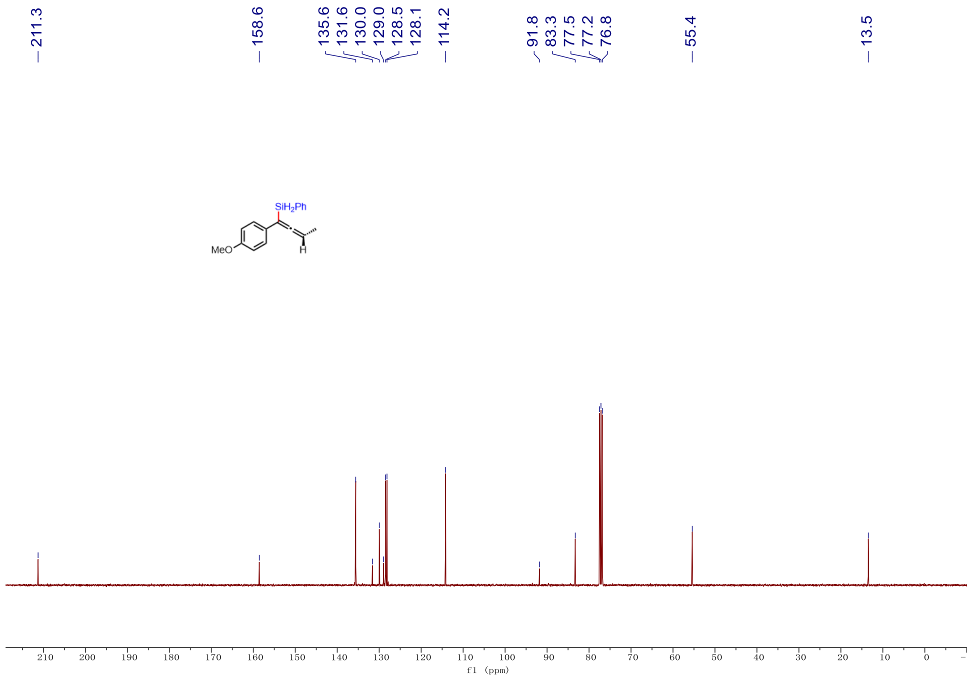

$[\alpha]_D^{20}$ +60.5° (*c* 2.0, ethyl acetate).

The enantiomeric excess of **6ja** was determined by chiral HPLC analysis on Chiralcel OJ-3 column.

Conditions: hexane : isopropanol = 99:1, flow rate = 0.5 mL/min, UV-Vis detection at λ = 254 nm.

t_{R1} = 28.9 min (minor), t_{R2} = 31.4 min (major).

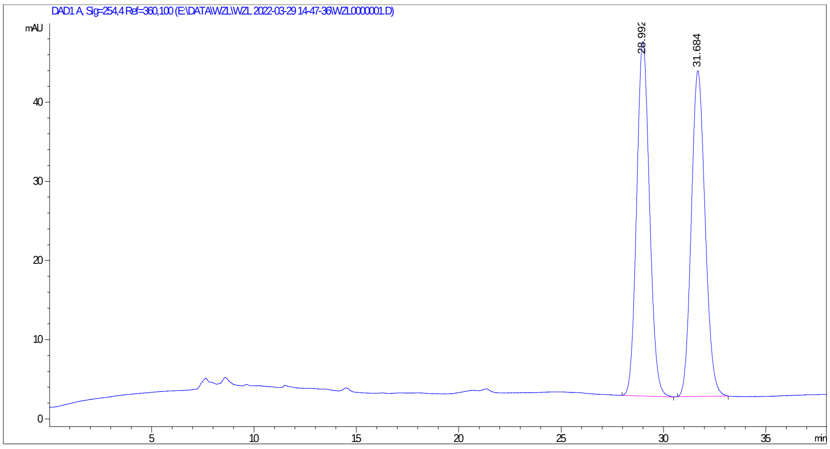

Signal 1: DAD1 A, Sig=254,4 Ref=360,100

Peak #	RetTime [min]	Type	Width [min]	Area [mAU*s]	Height [mAU]	Area %
1	28.992	BB	0.6731	1954.17664	44.77805	50.3054
2	31.684	BB	0.6976	1930.44812	41.12518	49.6946
Totals :				3884.62476	85.90323	

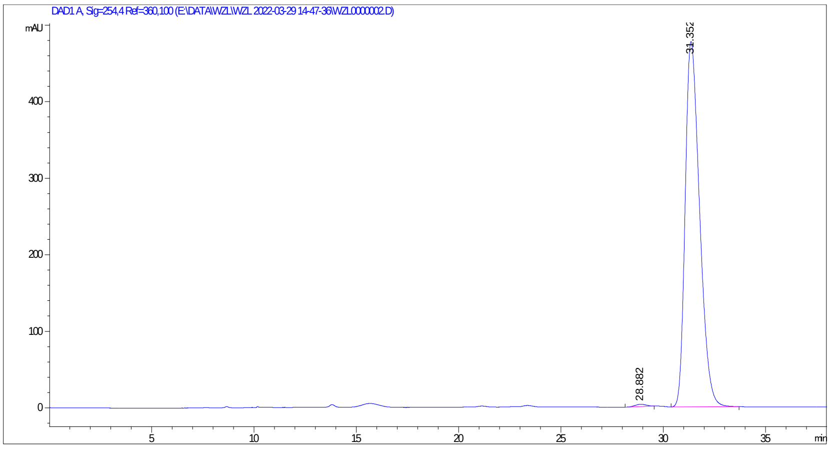

Signal 1: DAD1 A, Sig=254,4 Ref=360,100

Peak #	RetTime [min]	Type	Width [min]	Area [mAU*s]	Height [mAU]	Area %
1	28.882	BB	0.4478	112.28564	3.00584	0.4780
2	31.352	BB	0.7613	2.33787e4	477.44171	99.5220
Totals :				2.34910e4	480.44756	

(*R*)-(1-(Benzo[*d*][1,3]dioxol-5-yl)buta-1,2-dien-1-yl)(phenyl)silane (6ka)

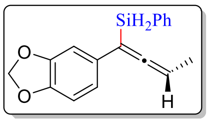

Prepared according to procedure 4 from **1k** (0.2 mmol, 34.4 mg) and **2a** (0.4 mmol, 44.3 mg). The product was isolated in 77% yield with 99% ee value (43.3 mg) as colorless oil.

R*f*: 0.35 (ethyl acetate : petroleum ether = 1:50).

HRMS (ESI) (m/z): Calcd for $C_{17}H_{17}O_2Si$ $[M+H]^+$: 281.0998, found: 281.1013.

^{1}H NMR (400 MHz, $CDCl_3$) δ 7.7 – 7.6 (m, 2H), 7.5 – 7.3 (m, 3H), 6.9 (d, *J* = 2.0 Hz, 1H), 6.8 (dd, *J* = 8.0, 2.0 Hz, 1H), 6.7 (d, *J* = 8.0 Hz, 1H), 5.9 (s, 2H), 5.2 (q, *J* = 7.2 Hz, 1H), 4.87 (d, *J* = 6.8 Hz, 1H), 4.85 (d, *J* = 6.8 Hz, 1H), 1.7 (d, *J* = 7.2 Hz, 3H).

^{13}C NMR (101 MHz, $CDCl_3$) δ 211.4, 148.1, 146.6, 135.6, 131.5, 130.9, 130.1, 128.2, 120.9, 108.4, 107.8, 101.2, 92.4, 83.7, 13.5.

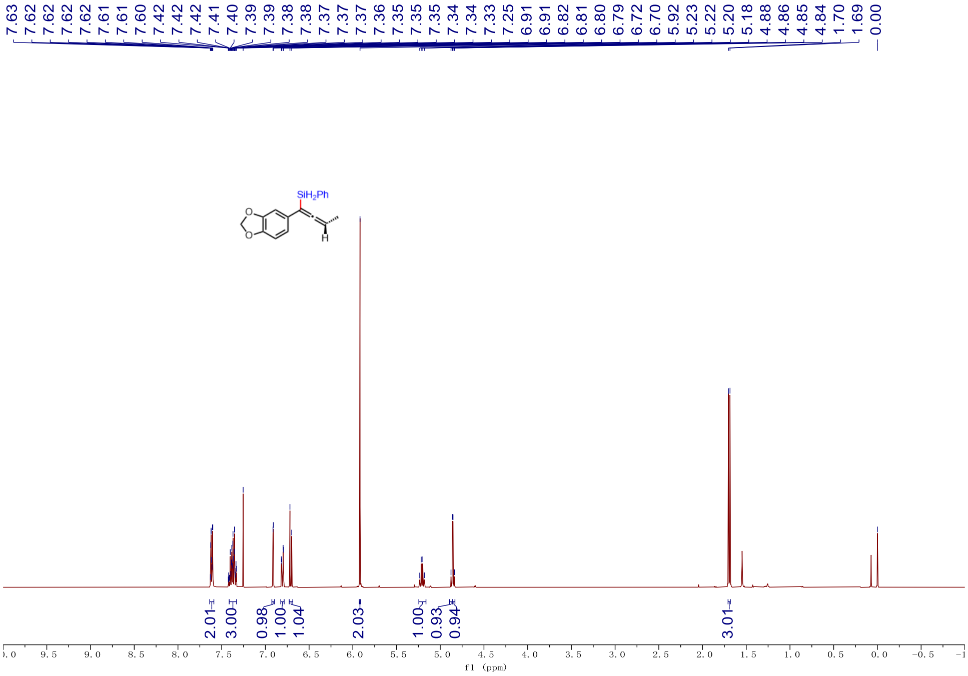

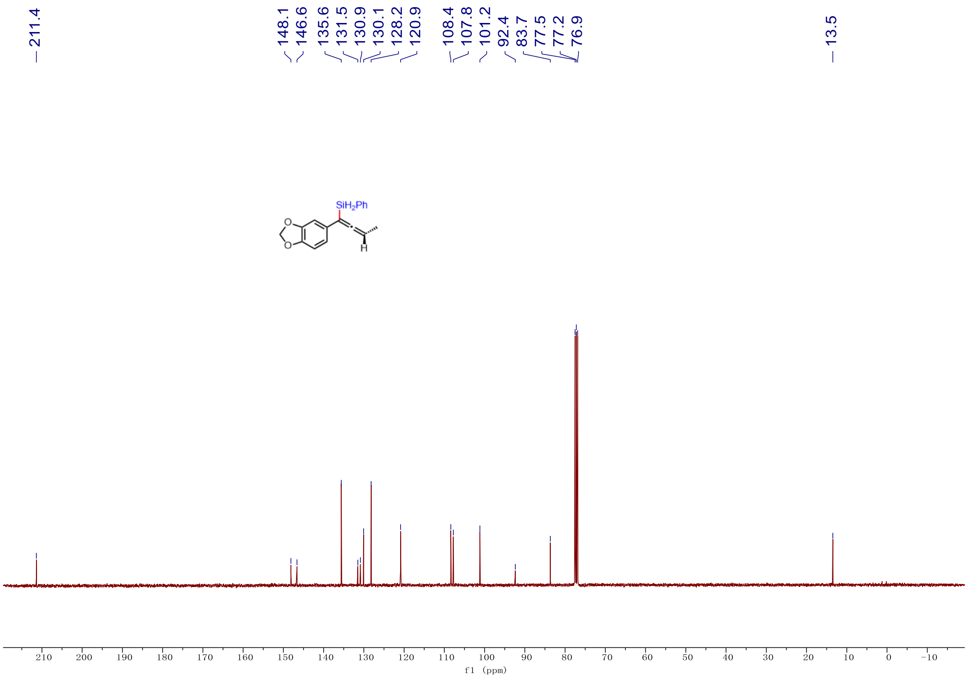

$[\alpha]_D^{20}$ +75.7° (*c* 2.15, ethyl acetate).

The enantiomeric excess of **6ka** was determined by chiral HPLC analysis on Chiralcel OJ-3 column.

Conditions: hexane : isopropanol = 98:2, flow rate = 0.5 mL/min, UV-Vis detection at λ = 254 nm.

t_{R1} = 24.1 min (minor), t_{R2} = 31.6 min (major).

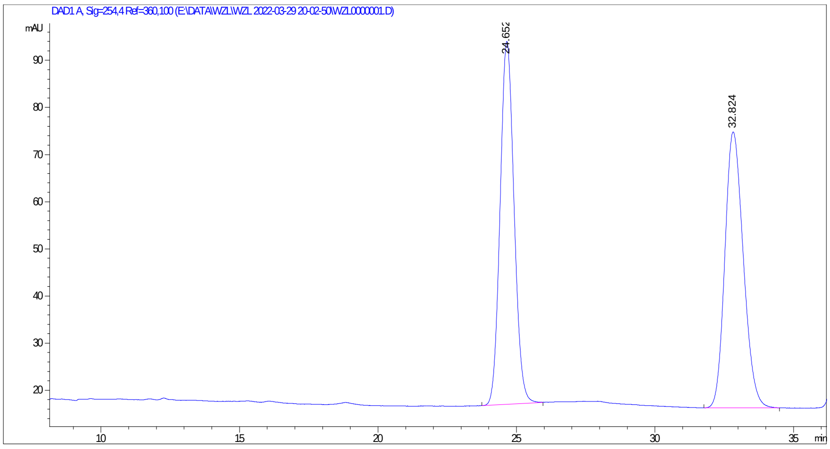

Signal 1: DAD1 A, Sig=254,4 Ref=360,100

Peak #	RetTime [min]	Type	Width [min]	Area [mAU*s]	Height [mAU]	Area %
1	24.652	BB	0.5483	2694.23242	76.98211	49.8368
2	32.824	BB	0.7086	2711.87915	58.52391	50.1632
Totals :				5406.11157	135.50602	

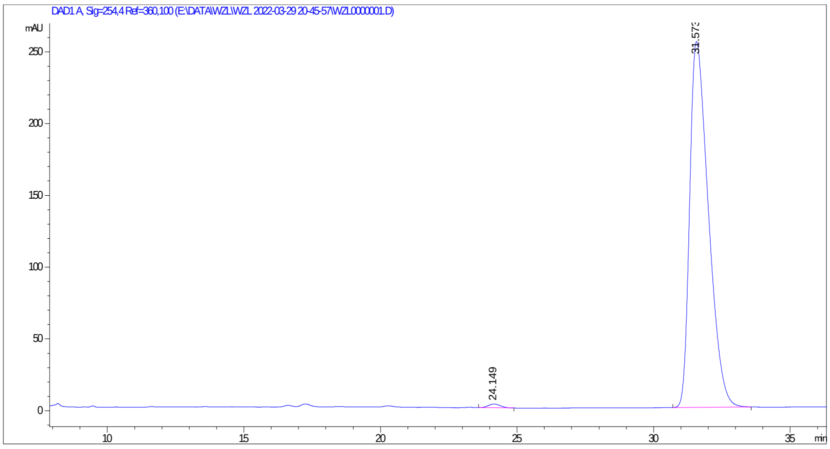

Signal 1: DAD1 A, Sig=254,4 Ref=360,100

Peak #	RetTime [min]	Type	Width [min]	Area [mAU*s]	Height [mAU]	Area %
1	24.149	BB	0.3844	83.74864	2.63771	0.6853
2	31.573	BB	0.7234	1.21364e4	254.92961	99.3147
Totals :				1.22201e4	257.56732	

(*R*)-Phenyl(1-(4-(prop-1-en-2-yl)phenyl)buta-1,2-dien-1-yl)silane (6la)

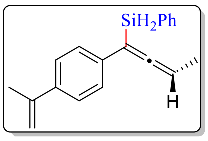

Prepared according to procedure 4 from **1l** (0.2 mmol, 33.6 mg) and **2a** (0.4 mmol, 44.3 mg). The product was isolated in 69% yield with 90% ee value (38.2 mg) as colorless oil.

$\mathbf{R}_f$: 0.50 (petroleum ether).

HRMS (ESI) (m/z): Calcd for $C_{19}H_{21}Si$ $[M+H]^+$: 277.1413, found: 277.1416.

^{1}H NMR (400 MHz, $CDCl_3$) δ 7.65 – 7.60 (m, 2H), 7.43 – 7.30 (m, 7H), 5.34 (s, 1H), 5.23 (q, *J* = 7.1 Hz, 1H), 5.04 (q, *J* = 1.6 Hz, 1H), 4.93 – 4.86 (m, 2H), 2.11 (s, 3H), 1.71 (d, *J* = 7.2 Hz, 3H).

^{13}C NMR (101 MHz, $CDCl_3$) δ 212.0, 142.9, 139.5, 136.0, 135.6, 131.5, 130.0, 128.2, 127.2, 125.8, 112.3, 92.3, 83.4, 21.9, 13.3.

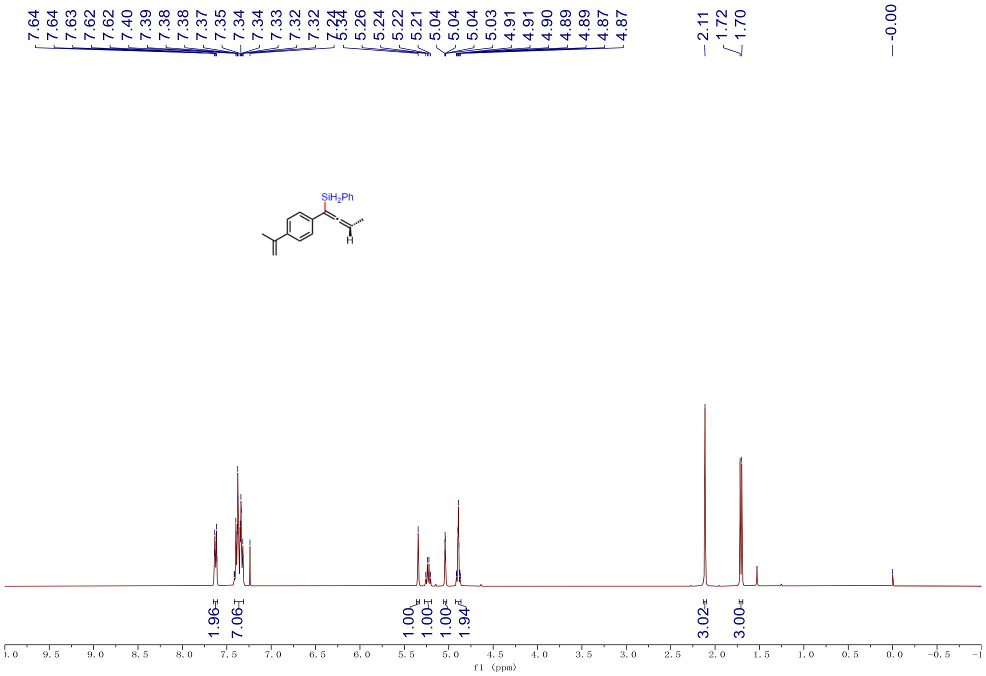

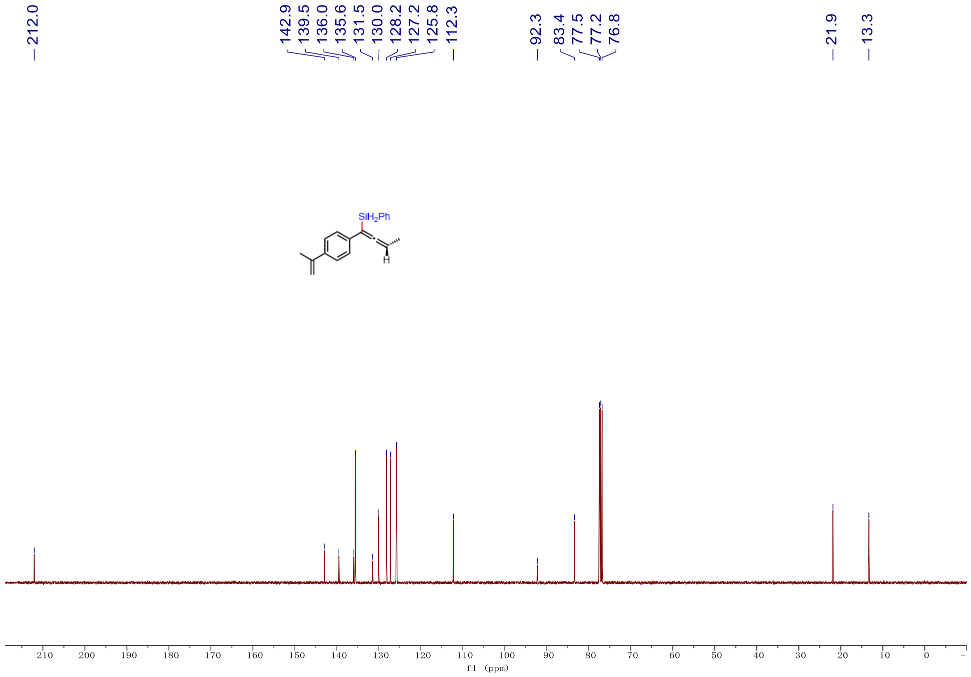

$[\alpha]_D^{20}$ +35.5° (*c* 1.9, ethyl acetate).

The enantiomeric excess of **6la** was determined by chiral HPLC analysis on Chiralcel OJ-3 column.

Conditions: hexane : isopropanol = 98:2, flow rate = 0.5 mL/min, UV-Vis detection at λ = 254 nm.

t_{R1} = 15.3 min (minor), t_{R2} = 17.0 min (major).

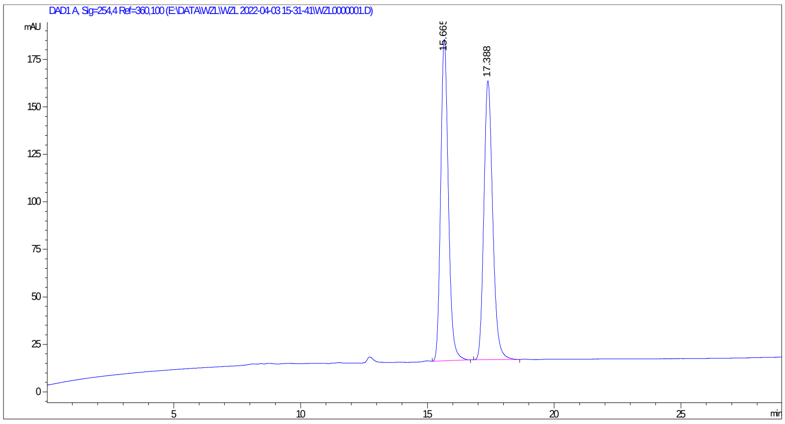

Signal 1: DAD1 A, Sig=254,4 Ref=360,100

Peak #	RetTime [min]	Type	Width [min]	Area [mAU*s]	Height [mAU]	Area %
1	15.665	BB	0.3233	3558.04443	169.23187	50.0481
2	17.388	BB	0.3742	3551.20825	146.83270	49.9519
Totals :				7109.25269	316.06458	

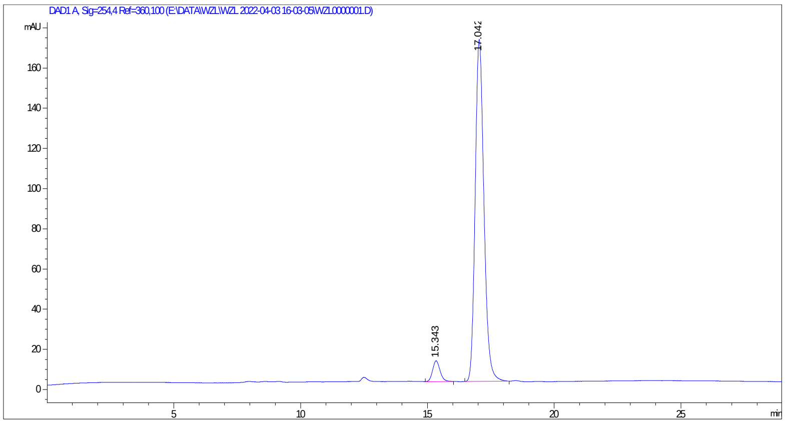

Signal 1: DAD1 A, Sig=254,4 Ref=360,100

Peak #	RetTime [min]	Type	Width [min]	Area [mAU*s]	Height [mAU]	Area %
1	15.343	BB	0.3216	210.06747	10.31301	4.8975
2	17.042	BB	0.3695	4079.20728	170.24422	95.1025
Totals :				4289.27475	180.55723	

(*R*)-Phenyl(1-(thiophen-3-yl)buta-1,2-dien-1-yl)silane (6ma)

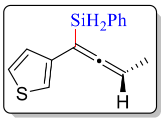

Prepared according to procedure 4 from **1m** (0.2 mmol, 26.8 mg) and **2a** (0.4 mmol, 44.3 mg). The product was isolated in 72% yield with 98% ee value (34.9 mg) as colorless oil.

R*f*: 0.60 (petroleum ether).

HRMS (ESI) (m/z): Calcd for $C_{14}H_{15}SSi$ $[M+H]^+$: 243.0664, found: 243.0670.

^{1}H NMR (400 MHz, $CDCl_3$) δ 7.66 – 7.61 (m, 2H), 7.44 – 7.33 (m, 3H), 7.26 – 7.21 (m, 1H), 7.12 (dd, *J* = 5.0, 1.3 Hz, 1H), 7.06 – 7.03 (m, 1H), 5.18 (q, *J* = 7.2 Hz, 1H), 4.86 (d, *J* = 6.8 Hz, 1H), 4.84 (d, *J* = 6.8 Hz, 1H), 1.69 (d, *J* = 7.1 Hz, 3H).

^{13}C NMR (101 MHz, $CDCl_3$) δ 211.7, 137.7, 135.6, 131.2, 130.1, 128.2, 127.3, 125.5, 120.7, 87.6, 83.0, 13.4.

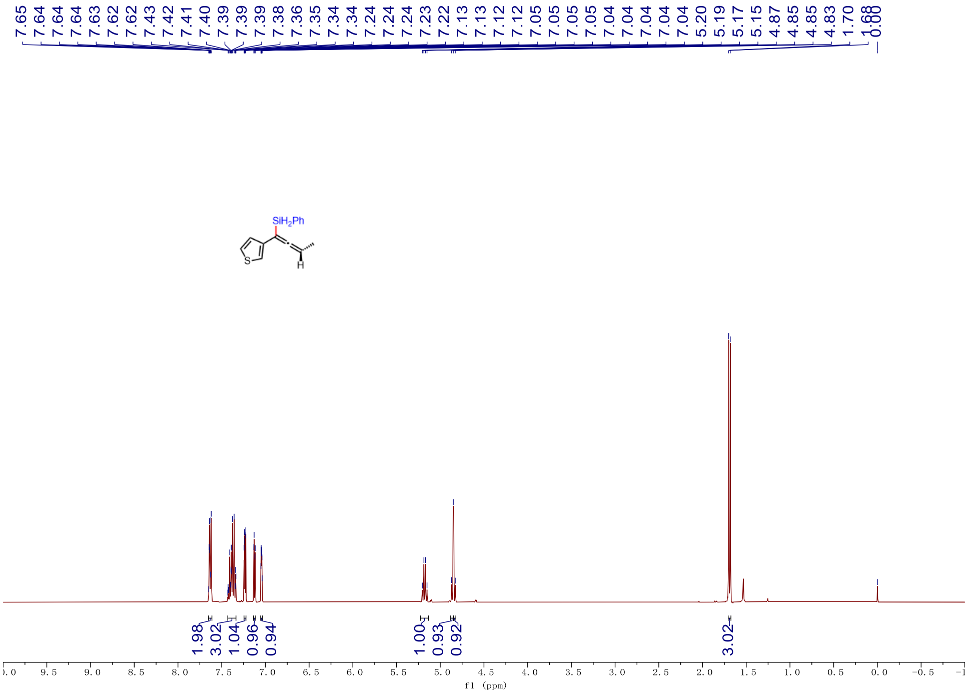

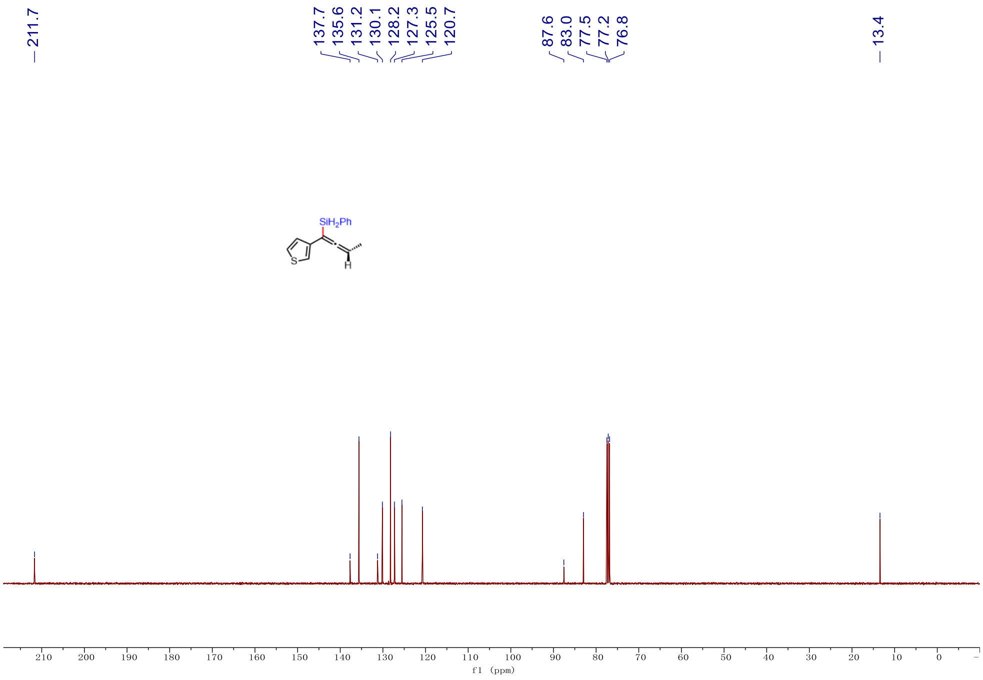

$[\alpha]_D^{20}$ +46.1° (c 1.9, ethyl acetate).
The enantiomeric excess of **6ma** was determined by chiral HPLC analysis on Chiralcel OJ-3 column.
Conditions: hexane : isopropanol = 99:1, flow rate = 0.5 mL/min, UV-Vis detection at λ = 254 nm.
t_{R1} = 13.4 min (major), t_{R2} = 14.2 min (minor).

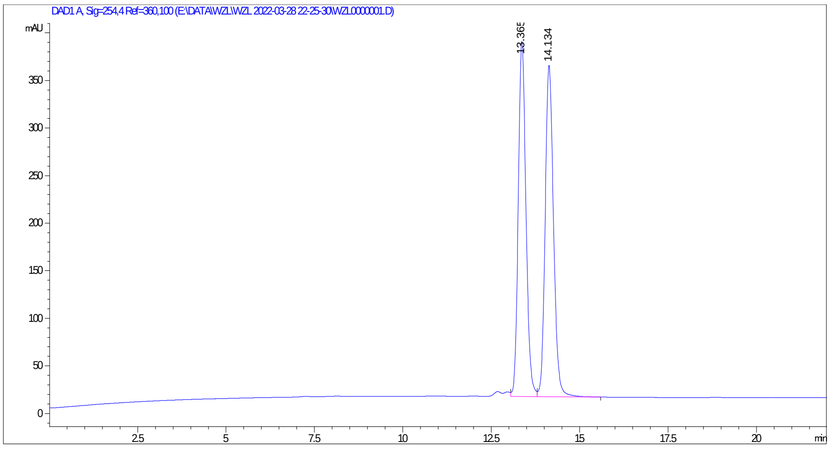

Signal 1: DAD1 A, Sig=254,4 Ref=360,100

Peak #	RetTime [min]	Type	Width [min]	Area [mAU*s]	Height [mAU]	Area %
1	13.365	VV	0.2343	5641.59521	373.33130	49.6123
2	14.134	VB	0.2518	5729.76367	348.56696	50.3877
Totals :				1.13714e4	721.89825	

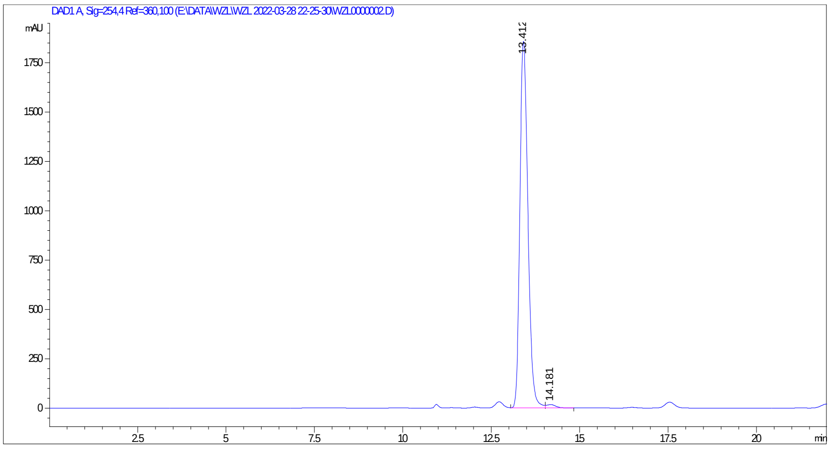

Signal 1: DAD1 A, Sig=254,4 Ref=360,100

Peak #	RetTime [min]	Type	Width [min]	Area [mAU*s]	Height [mAU]	Area %
1	13.412	VV	0.2463	2.93437e4	1857.50220	98.9233
2	14.181	VB	0.2753	319.39310	17.14712	1.0767
Totals :				2.96631e4	1874.64932	

(R)-3-(1-(Phenylsilyl)buta-1,2-dien-1-yl)-1-tosyl-1H-indole (6na)

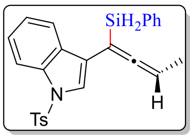

Prepared according to procedure 4 from **1o** (0.2 mmol, 64.3 mg) and **2a** (0.6 mmol, 64.9 mg). The reaction mixture was stirred at -50 °C for 30 h in 2.0 mL dry DCE. The product was isolated in 57% yield with 90% ee value (49.1 mg) as pale-yellow oil.

R$_f$: 0.30 (ethyl acetate : petroleum ether = 1:20).

HRMS (ESI) (m/z): Calcd for $C_{25}H_{24}NO_2SSi$ $[M+H]^+$: 430.1297, found: 430.1304.

^{1}H NMR (400 MHz, $CDCl_3$) δ 7.94 (t, J = 7.5 Hz, 2H), 7.67 – 7.62 (m, 2H), 7.61 – 7.55 (m, 2H), 7.49 – 7.42 (m, 1H), 7.41 – 7.35 (m, 3H), 7.33 – 7.27 (m, 1H), 7.24 – 7.19 (m, 1H), 7.13 (d, J = 8.2 Hz, 2H), 5.26 (q, J = 7.2 Hz, 1H), 4.91 (d, J = 6.8 Hz, 1H), 4.88 (d, J = 6.8 Hz, 1H), 2.32 (s, 3H), 1.76 (d, J = 7.1 Hz, 3H).

^{13}C NMR (101 MHz, $CDCl_3$) δ 212.1, 144.9, 135.7, 135.5, 135.1, 131.0, 130.3, 130.2, 129.9, 128.3, 126.9, 125.1, 123.8, 123.5, 121.2, 118.0, 113.8, 84.2, 83.2, 21.7, 13.9.

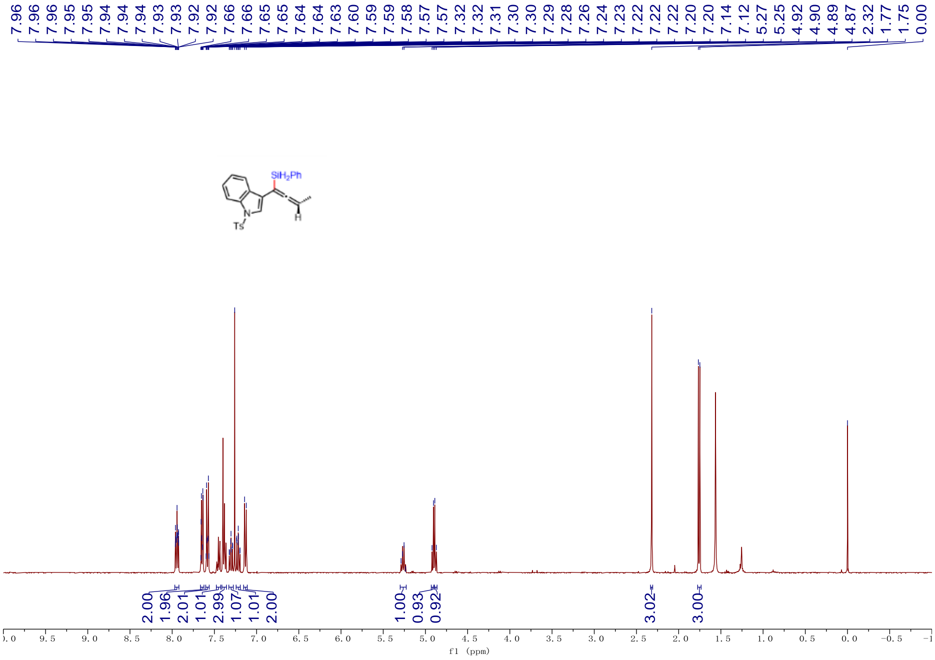

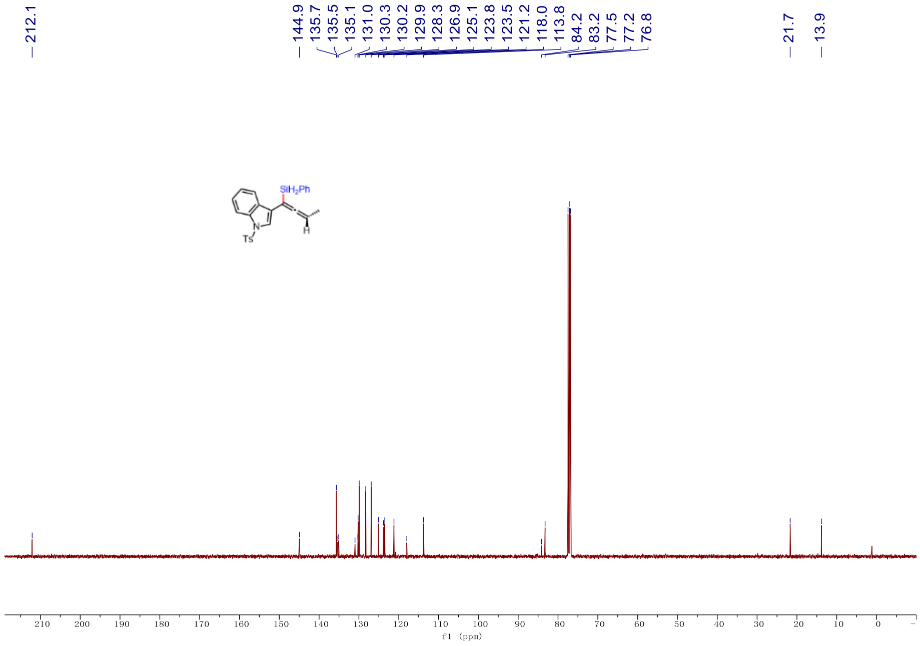

$[\alpha]_D^{20}$ +33.4° (c 2.45, ethyl acetate).

The enantiomeric excess of 6**na** was determined by chiral HPLC analysis on Chiralcel OJ-3 column.

Conditions: hexane : isopropanol = 90:10, flow rate = 0.5 mL/min, UV-Vis detection at λ = 254 nm.

t_{R1} = 26.6 min (minor), t_{R2} = 43.6 min (major).

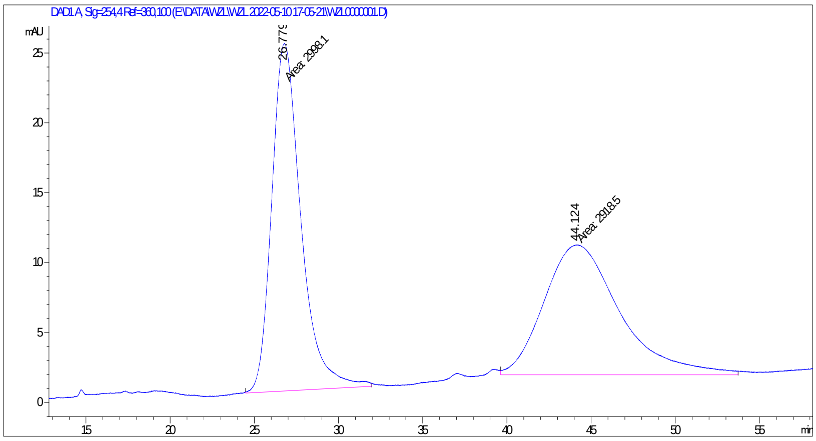

Signal 1: DAD1 A, Sig=254,4 Ref=360,100

Peak #	RetTime [min]	Type	Width [min]	Area [mAU*s]	Height [mAU]	Area %
1	26.779	MM	2.0100	2998.09546	24.85973	50.6727
2	44.124	MM	5.2227	2918.49780	9.31355	49.3273
Totals :				5916.59326	34.17327	

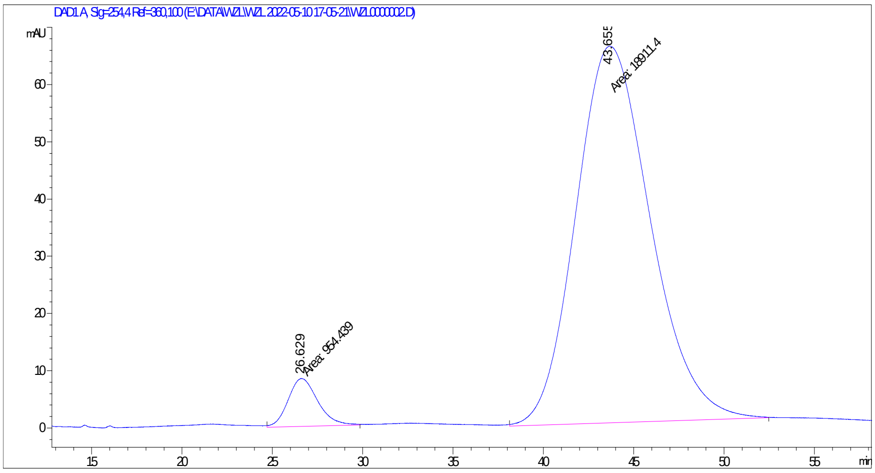

Signal 1: DAD1 A, Sig=254,4 Ref=360,100

Peak #	RetTime [min]	Type	Width [min]	Area [mAU*s]	Height [mAU]	Area %
1	26.629	MM	1.9023	954.43921	8.36227	4.8044
2	43.655	MM	4.7907	1.89114e4	65.79268	95.1956
Totals :				1.98659e4	74.15495	

(*R*)-Phenyl(7-phenylhepta-2,3-dien-4-yl)silane (6oa)

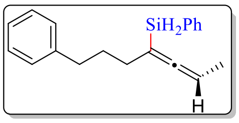

Prepared according to procedure 4 from **1o** (0.2 mmol, 34.1 mg) and **2a** (0.4 mmol, 44.3 mg). The product was isolated in 58% yield with 98% ee value (32.4 mg) as colorless oil.

R*f*: 0.50 (petroleum ether).

HRMS (ESI) (m/z): Calcd for $C_{19}H_{23}Si$ $[M+H]^+$: 279.1569, found: 279.1591.

^{1}H NMR (400 MHz, $CDCl_3$) δ 7.60 – 7.57 (m, 2H), 7.44 – 7.33 (m, 3H), 7.29 – 7.23 (m, 2H), 7.19 – 7.11 (m, 3H), 4.92 – 4.82 (m, 1H), 4.58 (d, *J* = 1.0 Hz, 2H), 2.60 (dd, *J* = 7.6, 7.6 Hz, 2H), 2.08 (ddd, *J* = 7.4, 7.4, 2.9 Hz, 2H), 1.82 – 1.72 (m, 2H), 1.62 (d, *J* = 7.0 Hz, 3H).

^{13}C NMR (101 MHz, $CDCl_3$) δ 209.5, 142.6, 135.6, 131.9, 129.9, 128.6, 128.4, 128.1, 125.8, 89.0, 80.9, 35.4, 30.8, 30.7, 13.8.

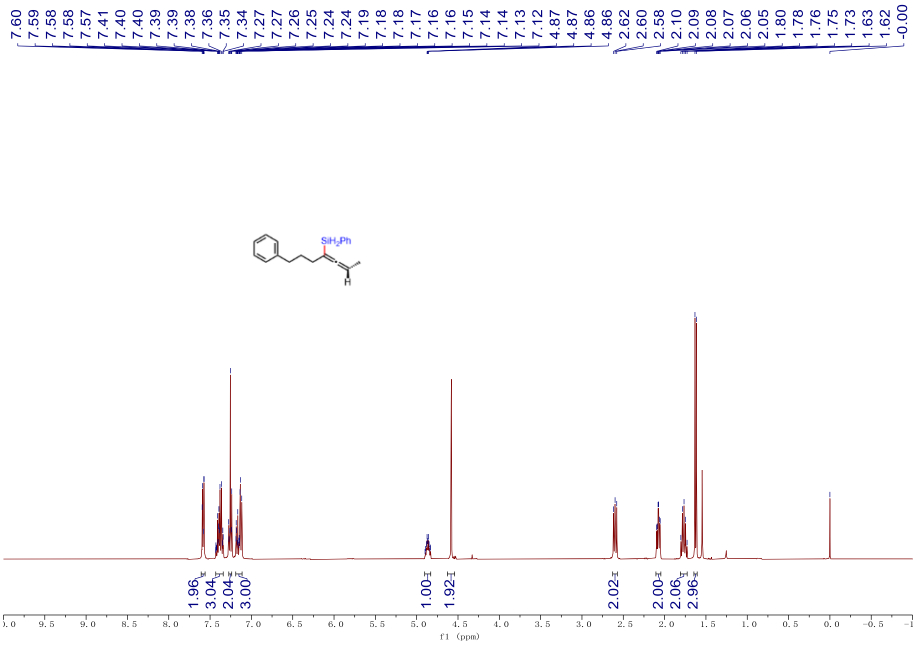

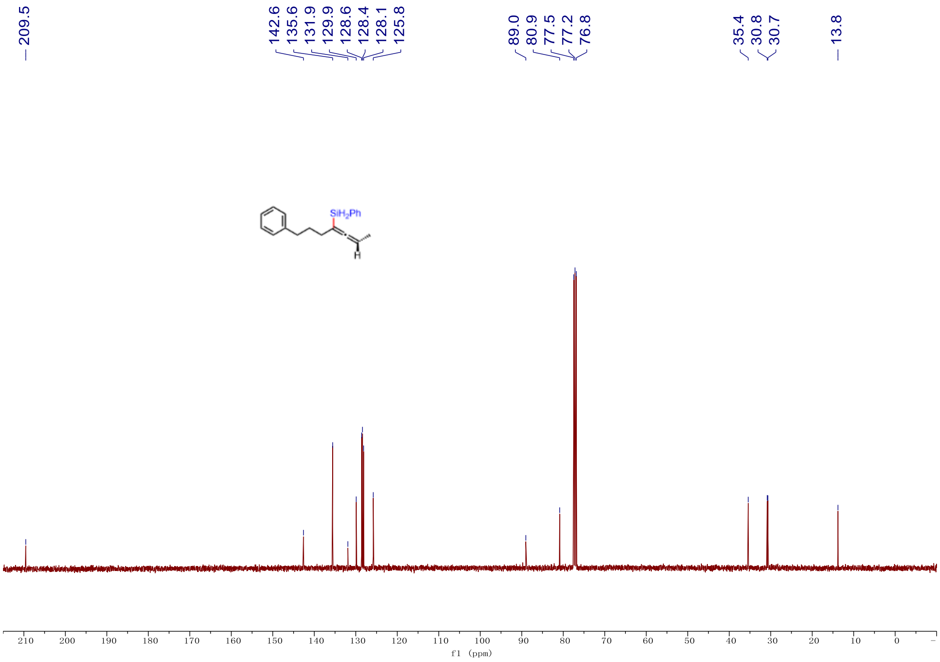

$[\alpha]_D^{20}$ -4.1° (c 1.6, ethyl acetate).
The enantiomeric excess of **6oa** was determined by chiral HPLC analysis on Chiralcel OJ-3 column.
Conditions: hexane : isopropanol = 100:1, flow rate = 0.5 mL/min, UV-Vis detection at λ = 220 nm.
t_{R1} = 18.9 min (minor), t_{R2} = 28.5 min (major).

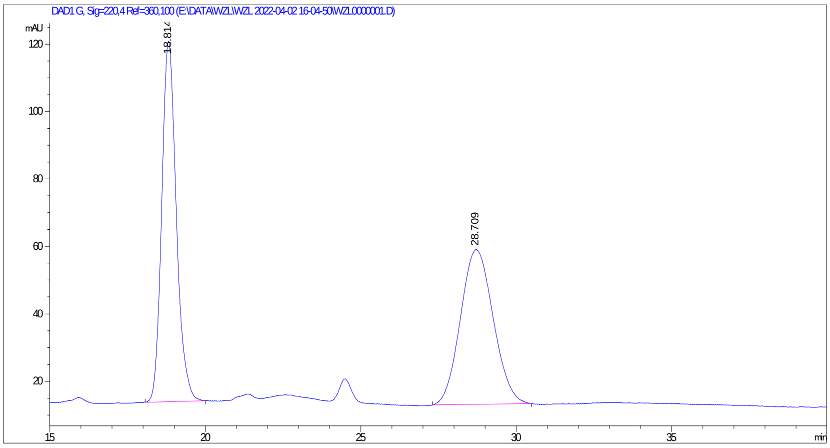

Signal 7: DAD1 G, Sig=220,4 Ref=360,100

Peak #	RetTime [min]	Type	Width [min]	Area [mAU*s]	Height [mAU]	Area %
1	18.814	BB	0.5059	3479.55298	106.84384	50.8192
2	28.709	BB	0.8686	3367.36865	45.97173	49.1808

Totals : 6846.92163 152.81557

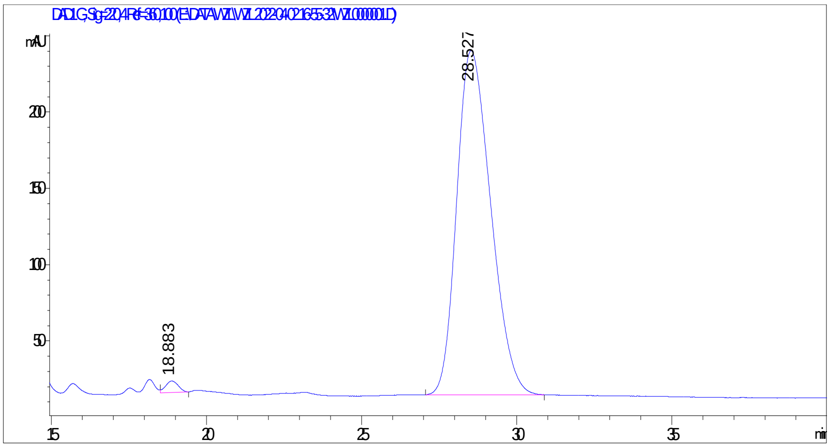

Signal 7: DAD1 G, Sig=220,4 Ref=360,100

Peak #	RetTime [min]	Type	Width [min]	Area [mAU*s]	Height [mAU]	Area %
1	18.883	VB	0.3462	215.12585	7.55406	1.2351
2	28.527	BB	1.1156	1.72031e4	225.61787	98.7649

Totals : 1.74182e4 233.17193

(*R*)-(7-Chlorohepta-2,3-dien-4-yl)(phenyl)silane (6pa)

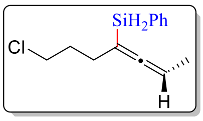

Prepared according to procedure 4 from **1p** (0.2 mmol, 25.7 mg) and **2a** (0.4 mmol, 44.3 mg). The product was isolated in 45% yield with 96% ee value (21.3 mg) as colorless oil.

R*f*: 0.50 (petroleum ether).

HRMS (ESI) (m/z): Calcd for $C_{13}H_{18}ClSi$ $[M+H]^+$: 237.0866, found: 237.0832.

^{1}H NMR (400 MHz, $CDCl_3$) δ 7.62 – 7.55 (m, 2H), 7.46 – 7.33 (m, 3H), 4.96 – 4.85 (m, 1H), 4.58 (s, 2H), 3.53 (dd, *J* = 6.6, 6.6 Hz, 2H), 2.17 (ddd, *J* = 7.2, 7.2, 2.9 Hz, 2H), 1.98 – 1.88 (m, 2H), 1.62 (d, *J* = 7.0 Hz, 3H).

^{13}C NMR (101 MHz, $CDCl_3$) δ 209.5, 135.5, 131.5, 130.0, 128.2, 88.1, 81.5, 44.5, 31.8, 28.1, 13.7.

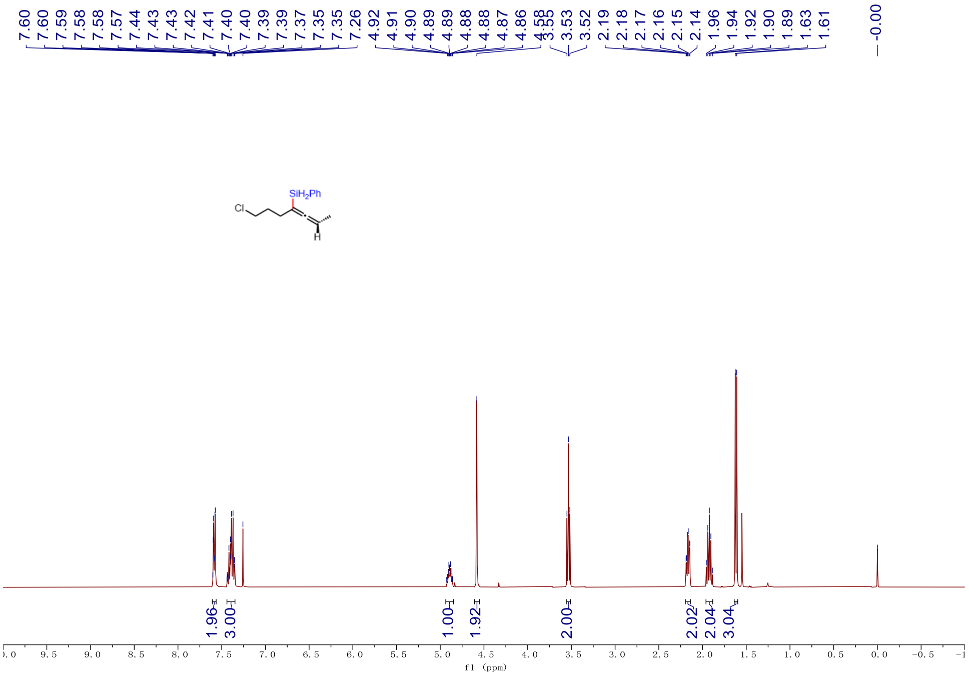

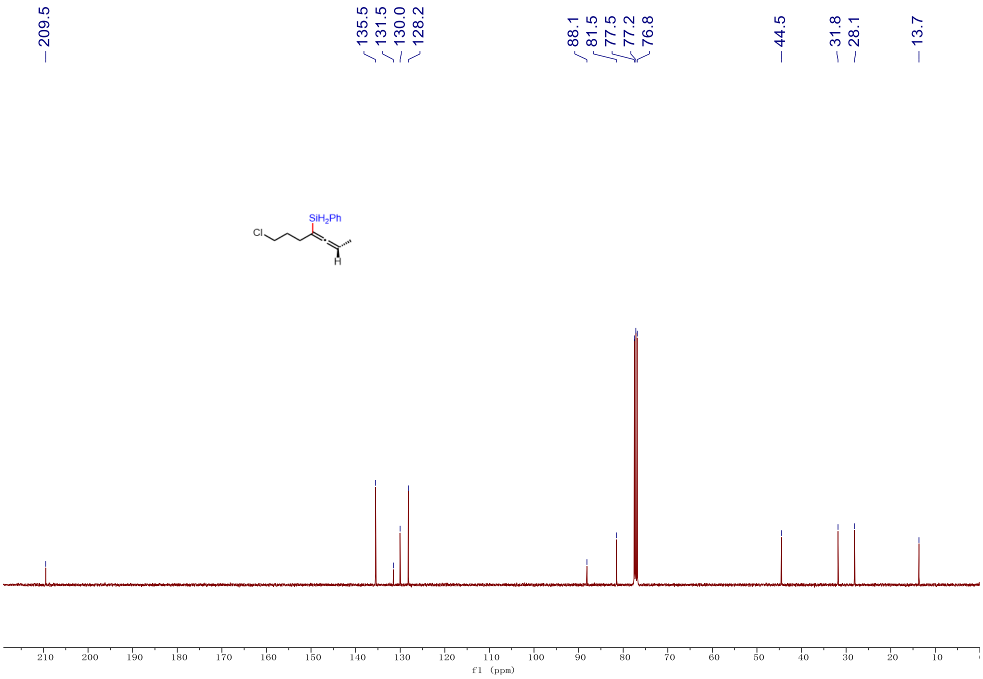

$[\alpha]_D^{20}$ -1.1° (*c* 2.1, ethyl acetate).

The enantiomeric excess of **6pa** was determined by chiral HPLC analysis on Chiralcel OJ-3 column.

Conditions: hexane : isopropanol = 100:1, flow rate = 0.5 mL/min, UV-Vis detection at λ = 254 nm.

t_{R1} = 11.3 min (minor), t_{R2} = 13.2 min (major).

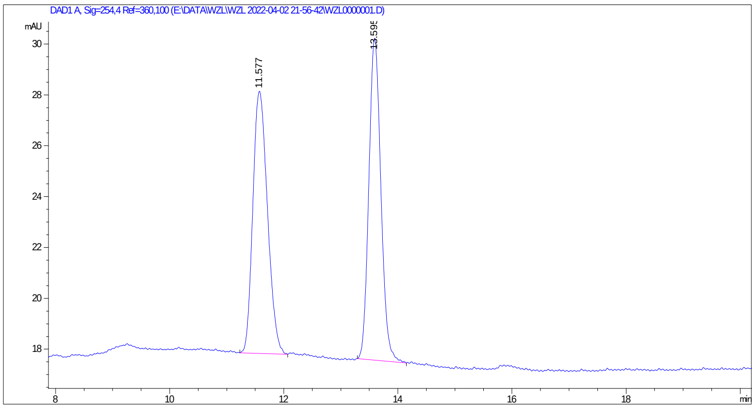

Signal 1: DAD1 A, Sig=254,4 Ref=360,100

Peak #	RetTime [min]	Type	Width [min]	Area [mAU*s]	Height [mAU]	Area %
1	11.577	BB	0.2735	183.38835	10.30890	49.7507
2	13.595	BB	0.2248	185.22615	12.65535	50.2493
Totals :				368.61450	22.96424	

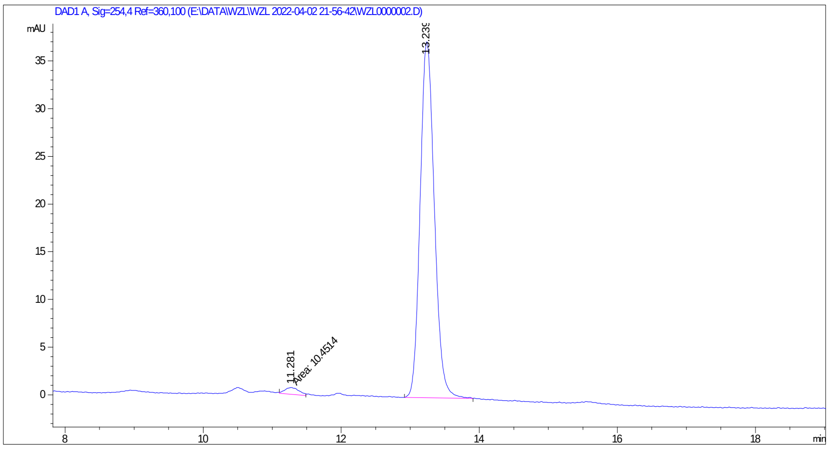

Signal 1: DAD1 A, Sig=254,4 Ref=360,100

Peak #	RetTime [min]	Type	Width [min]	Area [mAU*s]	Height [mAU]	Area %
1	11.281	MM	0.2416	10.45135	7.20853e-1	1.9221
2	13.239	BB	0.2228	533.30090	37.30573	98.0779
Totals :				543.75226	38.02658	

(*R*)-(1-(Cyclohex-1-en-1-yl)buta-1,2-dien-1-yl)(phenyl)silane (6qa)

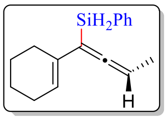

Prepared according to procedure 4 from **1q** (0.2 mmol, 26.4 mg) and **2a** (0.4 mmol, 44.3 mg). The product was isolated in 79% yield with 98% ee value (38.0 mg) as colorless oil.

R*f*: 0.80 (petroleum ether).

HRMS (EI) (m/z): Calcd for $C_{16}H_{20}Si$ $[M]^+$: 240.1334, found: 240.1336.

^{1}H NMR (400 MHz, $CDCl_3$) δ 7.63 – 7.56 (m, 2H), 7.43 – 7.32 (m, 3H), 5.79 – 5.70 (m, 1H), 5.02 (q, *J* = 7.1 Hz, 1H), 4.73 (d, *J* = 6.8 Hz, 1H), 4.70 (d, *J* = 6.8 Hz, 1H), 2.18 – 2.03 (m, 4H), 1.69 – 1.62 (m, 2H), 1.60 (d, *J* = 7.1 Hz, 3H), 1.58 – 1.52 (m, 2H).

^{13}C NMR (101 MHz, $CDCl_3$) δ 210.8, 135.6, 134.0, 132.2, 129.7, 128.0, 126.3, 94.8, 83.1, 27.7, 26.2, 23.0, 22.4, 13.8.

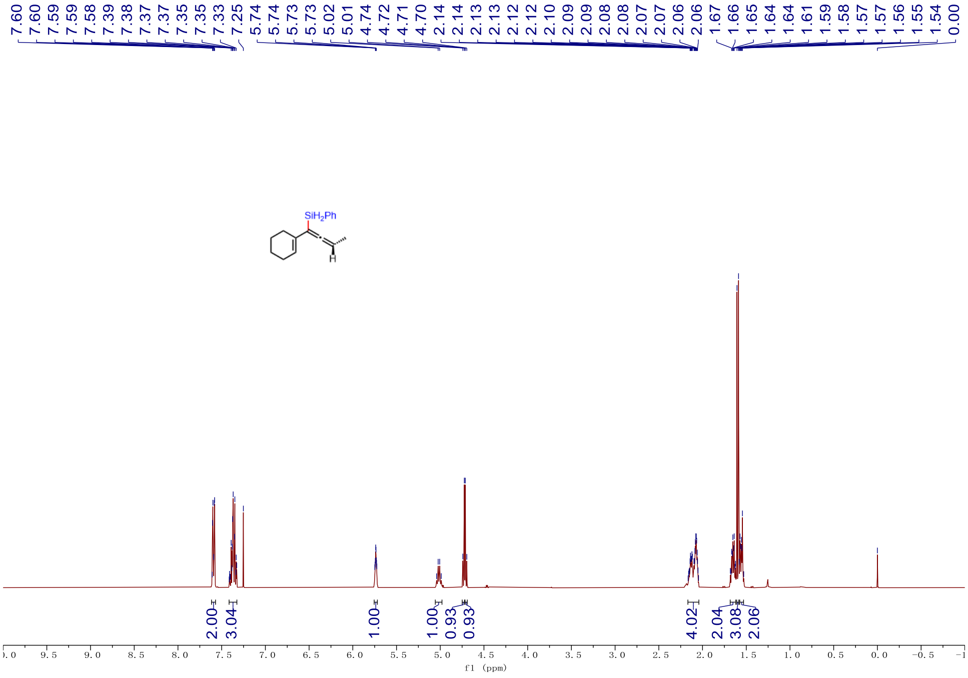

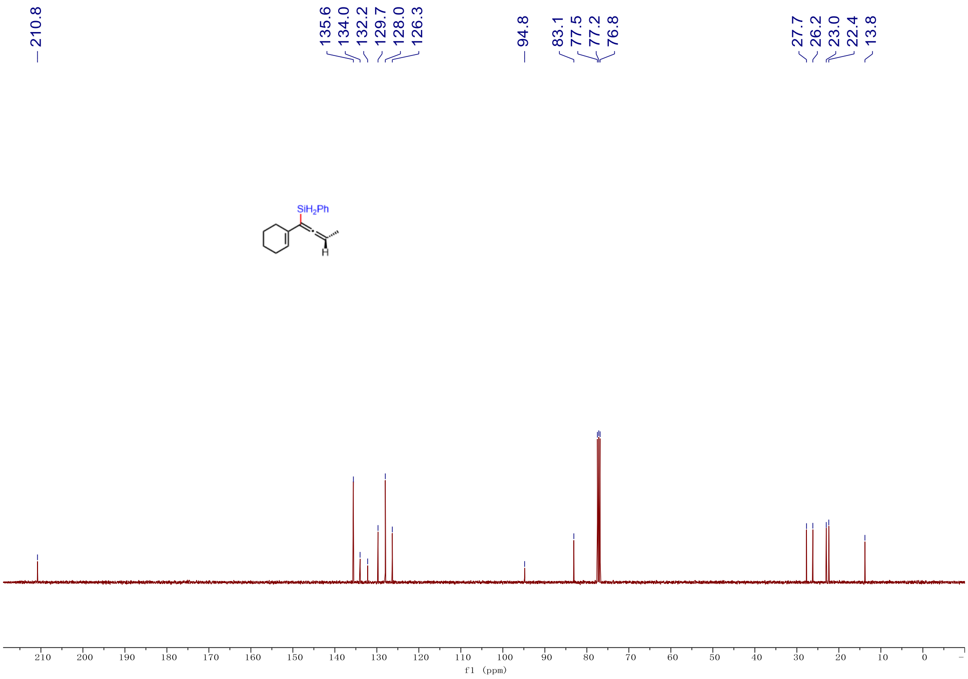

$[\alpha]_D^{20}$ +3.2° (*c* 1.9, ethyl acetate).

The enantiomeric excess of **6qa** was determined by chiral HPLC analysis on Chiralcel OJ-3 column.

Conditions: hexane : isopropanol = 99:1, flow rate = 0.5 mL/min, UV-Vis detection at λ = 220 nm.

t_{R1} = 8.9 min (minor), t_{R2} = 9.3 min (major).

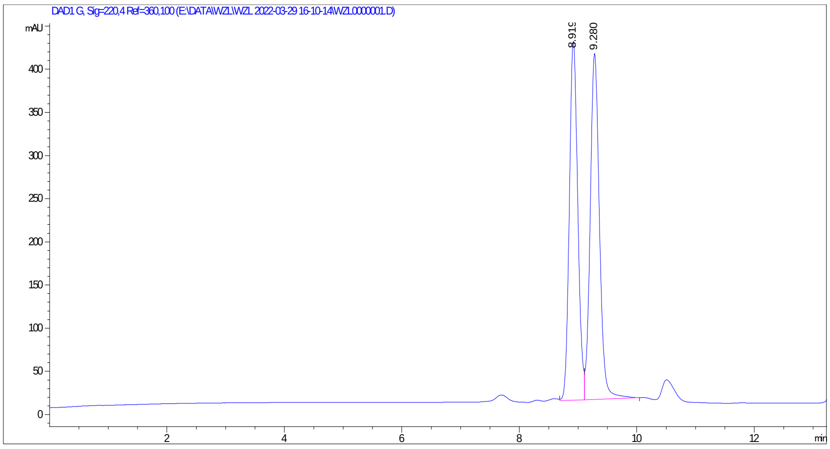

Signal 7: DAD1 G, Sig=220,4 Ref=360,100

Peak #	RetTime [min]	Type	Width [min]	Area [mAU*s]	Height [mAU]	Area %
1	8.919	VV	0.1542	4141.19775	415.76837	48.5780
2	9.280	VB	0.1676	4383.64990	400.67416	51.4220
Totals :				8524.84766	816.44254	

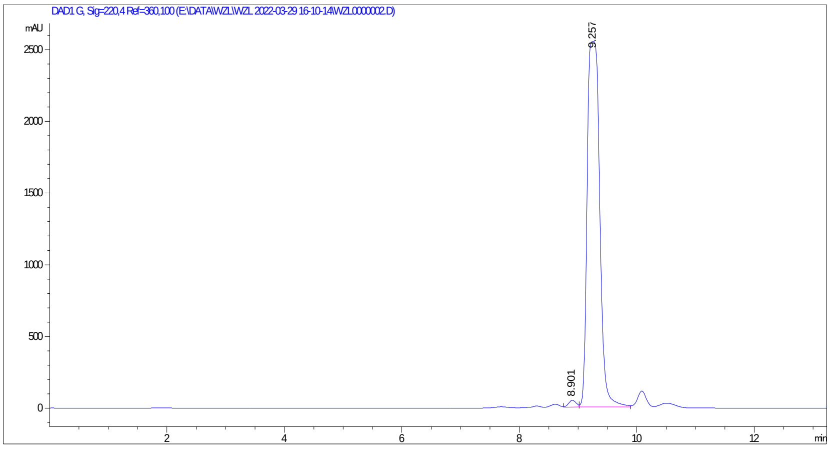

Signal 7: DAD1 G, Sig=220,4 Ref=360,100

Peak #	RetTime [min]	Type	Width [min]	Area [mAU*s]	Height [mAU]	Area %
1	8.901	VV	0.1432	427.86746	47.43379	1.1277
2	9.257	VV	0.2336	3.75143e4	2550.14233	98.8723
Totals :				3.79421e4	2597.57613	

(*R*)-(*E*)-(1-(cyclooct-1-en-1-yl)buta-1,2-dien-1-yl)(phenyl)silane (6ra)

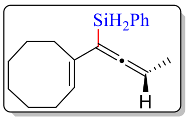

Prepared according to procedure 4 from **1r** (0.2 mmol, 32.1 mg) and **2a** (0.4 mmol, 44.3 mg). The product was isolated in 78% yield with 93% ee value (41.9 mg) as colorless oil

R*f*: 0.80 (petroleum ether).

HRMS (ESI) (m/z): Calcd for $C_{18}H_{25}Si$ $[M+H]^+$: 269.1726, found: 269.1724.

^{1}H NMR (400 MHz, $CDCl_3$) δ 7.62 – 7.57 (m, 2H), 7.41 – 7.31 (m, 3H), 5.65 (t, *J* = 8.1 Hz, 1H), 5.02 (q, *J* = 7.1 Hz, 1H), 4.74 (d, *J* = 6.8 Hz, 1H), 4.71 (d, *J* = 6.8 Hz, 1H), 2.45 – 2.32 (m, 2H), 2.20 – 2.10 (m, 2H), 1.62 (d, *J* = 7.0 Hz, 3H), 1.54 – 1.38 (m, 8H).

^{13}C NMR (101 MHz, $CDCl_3$) δ 211.1, 137.1, 135.5, 132.3, 129.7, 129.3, 128.0, 94.4, 82.7, 30.5, 28.9, 27.7, 27.7, 27.0, 26.2, 13.7.

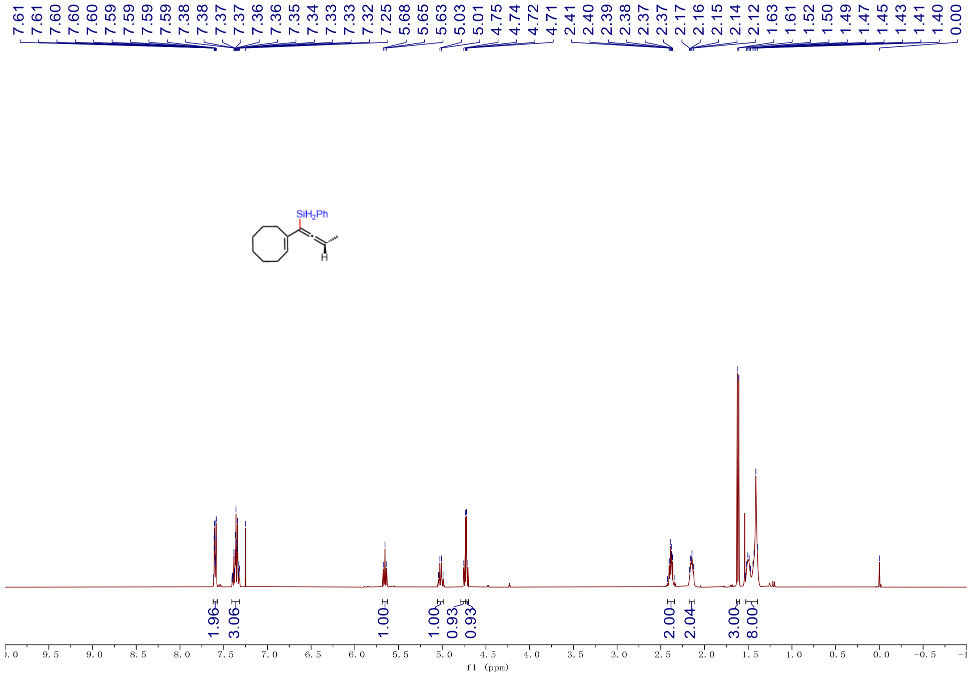

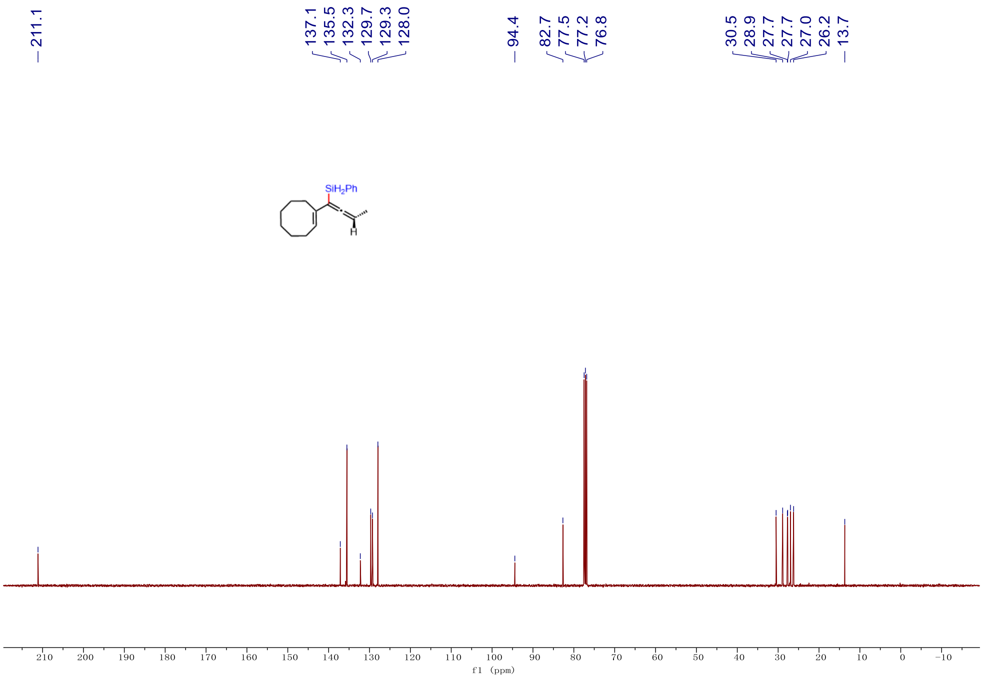

$[\alpha]_D^{20}$ +10.9° (c 2.05, ethyl acetate).

The enantiomeric excess of **6ra** was determined by chiral HPLC analysis on Chiralcel OJ-3 column.

Conditions: hexane : isopropanol = 100:0, flow rate = 0.5 mL/min, UV-Vis detection at λ = 254 nm.

t_{R1} = 9.7 min (minor), t_{R2} = 10.3 min (major).

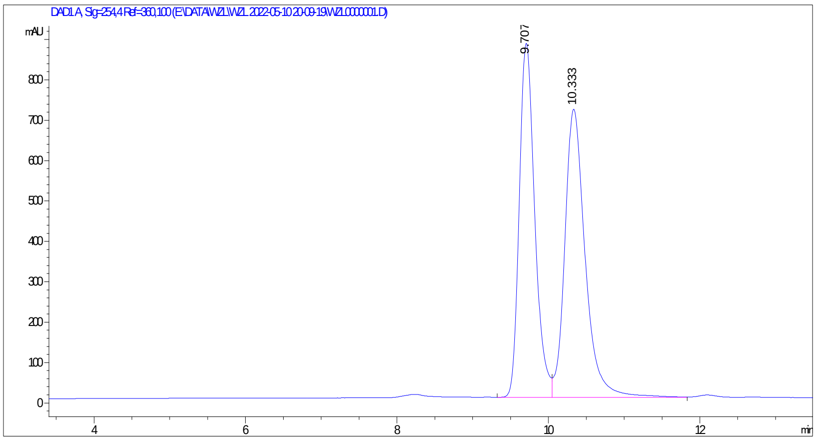

Signal 1: DAD1 A, Sig=254,4 Ref=360,100

Peak #	RetTime [min]	Type	Width [min]	Area [mAU*s]	Height [mAU]	Area %
1	9.707	BV	0.2174	1.24249e4	876.41150	48.2414
2	10.333	VV	0.2821	1.33308e4	713.20087	51.7586

Totals : 2.57556e4 1589.61237

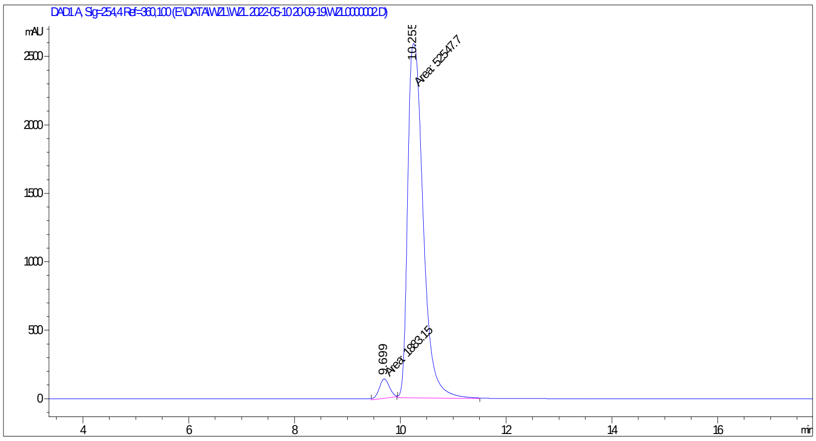

Signal 1: DAD1 A, Sig=254,4 Ref=360,100

Peak #	RetTime [min]	Type	Width [min]	Area [mAU*s]	Height [mAU]	Area %
1	9.699	MM	0.2229	1883.15369	140.83405	3.4597
2	10.255	MM	0.3383	5.25477e4	2588.73877	96.5403

Totals : 5.44308e4 2729.57281

(*R*)-(3,5-Dimethylphenyl)(1-phenylbuta-1,2-dien-1-yl)silane (6ac)

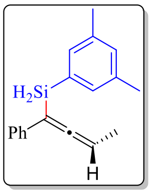

Prepared according to procedure 4 from **1a** (0.2 mmol, 25.6 mg) and **2c** (0.6 mmol, 81.8 mg). The reaction mixture was stirred at -50 °C for 24 h in 2.0 mL dry DCE. The product was isolated in 79% yield with 96% ee value (41.8 mg) as colorless oil.

R*f*: 0.60 (petroleum ether).

HRMS (ESI) (m/z): Calcd for $C_{18}H_{21}Si$ $[M+H]^+$: 265.1413, found: 265.1408.

^{1}H NMR (400 MHz, $CDCl_3$) δ 7.38 (dd, *J* = 8.3, 1.0 Hz, 2H), 7.30 – 7.23 (m, 4H), 7.19 – 7.14 (m, 1H), 7.03 (s, 1H), 5.22 (q, *J* = 7.1 Hz, 1H), 4.86 (d, *J* = 6.8 Hz, 1H), 4.83 (d, *J* = 6.8 Hz, 1H), 2.30 (s, 6H), 1.73 (d, *J* = 7.1 Hz, 3H).

^{13}C NMR (101 MHz, $CDCl_3$) δ 211.9, 137.5, 137.0, 133.3, 131.8, 131.1, 128.7, 127.5, 126.6, 92.7, 83.2, 21.4, 13.3.

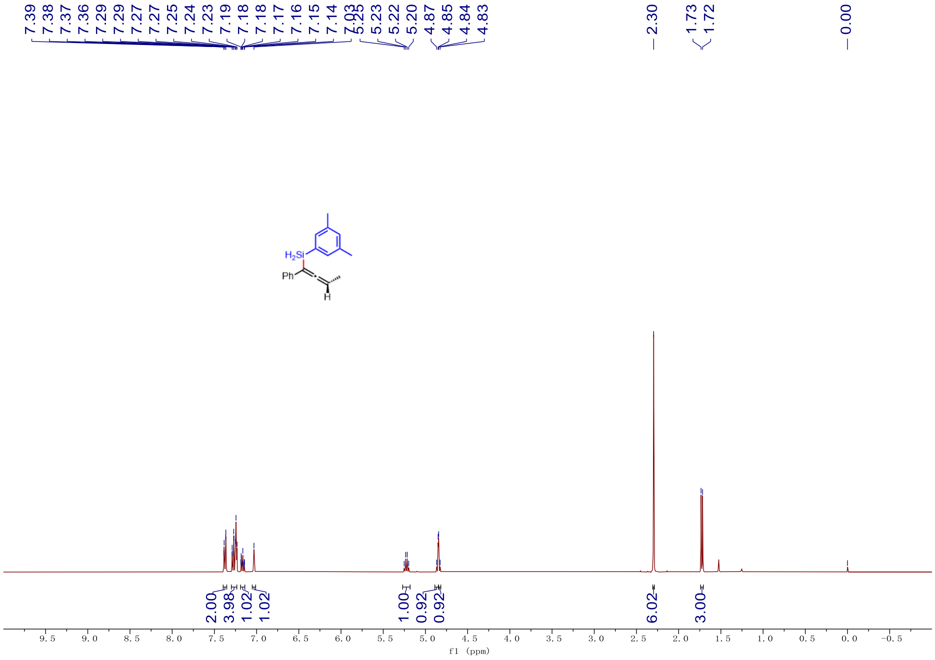

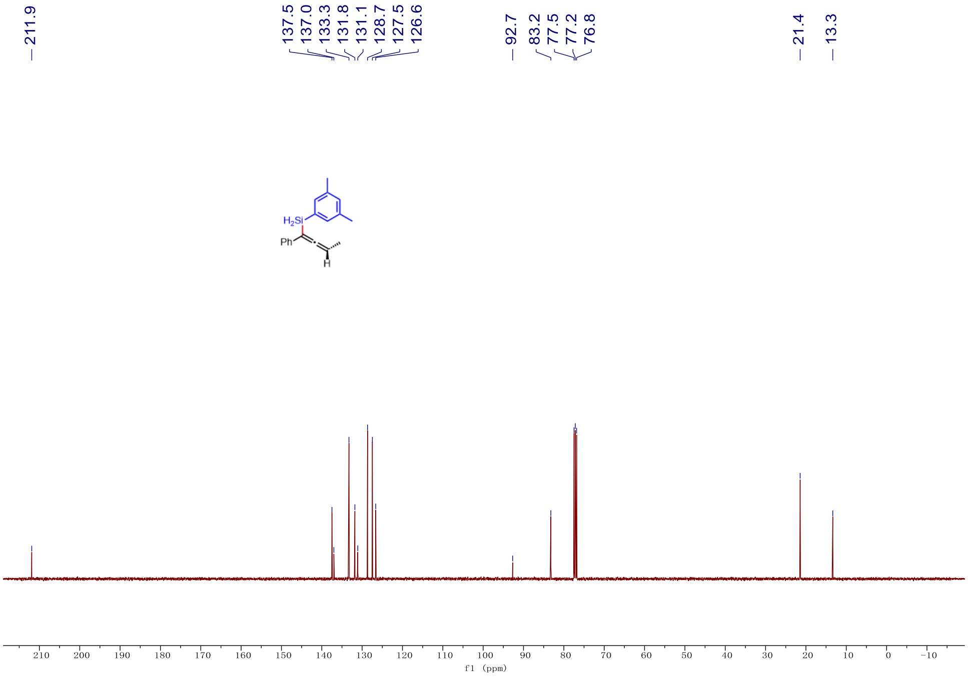

$[\alpha]_D^{20}$ +31.7° (*c* 2.05, ethyl acetate).

The enantiomeric excess of **6ac** was determined by chiral HPLC analysis on Chiralcel OJ-3 column.

Conditions: hexane : isopropanol = 98:2, flow rate = 0.5 mL/min, UV-Vis detection at λ = 254 nm.

t_{R1} = 10.9 min (major), t_{R2} = 12.5 min (minor).

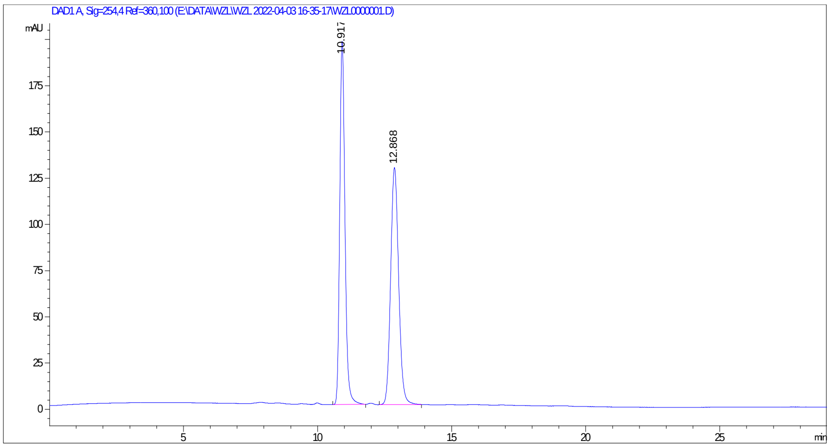

Signal 1: DAD1 A, Sig=254,4 Ref=360,100

Peak #	RetTime [min]	Type	Width [min]	Area [mAU*s]	Height [mAU]	Area %
1	10.917	BB	0.2081	2662.10742	196.44157	50.0708
2	12.868	BB	0.3236	2654.58276	128.19327	49.9292
Totals :				5316.69019	324.63484	

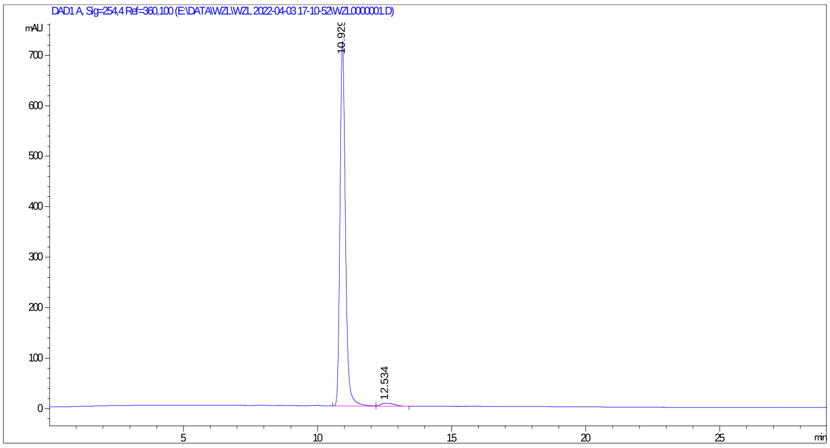

Signal 1: DAD1 A, Sig=254,4 Ref=360,100

Peak #	RetTime [min]	Type	Width [min]	Area [mAU*s]	Height [mAU]	Area %
1	10.929	BV	0.2156	1.01256e4	722.29535	98.1495
2	12.534	VB	0.4115	190.90749	5.63121	1.8505
Totals :				1.03165e4	727.92656	

(*R*)-(4-Fluorophenyl)(1-phenylbuta-1,2-dien-1-yl)silane (6ad)

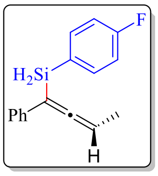

Prepared according to procedure 4 from **1a** (0.2 mmol, 25.6 mg) and **2d** (0.4 mmol, 50.5 mg). The product was isolated in 60% yield with 98% ee value (30.5 mg) as colorless oil.

R_f: 0.58(petroleum ether).

HRMS (EI) (m/z): Calcd for $C_{16}H_{15}FSi$ $[M]^+$: 254.0927, found: 254.0932.

^{1}H NMR (400 MHz, $CDCl_3$) δ 7.64 – 7.55 (m, 2H), 7.39 – 7.32 (m, 2H), 7.32 – 7.22 (m, 2H), 7.22 – 7.13 (m, 1H), 7.05 (tt, *J* = 9.1, 2.3 Hz, 2H), 5.22 (q, *J* = 7.1 Hz, 1H), 4.90 (d, *J* = 6.8 Hz, 1H), 4.87 (d, *J* = 6.8 Hz, 1H), 1.70 (d, *J* = 7.1 Hz, 3H).

^{13}C NMR (101 MHz, $CDCl_3$) δ 212.0, 164.4 (d, *J* = 249.4 Hz), 137.6 (d, *J* = 7.7 Hz), 136.7, 128.7, 127.4, 126.9 (d, *J* = 3.7 Hz), 126.8, 115.5 (d, *J* = 19.9 Hz), 92.5, 83.5, 13.3.

^{19}F NMR (376 MHz, $CDCl_3$) δ -110.4.

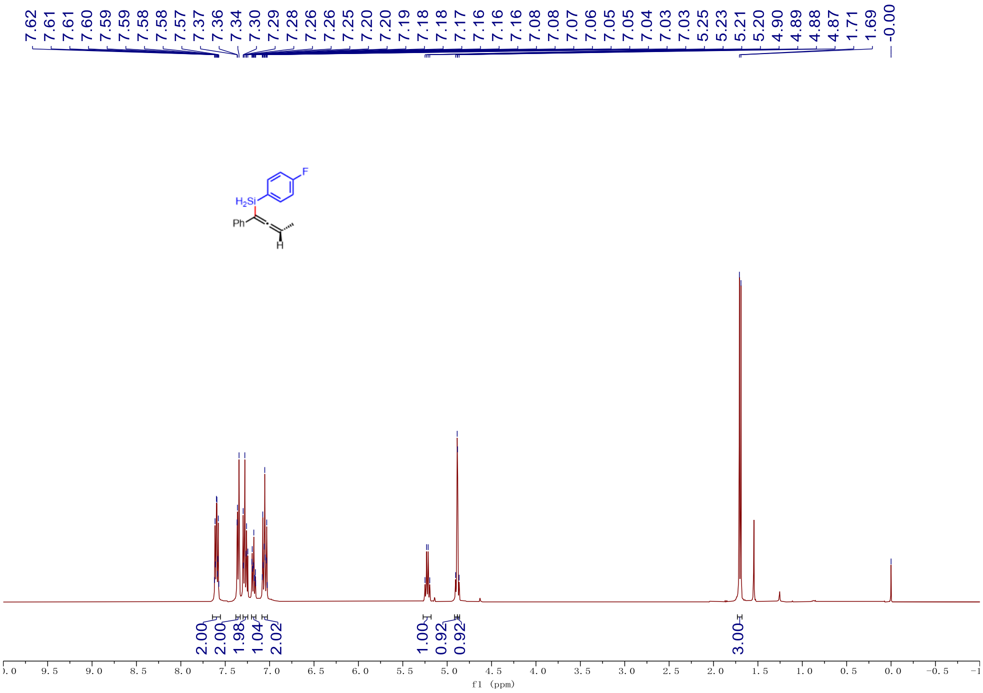

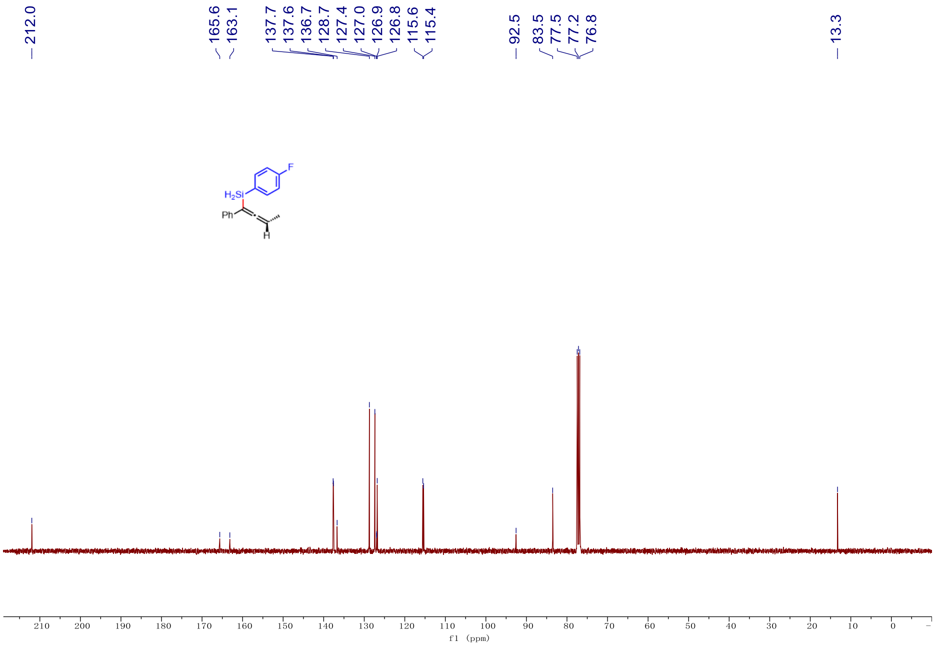
212.0
165.6
163.1
137.7
137.6
136.7
128.7
127.4
127.0
126.9
126.8
115.6
115.4
92.5
83.5
77.5
77.2
76.8
13.3
H2Si
F
Ph
H
f1 (ppm)

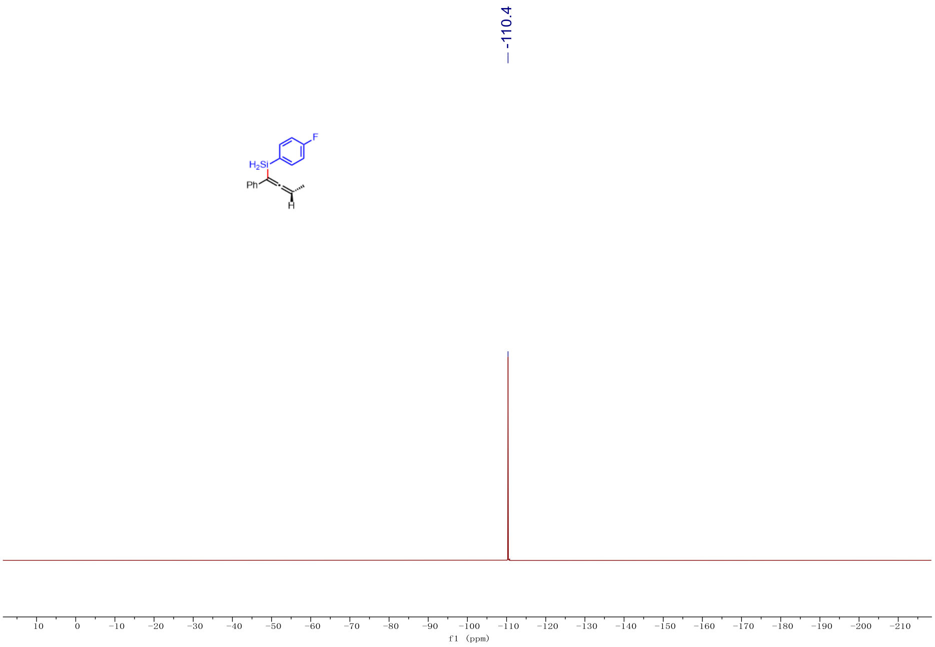
-110.4
H2Si
F
Ph
H
f1 (ppm)

$[\alpha]_D^{20}$ +52.4° (c 1.5, ethyl acetate).
The enantiomeric excess of **6ad** was determined by chiral HPLC analysis on Chiralcel OJ-3 column.
Conditions: hexane : isopropanol = 99:1, flow rate = 0.5 mL/min, UV-Vis detection at λ = 254 nm.
t_{R1} = 13.9 min (major), t_{R2} = 17.5 min (minor).

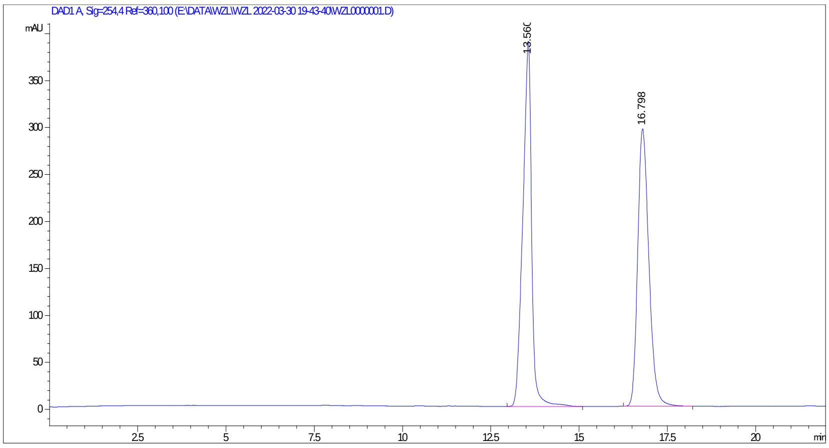

Signal 1: DAD1 A, Sig=254,4 Ref=360,100

Peak #	RetTime [min]	Type	Width [min]	Area [mAU*s]	Height [mAU]	Area %
1	13.560	BB	0.2452	6707.92529	388.95358	50.7694
2	16.798	BB	0.3389	6504.60596	295.36862	49.2306
Totals :				1.32125e4	684.32220	

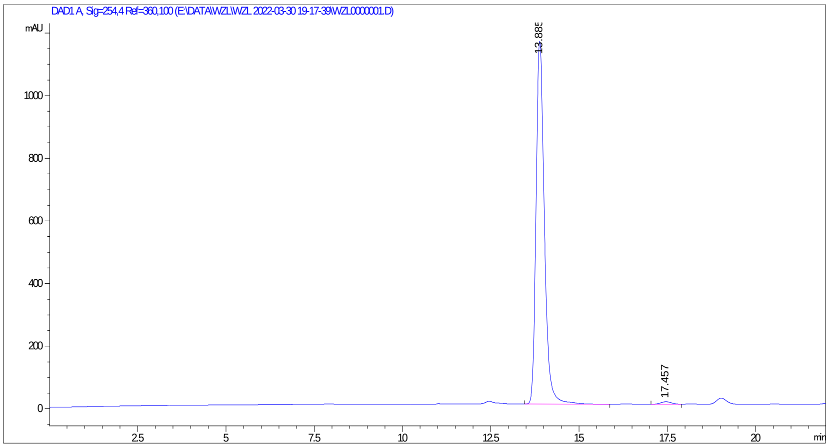

Signal 1: DAD1 A, Sig=254,4 Ref=360,100

Peak #	RetTime [min]	Type	Width [min]	Area [mAU*s]	Height [mAU]	Area %
1	13.885	BB	0.2510	1.89610e4	1158.01025	99.1500
2	17.457	BB	0.3220	162.54800	7.96713	0.8500
Totals :				1.91235e4	1165.97739	

(*R*)-(4-Chlorophenyl)(1-phenylbuta-1,2-dien-1-yl)silane (6ae)

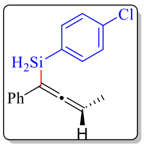

Prepared according to procedure 4 from **1a** (0.2 mmol, 25.6 mg) and **2e** (0.4 mmol, 57.0 mg). The product was isolated in 70% yield with 98% ee value (37.8 mg) as colorless oil.

R*f*: 0.65 (petroleum ether).

HRMS (EI) (m/z): Calcd for $C_{16}H_{15}ClSi$ $[M]^+$: 270.0632, found: 270.0636.

^{1}H NMR (400 MHz, $CDCl_3$) δ 7.56 – 7.52 (m, 2H), 7.36 – 7.31 (m, 4H), 7.30 – 7.25 (m, 2H), 7.21 – 7.15 (m, 1H), 5.23 (q, *J* = 7.1 Hz, 1H), 4.88 (d, *J* = 6.8 Hz, 1H), 4.86 (d, *J* = 6.8 Hz, 1H), 1.70 (d, *J* = 7.2 Hz, 3H).

^{13}C NMR (101 MHz, $CDCl_3$) δ 212.0, 136.9, 136.6, 136.5, 129.8, 128.8, 128.4, 127.4, 126.8, 92.3, 83.6, 13.3.

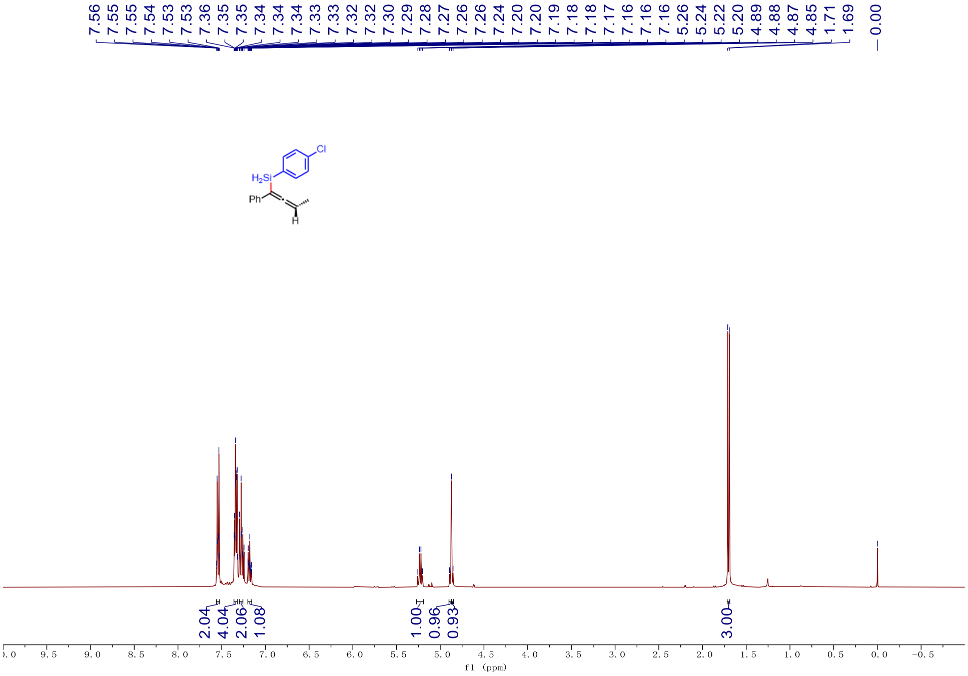

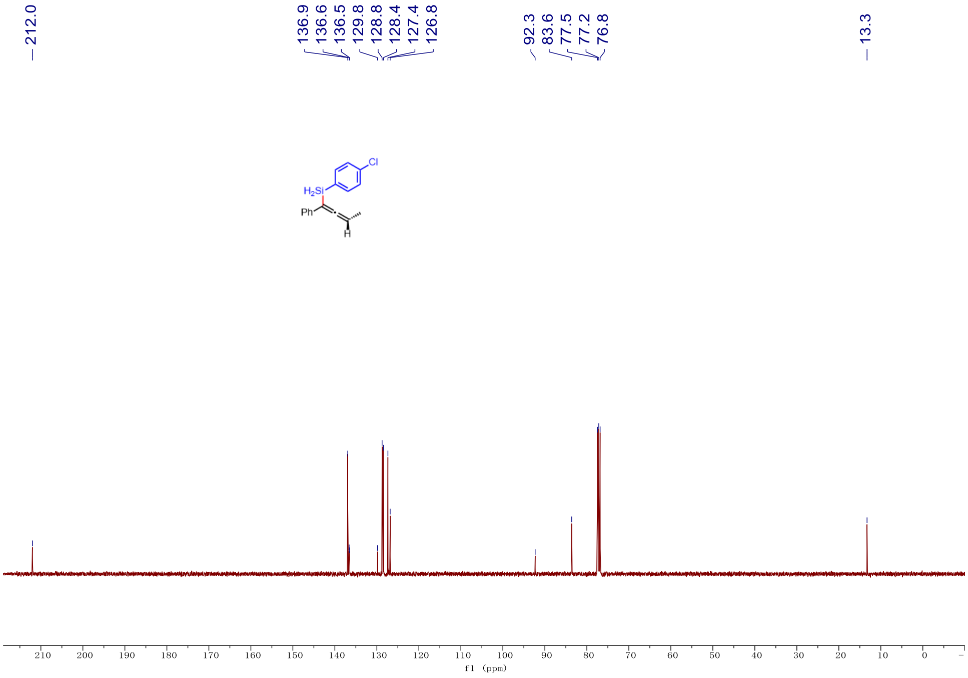

$[\alpha]_D^{20}$ +43.8° (*c* 1.85, ethyl acetate).

The enantiomeric excess of **6ae** was determined by chiral HPLC analysis on Chiralcel OJ-3 column.

Conditions: hexane : isopropanol = 99:1, flow rate = 0.5 mL/min, UV-Vis detection at λ = 254 nm.

t_{R1} = 13.1 min (major), t_{R2} = 16.4 min (minor).

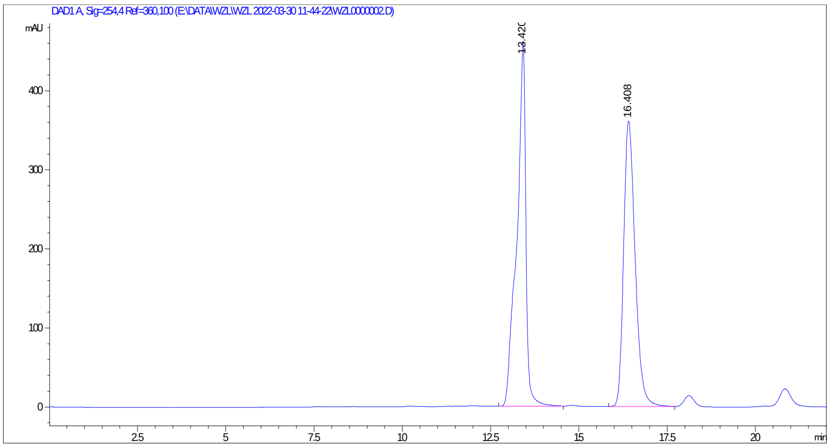

Signal 1: DAD1 A, Sig=254,4 Ref=360,100

Peak #	RetTime [min]	Type	Width [min]	Area [mAU*s]	Height [mAU]	Area %
1	13.420	BB	0.2455	8207.68457	461.52078	50.1576
2	16.408	BB	0.3477	8156.11035	360.86505	49.8424

Totals : 1.63638e4 822.38583

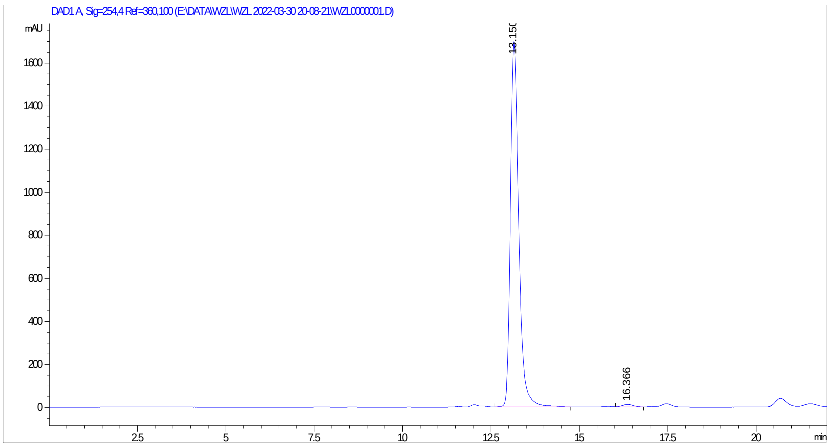

Signal 1: DAD1 A, Sig=254,4 Ref=360,100

Peak #	RetTime [min]	Type	Width [min]	Area [mAU*s]	Height [mAU]	Area %
1	13.150	BB	0.2560	2.82026e4	1696.10010	99.1158
2	16.366	VB	0.3194	251.60243	11.96073	0.8842

Totals : 2.84542e4 1708.06083

(*R*)-(1-Phenylbuta-1,2-dien-1-yl)(4-(trifluoromethyl)phenyl)silane (6af)

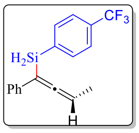

Prepared according to procedure 4 from **1a** (0.2 mmol, 25.6 mg) and **2f** (0.6 mmol, 105.7 mg). The reaction mixture was stirred at -50 °C for 24 h in 2.0 mL dry DCE. The product was isolated in 80% yield with 98% ee value (48.7 mg) as colorless oil.

R*f*: 0.60 (petroleum ether).

HRMS (EI) (m/z): Calcd for $C_{17}H_{15}F_3Si$ $[M]^+$: 304.0895, found: 304.0900.

^{1}H NMR (400 MHz, $CDCl_3$) δ 7.77 – 7.71 (m, 2H), 7.60 (dd, *J* = 7.5, 0.8 Hz, 2H), 7.39 – 7.33 (m, 2H), 7.32 – 7.26 (m, 2H), 7.22 – 7.16 (m, 1H), 5.29 (q, *J* = 7.2 Hz, 1H), 4.93 (d, *J* = 6.8 Hz, 1H), 4.91 (d, *J* = 6.8 Hz, 1H), 1.70 (d, *J* = 7.1 Hz, 3H).

^{13}C NMR (101 MHz, $CDCl_3$) δ 212.2, 136.7, 136.4, 135.9, 131.9 (q, *J* = 32.2 Hz), 128.8, 127.3, 126.9, 124.7 (q, *J* = 3.7 Hz), 124.2 (q, *J* = 272.3 Hz), 92.0, 83.8, 13.2.

^{19}F NMR (376 MHz, $CDCl_3$) δ -63.0.

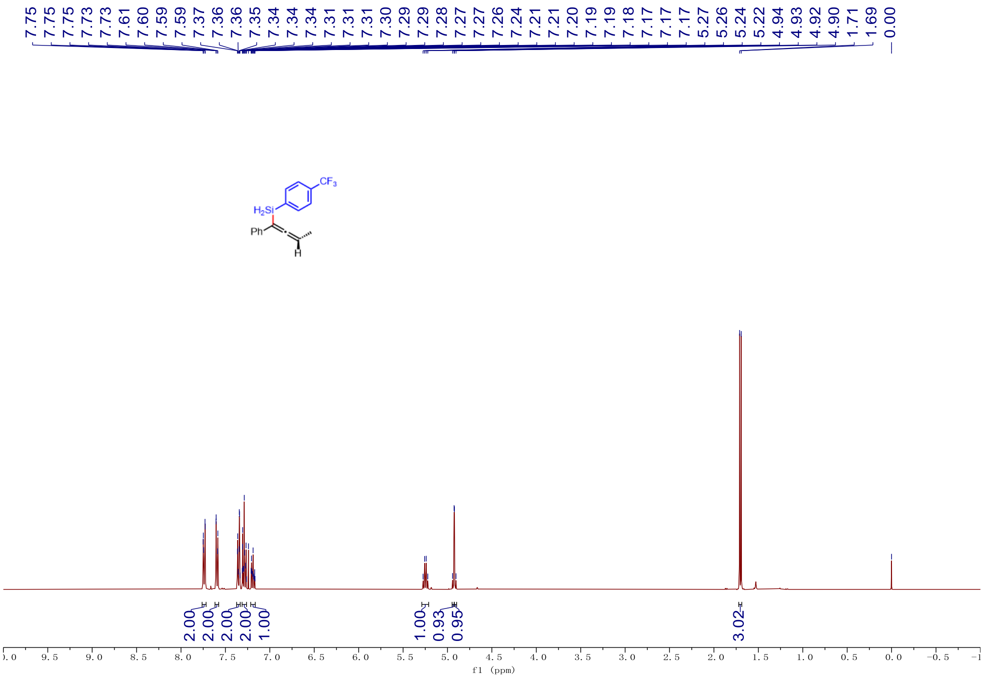

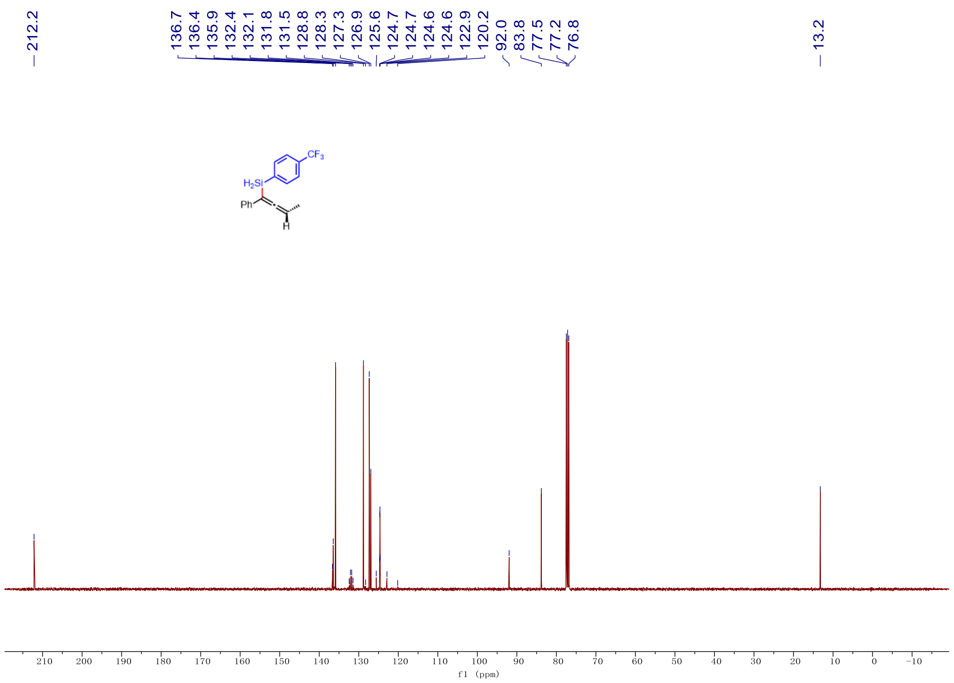
212.2
136.7
136.4
135.9
132.4
132.1
131.8
131.5
128.8
128.3
127.3
126.9
125.6
124.7
124.7
124.6
124.6
122.9
120.2
92.0
83.8
77.5
77.2
76.8
13.2
CF3
H2Si
Ph
H
f1 (ppm)

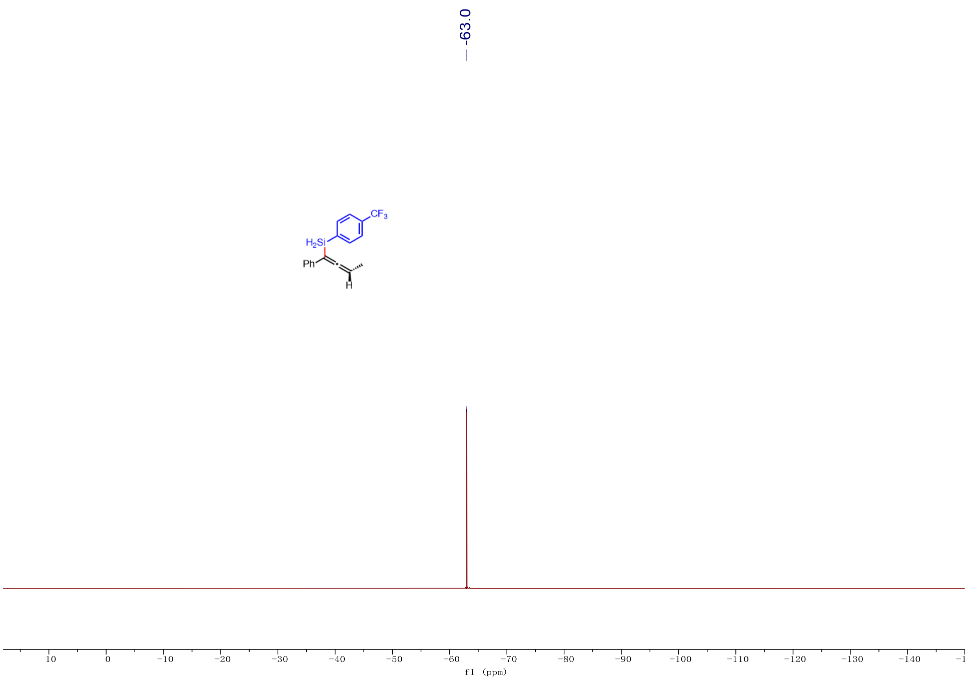
-63.0
CF3
H2Si
Ph
H
f1 (ppm)

$[\alpha]_D^{20}$ +39.3° (*c* 2.4, ethyl acetate).

The enantiomeric excess of **6af** was determined by chiral HPLC analysis on Chiralcel OJ-3 column.

Conditions: hexane : isopropanol = 98:2, flow rate = 0.5 mL/min, UV-Vis detection at λ = 254 nm.

t_{R1} = 10.3min (major), t_{R2} = 12.3 min (minor).

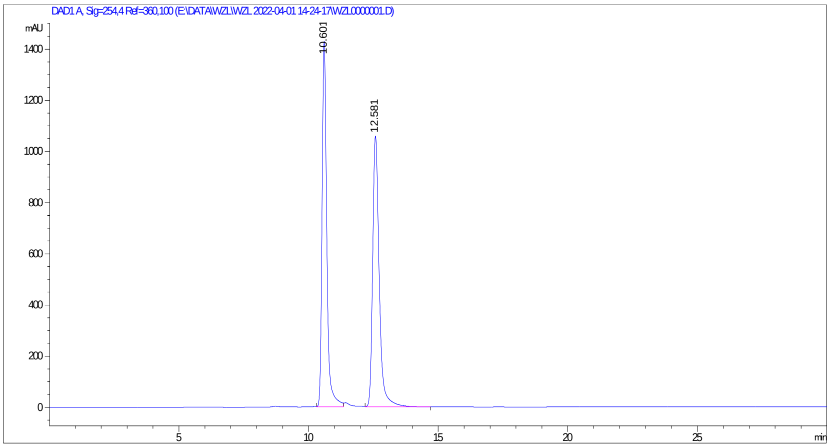

Signal 1: DAD1 A, Sig=254,4 Ref=360,100

Peak #	RetTime [min]	Type	Width [min]	Area [mAU*s]	Height [mAU]	Area %
1	10.601	VV	0.1946	1.82079e4	1429.85803	49.4729
2	12.581	VB	0.2669	1.85959e4	1058.70264	50.5271

Totals : 3.68038e4 2488.56067

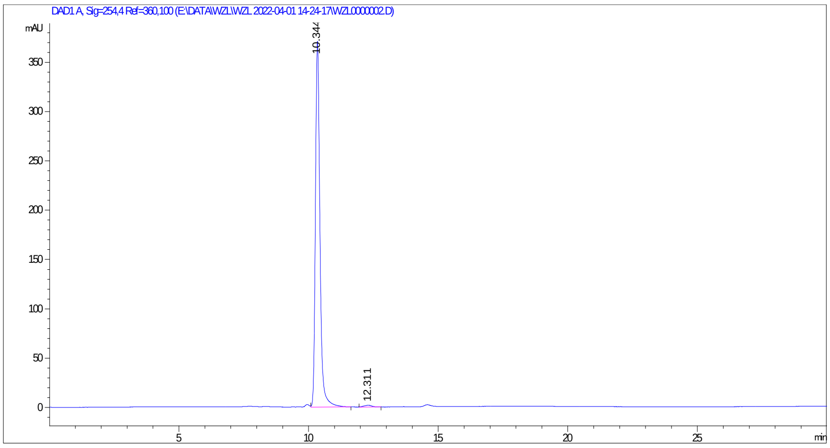

Signal 1: DAD1 A, Sig=254,4 Ref=360,100

Peak #	RetTime [min]	Type	Width [min]	Area [mAU*s]	Height [mAU]	Area %
1	10.344	VB	0.1835	4500.63916	370.95486	99.1753
2	12.311	BB	0.2524	37.42366	1.81654	0.8247

Totals : 4538.06282 372.77141

(*R*)-(1-([1,1'-biphenyl]-4-yl)buta-1,2-dien-1-yl)(naphthalen-2-yl)silane (6ei)

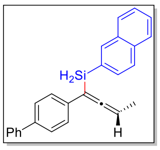

Prepared according to procedure 4 from **1e** (0.2 mmol, 25.6 mg) and **2i** (0.6 mmol, 105.7 mg). The product was isolated in 57% yield with 91% ee value (41.3 mg) as colorless oil.

R*f*: 0.32 (petroleum ether).

HRMS (EI) (m/z): Calcd for $C_{26}H_{22}Si$ $[M]^+$: 362.1491, found:362.1481.

^{1}H NMR (400 MHz, $CDCl_3$) δ 8.21 (s, 1H), 7.88 – 7.81 (m, 3H), 7.70 (d, *J* = 8.4 Hz, 1H), 7.60 – 7.54 (m, 2H), 7.56 – 7.45 (m, 6H), 7.42 (dd, *J* = 8.4, 6.9 Hz, 2H), 7.37 – 7.29 (m, 1H), 5.29 (q, *J* = 7.2 Hz, 1H), 5.07 (d, *J* = 6.8 Hz, 1H), 5.05 (d, *J* = 6.8 Hz, 1H), 1.75 (d, *J* = 7.2 Hz, 3H).

^{13}C NMR (101 MHz, $CDCl_3$) δ 212.2, 140.9, 139.6, 136.9, 135.9, 134.2, 133.1, 131.2, 128.9, 128.9, 128.3, 127.9, 127.8, 127.4, 127.3, 127.1, 126.9, 126.2, 92.2, 83.6, 13.4.

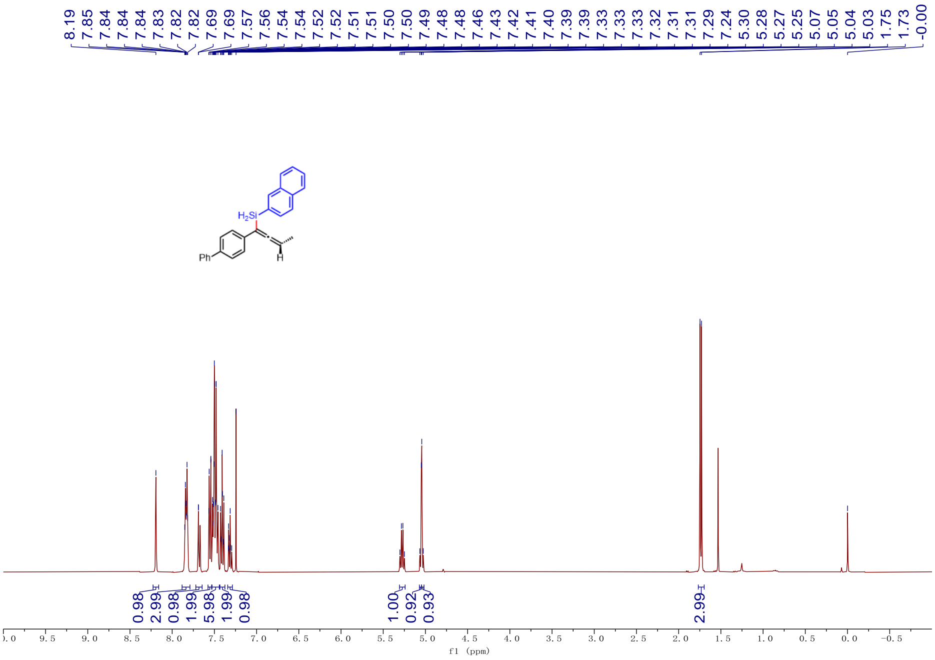

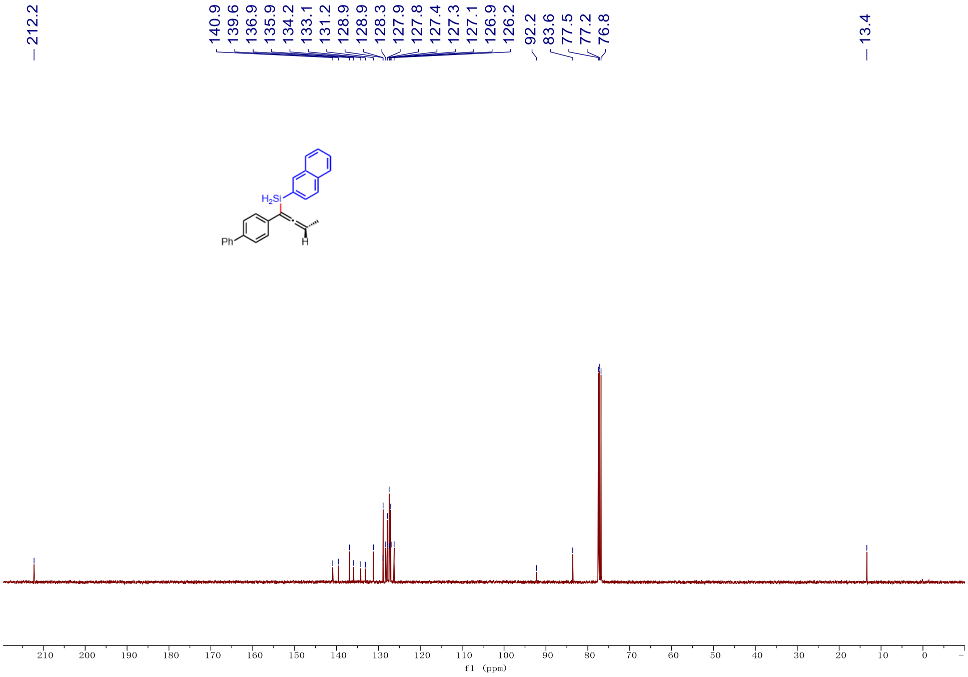

$[\alpha]_D^{20}$ +89.5° (*c* 1.5, ethyl acetate).

The enantiomeric excess of **6ei** was determined by chiral HPLC analysis on Chiralcel OJ-3 column.

Conditions: hexane : isopropanol = 70:30, flow rate = 0.45 mL/min, UV-Vis detection at λ = 254 nm.

t_{R1} = 40.3 min (major), t_{R2} = 86.7 min (minor).

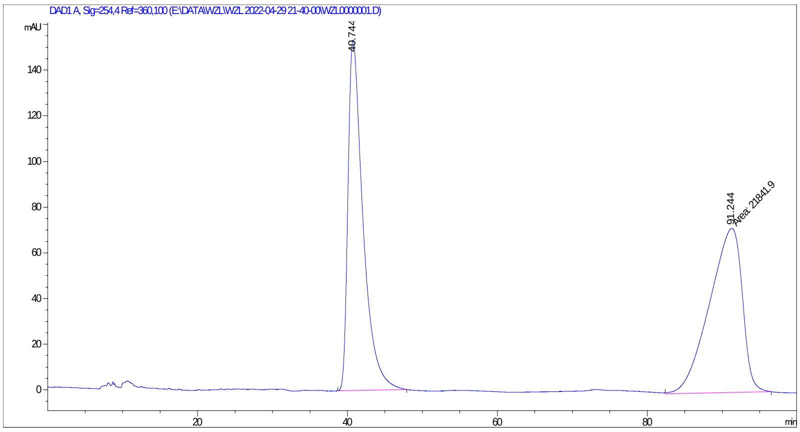

Signal 1: DAD1 A, Sig=254,4 Ref=360,100

Peak #	RetTime [min]	Type	Width [min]	Area [mAU*s]	Height [mAU]	Area %
1	40.744	BB	1.9410	2.13728e4	153.44785	49.4572
2	91.244	MM	5.0673	2.18419e4	71.83896	50.5428

Totals : 4.32147e4 225.28680

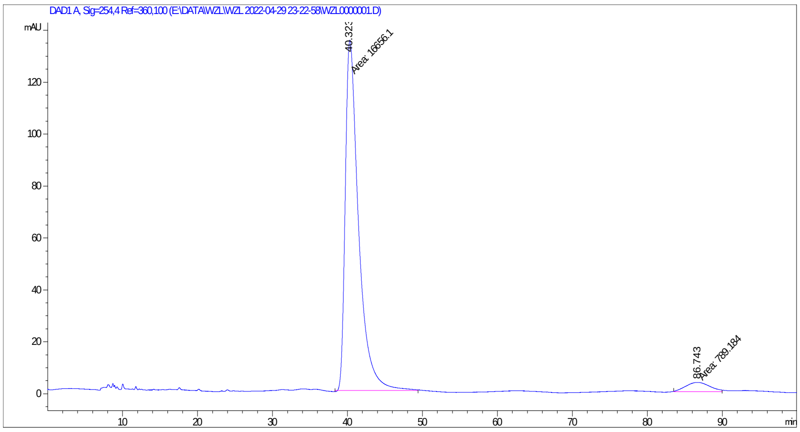

Signal 1: DAD1 A, Sig=254,4 Ref=360,100

Peak #	RetTime [min]	Type	Width [min]	Area [mAU*s]	Height [mAU]	Area %
1	40.323	MM	2.0567	1.66561e4	134.97601	95.4762
2	86.743	MM	3.6917	789.18384	3.56284	4.5238

Totals : 1.74452e4 138.53886

(*R*)-Dimethoxy(phenyl)(1-phenylbuta-1,2-dien-1-yl)silane (10)

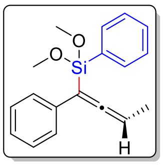

Prepared according to procedure 10 from **6aa** (0.2 mmol, 47.3 mg). The product was isolated in 77% yield with 96% ee value (45.8 mg) as colorless oil.

R_f: 0.45 (ethyl acetate : petroleum ether = 1:20).

HRMS (EI) (m/z): Calcd for $C_{18}H_{20}O_2Si$ $[M]^+$: 296.1233, found: 296.1232.

^{1}H NMR (400 MHz, $CDCl_3$) δ 7.69 – 7.65 (m, 2H), 7.45 (d, *J* = 8.0 Hz, 2H), 7.42 – 7.38 (m, 1H), 7.38 – 7.33 (m, 2H), 7.28 – 7.24 (m, 2H), 7.16 (tt, *J* = 7.2, 1.6 Hz, 1H), 5.26 (q, *J* = 7.2 Hz, 1H), 3.63 (s, 3H), 3.62 (s, 3H), 1.71 (d, *J* = 7.2 Hz, 3H).

^{13}C NMR (101 MHz, $CDCl_3$) δ 213.0, 136.5, 134.6, 132.8, 130.4, 128.6, 127.9, 127.9, 126.6, 94.6, 84.1, 51.4, 51.4, 13.3.

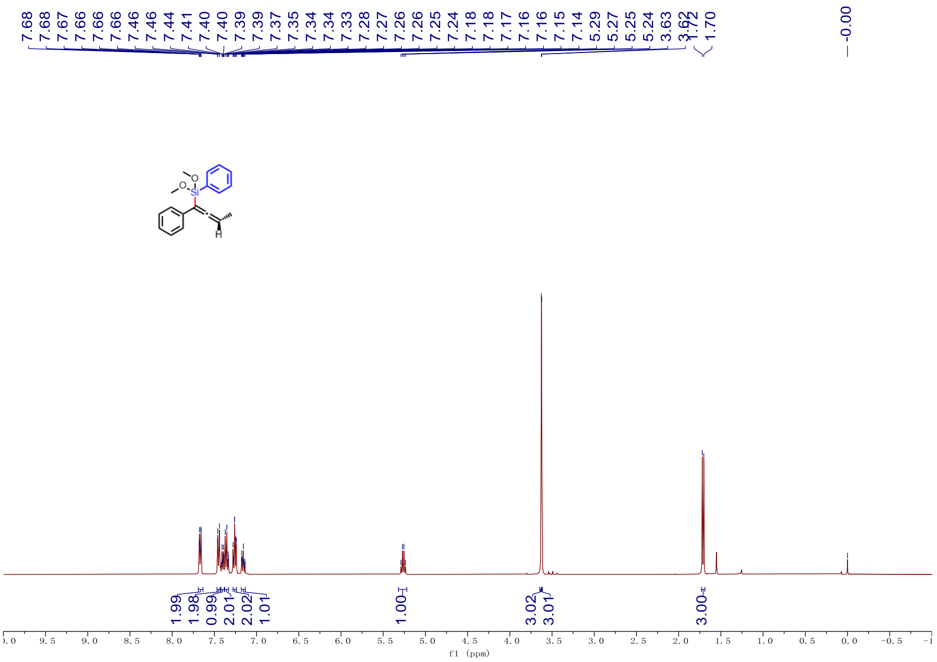

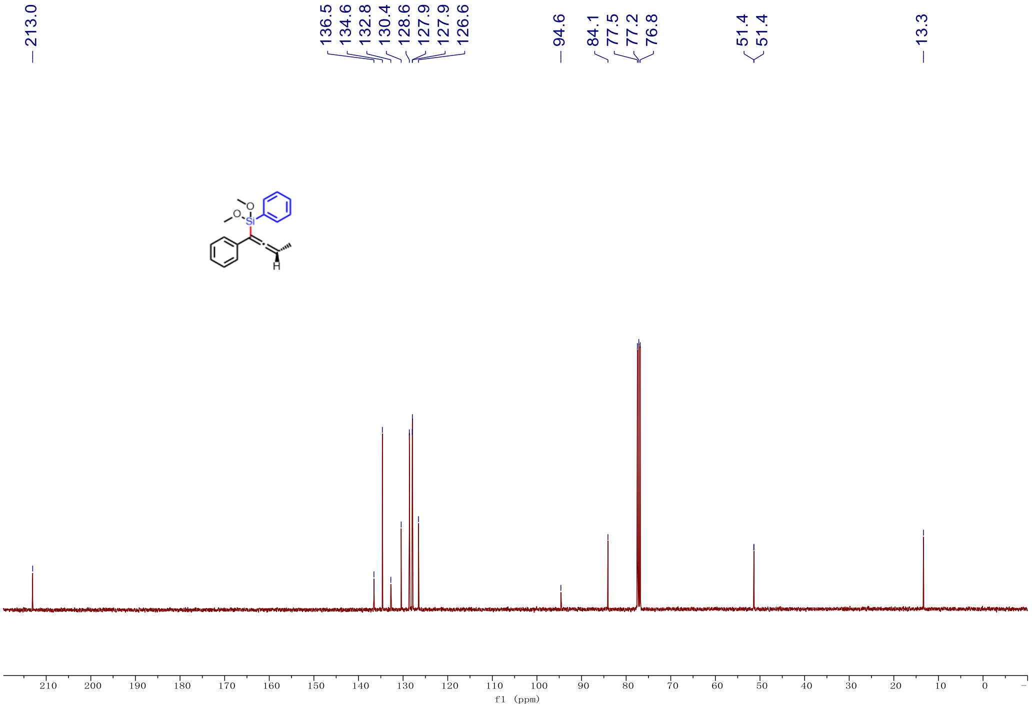

$[\alpha]_D^{20}$ +23.7° (c 2.25, ethyl acetate).

The enantiomeric excess of **10** was determined by chiral HPLC analysis on Chiralcel OJ-3 column.

Conditions: hexane : isopropanol = 98:2, flow rate = 0.5 mL/min, UV-Vis detection at λ = 254 nm.

t_{R1} = 14.4 min (minor), t_{R2} = 15.4 min (major).

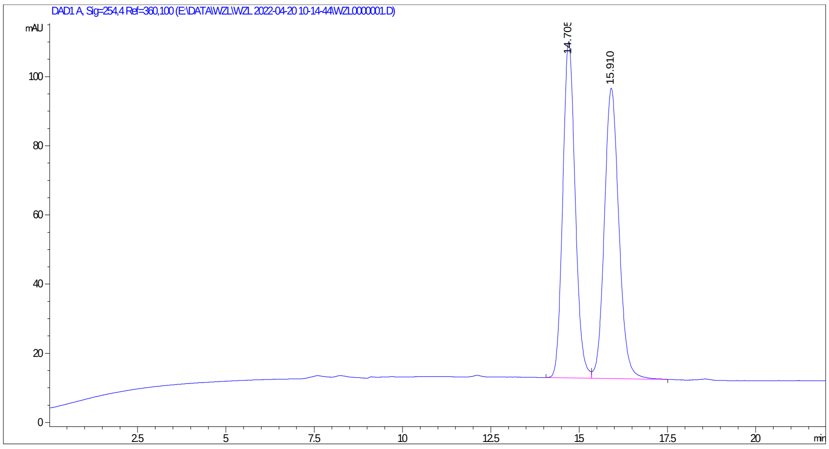

Signal 1: DAD1 A, Sig=254,4 Ref=360,100

Peak #	RetTime [min]	Type	Width [min]	Area [mAU*s]	Height [mAU]	Area %
1	14.705	BV	0.3794	2379.98877	97.28748	49.6856
2	15.910	VB	0.4429	2410.11304	83.96436	50.3144

Totals : 4790.10181 181.25184

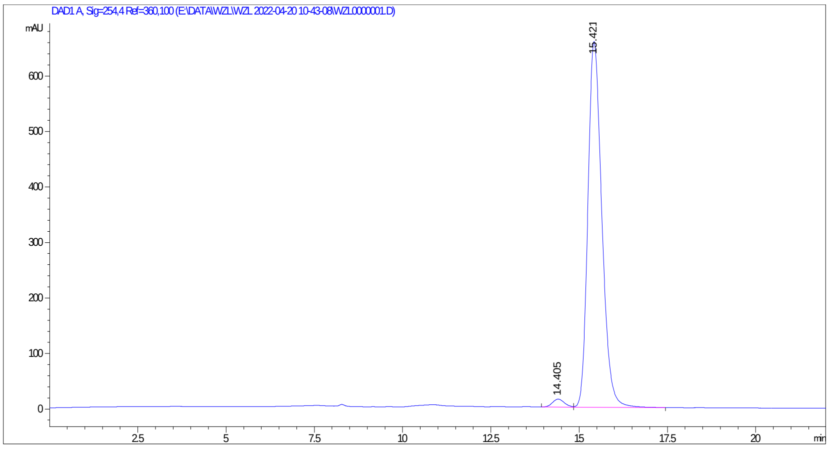

Signal 1: DAD1 A, Sig=254,4 Ref=360,100

Peak #	RetTime [min]	Type	Width [min]	Area [mAU*s]	Height [mAU]	Area %
1	14.405	BV	0.3528	335.35742	14.34383	1.8238
2	15.421	VB	0.4256	1.80527e4	658.95917	98.1762

Totals : 1.83881e4 673.30300

(*R*)-Phenyl(1-phenylbuta-1,2-dien-1-yl)silanediol (11)

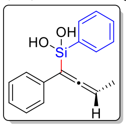

Prepared according to procedure 11 from **6aa** (0.2 mmol, 47.3 mg). The product was isolated in 55% yield with 97% ee value (29.6 mg) as colorless oil.

R*f*: 0.40 (ethyl acetate : petroleum ether = 2:1).

HRMS (EI) (m/z): Calcd for $C_{16}H_{16}O_2Si$ $[M]^+$: 268.0920, found: 268.0921.

^{1}H NMR (400 MHz, $CDCl_3$) δ 7.73 (tt, *J* = 6.4, 1.6 Hz, 2H), 7.47 – 7.39 (m, 3H), 7.41 – 7.33 (m, 2H), 7.26 (tt, *J* = 7.2, 1.6 Hz, 2H), 7.17 (tt, *J* = 7.2, 1.6 Hz, 1H), 5.32 (q, *J* = 7.2 Hz, 1H), 3.08 (s, 2H), 1.74 (d, *J* = 7.2 Hz, 3H).

^{13}C NMR (101 MHz, $CDCl_3$) δ 211.8, 136.0, 134.4, 134.3, 130.6, 128.8, 128.1, 128.0, 126.8, 97.1, 84.7, 13.4.

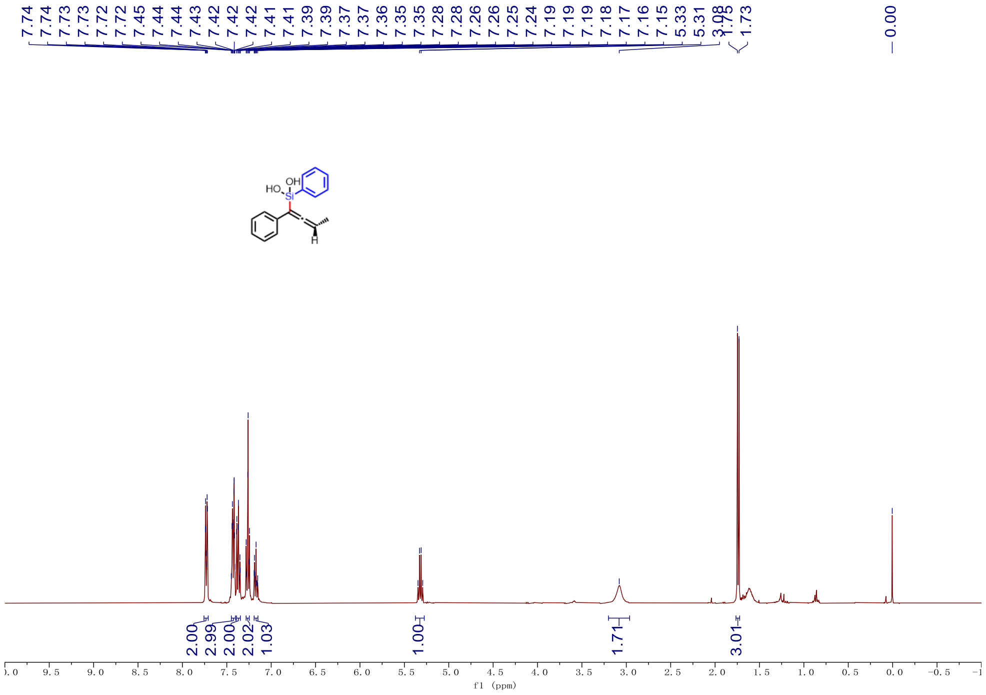

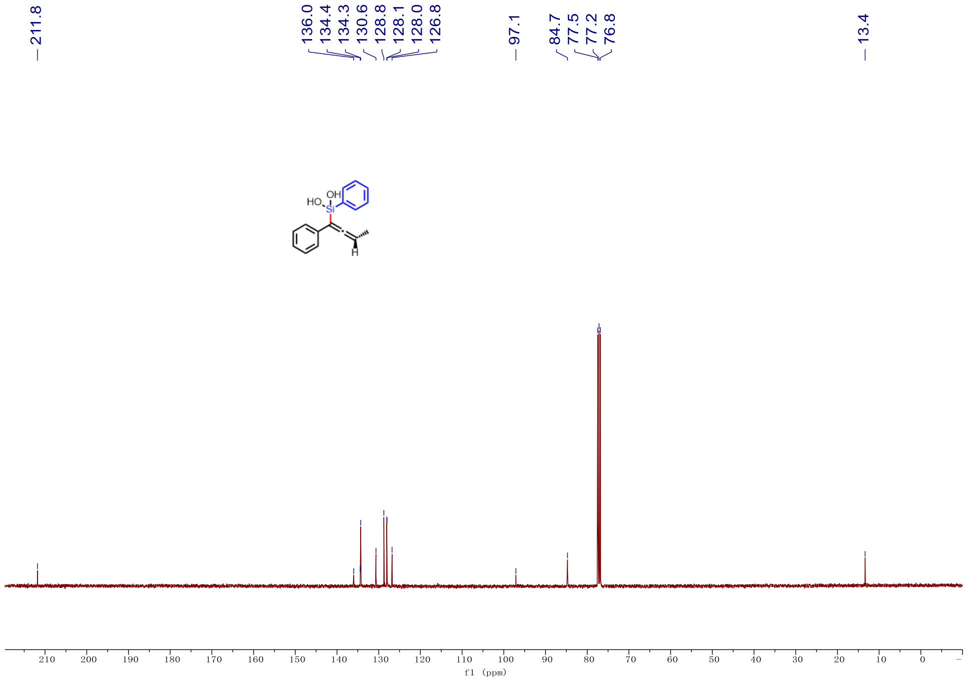

$[\alpha]_D^{20}$ +5.0° (c 1.45, ethyl acetate).

The enantiomeric excess of **11** was determined by chiral HPLC analysis on Chiralcel OJ-3 column.

Conditions: hexane : isopropanol = 90:10, flow rate = 0.5 mL/min, UV-Vis detection at λ = 254 nm.

t_{R1} = 28.1 min (major), t_{R2} = 30.2 min (minor).

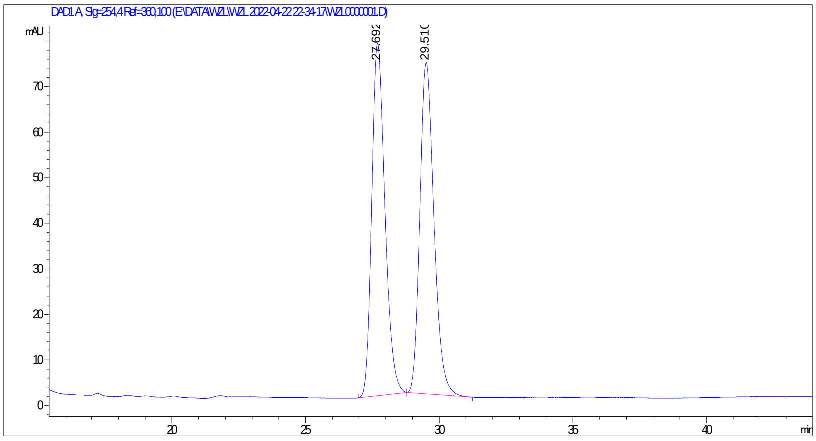

Signal 1: DAD1 A, Sig=254,4 Ref=360,100

Peak #	RetTime [min]	Type	Width [min]	Area [mAU*s]	Height [mAU]	Area %
1	27.692	BB	0.5297	2650.74048	77.37451	49.8295
2	29.510	BB	0.5618	2668.87964	72.77712	50.1705

Totals : 5319.62012 150.15163

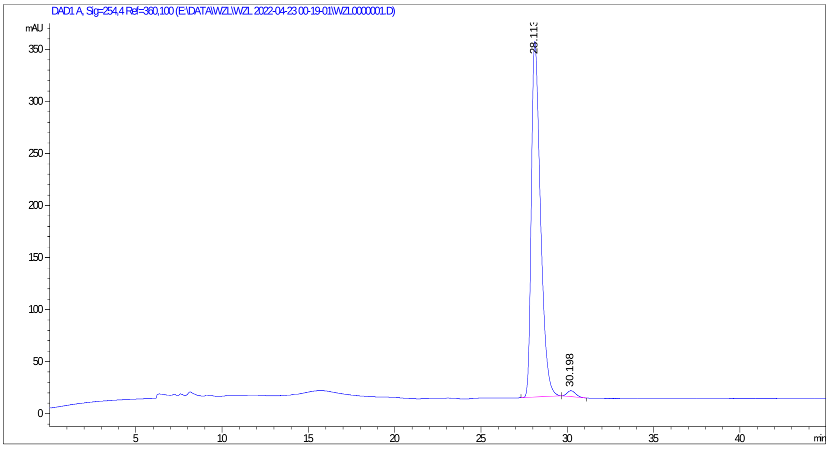

Signal 1: DAD1 A, Sig=254,4 Ref=360,100

Peak #	RetTime [min]	Type	Width [min]	Area [mAU*s]	Height [mAU]	Area %
1	28.113	BB	0.5593	1.25765e4	341.79681	98.4543
2	30.198	BB	0.4776	197.44060	5.70787	1.5457

Totals : 1.27739e4 347.50468

Cinnamyl(phenyl)((*R*)-1-phenylbuta-1,2-dien-1-yl)silane (12)

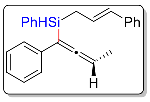

Prepared according to procedure 12 from **6aa** (0.15 mmol, 35.5 mg). The product was isolated in 79% yield and 48:52 *dr* value with 93%, 92% ee value (28.0 mg) as colorless oil.

R*f*: 0.30 (petroleum ether).

HRMS (ESI) (m/z): Calcd for $C_{25}H_{25}Si$ $[M+H]^+$: 353.1726, found: 353.1750.

^{1}H NMR (400 MHz, $CDCl_3$) δ 7.64 – 7.59 (m, 2H), 7.41 – 7.31 (m, 6H), 7.27 – 7.21 (m, 5H), 7.15 (t, *J* = 6.5 Hz, 2H), 6.33 – 6.18 (m, 2H), 5.29 (p, *J* = 6.9 Hz, 1H), 4.96 – 4.88 (m, 1H), 2.23 – 2.11 (m, 2H), 1.75 (d, *J* = 7.2 Hz, 3H).

^{13}C NMR (101 MHz, $CDCl_3$) δ 211.3, 211.3, 138.3, 138.3, 137.1, 137.1, 135.1, 133.8, 133.8, 130.2, 130.2, 129.9, 129.9, 128.6, 128.5, 128.1, 128.1, 127.7, 126.6, 126.2, 126.2, 125.8, 94.6, 94.5, 83.7, 83.7, 19.5, 19.4, 13.6, 13.5.

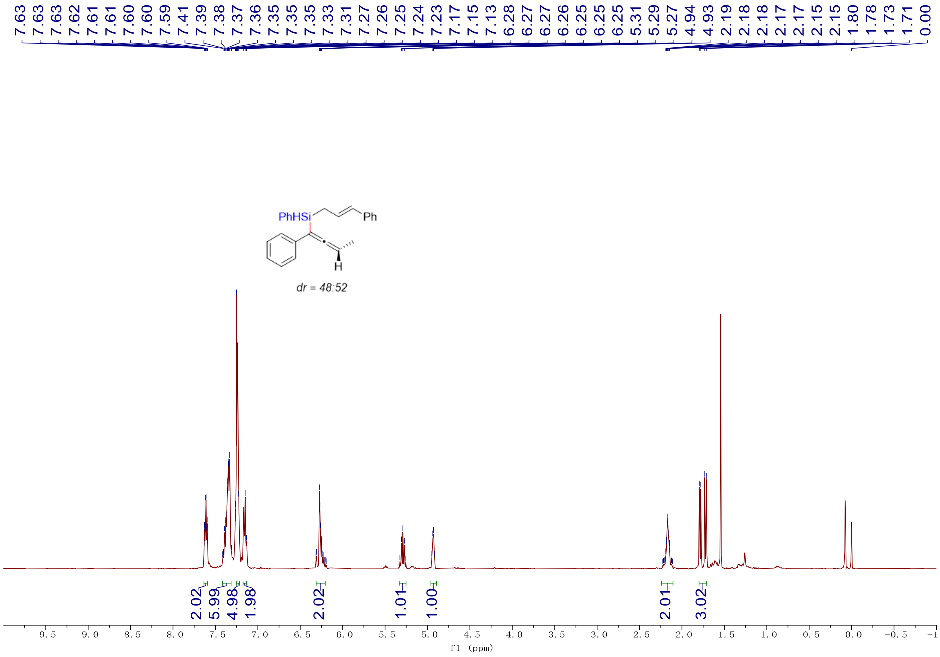

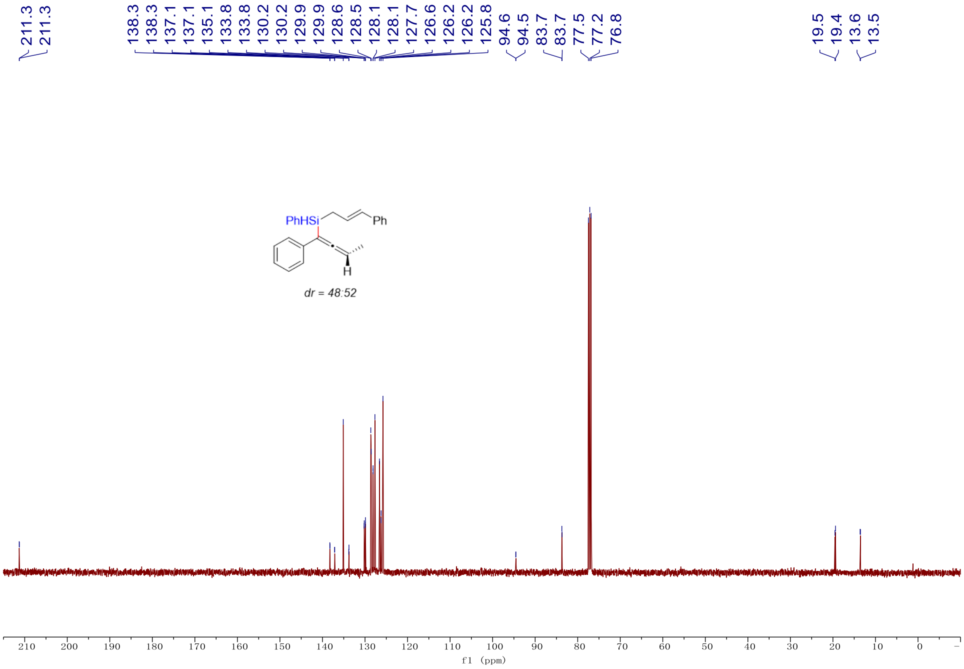

$[\alpha]_D^{20}$ +21° (*c* 1.35, ethyl acetate).
The enantiomeric excess of **12** was determined by chiral HPLC analysis on Chiralcel OD-3 column.
Conditions: hexane, flow rate = 0.5 mL/min, UV-Vis detection at λ = 254 nm.
t_{R1} = 36.7 min (major), t_{R2} = 47.7 min (one isomer).

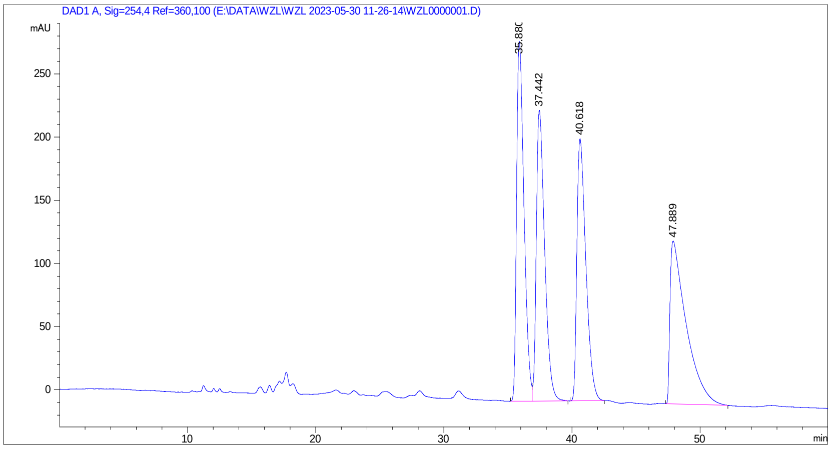

Signal 1: DAD1 A, Sig=254,4 Ref=360,100

Peak #	RetTime [min]	Type	Width [min]	Area [mAU*s]	Height [mAU]	Area %
1	35.880	BV	0.6042	1.14285e4	284.75943	26.3986
2	37.442	VB	0.6614	1.04058e4	230.19209	24.0363
3	40.618	BB	0.7126	1.01147e4	207.53165	23.3638
4	47.889	BB	1.1518	1.13431e4	129.00601	26.2012
Totals :				4.32922e4	851.48918	

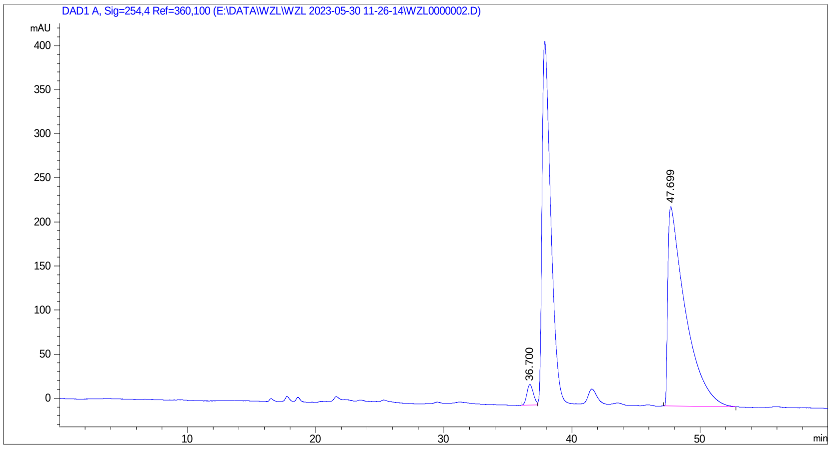

Signal 1: DAD1 A, Sig=254,4 Ref=360,100

Peak #	RetTime [min]	Type	Width [min]	Area [mAU*s]	Height [mAU]	Area %
1	36.700	BV	0.4670	827.43134	23.37611	3.6957
2	47.699	BB	1.2417	2.15618e4	226.27592	96.3043
Totals :				2.23892e4	249.65203	

t_{R1} = 37.8 min (major), t_{R2} = 41.5 min (another isomer)

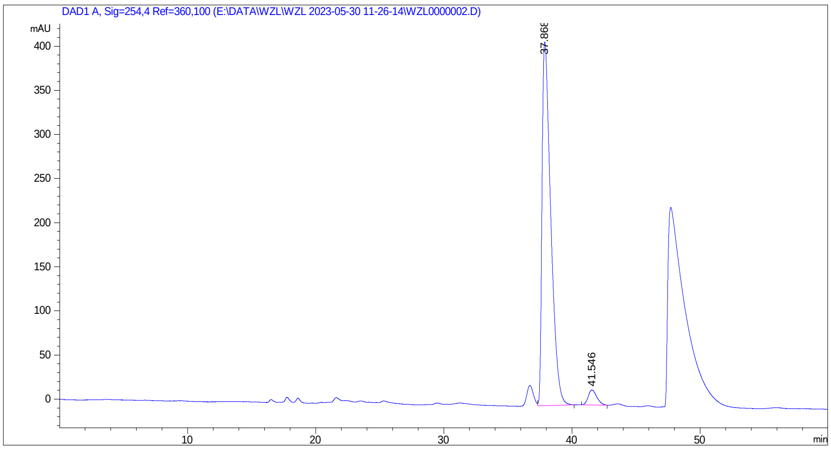

Signal 1: DAD1 A, Sig=254,4 Ref=360,100

Peak #	RetTime [min]	Type	Width [min]	Area [mAU*s]	Height [mAU]	Area %
1	37.868	VB	0.7070	1.94649e4	412.13058	96.1082
2	41.546	BB	0.5919	788.20435	17.09640	3.8918

Totals : 2.02531e4 429.22698

(4-Methoxyphenyl)(1-phenylbuta-1,2-dien-1-yl-4-d)silane-*d*$_2$ (*d*-3ag)

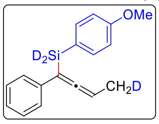

Prepared according to procedure 2 from **1a** (0.2 mmol, 25.6 mg) and ***d*-2g** (0.6 mmol, 84.6 mg). The product was isolated in 65% yield (35.1 mg) as colorless oil.

R$_f$: 0.30 (ethyl acetate : petroleum ether = 1:100).

HRMS (ESI) (m/z): Calcd for $C_{17}H_{16}D_3OSi$ $[M+H]^+$: 270.1393, found: 270.1412.

^{1}H NMR (400 MHz, $CDCl_3$) δ 7.56 (dt, *J* = 8.8, 2.0 Hz, 2H), 7.39 – 7.36 (m, 2H), 7.30 – 7.26 (m, 2H), 7.18 (tt, *J* = 8.0, 1.6 Hz, 1H), 6.92 (dt, *J* = 8.8, 2.0 Hz, 2H), 5.22 (t, *J* = 7.0 Hz, 1H), 3.82 (s, 3H), 1.71 (dt, *J* = 7.9, 2.0 Hz, 2H).

^{13}C NMR (101 MHz, $CDCl_3$) δ 211.9, 161.2, 137.1, 137.0, 128.7, 127.4, 126.6, 122.0, 114.0, 92.8, 83.1, 55.2, 13.1 (t, *J* = 19.5 Hz).

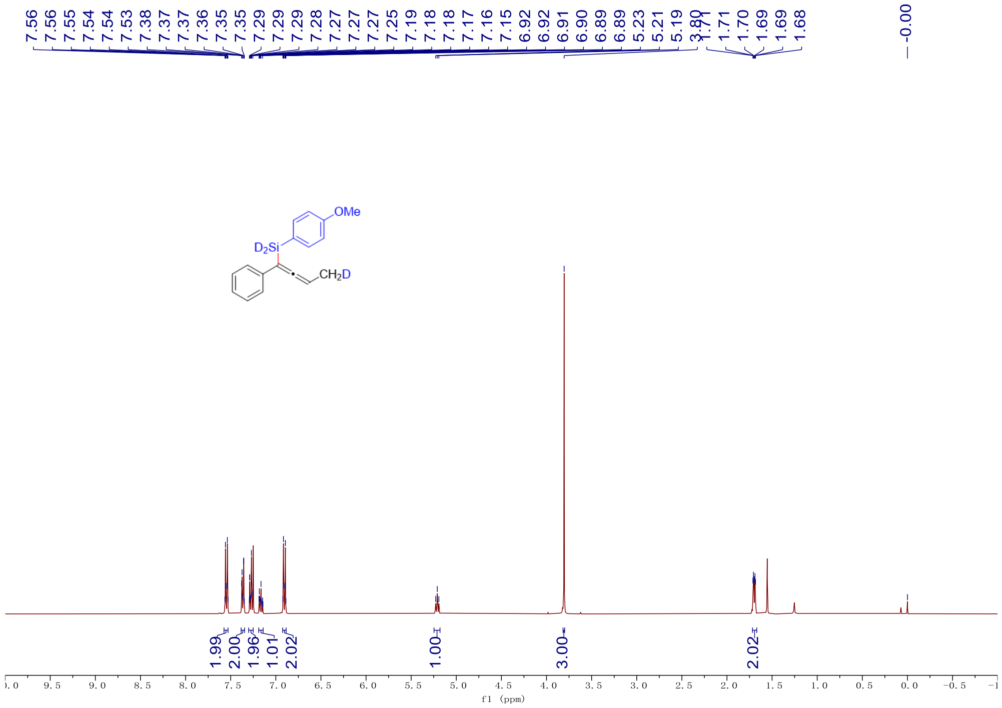

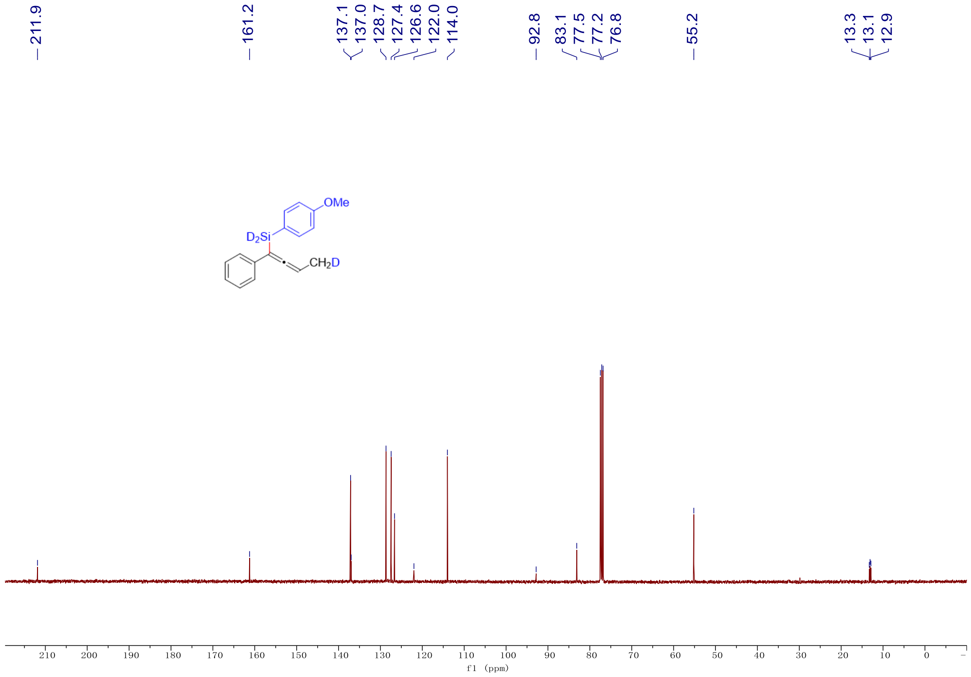

Phenyl(4-phenylbut-3-yn-2-yl)silane (5aa)

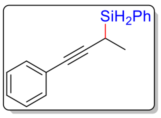

Prepared according to procedure 5 from **1a** (0.2 mmol, 25.6 mg) and **2a** (0.3 mmol, 32.5 mg). The product was isolated in 80% yield (37.6 mg) as colorless oil.

R*f*: 0.52 (petroleum ether).

HRMS (ESI) (m/z): Calcd for $C_{16}H_{17}Si$ $[M+H]^+$: 237.1100, found: 237.1113.

^{1}H NMR (400 MHz, $CDCl_3$) δ 7.73 – 7.66 (m, 2H), 7.48 – 7.38 (m, 1H), 7.39 (d, *J* = 7.2 Hz, 2H), 7.37 – 7.33 (m, 2H), 7.28 – 7.23 (m, 3H), 4.44 (d, *J* = 2.8 Hz, 2H), 2.35 (qt, *J* = 7.3, 2.8 Hz, 1H), 1.38 (d, *J* = 7.2 Hz, 3H).

^{13}C NMR (101 MHz, $CDCl_3$) δ 130.6, 128.3, 128.1, 124.3, 92.4, 81.6, 16.5, 9.8.

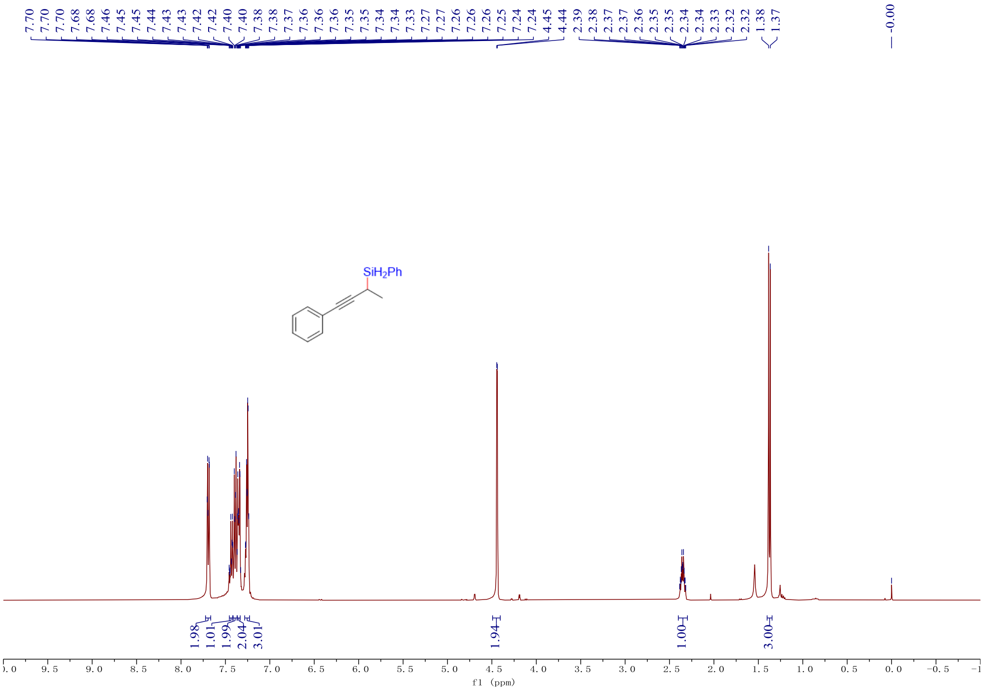

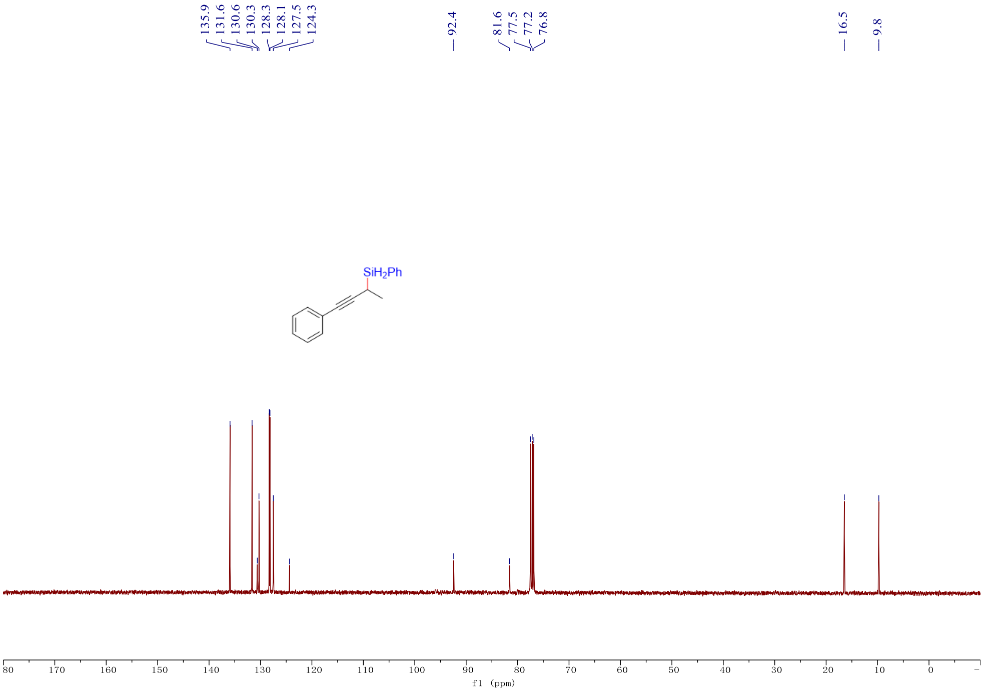

Phenyl(4-(*o*-tolyl)but-3-yn-2-yl)silane (5ba)

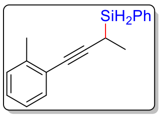

Prepared according to procedure 5 from **1b** (0.2 mmol, 28.4 mg) and **2a** (0.3 mmol, 32.5 mg). The product was isolated in 95% yield (47.4 mg) as colorless oil.

R*f*: 0.71 (petroleum ether).

HRMS (ESI) (m/z): Calcd for $C_{17}H_{19}Si$ $[M+H]^+$: 251.1256, found: 251.1259.

^{1}H NMR (400 MHz, $CDCl_3$) δ 7.71 – 7.66 (m, 2H), 7.45 – 7.35 (m, 3H), 7.32 (d, *J* = 7.6 Hz, 1H), 7.17 – 7.12 (m, 2H), 7.11 – 7.05 (m, 1H), 4.45 (d, *J* = 2.9 Hz, 2H), 2.44 – 2.37 (m, 1H), 2.33 (s, 3H), 1.40 (d, *J* = 7.2 Hz, 3H).

^{13}C NMR (101 MHz, $CDCl_3$) δ 140.0, 135.9, 131.9, 130.7, 130.3, 129.4, 128.2, 127.5, 125.5, 124.1, 96.4, 80.4, 20.8, 16.7, 10.0.

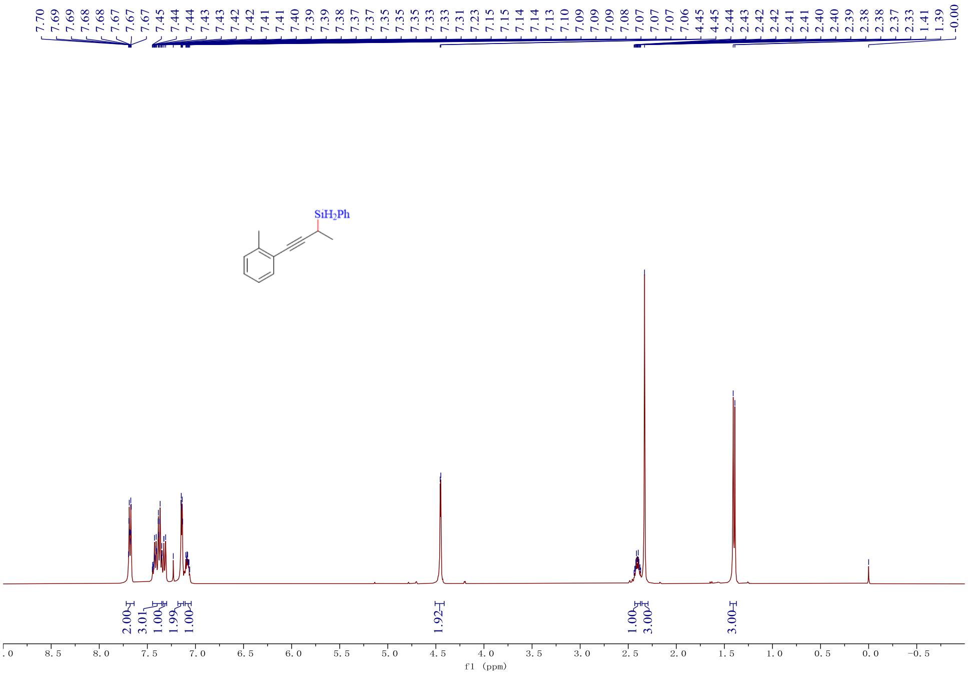

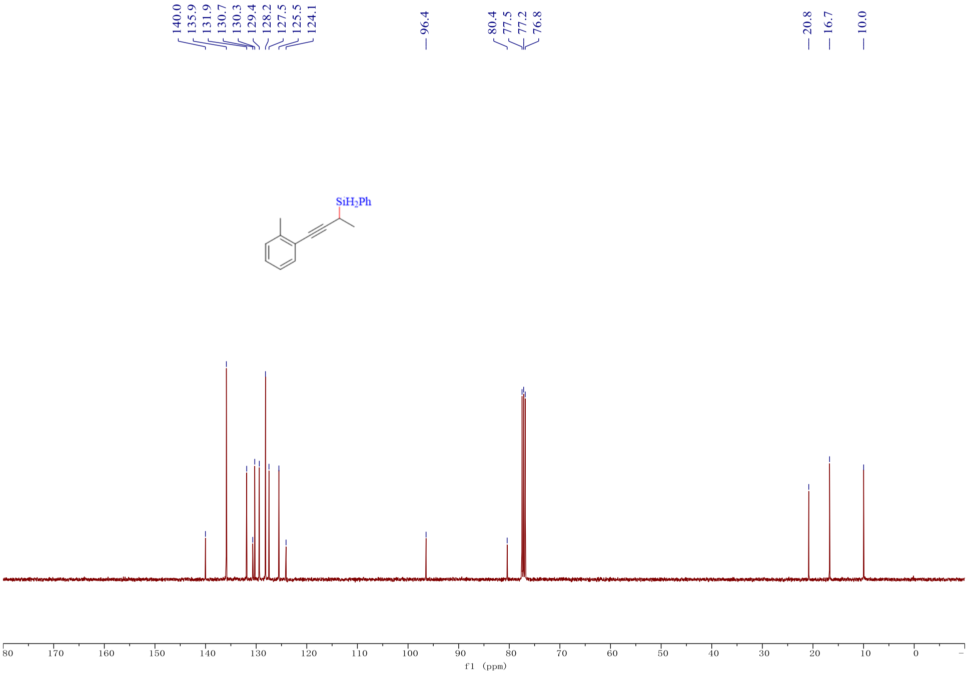

Phenyl(4-(*m*-tolyl)but-3-yn-2-yl)silane (5ca)

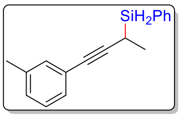

Prepared according to procedure 5 from **1c** (0.2 mmol, 28.4 mg) and **2a** (0.3 mmol, 32.5 mg). The product was isolated in 86% yield (42.9 mg) as colorless oil.

R*f*: 0.52 (petroleum ether).

HRMS (ESI) (m/z): Calcd for $C_{17}H_{19}Si$ $[M+H]^+$: 251.1256, found: 251.1269.

^{1}H NMR (400 MHz, $CDCl_3$) δ 7.71 – 7.68 (m, 2H), 7.46 – 7.36 (m, 3H), 7.17 – 7.14 (m, 3H), 7.07 – 7.04 (m, 1H), 4.43 (d, *J* = 2.8 Hz, 2H), 2.38 – 2.32 (m, 1H), 2.30 (s, 3H), 1.37 (d, *J* = 7.2 Hz, 3H).

^{13}C NMR (101 MHz, $CDCl_3$) δ 137.9, 135.9, 132.3, 130.7, 130.3, 128.7, 128.4, 128.2, 128.1, 124.2, 92.0, 81.7, 21.3, 16.5, 9.8.

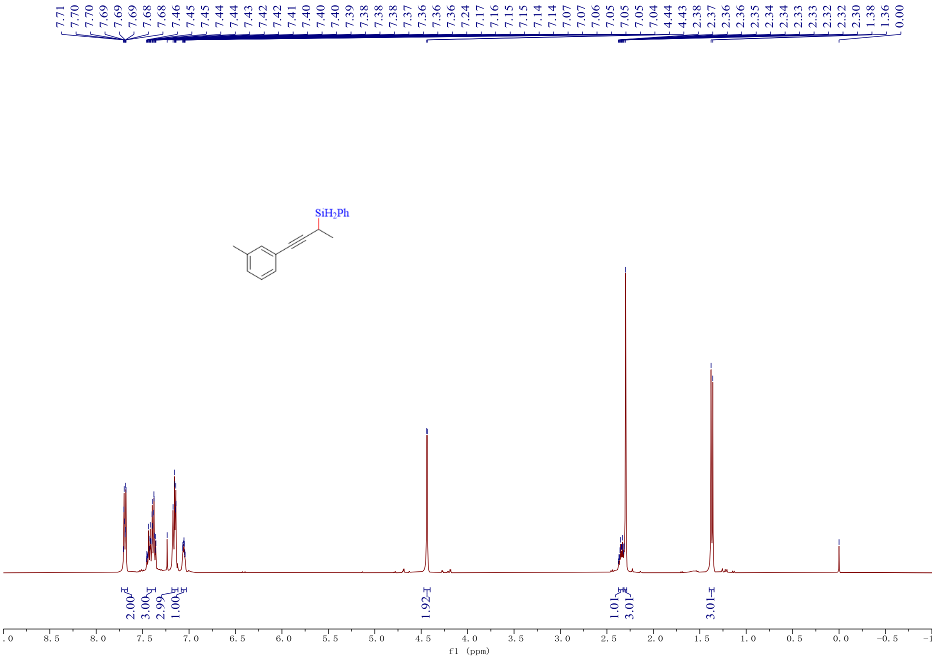

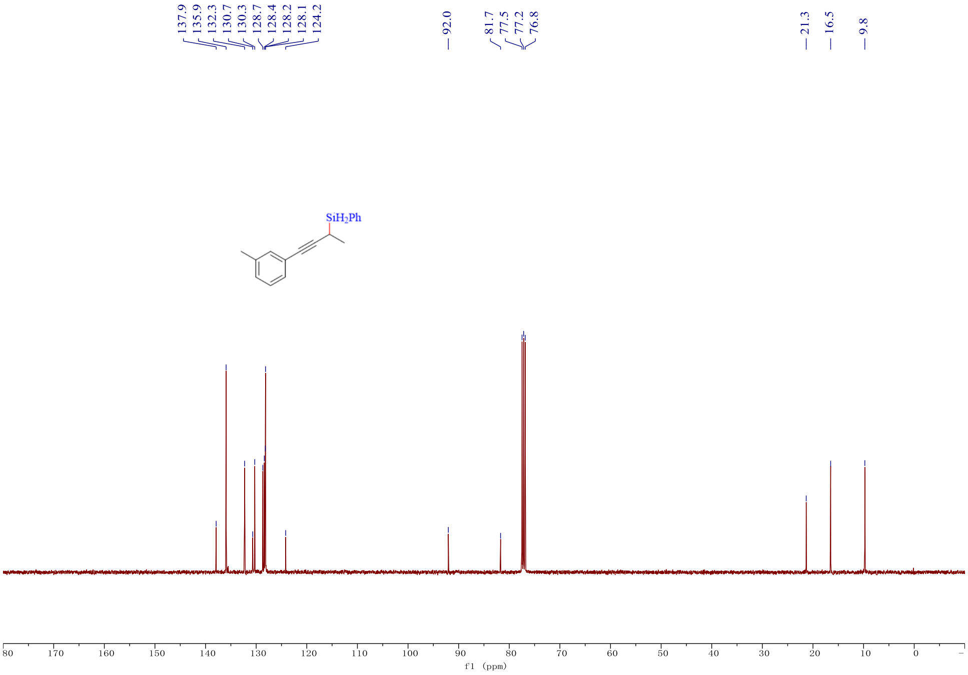

Phenyl(4-(*p*-tolyl)but-3-yn-2-yl)silane (5da)

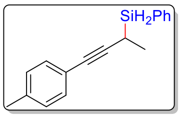

Prepared according to procedure 5 from **1d** (0.2 mmol, 28.4 mg) and **2a** (0.3 mmol, 32.5 mg). The product was isolated in 84% yield (42.1 mg) as colorless oil.

R*f*: 0.42 (petroleum ether).

HRMS (ESI) (m/z): Calcd for $C_{17}H_{19}Si$ $[M+H]^+$: 251.1256, found: 251.1253.

^{1}H NMR (400 MHz, $CDCl_3$) δ 7.69 (d, *J* = 6.8 Hz, 2H), 7.46 – 7.35 (m, 3H), 7.24 (d, *J* = 7.6 Hz, 2H), 7.07 (d, *J* = 7.6 Hz, 2H), 4.43 (d, *J* = 2.8 Hz, 2H), 2.38 – 2.33 (m, 1H), 2.32 (s, 3H), 1.37 (d, *J* = 7.3 Hz, 3H).

^{13}C NMR (101 MHz, $CDCl_3$) δ 137.4, 135.9, 131.5, 130.7, 130.3, 129.0, 128.1, 121.3, 91.5, 81.6, 21.5, 16.5, 9.7.

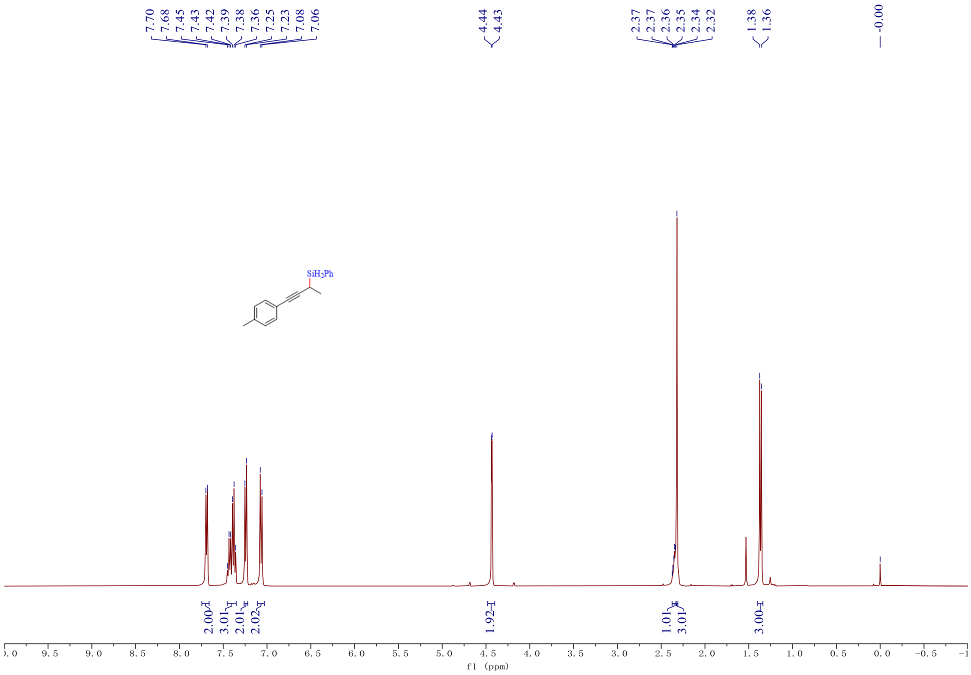

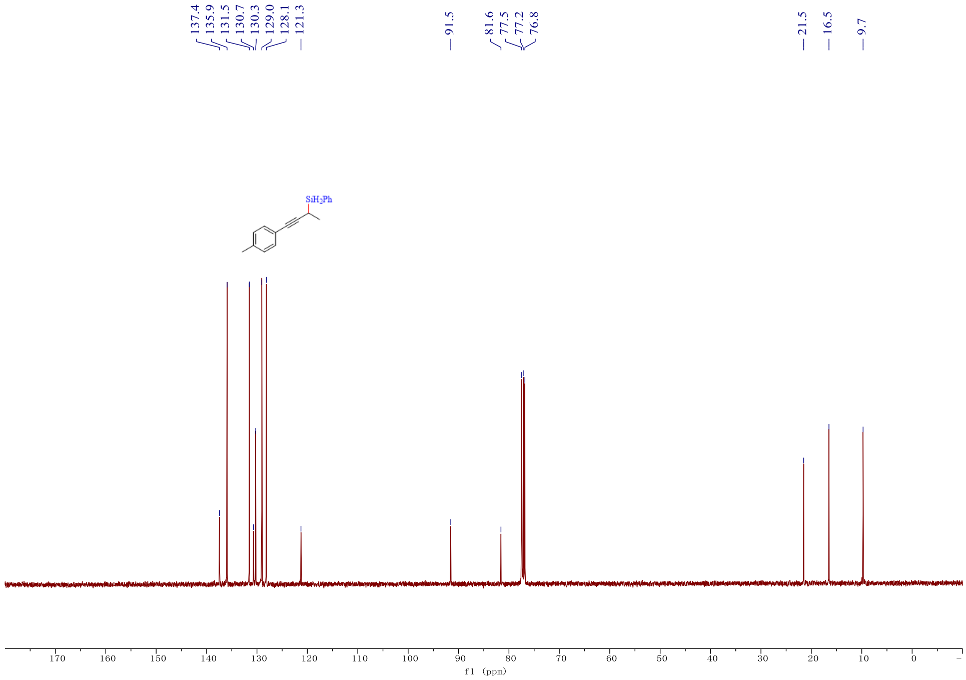

(4-(4-(*tert*-Butyl)phenyl)but-3-yn-2-yl)(phenyl)silane (5ea)

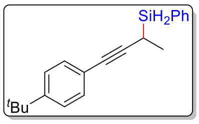

Prepared according to procedure 5 from **1e** (0.2 mmol, 36.9 mg) and **2a** (0.3 mmol, 32.5 mg). The product was isolated in 86% yield (50.5 mg) as colorless oil.

R*f*: 0.60 (petroleum ether).

HRMS (ESI) (m/z): Calcd for $C_{20}H_{25}Si$ $[M+H]^+$: 293.1726, found: 293.1742.

^{1}H NMR (400 MHz, $CDCl_3$) δ 7.73 – 7.66 (m, 2H), 7.46 – 7.33 (m, 3H), 7.28 (s, 4H), 4.43 (d, *J* = 2.8 Hz, 2H), 2.34 (m, 1H), 1.37 (d, *J* = 7.2 Hz, 3H), 1.29 (s, 9H).

^{13}C NMR (101 MHz, $CDCl_3$) δ 150.7, 136.0, 131.3, 130.7, 130.3, 128.1, 125.3, 121.4, 91.6, 81.6, 34.8, 31.4, 16.5, 9.8.

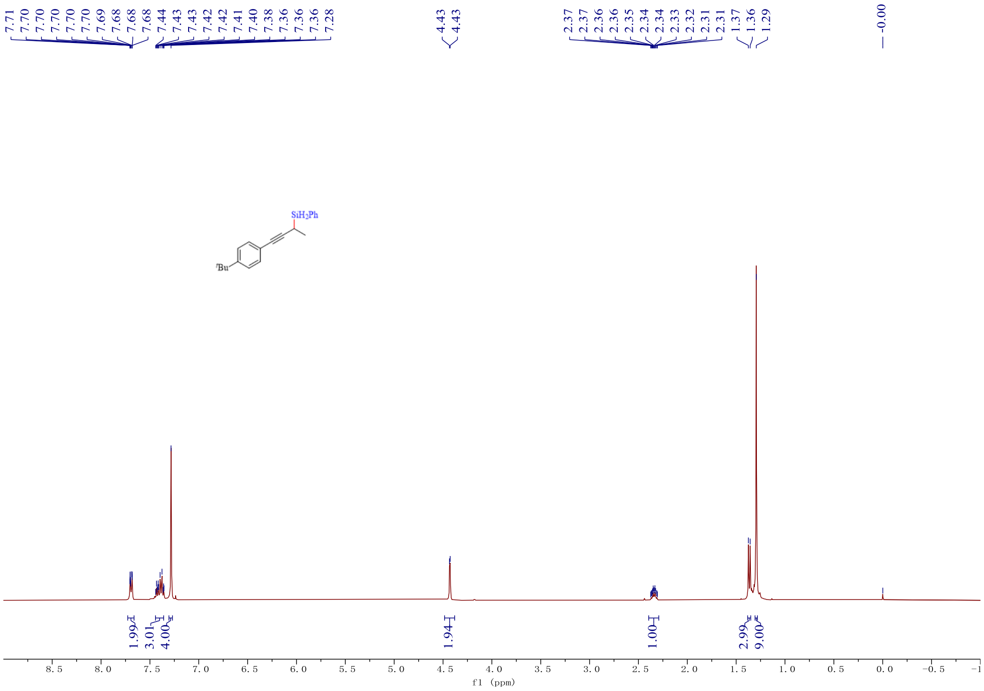

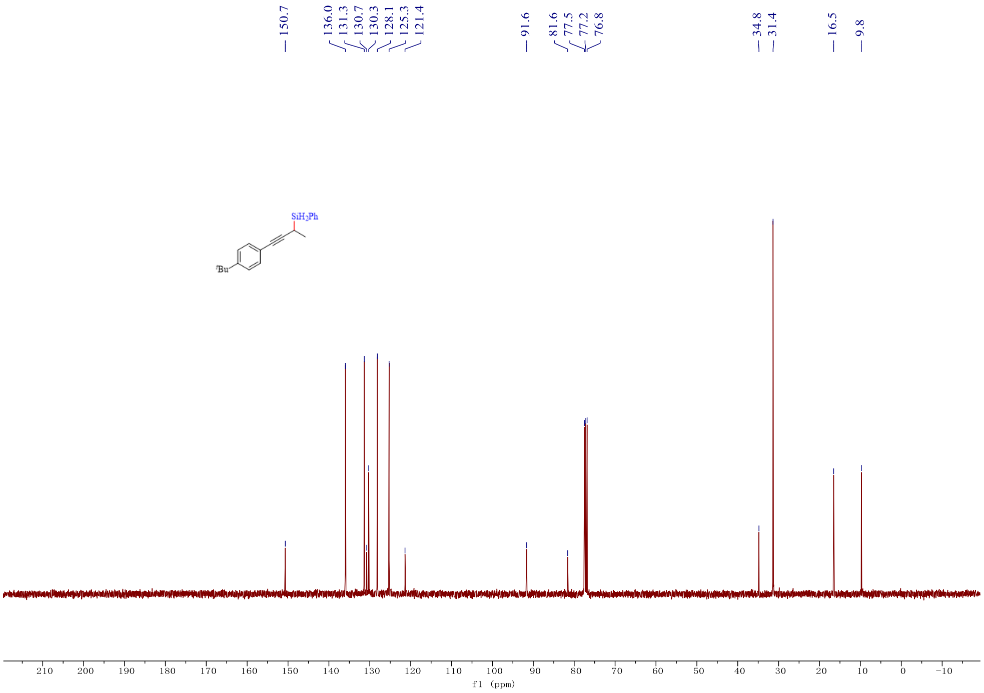

(4-([1,1'-Biphenyl]-4-yl)but-3-yn-2-yl)(phenyl)silane (5fa)

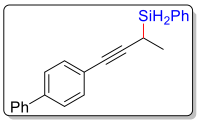

Prepared according to procedure 5 from **1f** (0.2 mmol, 40.9 mg) and **2a** (0.3 mmol, 32.5 mg). The product was isolated in 85% yield (52.9 mg) as colorless oil.

R*f*: 0.30 (petroleum ether).

HRMS (ESI) (m/z): Calcd for $C_{22}H_{20}NaSi$ $[M+Na]^+$: 335.1232, found: 335.1266.

^{1}H NMR (400 MHz, $CDCl_3$) δ 7.70 (dt, *J* = 8.2, 1.6 Hz, 2H), 7.58 – 7.55 (m, 2H), 7.52 – 7.48 (m, 2H), 7.44 – 7.36 (m, 7H), 7.35 – 7.30 (m, 1H), 4.46 (d, *J* = 2.8 Hz, 2H), 2.42 – 2.33 (m, 1H), 1.39 (d, *J* = 7.2 Hz, 3H).

^{13}C NMR (101 MHz, $CDCl_3$) δ 140.7, 140.2, 135.9, 132.0, 130.6, 130.3, 128.9, 128.2, 127.6, 127.1, 127.0, 123.3, 93.2, 81.4, 16.5, 9.9.

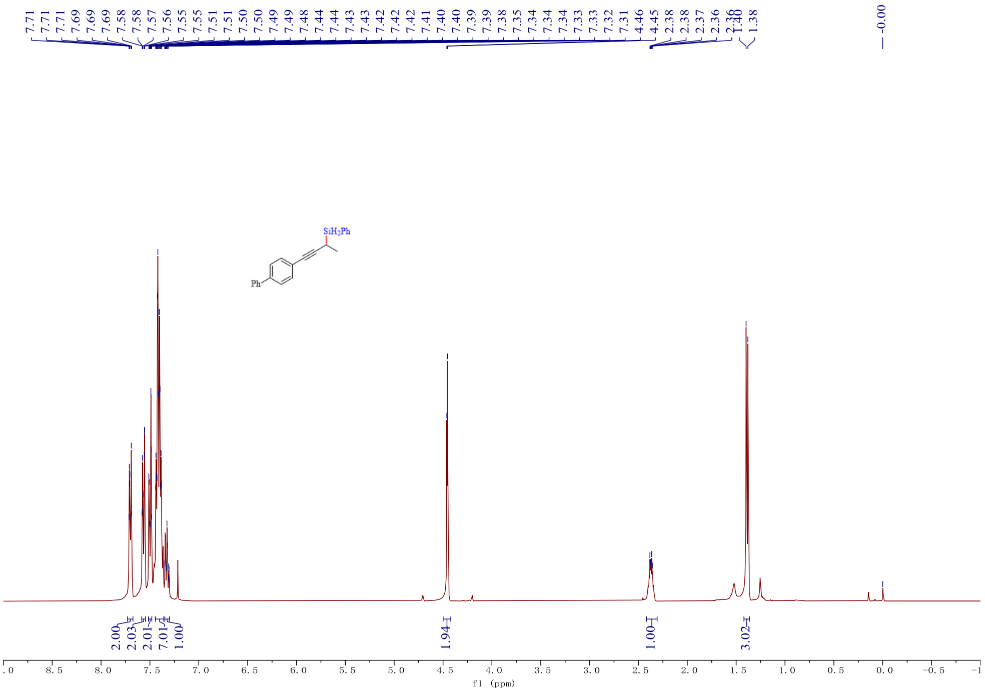

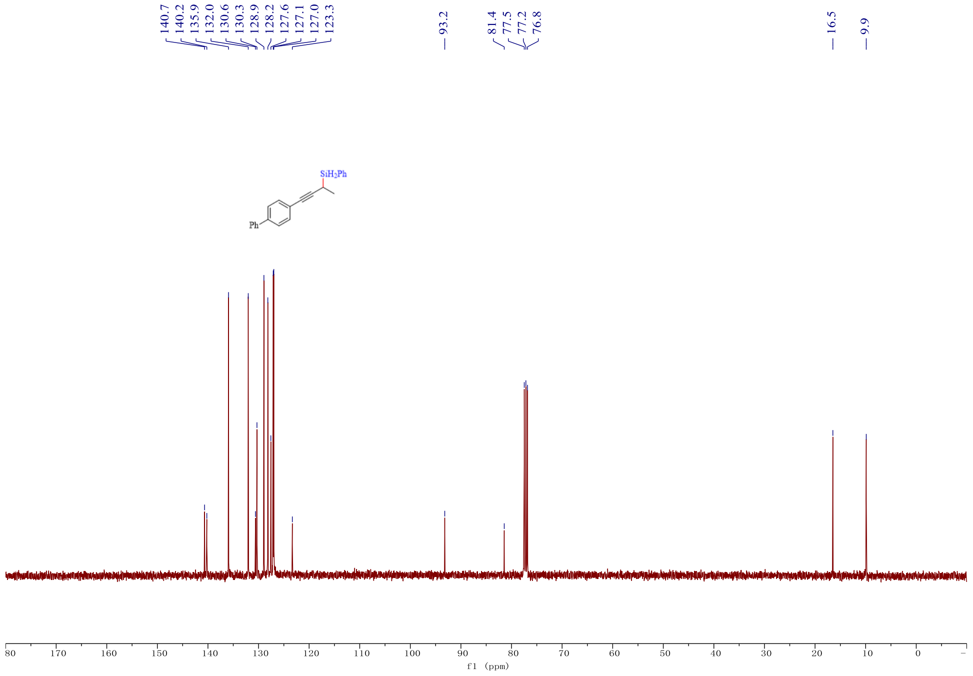

(4-(2-Fluorophenyl)but-3-yn-2-yl)(phenyl)silane (5ga)

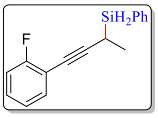

Prepared according to procedure 5 from **1g** (0.2 mmol, 29.2 mg) and **2a** (0.3 mmol, 32.5 mg). The product was isolated in 80% yield (40.5 mg) as colorless oil.

R*f*: 0.43 (petroleum ether).

HRMS (ESI) (m/z): Calcd for $C_{16}H_{16}FSi$ $[M+H]^+$: 255.1005, found: 255.0990.

^{1}H NMR (400 MHz, $CDCl_3$) δ 7.73 – 7.67 (m, 2H), 7.46 – 7.31 (m, 4H), 7.25 – 7.18 (m, 1H), 7.07 – 6.98 (m, 2H), 4.46 (d, *J* = 2.8 Hz, 2H), 2.45 – 2.34 (m, 1H), 1.39 (d, *J* = 7.2 Hz, 3H).

^{13}C NMR (101 MHz, $CDCl_3$) δ 162.9 (d, *J* = 250.2 Hz), 135.9, 133.7 (d, *J* = 1.6 Hz), 130.4, 130.3, 129.1 (d, *J* = 7.8 Hz), 128.2, 123.9 (d, *J* = 3.8 Hz), 115.5 (d, *J* = 21.1 Hz), 112.8 (d, *J* = 16.0 Hz), 97.9 (d, *J* = 3.2 Hz), 74.8, 16.4, 10.1.

^{19}F NMR (376 MHz, $CDCl_3$) δ -110.75.

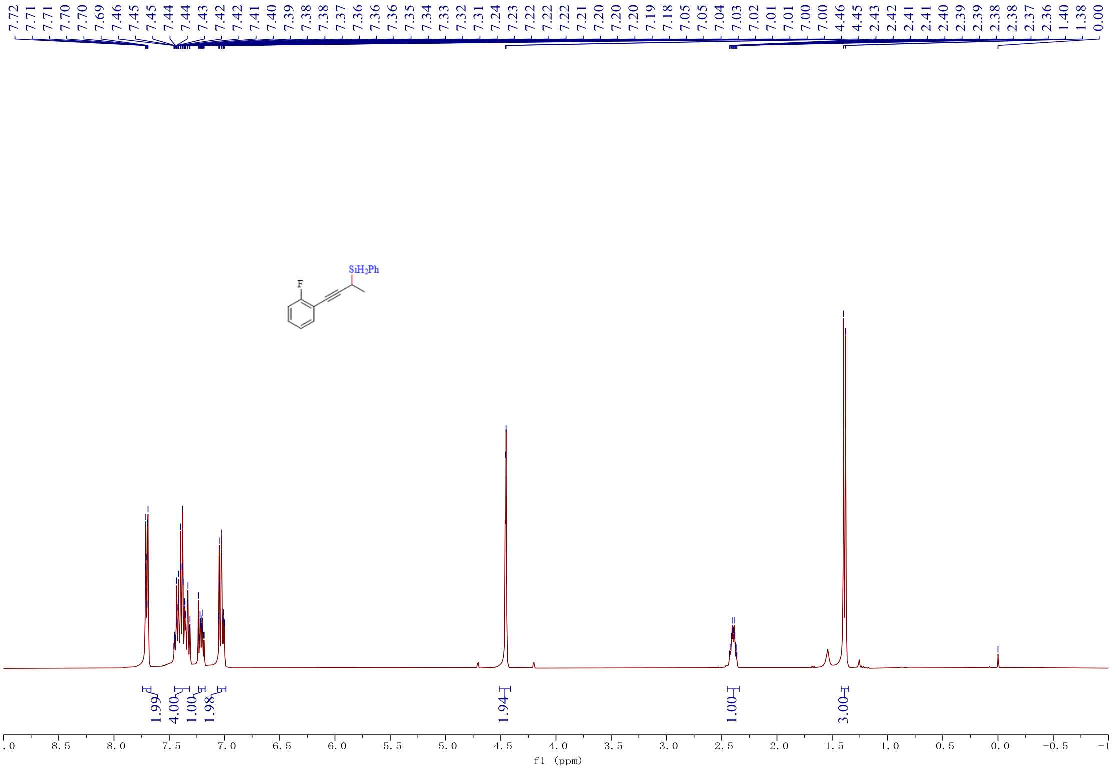

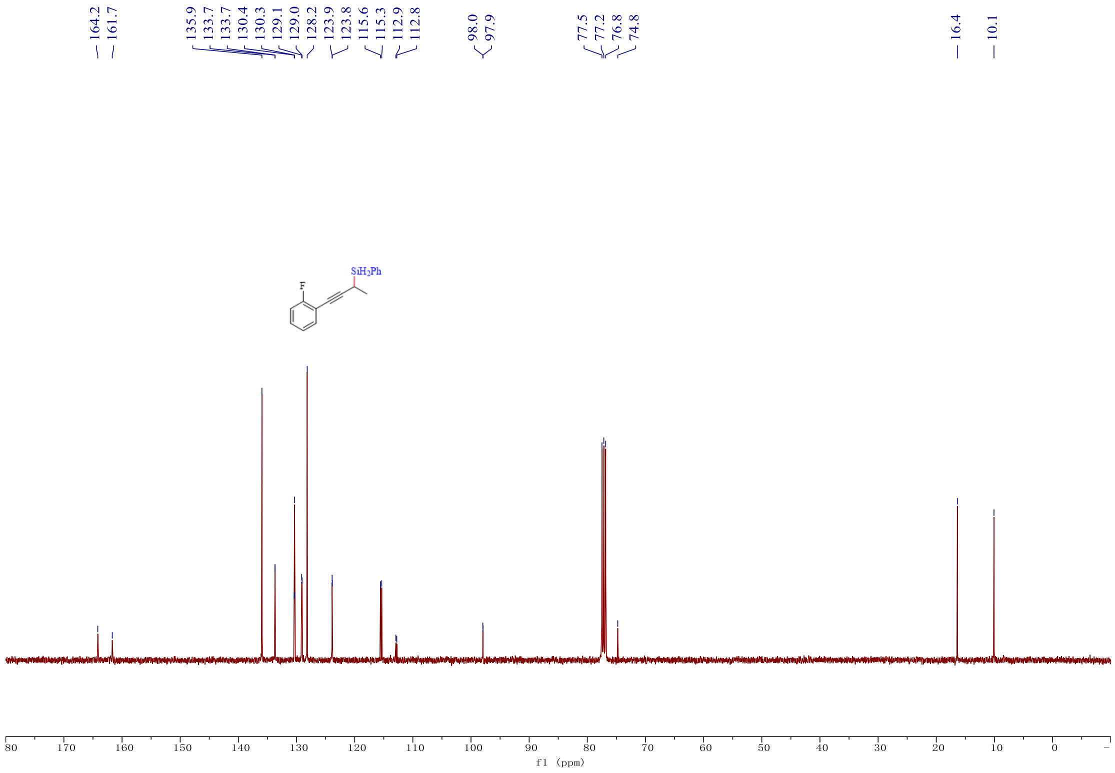
164.2
161.7
135.9
133.7
133.7
130.4
130.3
129.1
129.0
128.2
123.9
123.8
115.6
115.3
112.9
112.8
98.0
97.9
77.5
77.2
76.8
74.8
16.4
10.1
SiH2Ph
F
f1 (ppm)

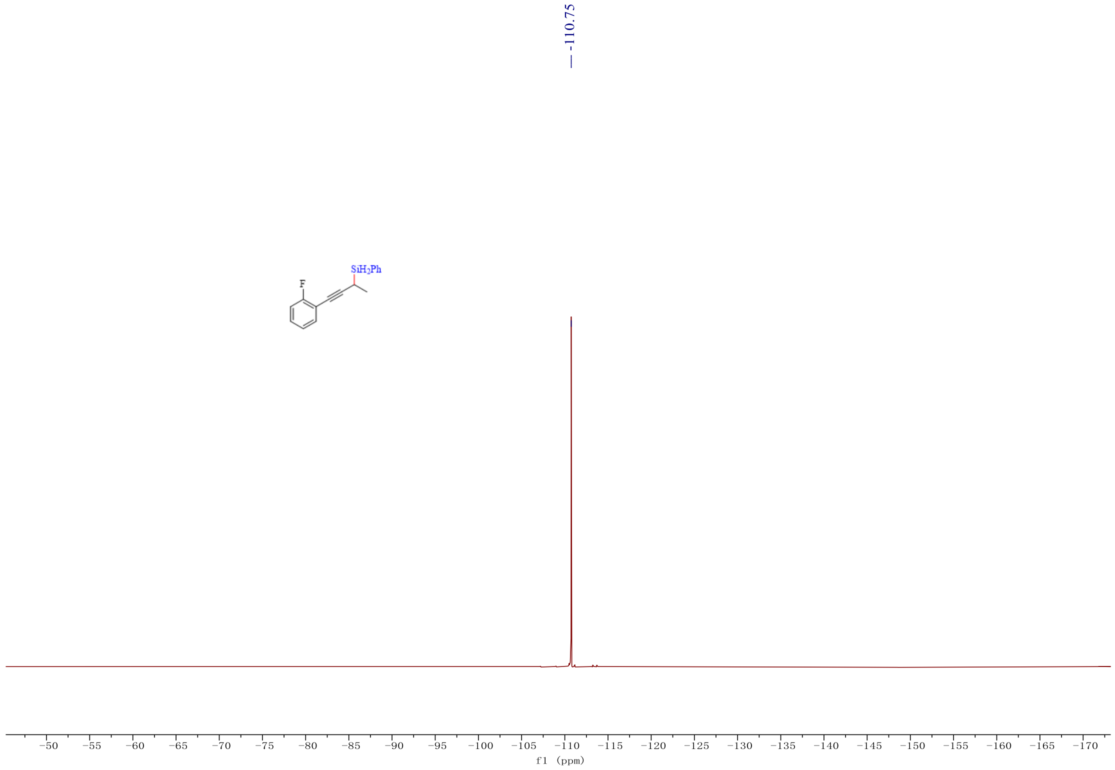
-110.75
SiH2Ph
F
f1 (ppm)

(4-(4-Fluorophenyl)but-3-yn-2-yl)(phenyl)silane (5ha)

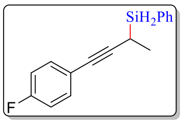

Prepared according to procedure 5 from **1h** (0.2 mmol, 29.2 mg) and **2a** (0.3 mmol, 32.5 mg). The product was isolated in 62% yield (31.3 mg) as colorless oil.

R*f*: 0.43 (petroleum ether).

HRMS (ESI) (m/z): Calcd for $C_{16}H_{16}FSi$ $[M+H]^+$: 255.1005, found: 255.0991.

^{1}H NMR (400 MHz, $CDCl_3$) δ 7.71 – 7.64 (m, 2H), 7.49 – 7.34 (m, 3H), 7.30 (m, 2H), 6.95 (tt, *J* = 8.8, 2.4 Hz, 2H), 4.43 (d, *J* = 2.8 Hz, 2H), 2.33 (qt, *J* = 7.2, 2.8 Hz, 1H), 1.37 (d, *J* = 7.2 Hz, 3H).

^{13}C NMR (101 MHz, $CDCl_3$) δ 162.1 (d, *J* = 248.0 Hz), 135.9, 133.4 (d, *J* = 8.1 Hz), 130.6, 130.3, 128.2, 120.4 (d, *J* = 3.5 Hz), 115.5 (d, *J* = 21.9 Hz), 92.1, 80.5, 16.4, 9.7.

^{19}F NMR (376 MHz, $CDCl_3$) δ -112.57.

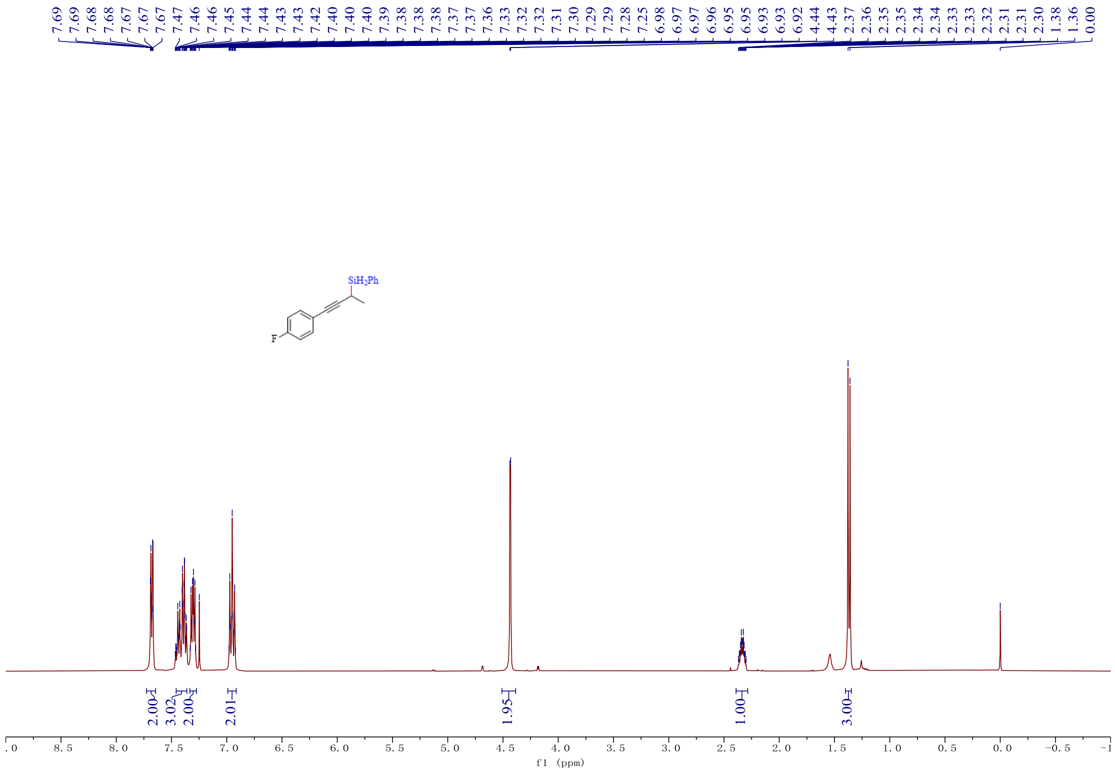

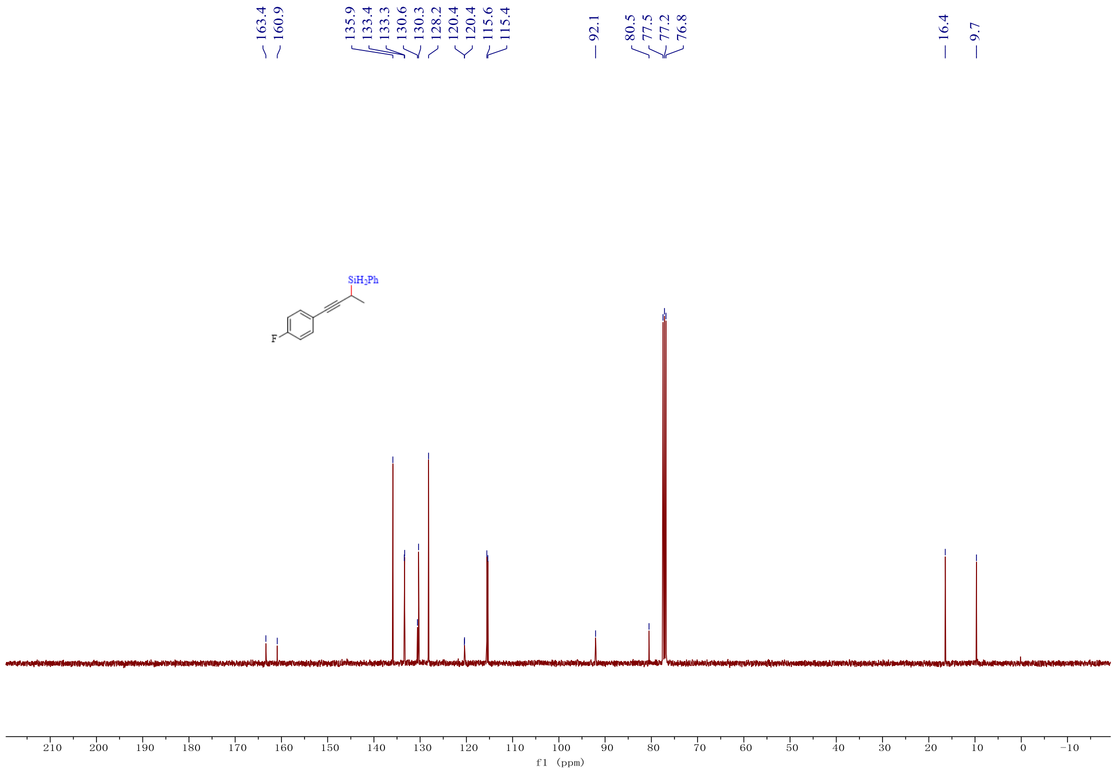
163.4
160.9
135.9
133.4
133.3
130.6
130.3
128.2
120.4
120.4
115.6
115.4
92.1
80.5
77.5
77.2
76.8
16.4
9.7
SiH2Ph
F
210 200 190 180 170 160 150 140 130 120 110 100 90 80 70 60 50 40 30 20 10 0 −10
f1 (ppm)

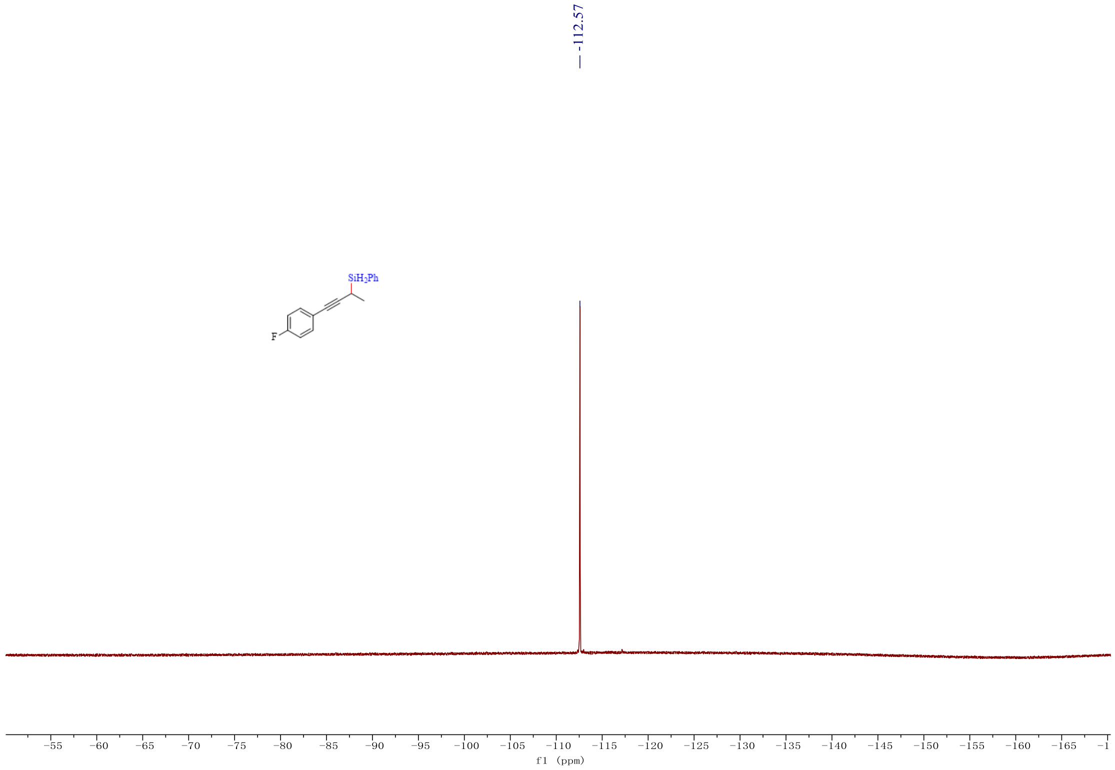
-112.57
SiH2Ph
F
−55 −60 −65 −70 −75 −80 −85 −90 −95 −100 −105 −110 −115 −120 −125 −130 −135 −140 −145 −150 −155 −160 −165
f1 (ppm)

(4-(2-Chlorophenyl)but-3-yn-2-yl)(phenyl)silane (5ia)

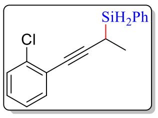

Prepared according to procedure 5 from **1i** (0.2 mmol, 32.5 mg) and **2a** (0.3 mmol, 32.5 mg). The product was isolated in 85% yield (46.2 mg) as colorless oil.

R*f*: 0.60 (petroleum ether).

HRMS (ESI) (m/z): Calcd for $C_{16}H_{16}ClSi$ $[M+H]^+$: 271.0710, found: 271.0707.

^{1}H NMR (400 MHz, $CDCl_3$) δ 7.73 – 7.68 (m, 2H), 7.48 – 7.39 (m, 1H), 7.40 – 7.33 (m, 4H), 7.19 – 7.12 (m, 2H), 4.47 (d, *J* = 2.8 Hz, 2H), 2.42 (qt, *J* = 7.2, 2.8 Hz, 1H), 1.41 (d, *J* = 7.2 Hz, 3H).

^{13}C NMR (101 MHz, $CDCl_3$) δ 136.0, 135.9, 133.4, 130.4, 130.3, 129.2, 128.5, 128.2, 126.4, 124.2, 98.2, 78.4, 16.4, 10.1.

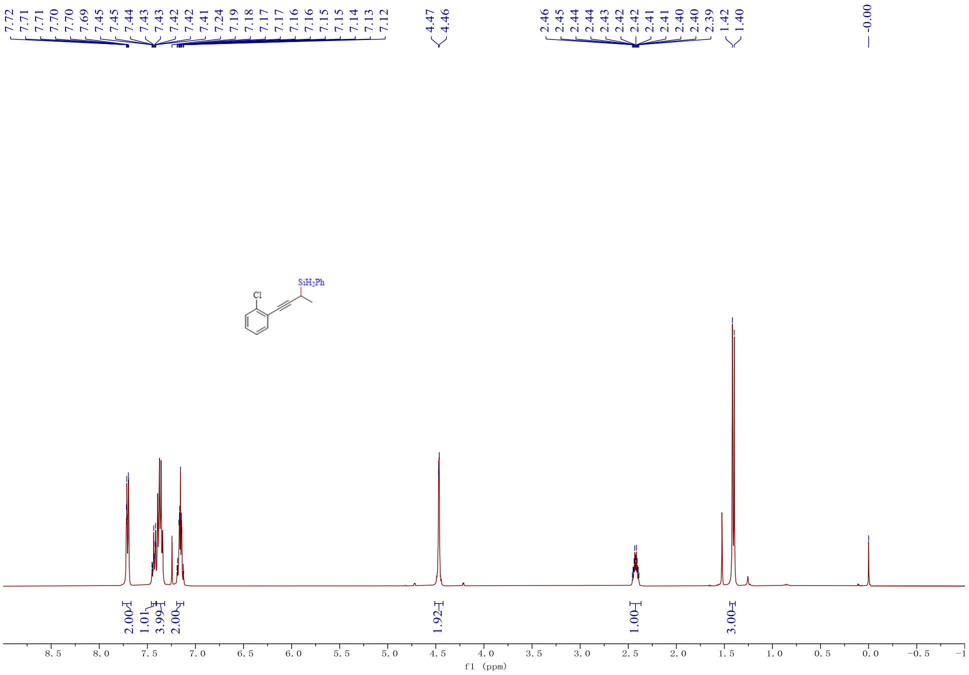

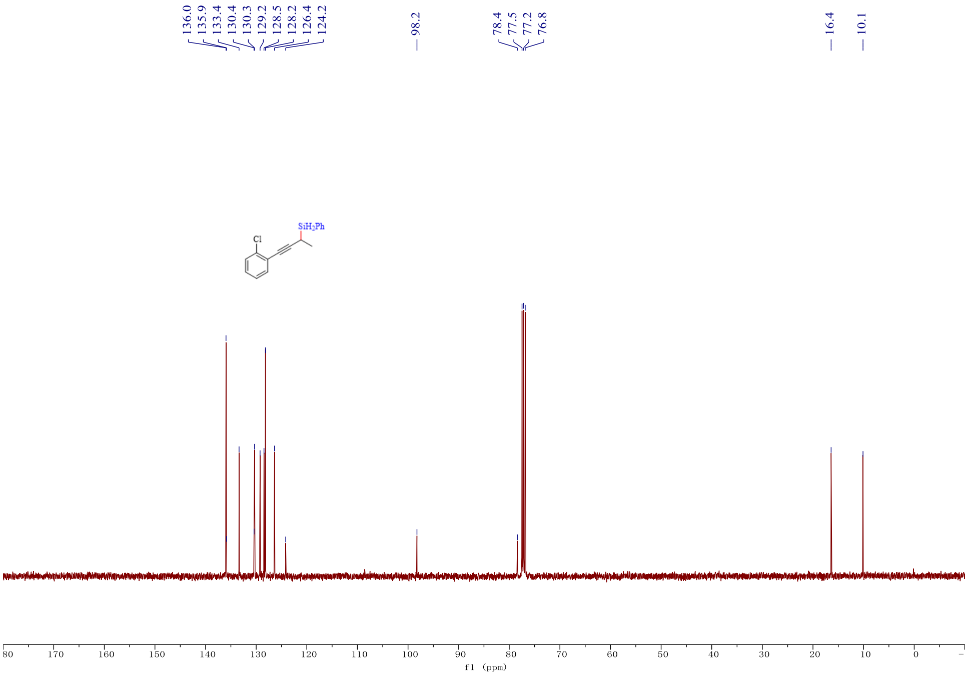

(4-(4-Chlorophenyl)but-3-yn-2-yl)(phenyl)silane (5ja)

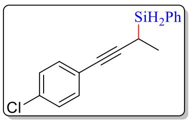

Prepared according to procedure 5 from **1j** (0.2 mmol, 40.9 mg) and **2a** (0.3 mmol, 32.5 mg). The product was isolated in 68% yield (36.7 mg) as colorless oil.

R*f*: 0.60 (petroleum ether).

HRMS (ESI) (m/z): Calcd for $C_{16}H_{15}ClNaSi$ $[M+Na]^+$: 293.0529, found: 293.0561.

^{1}H NMR (400 MHz, $CDCl_3$) δ 7.69 – 7.65 (m, 2H), 7.44 (tt, *J* = 7.2, 1.6 Hz, 1H), 7.40 – 7.35 (m, 2H), 7.27 – 7.21 (m, 4H), 4.43 (d, *J* = 2.8 Hz, 2H), 2.34 (qt, *J* = 7.2, 2.8 Hz, 1H), 1.37 (d, *J* = 7.2 Hz, 3H).

^{13}C NMR (101 MHz, $CDCl_3$) δ 135.9, 133.4, 132.9, 130.5, 130.4, 128.6, 128.2, 122.9, 93.6, 80.5, 16.4, 9.8.

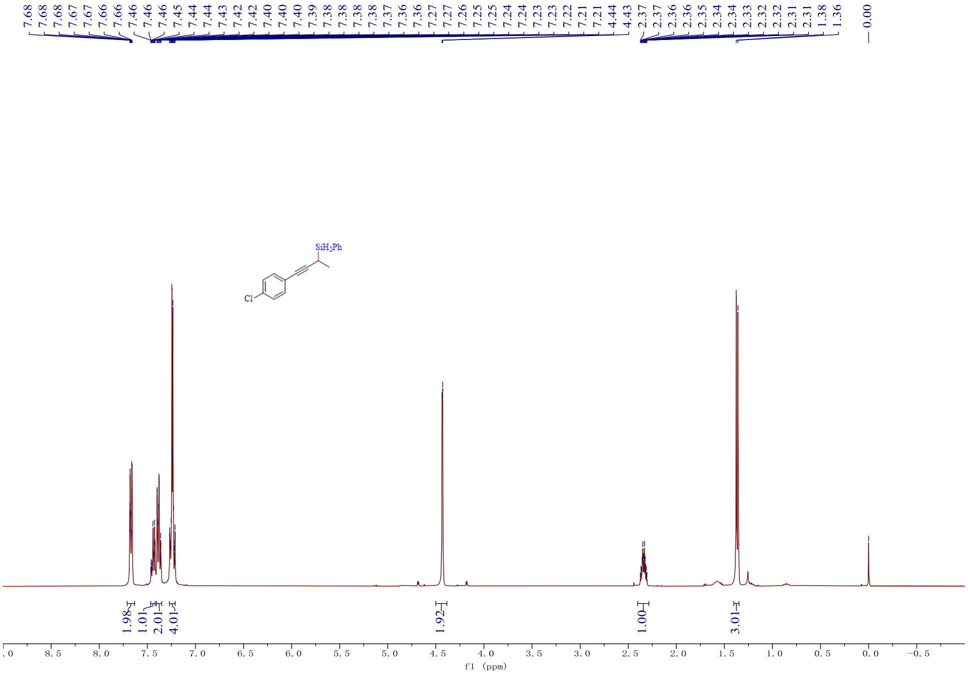

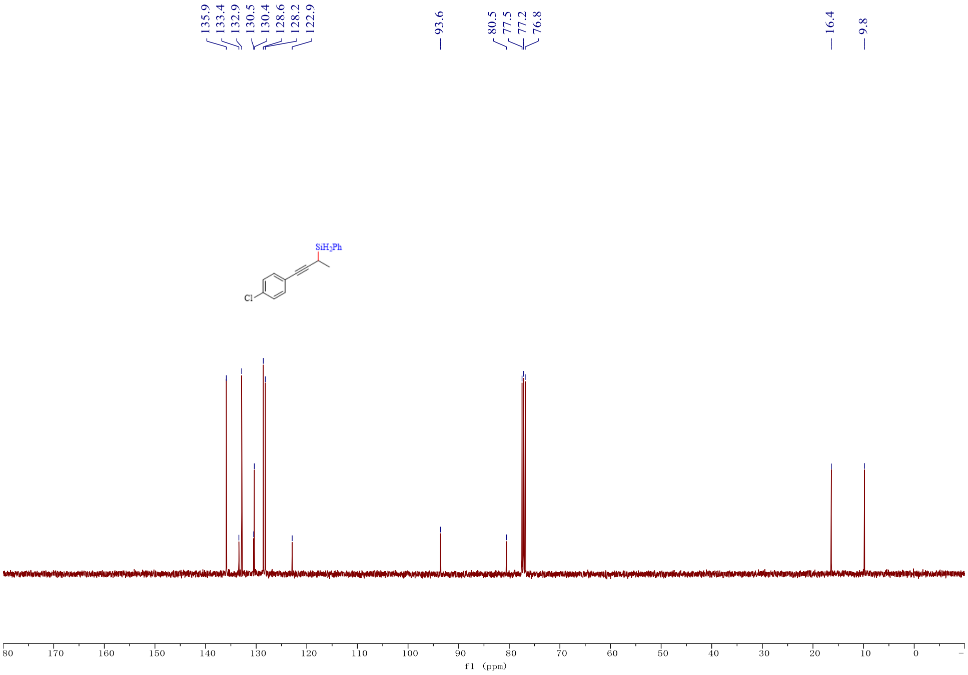

(4-(4-Bromophenyl)but-3-yn-2-yl)(phenyl)silane (5ka)

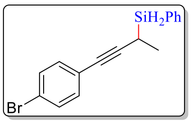

Prepared according to procedure 5 from **1k** (0.2 mmol, 41.4 mg) and **2a** (0.3 mmol, 32.5 mg). The product was isolated in 80% yield (50.0 mg) as colorless oil.

R*f* : 0.64 (petroleum ether).

HRMS (ESI) (m/z): Calcd for $C_{16}H_{16}BrSi$ $[M+H]^+$: 315.0205, found: 315.0219.

^{1}H NMR (400 MHz, $CDCl_3$) δ 7.67 (m, 2H), 7.44 (t, *J* = 7.4 Hz, 1H), 7.41 – 7.36 (m, 4H), 7.19 (d, *J* = 8.6 Hz, 2H), 4.43 (d, *J* = 2.8 Hz, 2H), 2.38 – 2.29 (m, 1H), 1.37 (d, *J* = 7.2 Hz, 3H).

^{13}C NMR (101 MHz, $CDCl_3$) δ 135.9, 133.1, 131.5, 130.5, 130.4, 128.2, 123.3, 121.6, 93.8, 80.6, 16.3, 9.8.

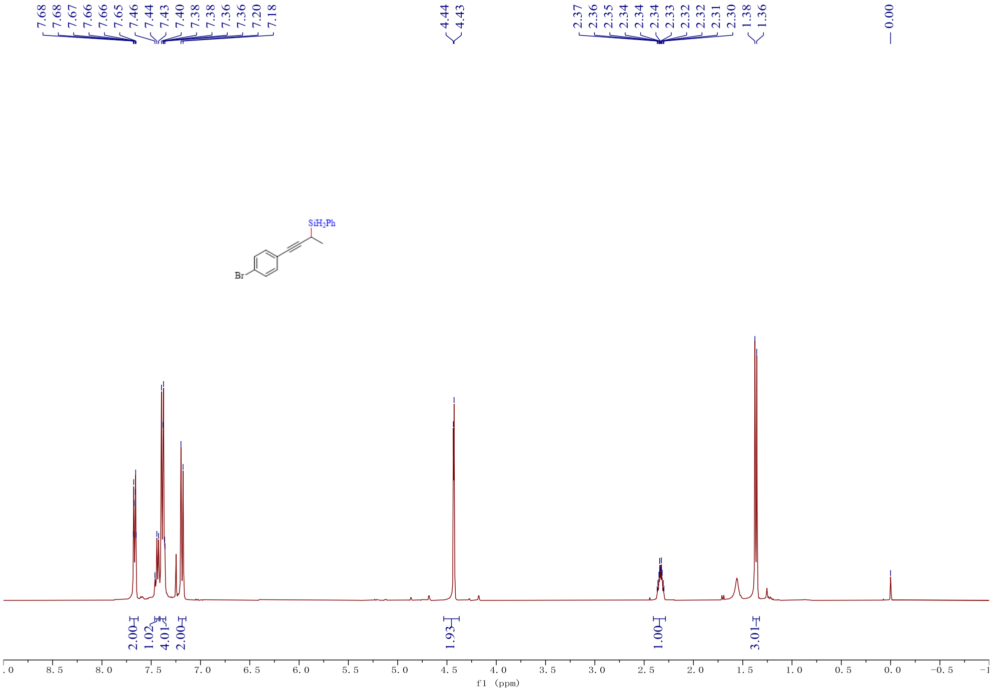

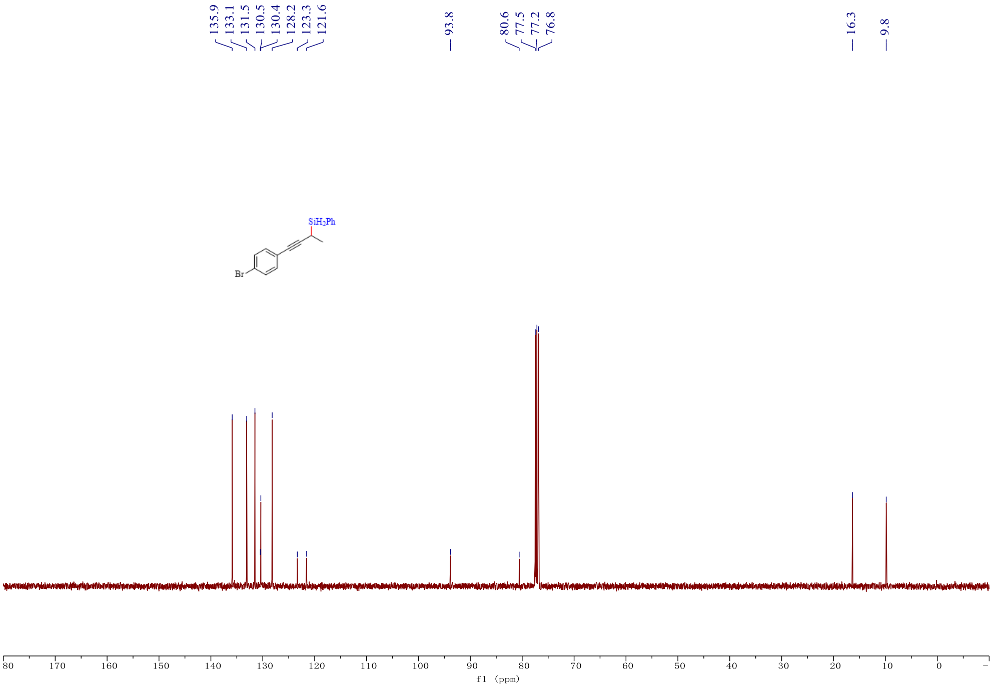

(4-(Naphthalen-1-yl)but-3-yn-2-yl)(phenyl)silane (5la)

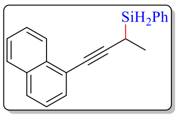

Prepared according to procedure 5 from **1l** (0.2 mmol, 35.6 mg) and **2a** (0.3 mmol, 32.5 mg). The product was isolated in 75% yield (43.0 mg) as colorless oil.

R*f*: 0.42 (petroleum ether).

HRMS (ESI) (m/z): Calcd for $C_{20}H_{19}Si$ $[M+H]^+$: 287.1256, found: 287.1260.

^{1}H NMR (400 MHz, $CDCl_3$) δ 8.21 – 8.13 (m, 1H), 7.85 – 7.76 (m, 1H), 7.74 (m, 3H), 7.57 (dd, *J* = 7.1, 1.2 Hz, 1H), 7.51 – 7.41 (m, 3H), 7.38 (m, 3H), 4.53 (d, *J* = 2.8 Hz, 2H), 2.53 (qt, *J* = 7.2, 2.9 Hz, 1H), 1.49 (d, *J* = 7.3 Hz, 3H).

^{13}C NMR (101 MHz, $CDCl_3$) δ 136.0, 133.6, 133.3, 130.6, 130.4, 130.0, 128.3, 128.2, 127.9, 126.5, 126.5, 126.3, 125.4, 122.0, 97.5, 79.5, 16.7, 10.2

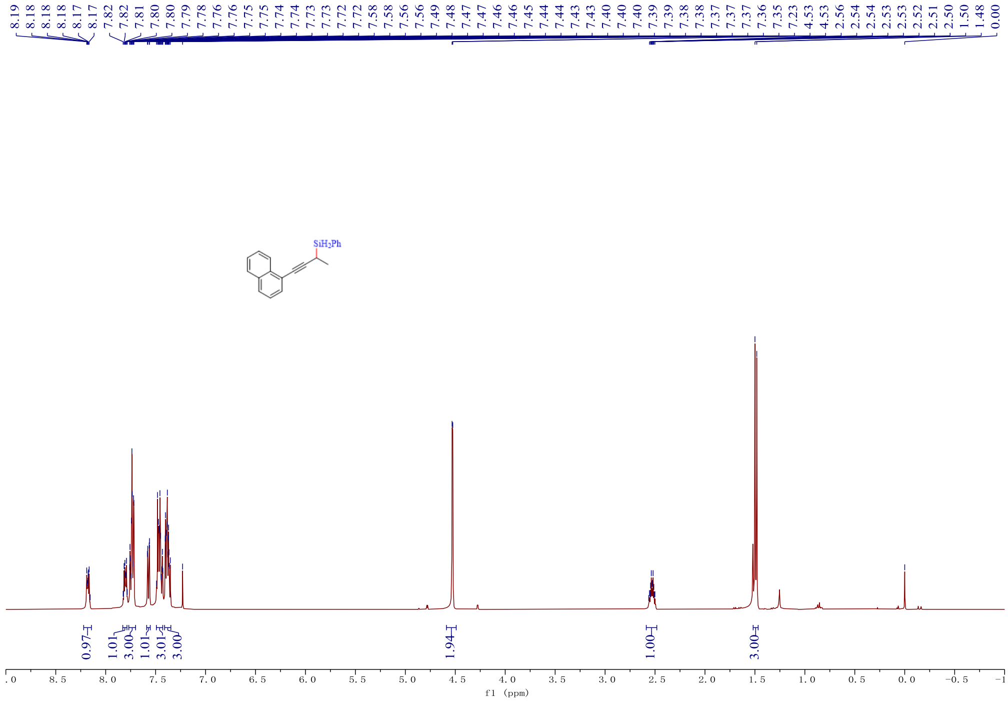

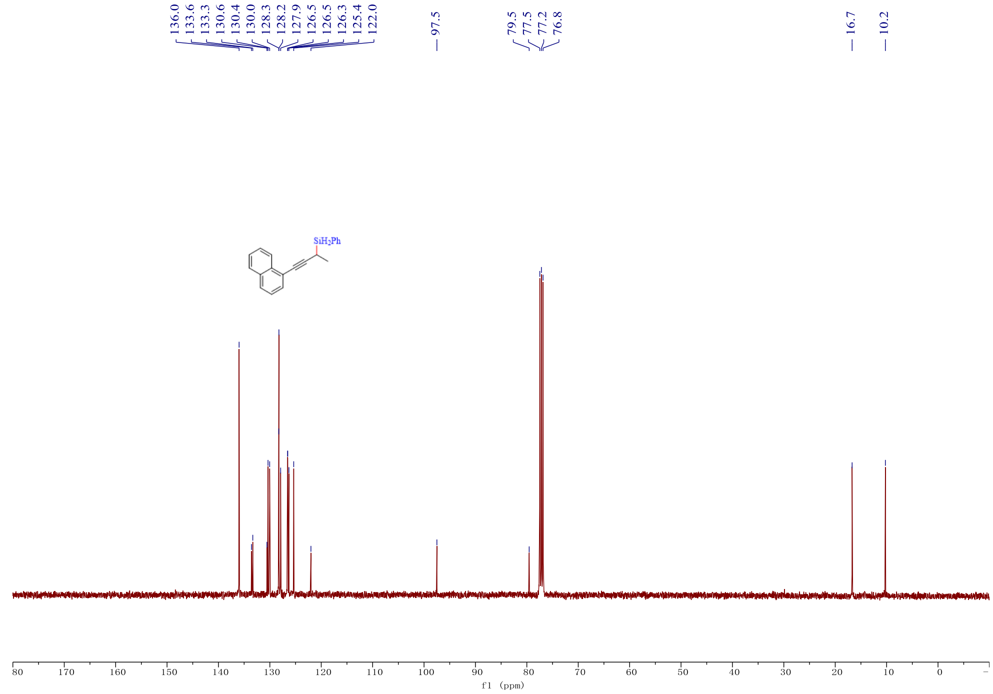

(4-(Naphthalen-2-yl)but-3-yn-2-yl)(phenyl)silane (5ma)

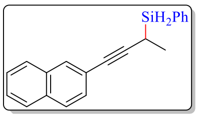

Prepared according to procedure 5 from **1m** (0.2 mmol, 35.6 mg) and **2a** (0.3 mmol, 32.5 mg). The product was isolated in 74% yield (42.4 mg) as colorless oil.

R*f*: 0.36 (petroleum ether).

HRMS (ESI) (m/z): Calcd for $C_{20}H_{19}Si$ $[M+H]^+$: 287.1256, found: 287.1258.

^{1}H NMR (400 MHz, $CDCl_3$) δ 7.85 (s, 1H), 7.82 – 7.68 (m, 5H), 7.50 – 7.35 (m, 6H), 4.48 (d, *J* = 2.8 Hz, 2H), 2.46 – 2.35 (m, 1H), 1.41 (d, *J* = 7.3 Hz, 3H).

^{13}C NMR (101 MHz, $CDCl_3$) δ 136.0, 133.2, 132.5, 131.0, 130.6, 130.3, 128.9, 128.2, 127.9, 127.8, 127.7, 126.5, 126.3, 121.7, 92.9, 81.9, 16.5, 9.9.

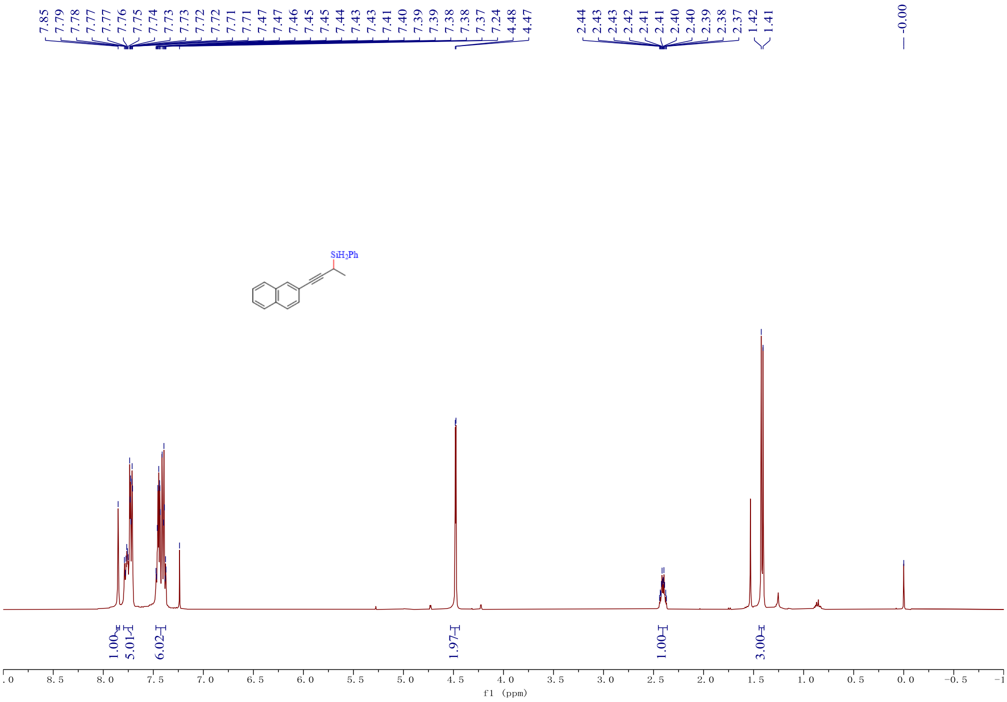

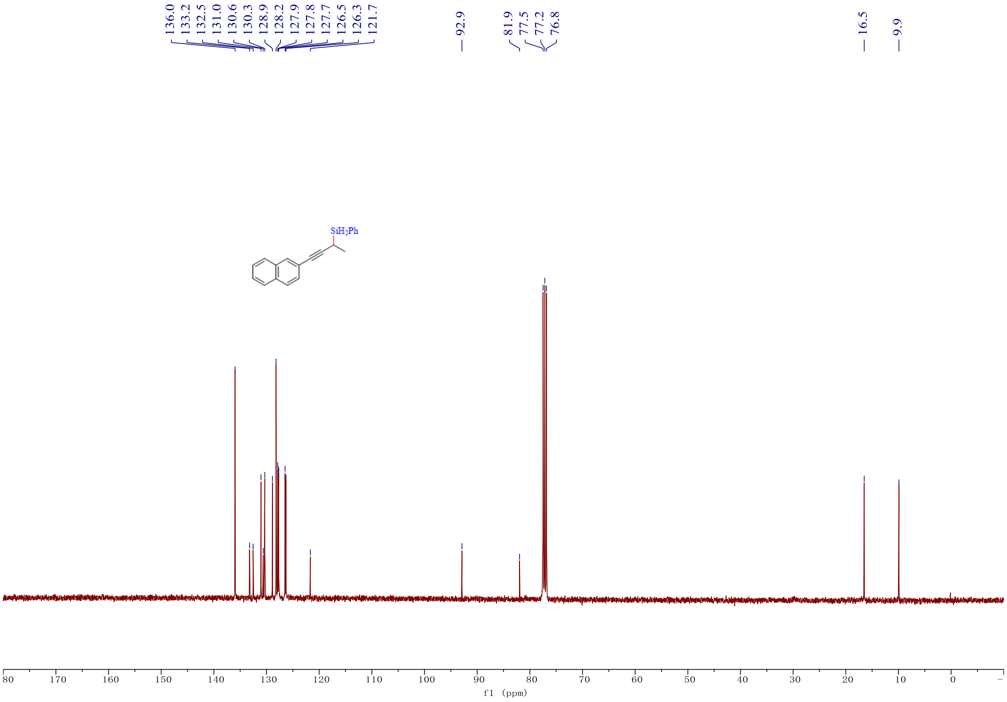

(4-(4-Methoxyphenyl)but-3-yn-2-yl)(phenyl)silane (5na)

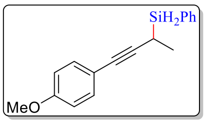

Prepared according to procedure 5 from **1n** (0.2 mmol, 31.6 mg) and **2a** (0.3 mmol, 32.5 mg). The product was isolated in 78% yield (41.6 mg) as colorless oil.

R*f*: 0.30 (ethyl acetate : petroleum ether = 1:100).

HRMS (ESI) (m/z): Calcd for $C_{17}H_{19}OSi$ $[M+H]^+$: 267.1205, found: 267.1197.

^{1}H NMR (400 MHz, $CDCl_3$) δ 7.71 – 7.67 (m, 2H), 7.46 – 7.42 (m, 1H), 7.38 (tt, *J* = 6.8, 1.7 Hz, 2H), 7.29 (m, 2H), 6.80 (dt, *J* = 8.8, 2.4 Hz, 2H), 4.43 (d, *J* = 2.8 Hz, 2H), 3.79 (s, 3H), 2.34 (qt, *J* = 7.2, 2.8 Hz, 1H), 1.36 (d, *J* = 7.2 Hz, 3H).

^{13}C NMR (101 MHz, $CDCl_3$) δ 159.1, 135.9, 133.0, 130.8, 130.3, 128.1, 116.5, 113.9, 90.7, 81.3, 55.4, 16.6, 9.7.

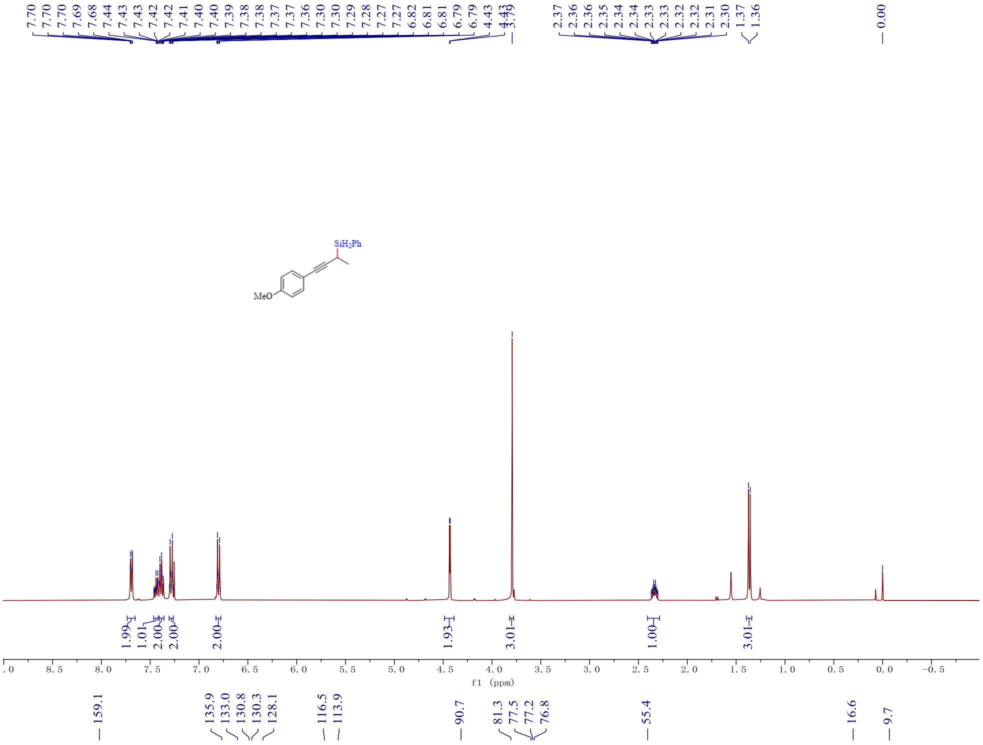

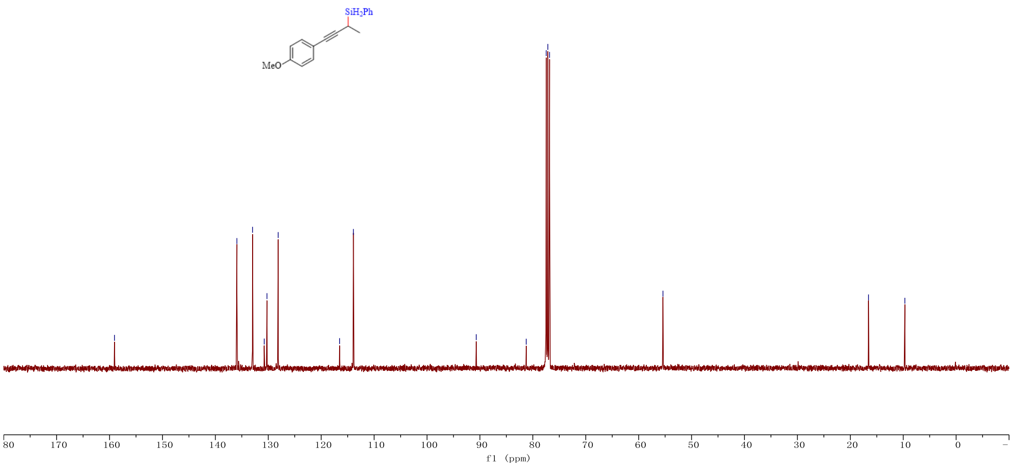

(4-(Benzo[*d*][1,3]dioxol-5-yl)but-3-yn-2-yl)(phenyl)silane (5oa)

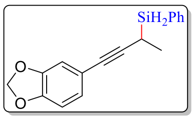

Prepared according to procedure 5 from **1o** (0.2 mmol, 34.5 mg) and **2a** (0.3 mmol, 32.5 mg). The product was isolated in 63% yield (35.3 mg) as colorless oil.

R*f*: 0.25 (ethyl acetate : petroleum ether = 1:100).

HRMS (ESI) (m/z): Calcd for $C_{17}H_{17}O_2Si$ $[M+H]^+$: 281.0998, found: 281.1012.

^{1}H NMR (400 MHz, $CDCl_3$) δ 7.68 (dd, *J* = 7.9, 1.5 Hz, 2H), 7.46 – 7.42 (m, 1H), 7.41 – 7.36 (m, 2H), 6.86 (dd, *J* = 8.0, 1.6 Hz, 1H), 6.79 (d, *J* = 1.6 Hz, 1H), 6.70 (d, *J* = 8.0 Hz, 1H), 5.94 (s, 2H), 4.42 (d, *J* = 2.8 Hz, 2H), 2.32 (qt, *J* = 7.2, 2.8 Hz, 1H), 1.36 (d, *J* = 7.2 Hz, 3H).

^{13}C NMR (101 MHz, $CDCl_3$) δ 147.4, 147.3, 135.9, 130.7, 130.3, 128.2, 125.9, 117.7, 111.8, 108.4, 101.2, 90.6, 81.3, 16.5, 9.7.

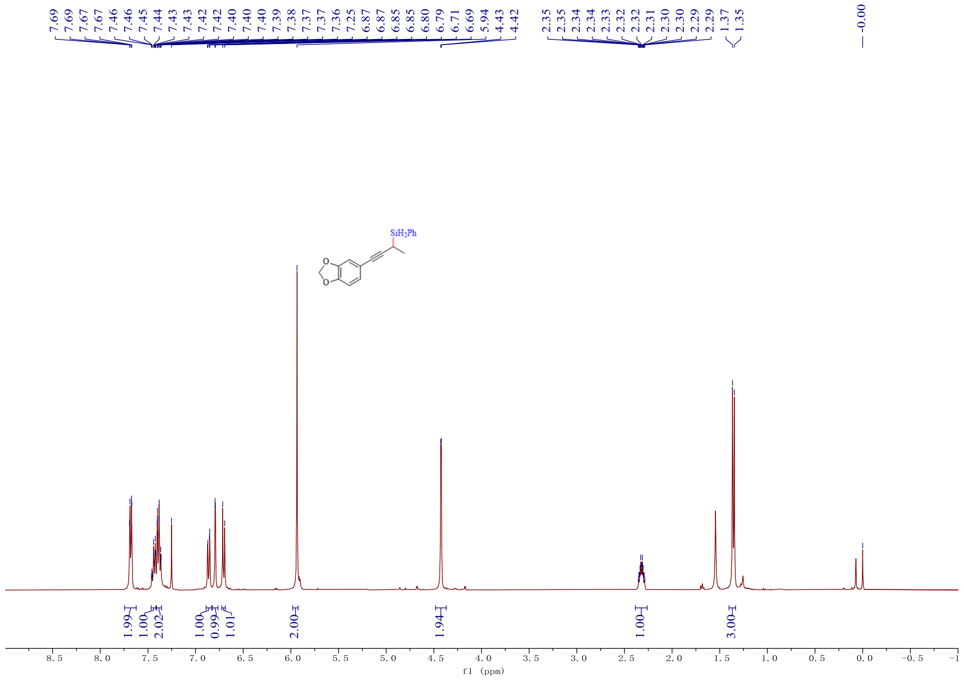

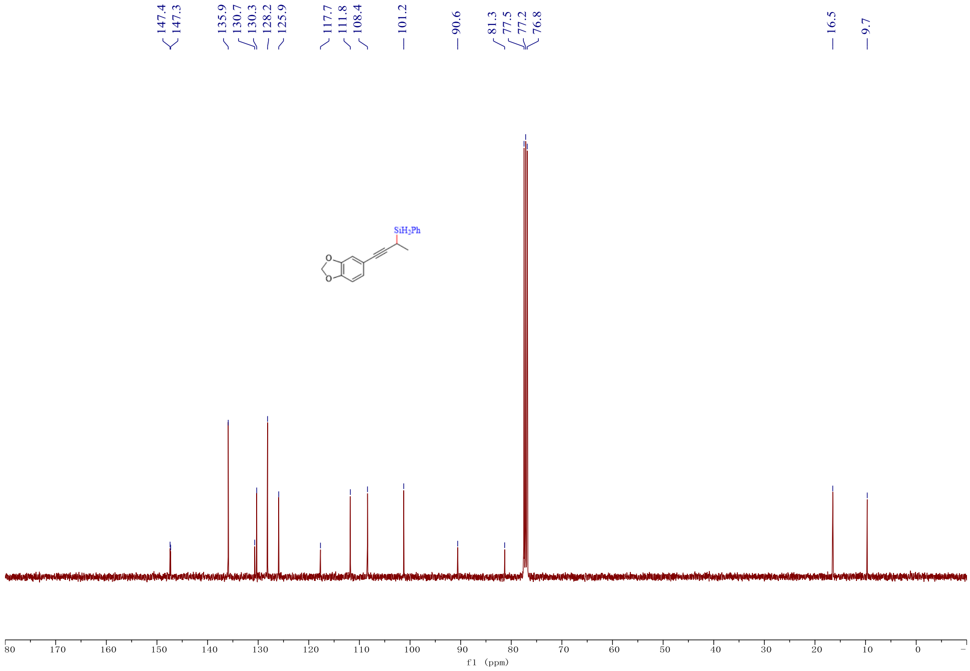

N,N-diphenyl-4-(3-(phenylsilyl)but-1-yn-1-yl)aniline (5pa)

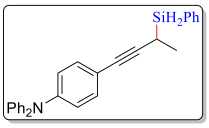

Prepared according to procedure 5 from **1p** (0.2 mmol, 59.1 mg) and **2a** (0.3 mmol, 32.5 mg). The product was isolated in 74% yield (59.1 mg) as colorless oil.

R*f* : 0.50 (ethyl acetate : petroleum ether = 1:100).

HRMS (ESI) (m/z): Calcd for $C_{28}H_{26}NSi$ $[M+H]^+$: 404.1835, found: 404.1829.

^{1}H NMR (400 MHz, $CDCl_3$) δ 7.69 (dt, *J* = 6.5, 1.6 Hz, 2H), 7.45 – 7.35 (m, 3H), 7.25 – 7.19 (m, 6H), 7.09 – 7.06 (m, 4H), 7.02 (tt, *J* = 7.1, 1.2 Hz, 2H), 6.95 (dt, *J* = 8.8, 2.4 Hz, 2H), 4.43 (d, *J* = 2.8 Hz, 2H), 2.34 (qt, *J* = 7.2, 2.8 Hz, 1H), 1.36 (d, *J* = 7.2 Hz, 3H).

^{13}C NMR (101 MHz, $CDCl_3$) δ 147.5, 147.2, 135.9, 132.5, 130.7, 130.3, 129.4, 128.1, 124.7, 123.3, 123.1, 117.7, 91.5, 81.5, 16.6, 9.8.

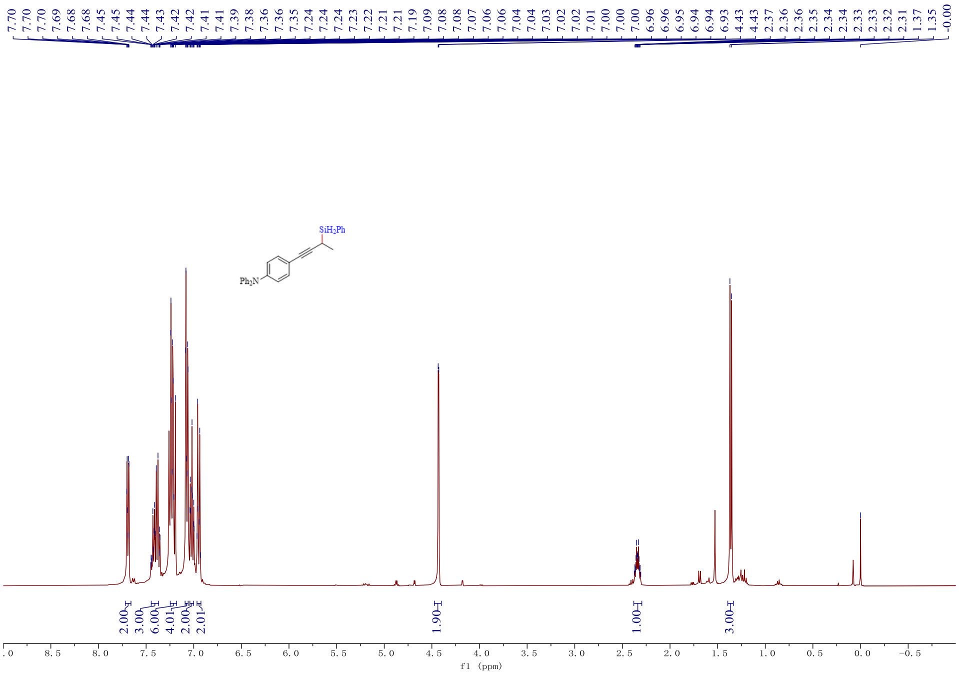

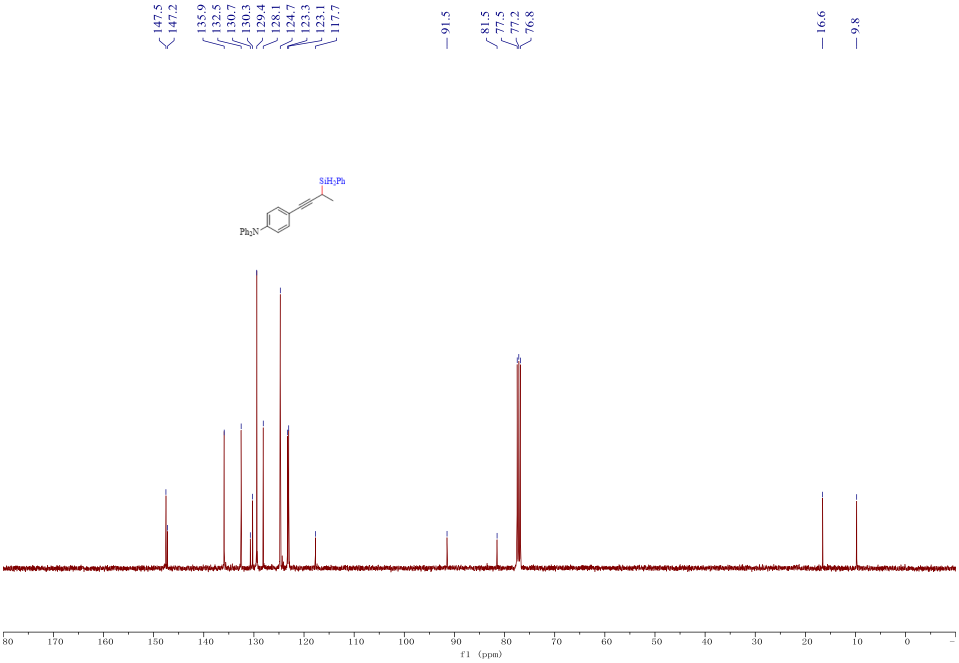

N-methyl-*N*-(4-(3-(phenylsilyl)but-1-yn-1-yl)phenyl)benzamide (5qa)

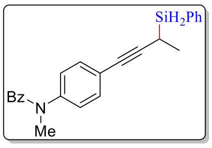

Prepared according to procedure 5 from **1q** (0.2 mmol, 52.3 mg) and **2a** (0.3 mmol, 32.5 mg). The product was isolated in 82% yield (60.8 mg) as colorless oil.

R*f*: 0.36 (ethyl acetate : petroleum ether = 1:7).

HRMS (ESI) (m/z): Calcd for $C_{24}H_{24}NOSi$ $[M+H]^+$: 370.1627, found: 370.1634.

^{1}H NMR (400 MHz, $CDCl_3$) δ 7.67 – 7.64 (m, 2H), 7.46 – 7.35 (m, 3H), 7.29 – 7.23 (m, 3H), 7.20 – 7.15 (m, 4H), 6.92 (d, *J* = 8.5 Hz, 2H), 4.41 (d, *J* = 2.8 Hz, 2H), 3.47 (s, 3H), 2.32 (qt, *J* = 7.2, 2.8 Hz, 1H), 1.34 (d, *J* = 7.2 Hz, 3H).

^{13}C NMR (101 MHz, $CDCl_3$) δ 170.7, 144.1, 135.8, 132.4, 130.4, 130.3, 129.9, 128.8, 128.2, 128.0, 126.7, 122.4, 93.4, 80.7, 38.4, 16.4, 9.7.

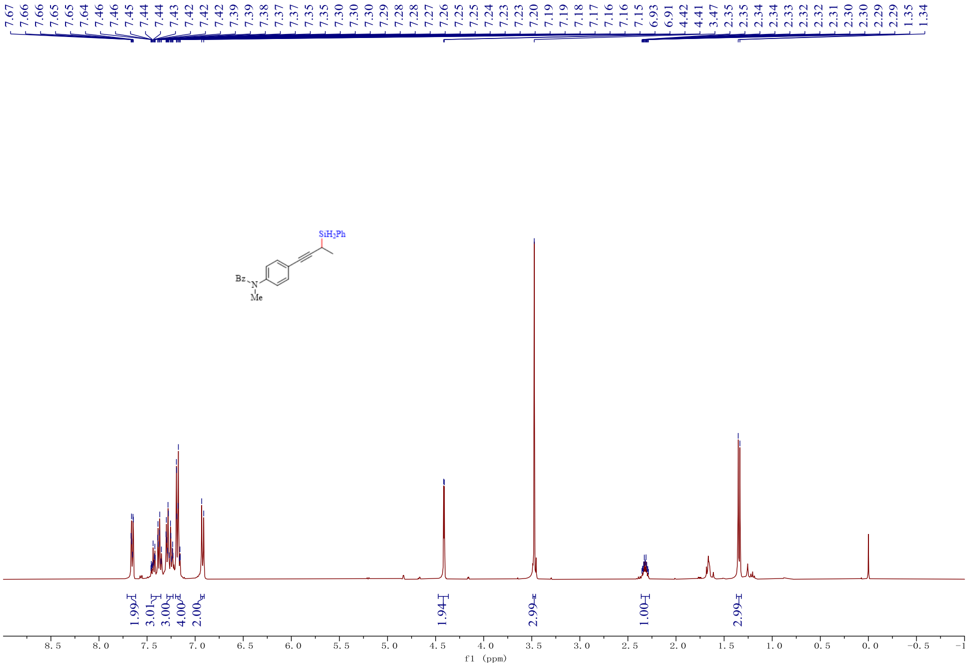

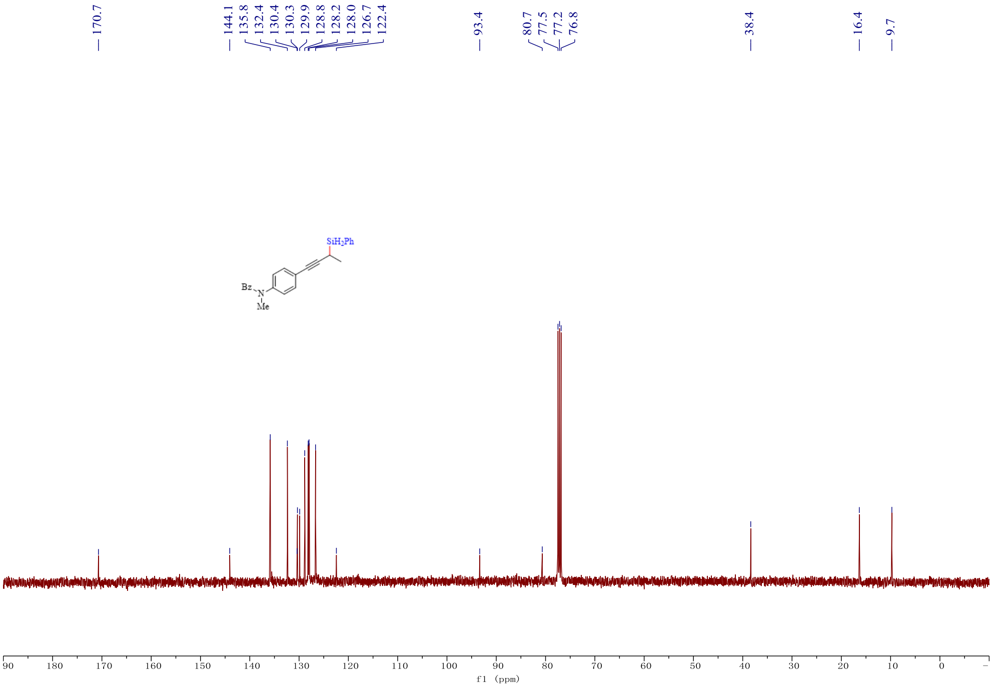

N-methyl-*N*-(2-(3-(phenylsilyl)but-1-yn-1-yl)phenyl)acetamide (5ra)

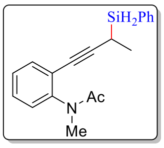

Prepared according to procedure 5 from **1r** (0.2 mmol, 39.9 mg) and **2a** (0.3 mmol, 32.5 mg). The product was isolated in 86% yield (52.8 mg) as colorless oil.

R*f*: 0.30 (ethyl acetate : petroleum ether = 1:5).

HRMS (ESI) (m/z): Calcd for $C_{19}H_{22}NOSi$ $[M+H]^+$: 308.1471, found: 308.1475.

^{1}H NMR (400 MHz, $CDCl_3$) δ 7.64 (dt, *J* = 7.9, 1.3 Hz, 2H), 7.47 – 7.33 (m, 4H), 7.35 – 7.22 (m, 2H), 7.15 (d, *J* = 7.7 Hz, 1H), 4.48 – 4.39 (m, 2H), 3.17 (s, 3H), 2.36 (m, 1H), 1.80 (d, *J* = 2.8 Hz, 3H), 1.36 (d, *J* = 7.2 Hz, 3H).

^{13}C NMR (101 MHz, $CDCl_3$) δ 170.7, 145.7, 145.7, 135.7, 133.2, 130.3, 130.2, 128.8, 128.2, 128.0, 123.5, 98.2, 77.2, 36.0, 22.1, 16.3, 9.9.

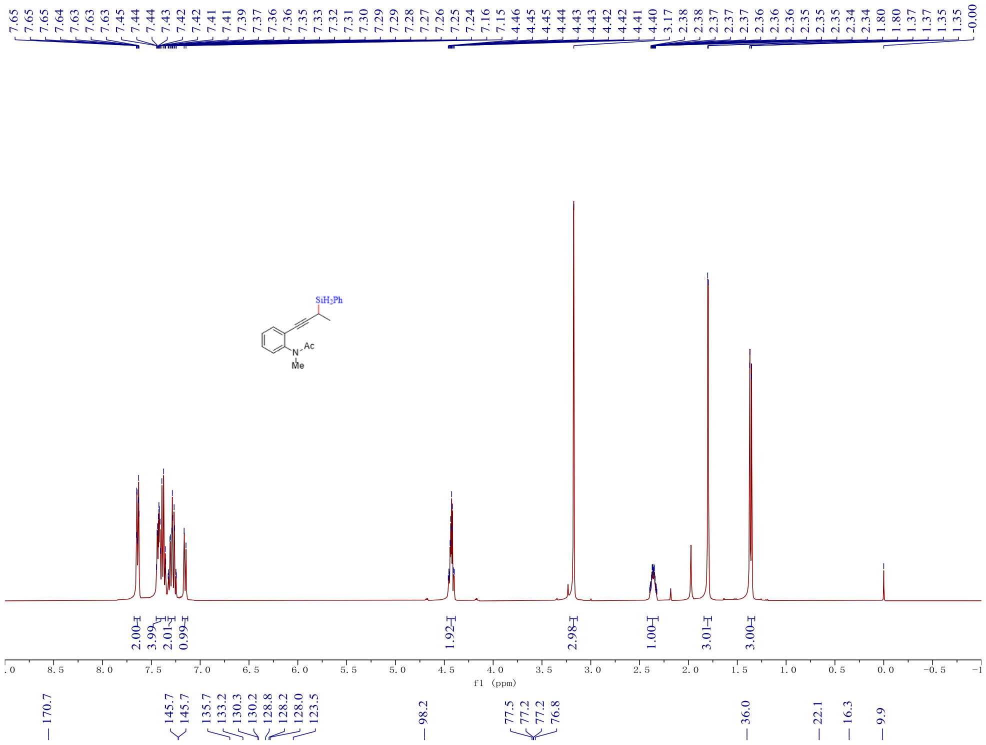

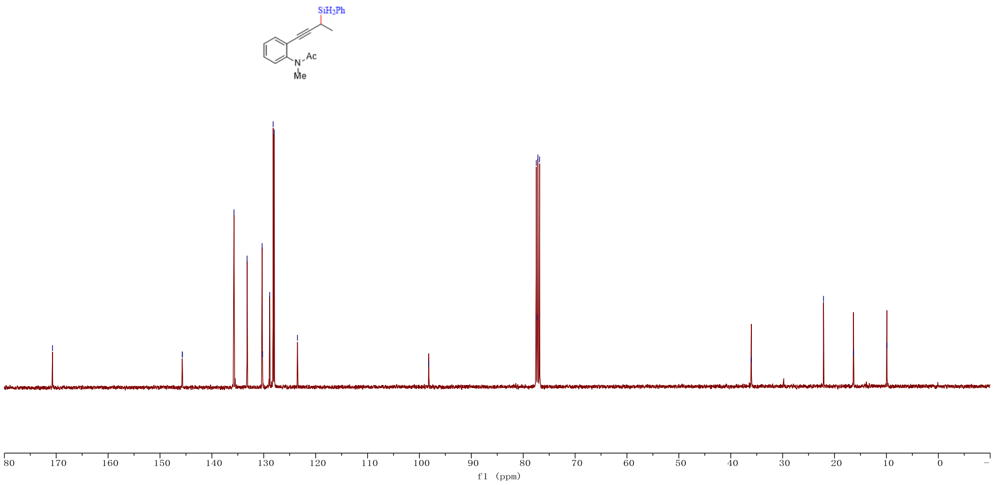

Methyl 4-(3-(phenylsilyl)but-1-yn-1-yl)benzoate (5sa)

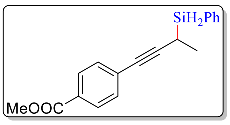

Prepared according to procedure 5 from **1s**(0.2 mmol, 37.3 mg) and **2a** (0.3 mmol, 32.5 mg). The product was isolated in 76% yield (44.7 mg) as colorless oil.

R*f*: 0.50 (ethyl acetate : petroleum ether = 1:20).

HRMS (ESI) (m/z): Calcd for $C_{18}H_{19}O_2Si$ $[M+H]^+$: 295.1154 found: 295.1151.

^{1}H NMR (400 MHz, $CDCl_3$) δ 7.98 – 7.90 (m, 2H), 7.69 – 7.66 (m, 2H), 7.45 (tt, 1H), 7.42 – 7.37 (m, 4H), 4.45 (d, *J* = 2.9 Hz, 2H), 3.91 (s, 3H), 2.38 (qt, *J* = 7.2, 2.8 Hz, 1H), 1.39 (d, *J* = 7.2 Hz, 3H).

^{13}C NMR (101 MHz, $CDCl_3$) δ 166.9, 135.9, 131.5, 130.4, 130.3, 129.5, 129.2, 128.8, 128.2, 96.1, 81.1, 52.3, 16.3, 10.0.

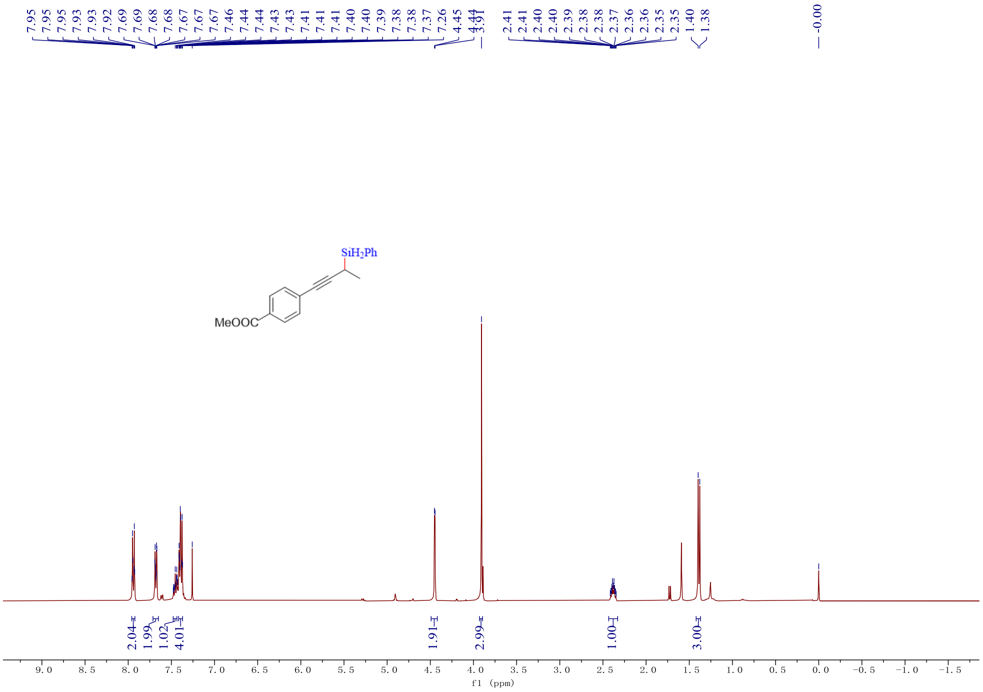

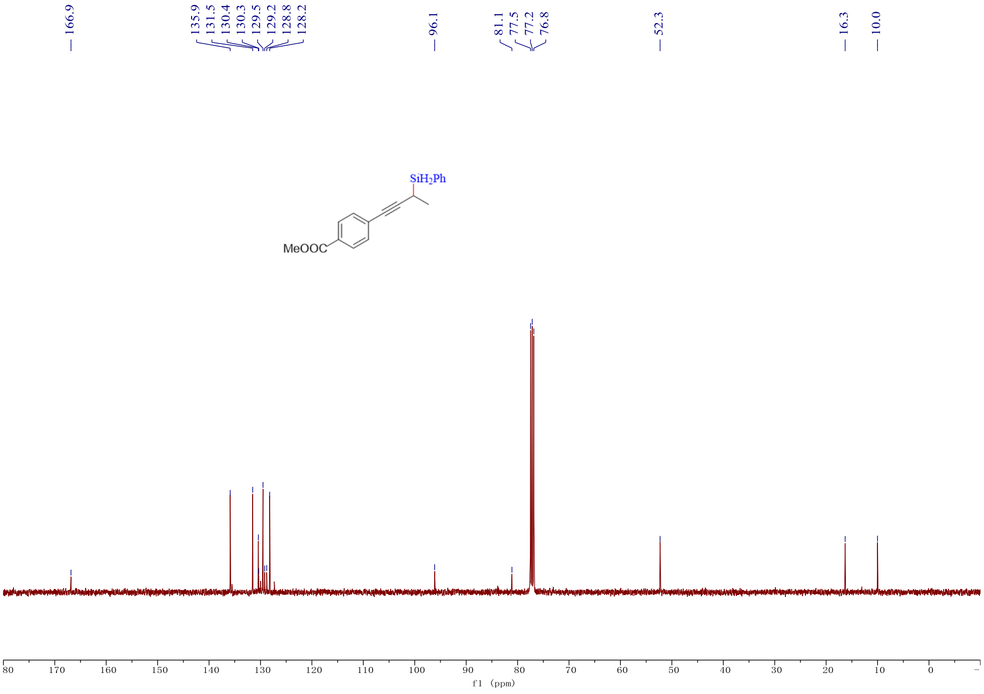

Phenyl(4-(4-(trifluoromethyl)phenyl)but-3-yn-2-yl)silane (5ta)

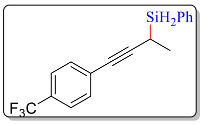

Prepared according to procedure 5 from **1t** (0.2 mmol, 39.2 mg) and **2a** (0.3 mmol, 32.5 mg). The product was isolated in 43% yield (25.9 mg) as colorless oil.

R*f*: 0.60 (petroleum ether).

HRMS (ESI) (m/z): Calcd for $C_{17}H_{15}F_3SiNa$ $[M+Na]^+$: 327.0793, found: 327.0838.

^{1}H NMR (400 MHz, $CDCl_3$) δ 7.68 (dt, *J* = 6.7, 1.5 Hz, 2H), 7.51 (d, *J* = 8.2 Hz, 2H), 7.48 – 7.37 (m, 5H), 4.45 (d, *J* = 2.8 Hz, 2H), 2.37 (qt, *J* = 7.2, 2.8 Hz, 1H), 1.39 (d, *J* = 7.2 Hz, 3H).

^{13}C NMR (101 MHz, $CDCl_3$) δ 135.9, 131.8, 130.4, 130.3, 129.2 (q, *J* = 32.6 Hz), 128.2, 125.2 (q, *J* = 3.8 Hz), 124.2 (q, *J* = 271.3 Hz), 95.5, 80.5, 16.3, 9.9.

^{19}F NMR (376 MHz, $CDCl_3$) δ -62.7.

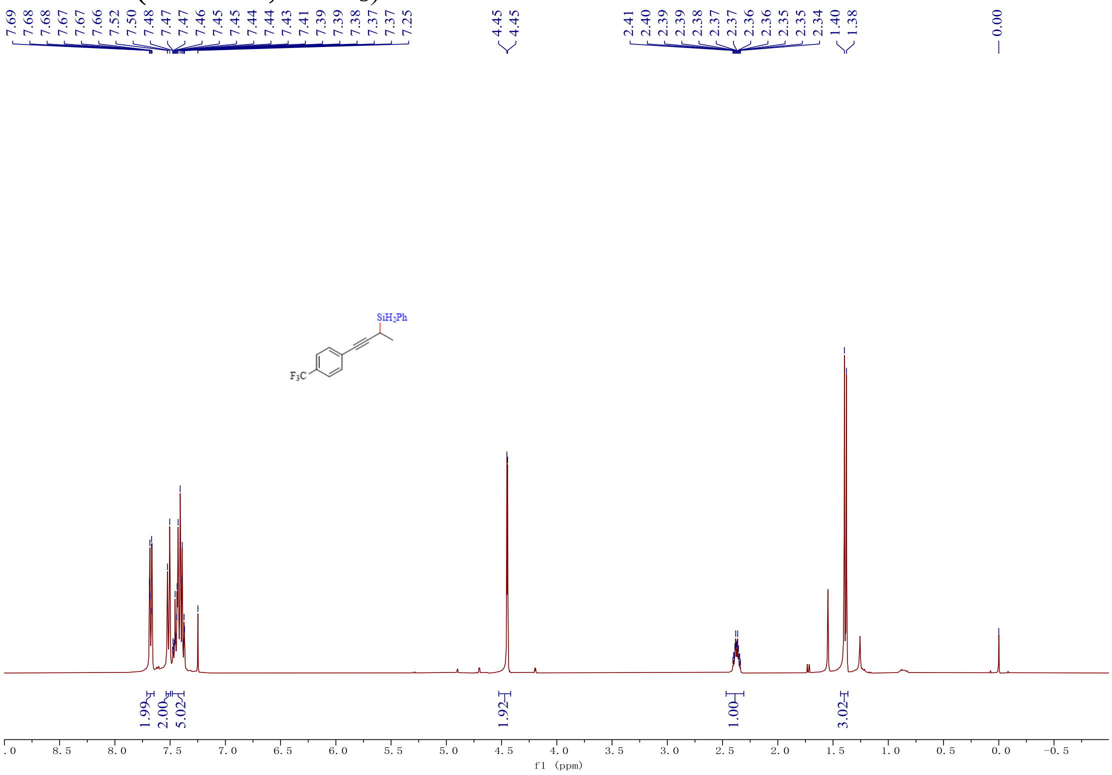

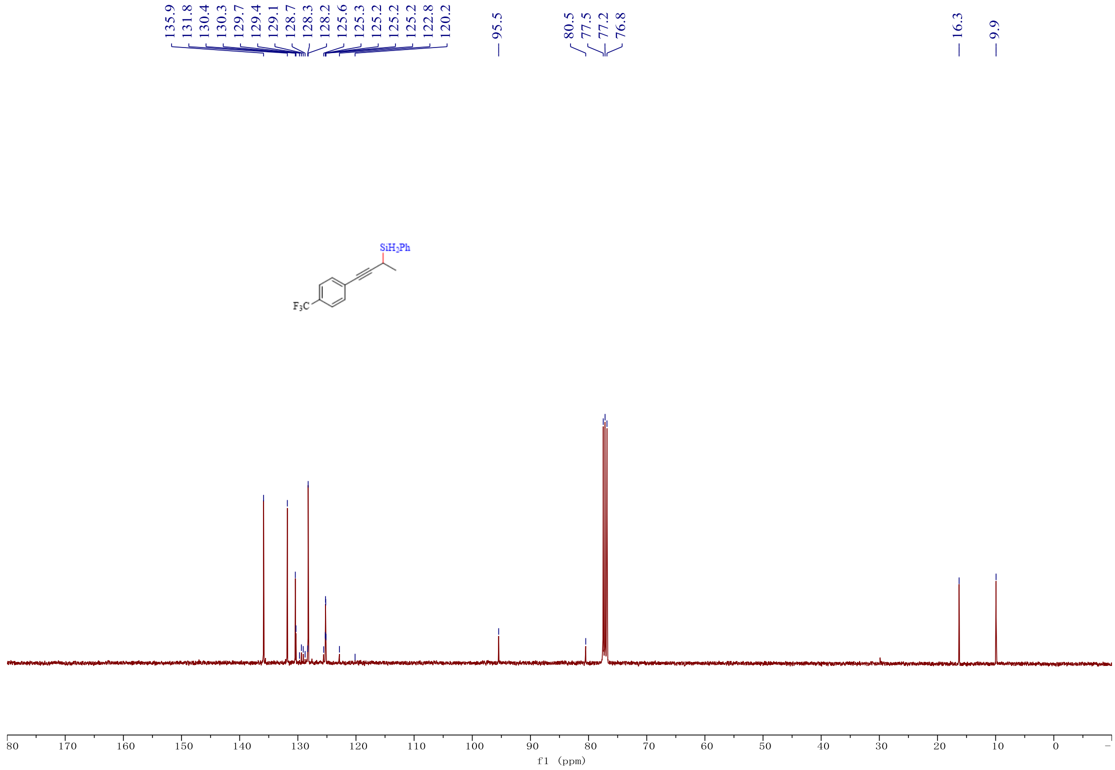
135.9
131.8
130.4
130.3
129.7
129.4
129.1
128.7
128.3
128.2
125.6
125.3
125.2
125.2
125.2
122.8
120.2
95.5
80.5
77.5
77.2
76.8
16.3
9.9
SiH2Ph
F3C
f1 (ppm)

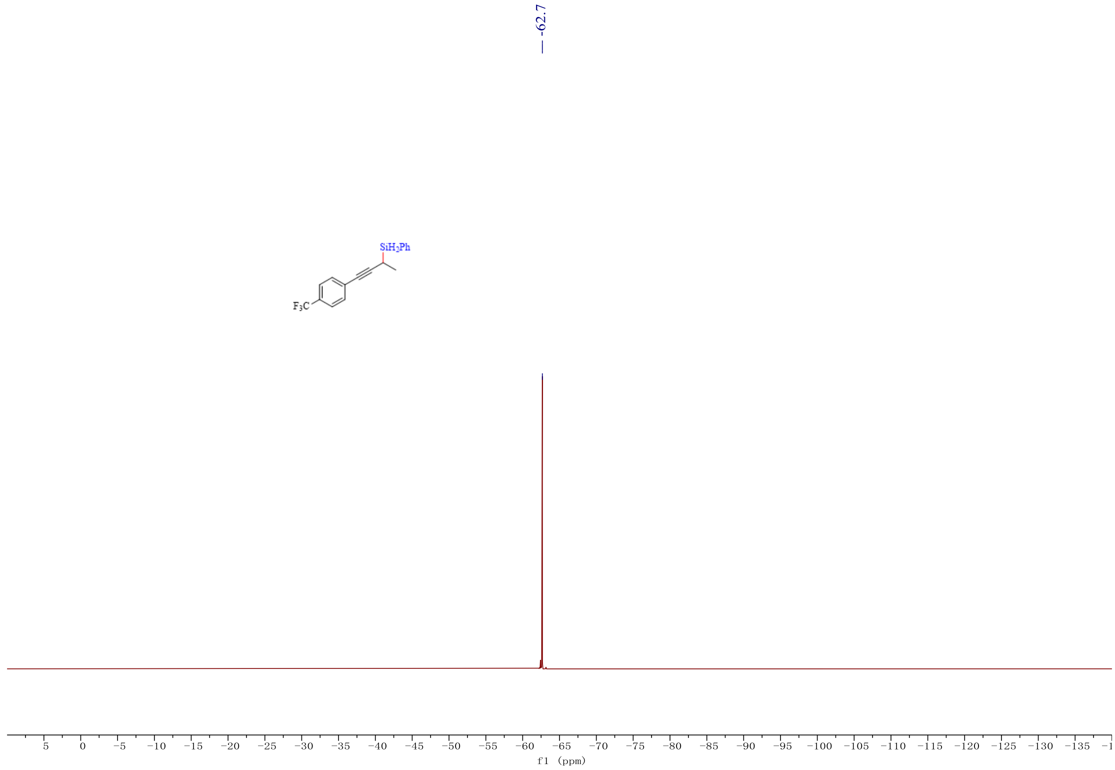
-62.7
SiH2Ph
F3C
f1 (ppm)

Phenyl(4-(3-(trifluoromethyl)phenyl)but-3-yn-2-yl)silane (5ua)

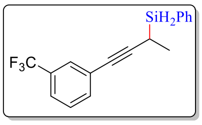

Prepared according to procedure 5 from **1u** (0.2 mmol, 39.2 mg) and **2a** (0.3 mmol, 32.5 mg). The product was isolated in 46% yield (28.0 mg) as colorless oil.

R*f*: 0.63 (petroleum ether).

HRMS (ESI) (m/z): Calcd for $C_{17}H_{15}F_3SiNa$ $[M+Na]^+$: 327.0793, found: 327.0820.

^{1}H NMR (400 MHz, $CDCl_3$) δ 7.68 (d, *J* = 6.6 Hz, 2H), 7.57 (s, 1H), 7.49 (d, *J* = 8.4 Hz, 2H), 7.45 (d, *J* = 7.4 Hz, 1H), 7.40 (d, *J* = 7.4 Hz, 2H), 7.37 (d, *J* = 8.6 Hz, 1H), 4.45 (d, *J* = 2.8 Hz, 2H), 2.41 – 2.33 (m, 1H), 1.39 (d, *J* = 7.2 Hz, 3H).

^{13}C NMR (101 MHz, $CDCl_3$) δ 135.9, 134.7, 130.9 (q, *J* = 32.3 Hz), 130.4, 130.3, 128.8, 128.4 (q, *J* = 3.9 Hz), 128.2, 125.3, 124.0 (q, *J* = 3.7 Hz), 122.6 (q, *J* = 272.5 Hz), 94.4, 80.3, 16.3, 9.8.

^{19}F NMR (376 MHz, $CDCl_3$) δ -62.92.

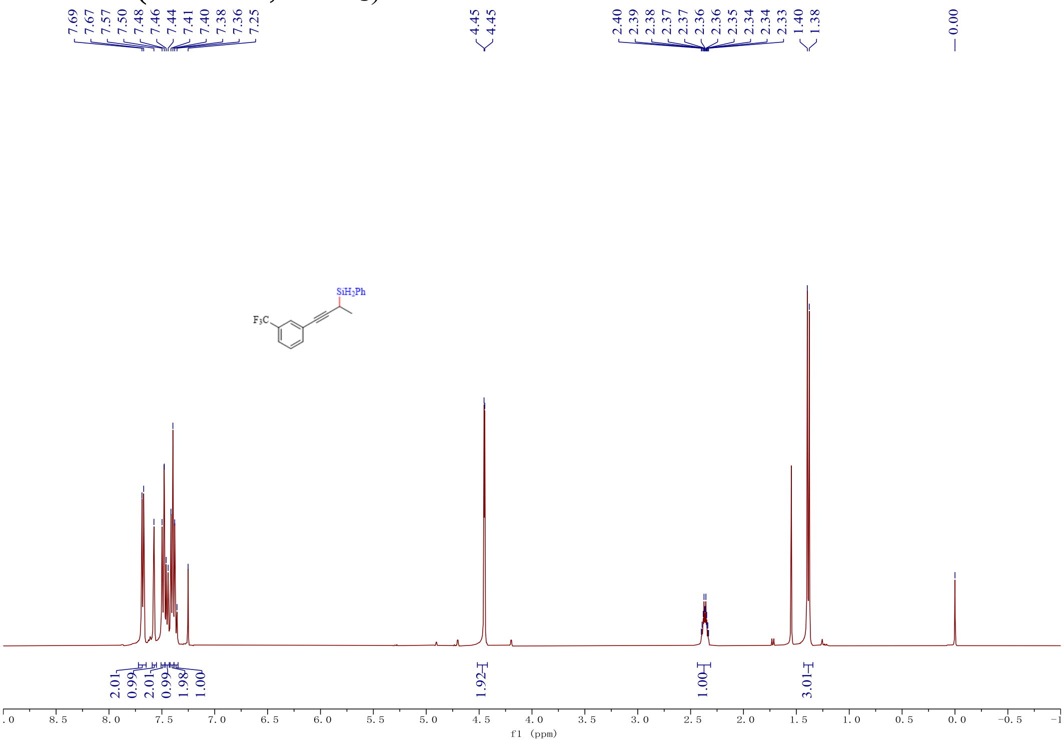

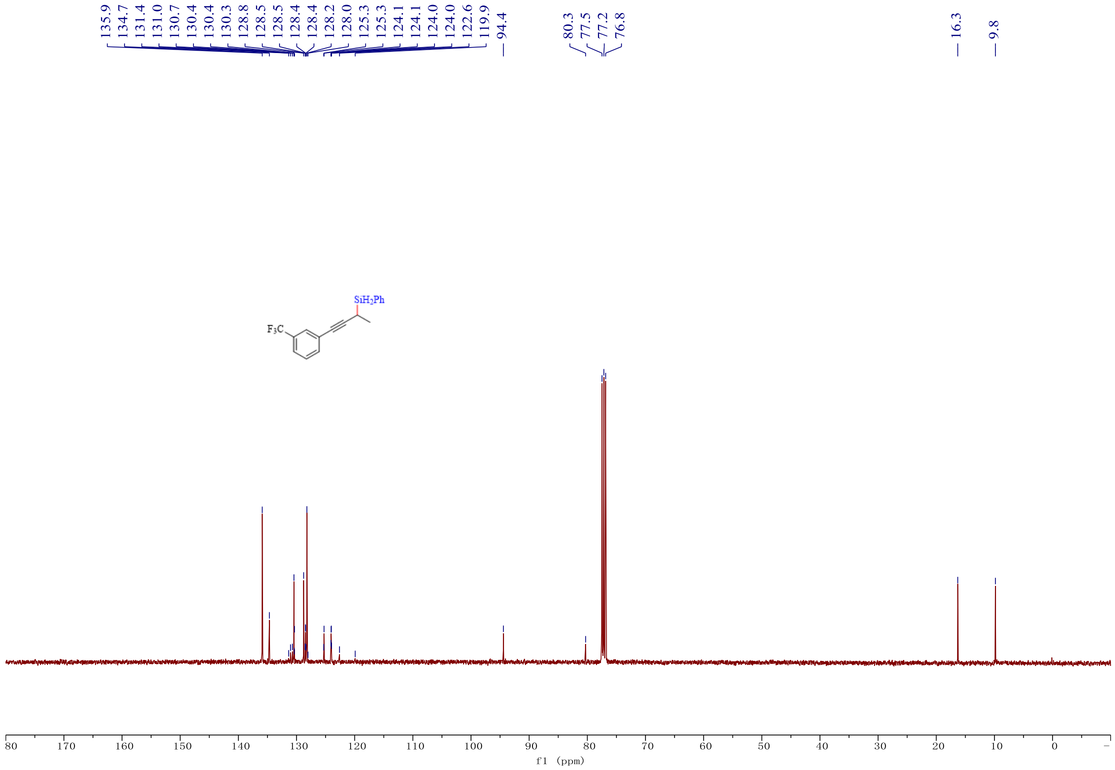
135.9
134.7
131.4
131.0
130.7
130.4
130.4
130.3
128.8
128.5
128.5
128.4
128.4
128.2
128.0
125.3
125.3
124.1
124.1
124.0
124.0
122.6
119.9
94.4
80.3
77.5
77.2
76.8
16.3
9.8
SiH2Ph
F3C
f1 (ppm)

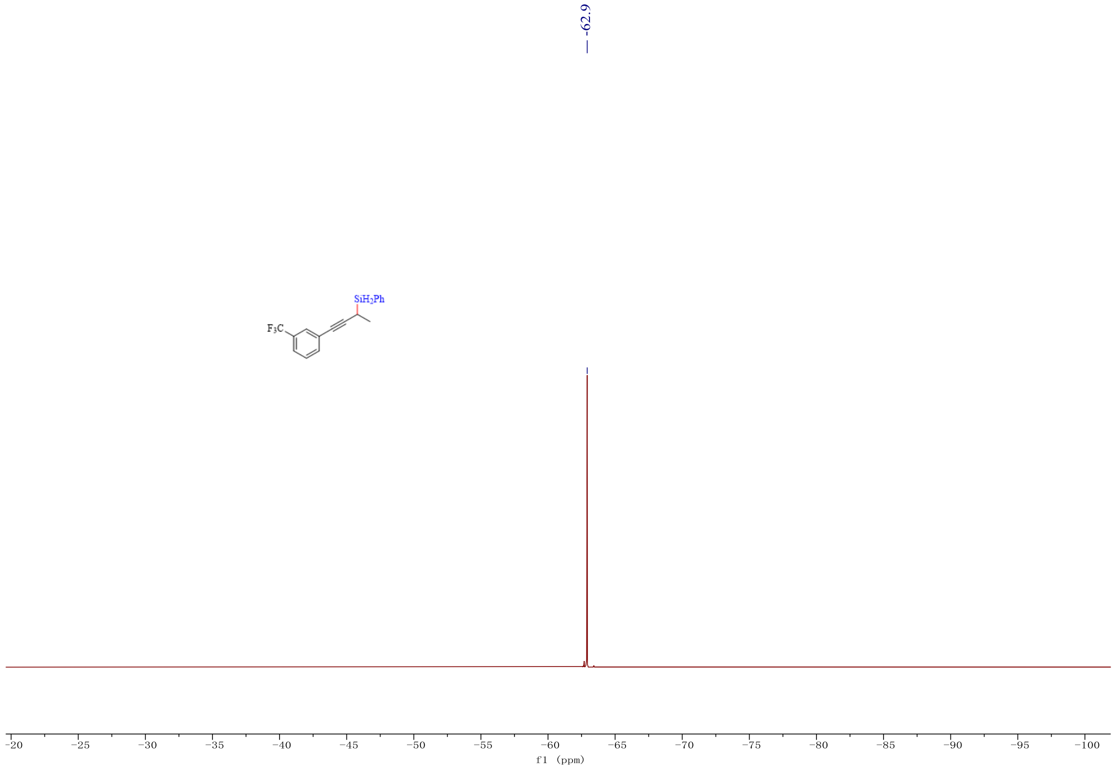
-62.9
SiH2Ph
F3C
f1 (ppm)

Phenyl(4-(thiophen-3-yl)but-3-yn-2-yl)silane (5va)

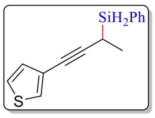

Prepared according to procedure 5 from **1v** (0.2 mmol, 26.8 mg) and **2a** (0.3 mmol, 32.5 mg). The product was isolated in 81% yield (39.1 mg) as colorless oil.

R*f*: 0.47 (petroleum ether).

HRMS (ESI) (m/z): Calcd for $C_{14}H_{14}SNaSi$ $[M+Na]^+$: 265.0483, found: 265.0492.

^{1}H NMR (400 MHz, $CDCl_3$) δ 7.68 (dd, *J* = 7.9, 1.5 Hz, 2H), 7.47 – 7.36 (m, 3H), 7.29 (dd, *J* = 3.0, 1.2 Hz, 1H), 7.21 (dd, *J* = 5.0, 3.0 Hz, 1H), 7.02 (dd, *J* = 5.0, 1.2 Hz, 1H), 4.43 (d, *J* = 2.8 Hz, 2H), 2.39 – 2.27 (m, 1H), 1.36 (d, *J* = 7.2 Hz, 3H).

^{13}C NMR (101 MHz, $CDCl_3$) δ 135.9, 130.6, 130.3, 130.2, 128.1, 127.5, 125.0, 123.2, 91.8, 76.5, 16.4, 9.7.

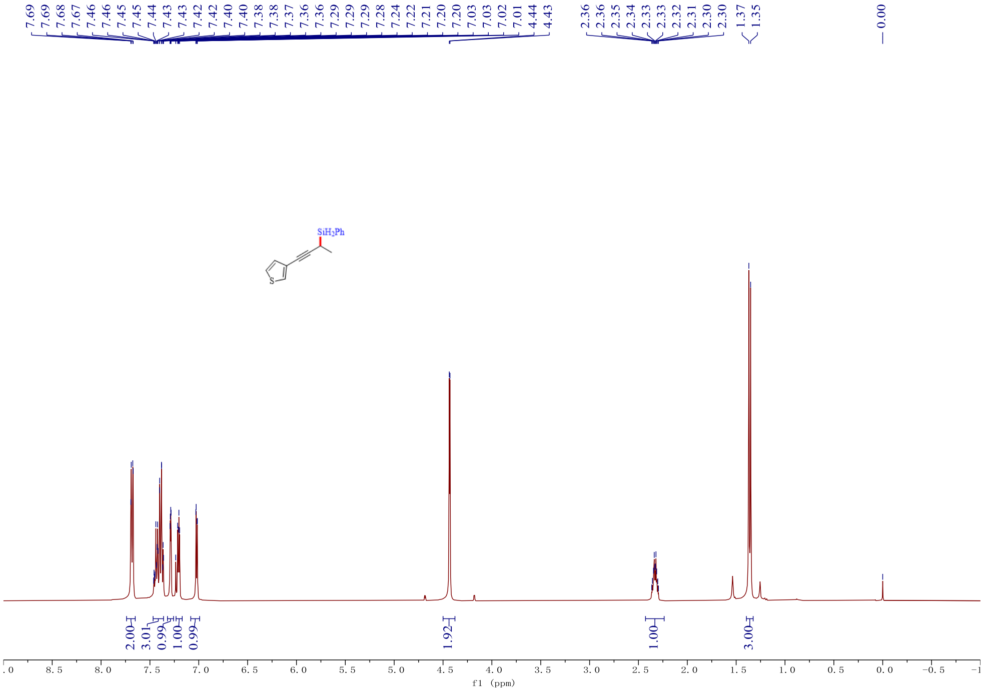

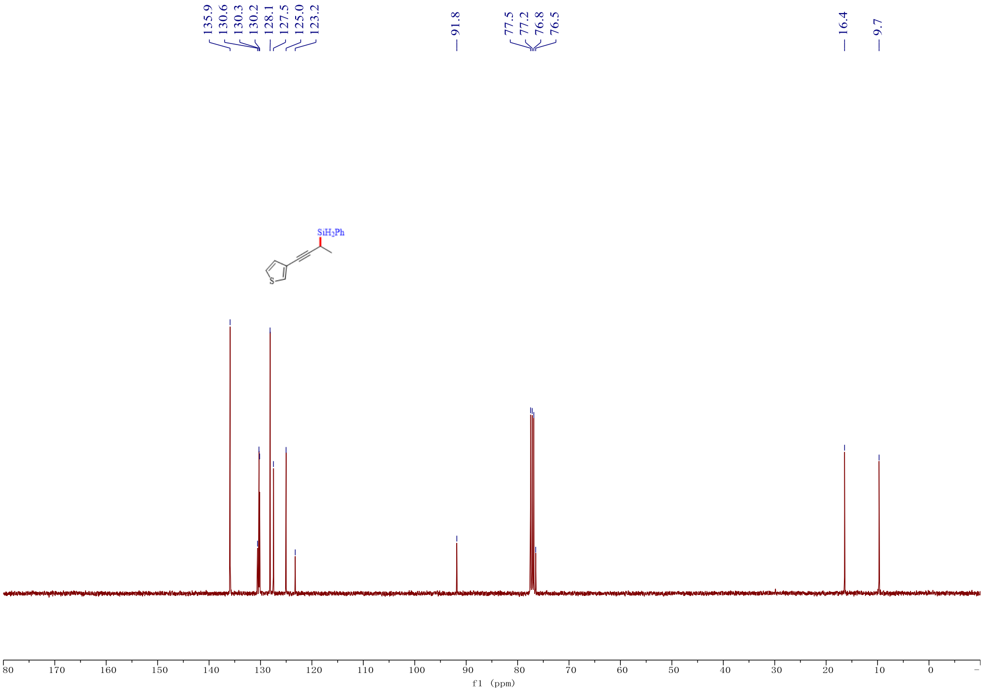

Phenyl(4-(4-vinylphenyl)but-3-yn-2-yl)silane (5wa)

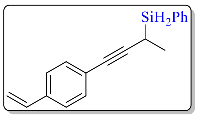

Prepared according to procedure 5 from **1w** (0.2 mmol, 30.9 mg) and **2a** (0.3 mmol, 32.5 mg). The product was isolated in 87% yield (45.9 mg) as colorless oil.

R*f*: 0.50 (petroleum ether).

HRMS (ESI) (m/z): Calcd for $C_{18}H_{19}Si$ $[M+H]^+$: 263.1256, found: 263.1255.

^{1}H NMR (400 MHz, $CDCl_3$) δ 7.71 – 7.67 (m, 2H), 7.46 – 7.35 (m, 3H), 7.30 (s, 4H), 6.67 (dd, *J* = 17.6, 10.9 Hz, 1H), 5.72 (d, *J* = 17.6 Hz, 1H), 5.24 (d, *J* = 10.9 Hz, 1H), 4.44 (d, *J* = 2.9 Hz, 2H), 2.36 (qt, *J* = 7.2, 2.9 Hz, 1H), 1.37 (d, *J* = 7.2 Hz, 3H).

^{13}C NMR (101 MHz, $CDCl_3$) δ 136.7, 136.5, 135.9, 131.8, 130.6, 130.3, 128.2, 126.1, 123.7, 114.3, 93.2, 81.6, 16.4, 9.9.

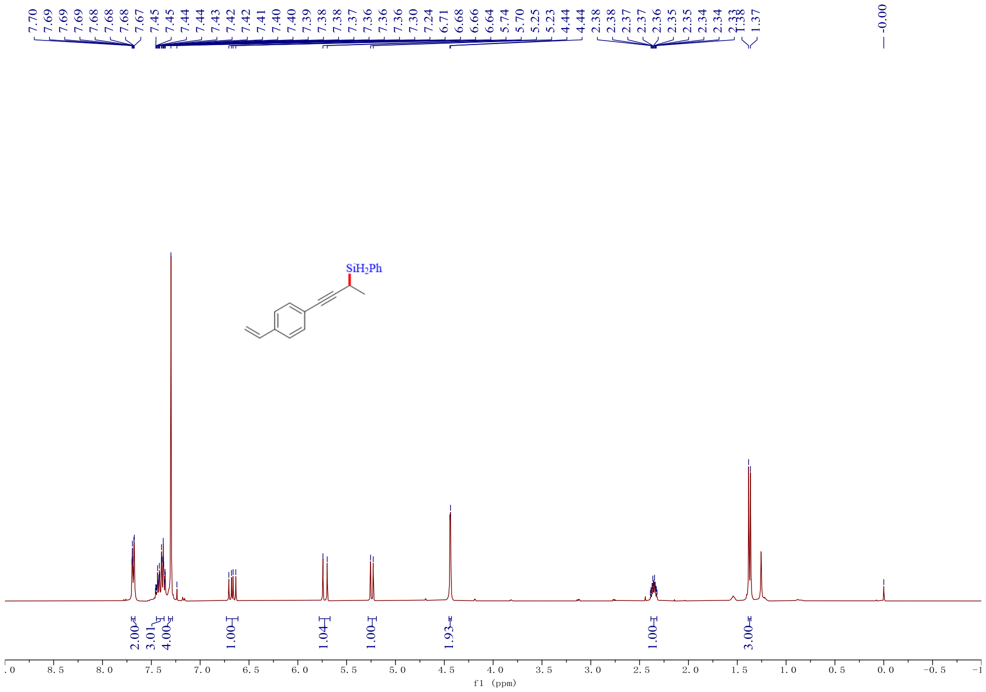

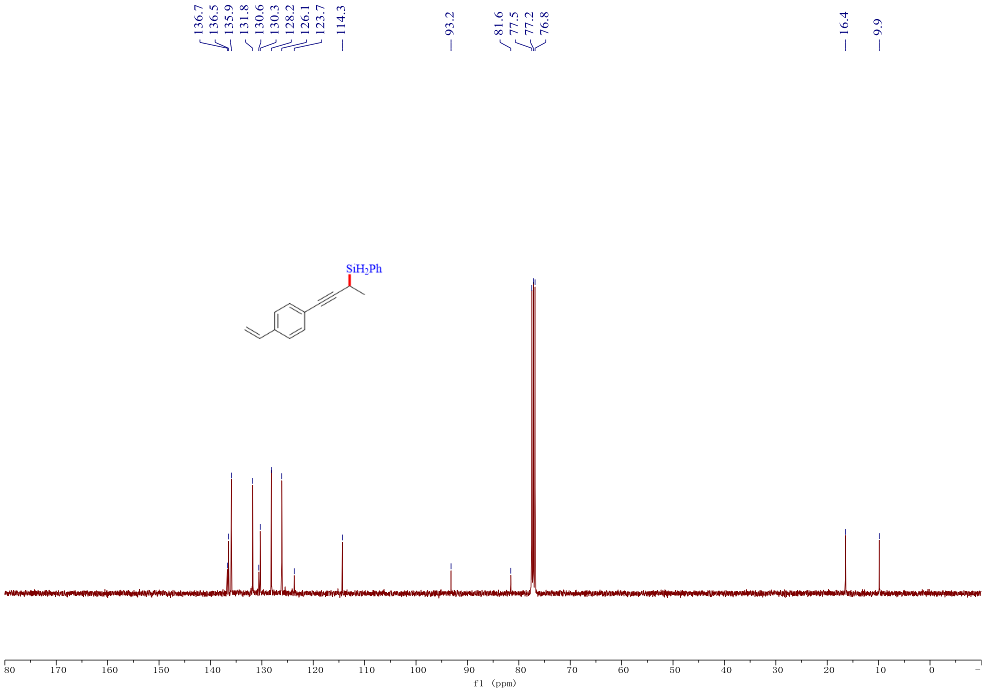

Phenyl(4-(4-(prop-1-en-2-yl)phenyl)but-3-yn-2-yl)silane (5xa)

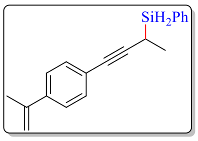

Prepared according to procedure 5 from **1x** (0.2 mmol, 33.6 mg) and **2a** (0.3 mmol, 32.5 mg). The product was isolated in 80% yield (44.0 mg) as colorless oil.

R*f*: 0.45 (petroleum ether).

HRMS (ESI) (m/z): Calcd for $C_{19}H_{21}Si$ $[M+H]^+$: 277.1413, found: 277.1406.

^{1}H NMR (400 MHz, $CDCl_3$) δ 7.69 (d, *J* = 6.9 Hz, 2H), 7.47 – 7.35 (m, 5H), 7.30 (d, *J* = 8.2 Hz, 2H), 5.37 (s, 1H), 5.09 (s, 1H), 4.44 (d, *J* = 2.8 Hz, 2H), 2.42 – 2.30 (m, 1H), 2.13 (s, 3H), 1.38 (d, *J* = 7.3 Hz, 3H).

^{13}C NMR (101 MHz, $CDCl_3$) δ 142.8, 140.2, 135.9, 131.5, 130.6, 130.3, 128.1, 125.4, 123.3, 112.9, 93.0, 81.5, 21.8, 16.5, 9.9.

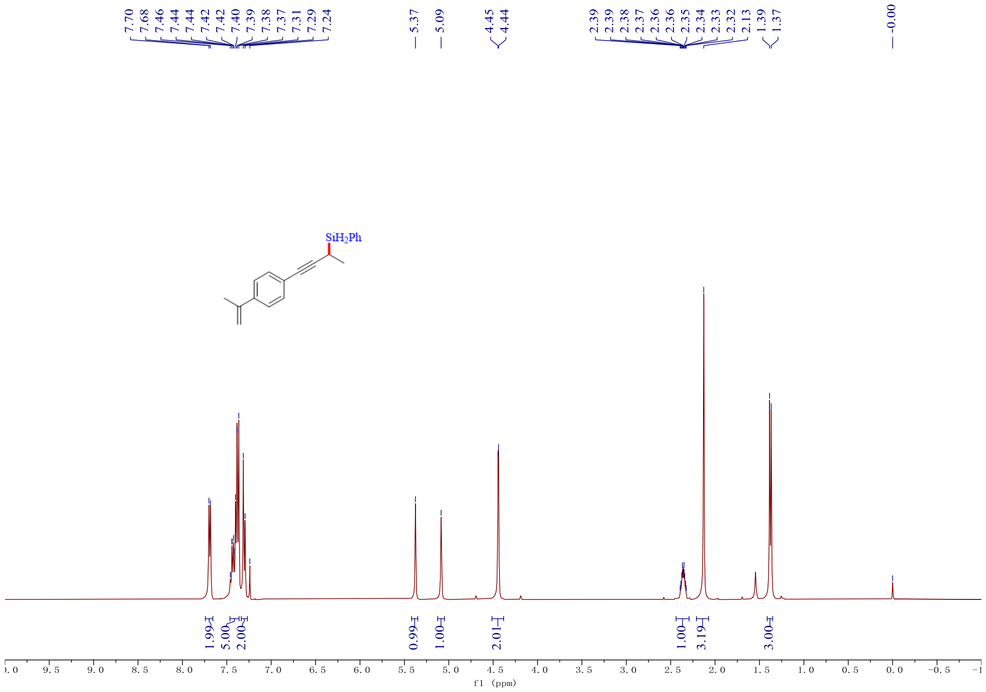

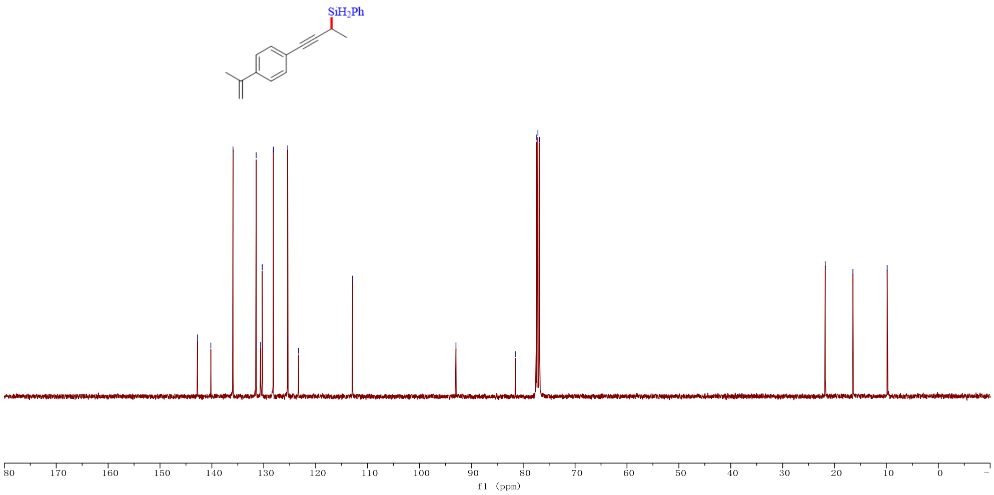

Trimethyl((4-(3-(phenylsilyl)but-1-yn-1-yl)phenyl)ethynyl)silane (5ya)

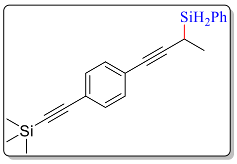

Prepared according to procedure 5 from **1y** (0.2 mmol, 44.9 mg) and **2a** (0.3 mmol, 32.5 mg). The product was isolated in 63% yield (41.7 mg) as colorless oil.

R*f*: 0. 51 (petroleum ether).

HRMS (ESI) (m/z): Calcd for $C_{21}H_{25}Si_2$ [M+H]$^+$: 333.1495, found: 333.1502.

^{1}H NMR (400 MHz, $CDCl_3$) δ 7.67 (d, *J* = 7.2 Hz, 2H), 7.46 – 7.34 (m, 5H), 7.25 (d, *J* = 8.1 Hz, 2H), 4.43 (d, *J* = 2.8 Hz, 2H), 2.35 (qt, *J* = 7.2, 2.8 Hz, 1H), 1.37 (d, *J* = 7.2 Hz, 3H), 0.24 (s, 9H).

^{13}C NMR (101 MHz, $CDCl_3$) δ 135.9, 131.9, 131.4, 130.5, 130.4, 128.2, 124.5, 122.1, 105.0, 95.7, 94.7, 81.4, 16.4, 9.9, 0.1.

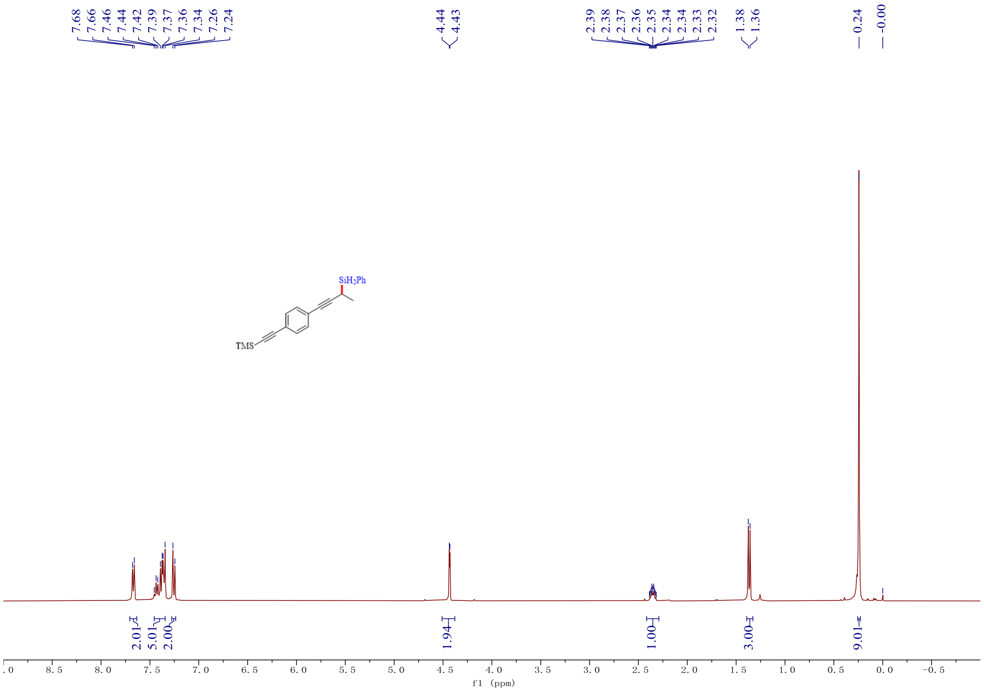

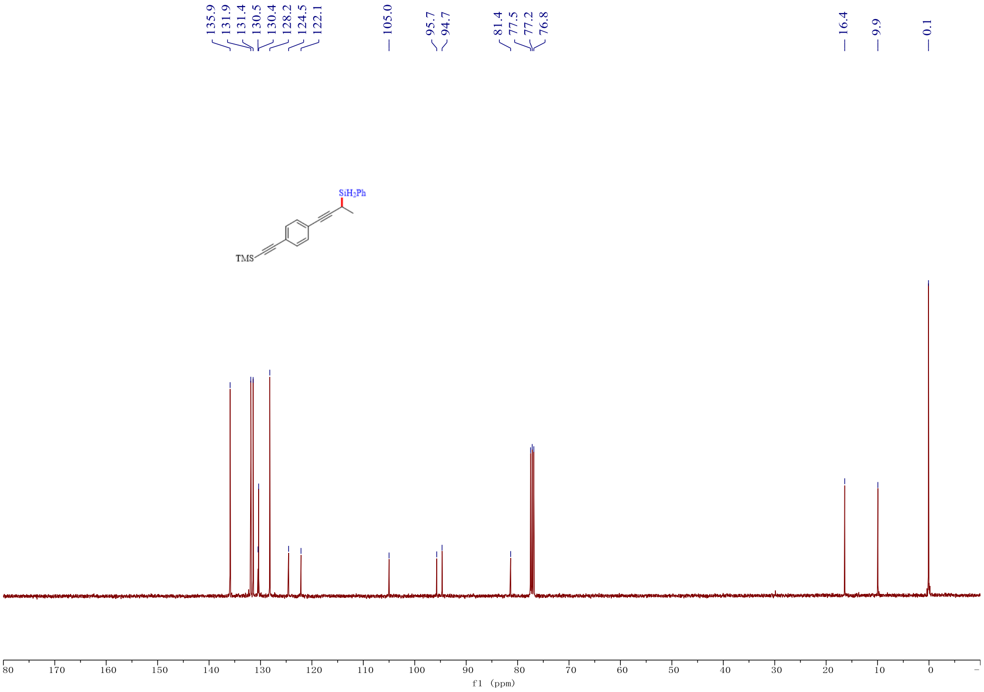

(*E*)-Phenyl(6-phenylhex-5-en-3-yn-2-yl)silane (5za)

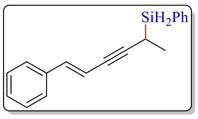

Prepared according to procedure 5 from **1z** (0.2 mmol, 30.9 mg) and **2a** (0.3 mmol, 32.5 mg). The product was isolated in 73% yield (38.0 mg) as colorless oil.

R*f*: 0.48 (petroleum ether).

HRMS (ESI) (m/z): Calcd for $C_{18}H_{19}Si$ $[M+H]^+$: 263.1256, found: 263.1257.

^{1}H NMR (400 MHz, $CDCl_3$) δ 7.67 (dt, *J* = 6.7, 1.6 Hz, 2H), 7.46 – 7.23 (m, 8H), 6.82 (d, *J* = 16.2 Hz, 1H), 6.17 (dd, *J* = 16.2, 2.4 Hz, 1H), 4.42 (m, 2H), 2.32 (qt, *J* = 7.2, 2.8 Hz, 1H), 1.34 (d, *J* = 7.2 Hz, 3H).

^{13}C NMR (101 MHz, $CDCl_3$) δ 139.8, 136.8, 135.9, 130.6, 130.3, 128.8, 128.3, 128.2, 126.2, 109.2, 95.0, 80.9, 16.5, 10.1.

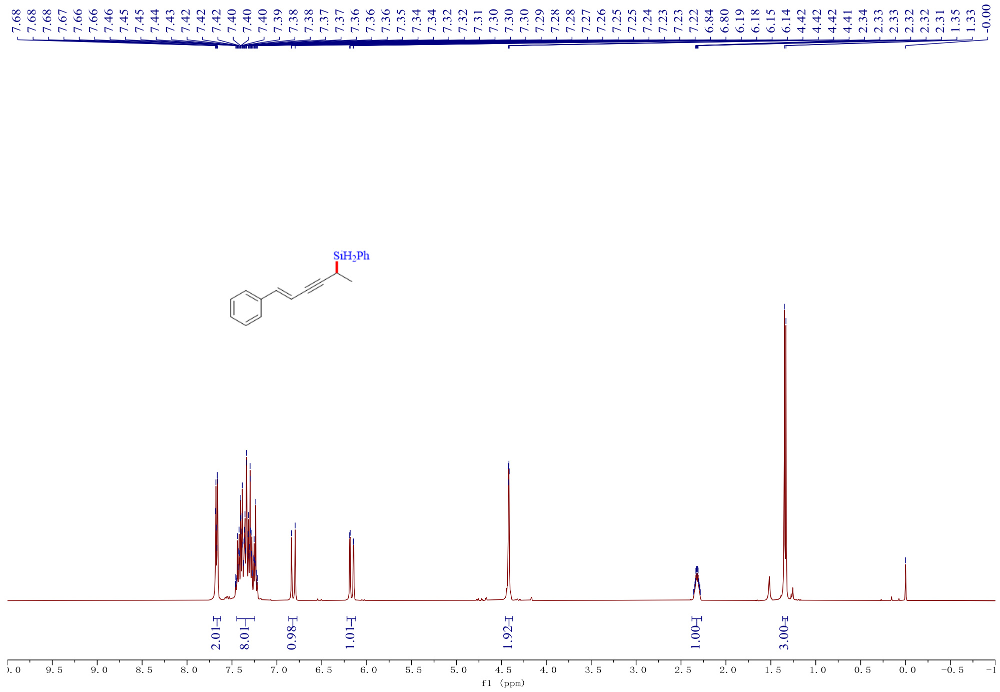

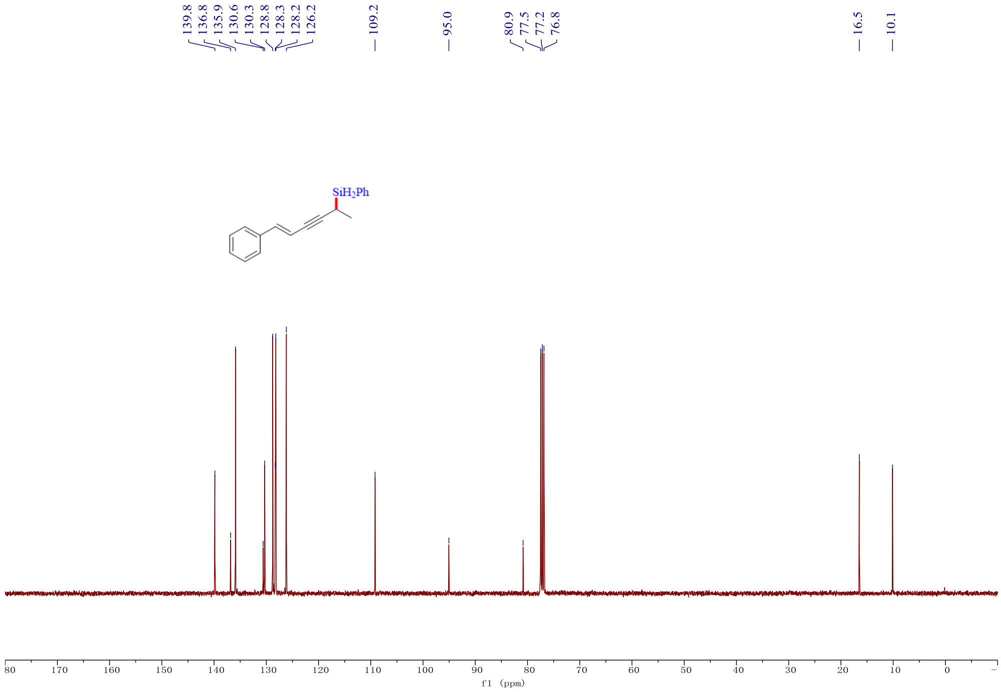

(*E*)-(4-(Cyclooct-1-en-1-yl)but-3-yn-2-yl)(phenyl)silane (5aaa)

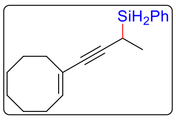

Prepared according to procedure 5 from **1aa** (0.2 mmol, 32.0 mg) and **2a** (0.3 mmol, 32.5 mg). The product was isolated in 44% yield (23.6 mg) as colorless oil.

R*f*: 0.65 (petroleum ether).

HRMS (ESI) (m/z): Calcd for $C_{18}H_{24}NaSi$ $[M+Na]^+$: 291.1545, found: 291.1566.

^{1}H NMR (400 MHz, $CDCl_3$) δ 7.71 – 7.59 (m, 2H), 7.47 – 7.32 (m, 3H), 5.95 (t, *J* = 8.3 Hz, 1H), 4.45 – 4.30 (m, 2H), 2.30 – 2.18 (m, 3H), 2.17 – 2.11 (m, 2H), 1.61 – 1.46 (m, 8H), 1.30 (d, *J* = 7.3 Hz, 3H).

^{13}C NMR (101 MHz, $CDCl_3$) δ 135.9, 135.8, 130.9, 130.2, 128.0, 124.3, 88.9, 84.0, 30.4, 30.0, 28.5, 27.1, 26.6, 26.0, 16.6, 9.6.

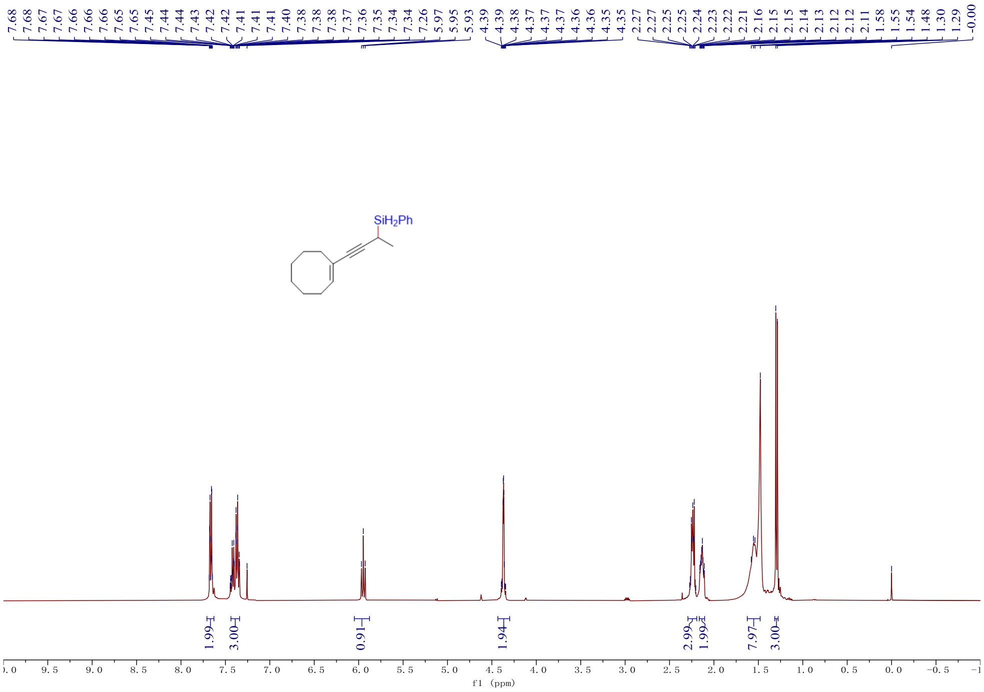

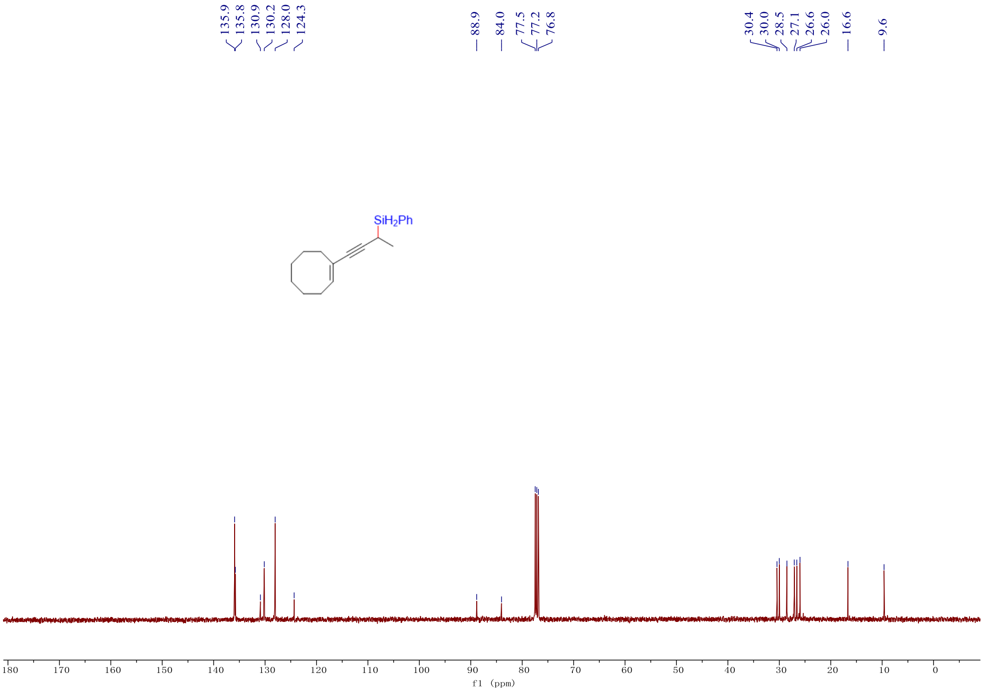

Phenyl(6-((tetrahydro-2*H*-pyran-2-yl)oxy)hex-3-yn-2-yl)silane (5aba)

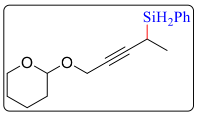

Prepared according to procedure 5 from **1ab** (0.2 mmol, 30.9 mg) and **2a** (0.3 mmol, 32.5 mg). The product was isolated in 51% yield (27.8 mg) as colorless oil.

R*f*: 0.48 (petroleum ether).

HRMS (ESI) (m/z): Calcd for $C_{17}H_{25}O_2Si$ $[M+H]^+$: 289.1624, found: 289.1596.

^{1}H NMR (400 MHz, $CDCl_3$) δ 7.64 (d, *J* = 6.6 Hz, 2H), 7.45 – 7.35 (m, 3H), 4.78 (s, 1H), 4.37 (d, *J* = 2.8 Hz, 2H), 4.29 (d, *J* = 15.0 Hz, 1H), 4.23 (d, *J* = 15.0 Hz, 1H), 3.83 (t, *J* = 10.1 Hz, 1H), 3.50 (dt, *J* = 10.5, 4.8 Hz, 1H), 2.22 – 2.14 (m, 1H), 1.88 – 1.78 (m, 1H), 1.75 – 1.67 (m, 1H), 1.63 – 1.49 (m, 4H), 1.29 (d, *J* = 7.3 Hz, 3H).

^{13}C NMR (101 MHz, $CDCl_3$) δ 135.8, 130.5, 130.3, 128.1, 96.5, 88.7, 76.7, 62.1, 54.8, 54.7, 30.5, 25.5, 19.3, 16.3, 9.0.

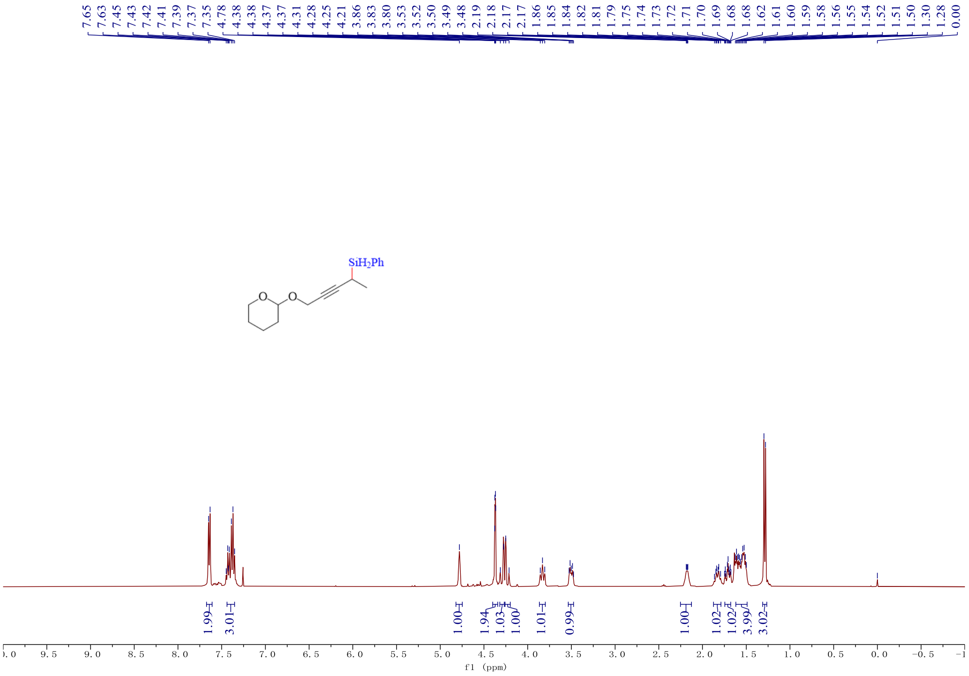

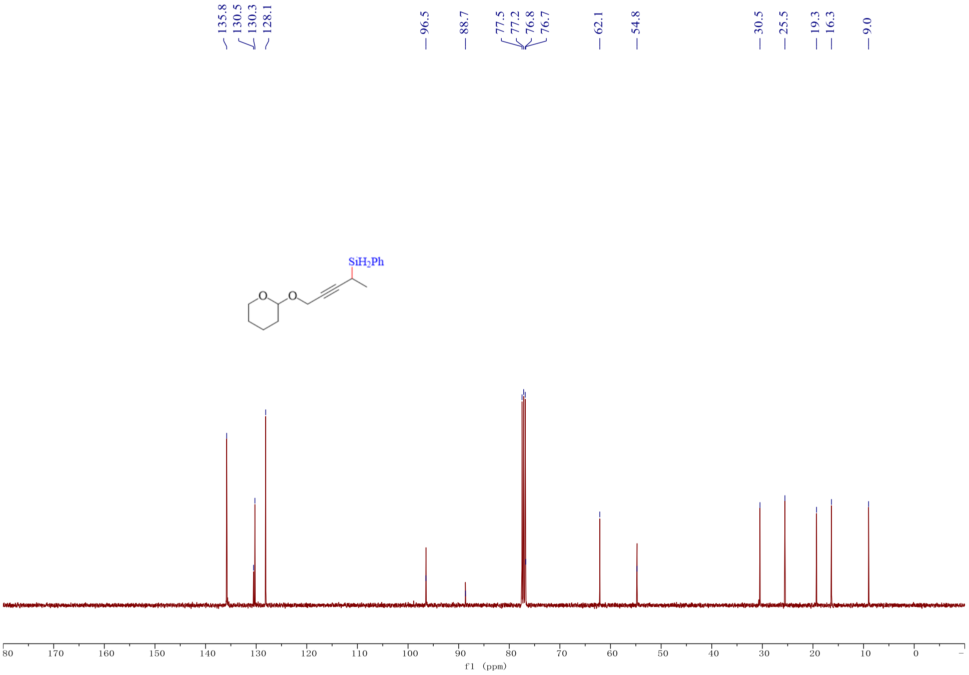

(4-Phenylbut-3-yn-2-yl)(o-tolyl)silane (5ab)

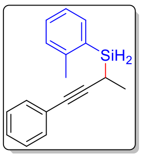

Prepared according to procedure 5 from **1a** (0.2 mmol, 25.6 mg) and **2b** (0.3 mmol, 36.7 mg). The product was isolated in 70% yield (35.2 mg) as colorless oil.

R*f*: 0.50 (petroleum ether).

HRMS (ESI) (m/z): Calcd for $C_{17}H_{19}Si$ $[M+H]^+$: 251.1256, found: 251.1262.

^{1}H NMR (400 MHz, $CDCl_3$) δ 7.63 (d, *J* = 6.9 Hz, 1H), 7.36 – 7.30 (m, 3H), 7.26 – 7.17 (m, 5H), 4.52 – 4.46 (m, 2H), 2.51 (s, 3H), 2.39 (qt, *J* = 7.2, 2.8 Hz, 1H), 1.39 (d, *J* = 7.2 Hz, 3H).

^{13}C NMR (101 MHz, $CDCl_3$) δ 144.4, 137.2, 131.6, 130.7, 130.1, 129.7, 128.2, 127.5, 125.3, 124.4, 92.7, 81.4, 23.1, 16.9, 9.5.

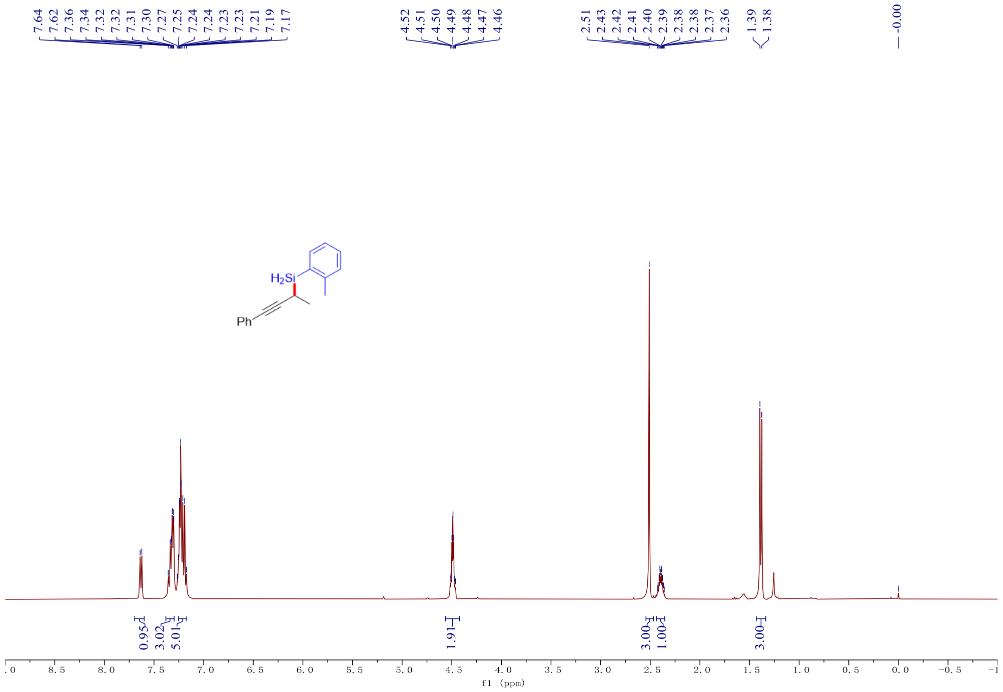

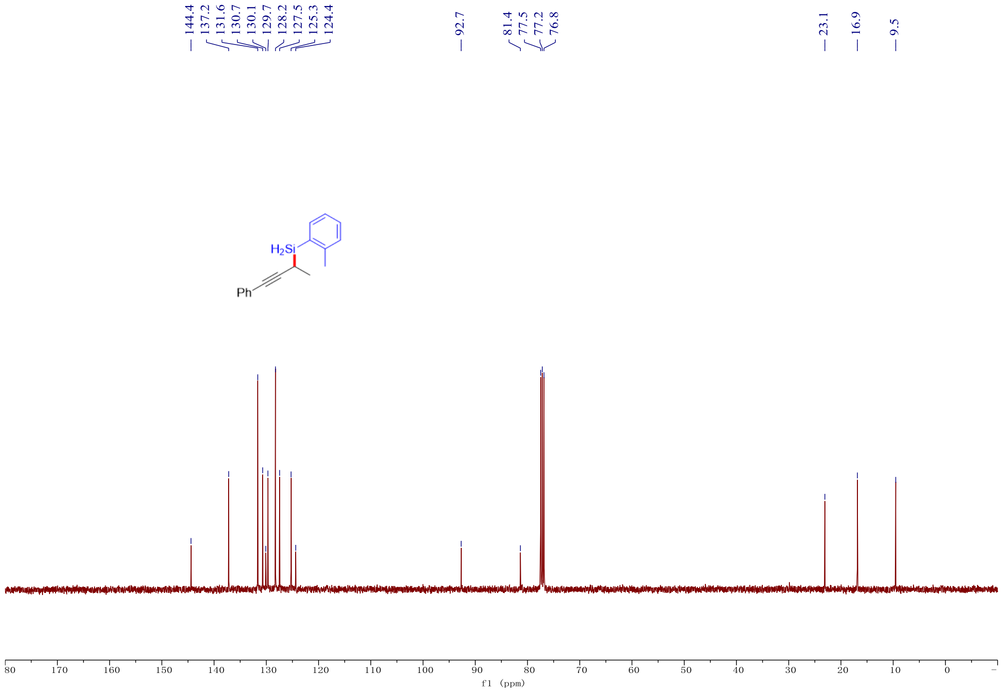

(4-Phenylbut-3-yn-2-yl)(*m*-tolyl)silane (5ac)

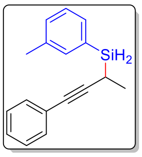

Prepared according to procedure 5 from **1a** (0.2 mmol, 25.6 mg) and **2c** (0.3 mmol, 36.7 mg). The product was isolated in 88% yield (44.0 mg) as colorless oil.

R_f: 0.60 (petroleum ether).

HRMS (ESI) (m/z): Calcd for $C_{17}H_{19}Si$ $[M+H]^+$: 251.1256, found: 251.1263.

^{1}H NMR (400 MHz, $CDCl_3$) δ 7.51 (s, 1H), 7.49 (d, *J* = 6.8 Hz, 1H), 7.38 – 7.33 (m, 2H), 7.29 – 7.21 (m, 5H), 4.42 (d, *J* = 2.8 Hz, 2H), 2.41 – 2.29 (m, 4H), 1.37 (d, *J* = 7.2 Hz, 3H).

^{13}C NMR (101 MHz, $CDCl_3$) δ 137.5, 136.6, 132.9, 131.6, 131.1, 130.4, 128.3, 128.1, 127.5, 124.4, 92.6, 81.6, 21.6, 16.5, 9.8.

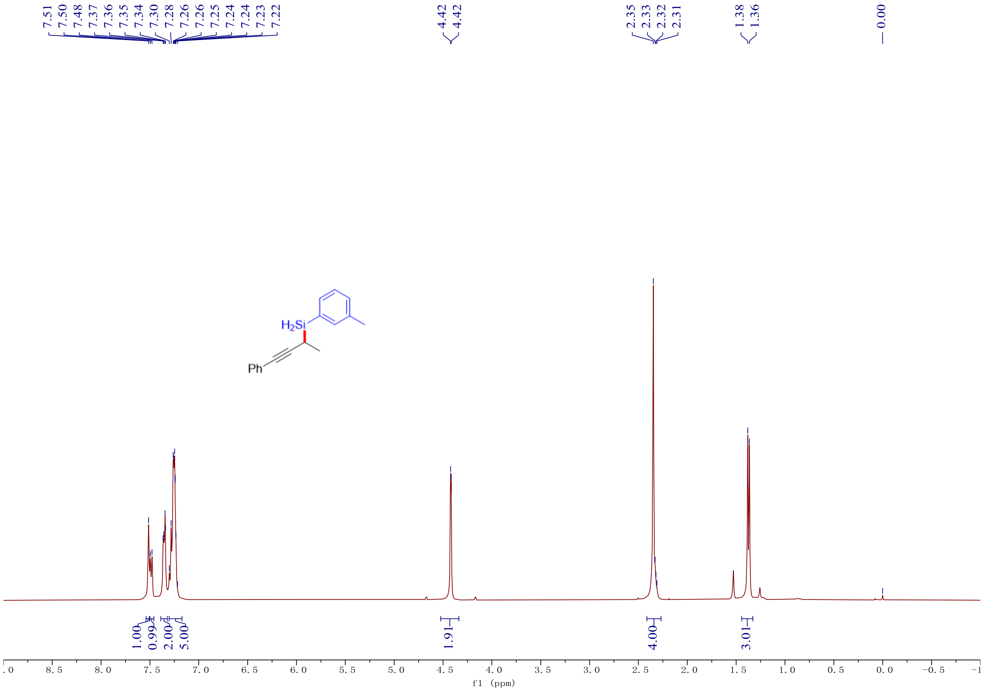

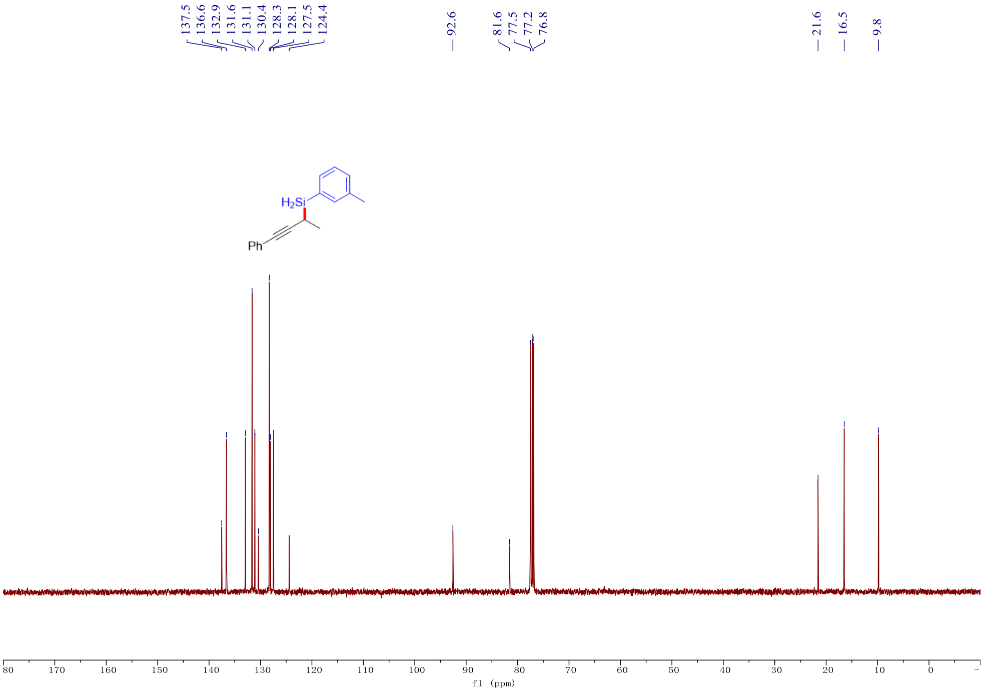

(3,5-Dimethylphenyl)(4-phenylbut-3-yn-2-yl)silane (5ad)

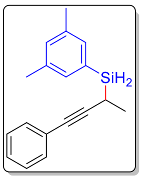

Prepared according to procedure 5 from **1a** (0.2 mmol, 25.6 mg) and **2d** (0.3 mmol, 40.9 mg). The product was isolated in 89% yield (47.3 mg) as colorless oil.

R$_f$: 0.50 (petroleum ether).

HRMS (ESI) (m/z): Calcd for $C_{18}H_{21}Si$ $[M+H]^+$: 265.1413, found: 265.1420.

^{1}H NMR (400 MHz, $CDCl_3$) δ 7.36 (dd, J = 7.4, 2.3 Hz, 2H), 7.31 (s, 2H), 7.28 – 7.21 (m, 3H), 7.07 (s, 1H), 4.40 (d, J = 2.8 Hz, 2H), 2.31 (s, 7H), 1.37 (d, J = 7.2 Hz, 3H).

^{13}C NMR (101 MHz, $CDCl_3$) δ 137.5, 133.6, 132.0, 131.6, 130.2, 128.3, 127.4, 124.4, 92.7, 81.5, 21.4, 16.5, 9.8.

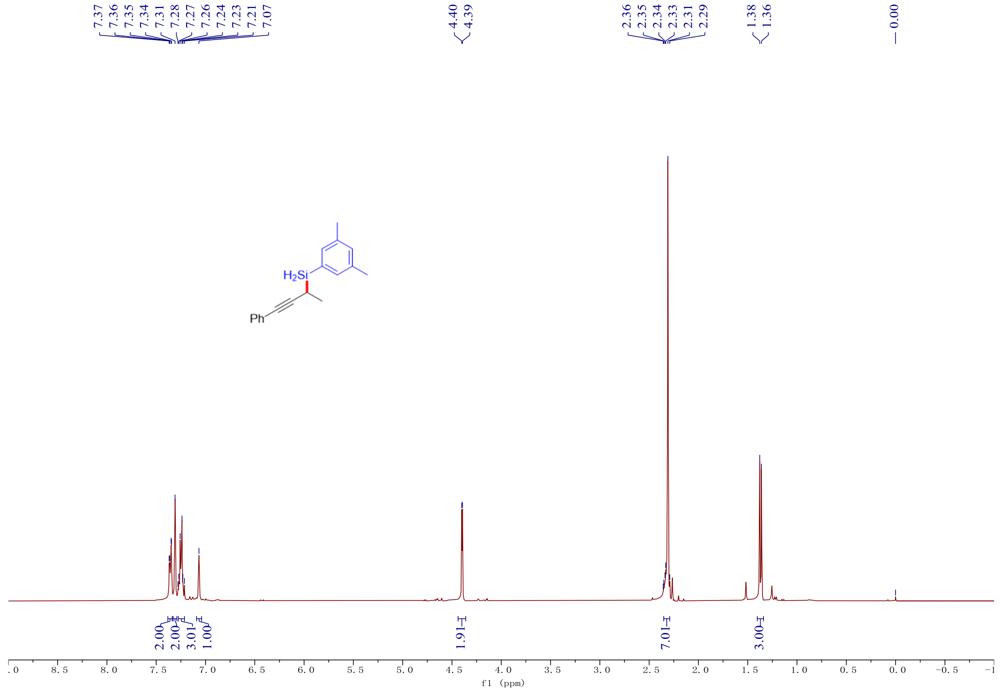

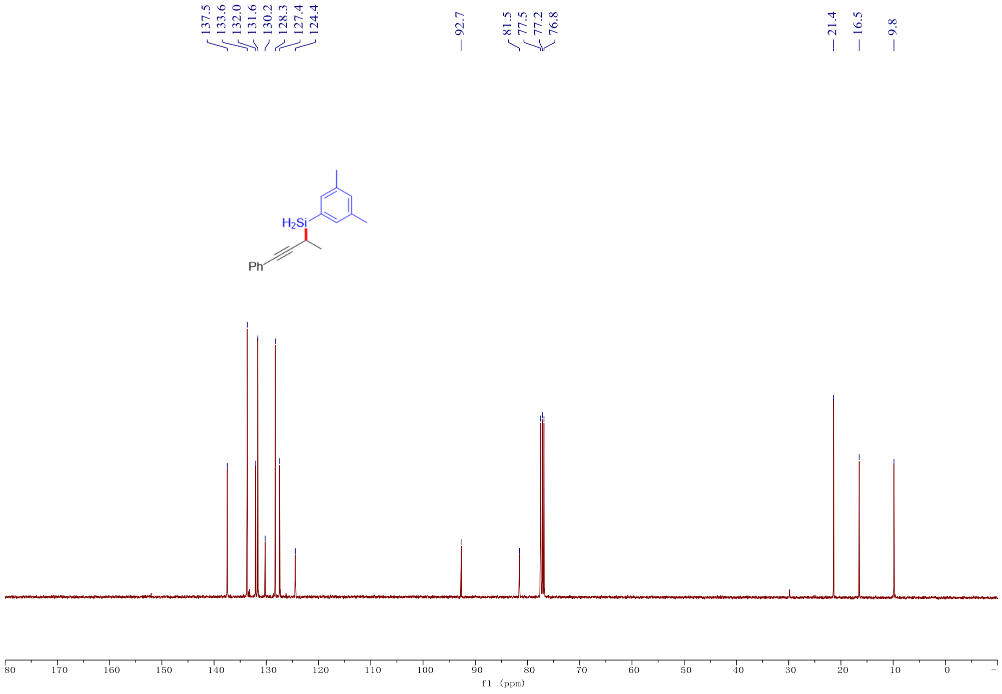

[1,1'-Biphenyl]-4-yl(4-phenylbut-3-yn-2-yl)silane (5ae)

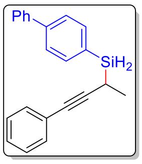

Prepared according to procedure 5 from **1a** (0.2 mmol, 25.6 mg) and **2e** (0.3 mmol, 55.3 mg). The product was isolated in 82% yield (51.5 mg) as colorless oil.

R*f*: 0.30 (petroleum ether).

HRMS (ESI) (m/z): Calcd for $C_{22}H_{21}Si$ $[M+H]^+$: 313.1413, found: 313.1420.

^{1}H NMR (400 MHz, $CDCl_3$) δ 7.79 – 7.75 (m, 2H), 7.64 – 7.58 (m, 4H), 7.44 (t, *J* = 7.6 Hz, 2H), 7.40 – 7.31 (m, 3H), 7.26 (dq, *J* = 8.5, 2.8, 2.2 Hz, 3H), 4.48 (d, *J* = 2.8 Hz, 2H), 2.38 (qt, *J* = 7.2, 2.8 Hz, 1H), 1.40 (d, *J* = 7.2 Hz, 3H).

^{13}C NMR (101 MHz, $CDCl_3$) δ 143.0, 140.9, 136.4, 131.6, 129.3, 129.0, 128.3, 127.8, 127.5, 127.3, 126.9, 124.3, 92.4, 81.6, 16.5, 9.8.

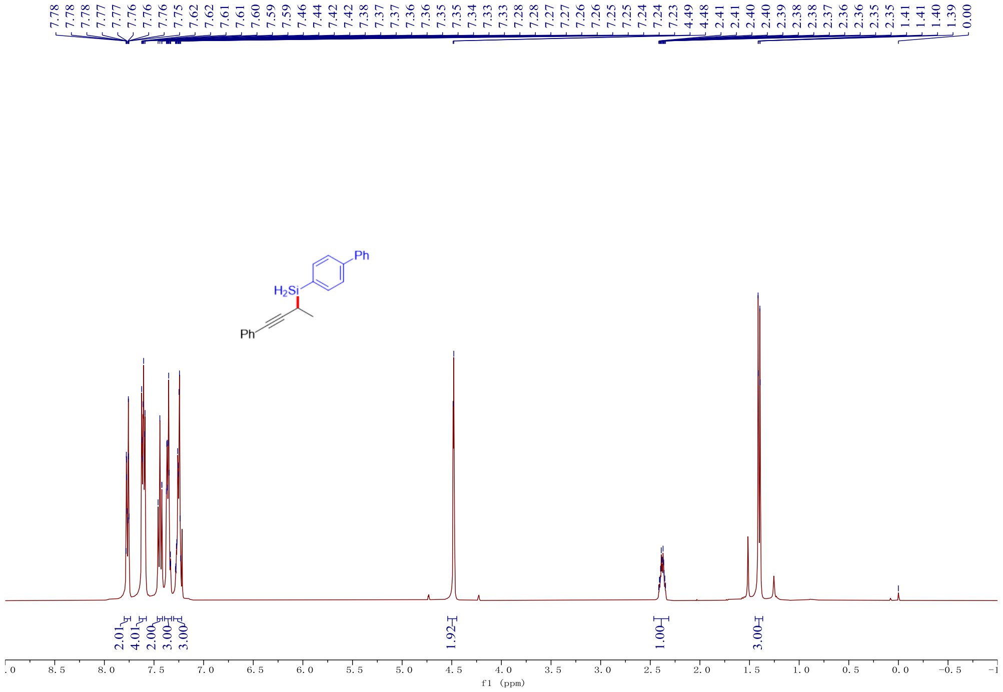

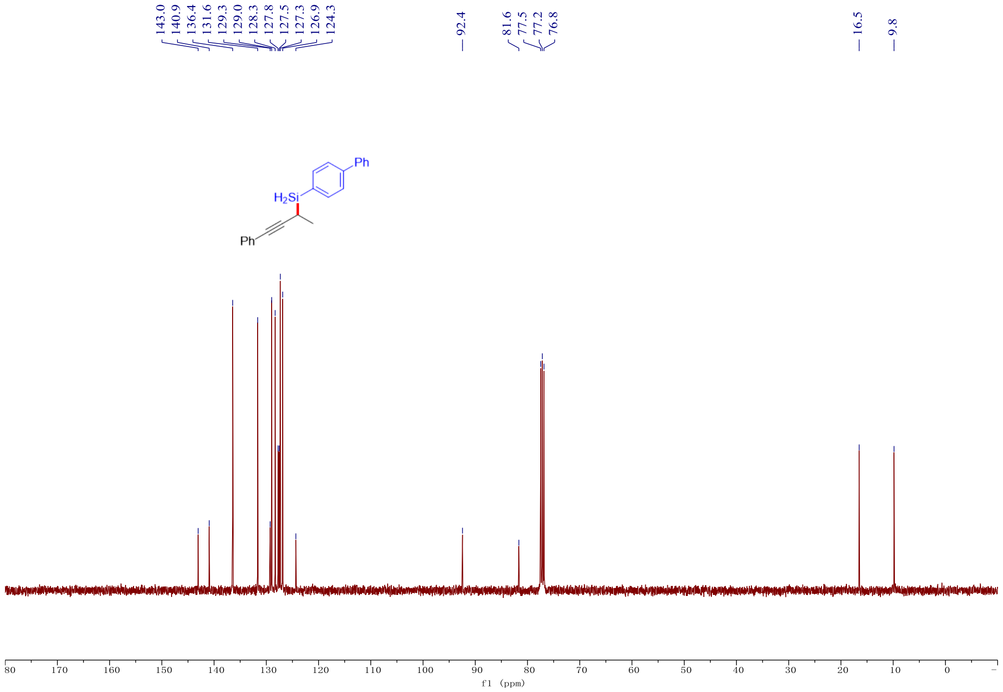

(4-Fluorophenyl)(4-phenylbut-3-yn-2-yl)silane (5af)

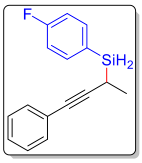

Prepared according to procedure 5 from **1a** (0.2 mmol, 25.6 mg) and **2f** (0.3 mmol, 37.9 mg). The product was isolated in 84% yield (42.7 mg) as colorless oil.

R*f*: 0.70 (petroleum ether).

HRMS (ESI) (m/z): Calcd for $C_{16}H_{16}FSi$ $[M+H]^+$: 255.1005, found: 255.1016.

^{1}H NMR (400 MHz, $CDCl_3$) δ 7.70 – 7.64 (m, 2H), 7.37 – 7.32 (m, 2H), 7.29 – 7.22 (m, 3H), 7.08 (t, *J* = 8.6 Hz, 2H), 4.47 – 4.40 (m, 2H), 2.39 – 2.27 (m, 1H), 1.36 (d, *J* = 7.2 Hz, 3H).

^{13}C NMR (101 MHz, $CDCl_3$) δ 164.6 (d, *J* = 249.8 Hz), 138.0 (d, *J* = 7.7 Hz), 131.6, 128.3, 127.6, 126.0 (d, *J* = 3.8 Hz), 124.2, 115.5 (d, *J* = 20.0 Hz), 92.2, 81.7, 16.4, 9.8.

^{19}F NMR (376 MHz, $CDCl_3$) δ -109.82.

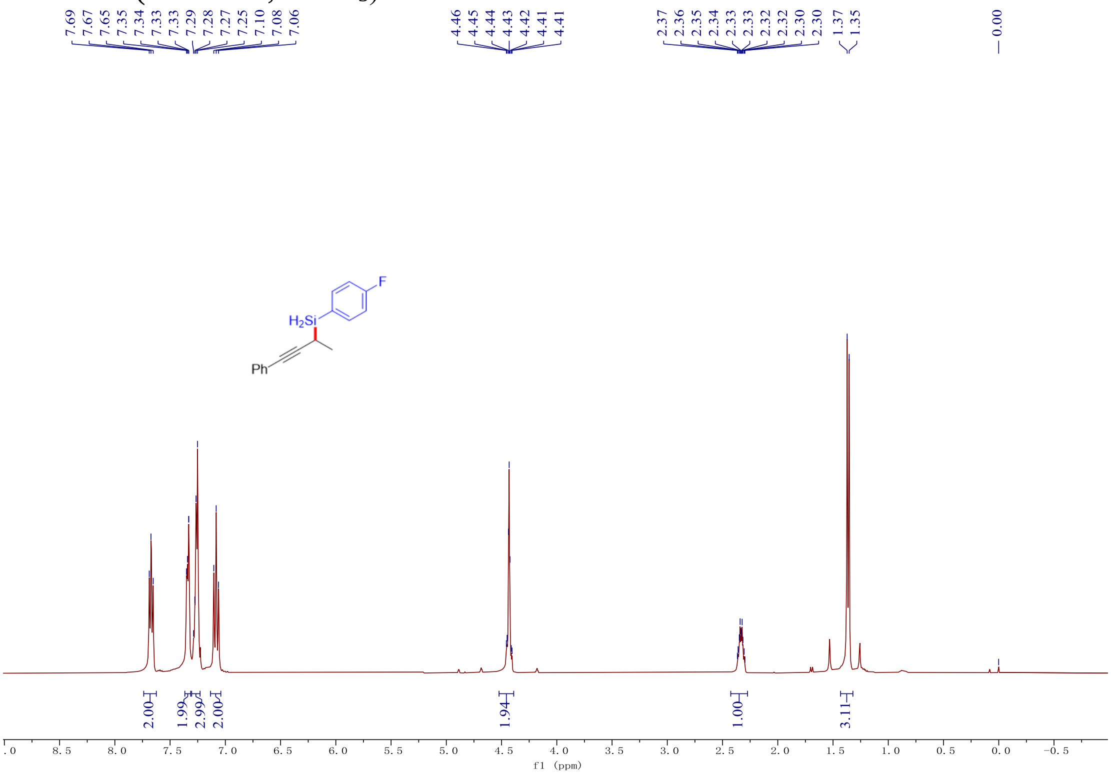

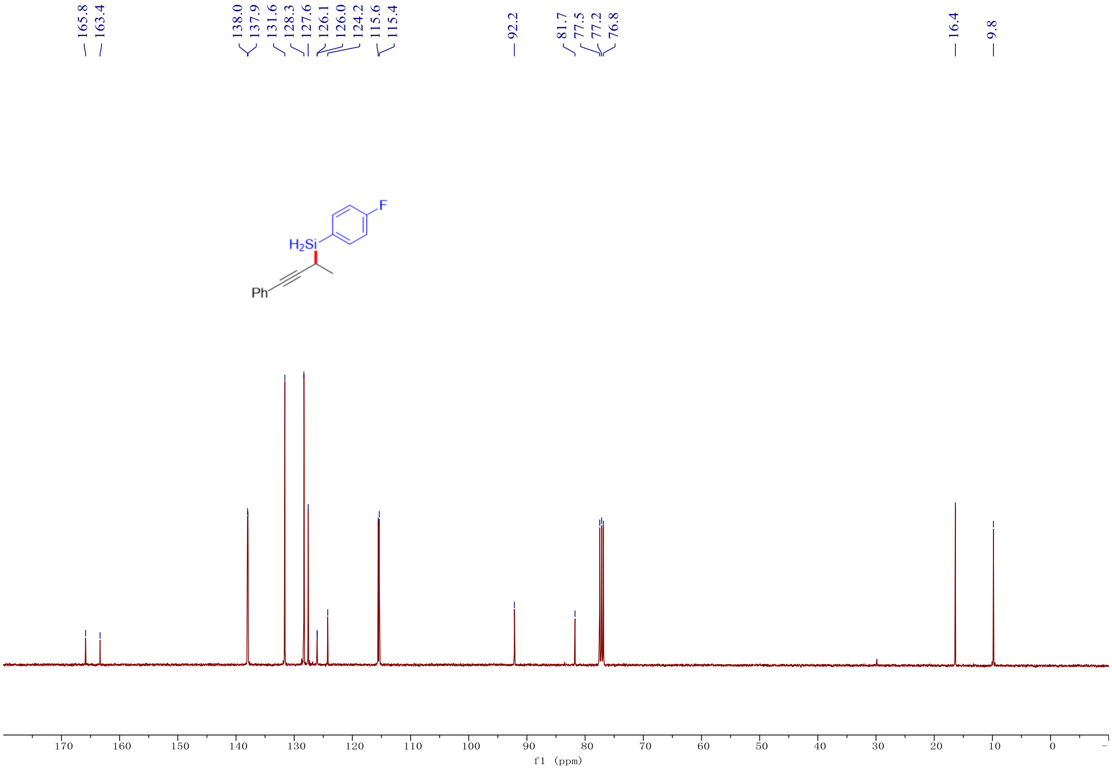
165.8
163.4
138.0
137.9
131.6
128.3
127.6
126.1
126.0
124.2
115.6
115.4
92.2
81.7
77.5
77.2
76.8
16.4
9.8
H2Si
F
Ph
f1 (ppm)

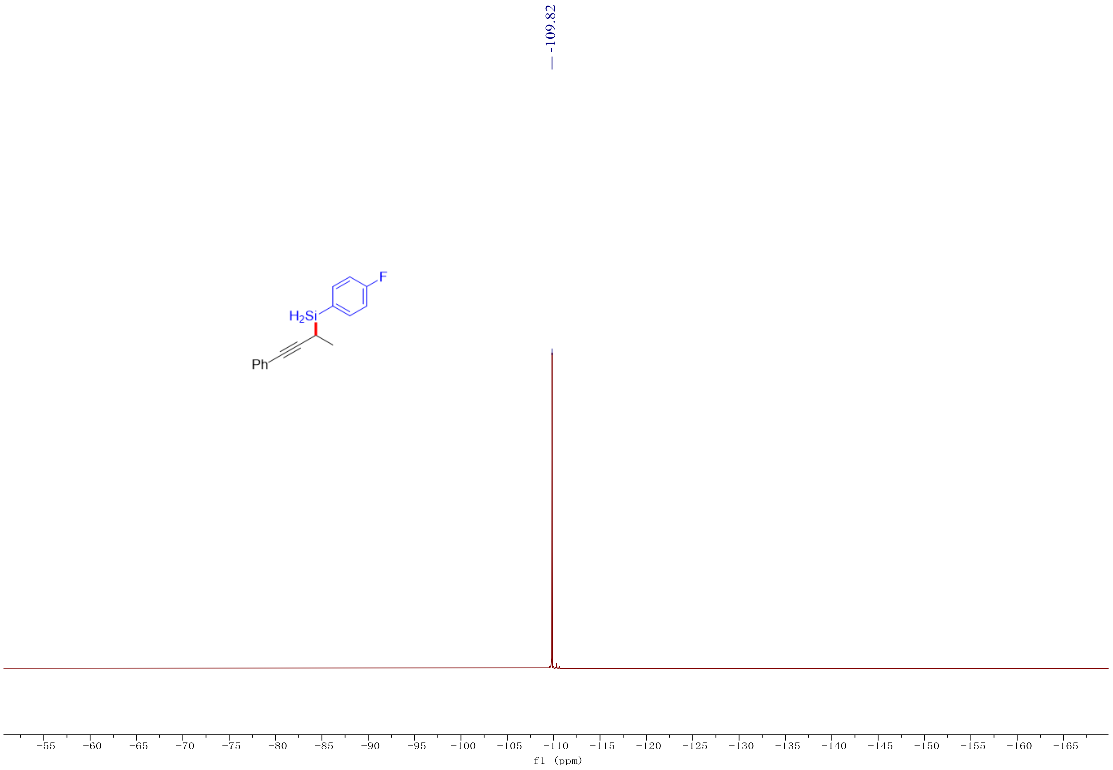
-109.82
H2Si
F
Ph
f1 (ppm)

(4-Chlorophenyl)(4-phenylbut-3-yn-2-yl)silane (5ag)

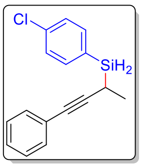

Prepared according to procedure 5 from **1a** (0.2 mmol, 25.6 mg) and **2g** (0.3 mmol, 42.8 mg). The product was isolated in 83% yield (45.1 mg) as colorless oil.

R*f*: 0.65 (petroleum ether).

HRMS (ESI) (m/z): Calcd for $C_{16}H_{15}ClNaSi$ $[M+Na]^+$: 293.0529, found: 293.0490.

^{1}H NMR (400 MHz, $CDCl_3$) δ 7.62 (td, *J* = 8.3, 1.8 Hz, 2H), 7.38 (t, *J* = 1.9 Hz, 1H), 7.37 – 7.32 (m, 3H), 7.30 – 7.24 (m, 3H), 4.47 – 4.37 (m, 2H), 2.34 (qt, *J* = 7.2, 2.8 Hz, 1H), 1.37 (d, *J* = 7.2 Hz, 3H).

^{13}C NMR (101 MHz, $CDCl_3$) δ 137.3, 136.9, 131.6, 128.9, 128.4, 128.3, 127.6, 124.2, 92.0, 81.8, 16.4, 9.7.

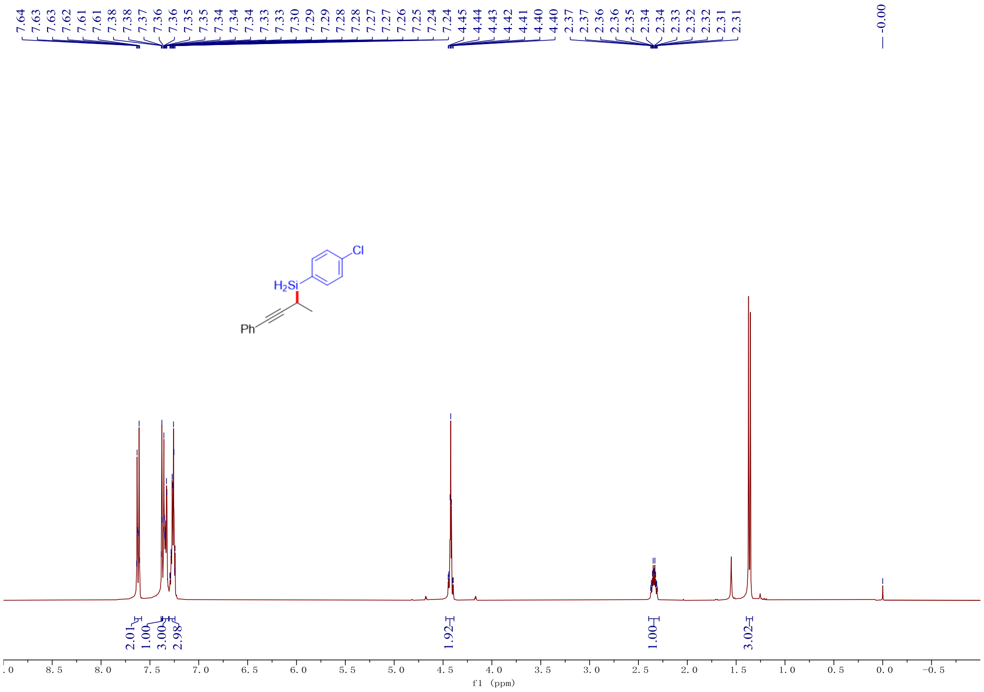

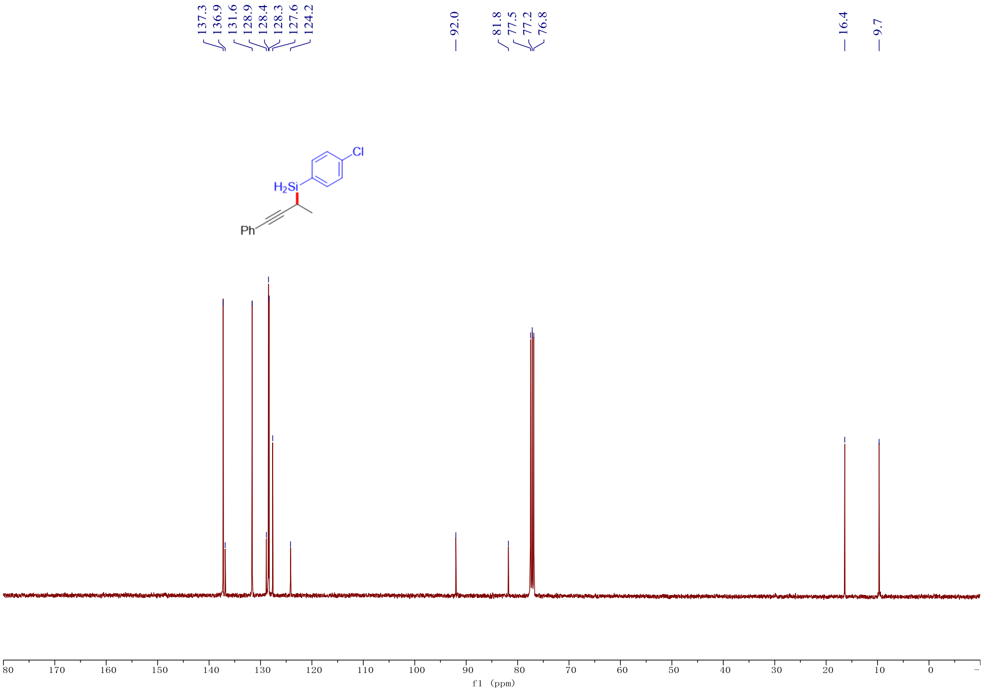

(4-Methoxyphenyl)(4-phenylbut-3-yn-2-yl)silane (5ah)

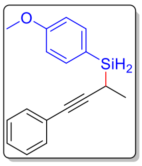

Prepared according to procedure 5 from **1a** (0.2 mmol, 25.6 mg) and **2h** (0.3 mmol, 41.5 mg). The product was isolated in 86% yield (45.6 mg) as colorless oil.

R*f*: 0.50 (ethyl acetate : petroleum ether = 1:50).

HRMS (ESI) (m/z): Calcd for $C_{17}H_{19}OSi$ $[M+H]^+$: 267.1205, found: 267.1202.

^{1}H NMR (400 MHz, $CDCl_3$) δ 7.64 – 7.60 (m, 2H), 7.37 – 7.33 (m, 2H), 7.29 – 7.23 (m, 3H), 6.93 (d, *J* = 8.7 Hz, 2H), 4.42 (d, *J* = 2.8 Hz, 2H), 3.81 (s, 3H), 2.36 – 2.25 (m, 1H), 1.36 (d, *J* = 7.2 Hz, 3H).

^{13}C NMR (101 MHz, $CDCl_3$) δ 161.5, 137.5, 131.6, 128.7, 127.5, 124.4, 121.2, 114.0, 92.7, 81.5, 55.2, 16.4, 10.0.

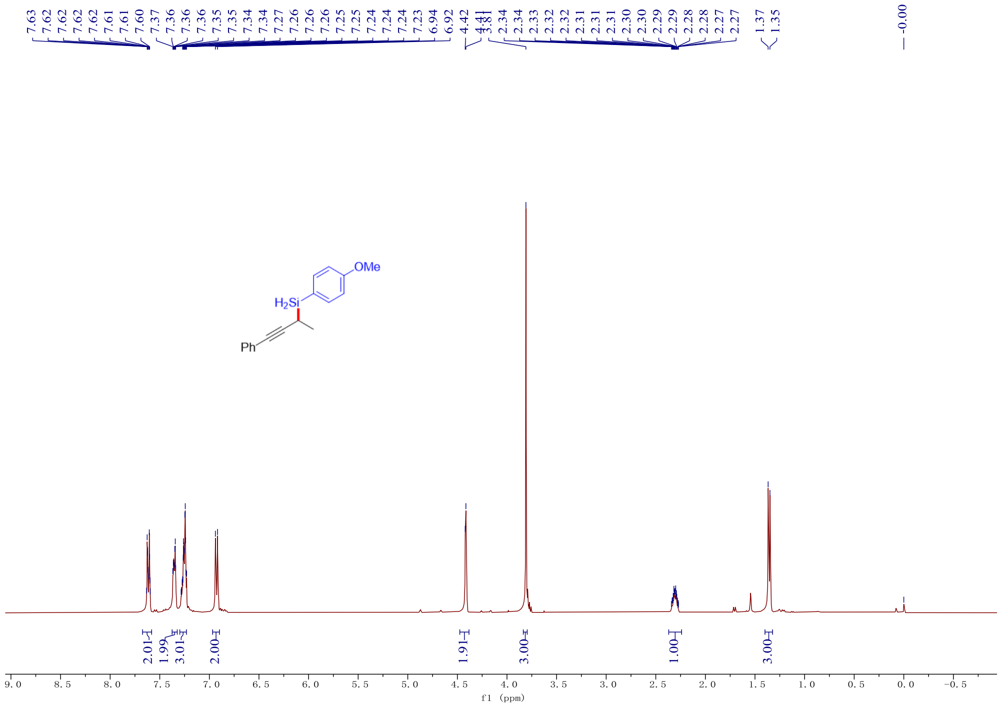

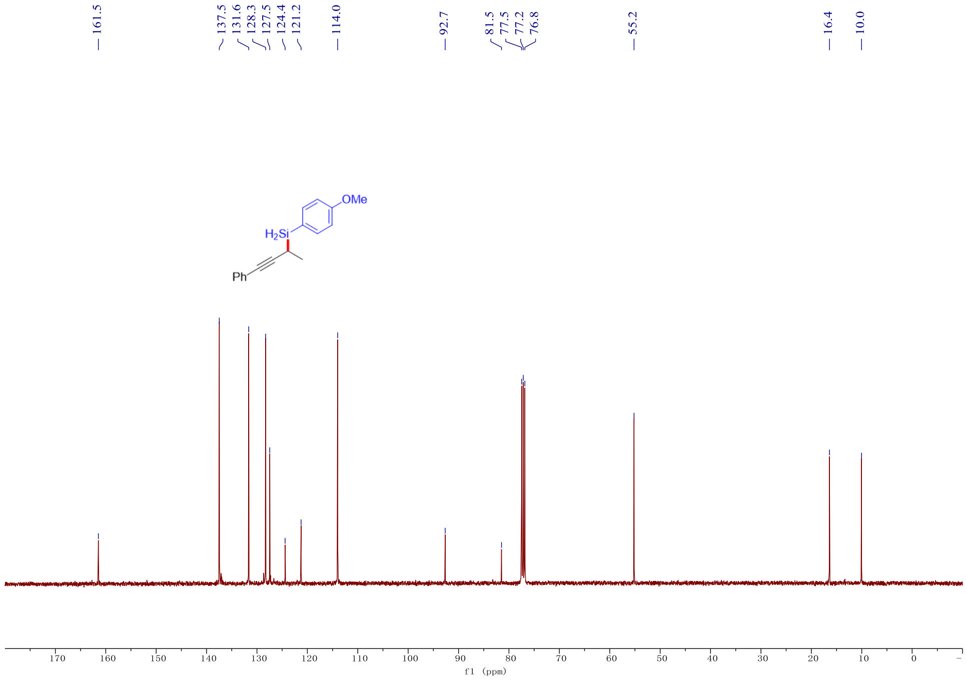

(4-Methoxyphenyl)(4-phenylbut-3-yn-2-yl-1-*d*)silane-*d*$_2$ (*d*-5ah)

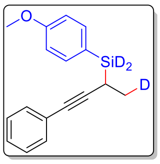

Prepared according to procedure 5 from **1a** (0.2 mmol, 25.6 mg) and ***d*-2h** (0.3 mmol, 41.5 mg). The product was isolated in 87% yield (47.0 mg) as colorless oil.

R*f*: 0.48 (ethyl acetate : petroleum ether = 1:50).

HRMS (ESI) (m/z): Calcd for $C_{17}H_{16}D_3OSi$ $[M+H]^+$: 270.1393, found: 270.1394.

^{1}H NMR (400 MHz, $CDCl_3$) δ 7.64 – 7.60 (m, 2H), 7.38 – 7.34 (m, 2H), 7.29 – 7.24 (m, 3H), 6.95 – 6.91 (m, 2H), 3.81 (s, 3H), 2.30 (t, *J* = 7.2 Hz, 1H), 1.34 (d, *J* = 7.2 Hz, 2H).

^{13}C NMR (101 MHz, $CDCl_3$) δ 161.4, 137.5, 131.6, 128.3, 127.4, 124.4, 121.2, 114.0, 92.7, 81.4, 55.2, 16.1 (d, *J* = 19.9 Hz), 9.8.

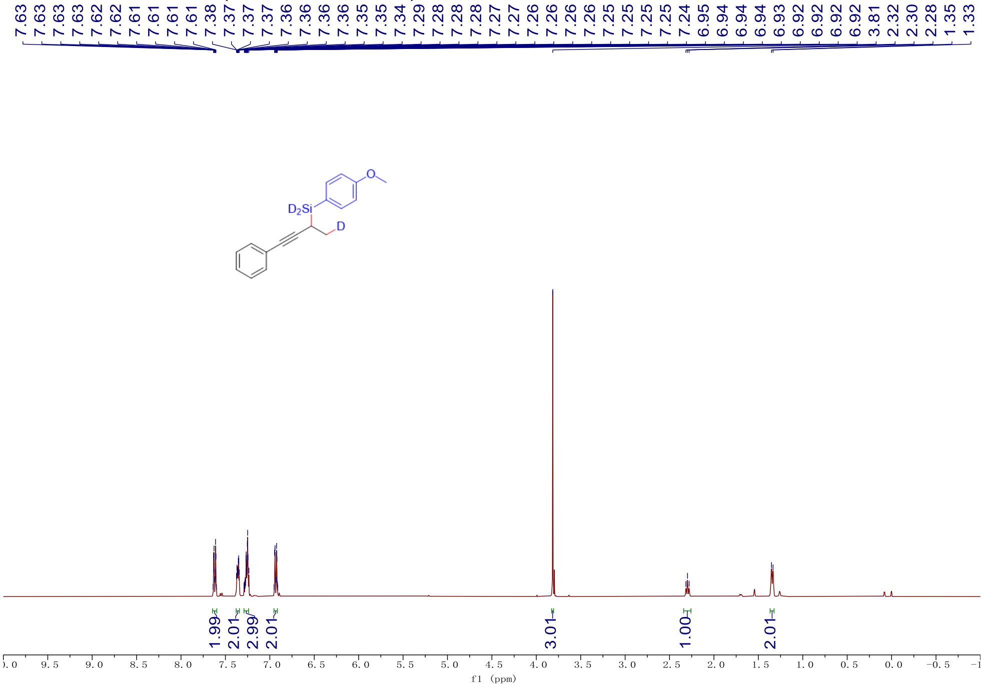

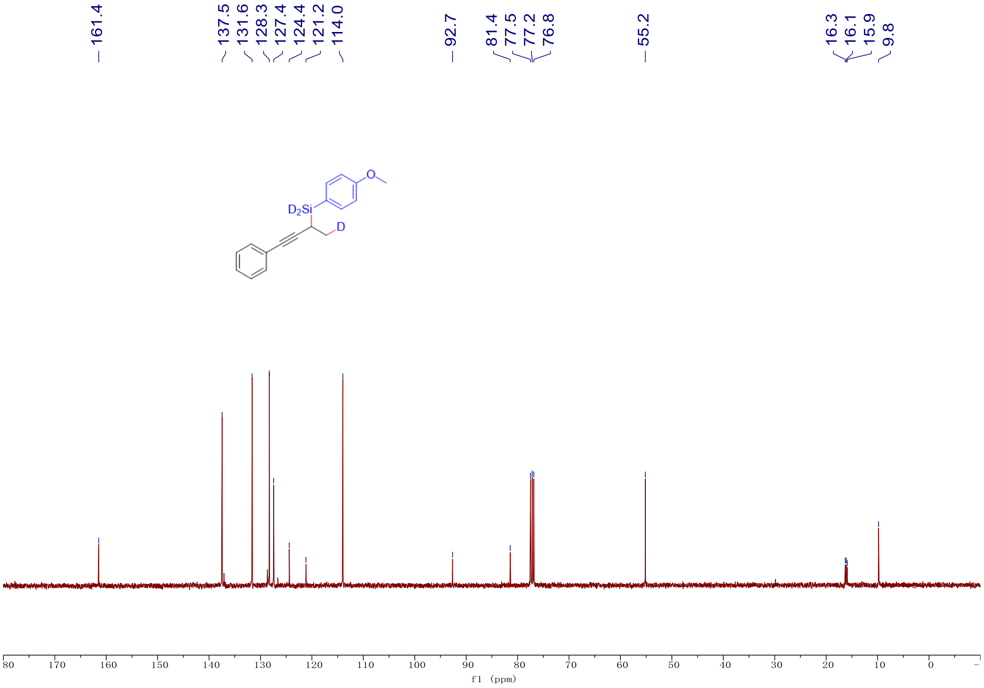

N,*N*-Dimethyl-4-((4-phenylbut-3-yn-2-yl)silyl)aniline (5ai)

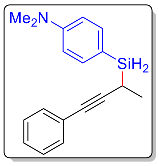

Prepared according to procedure 5 from **1a** (0.2 mmol, 25.6 mg) and **2i** (0.3 mmol, 45.4 mg). The product was isolated in 58% yield (32.2 mg) as colorless oil.

R*f*: 0.47 (ethyl acetate : petroleum ether = 1:50).

HRMS (ESI) (m/z): Calcd for $C_{18}H_{22}NSi$ $[M+H]^+$: 280.1522, found: 280.1527.

^{1}H NMR (400 MHz, $CDCl_3$) δ 7.56 – 7.52 (m, 2H), 7.39 – 7.36 (m, 2H), 7.29 – 7.23 (m, 3H), 6.72 (d, *J* = 8.7 Hz, 2H), 4.43 – 4.37 (m, 2H), 2.97 (s, 6H), 2.34 – 2.22 (m, 1H), 1.36 (d, *J* = 7.2 Hz, 3H).

^{13}C NMR (101 MHz, $CDCl_3$) δ 151.8, 137.1, 131.7, 128.2, 127.3, 124.6, 115.0, 112.0, 93.2, 81.2, 40.2, 16.4, 10.3.

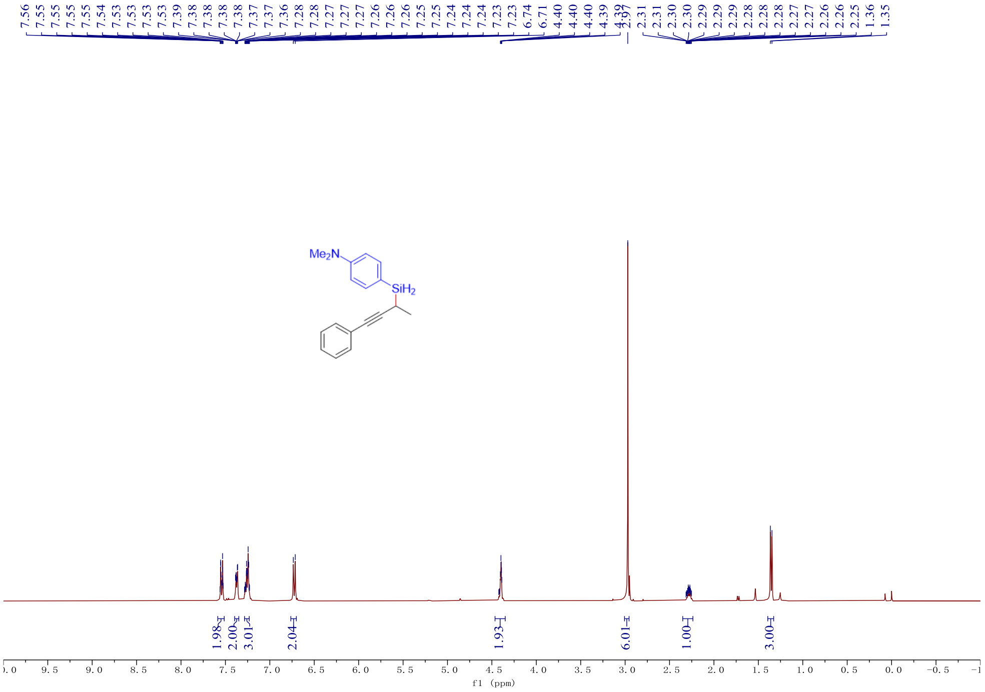

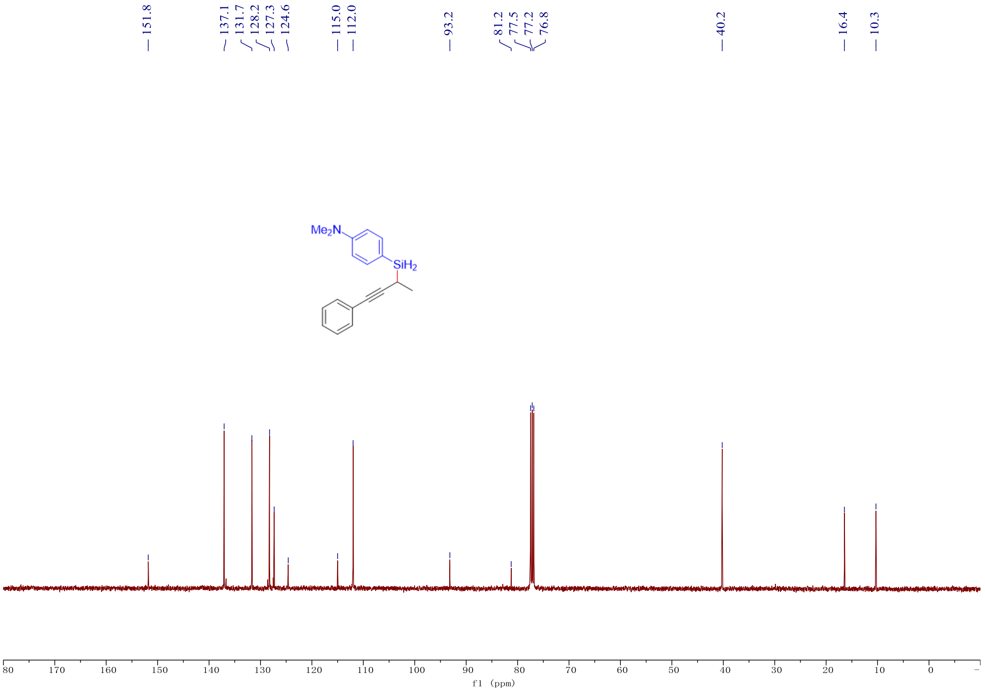

(4-Phenylbut-3-yn-2-yl)(4-(trifluoromethyl)phenyl)silane (5aj)

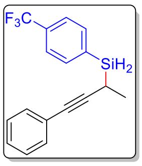

Prepared according to procedure 5 from **1a** (0.2 mmol, 25.6 mg) and **2j** (0.3 mmol, 52.9 mg). The product was isolated in 55% yield (33.5 mg) as colorless oil.

R*f*: 0.42 (petroleum ether).

HRMS (ESI) (m/z): Calcd for $C_{17}H_{16}F_3Si$ $[M+H]^+$: 305.0973, found: 305.0975.

^{1}H NMR (400 MHz, $CDCl_3$) δ 7.83 (d, *J* = 7.7 Hz, 2H), 7.63 (d, *J* = 7.7 Hz, 2H), 7.34 – 7.30 (m, 2H), 7.29 – 7.25 (m, 3H), 4.52 – 4.42 (m, 2H), 2.45 – 2.33 (m, 1H), 1.39 (d, *J* = 7.3 Hz, 3H).

^{13}C NMR (101 MHz, $CDCl_3$) δ 136.3, 135.6, 132.2 (q, *J* = 32.4 Hz), 131.6, 128.4, 127.7, 124.7 (q, *J* = 3.8 Hz), 124.2 (q, *J* = 272.2 Hz), 124.1, 91.7, 82.0, 16.4, 9.5.

^{19}F NMR (376 MHz, $CDCl_3$) δ -63.1.

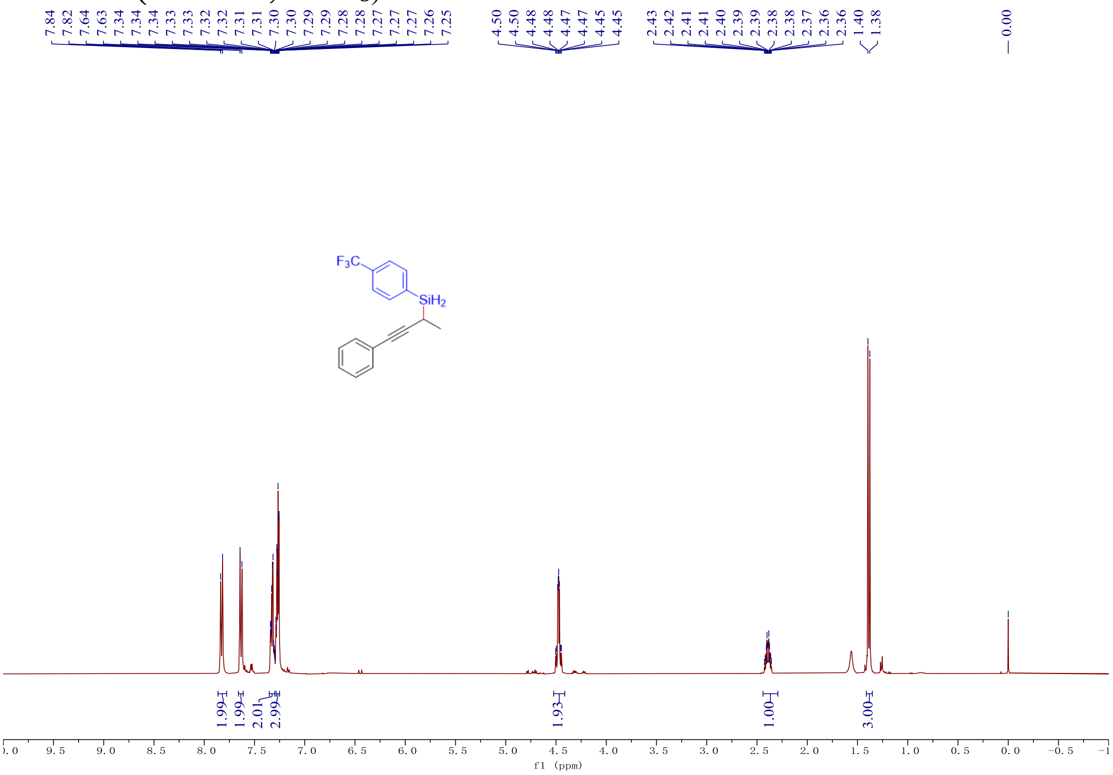

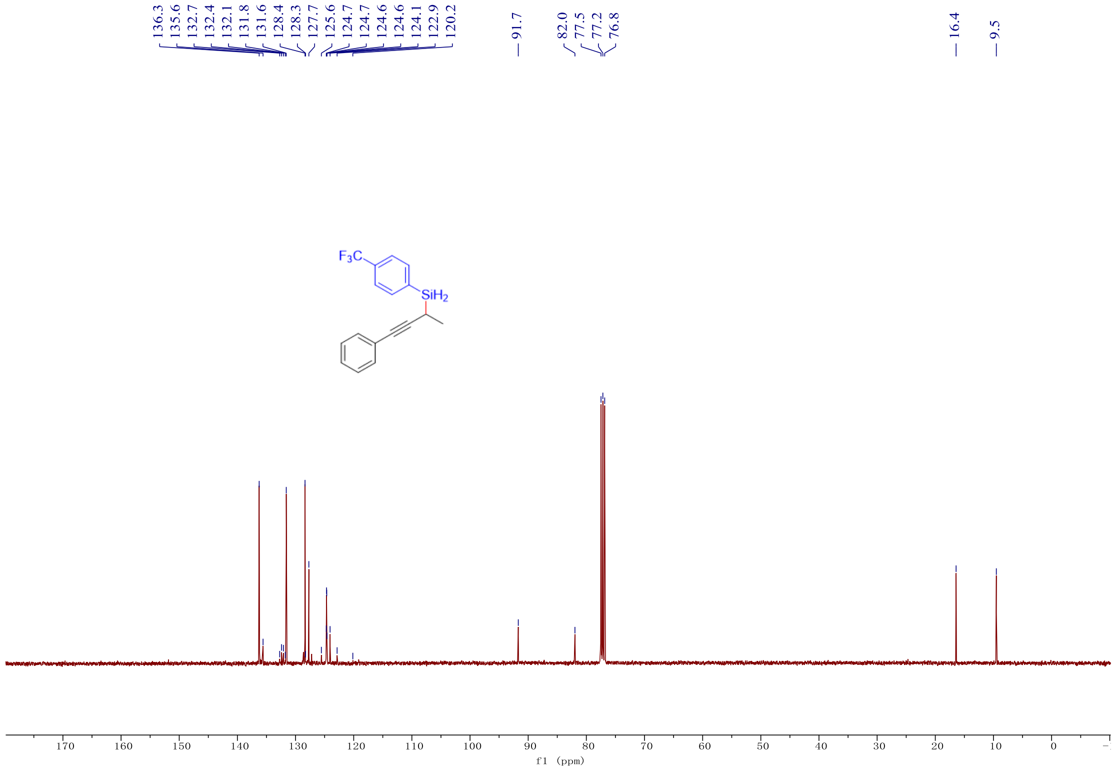
136.3
135.6
132.7
132.4
132.1
131.8
131.6
128.4
128.3
127.7
125.6
124.7
124.7
124.6
124.6
124.1
122.9
120.2
91.7
82.0
77.5
77.2
76.8
16.4
9.5
F3C
SiH2
f1 (ppm)

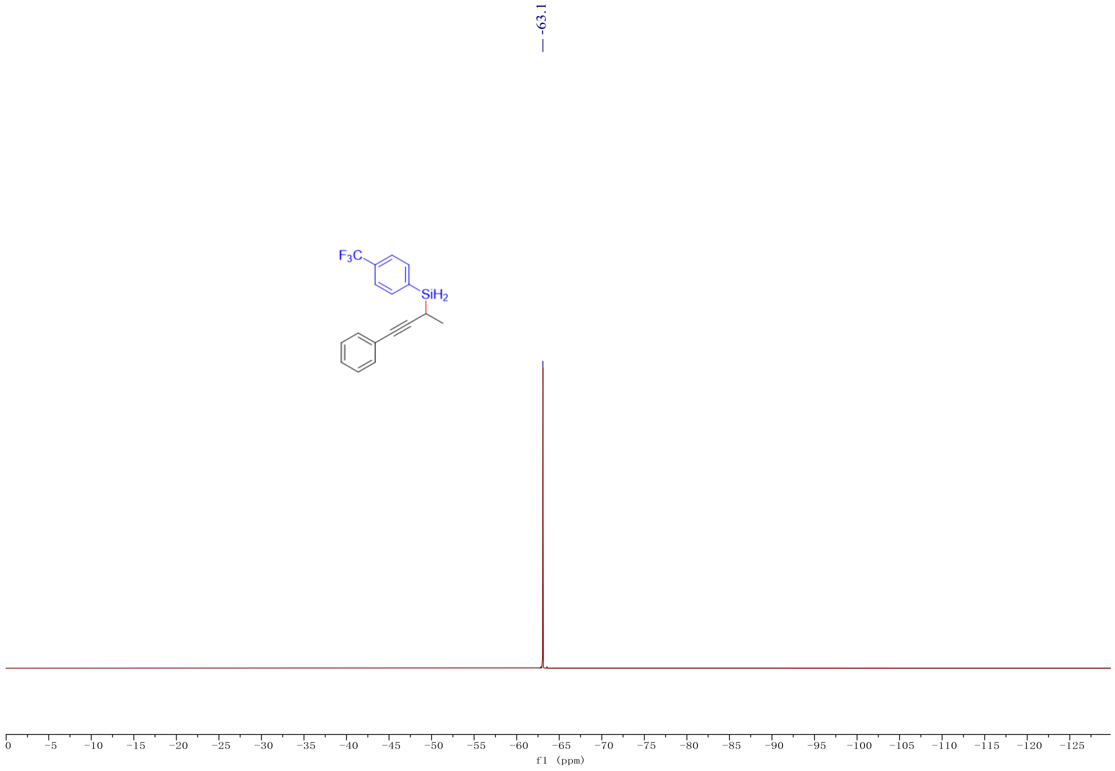
-63.1
F3C
SiH2
f1 (ppm)

(2-(Cyclopentyloxy)phenyl)(4-phenylbut-3-yn-2-yl)silane (5ak)

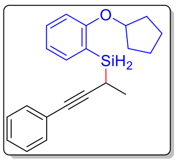

Prepared according to procedure 5 from **1a** (0.2 mmol, 25.6 mg) and **2k** (0.3 mmol, 57.7 mg). The product was isolated in 78% yield (50.1 mg) as colorless oil.

R*f*: 0.30 (ethyl acetate : petroleum ether = 1:100).

HRMS (ESI) (m/z): Calcd for $C_{21}H_{25}OSi$ $[M+H]^+$: 321.1675, found: 321.1667.

^{1}H NMR (400 MHz, $CDCl_3$) δ 7.56 (d, *J* = 7.1 Hz, 1H), 7.38 (t, *J* = 7.8 Hz, 1H), 7.33 – 7.29 (m, 2H), 7.23 (dt, *J* = 7.6, 2.3 Hz, 3H), 6.93 (t, *J* = 7.3 Hz, 1H), 6.83 (d, *J* = 8.2 Hz, 1H), 4.81 (p, *J* = 4.1 Hz, 1H), 4.41 (dd, *J* = 7.8, 2.9 Hz, 1H), 4.31 (dd, *J* = 7.8, 2.9 Hz, 1H), 2.46 (qt, *J* = 7.2, 2.9 Hz, 1H), 1.92 – 1.85 (m, 4H), 1.81 (td, *J* = 7.7, 3.5 Hz, 2H), 1.69 – 1.59 (m, 2H), 1.35 (d, *J* = 7.2 Hz, 3H).

^{13}C NMR (101 MHz, $CDCl_3$) δ 162.8, 138.0, 132.1, 131.7, 128.2, 127.3, 124.6, 120.3, 120.1, 111.3, 93.3, 81.0, 79.3, 33.0, 33.0, 24.2, 24.2, 16.8, 9.2.

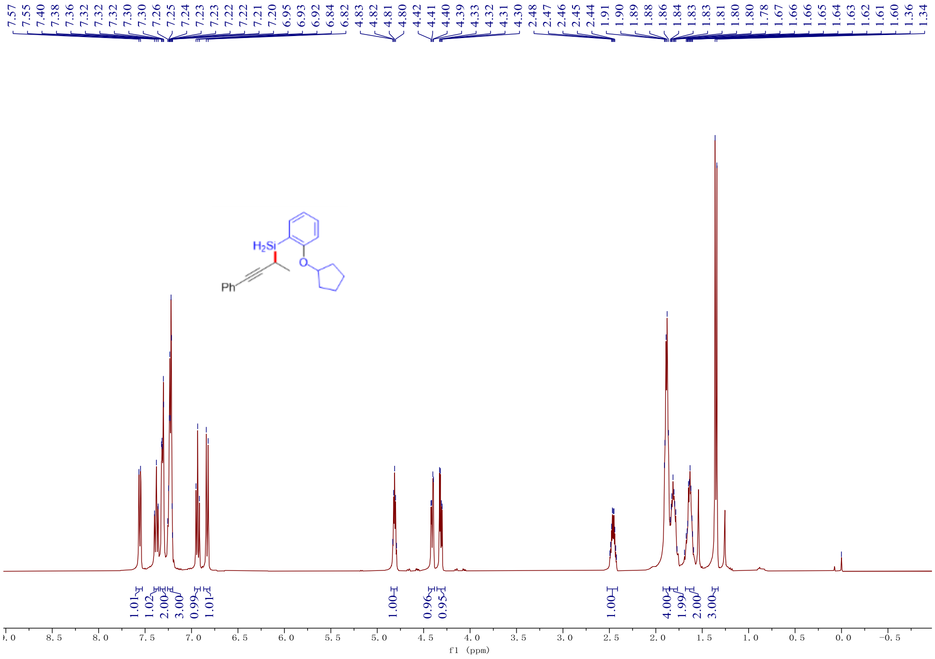

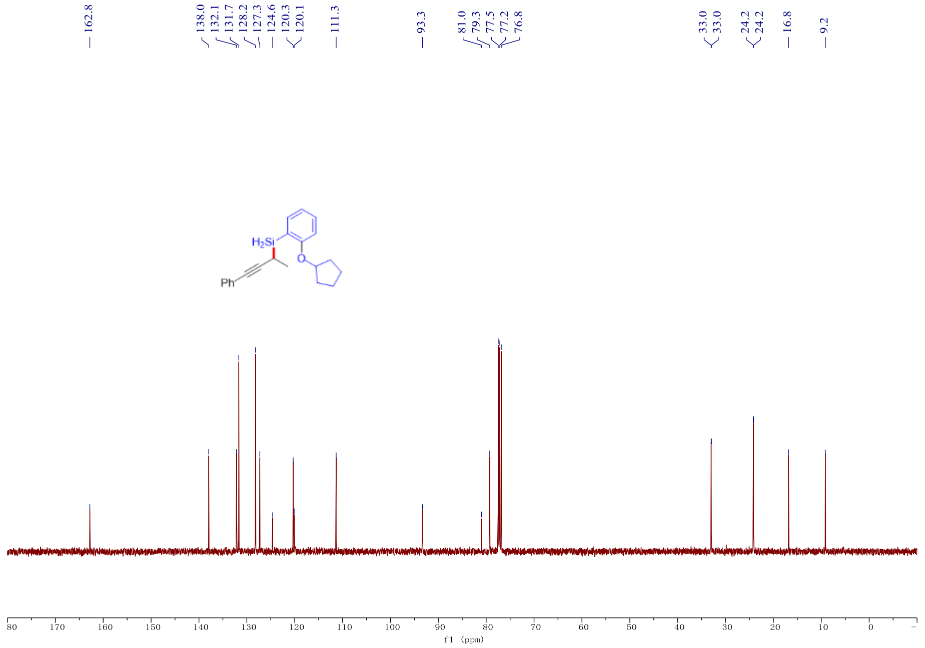

Naphthalen-1-yl(4-phenylbut-3-yn-2-yl)silane (5al)

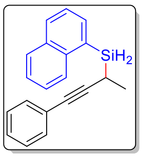

Prepared according to procedure 5 from **1a** (0.2 mmol, 25.6 mg) and **2l** (0.3 mmol, 47.5 mg). The product was isolated in 66% yield (37.9 mg) as colorless oil.

R*f*: 0.30 (petroleum ether).

HRMS (ESI) (m/z): Calcd for $C_{20}H_{19}Si$ $[M+H]^+$: 287.1256, found: 287.1262.

^{1}H NMR (400 MHz, $CDCl_3$) δ 8.18 (dd, *J* = 6.3, 3.3 Hz, 1H), 7.97 – 7.84 (m, 3H), 7.54 – 7.47 (m, 3H), 7.29 – 7.22 (m, 5H), 4.81 (dd, *J* = 7.1, 3.2 Hz, 1H), 4.76 (dd, *J* = 7.1, 3.2 Hz, 1H), 2.51 (tp, *J* = 7.2, 3.2 Hz, 1H), 1.38 (d, *J* = 7.2 Hz, 3H).

^{13}C NMR (101 MHz, $CDCl_3$) δ 137.4, 137.0, 133.3, 131.6, 131.2, 129.5, 129.0, 128.2, 127.5, 126.5, 126.0, 125.3, 124.3, 92.7, 81.6, 16.9, 10.0.

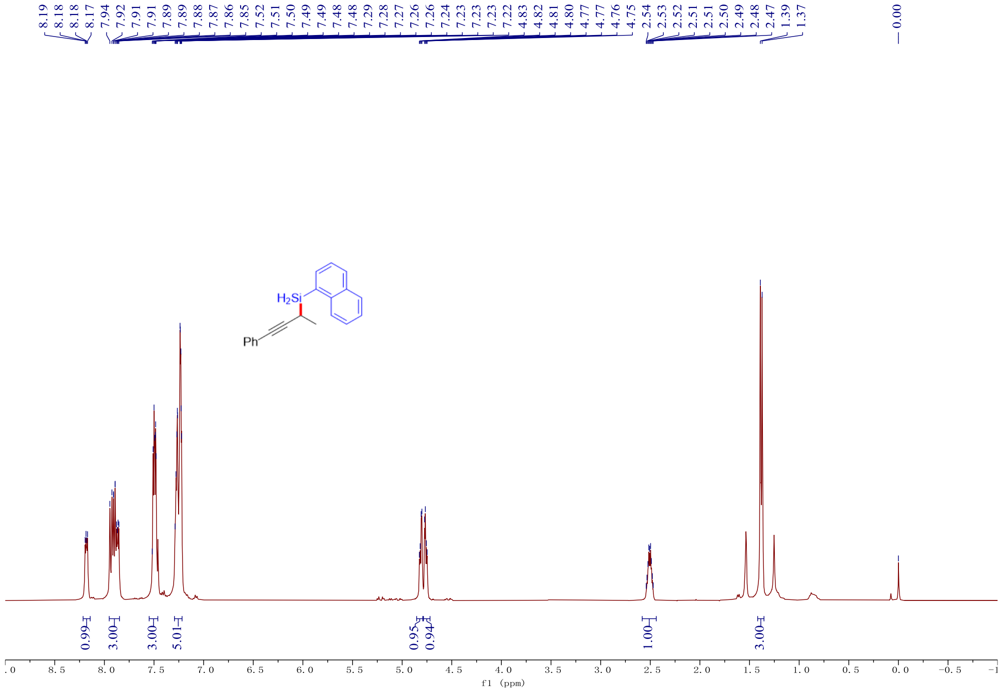

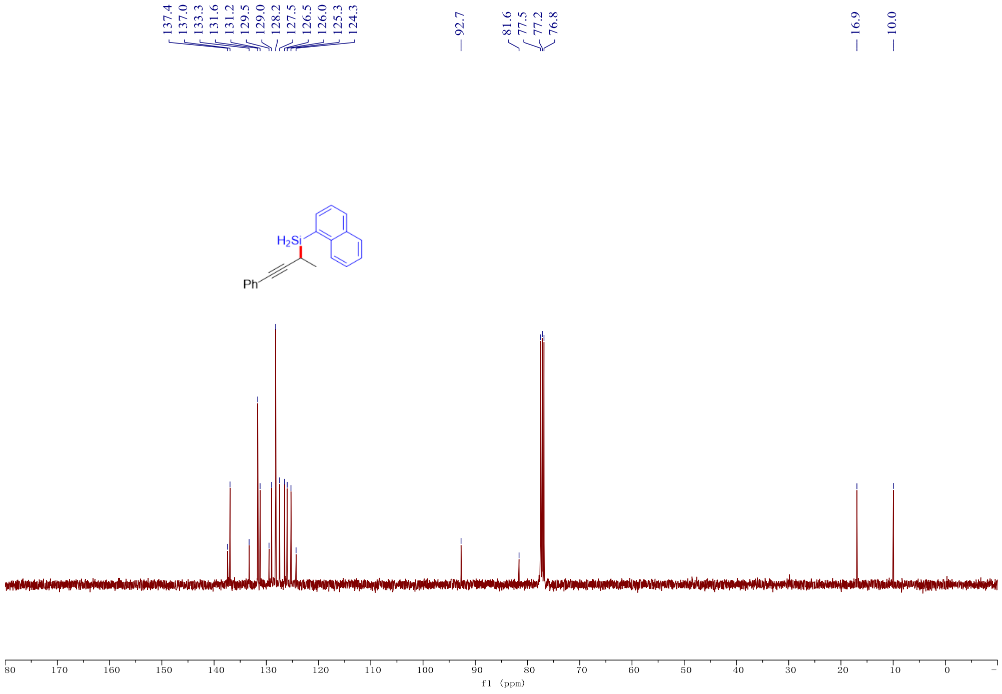

Octyl(4-phenylbut-3-yn-2-yl)silane (5am)

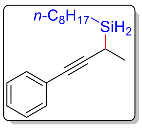

Prepared according to procedure 5 from **1a** (0.2 mmol, 25.6 mg) and **2m** (0.3 mmol, 43.3 mg). The product was isolated in 75% yield (40.6 mg) as colorless oil.

R*f*: 0.71 (petroleum ether).

HRMS (ESI) (m/z): Calcd for $C_{18}H_{29}Si$ $[M+H]^+$: 273.2039, found: 273.2027.

^{1}H NMR (400 MHz, $CDCl_3$) δ 7.37 (dd, *J* = 7.6, 2.1 Hz, 2H), 7.29 – 7.22 (m, 3H), 3.86 – 3.76 (m, 2H), 2.22 – 2.11 (m, 1H), 1.48 (q, *J* = 7.9, 7.5 Hz, 2H), 1.36 (d, *J* = 7.2 Hz, 5H), 1.31 – 1.23 (m, 7H), 0.93 – 0.83 (m, 5H).

^{13}C NMR (101 MHz, $CDCl_3$) δ 131.6, 128.3, 127.4, 124.5, 93.0, 80.8, 33.0, 32.0, 29.4, 29.3, 25.2, 22.8, 16.9, 14.3, 8.6, 8.3.

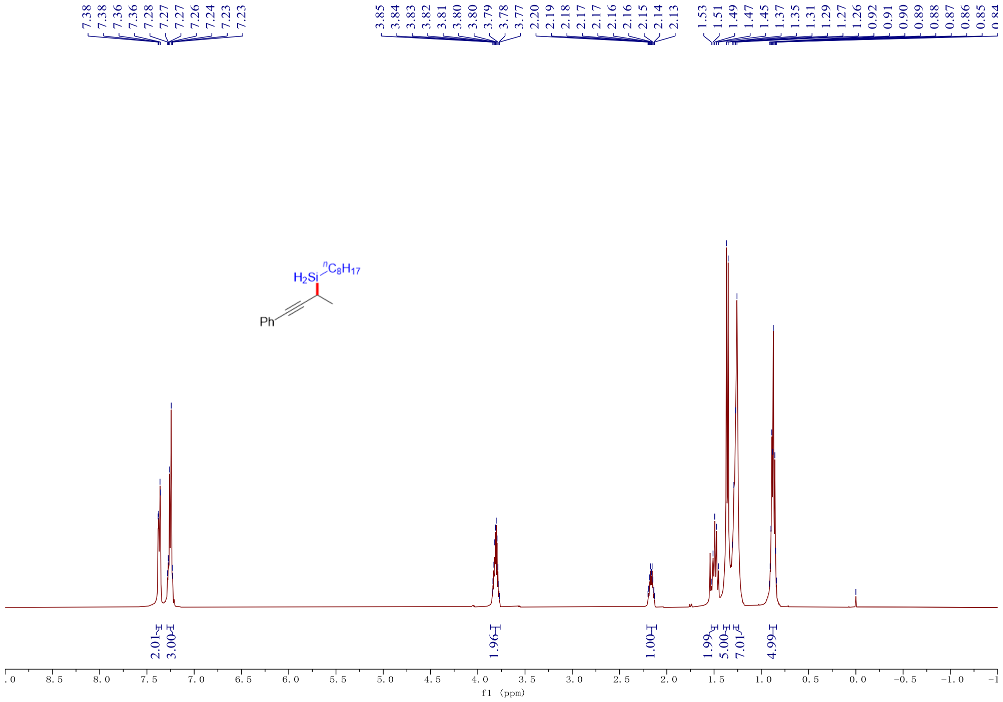

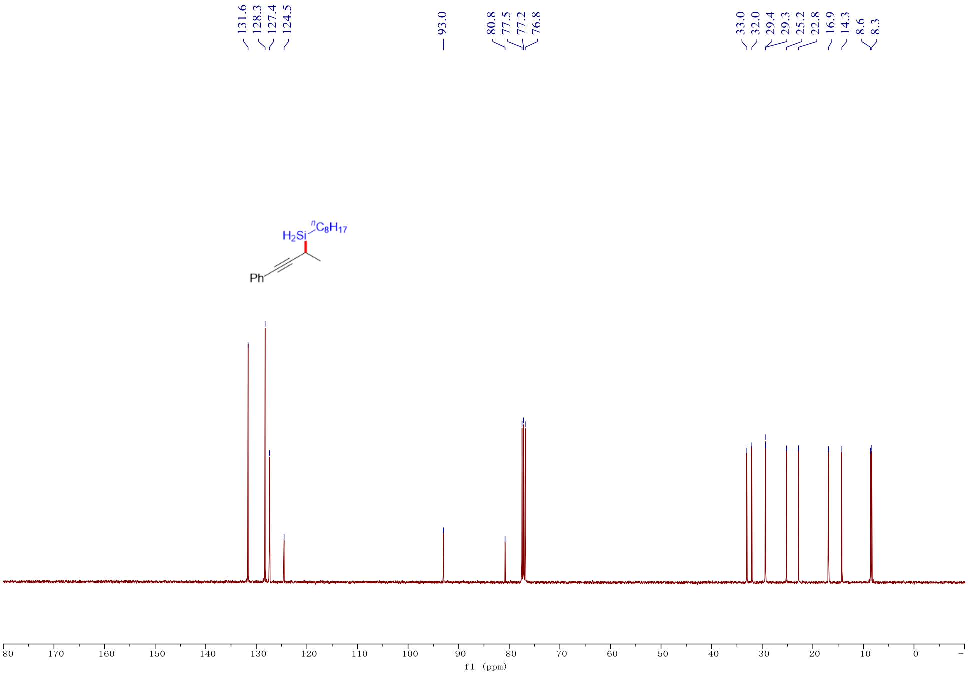

4-(But-1-yn-1-yl-3-*d*)-1,1'-biphenyl (15)

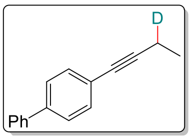

HRMS (ESI) (m/z): Calcd for $C_{16}H_{14}D$ $[M+H]^+$: 208.1237, found: 208.1233.

^{1}H NMR (400 MHz, $CDCl_3$) δ 7.58 (d, *J* = 7.0 Hz, 2H), 7.52 (d, *J* = 8.3 Hz, 2H), 7.49 – 7.40 (m, 4H), 7.34 (t, *J* = 7.4 Hz, 1H), 2.50 – 2.38 (m, 1H), 1.25 (d, *J* = 7.4 Hz, 3H).

^{13}C NMR (101 MHz, $CDCl_3$) δ 140.7, 140.4, 132.1, 128.9, 127.6, 127.1, 127.0, 123.1, 92.5, 79.9, 14.0, 13.1 (t, *J* = 20.2 Hz).

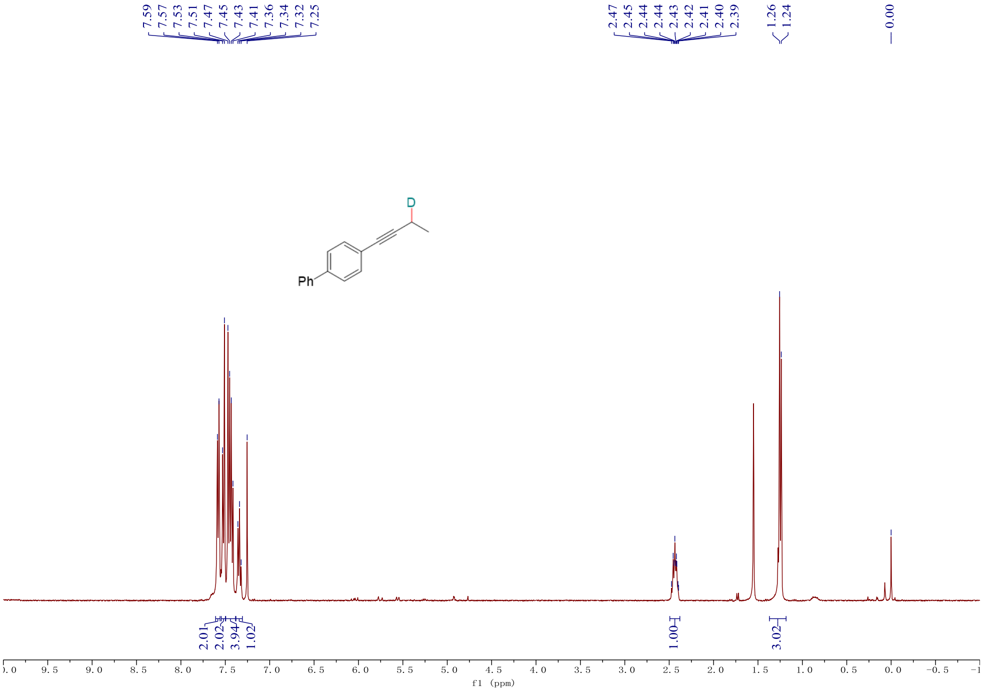

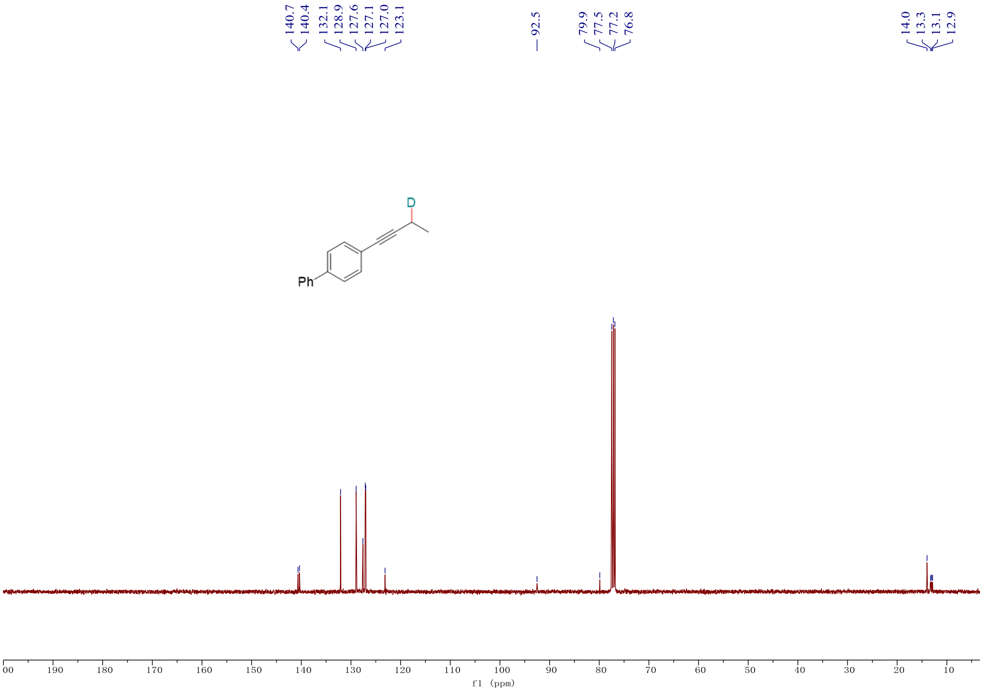

4-(Buta-1,2-dien-1-yl-1-*d*)-1,1'-biphenyl (16)

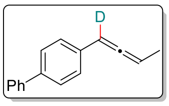

HRMS (ESI) (m/z): Calcd for $C_{16}H_{14}D$ $[M+H]^+$: 208.1237, found: 208.1228.

^{1}H NMR (400 MHz, $CDCl_3$) δ 7.60 – 7.57 (m, 2H), 7.55 – 7.52 (m, 2H), 7.42 (t, *J* = 7.5 Hz, 2H), 7.37 – 7.30 (m, 3H), 5.57 (q, *J* = 7.2 Hz, 1H), 1.80 (d, *J* = 7.1 Hz, 3H).

^{13}C NMR (101 MHz, $CDCl_3$) δ 206.4, 141.0, 139.7, 134.3, 128.9, 127.4, 127.3, 127.2, 127.1, 93.6 (t, *J* = 24.9 Hz), 89.9, 14.3.

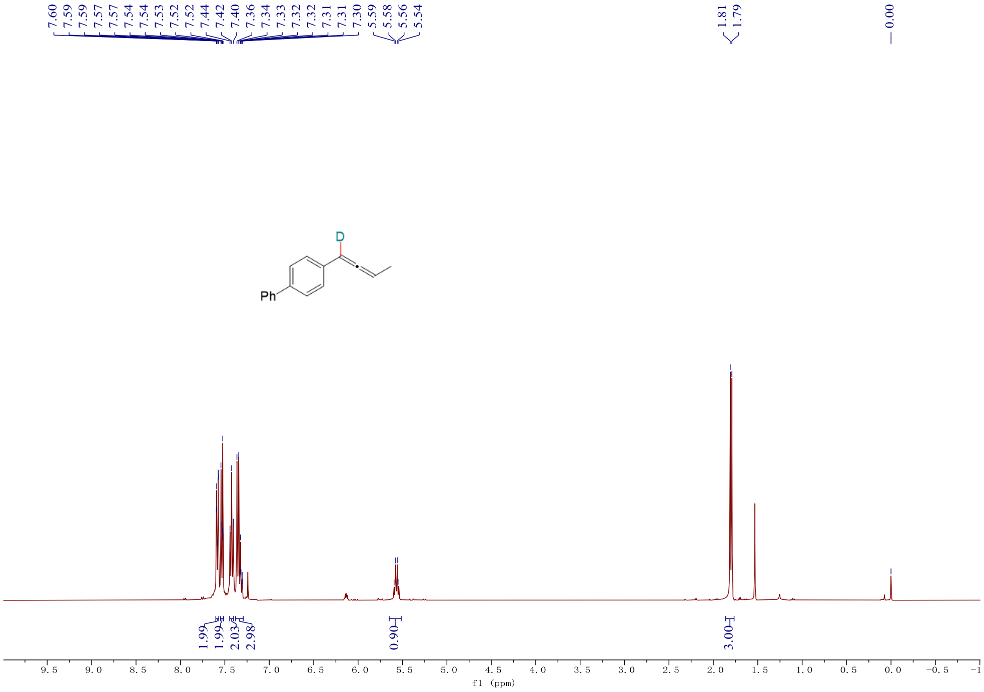

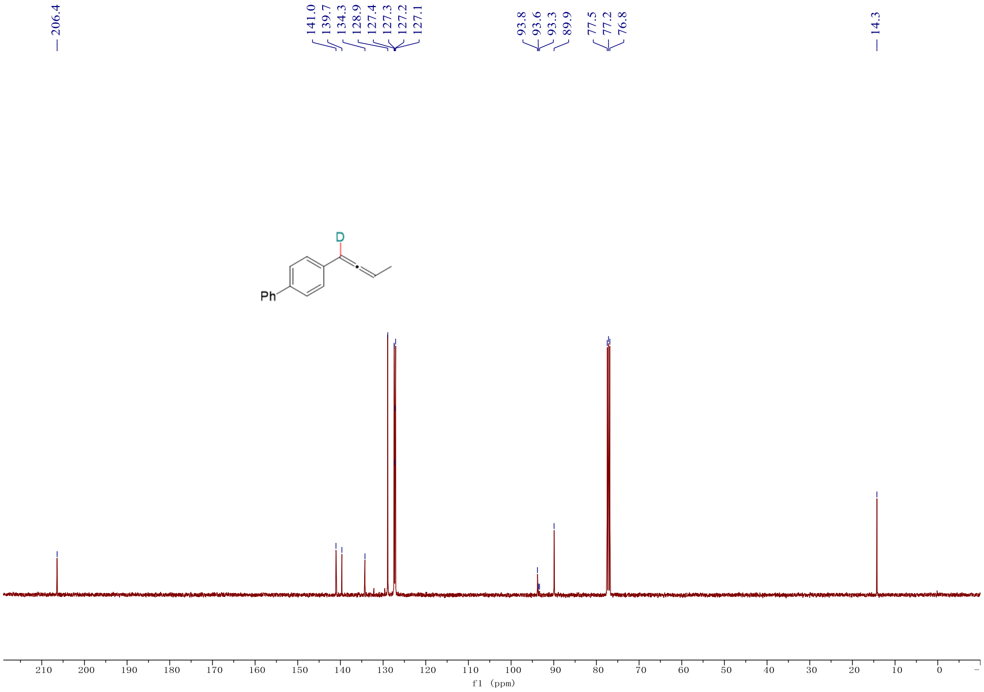

(2-(Cyclopentyloxy)phenyl)(4-phenylbut-3-yn-2-yl)silane (8ak)

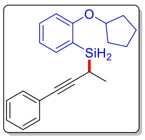

Prepared according to procedure 6 from **1a** (0.2 mmol, 25.6 mg) and **2k** (0.6 mmol, 115.4 mg). The product was isolated in 70% yield with 60% ee value (44.9 mg) as colorless oil.

The characterization data and spectrums of **8ak** are same to **5ak**.

$[\alpha]_D^{20}$ +20.9° (*c* 1.5, ethyl acetate).

The enantiomeric excess of **8ak** was determined by chiral HPLC analysis on Chiralcel OD-3 column.

Conditions: hexane, flow rate = 0.5 mL/min, UV-Vis detection at λ = 254 nm.

t_{R1} = 35.6 min (major), t_{R2} = 44.3 min (minor).

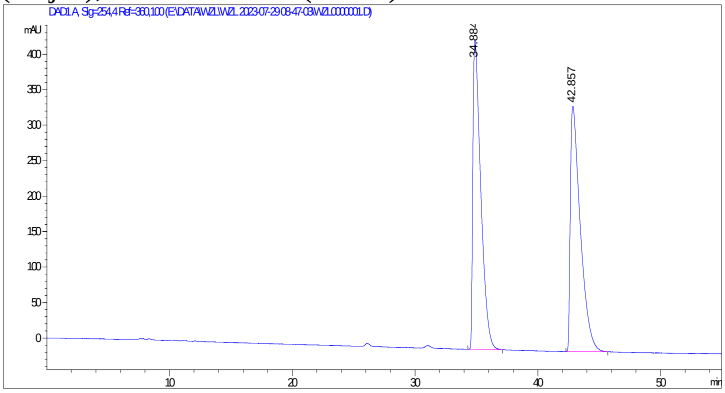

Signal 1: DAD1 A, Sig=254,4 Ref=360,100

Peak #	RetTime [min]	Type	Width [min]	Area [mAU*s]	Height [mAU]	Area %
1	34.884	VB	0.6577	1.95600e4	435.74072	50.0132
2	42.857	BB	0.7716	1.95497e4	345.80411	49.9868
Totals :				3.91096e4	781.54483	

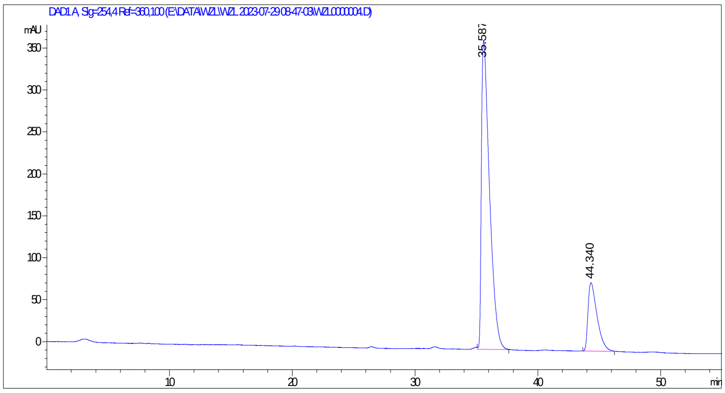

Signal 1: DAD1 A, Sig=254,4 Ref=360,100

Peak #	RetTime [min]	Type	Width [min]	Area [mAU*s]	Height [mAU]	Area %
1	35.587	VB	0.6529	1.69005e4	368.58853	80.1121
2	44.340	VV	0.6133	4195.57813	81.66986	19.8879
Totals :				2.10961e4	450.25839	

(2-(Cyclopentyloxy)phenyl)(4-(*o*-tolyl)but-3-yn-2-yl)silane (8bk)

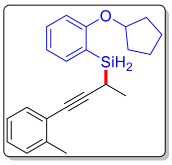

Prepared according to procedure 6 from **1b** (0.2 mmol, 28.4 mg) and **2k** (0.6 mmol, 115.4 mg). The product was isolated in 92% yield with 71% ee value (61.5 mg) as colorless oil.

R*f*: 0.32 (ethyl acetate : petroleum ether = 1:100).

HRMS (ESI) (m/z): Calcd for $C_{22}H_{27}OSi$ $[M+H]^+$: 335.1831, found: 335.1833.

^{1}H NMR (400 MHz, $CDCl_3$) δ 7.55 (dd, *J* = 7.1, 1.8 Hz, 1H), 7.37 (dd, *J* = 7.7, 1.8 Hz, 1H), 7.29 (d, *J* = 7.0 Hz, 1H), 7.15 – 7.09 (m, 2H), 7.08 – 7.03 (m, 1H), 6.92 (t, *J* = 7.2 Hz, 1H), 6.82 (d, *J* = 8.3 Hz, 1H), 4.81 (p, *J* = 4.0 Hz, 1H), 4.41 (dd, *J* = 7.7, 3.0 Hz, 1H), 4.32 (dd, *J* = 7.7, 3.5 Hz, 1H), 2.57 – 2.46 (m, 1H), 2.30 (s, 3H), 1.91 – 1.78 (m, 6H), 1.68 – 1.59 (m, 2H), 1.37 (d, *J* = 7.3 Hz, 3H).

^{13}C NMR (101 MHz, $CDCl_3$) δ 162.9, 140.1, 138.1, 132.2, 131.9, 129.4, 127.4, 125.5, 124.4, 120.4, 120.2, 111.4, 97.4, 79.9, 79.3, 33.1, 33.1, 24.3, 20.9, 17.1, 9.5.

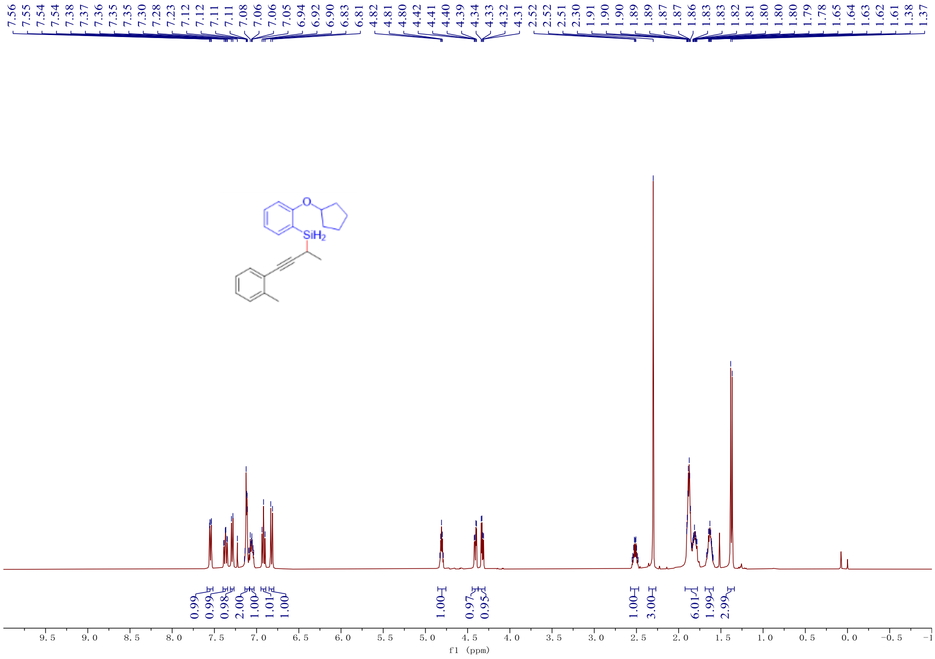

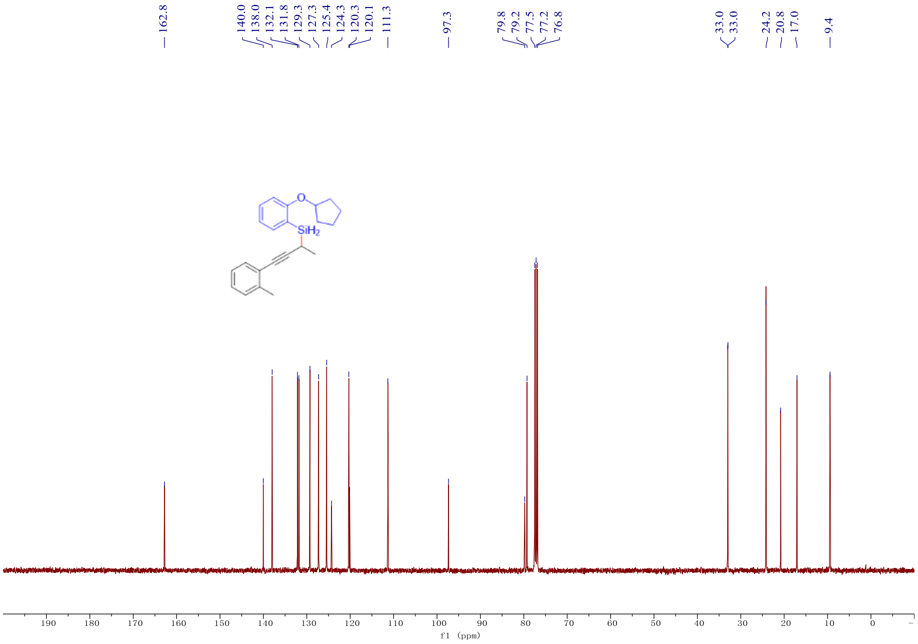

$[\alpha]_D^{20}$ +43.8° (*c* 2.0, ethyl acetate).
The enantiomeric excess of **8bk** was determined by chiral HPLC analysis on Chiralcel OD-3 column.
Conditions: hexane, flow rate = 0.5 mL/min, UV-Vis detection at λ = 254 nm.
t_{R1} = 19.4 min (major), t_{R2} = 22.9 min (minor).

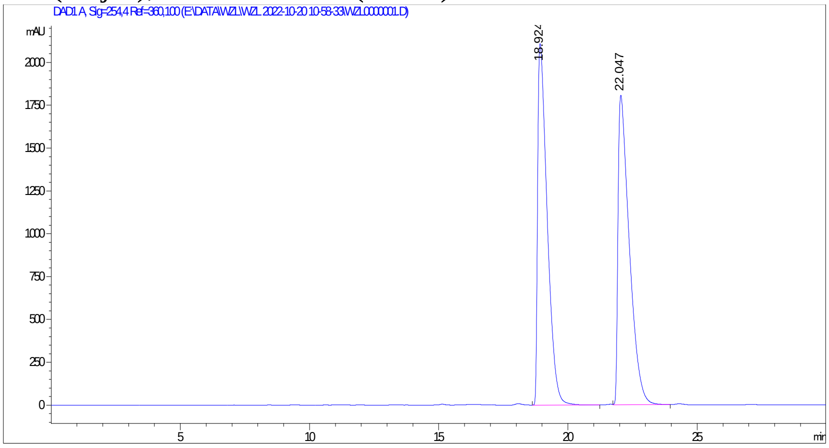

Signal 1: DAD1 A, Sig=254,4 Ref=360,100

Peak #	RetTime [min]	Type	Width [min]	Area [mAU*s]	Height [mAU]	Area %
1	18.924	VB	0.3818	5.42269e4	2110.35913	49.6670
2	22.047	VB	0.4486	5.49539e4	1807.80127	50.3330

Totals : 1.09181e5 3918.16040

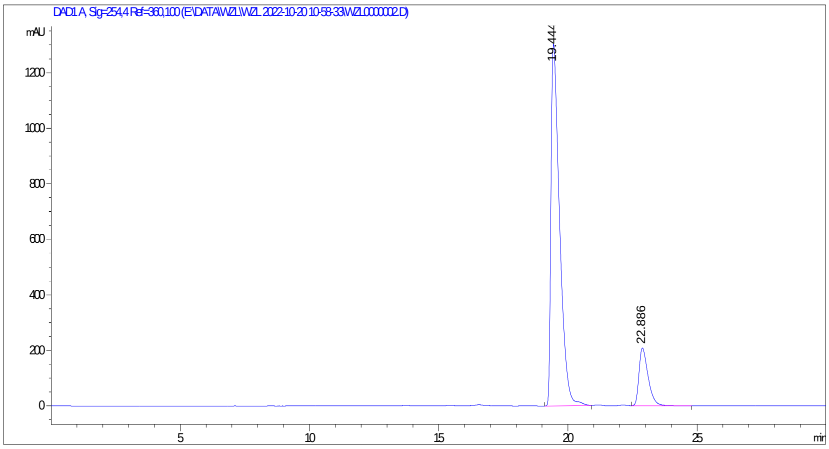

Signal 1: DAD1 A, Sig=254,4 Ref=360,100

Peak #	RetTime [min]	Type	Width [min]	Area [mAU*s]	Height [mAU]	Area %
1	19.444	BB	0.3444	3.02110e4	1303.49268	85.4189
2	22.886	VB	0.3743	5157.05029	208.68921	14.5811

Totals : 3.53681e4 1512.18188

(4-(4-(*tert*-Butyl)phenyl)but-3-yn-2-yl)(2-(cyclopentyloxy)phenyl)silane (8ck)

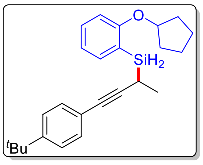

Prepared according to procedure 6 from **1c** (0.2 mmol, 36.8 mg) and **2k** (0.6 mmol, 115.4 mg). The product was isolated in 75% yield with 55% ee value (56.5 mg) as colorless oil.

R*f*: 0.35 (ethyl acetate : petroleum ether = 1:100).

HRMS (ESI) (m/z): Calcd for $C_{25}H_{33}OSi$ $[M+H]^+$: 377.2301, found: 377.2296.

^{1}H NMR (400 MHz, $CDCl_3$) δ 7.56 (dd, *J* = 7.1, 1.8 Hz, 1H), 7.38 (ddd, *J* = 8.3, 7.4, 1.8 Hz, 1H), 7.25 (s, 4H), 6.93 (t, *J* = 7.2 Hz, 1H), 6.83 (d, *J* = 8.3 Hz, 1H), 4.81 (p, *J* = 4.1 Hz, 1H), 4.39 (dd, *J* = 7.8, 2.9 Hz, 1H), 4.30 (dd, *J* = 7.7, 3.5 Hz, 1H), 2.50 – 2.39 (m, 1H), 1.92 – 1.77 (m, 6H), 1.68 – 1.60 (m, 2H), 1.34 (d, *J* = 7.3 Hz, 3H), 1.28 (s, 9H).

^{13}C NMR (101 MHz, $CDCl_3$) δ 162.8, 150.4, 138.0, 132.1, 131.4, 125.2, 121.6, 120.3, 120.2, 111.3, 92.5, 81.0, 79.2, 34.8, 33.0, 33.0, 31.3, 24.2, 16.9, 9.2.

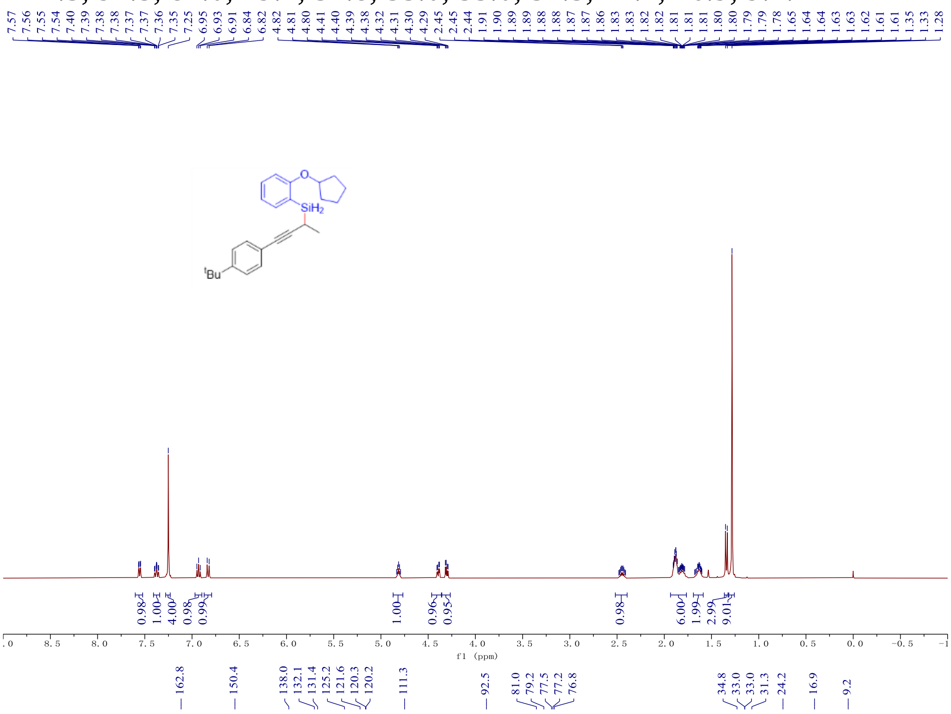

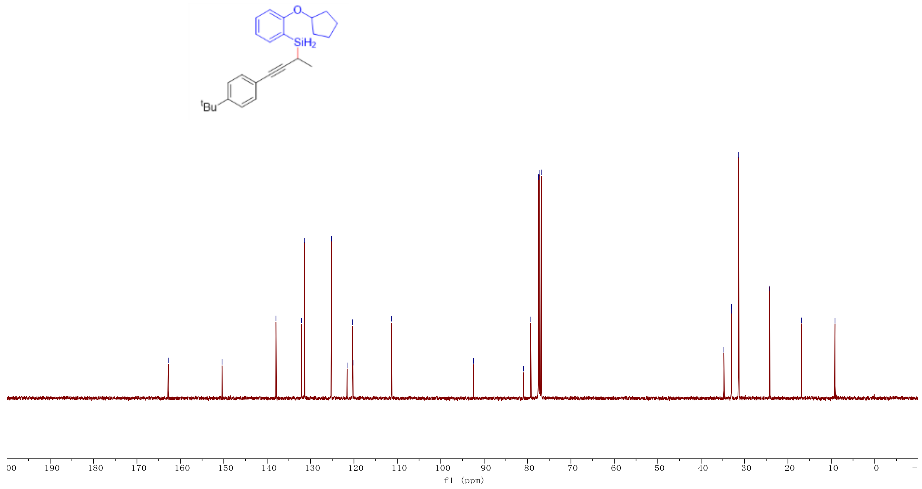

$[\alpha]_D^{20}$ +40.0° (c 0.75, ethyl acetate).

The enantiomeric excess of **8ck** was determined by chiral HPLC analysis on Chiralcel OD-3 column.

Conditions: hexane, flow rate = 0.5 mL/min, UV-Vis detection at λ = 254 nm.

t_{R1} = 16.1 min (major), t_{R2} = 23.0 min (minor).

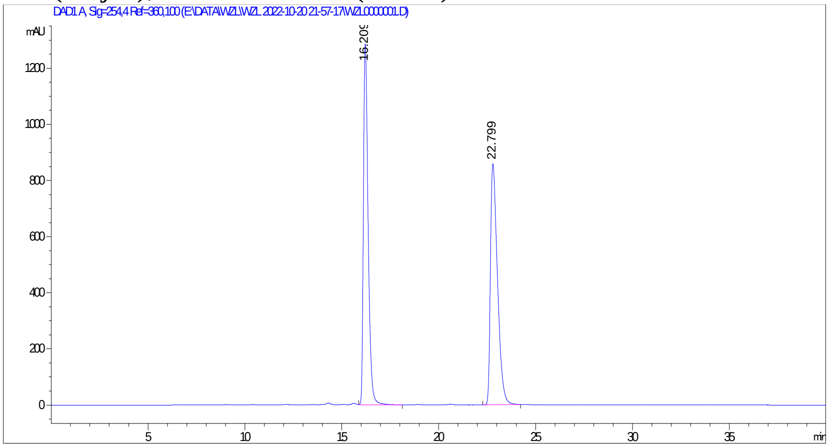

Signal 1: DAD1 A, Sig=254,4 Ref=360,100

Peak #	RetTime [min]	Type	Width [min]	Area [mAU*s]	Height [mAU]	Area %
1	16.209	VB	0.2568	2.17099e4	1286.82361	50.0273
2	22.799	BB	0.3806	2.16862e4	858.69678	49.9727
Totals :				4.33961e4	2145.52039	

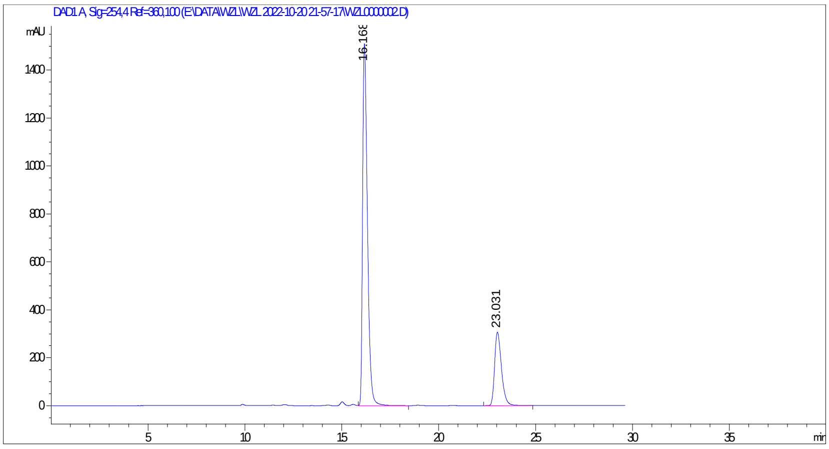

Signal 1: DAD1 A, Sig=254,4 Ref=360,100

Peak #	RetTime [min]	Type	Width [min]	Area [mAU*s]	Height [mAU]	Area %
1	16.168	VB	0.2603	2.59336e4	1510.29565	77.5676
2	23.031	BB	0.3757	7499.94434	306.28253	22.4324
Totals :				3.34335e4	1816.57819	

1-(Buta-1,2-dien-1-yl)-4-(*tert*-butyl)benzene (9)

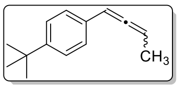

HRMS (ESI) (m/z): Calcd for $C_{14}H_{19}O_3Si$ $[M+H]^+$: 187.1487, found: 187.1490.

^{1}H NMR (400 MHz, $CDCl_3$) δ 7.33 (d, *J* = 8.4 Hz, 2H), 7.22 (d, *J* = 8.4 Hz, 2H), 6.08 (dq, *J* = 6.4, 3.2 Hz, 1H), 5.51 (p, *J* = 6.9 Hz, 1H), 1.77 (dd, *J* = 7.1, 3.2 Hz, 3H), 1.31 (s, 9H).

^{13}C NMR (101 MHz, $CDCl_3$) δ 206.0, 149.9, 132.2, 126.5, 125.6, 93.7, 89.5, 34.7, 31.5, 14.3.

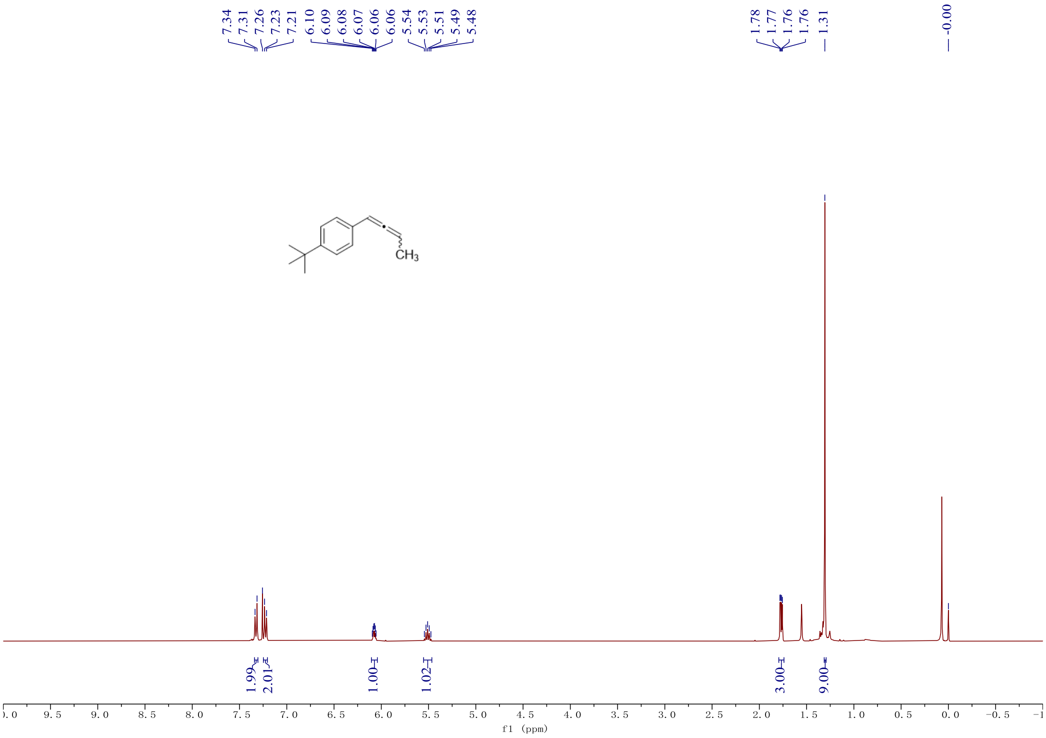

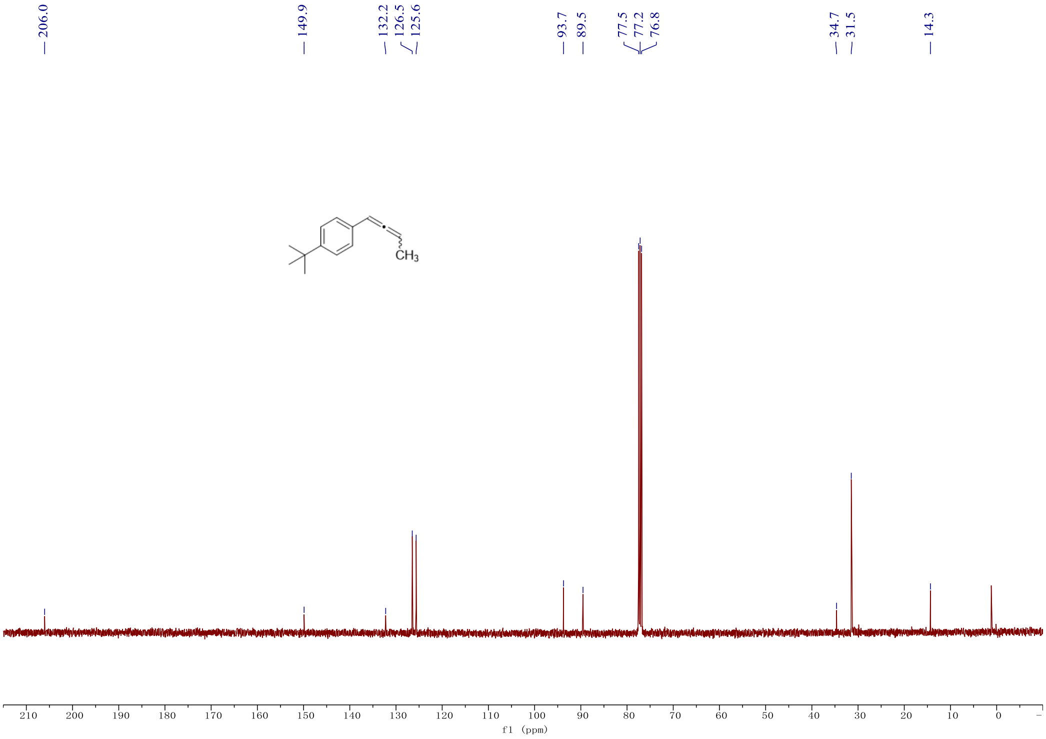

The enantiomeric excess of **9** was determined by chiral HPLC analysis on Chiralcel OJ-3 column.

Conditions: hexane: isopropyl alcohol = 400:1, flow rate = 0.5 mL/min, UV-Vis detection at λ = 254 nm.

t_{R1} = 21.1 min (major), t_{R2} = 34.1 min (minor).

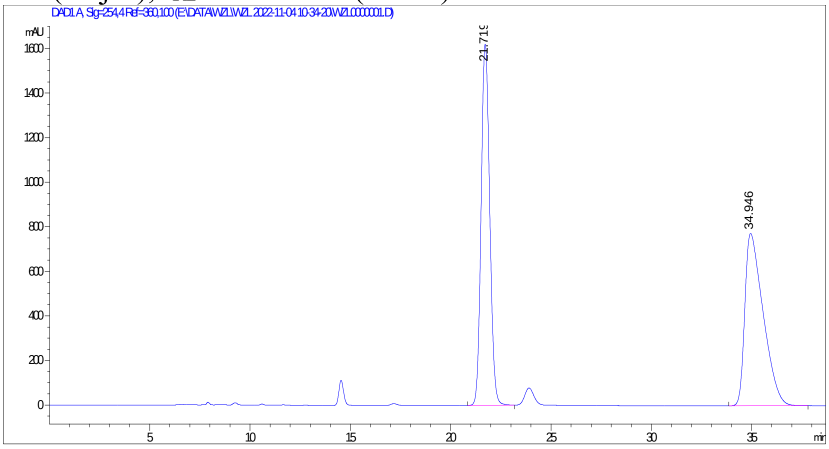

Signal 1: DAD1 A, Sig=254,4 Ref=360,100

Peak #	RetTime [min]	Type	Width [min]	Area [mAU*s]	Height [mAU]	Area %
1	21.719	BB	0.4653	4.82473e4	1620.87170	49.6056
2	34.946	BB	0.9333	4.90146e4	773.50287	50.3944

Totals : 9.72619e4 2394.37457

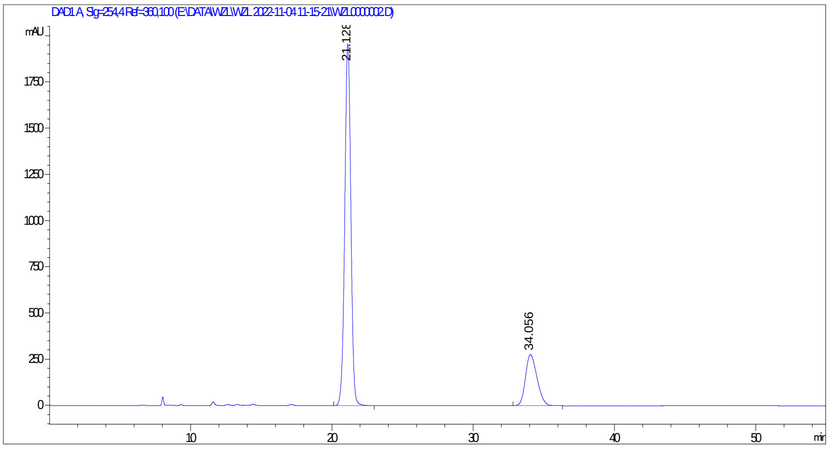

Signal 1: DAD1 A, Sig=254,4 Ref=360,100

Peak #	RetTime [min]	Type	Width [min]	Area [mAU*s]	Height [mAU]	Area %
1	21.128	BB	0.4656	5.86486e4	1957.43738	78.9258
2	34.056	BB	0.8702	1.56599e4	276.72369	21.0742

Totals : 7.43084e4 2234.16107

(2-(Cyclopentyloxy)phenyl)(4-(4-methoxyphenyl)but-3-yn-2-yl)silane (8dk)

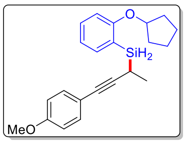

Prepared according to procedure 6 from **1d** (0.2 mmol, 31.6 mg) and **2k** (0.6 mmol, 115.4 mg). The product was isolated in 88% yield with 55% ee value (61.8 mg) as colorless oil.

R*f*: 0.24 (ethyl acetate : petroleum ether = 1:100).

HRMS (ESI) (m/z): Calcd for $C_{22}H_{27}O_2Si$ $[M+H]^+$: 351.1780, found: 351.1777.

^{1}H NMR (400 MHz, $CDCl_3$) δ 7.55 (dd, *J* = 7.1, 1.8 Hz, 1H), 7.37 (ddd, *J* = 8.2, 7.3, 1.8 Hz, 1H), 7.27 – 7.22 (m, 2H), 6.93 (t, *J* = 7.3 Hz, 1H), 6.83 (d, *J* = 8.3 Hz, 1H), 6.79 – 6.75 (m, 2H), 4.81 (p, *J* = 4.1 Hz, 1H), 4.40 (dd, *J* = 7.8, 2.9 Hz, 1H), 4.30 (dd, *J* = 7.7, 3.6 Hz, 1H), 3.78 (s, 3H), 2.49 – 2.40 (m, 1H), 1.92 – 1.78 (m, 6H), 1.67 – 1.60 (m, 2H), 1.34 (d, *J* = 7.3 Hz, 3H).

^{13}C NMR (101 MHz, $CDCl_3$) δ 162.8, 158.9, 138.0, 133.0, 132.1, 120.3, 120.3, 116.8, 113.8, 111.4, 91.6, 80.7, 79.3, 55.4, 33.0, 33.0, 24.2, 16.9, 9.1.

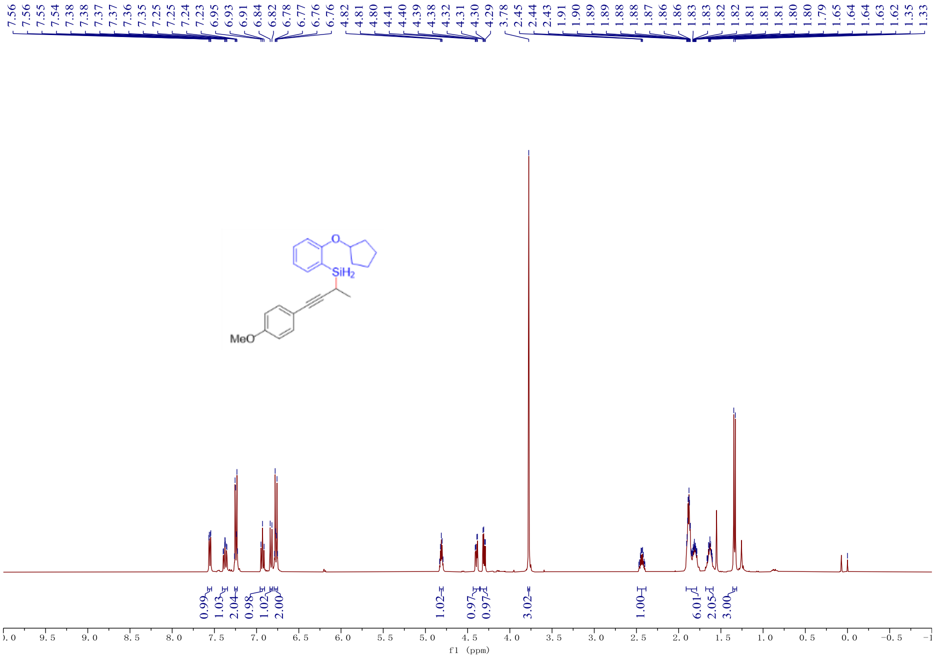

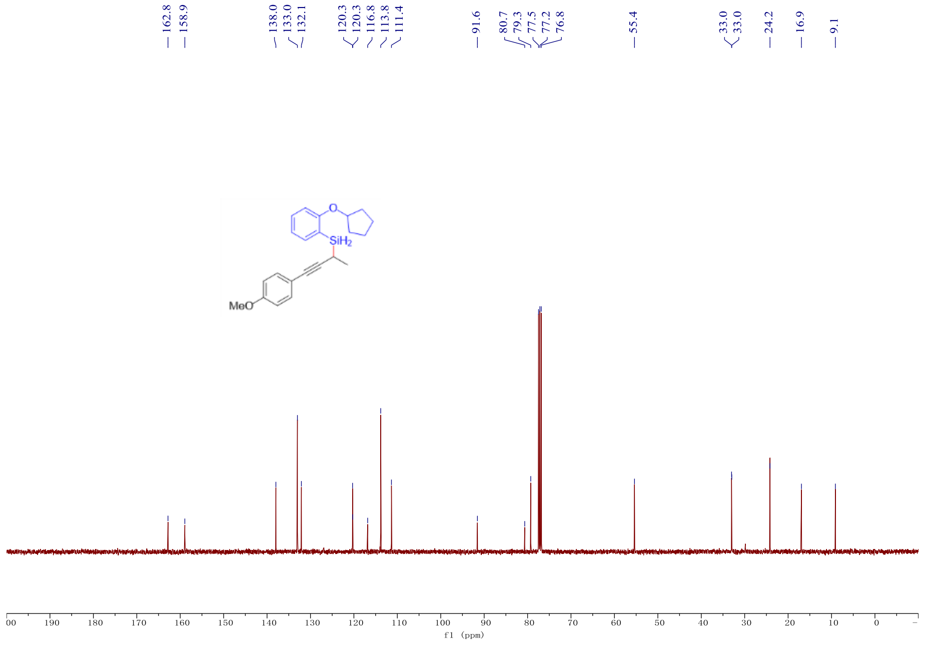

$[\alpha]_D^{20}$ +10.0° (*c* 0.6, ethyl acetate).

The enantiomeric excess of **8dk** was determined by chiral HPLC analysis on Chiralcel OD-3 column.

Conditions: hexane, flow rate = 0.5 mL/min, UV-Vis detection at λ = 254 nm.

t_{R1} = 50.3 min (major), t_{R2} = 59.2 min (minor).

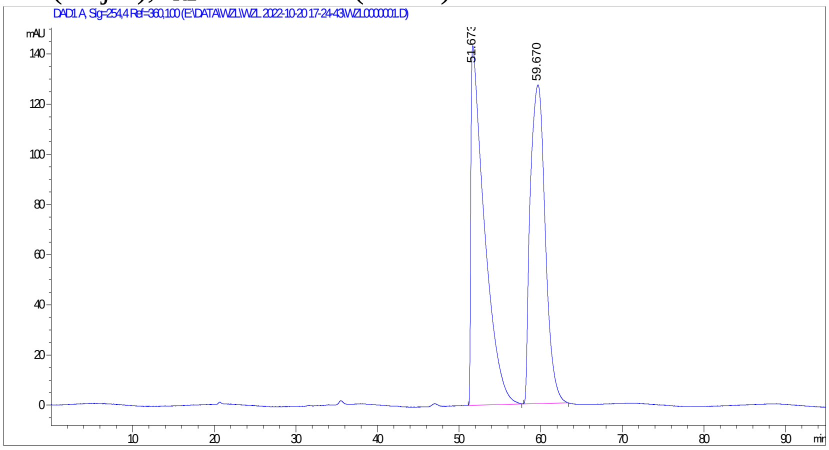

Signal 1: DAD1 A, Sig=254,4 Ref=360,100

Peak #	RetTime [min]	Type	Width [min]	Area [mAU*s]	Height [mAU]	Area %
1	51.673	BB	1.4618	1.64965e4	143.42990	50.5315
2	59.670	BB	1.6721	1.61495e4	127.12213	49.4685

Totals : 3.26460e4 270.55203

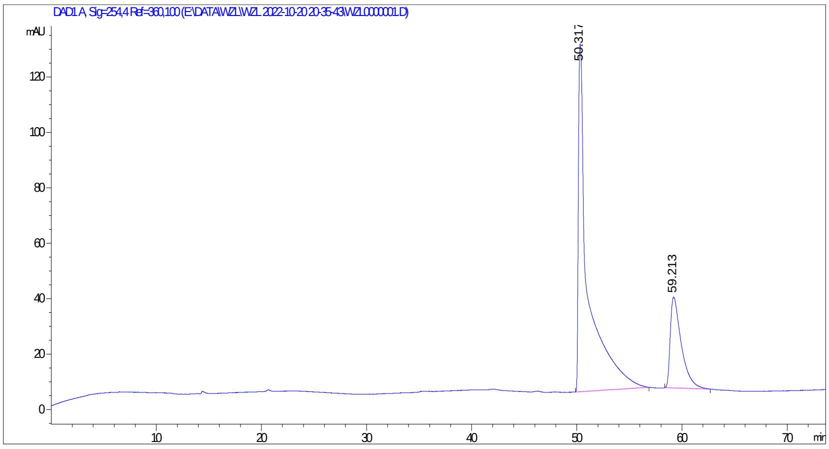

Signal 1: DAD1 A, Sig=254,4 Ref=360,100

Peak #	RetTime [min]	Type	Width [min]	Area [mAU*s]	Height [mAU]	Area %
1	50.317	BB	0.8768	8014.74463	125.55083	76.9840
2	59.213	BB	0.9887	2396.17603	32.86152	23.0160

Totals : 1.04109e4 158.41234

(4-(Benzo[*d*][1,3]dioxol-5-yl)but-3-yn-2-yl)(2-(cyclopentyloxy)phenyl)silane (8ek)

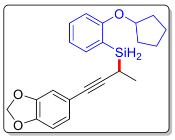

Prepared according to procedure 6 from **1e** (0.2 mmol, 34.4 mg) and **2k** (0.6 mmol, 115.4 mg). The product was isolated in 82% yield with 59% ee value (59.9 mg) as colorless oil.

R*f*: 0.32 (ethyl acetate : petroleum ether = 1:50).

HRMS (ESI) (m/z): Calcd for $C_{22}H_{25}O_3Si$ $[M+H]^+$: 365.1573, found: 365.1561.

^{1}H NMR (400 MHz, $CDCl_3$) δ 7.55 (dd, *J* = 7.2, 1.8 Hz, 1H), 7.38 (ddd, *J* = 8.3, 7.4, 1.8 Hz, 1H), 6.93 (t, *J* = 7.3 Hz, 1H), 6.83 (dd, *J* = 8.0, 1.7 Hz, 2H), 6.76 (d, *J* = 1.5 Hz, 1H), 6.68 (d, *J* = 8.0 Hz, 1H), 5.92 (s, 2H), 4.81 (p, *J* = 4.0 Hz, 1H), 4.38 (dd, *J* = 7.8, 2.9 Hz, 1H), 4.30 (dd, *J* = 7.8, 3.5 Hz, 1H), 2.48 – 2.37 (m, 1H), 1.93 – 1.76 (m, 6H), 1.69 – 1.58 (m, 2H), 1.33 (d, *J* = 7.3 Hz, 3H).

^{13}C NMR (101 MHz, $CDCl_3$) δ 162.8, 147.3, 147.1, 137.9, 132.1, 125.9, 120.3, 120.2, 117.9, 111.8, 111.3, 108.3, 101.2, 91.4, 80.7, 79.2, 33.0, 33.0, 24.2, 16.8, 9.1.

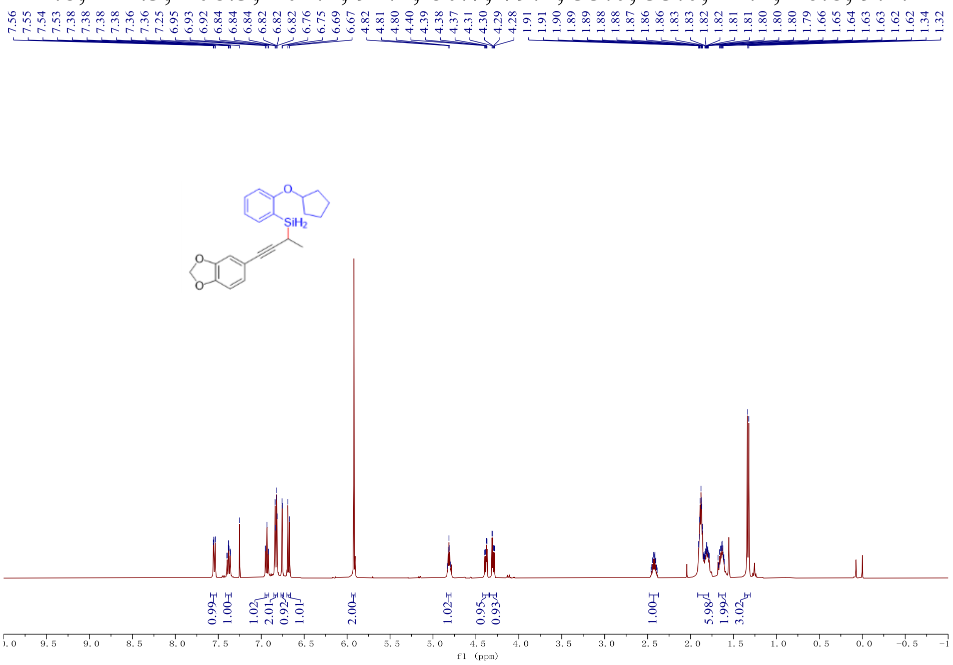

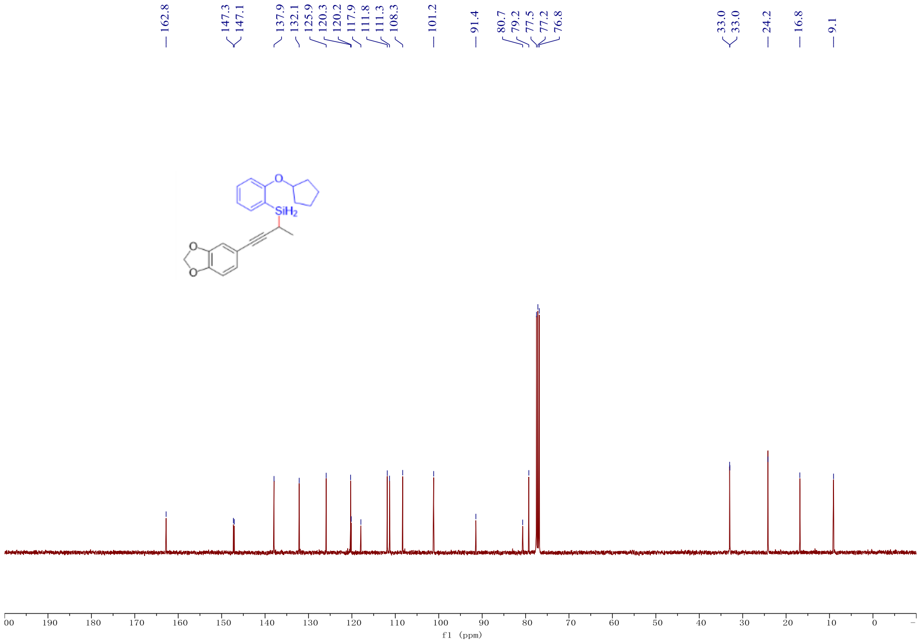

$[\alpha]_D^{20}$ +28.4° (c 2.0, ethyl acetate).

The enantiomeric excess of **8ek** was determined by chiral HPLC analysis on Chiralcel OD-3 column.

Conditions: hexane, flow rate = 0.5 mL/min, UV-Vis detection at λ = 254 nm.

t_{R1} = 49.0 min (major), t_{R2} = 53.1 min (minor).

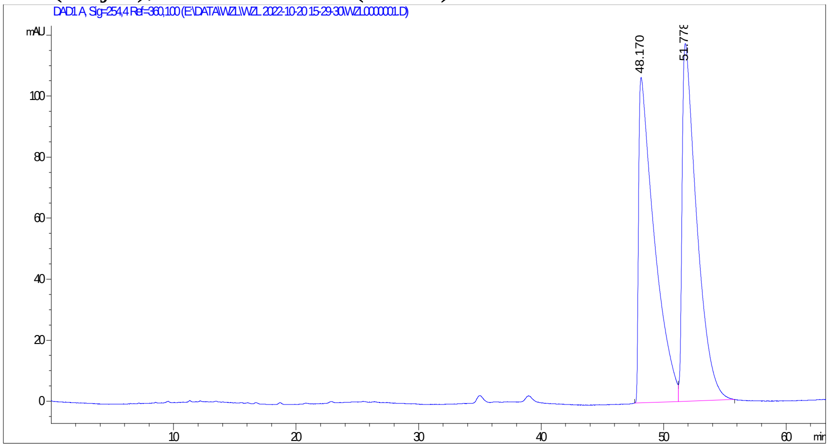

Signal 1: DAD1 A, Sig=254,4 Ref=360,100

Peak #	RetTime [min]	Type	Width [min]	Area [mAU*s]	Height [mAU]	Area %
1	48.170	BV	1.1908	9545.96777	106.59071	49.3969
2	51.778	VB	1.1454	9779.06836	117.20991	50.6031

Totals : 1.93250e4 223.80061

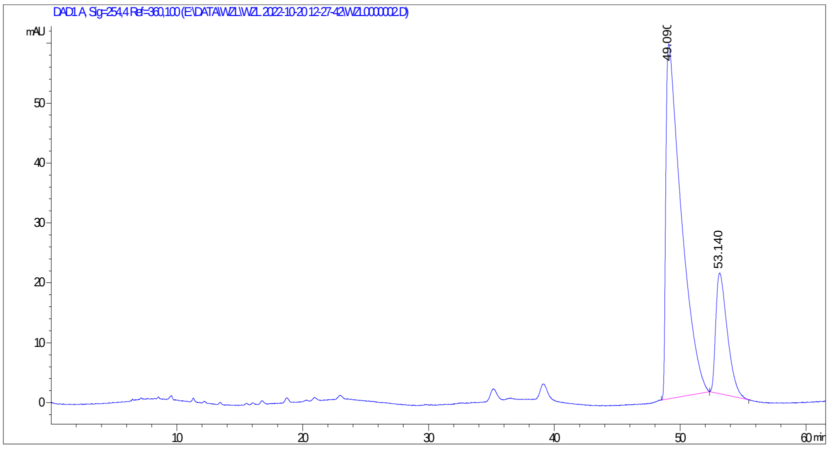

Signal 1: DAD1 A, Sig=254,4 Ref=360,100

Peak #	RetTime [min]	Type	Width [min]	Area [mAU*s]	Height [mAU]	Area %
1	49.090	BB	1.1000	5058.00391	59.24686	79.6501
2	53.140	BB	0.7843	1292.27246	20.14682	20.3499

Totals : 6350.27637 79.39368

(2-(Cyclopentyloxy)phenyl)(4-(thiophen-3-yl)but-3-yn-2-yl)silane (8fk)

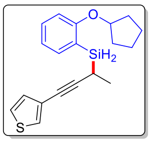

Prepared according to procedure 6 from **1f** (0.2 mmol, 26.8 mg) and **2k** (0.6 mmol, 115.4 mg). The product was isolated in 79% yield with 61% ee value (51.7 mg) as colorless oil.

R*f*: 0.35 (ethyl acetate : petroleum ether = 1:100).

HRMS (ESI) (m/z): Calcd for $C_{19}H_{23}OSSi$ $[M+H]^+$: 327.1239, found: 327.1245.

^{1}H NMR (400 MHz, $CDCl_3$) δ 7.55 (dd, *J* = 7.1, 1.8 Hz, 1H), 7.38 (ddd, *J* = 8.8, 7.4, 1.8 Hz, 1H), 7.26 – 7.23 (m, 1H), 7.18 (dd, *J* = 4.9, 3.0 Hz, 1H), 6.98 (dd, *J* = 4.9, 1.2 Hz, 1H), 6.93 (t, *J* = 7.2 Hz, 1H), 6.83 (d, *J* = 8.3 Hz, 1H), 4.81 (p, *J* = 4.1 Hz, 1H), 4.40 (dd, *J* = 7.8, 2.8 Hz, 1H), 4.30 (dd, *J* = 7.8, 3.6 Hz, 1H), 2.44 (qt, *J* = 7.1, 3.3 Hz, 1H), 1.95 – 1.73 (m, 6H), 1.64 (tdd, *J* = 10.2, 3.9, 1.9 Hz, 2H), 1.34 (d, *J* = 7.3 Hz, 3H).

^{13}C NMR (101 MHz, $CDCl_3$) δ 162.8, 138.0, 132.2, 130.3, 127.3, 124.8, 123.5, 120.3, 120.1, 111.3, 92.7, 79.3, 75.8, 33.0, 33.0, 24.2, 16.8, 9.1.

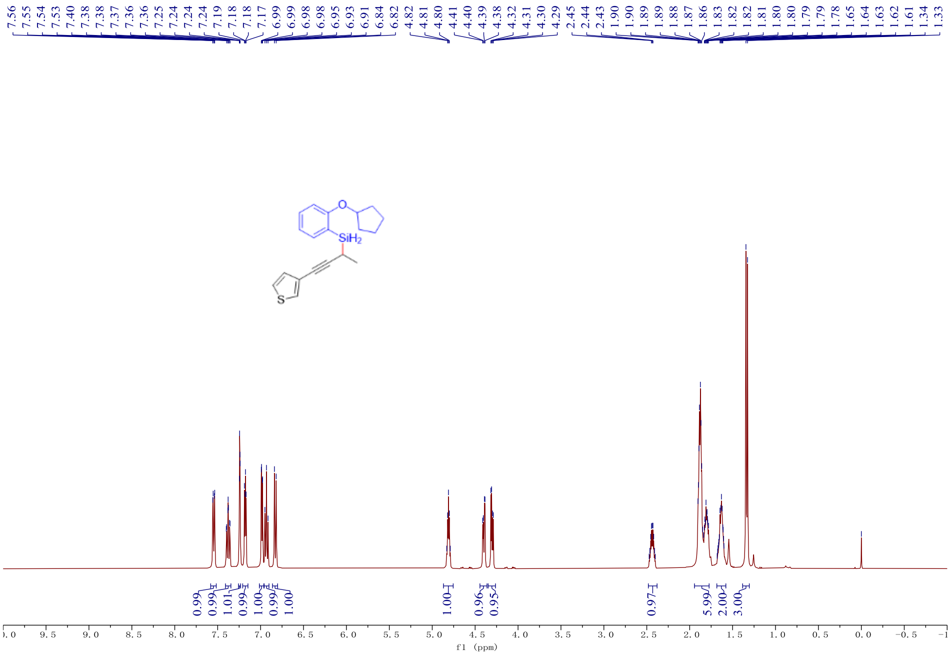

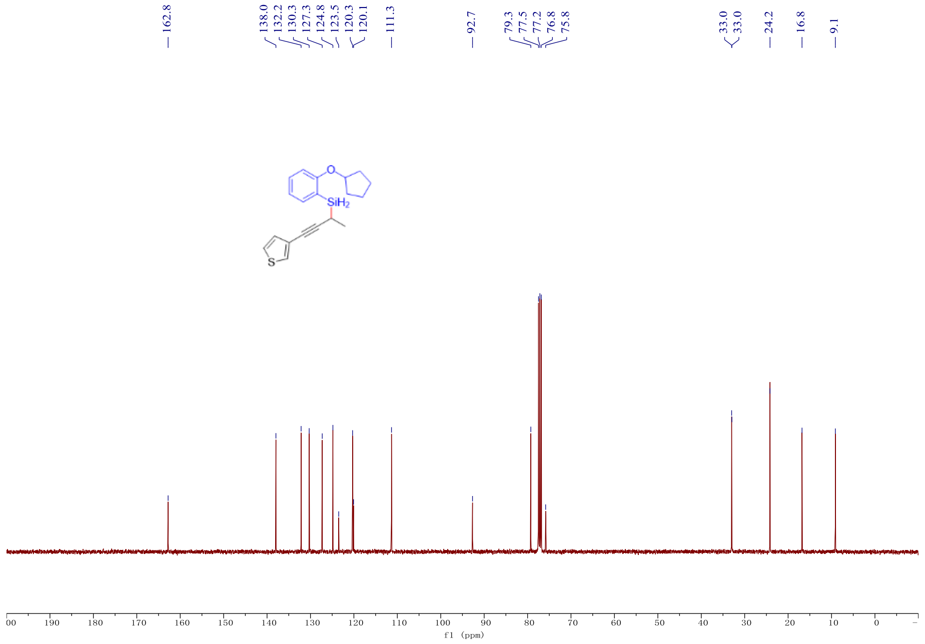

$[\alpha]_D^{20}$ +23.8° (*c* 1.0, ethyl acetate).

The enantiomeric excess of **8fk** was determined by chiral HPLC analysis on Chiralcel OD-3 column.

Conditions: hexane, flow rate = 0.5 mL/min, UV-Vis detection at λ = 254 nm.

t_{R1} = 33.3 min (major), t_{R2} = 44.2 min (minor).

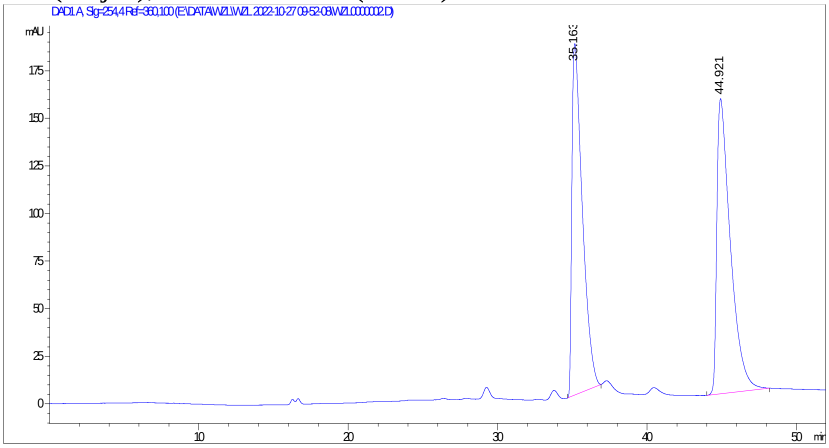

Signal 1: DAD1 A, Sig=254,4 Ref=360,100

Peak #	RetTime [min]	Type	Width [min]	Area [mAU*s]	Height [mAU]	Area %
1	35.163	MM	0.8448	9444.70605	186.33902	49.0276
2	44.921	MM	1.0503	9819.34570	155.81482	50.9724

Totals : 1.92641e4 342.15384

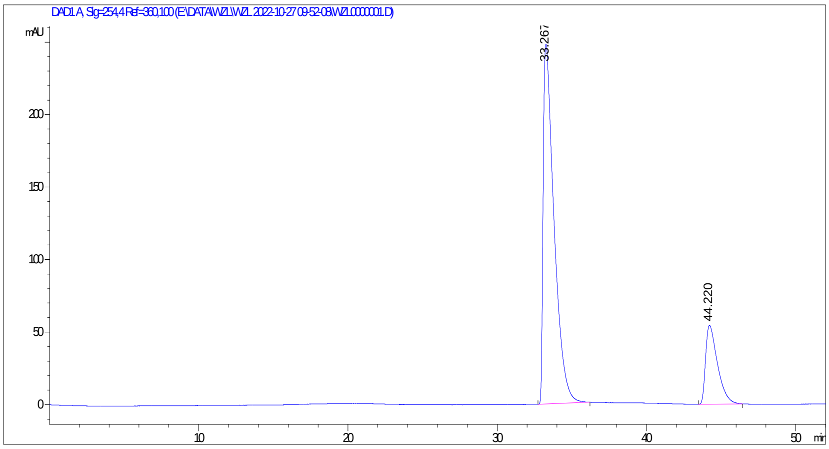

Signal 1: DAD1 A, Sig=254,4 Ref=360,100

Peak #	RetTime [min]	Type	Width [min]	Area [mAU*s]	Height [mAU]	Area %
1	33.267	BB	0.7413	1.25404e4	248.24974	80.5975
2	44.220	BB	0.7947	3018.89014	54.46304	19.4025

Totals : 1.55593e4 302.71278

Dimethoxy(1-(4-methoxyphenyl)buta-1,2-dien-1-yl)(phenyl)silane (13)

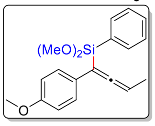

Prepared according to procedure 10. The product was isolated in 66% yield (43.2 mg) as colorless oil.

R*f* : 0.2 (ethyl acetate : petroleum ether = 1:100).

HRMS (ESI) (m/z): Calcd for $C_{19}H_{23}O_3Si$ $[M+H]^+$: 327.1416, found: 327.1425.

^{1}H NMR (400 MHz, $CDCl_3$) δ 7.68 – 7.64 (m, 2H), 7.43 – 7.32 (m, 5H), 6.84 – 6.79 (m, 2H), 5.24 (q, *J* = 6.8 Hz, 1H), 3.77 (s, 3H), 3.62 (s, 6H), 1.70 (d, *J* = 7.0 Hz, 3H).

^{13}C NMR (101 MHz, $CDCl_3$) δ 212.3, 158.5, 134.6, 132.8, 130.4, 128.9, 128.6, 127.9, 114.1, 93.8, 84.1, 55.4, 51.4, 51.3, 13.5.

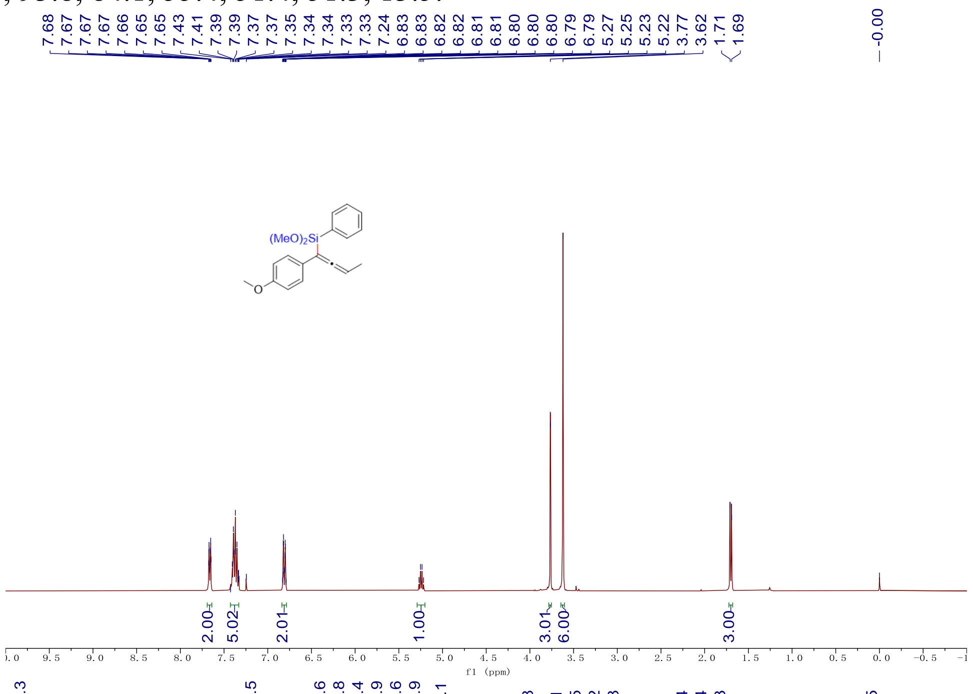

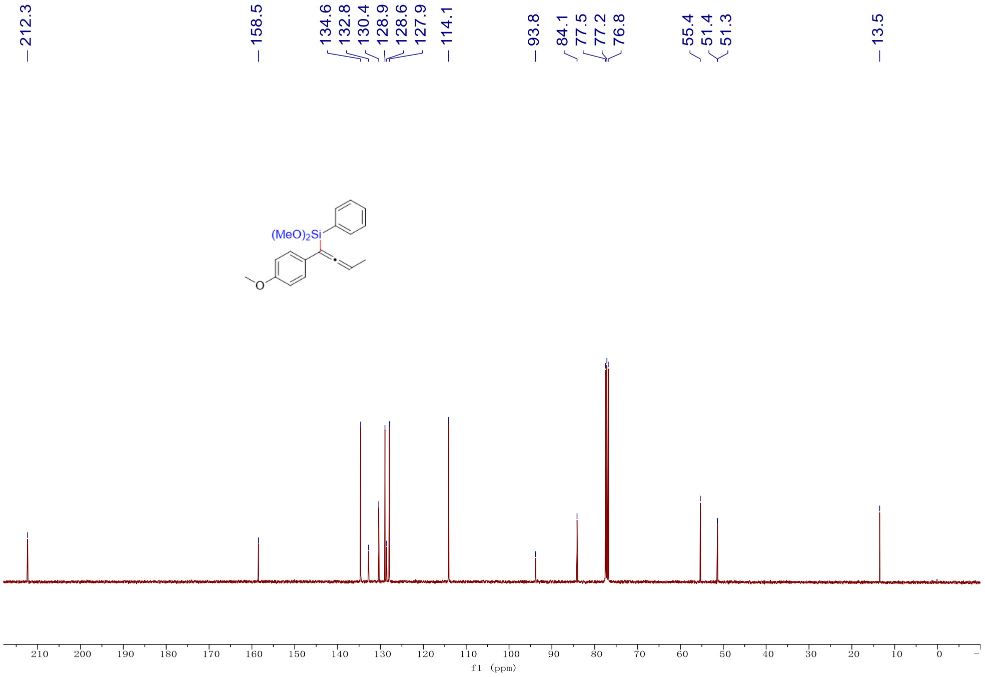

(2-Iodo-6-methoxy-1-methyl-1*H*-inden-3-yl)dimethoxy(phenyl)silane (14)

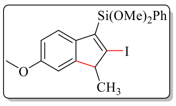

Prepared according to procedure 13 from **13** (0.2 mmol, 65.3 mg). The product was isolated in 84% yield (76.0 mg) as colorless oil.

R*f*: 0.15 (ethyl acetate : petroleum ether = 1:100).

HRMS (ESI) (m/z): Calcd for $C_{19}H_{22}IO_3Si$ $[M+H]^+$: 453.0383, found: 453.0383.

^{1}H NMR (400 MHz, $CDCl_3$) δ 7.75 – 7.71 (m, 3H), 7.45 – 7.40 (m, 1H), 7.37 (m, 2H), 6.96 (d, *J* = 2.4 Hz, 1H), 6.77 (dd, *J* = 8.5, 2.5 Hz, 1H), 3.81 (s, 3H), 3.67 (s, 3H), 3.67 (s, 3H), 3.43 (q, *J* = 7.5 Hz, 1H), 1.32 (d, *J* = 7.6 Hz, 3H).

^{13}C NMR (101 MHz, $CDCl_3$) δ 157.9, 151.1, 141.8, 140.5, 134.9, 131.9, 130.6, 128.0, 123.1, 119.6, 111.9, 109.3, 55.8, 55.7, 50.9, 18.2.

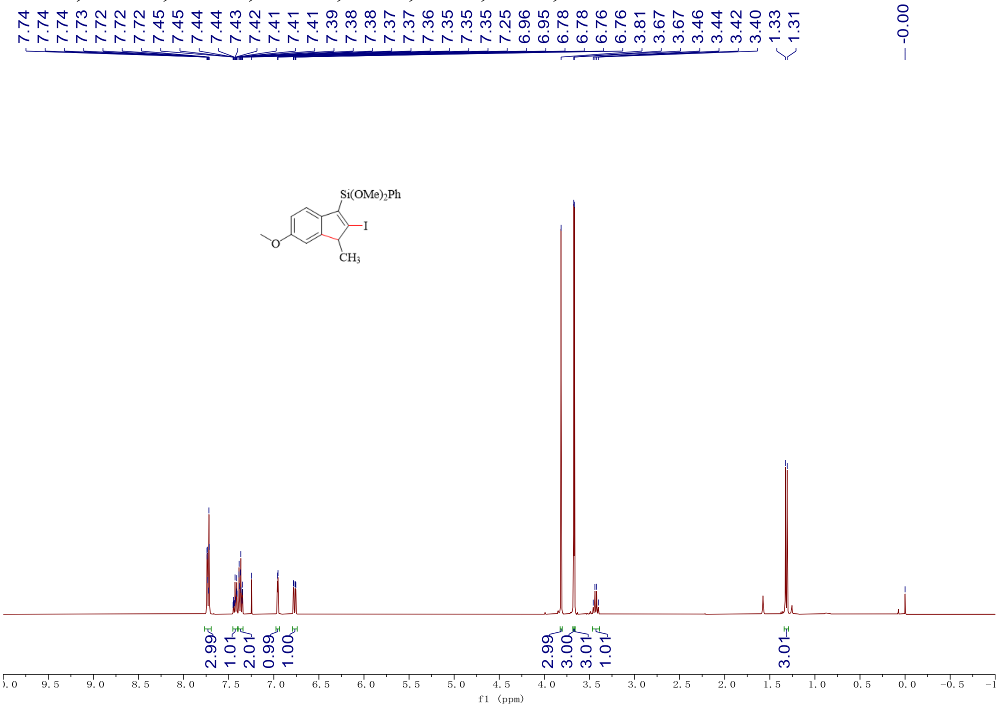

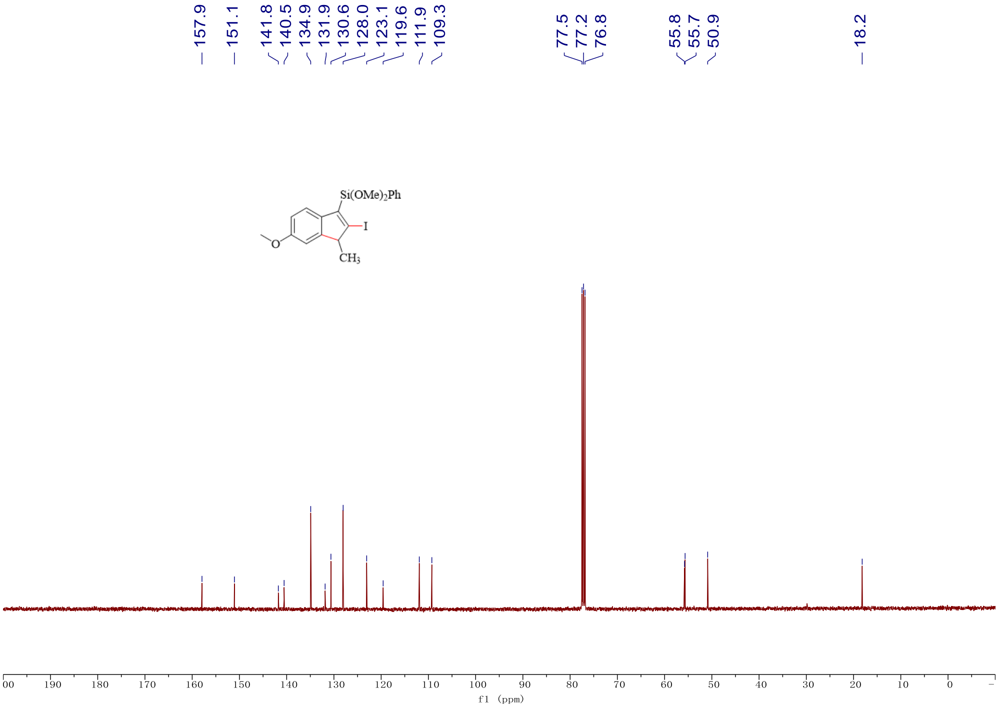

Crystal Data for **6ei**

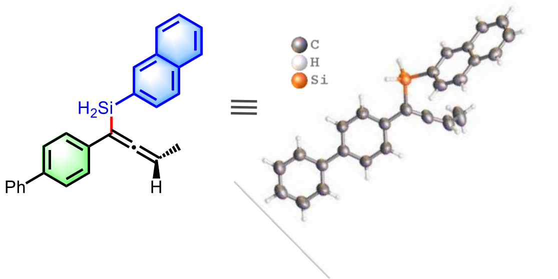

Supplementary Tab 2. ORTEP diagram of complex **6ei**. Thermal ellipsoids are shown at the 35% level

Empirical formula	$C_{26}H_{22}Si$
Formula weight	362.52
Temperature/K	293(2)
Crystal system	monoclinic
Space group	$P2_1$
a/Å	8.0263(3)
b/Å	5.7736(3)
c/Å	22.2010(8)
α/°	90
β/°	97.375(4)
γ/°	90
Volume/Å^3	1020.31(8)
Z	2
ρcalcg/cm^3	1.180
μ/mm-1	1.043
F(000)	384.0
Crystal size/mm^3	0.25 × 0.23 × 0.22
Radiation	Cu Kα (λ = 1.54184)
2Θ range for data collection/°	8.032 to 145.672
Index ranges	$-9 \leq h \leq 9$, $-6 \leq k \leq 6$, $-27 \leq l \leq 25$
Reflections collected	7803
Independent reflections	3743 [R_{int} = 0.0252, R_{sigma} = 0.0348]
Data/restraints/parameters	3743/1/253
Goodness-of-fit on F^2	1.050
Final R indexes [I>=2σ (I)]	R_1 = 0.0416, wR_2 = 0.1097
Final R indexes [all data]	R_1 = 0.0454, wR_2 = 0.1142
Largest diff. peak/hole / e Å^{-3}	0.16/-0.21
Flack parameter	0.012(17)

4. Computational Details.

Calculations were performed with Gaussian 16 [2] software. Geometrical optimizations including transition state optimization were performed using the PBE0 [3] hybrid functional (keyword: PBE1PBE) with DFT-D3 empirical dispersion correction, with Beck-Jonson damping.[4] For C, O, P, Si and H atoms, 6-31G(d) [5,6] basis set was used, while the SDD [7] effective core potential basis set was used for Cu. The SMD solvation model [8] was used (dichloroethane) to account for the solvent surroundings used in the experiment. Harmonic frequency calculations were performed at the same level of theory at 313K on the located transition structures to (1) verify that they are first order saddle points (only one imaginary frequency) and (2) obtain thermodynamic correction for Gibbs free energy used for the final Gibbs free energy calculation. Intrinsic reaction coordinate (IRC) calculation were performed to verify that the transition structures indeed connect to the putative structures starting complex and product complexes, or to provide reasonable initial guesses for them. To obtain more accurate Gibbs free energies, single point energy calculations were performed with MN15 functional [9] and def2-tzvpp [10] basis set and SMD solvation model for dichloroethane. Thus-obtained electronic energies at the SMD (dichloroethane)-MN15/def2-tzvpp level were combined with the thermodynamic corrections for Gibbs free energies obtained in the harmonic frequency calculations above to obtain the final Gibbs free energies. The same level of theory was also used to calculate the electronic energies for the distortion-interaction analysis. For interaction region indicator (IRI) analysis calculation, [11] the wavefunction file was obtained by performing a single point calculation at the same level of theory as the geometric optimization above. The wfn file was then processed by Multiwfn software. [12] The graphs were rendered by VMD. [13] The 3D structures were generated by cylview 1.0. [14]

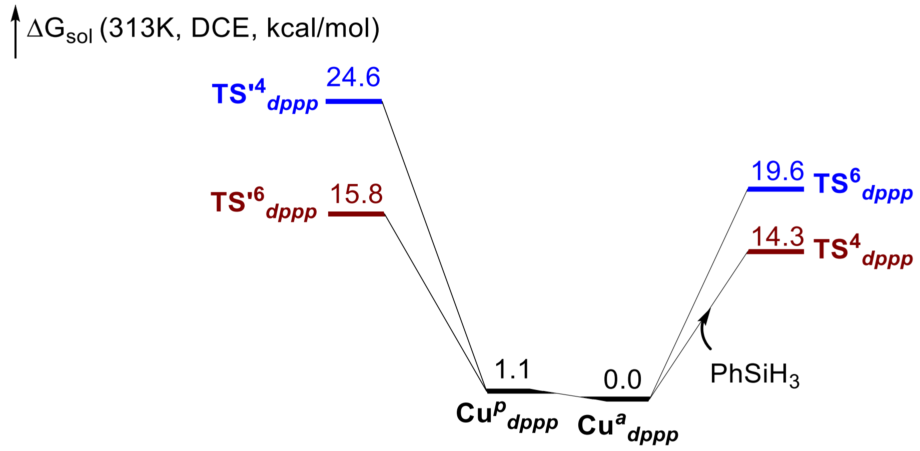

Supplementary Fig 3 Comparison of the TS energies from propargyl and alleneyl copper in the dppp-ligated pathway.

Supplementary Tab 3. Computed electronic energies (E_{sol}), thermal correction to Gibbs free energy (G_c), and Gibbs free energies (G_{sol}) for all stationary points and imaginary frequencies (IFs) of the TSs. (energies in Hartree or kcal/mol).

**Structure**	**E_{sol}(MN15)**	**G_{corr}(PBE0)**	**G_{sol}(MN15)**	**G_{rel}/kcal•mol^{-1}**	**IF/cm^{-1}**
**$PhSiH_3$**	-522.6287345	0.081642	-522.5470925	ref	
**Cu^a_{dppp}**	-3753.043386	0.532886	-3752.5105	0.0	
**Cu^p_{dppp}**	-3753.040774	0.532093	-3752.508681	1.14	
**TS'^4_{dppp}**					
**TS'^6_{dppp}**					
**TS^6_{dppp}**	-4275.668885	0.642538	-4275.026347	19.61	40.34i
**TS^4_{dppp}**	-4275.680427	0.645668	-4275.034759	14.33	95.77i
**Cu^a_{xant}**	-4288.781376	0.667026	-4288.11435	0.0	
**Cu^p_{xant}**	-4288.775363	0.66814	-4288.107223	4.47	
**TS^i_{xant}**	-4288.769824	0.667113	-4288.102711	7.30	70.19i
**TS^6_{xant}**	-4811.4109978	0.776009	-4810.634989	16.60	137.63i
**TS^4_{xant}**	-4811.411555	0.777949	-4810.633606	17.47	27.29i
**Cu^a_{xantcy}**	-4303.229348	0.946987	-4302.282361	0.0	
**Cu^p_{xantcy}**	-4303.218354	0.947067	-4302.271287	6.95	
**TS^i_{xantcy}**	-4303.212782	0.949247	-4302.263535	11.81	79.86i
**TS^6_{xantcy}**	-4825.857759	1.058531	-4824.799228	18.97	71.42i
**TS^4_{xantcy}**	-4825.851781	1.057825	-4824.793956	22.28	98.71i

Cartesian coordinates of the intermediates and transition structures.

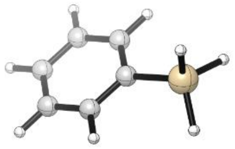

$PhSiH_3$

C	0.0030541	1.5129661	-1.2060610
C	-0.0100089	0.1203361	-1.2045120
C	-0.0123449	-0.5990579	0.0000000
C	-0.0100089	0.1203361	1.2045120
C	0.0030541	1.5129661	1.2060610
C	0.0104151	2.2108011	0.0000000
H	0.0035431	2.0541041	-2.1486530
H	-0.0224909	-0.4094199	-2.1549820
H	-0.0224909	-0.4094199	2.1549820
H	0.0035431	2.0541041	2.1486530
H	0.0177601	3.2977041	0.0000000
Si	0.0066071	-2.4723539	0.0000000
H	1.3965471	-3.0144699	0.0000000
H	-0.6835899	-2.9892979	1.2147900
H	-0.6835899	-2.9892979	-1.2147900

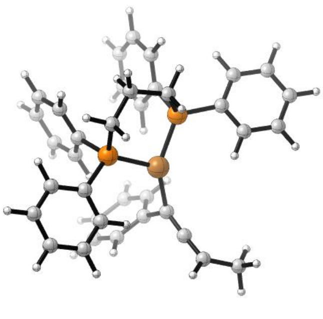

Cu^{a}_{dppp}

C	-0.4691952	-0.9636415	-2.9016926
C	0.9505738	-1.5375795	-2.8405446
C	2.0734658	-0.5598935	-2.4757176
H	-1.1425872	-1.6882995	-3.3764926
H	0.9804158	-2.3970935	-2.1607636
H	1.1791698	-1.9351755	-3.8370436
H	3.0428508	-1.0010915	-2.7365236
H	1.9846448	0.3637615	-3.0617176
H	-0.4821702	-0.0692825	-3.5380766
Cu	0.2064868	1.0279835	-0.1781326
P	2.1117608	-0.0406615	-0.6955436
P	-1.1811542	-0.4138435	-1.2875496
C	3.7327788	0.7950305	-0.5590946
C	4.9504638	0.1144035	-0.4528316
C	3.7292458	2.1944745	-0.5815626
C	6.1450618	0.8249575	-0.3795806
H	4.9681868	-0.9710005	-0.4114996
C	4.9261648	2.9034825	-0.5198366
H	2.7802758	2.7248725	-0.6354536
C	6.1350588	2.2187555	-0.4178186
H	7.0861728	0.2893995	-0.2900366
H	4.9128298	3.9896275	-0.5393486
H	7.0693188	2.7701975	-0.3594706
C	2.2710768	-1.6237035	0.2011334
C	2.9693068	-2.7274015	-0.3062986
C	1.6045398	-1.7472795	1.4254874
C	3.0065768	-3.9239855	0.4034734

H	3.4763218	-2.6647575	-1.2655886
C	1.6435068	-2.9436575	2.1356624
H	1.0322188	-0.9083935	1.8135574
C	2.3437238	-4.0335475	1.6250264
H	3.5478418	-4.7742905	-0.0017126
H	1.1092438	-3.0272945	3.0775144
H	2.3665158	-4.9714515	2.1727914
C	-2.8477222	0.1657935	-1.7620816
C	-3.8165282	-0.6961385	-2.2886386
C	-3.1495232	1.5196125	-1.5843266
C	-5.0687532	-0.2053305	-2.6435646
H	-3.5961572	-1.7544905	-2.4082676
C	-4.4047112	2.0088465	-1.9421136
H	-2.4026072	2.1780535	-1.1443086
C	-5.3625192	1.1482015	-2.4726796
H	-5.8187972	-0.8781745	-3.0501746
H	-4.6355712	3.0610035	-1.7991536
H	-6.3424342	1.5285405	-2.7478556
C	-1.5067932	-1.9395585	-0.3401836
C	-1.3840192	-3.2310415	-0.8612796
C	-1.8671412	-1.7762305	1.0049074
C	-1.6090432	-4.3403275	-0.0477686
H	-1.1107342	-3.3844875	-1.9010926
C	-2.1070222	-2.8842315	1.8088394
H	-1.9508812	-0.7770865	1.4256074
C	-1.9711292	-4.1698575	1.2861104
H	-1.5025142	-5.3396875	-0.4606786

H	-2.3860172	-2.7386075	2.8487924
H	-2.1439132	-5.0369695	1.9175584
C	-0.7745192	5.0044535	0.2665584
H	-1.5913552	5.3261785	-0.3845526
C	0.1867088	6.0740375	0.7120124
H	0.6703648	6.5650395	-0.1442686
H	-0.3190722	6.8649495	1.2832424
H	0.9729438	5.6527615	1.3468504
C	-0.6945272	3.7312225	0.6067414
C	-0.5619982	2.4687865	0.9040524
C	-1.2446632	1.8169125	2.0409114
C	-0.5082982	1.1096085	3.0084824
C	-2.6468502	1.7740085	2.1299074
C	-1.1429102	0.3904605	4.0167234
H	0.5782908	1.1300115	2.9547324
C	-3.2819412	1.0447955	3.1321074
H	-3.2342232	2.3030505	1.3841104
C	-2.5360232	0.3453245	4.0803964
H	-0.5464012	-0.1448575	4.7516474
H	-4.3684422	1.0191135	3.1695064
H	-3.0332422	-0.2273115	4.8584744

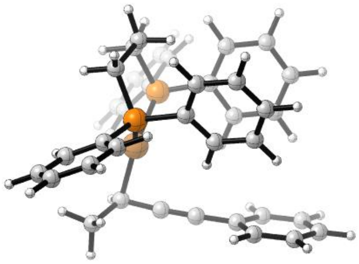

Cu^{p}$_{\textit{dppp}}$

C	-1.0036851	-2.6984950	1.6921076

C	0.2490189	-2.2867520	2.4717936
C	1.5764019	-2.3751930	1.7137656
H	-1.8397041	-2.8566730	2.3841286
H	0.1231889	-1.2762110	2.8799116
H	0.3250129	-2.9536160	3.3394706
H	2.4123419	-2.3289510	2.4214686
H	1.6587519	-3.3421070	1.2009596
H	-0.8367041	-3.6599210	1.1895686
Cu	0.1466819	-1.2166340	-1.1202024
P	1.8192889	-1.0809680	0.4086426
P	-1.5296471	-1.5242470	0.3622256
C	3.5592459	-1.3781810	-0.0738034
C	4.6380069	-0.6376380	0.4180786
C	3.7984229	-2.4063550	-0.9946884
C	5.9353749	-0.9278490	0.0007316
H	4.4655969	0.1704670	1.1232146
C	5.0956319	-2.7034160	-1.3996324
H	2.9595839	-2.9649840	-1.4052254
C	6.1671539	-1.9619710	-0.9033324
H	6.7670419	-0.3431440	0.3843886
H	5.2693379	-3.5045520	-2.1127274
H	7.1798269	-2.1853830	-1.2272044
C	1.8373439	0.4901610	1.3356526
C	2.2984119	0.6020920	2.6536046
C	1.3369539	1.6258140	0.6890676
C	2.2575989	1.8292540	3.3093336
H	2.6898189	-0.2669910	3.1754096

C	1.3042249	2.8541980	1.3431036
H	0.9536549	1.5397300	-0.3256414
C	1.7610839	2.9559050	2.6546586
H	2.6124019	1.9053990	4.3334046
H	0.9064229	3.7232780	0.8268926
H	1.7258939	3.9112670	3.1710336
C	-3.1180021	-2.2559360	-0.1782744
C	-4.2620321	-2.2557360	0.6277556
C	-3.1679871	-2.8525260	-1.4418354
C	-5.4352941	-2.8523010	0.1771486
H	-4.2407991	-1.7764450	1.6034416
C	-4.3420231	-3.4544770	-1.8899234
H	-2.2814471	-2.8333010	-2.0728174
C	-5.4754901	-3.4545800	-1.0806064
H	-6.3214151	-2.8452280	0.8059076
H	-4.3729571	-3.9157260	-2.8731574
H	-6.3937051	-3.9179020	-1.4308534
C	-2.0140851	-0.0064220	1.2566396
C	-2.3427541	0.0277750	2.6166296
C	-2.0314391	1.1851200	0.5219776
C	-2.6826891	1.2330840	3.2267396
H	-2.3303171	-0.8808020	3.2121766
C	-2.3827151	2.3862630	1.1289936
H	-1.7438971	1.1717040	-0.5269814
C	-2.7044461	2.4121320	2.4845816
H	-2.9289461	1.2495790	4.2848496
H	-2.3833341	3.3008270	0.5425206

H	-2.9651461	3.3511580	2.9650546
C	0.2365409	-1.0436450	-3.1315324
H	-0.6002321	-1.5694020	-3.6058734
C	1.5718999	-1.3767450	-3.7893054
H	1.7513669	-2.4580820	-3.7712274
H	1.6238849	-1.0526060	-4.8413894
H	2.4053109	-0.9043030	-3.2571264
C	-0.0430781	0.3363840	-2.9549264
C	-0.2635551	1.5234610	-2.7098754
C	-0.5338181	2.8746260	-2.3943654
C	0.5099119	3.7773100	-2.0967784
C	-1.8593231	3.3575900	-2.3399944
C	0.2345939	5.0927730	-1.7448094
H	1.5370729	3.4257150	-2.1375344
C	-2.1232351	4.6747530	-1.9855234
H	-2.6759631	2.6797090	-2.5724184
C	-1.0816281	5.5519790	-1.6805764
H	1.0569669	5.7664040	-1.5170134
H	-3.1534241	5.0199900	-1.9474994
H	-1.2923511	6.5804970	-1.4023584

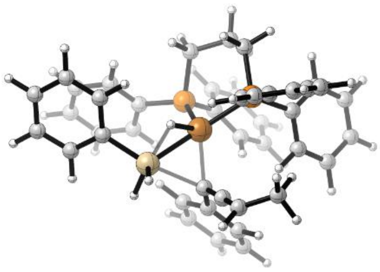

TS^{4}$_{dppp}$

C	0.9887605	0.1513886	-2.8556225
C	-0.4626875	0.2826556	-3.3309455

C	-1.4278865	-0.8088834	-2.8587685
H	1.6373885	0.7691056	-3.4873715
H	-0.8504285	1.2748566	-3.0680845
H	-0.4488535	0.2475966	-4.4269045
H	-2.3517035	-0.7790654	-3.4486395
H	-0.9853375	-1.7986764	-3.0299505
H	1.3246745	-0.8868844	-2.9644945
Cu	0.0276605	-0.7700924	0.1844755
P	-1.8483425	-0.7789614	-1.0609945
P	1.2569005	0.6424006	-1.0875155
C	-2.9921665	-2.1954954	-0.9022485
C	-4.3654415	-2.0788474	-1.1408265
C	-2.4618095	-3.4342044	-0.5255785
C	-5.1954175	-3.1880844	-1.0042475
H	-4.7881775	-1.1177634	-1.4212865
C	-3.2922115	-4.5444894	-0.4009535
H	-1.3986085	-3.5173454	-0.3125255
C	-4.6605385	-4.4215604	-0.6358315
H	-6.2623095	-3.0886204	-1.1841255
H	-2.8724395	-5.5021574	-0.1058485
H	-5.3109495	-5.2849284	-0.5265335
C	-2.9150135	0.6835276	-0.8312445
C	-3.5105265	1.3906126	-1.8798745
C	-3.1073365	1.1256566	0.4847645
C	-4.2818075	2.5212006	-1.6154235
H	-3.3752065	1.0712496	-2.9089465
C	-3.8870575	2.2456876	0.7480945

H	-2.6153855	0.6007496	1.2996255
C	-4.4729185	2.9489796	-0.3040045
H	-4.7331905	3.0681086	-2.4384085
H	-4.0210985	2.5796346	1.7732875
H	-5.0723595	3.8322316	-0.1022755
C	3.0727795	0.7814446	-0.9623695
C	3.6494115	1.8115406	-0.2082835
C	3.9021595	-0.1878024	-1.5374555
C	5.0295035	1.8711336	-0.0381165
H	3.0171895	2.5746046	0.2371905
C	5.2819985	-0.1200114	-1.3719175
H	3.4780485	-1.0133664	-2.0995375
C	5.8493045	0.9070196	-0.6212595
H	5.4642045	2.6766726	0.5474645
H	5.9109385	-0.8839064	-1.8184945
H	6.9265465	0.9547326	-0.4885325
C	0.7047515	2.3839086	-1.0892165
C	1.2053195	3.3055906	-2.0190165
C	-0.2588815	2.7972486	-0.1676015
C	0.7372415	4.6145296	-2.0287325
H	1.9690305	3.0021936	-2.7308805
C	-0.7283535	4.1090916	-0.1779135
H	-0.6363565	2.0929566	0.5649465
C	-0.2332975	5.0166456	-1.1089825
H	1.1287965	5.3234776	-2.7529665
H	-1.4816275	4.4131896	0.5428305
H	-0.5982905	6.0400666	-1.1198065

C	-2.1752055	-2.1067764	3.1378305
H	-1.9021775	-2.8243274	3.9153595
C	-3.5988625	-2.1440814	2.6550865
H	-3.8340605	-3.1154824	2.2024445
H	-4.2990895	-1.9921064	3.4865235
H	-3.7888775	-1.3754984	1.9020225
C	-1.2407005	-1.2876984	2.7081535
C	-0.2427815	-0.5593084	2.2624145
C	0.0474775	0.7957826	2.7875055
C	-0.9266815	1.5257356	3.4877075
C	1.2710805	1.4297376	2.5231635
C	-0.6942725	2.8367796	3.8918535
H	-1.8830975	1.0529196	3.6998545
C	1.5116925	2.7339226	2.9406295
H	2.0371845	0.8932316	1.9719425
C	0.5281995	3.4498556	3.6210075
H	-1.4732085	3.3828476	4.4183595
H	2.4698605	3.1985396	2.7208035
H	0.7103935	4.4744846	3.9331545
C	2.9858575	-2.6491944	0.7721065
C	2.9257735	-3.3440894	-0.4429015
C	4.0742715	-3.8330364	-1.0620685
C	5.3217905	-3.6221864	-0.4751405
C	5.4085215	-2.9230134	0.7273185
C	4.2507745	-2.4434324	1.3370375
H	1.9563425	-3.4969744	-0.9183845
H	4.0004015	-4.3726414	-2.0036705

H	6.2223725	-3.9942774	-0.9574405
H	6.3795435	-2.7449294	1.1834915
H	4.3297085	-1.8872804	2.2706995
Si	1.4133395	-2.0243174	1.6760155
H	2.1650025	-1.2033004	2.7048665
H	0.5306045	-2.3561114	0.2969055
H	0.8602285	-3.1843144	2.4432285

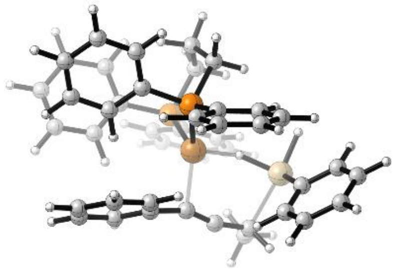

TS^{6}$_{dppp}$

C	-0.3072383	-0.6761695	-2.7675090
C	1.1635827	-0.3921835	-3.0899740
C	1.7385767	0.9266875	-2.5630450
H	-0.6818173	-1.4642765	-3.4307670
H	1.7831277	-1.2365945	-2.7637970
H	1.2527877	-0.3613175	-4.1825810
H	2.6753697	1.1603615	-3.0826400
H	1.0483877	1.7492335	-2.7898320
H	-0.9136413	0.2180505	-2.9563980
Cu	0.0301717	0.5960545	0.2288620
P	2.0336457	1.0289905	-0.7382050
P	-0.6078523	-1.1825535	-1.0093700
C	2.7957537	2.6850075	-0.5857100
C	4.1270887	2.8736315	-0.2027440
C	1.9908237	3.8053665	-0.8342470

C	4.6435967	4.1611575	-0.0718000
H	4.7622927	2.0149475	-0.0060860
C	2.5128057	5.0888925	-0.7124520
H	0.9489087	3.6743865	-1.1152130
C	3.8405827	5.2698885	-0.3275530
H	5.6793157	4.2956695	0.2279090
H	1.8776237	5.9480545	-0.9091210
H	4.2462287	6.2724065	-0.2250520
C	3.3979317	-0.1287315	-0.3936800
C	4.2157047	-0.6840065	-1.3827750
C	3.6078967	-0.4873985	0.9436140
C	5.2136207	-1.5931785	-1.0386170
H	4.0805137	-0.4153455	-2.4260110
C	4.6162517	-1.3799695	1.2886950
H	2.9618277	-0.0761405	1.7145100
C	5.4161677	-1.9416525	0.2947890
H	5.8359077	-2.0275625	-1.8158820
H	4.7652567	-1.6497565	2.3305360
H	6.1946247	-2.6519135	0.5586110
C	-2.3244873	-1.7982745	-1.0345610
C	-2.6678163	-3.0183205	-0.4399330
C	-3.3347863	-0.9925485	-1.5744320
C	-3.9974123	-3.4305195	-0.4012060
H	-1.8940213	-3.6485015	-0.0107670
C	-4.6597113	-1.4145455	-1.5450830
H	-3.0965063	-0.0231915	-2.0030920
C	-4.9947663	-2.6331515	-0.9580760

H	-4.2519433	-4.3802015	0.0616300
H	-5.4316313	-0.7759475	-1.9621540
H	-6.0314283	-2.9570755	-0.9291930
C	0.4078557	-2.6890205	-0.8467130
C	0.3749037	-3.7090315	-1.8075720
C	1.2621597	-2.8108935	0.2513740
C	1.1941517	-4.8241565	-1.6732780
H	-0.2963383	-3.6351185	-2.6594820
C	2.0820187	-3.9292145	0.3858940
H	1.2855307	-2.0291885	1.0038350
C	2.0516767	-4.9330445	-0.5769690
H	1.1657127	-5.6096645	-2.4232980
H	2.7454787	-4.0064805	1.2420090
H	2.6935237	-5.8039815	-0.4761890
C	-1.5298163	3.1782795	1.8649360
H	-2.5281493	3.4002185	2.2591410
C	-0.5411643	4.3174735	2.0703900
H	-0.9415003	5.2442255	1.6438420
H	-0.3166043	4.5019045	3.1288690
H	0.4063577	4.1025255	1.5602210
C	-1.0729753	1.9085035	2.2305530
C	-0.6503703	0.7379065	2.2521870
C	-0.2610483	-0.5324215	2.8048410
C	0.9629407	-0.6948785	3.4755980
C	-1.0910473	-1.6549505	2.6317910
C	1.3476467	-1.9464655	3.9470870
H	1.6027167	0.1704585	3.6244060

C	-0.7064663	-2.8972305	3.1202320
H	-2.0299623	-1.5388305	2.0981350
C	0.5170737	-3.0523345	3.7719030
H	2.3002657	-2.0556735	4.4586300
H	-1.3599543	-3.7534725	2.9765940
H	0.8214267	-4.0281335	4.1390740
C	-3.6953553	2.2073165	-0.0442740
C	-4.6462693	2.4133645	-1.0562080
C	-5.9484853	1.9294845	-0.9480000
C	-6.3235493	1.1885865	0.1711170
C	-5.3906283	0.9474115	1.1765190
C	-4.0992833	1.4612725	1.0728930
H	-4.3578743	2.9670555	-1.9478240
H	-6.6679813	2.1234935	-1.7400940
H	-7.3339623	0.7964485	0.2552790
H	-5.6689913	0.3584095	2.0471300
H	-3.3912053	1.2591175	1.8705740
Si	-1.9770413	2.9992855	-0.2808960
H	-1.9376323	4.5156675	-0.2731490
H	-0.6823173	2.1325885	-0.3130140
H	-2.0367783	2.9254325	-1.8376900

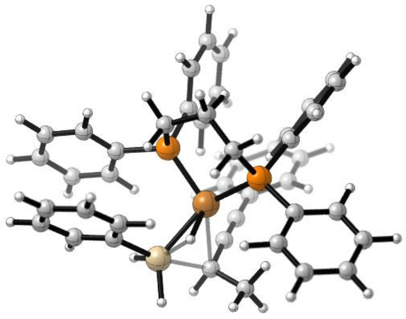

TS'[4] ***dppp***

C	-1.5555642	0.4020658	-2.3204664
C	-0.3687472	1.1995398	-2.8769664
C	0.1355738	2.3802488	-2.0430794
H	-1.9578762	-0.2277622	-3.1223284
H	0.4693638	0.5286558	-3.1006684
H	-0.6915272	1.6049598	-3.8433474
H	0.7469578	3.0360648	-2.6734034
H	-0.7055582	2.9816928	-1.6779104
H	-2.3637382	1.0781528	-2.0156444
Cu	-0.3846702	0.7967898	0.6870656
P	1.1338858	1.8992648	-0.5569554
P	-1.1978172	-0.6447882	-0.8467774
C	1.7931458	3.4999218	0.0179466
C	3.1630788	3.7626928	0.1093326
C	0.8781878	4.4717988	0.4462336
C	3.6100258	4.9849618	0.6076126
H	3.8825938	3.0142938	-0.2092804
C	1.3283578	5.6937328	0.9334176
H	-0.1886102	4.2624148	0.4164426
C	2.6962198	5.9531018	1.0154026
H	4.6769118	5.1791608	0.6744446

H	0.6102618	6.4412728	1.2587216
H	3.0473688	6.9055688	1.4021566
C	2.5486738	1.0030418	-1.2663004
C	3.2542478	1.4655498	-2.3854724
C	2.9031118	-0.2185082	-0.6879004
C	4.2956458	0.7105348	-2.9140654
H	2.9942088	2.4166928	-2.8429484
C	3.9469468	-0.9733362	-1.2175284
H	2.3467278	-0.5822922	0.1708936
C	4.6408788	-0.5099932	-2.3314034
H	4.8382428	1.0722698	-3.7829074
H	4.2034378	-1.9252102	-0.7622224
H	5.4515228	-1.0992812	-2.7509894
C	-2.7224582	-1.6031482	-0.5462904
C	-3.7744512	-1.6929122	-1.4602034
C	-2.8070882	-2.2939642	0.6685206
C	-4.8951282	-2.4638452	-1.1623144
H	-3.7385472	-1.1547412	-2.4019594
C	-3.9206232	-3.0749262	0.9569016
H	-2.0048242	-2.2040242	1.3968836
C	-4.9688422	-3.1588362	0.0419536
H	-5.7134742	-2.5188202	-1.8748394
H	-3.9762282	-3.6067662	1.9026356
H	-5.8443182	-3.7601982	0.2706996
C	-0.0452152	-1.9283232	-1.4572304
C	0.1791888	-2.1891432	-2.8125264
C	0.6326958	-2.6855112	-0.4969604

C	1.0840008	-3.1772902	-3.1940724
H	-0.3451452	-1.6291622	-3.5807834
C	1.5253798	-3.6814392	-0.8770904
H	0.4752778	-2.4761492	0.5563906
C	1.7590228	-3.9224082	-2.2295714
H	1.2590438	-3.3652312	-4.2497814
H	2.0488128	-4.2501632	-0.1136904
H	2.4669918	-4.6888432	-2.5326504
C	-0.3187782	0.8448988	3.1759006
H	-0.8737322	0.5860338	4.0888216
C	0.4517948	2.1493858	3.3764126
H	-0.2433882	2.9935208	3.4568336
H	1.0643208	2.1258508	4.2875376
H	1.1163718	2.3574298	2.5328026
C	0.5310158	-0.2709572	2.8632366
C	1.2756478	-1.2279182	2.6909386
C	2.1439108	-2.3415492	2.5431546
C	3.4945058	-2.1677612	2.1848916
C	1.6687908	-3.6516702	2.7485266
C	4.3299898	-3.2663762	2.0222986
H	3.8773328	-1.1627572	2.0344996
C	2.5138708	-4.7435942	2.5913476
H	0.6292338	-3.7982012	3.0275206
C	3.8466598	-4.5593862	2.2220876
H	5.3684718	-3.1105512	1.7422786
H	2.1276828	-5.7461692	2.7541216
H	4.5031718	-5.4153162	2.0956396

C	-4.8679792	0.5106308	1.1656736
C	-5.9802252	0.6100028	0.3322516
C	-5.9915032	1.5398878	-0.7063064
C	-4.8858802	2.3673148	-0.8997444
C	-3.7806872	2.2538958	-0.0565204
C	-3.7449722	1.3293518	0.9950556
H	-4.8598312	-0.2391972	1.9555146
H	-6.8329552	-0.0484242	0.4809626
H	-6.8531282	1.6151408	-1.3651854
H	-4.8850052	3.0950348	-1.7086144
H	-2.9149332	2.8972978	-0.2234904
Si	-2.2272422	1.1859628	2.1875696
H	-2.6921452	-0.1403142	2.7852596
H	-1.3631102	2.1220558	1.1083786
H	-2.4828162	2.2690188	3.1952176

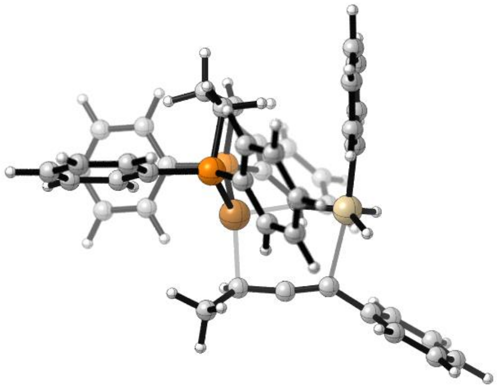

TS'^{6}$_{dppp}$

C	-1.7858749	-1.4016625	1.6657387
C	-2.5196369	-0.0995555	1.9990137
C	-1.6380409	1.1541465	2.0590607
H	-2.4111639	-2.2504405	1.9655607
H	-3.3623179	0.0501095	1.3106597

H	-2.9692409	-0.2284405	2.9908767
H	-2.1151309	1.9299315	2.6699497
H	-0.6875429	0.9144225	2.5514747
H	-0.8489869	-1.4678515	2.2331867
Cu	-0.2727299	0.2408375	-0.8697093
P	-1.1867979	1.8514615	0.4127557
P	-1.3305689	-1.6134065	-0.1198633
C	-0.1584979	3.2954715	0.8494647
C	-0.6557389	4.3737475	1.5901867
C	1.1824461	3.2909575	0.4502637
C	0.1829811	5.4279055	1.9369587
H	-1.7014279	4.3947495	1.8883977
C	2.0216971	4.3455635	0.8045387
H	1.5664441	2.4608795	-0.1394373
C	1.5232151	5.4121975	1.5481117
H	-0.2075749	6.2640405	2.5103567
H	3.0639941	4.3318525	0.4976057
H	2.1762411	6.2359825	1.8225777
C	-2.7447279	2.5511205	-0.2368173
C	-3.8792689	2.7912445	0.5452687
C	-2.8040709	2.8022955	-1.6148843
C	-5.0481439	3.2737695	-0.0395883
H	-3.8646169	2.5954445	1.6132407
C	-3.9685729	3.2927025	-2.1965033
H	-1.9325019	2.5987425	-2.2335583
C	-5.0953569	3.5249775	-1.4087143
H	-5.9245399	3.4505995	0.5775177

H	-4.0000719	3.4829705	-3.2655233
H	-6.0095619	3.8968765	-1.8624373
C	-0.3708819	-3.1646495	-0.1014243
C	-0.6578159	-4.2399825	0.7488597
C	0.7561451	-3.2333795	-0.9277213
C	0.1619741	-5.3644965	0.7612117
H	-1.5140619	-4.2015505	1.4161177
C	1.5751911	-4.3595815	-0.9169303
H	1.0065901	-2.3833385	-1.5573903
C	1.2789661	-5.4261725	-0.0715303
H	-0.0676659	-6.1917405	1.4269347
H	2.4520251	-4.3953795	-1.5572123
H	1.9211471	-6.3022345	-0.0543763
C	-2.9346759	-1.9942675	-0.9104613
C	-3.4595999	-3.2861735	-1.0204903
C	-3.6720509	-0.9136195	-1.4133653
C	-4.7038329	-3.4894415	-1.6118253
H	-2.8928409	-4.1373655	-0.6542763
C	-4.9222869	-1.1174335	-1.9904533
H	-3.2639119	0.0926885	-1.3593613
C	-5.4397349	-2.4070465	-2.0916023
H	-5.0996589	-4.4976355	-1.6980953
H	-5.4839189	-0.2676995	-2.3689513
H	-6.4107659	-2.5703645	-2.5505993
C	0.6885191	0.5936885	-2.7337193
H	0.3102141	1.6140615	-2.8653623
C	0.1443681	-0.4131705	-3.7347313

H	-0.9504149	-0.4568575	-3.6963963
H	0.4256271	-0.1574625	-4.7662633
H	0.5214801	-1.4199695	-3.5292753
C	1.9692941	0.5116325	-2.2610763
C	3.0553361	0.3723305	-1.6266573
C	4.4776921	0.5394465	-1.8114553
C	5.3027501	-0.5633845	-2.0920813
C	5.0855461	1.7925045	-1.6181733
C	6.6807211	-0.4090955	-2.2063933
H	4.8481791	-1.5412445	-2.2260813
C	6.4637491	1.9392005	-1.7313533
H	4.4604051	2.6506485	-1.3849133
C	7.2709921	0.8412505	-2.0279823
H	7.2981781	-1.2742845	-2.4351033
H	6.9108231	2.9196595	-1.5864303
H	8.3476071	0.9582285	-2.1146973
C	1.8291981	-2.3554265	2.5372807
C	1.2492401	-2.7941555	3.7275467
C	0.8885791	-1.8722925	4.7088427
C	1.1201491	-0.5135655	4.4944207
C	1.7000111	-0.0877805	3.2997377
C	2.0601611	-0.9945405	2.2908457
H	2.0896981	-3.0906505	1.7771287
H	1.0723131	-3.8556735	3.8853397
H	0.4331501	-2.2094725	5.6365517
H	0.8486881	0.2116625	5.2583017
H	1.8693481	0.9787935	3.1465027

Si	2.6327411	-0.3737595	0.5655447
H	1.2108841	-0.1502425	0.0747647
H	3.4140421	0.8460235	0.9436357
H	3.4180811	-1.5588775	0.0989117

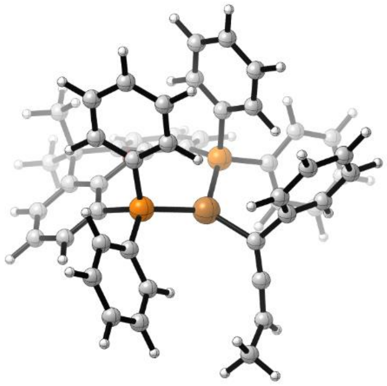

Cu^{a}$_{xant}$

C	0.0695861	-4.0087734	-1.2574500
C	0.3327801	-2.7551994	-0.6952190
C	1.6702461	-2.3772534	-0.5573170
C	2.7422481	-3.1594094	-0.9873070
C	2.4392371	-4.3937994	-1.5615770
C	1.1167391	-4.8191184	-1.6842480
C	4.1487271	-2.6204074	-0.7490100
C	4.0914051	-1.1111124	-0.9587490
C	2.9542081	-0.4444954	-0.5078870
C	2.7953501	0.9417916	-0.5618000
C	3.8420361	1.6937816	-1.1018450
C	4.9856461	1.0572806	-1.5778470
C	5.1089401	-0.3297884	-1.5086770
H	-0.9574929	-4.3389384	-1.3796170
H	3.2390101	-5.0375524	-1.9138520
H	0.9022001	-5.7884004	-2.1251940

H	3.7557881	2.7745806	-1.1603970
H	5.7915651	1.6477366	-2.0041950
H	6.0123401	-0.8021264	-1.8813770
P	-0.9628799	-1.5324864	-0.2737580
P	1.1983861	1.6227676	0.0108790
C	-2.4601379	-2.2649264	-1.0185950
C	-3.3281589	-3.1224304	-0.3355590
C	-2.7426099	-1.9130044	-2.3444820
C	-4.4687409	-3.6115124	-0.9682810
H	-3.1197899	-3.4007524	0.6935850
C	-3.8739179	-2.4165264	-2.9798530
H	-2.0806809	-1.2304804	-2.8725740
C	-4.7427699	-3.2605434	-2.2891140
H	-5.1435089	-4.2702234	-0.4279730
H	-4.0854059	-2.1368764	-4.0084100
H	-5.6345489	-3.6420564	-2.7791930
C	-1.2086999	-1.7065314	1.5288070
C	-2.3274829	-1.0837184	2.1010570
C	-0.2854079	-2.3512014	2.3562580
C	-2.5253459	-1.1258014	3.4766700
H	-3.0478729	-0.5717254	1.4680390
C	-0.4787539	-2.3741694	3.7361770
H	0.5858601	-2.8393534	1.9289020
C	-1.5992889	-1.7680664	4.2988440
H	-3.4015729	-0.6466054	3.9052900
H	0.2484631	-2.8736274	4.3708430
H	-1.7494599	-1.7917154	5.3748990

C	1.3944901	3.4164576	-0.2702440
C	2.0736911	4.2571676	0.6183910
C	0.8346491	3.9524326	-1.4361180
C	2.1950521	5.6158996	0.3390340
H	2.5048771	3.8501396	1.5293080
C	0.9670921	5.3097096	-1.7178420
H	0.2851081	3.3047546	-2.1157720
C	1.6454631	6.1422836	-0.8295250
H	2.7199171	6.2649666	1.0350040
H	0.5301141	5.7175756	-2.6253370
H	1.7406741	7.2034106	-1.0441240
C	1.2850451	1.4302786	1.8272040
C	0.0899551	1.5462936	2.5472480
C	2.4738401	1.1535756	2.5094130
C	0.0865771	1.4007296	3.9306520
H	-0.8418349	1.7394516	2.0210950
C	2.4651181	0.9970306	3.8937710
H	3.4069141	1.0554156	1.9611120
C	1.2740301	1.1224006	4.6058730
H	-0.8475499	1.4881286	4.4780980
H	3.3925051	0.7769576	4.4161180
H	1.2694791	0.9961416	5.6852490
O	1.9084201	-1.1571294	0.0217660
Cu	-0.7345829	0.7085526	-0.7477810
C	-2.5785739	2.7829516	-3.6569340
H	-2.8755899	2.1980426	-4.5309820
C	-2.2447399	4.2310636	-3.8995310

H	-1.3954869	4.3425286	-4.5899230
H	-3.0877309	4.7729376	-4.3515660
H	-1.9867649	4.7372776	-2.9632440
C	-2.5363379	2.1945326	-2.4726700
C	-2.4037529	1.6296196	-1.3069570
C	-3.4816279	1.5146956	-0.3063680
C	-4.5508339	0.6168176	-0.4709950
C	-3.4169229	2.2381426	0.8982470
C	-5.5019629	0.4397806	0.5313390
H	-4.6185809	0.0409646	-1.3899360
C	-4.3744379	2.0692996	1.8939200
H	-2.5973639	2.9382056	1.0464640
C	-5.4202669	1.1610046	1.7222500
H	-6.3110369	-0.2711854	0.3791610
H	-4.2995829	2.6463456	2.8129660
H	-6.1612949	1.0194706	2.5046160
C	5.1816581	-3.2887934	-1.6499780
H	6.1816451	-2.8956284	-1.4428980
H	5.2188881	-4.3653664	-1.4579570
H	4.9582441	-3.1315044	-2.7108020
C	4.5216001	-2.8821514	0.7257880
H	4.5333491	-3.9596884	0.9254300
H	5.5166821	-2.4757914	0.9397490
H	3.8055421	-2.4134234	1.4093230

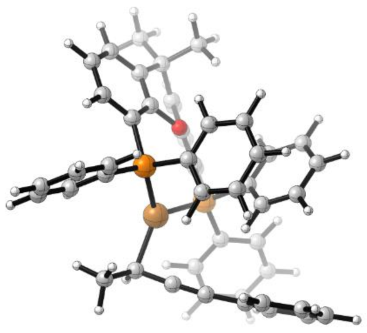

Cu^{p}$_{xant}$

C	-0.0954517	-3.9180519	-1.4258246
C	0.2021953	-2.6963529	-0.8180536
C	1.4800333	-2.5330709	-0.2824106
C	2.4652293	-3.5165799	-0.3088056
C	2.1406293	-4.7177549	-0.9421426
C	0.8754663	-4.9147449	-1.4951546
C	3.7702963	-3.2216509	0.4228964
C	4.0860203	-1.7449799	0.2070744
C	3.0151653	-0.8515019	0.1695784
C	3.1592843	0.5246931	-0.0190666
C	4.4612863	1.0292981	-0.1096506
C	5.5527483	0.1686661	-0.0519266
C	5.3669413	-1.2062449	0.0875464
H	-1.0810517	-4.0836669	-1.8506876
H	2.8744963	-5.5156779	-0.9981616
H	0.6421633	-5.8597079	-1.9775136
H	4.6187933	2.0940161	-0.2494916
H	6.5590303	0.5703231	-0.1291026
H	6.2334623	-1.8596379	0.1080774
P	-0.9189377	-1.2579489	-0.7052836

P	1.6541613	1.5322451	-0.3065746
C	-2.4684337	-1.8921899	-1.4423896
C	-3.4108517	-2.6199859	-0.7074766
C	-2.7142387	-1.6198739	-2.7926756
C	-4.5805237	-3.0675129	-1.3162706
H	-3.2358927	-2.8292589	0.3440844
C	-3.8818317	-2.0724579	-3.4015336
H	-1.9908587	-1.0427559	-3.3636196
C	-4.8183677	-2.7937249	-2.6625646
H	-5.3095097	-3.6272819	-0.7362506
H	-4.0650687	-1.8514929	-4.4496196
H	-5.7346077	-3.1386389	-3.1343996
C	-1.3195707	-1.1969389	1.0809374
C	-2.0243267	-0.0799479	1.5416524
C	-0.9884417	-2.2170289	1.9783594
C	-2.4140367	0.0052461	2.8744514
H	-2.2725357	0.7209601	0.8519184
C	-1.3600987	-2.1183339	3.3174814
H	-0.4451857	-3.0931389	1.6350744
C	-2.0786417	-1.0119769	3.7662274
H	-2.9718257	0.8730731	3.2143694
H	-1.0940447	-2.9138139	4.0087784
H	-2.3735607	-0.9409799	4.8098844
C	2.3869653	3.1103991	-0.8717216
C	2.6478333	4.1883511	-0.0204766
C	2.7207963	3.2105591	-2.2279676
C	3.2269513	5.3521101	-0.5226846

H	2.3993183	4.1216871	1.0348204
C	3.3107643	4.3683631	-2.7247366
H	2.5113333	2.3770941	-2.8949726
C	3.5603183	5.4435191	-1.8724716
H	3.4219403	6.1870851	0.1451014
H	3.5655323	4.4357231	-3.7790006
H	4.0118043	6.3523691	-2.2611586
C	0.9986813	1.9235351	1.3516724
C	-0.0851347	2.8109981	1.4246574
C	1.4896233	1.3416081	2.5225654
C	-0.6457277	3.1325981	2.6555214
H	-0.4881637	3.2520211	0.5153214
C	0.9099783	1.6503031	3.7519624
H	2.3271793	0.6507891	2.4799874
C	-0.1502107	2.5503281	3.8223674
H	-1.4806687	3.8266501	2.7024054
H	1.2943843	1.1881291	4.6572774
H	-0.5966077	2.7919441	4.7832054
O	1.7346413	-1.3182169	0.3008834
Cu	-0.0775567	0.7107321	-1.5699806
C	-1.1057957	1.7707571	-2.9986316
H	-1.2774167	1.1092411	-3.8579856
C	-0.5438017	3.1193161	-3.4395426
H	0.3718063	2.9867971	-4.0268776
H	-1.2508227	3.6915061	-4.0630456
H	-0.2853597	3.7504441	-2.5810156
C	-2.2502787	1.8298761	-2.1646236

C	-3.2072887	1.8541021	-1.3899646
C	-4.2418437	1.7727071	-0.4296086
C	-5.1116497	0.6604741	-0.4058266
C	-4.4037907	2.7637301	0.5623184
C	-6.0832827	0.5416961	0.5787804
H	-4.9985847	-0.1111299	-1.1618256
C	-5.3832457	2.6350891	1.5401834
H	-3.7437527	3.6271971	0.5585634
C	-6.2277247	1.5240501	1.5603034
H	-6.7329657	-0.3304609	0.5816134
H	-5.4863467	3.4098291	2.2964674
H	-6.9884987	1.4254721	2.3298144
C	4.9067153	-4.1270569	-0.0404876
H	5.8198903	-3.9188799	0.5252844
H	4.6564443	-5.1769909	0.1392554
H	5.1200103	-3.9976219	-1.1071336
C	3.5285633	-3.4462239	1.9308094
H	3.2641933	-4.4933059	2.1176474
H	4.4362503	-3.2080769	2.4969904
H	2.7150903	-2.8148349	2.3042204

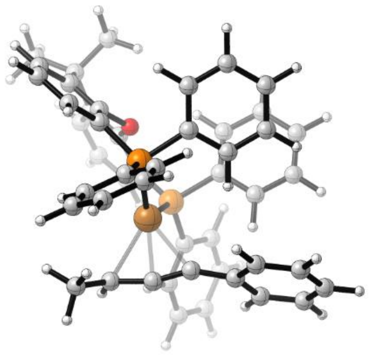

TS^{i}$_{xant}$

C	1.1271101	-4.0237744	-0.9817633
C	0.9899261	-2.7156734	-0.5086133
C	2.1559981	-2.0287814	-0.1619323
C	3.4347981	-2.5748994	-0.2609543
C	3.5325751	-3.8767134	-0.7536413
C	2.3908131	-4.5936334	-1.1080263
C	4.6039291	-1.7349494	0.2412137
C	4.3055951	-0.2823544	-0.1122343
C	2.9808421	0.1443236	-0.0355363
C	2.5738471	1.4578946	-0.2810833
C	3.5664231	2.3971526	-0.5742673
C	4.8992481	2.0043346	-0.6599953
C	5.2636841	0.6766476	-0.4431593
H	0.2461431	-4.5896834	-1.2679053
H	4.5065801	-4.3440954	-0.8575313
H	2.4880331	-5.6076864	-1.4851013
H	3.2950111	3.4325846	-0.7531893
H	5.6623781	2.7397436	-0.8981093
H	6.3084481	0.3931206	-0.5207283
P	-0.5896739	-1.7934624	-0.4367683
P	0.7721651	1.7891596	-0.2519353
C	-1.8127239	-2.9900954	-1.0821953
C	-2.2224149	-4.1066964	-0.3411783
C	-2.3676179	-2.7657754	-2.3452613
C	-3.1668349	-4.9850724	-0.8623763
H	-1.8048449	-4.2847404	0.6463587

C	-3.3158249	-3.6451974	-2.8651143
H	-2.0651549	-1.8928864	-2.9149543
C	-3.7156679	-4.7544814	-2.1243773
H	-3.4783649	-5.8492844	-0.2817283
H	-3.7456759	-3.4577004	-3.8453673
H	-4.4584199	-5.4384064	-2.5264323
C	-1.0241129	-1.7701954	1.3451947
C	-2.3323759	-1.3883784	1.6722867
C	-0.1253839	-2.0727934	2.3695277
C	-2.7373159	-1.3256984	2.9999167
H	-3.0379289	-1.1380134	0.8844127
C	-0.5294599	-1.9914934	3.7019307
H	0.8896871	-2.3812414	2.1376177
C	-1.8336909	-1.6232074	4.0205697
H	-3.7568539	-1.0309694	3.2352717
H	0.1799621	-2.2247764	4.4915867
H	-2.1452709	-1.5648764	5.0600537
C	0.6595031	3.5265026	-0.8178763
C	0.0837761	4.5316426	-0.0351233
C	1.0866111	3.8347266	-2.1170183
C	-0.0605739	5.8220106	-0.5429533
H	-0.2553859	4.3106166	0.9725617
C	0.9554951	5.1267566	-2.6152103
H	1.5279321	3.0598006	-2.7387883
C	0.3767271	6.1237346	-1.8298123
H	-0.5126859	6.5934136	0.0748837
H	1.2982061	5.3527996	-3.6214783

H	0.2661611	7.1310786	-2.2220543
C	0.4013781	1.9060296	1.5345437
C	-0.9266959	1.7356286	1.9379497
C	1.3800731	2.2012826	2.4892327
C	-1.2764099	1.8798726	3.2775787
H	-1.6841149	1.4880216	1.1984527
C	1.0305411	2.3236526	3.8313737
H	2.4147771	2.3347766	2.1847537
C	-0.2978369	2.1692326	4.2262257
H	-2.3126649	1.7519906	3.5775687
H	1.7968751	2.5448756	4.5696527
H	-0.5683779	2.2695416	5.2740877
O	1.9921281	-0.7459374	0.2946297
Cu	-0.5737999	0.3056396	-1.3155333
C	-1.2014619	0.6528926	-3.5458033
H	-1.2295739	-0.3253634	-4.0296493
C	-0.6311539	1.7705806	-4.3858463
H	0.4054531	1.5638036	-4.6798493
H	-1.2021539	1.9208076	-5.3157833
H	-0.6413219	2.7194006	-3.8411543
C	-2.1500489	0.8825726	-2.5735873
C	-2.7500269	1.0204886	-1.4725693
C	-3.8931939	1.1895646	-0.6403303
C	-4.8313299	0.1460546	-0.4786933
C	-4.0781869	2.3660716	0.1169677
C	-5.8925029	0.2743246	0.4096997
H	-4.7050459	-0.7690224	-1.0522573

C	-5.1497099	2.4874886	0.9933347
H	-3.3605039	3.1762086	0.0140407
C	-6.0617849	1.4428906	1.1541907
H	-6.5976129	-0.5466664	0.5181607
H	-5.2696079	3.4061246	1.5630277
H	-6.8923769	1.5386086	1.8481057
C	5.9377541	-2.2055164	-0.3298923
H	6.7600161	-1.6042164	0.0697377
H	6.1349371	-3.2417464	-0.0389993
H	5.9573281	-2.1404944	-1.4232163
C	4.6468801	-1.8502444	1.7799507
H	4.8192221	-2.8915214	2.0752897
H	5.4593431	-1.2333434	2.1805907
H	3.7074421	-1.5147114	2.2324057

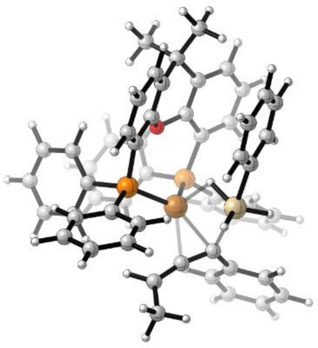

TS^4_{xant}

C	4.0181306	-0.4037661	-0.2894343
C	2.7443756	-0.2164101	0.2561867
C	2.3685186	1.0845609	0.5944457
C	3.1832826	2.1978389	0.3943707
C	4.4320976	1.9755029	-0.1852653

C	4.8497856	0.6876849	-0.5142093
C	2.6849606	3.5480119	0.8932507
C	1.1741146	3.5819009	0.7008547
C	0.4633216	2.3953989	0.8777987
C	-0.9275184	2.3083859	0.7924667
C	-1.6363344	3.4893789	0.5531007
C	-0.9570814	4.6887999	0.3637597
C	0.4339796	4.7319659	0.4267047
H	4.3484636	-1.4005771	-0.5616423
H	5.0958666	2.8122559	-0.3760993
H	5.8313246	0.5351619	-0.9535063
H	-2.7191594	3.4678539	0.4936567
H	-1.5165644	5.5984949	0.1656717
H	0.9426696	5.6778569	0.2718437
P	1.4997676	-1.5479971	0.4099007
P	-1.6950704	0.6483269	0.9140887
O	1.1256636	1.2282779	1.1533607
Cu	-0.5487404	-0.8831061	-0.3956083
C	-0.6664224	-3.8988661	-1.4200963
H	-0.2889604	-4.1417771	-0.4275423
C	-0.5815554	-4.9840551	-2.4560753
H	0.4572686	-5.3140731	-2.5912433
H	-1.1620834	-5.8660071	-2.1536593
H	-0.9605314	-4.6405451	-3.4237313
C	-1.1360314	-2.6889601	-1.6650653
C	-1.6733934	-1.5909111	-2.1891483
C	-3.1565754	-1.5020511	-2.1995313

C	-3.9355434	-1.8723181	-1.0915613
C	-3.8205374	-1.0995821	-3.3664693
C	-5.3235374	-1.8521031	-1.1489283
H	-3.4335424	-2.1671401	-0.1725753
C	-5.2127244	-1.0859391	-3.4281113
H	-3.2393394	-0.8182771	-4.2396573
C	-5.9709294	-1.4580041	-2.3205693
H	-5.9034514	-2.1345311	-0.2736553
H	-5.7057454	-0.7817191	-4.3483593
H	-7.0566624	-1.4371631	-2.3664093
C	3.3811946	4.7096519	0.1901597
H	3.0204066	5.6672759	0.5770187
H	4.4587846	4.6820759	0.3784597
H	3.2141776	4.6817039	-0.8917933
C	2.9708986	3.6298859	2.4078957
H	4.0495616	3.5676949	2.5920467
H	2.6010376	4.5800279	2.8101667
H	2.4814716	2.8142719	2.9514797
C	0.9405466	1.2771939	-3.4611843
C	2.2183506	0.9494099	-3.9313373
C	3.1990356	1.9207259	-4.1441413
C	2.9138636	3.2616219	-3.8935603
C	1.6462766	3.6189739	-3.4291203
C	0.6827876	2.6351069	-3.2158363
H	2.4595436	-0.0965561	-4.1292653
H	3.6725296	4.0237619	-4.0561683
H	1.4150326	4.6634569	-3.2284773

H	-0.2950644	2.9333929	-2.8324603
Si	-0.3727544	-0.0955221	-2.9730183
H	-1.5657894	0.5808079	-3.5969893
H	0.4088936	-1.2034791	-3.6461033
H	-0.1397354	0.3941549	-1.5073333
C	2.3797276	-2.9477951	-0.3709493
C	3.0613556	-3.9251911	0.3589787
C	2.4014916	-2.9970171	-1.7706693
C	3.7453096	-4.9439411	-0.3028703
H	3.0641566	-3.8952261	1.4443527
C	3.0977916	-4.0056081	-2.4285143
H	1.8713686	-2.2434921	-2.3460143
C	3.7656716	-4.9861961	-1.6946983
H	4.2687266	-5.7016531	0.2741747
H	3.1100806	-4.0301181	-3.5148693
H	4.3011436	-5.7804591	-2.2079433
C	1.4654726	-1.9914851	2.1809627
C	0.7179066	-3.1170951	2.5541857
C	2.1171466	-1.2447951	3.1644907
C	0.6502916	-3.5040331	3.8877097
H	0.1958586	-3.6977841	1.7960607
C	2.0234286	-1.6194131	4.5036897
H	2.7052036	-0.3739431	2.8899697
C	1.2982316	-2.7510011	4.8668107
H	0.0797646	-4.3865441	4.1647037
H	2.5279796	-1.0276321	5.2626897
H	1.2337796	-3.0449391	5.9109197

C	-1.7126784	0.2961609	2.7037747
C	-1.9838374	-1.0163631	3.1061857
C	-1.4853174	1.2802139	3.6708517
C	-2.0455584	-1.3363321	4.4591367
H	-2.1423594	-1.7888001	2.3566197
C	-1.5290384	0.9526939	5.0234977
H	-1.2744754	2.3020279	3.3675827
C	-1.8132384	-0.3529451	5.4190707
H	-2.2577284	-2.3574181	4.7630757
H	-1.3446544	1.7210019	5.7696157
H	-1.8479414	-0.6058571	6.4753587
C	-3.4381314	1.0338739	0.5213227
C	-3.7378114	1.4189949	-0.7930493
C	-4.4635904	0.9623399	1.4661597
C	-5.0399324	1.7507249	-1.1471423
H	-2.9476244	1.4623239	-1.5384753
C	-5.7733274	1.2728919	1.1010927
H	-4.2454594	0.6673669	2.4884167
C	-6.0623734	1.6722849	-0.2006293
H	-5.2597904	2.0512599	-2.1675263
H	-6.5661354	1.2098359	1.8418977
H	-7.0829764	1.9188059	-0.4809853
H	4.1854906	1.6328549	-4.5027683

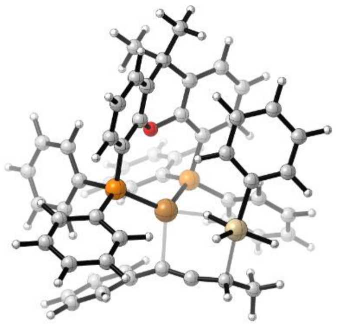

TS$^{6}_{xant}$

C	-0.5054615	-3.7601366	-1.5521246
C	-0.3951985	-2.6840096	-0.6677616
C	0.7158825	-2.6464466	0.1777014
C	1.6973225	-3.6362726	0.2017854
C	1.5670235	-4.6841266	-0.7103906
C	0.4793085	-4.7432786	-1.5808866
C	2.7827705	-3.5210916	1.2653274
C	3.1229235	-2.0416816	1.4023224
C	2.0807485	-1.1217056	1.2838974
C	2.2553475	0.2591064	1.4013544
C	3.5399485	0.7233694	1.7029354
C	4.5991675	-0.1685226	1.8311624
C	4.3947045	-1.5374016	1.6692184
H	-1.3545555	-3.8183736	-2.2263686
H	2.3154035	-5.4697016	-0.7408836
H	0.3948595	-5.5696146	-2.2807226
H	3.7173105	1.7884534	1.8114884
H	5.5944985	0.2058894	2.0522894
H	5.2373855	-2.2153926	1.7583194
P	-1.5795695	-1.2955196	-0.5764346

P	0.8020455	1.3444374	1.1186774
O	0.8093575	-1.5685076	1.0207884
Cu	-0.5691485	0.7531324	-0.6486316
C	-0.3124645	3.2614274	-3.2202776
H	-0.5924925	3.2201504	-4.2800846
C	0.3135955	4.5937384	-2.8304846
H	1.2376435	4.7596424	-3.3980356
H	-0.3564935	5.4410544	-3.0263986
H	0.5671495	4.6068774	-1.7650446
C	-1.3186335	2.8006854	-2.3731396
C	-2.0076835	2.2281174	-1.5068806
C	-3.2563575	2.1399114	-0.7888566
C	-4.3965365	1.5845884	-1.3954546
C	-3.3576605	2.6173364	0.5287574
C	-5.6041085	1.5297494	-0.7060526
H	-4.3256585	1.2015804	-2.4091326
C	-4.5676495	2.5557104	1.2118764
H	-2.4796455	3.0427714	1.0063784
C	-5.6976905	2.0142784	0.5990424
H	-6.4763335	1.1002074	-1.1920476
H	-4.6254255	2.9311814	2.2304234
H	-6.6420225	1.9670894	1.1344414
C	4.0070655	-4.3667326	0.9317704
H	4.7536685	-4.2924296	1.7282724
H	3.7316405	-5.4228446	0.8528664
H	4.4718355	-4.0569376	-0.0108026
C	2.1874625	-4.0050846	2.6046404

H	1.8939805	-5.0582826	2.5279474
H	2.9307545	-3.9058596	3.4038074
H	1.3043645	-3.4206146	2.8843874
C	2.7291235	0.5741224	-2.9124016
C	4.0172925	0.9639244	-3.3006316
C	5.1344585	0.1659334	-3.0450746
C	4.9780925	-1.0613996	-2.4048156
C	3.7043315	-1.4762296	-2.0138046
C	2.6039445	-0.6563366	-2.2515056
H	4.1516065	1.9183784	-3.8106896
H	6.1251435	0.5002754	-3.3464096
H	5.8426165	-1.6901496	-2.2047806
H	3.5718195	-2.4328356	-1.5152246
H	1.6186305	-0.9804196	-1.9120926
Si	1.1775345	1.6874314	-3.2566976
H	2.0110435	2.7448104	-3.9802716
H	0.4170085	0.8583374	-4.2550936
H	0.6121285	1.2969694	-1.8399226
C	-2.5542915	-1.6786366	0.9272574
C	-3.7104745	-0.9252266	1.1691474
C	-2.1564435	-2.6336866	1.8671194
C	-4.4539145	-1.1264016	2.3272544
H	-4.0340255	-0.1833686	0.4459384
C	-2.8949355	-2.8218576	3.0339684
H	-1.2744145	-3.2422326	1.6945174
C	-4.0438295	-2.0709066	3.2680804
H	-5.3516405	-0.5373686	2.4947894

H	-2.5706195	-3.5648886	3.7576584
H	-4.6189825	-2.2220706	4.1776074
C	-2.7312995	-1.6209636	-1.9533236
C	-3.8673935	-2.4278216	-1.8299256
C	-2.4483725	-1.0114416	-3.1819186
C	-4.7151455	-2.6103576	-2.9194306
H	-4.0945545	-2.9067126	-0.8816916
C	-3.2913615	-1.2060406	-4.2725936
H	-1.5721235	-0.3743906	-3.2781726
C	-4.4296855	-1.9997256	-4.1399246
H	-5.6000645	-3.2324576	-2.8149166
H	-3.0646855	-0.7271606	-5.2213686
H	-5.0954405	-2.1416496	-4.9870536
C	1.5591685	2.9826904	0.8426434
C	1.0902855	4.1294504	1.4914024
C	2.5462815	3.1133424	-0.1463336
C	1.6144035	5.3813714	1.1720334
H	0.3195555	4.0488784	2.2529734
C	3.0772625	4.3625864	-0.4498756
H	2.9106865	2.2350324	-0.6731106
C	2.6131685	5.5009884	0.2087444
H	1.2430755	6.2640594	1.6860104
H	3.8479785	4.4447804	-1.2116556
H	3.0243495	6.4773134	-0.0330016
C	0.0567475	1.4399794	2.7851284
C	-1.2908225	1.1037314	2.9323034
C	0.8040115	1.8053744	3.9124754

C	-1.8927195	1.1394474	4.1889844
H	-1.8690115	0.8080074	2.0624524
C	0.2016095	1.8435794	5.1650974
H	1.8558085	2.0600834	3.8085544
C	-1.1474875	1.5109744	5.3043344
H	-2.9409955	0.8720594	4.2900214
H	0.7846285	2.1298314	6.0364354
H	-1.6134425	1.5390714	6.2857924

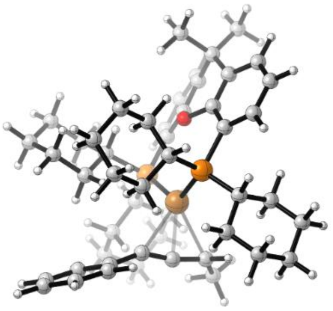

TS^{i}$_{xantcy}$

C	-3.4675736	3.0467313	-0.0617424
C	-2.5978856	1.9504483	0.0007336
C	-3.1804656	0.6947133	0.1950716
C	-4.5604666	0.4870953	0.2781676
C	-5.3880476	1.6022513	0.1648236
C	-4.8453676	2.8752233	0.0088616
C	-5.0552046	-0.9205027	0.5838396
C	-4.1142116	-1.9011297	-0.1019714
C	-2.7574976	-1.5775127	-0.1432624
C	-1.7667926	-2.4365157	-0.6262854
C	-2.1860046	-3.6904107	-1.0860354
C	-3.5349396	-4.0332557	-1.0911884

C	-4.4917056	-3.1454057	-0.6055944
H	-3.0719996	4.0494953	-0.1837774
H	-6.4655706	1.4852213	0.2179546
H	-5.5010486	3.7388723	-0.0570174
H	-1.4610756	-4.4100867	-1.4504614
H	-3.8415586	-5.0060267	-1.4652474
H	-5.5367416	-3.4380107	-0.6026844
P	-0.7622356	2.1091543	-0.1330584
P	-0.0166746	-1.8683897	-0.5301494
O	-2.3278746	-0.3672547	0.3340746
Cu	0.4477314	0.3367213	-1.0219574
C	1.5753014	0.9757503	-3.0671424
H	1.1732094	1.9677373	-3.2722344
C	1.7647234	0.0945873	-4.2780724
H	0.8112134	-0.1757317	-4.7502544
H	2.3687524	0.5953133	-5.0510404
H	2.2850184	-0.8325767	-4.0191444
C	2.3432144	0.8557283	-1.9388734
C	2.8399274	0.6519083	-0.7960054
C	3.9413374	0.7411613	0.1063116
C	4.3052964	1.9641263	0.7126126
C	4.6774424	-0.4085807	0.4692096
C	5.3369484	2.0249903	1.6420366
H	3.7667824	2.8669423	0.4401226
C	5.7149994	-0.3352887	1.3904626
H	4.4194584	-1.3618087	0.0161166
C	6.0500934	0.8780143	1.9938006

H	5.5919294	2.9828123	2.0904676
H	6.2651884	-1.2386737	1.6444346
H	6.8556594	0.9296193	2.7211626
C	-6.5081676	-1.1258407	0.1654206
H	-6.8411366	-2.1369797	0.4182136
H	-7.1655706	-0.4360657	0.7032316
H	-6.6463146	-0.9723037	-0.9103984
C	-4.9370826	-1.1421357	2.1073716
H	-5.5762736	-0.4304037	2.6424336
H	-5.2515336	-2.1596647	2.3664666
H	-3.9063766	-1.0036587	2.4516196
C	0.8645794	-3.1889807	-1.4911424
C	0.4771574	-3.0962637	-2.9720914
C	2.3838864	-3.0716997	-1.3293104
H	0.5582124	-4.1691767	-1.0994214
C	1.2124224	-4.1399227	-3.8103554
H	0.7271834	-2.0927547	-3.3335854
H	-0.6045656	-3.2094627	-3.1011384
C	3.1133504	-4.1197507	-2.1674294
H	2.7022514	-2.0651587	-1.6319314
H	2.6630004	-3.1868257	-0.2765494
C	2.7246464	-4.0307967	-3.6393584
H	0.9365244	-4.0245917	-4.8662154
H	0.8843444	-5.1451127	-3.5056884
H	4.1972744	-3.9957177	-2.0484124
H	2.8653814	-5.1217767	-1.7864404
H	3.2323094	-4.8134847	-4.2172724

H	3.0654254	-3.0664847	-4.0435294
C	0.2740994	-2.2385597	1.2826266
C	0.2495854	-3.7271017	1.6306676
C	1.5202684	-1.5589937	1.8501736
H	-0.5980696	-1.7629537	1.7583306
C	0.3428264	-3.9319467	3.1427446
H	1.1018104	-4.2280957	1.1505736
H	-0.6613986	-4.1977067	1.2411276
C	1.6131664	-1.7716177	3.3593766
H	2.4215904	-1.9646147	1.3756446
H	1.5022944	-0.4905637	1.6112566
C	1.5836994	-3.2547287	3.7184756
H	0.3486264	-5.0049597	3.3733746
H	-0.5550126	-3.5099047	3.6181506
H	2.5269834	-1.2994167	3.7421666
H	0.7659024	-1.2661177	3.8465446
H	1.6192554	-3.3855077	4.8074696
H	2.4820074	-3.7421017	3.3110386
C	-0.6337506	3.6973523	-1.1081834
C	0.8120474	4.1946493	-1.2311894
C	-1.2669636	3.4838393	-2.4913834
H	-1.1982096	4.4746063	-0.5739044
C	0.9141384	5.4197383	-2.1396214
H	1.4521084	3.3917373	-1.6179404
H	1.2004774	4.4594603	-0.2436584
C	-1.1451356	4.7210643	-3.3771154
H	-0.7705036	2.6352953	-2.9804684

H	-2.3211566	3.2058283	-2.3870284
C	0.3069764	5.1639813	-3.5136444
H	1.9666944	5.7161883	-2.2304804
H	0.3879974	6.2624643	-1.6666804
H	-1.5804546	4.5099643	-4.3620044
H	-1.7339646	5.5411603	-2.9395714
H	0.3779574	6.0643623	-4.1368844
H	0.8806824	4.3765813	-4.0247254
C	-0.3495256	2.5798263	1.6338436
C	-1.0064286	1.6184033	2.6322596
C	1.1621744	2.6073093	1.8714566
H	-0.7647636	3.5876683	1.7913606
C	-0.6667226	1.9926513	4.0742466
H	-0.6558376	0.5969063	2.4263466
H	-2.0939166	1.6137283	2.5031876
C	1.5045194	2.9906723	3.3090256
H	1.5705734	1.6128393	1.6512006
H	1.6556554	3.2957323	1.1828166
C	0.8394254	2.0507473	4.3072916
H	-1.1338296	1.2724853	4.7584106
H	-1.1070646	2.9749043	4.3013336
H	2.5942974	2.9786383	3.4373376
H	1.1718964	4.0220503	3.4993256
H	1.0550484	2.3656043	5.3362546
H	1.2637154	1.0440093	4.1874916

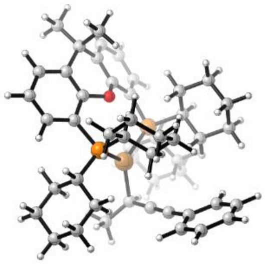

Cu^{p}$_{xantcy}$

C	4.1525555	2.2644532	-0.0189908
C	3.0568845	1.3941822	-0.0913118
C	3.3378275	0.0574012	-0.3950358
C	4.6302075	-0.4342728	-0.6019908
C	5.6901125	0.4622842	-0.4849348
C	5.4518765	1.8045532	-0.2020738
C	4.7754555	-1.8905248	-1.0230288
C	3.7189115	-2.6857748	-0.2699838
C	2.4719175	-2.0865648	-0.0874338
C	1.3777375	-2.7334268	0.4941302
C	1.5699125	-4.0591718	0.9015232
C	2.8088215	-4.6771598	0.7600162
C	3.8766445	-3.9951118	0.1819192
H	4.0018535	3.3166532	0.1951482
H	6.7095585	0.1197052	-0.6301708
H	6.2845455	2.4983582	-0.1276508
H	0.7525945	-4.6193808	1.3424152
H	2.9401095	-5.7022698	1.0949162
H	4.8327715	-4.4961368	0.0698912
P	1.3058745	1.9171542	0.1892452
P	-0.2067315	-1.8045688	0.6180402
O	2.2707105	-0.7958118	-0.5063938

Cu	-0.1358705	0.4142052	1.2794952
C	-1.3867775	1.3087052	2.6874402
H	-0.9386755	2.2927692	2.8589742
C	-1.7007175	0.6248722	4.0133432
H	-0.7858165	0.4531422	4.5944502
H	-2.3801415	1.2195242	4.6471792
H	-2.1783705	-0.3483928	3.8633262
C	-2.4468955	1.3726102	1.7546072
C	-3.3477805	1.3696412	0.9124252
C	-4.2835585	1.2606862	-0.1400538
C	-4.3163425	2.2026392	-1.1922518
C	-5.1961385	0.1827682	-0.1936508
C	-5.2019625	2.0564922	-2.2528968
H	-3.6311715	3.0451122	-1.1657918
C	-6.0782625	0.0483522	-1.2581468
H	-5.1921455	-0.5502218	0.6088132
C	-6.0873955	0.9782442	-2.2996608
H	-5.2001915	2.7934592	-3.0528688
H	-6.7651205	-0.7947088	-1.2768238
H	-6.7772505	0.8670552	-3.1316638
C	6.1828905	-2.4276458	-0.7841478
H	6.2576635	-3.4664068	-1.1202788
H	6.9159555	-1.8566168	-1.3619088
H	6.4617395	-2.3829148	0.2743352
C	4.4556095	-1.9830978	-2.5308098
H	5.1770885	-1.3912018	-3.1056568
H	4.5123465	-3.0254348	-2.8648698

H	3.4507175	-1.6066258	-2.7499808
C	-1.2422565	-2.9382688	1.6608372
C	-0.7008195	-2.9459918	3.0968352
C	-2.7159375	-2.5175948	1.6445862
H	-1.1797745	-3.9548348	1.2479082
C	-1.5437775	-3.8364018	4.0072592
H	-0.7097005	-1.9176218	3.4804352
H	0.3443105	-3.2749608	3.1157212
C	-3.5557515	-3.4000588	2.5670502
H	-2.7986765	-1.4666208	1.9520512
H	-3.1160715	-2.5783748	0.6276002
C	-3.0106605	-3.4173058	3.9912832
H	-1.1456895	-3.8014638	5.0292452
H	-1.4603045	-4.8805088	3.6701682
H	-4.5966505	-3.0520738	2.5588542
H	-3.5628025	-4.4265958	2.1709162
H	-3.6077605	-4.0902588	4.6197252
H	-3.1034835	-2.4119258	4.4264312
C	-0.7890695	-2.0019178	-1.1461738
C	-1.1223125	-3.4367778	-1.5548378
C	-1.9283485	-1.0456768	-1.4972748
H	0.0946675	-1.6805428	-1.7195838
C	-1.4638875	-3.5074218	-3.0437058
H	-1.9845095	-3.7919178	-0.9736528
H	-0.2832005	-4.1052938	-1.3259048
C	-2.2624325	-1.1205688	-2.9843498
H	-2.8255565	-1.2936128	-0.9194778

H	-1.6562245	-0.0246498	-1.2116108
C	-2.5979515	-2.5497738	-3.4023338
H	-1.7299635	-4.5368018	-3.3160668
H	-0.5700135	-3.2454108	-3.6290688
H	-3.0973695	-0.4455978	-3.2105908
H	-1.3985605	-0.7668008	-3.5668888
H	-2.8078155	-2.5946488	-4.4785468
H	-3.5164695	-2.8672688	-2.8865668
C	1.5701535	3.4623582	1.2003952
C	0.3325045	4.3652742	1.2544732
C	2.0379875	3.0447312	2.6011092
H	2.3620615	4.0561292	0.7254322
C	0.5408325	5.5428392	2.2069332
H	-0.5479505	3.7907852	1.5679142
H	0.1177725	4.7542372	0.2536752
C	2.2274645	4.2439632	3.5261152
H	1.2903685	2.3666642	3.0361002
H	2.9720335	2.4740662	2.5277042
C	0.9573445	5.0842182	3.5998242
H	-0.3795555	6.1382652	2.2545712
H	1.3225125	6.2009122	1.7987262
H	2.5206315	3.8959092	4.5245882
H	3.0531175	4.8677022	3.1521822
H	1.1062115	5.9496602	4.2577832
H	0.1494245	4.4824682	4.0422642
C	0.8304295	2.5527752	-1.5061748
C	1.2582395	1.5945252	-2.6233778

C	-0.6780325	2.8087592	-1.6066118
H	1.3690065	3.5043612	-1.6396558
C	0.8854775	2.1499902	-3.9973358
H	0.7635085	0.6242992	-2.4715208
H	2.3374945	1.4119772	-2.5822968
C	-1.0477035	3.3865442	-2.9711958
H	-1.2055885	1.8581402	-1.4621008
H	-1.0270005	3.4722832	-0.8113218
C	-0.6039955	2.4634522	-4.1008058
H	1.1817775	1.4349962	-4.7755938
H	1.4626285	3.0690832	-4.1770818
H	-2.1309005	3.5538332	-3.0193768
H	-0.5686455	4.3698682	-3.0899638
H	-0.8320085	2.9119902	-5.0760918
H	-1.1763595	1.5268992	-4.0407358

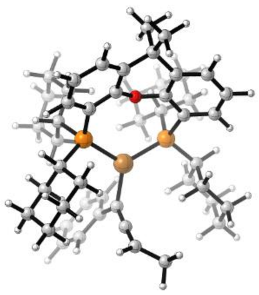

Cu$^{a}_{xantcy}$

C	-1.8724611	4.1110676	-0.5976687
C	-1.5975571	2.7649066	-0.3223507
C	-2.6716441	1.8726266	-0.4119067
C	-3.9543331	2.2479376	-0.8230827
C	-4.1691231	3.5901626	-1.1279267

C	-3.1394891	4.5193366	-0.9982257
C	-5.0354371	1.1747986	-0.8432207
C	-4.3759711	-0.1221214	-1.2927777
C	-3.0892651	-0.3830464	-0.8213837
C	-2.4064641	-1.5817334	-1.0486977
C	-3.0662231	-2.5530944	-1.8103437
C	-4.3350781	-2.3096634	-2.3289657
C	-4.9861791	-1.1052774	-2.0709867
H	-1.0863011	4.8540406	-0.5146777
H	-5.1492141	3.9215806	-1.4554837
H	-3.3252551	5.5670696	-1.2173317
H	-2.5884561	-3.5064704	-2.0108627
H	-4.8260981	-3.0705884	-2.9291107
H	-5.9824221	-0.9414744	-2.4691977
P	0.0945119	2.1387866	0.0715803
P	-0.7460281	-1.7714884	-0.2823367
O	-2.4360981	0.5646886	-0.0739077
Cu	0.7344299	-0.0304854	-0.4398967
C	3.1754009	-0.1956604	-3.5407697
H	3.3138149	0.7793806	-4.0173357
C	3.2462689	-1.3940424	-4.4490897
H	2.4334669	-1.3934244	-5.1903857
H	4.1876679	-1.4147794	-5.0162117
H	3.1794159	-2.3268864	-3.8802777
C	2.9568199	-0.2356664	-2.2360627
C	2.6614779	-0.2660534	-0.9621937
C	3.7171089	-0.3043714	0.0711883

C	4.9565749	0.3402056	-0.0962887
C	3.4820969	-0.9411914	1.3016063
C	5.9095269	0.3499896	0.9177923
H	5.1594969	0.8452576	-1.0384457
C	4.4388649	-0.9466344	2.3127523
H	2.5238839	-1.4310874	1.4567473
C	5.6579859	-0.2944024	2.1297193
H	6.8549619	0.8652266	0.7623993
H	4.2266379	-1.4528464	3.2519973
H	6.4009809	-0.2833594	2.9230653
C	-6.2167931	1.5597066	-1.7280297
H	-6.9822111	0.7778246	-1.7061167
H	-6.6902641	2.4751476	-1.3601617
H	-5.9114231	1.7188896	-2.7680017
C	-5.5385631	0.9751046	0.6026813
H	-5.9934501	1.9006436	0.9738553
H	-6.2904571	0.1782986	0.6338513
H	-4.7195081	0.7013396	1.2762303
C	-0.1233361	-3.3449514	-1.0380947
C	0.2295739	-3.0736444	-2.5073647
C	1.1064429	-3.8745404	-0.2926637
H	-0.9094661	-4.1112774	-0.9845617
C	0.7938219	-4.3184984	-3.1876827
H	0.9783909	-2.2700614	-2.5394727
H	-0.6465421	-2.7093134	-3.0567887
C	1.6643299	-5.1242564	-0.9713387
H	1.8784929	-3.0930704	-0.2734757

H	0.8548929	-4.1054394	0.7477723
C	2.0046489	-4.8610264	-2.4343127
H	1.0629189	-4.0806664	-4.2246737
H	0.0145479	-5.0940644	-3.2320577
H	2.5522399	-5.4717124	-0.4281657
H	0.9185989	-5.9309924	-0.9096267
H	2.3748869	-5.7767454	-2.9125277
H	2.8189359	-4.1236504	-2.4828967
C	-1.2875701	-2.1476294	1.4644383
C	-2.0116411	-3.4833284	1.6339373
C	-0.1641151	-1.9855884	2.4903463
H	-2.0131151	-1.3419644	1.6572473
C	-2.5464691	-3.6293084	3.0587273
H	-1.3155411	-4.3073444	1.4247613
H	-2.8327311	-3.5657084	0.9111693
C	-0.7080271	-2.1323474	3.9100753
H	0.6156899	-2.7395734	2.3230793
H	0.3123179	-1.0067084	2.3639033
C	-1.4342981	-3.4629994	4.0914623
H	-3.0350771	-4.6049344	3.1759043
H	-3.3185921	-2.8649444	3.2309033
H	0.1105929	-2.0392744	4.6350523
H	-1.4071491	-1.3071974	4.1129073
H	-1.8432761	-3.5385774	5.1068543
H	-0.7118421	-4.2850294	3.9788483
C	1.1437679	3.3628476	-0.8601867
C	2.6083549	3.3386926	-0.4154617

C	1.0147579	3.0444946	-2.3558717
H	0.7643779	4.3768206	-0.6771127
C	3.4713869	4.2492866	-1.2879857
H	2.9895829	2.3118976	-0.4708027
H	2.6890929	3.6605046	0.6285843
C	1.8855499	3.9618526	-3.2104157
H	1.3236659	2.0020326	-2.5191517
H	-0.0344701	3.1194786	-2.6683897
C	3.3438049	3.9018116	-2.7671557
H	4.5175159	4.1759776	-0.9642817
H	3.1648059	5.2950756	-1.1352987
H	1.7918209	3.6793976	-4.2667097
H	1.5213349	4.9966176	-3.1246697
H	3.9580749	4.5794326	-3.3736967
H	3.7296239	2.8854336	-2.9331597
C	0.2841709	2.5615786	1.8795533
C	-0.9790871	2.1884526	2.6644143
C	1.4959089	1.8262756	2.4649103
H	0.4404879	3.6485846	1.9559383
C	-0.8114711	2.4650266	4.1574403
H	-1.1914461	1.1195626	2.5121133
H	-1.8440561	2.7396606	2.2790913
C	1.6668019	2.1086956	3.9549573
H	1.3489869	0.7482156	2.3167673
H	2.4135259	2.0792826	1.9272423
C	0.4061149	1.7487096	4.7337723
H	-1.7220651	2.1643836	4.6913343

H	-0.6993031	3.5487016	4.3094673
H	2.5296059	1.5468846	4.3350393
H	1.8941329	3.1753256	4.0992993
H	0.5261309	1.9952136	5.7963173
H	0.2463519	0.6619886	4.6766723

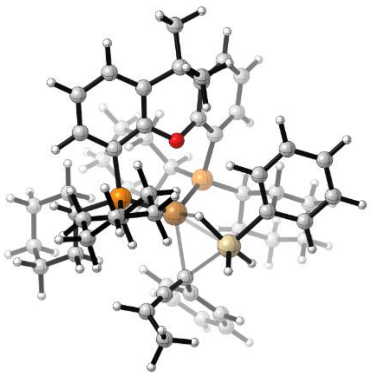

TS[4] $_{xantcy}$

C	-4.3314184	0.2176078	-0.7932149
C	-2.9892314	0.3801258	-0.4280659
C	-2.4745244	1.6781968	-0.4529459
C	-3.2394514	2.8064708	-0.7583839
C	-4.5661504	2.5993588	-1.1314329
C	-5.1051674	1.3134228	-1.1610879
C	-2.5823934	4.1687278	-0.5662779
C	-1.1420144	4.0404658	-1.0462129
C	-0.4809734	2.8345368	-0.7986969
C	0.8213566	2.5670828	-1.2273419
C	1.4996836	3.6035698	-1.8810129
C	0.8851366	4.8298578	-2.1067909
C	-0.4333034	5.0403568	-1.7089879
H	-4.7728554	-0.7742372	-0.7976339

H	-5.1943134	3.4453958	-1.3915529
H	-6.1410384	1.1695628	-1.4549179
H	2.5118846	3.4497328	-2.2411439
H	1.4292526	5.6208248	-2.6150169
H	-0.9086134	5.9926498	-1.9210039
P	-1.9358854	-0.9835642	0.2032151
P	1.4940486	0.8500618	-1.1550719
O	-1.1486654	1.8317708	-0.1426009
Cu	0.3226756	-0.4641052	0.3468441
C	-0.1576794	-3.5554302	3.1743581
H	-1.0858024	-3.9832512	2.7936931
C	0.1510096	-3.7893572	4.6285101
H	-0.6448064	-3.3859942	5.2696571
H	0.2302196	-4.8613932	4.8543151
H	1.0923636	-3.3114532	4.9171771
C	0.6275856	-2.8814252	2.3570941
C	1.4437356	-2.1244662	1.6736301
C	2.5841656	-2.7102112	0.9378631
C	2.4267696	-3.2083752	-0.3646649
C	3.8663906	-2.7524412	1.5086011
C	3.5077466	-3.7186442	-1.0756479
H	1.4418786	-3.1777842	-0.8233869
C	4.9521116	-3.2531622	0.7912221
H	4.0071236	-2.3982472	2.5254481
C	4.7815996	-3.7332872	-0.5057189
H	3.3563696	-4.1004522	-2.0829129
H	5.9361296	-3.2725492	1.2538311

H	5.6289626	-4.1236122	-1.0632819
C	-2.5725474	4.4819038	0.9451551
H	-2.0763214	5.4423428	1.1255161
H	-3.5994684	4.5432018	1.3232691
H	-2.0449254	3.7089408	1.5131971
C	-3.3342144	5.2786768	-1.2937749
H	-4.3600354	5.3538058	-0.9200489
H	-2.8596444	6.2475628	-1.1110109
H	-3.3691424	5.1061558	-2.3751029
C	3.3300816	1.1859088	-1.0743009
C	3.6752186	2.0470118	0.1433441
C	4.1609756	-0.1006732	-1.0668059
H	3.5806896	1.7398948	-1.9910629
C	5.1700176	2.3419428	0.2270421
H	3.3749696	1.5092488	1.0475181
H	3.1066446	2.9827318	0.1361621
C	5.6560706	0.1996918	-0.9739459
H	3.8626246	-0.7185992	-0.2121259
H	3.9716936	-0.6886312	-1.9686719
C	5.9830256	1.0520458	0.2463561
H	5.3692876	2.9392608	1.1257641
H	5.4745216	2.9534668	-0.6358299
H	6.2102386	-0.7467052	-0.9444139
H	5.9783066	0.7294718	-1.8831979
H	7.0574976	1.2721898	0.2853731
H	5.7375616	0.4851988	1.1565821
C	1.2347736	0.3532738	-2.9450809

C	1.4522216	-1.1487002	-3.1499749
C	-0.1541314	0.7480918	-3.4593409
H	1.9901526	0.9105968	-3.5207649
C	1.2817756	-1.5462392	-4.6151789
H	0.7248606	-1.6914012	-2.5315069
H	2.4392826	-1.4585082	-2.7985649
C	-0.3336044	0.3535228	-4.9248299
H	-0.9202634	0.2545818	-2.8445719
H	-0.3106714	1.8261128	-3.3549919
C	-0.0821374	-1.1327342	-5.1577199
H	1.4244466	-2.6295092	-4.7198519
H	2.0720786	-1.0647532	-5.2098529
H	-1.3432854	0.6277448	-5.2560259
H	0.3687076	0.9393628	-5.5362029
H	-0.1545064	-1.3675522	-6.2272179
H	-0.8643384	-1.7160642	-4.6532079
C	-2.6124944	-1.1915192	1.9346651
C	-2.6798614	0.1599698	2.6549961
C	-3.9536584	-1.9155762	2.0602711
H	-1.8416764	-1.7945732	2.4339301
C	-3.0225054	-0.0336172	4.1297631
H	-3.4517974	0.7838208	2.1829681
H	-1.7296854	0.6958748	2.5557841
C	-4.3214064	-2.1040162	3.5339301
H	-4.7366274	-1.3285622	1.5592481
H	-3.9217054	-2.8949242	1.5702311
C	-4.3439074	-0.7791262	4.2904741

H	-3.0682274	0.9412798	4.6315501
H	-2.2153074	-0.6032192	4.6134751
H	-5.2942274	-2.6062692	3.6111171
H	-3.5824724	-2.7728672	3.9994711
H	-4.5622064	-0.9518292	5.3519741
H	-5.1581974	-0.1527712	3.8966711
C	-2.5409834	-2.4598452	-0.7400539
C	-2.3320094	-2.2713892	-2.2428599
C	-1.8240354	-3.7207052	-0.2487339
H	-3.6166584	-2.5753282	-0.5497759
C	-2.7715534	-3.5054942	-3.0263969
H	-1.2667904	-2.0893862	-2.4295269
H	-2.8697014	-1.3843352	-2.5977139
C	-2.2592944	-4.9531292	-1.0382479
H	-0.7399664	-3.5812742	-0.3574159
H	-2.0134714	-3.8757042	0.8191261
C	-2.0490714	-4.7567032	-2.5364099
H	-2.5852984	-3.3476572	-4.0963989
H	-3.8568684	-3.6416602	-2.9104479
H	-1.7052674	-5.8310852	-0.6833899
H	-3.3240844	-5.1494392	-0.8438809
H	-2.3923854	-5.6394862	-3.0900679
H	-0.9723364	-4.6532032	-2.7390639
C	1.7589596	1.7853528	2.9835551
C	0.8061306	2.7306758	2.5851831
C	0.9751796	4.0946498	2.8297651
C	2.1137116	4.5437668	3.4955861

C	3.0767586	3.6219398	3.9106981
C	2.8977966	2.2650468	3.6480511
H	-0.0790994	2.3935818	2.0470141
H	0.2242726	4.8061148	2.4922081
H	2.2554996	5.6051708	3.6851631
H	3.9699926	3.9659668	4.4282141
H	3.6765906	1.5618838	3.9476171
Si	1.5629726	-0.1052902	2.5850311
H	3.0446616	-0.3113452	2.3280131
H	1.2010356	-0.6497502	3.9330951
H	0.1588726	0.1957738	1.9409881

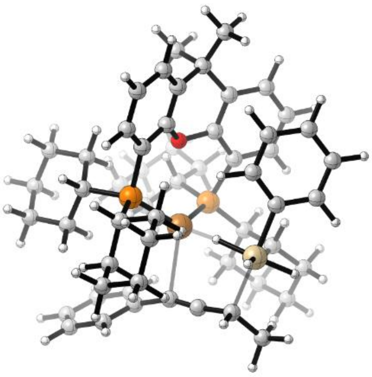

TS^{6}_{xantcy}

C	1.2731755	-4.2475948	-0.0463194
C	0.9657025	-2.9070868	0.2163156
C	1.9548395	-2.1375858	0.8366276
C	3.2307795	-2.6224328	1.1386996
C	3.5078165	-3.9500268	0.8166386
C	2.5316925	-4.7621718	0.2431666
C	4.1939545	-1.6936648	1.8682966
C	3.9402665	-0.2783858	1.3661856

C	2.6228585	0.0865532	1.0972436
C	2.2275355	1.3828852	0.7563336
C	3.2378355	2.3414052	0.6299486
C	4.5709725	1.9977282	0.8369176
C	4.9185895	0.7035792	1.2144576
H	0.5291425	-4.8946308	-0.4999584
H	4.4868025	-4.3665258	1.0307026
H	2.7558415	-5.8010688	0.0186516
H	2.9938115	3.3634752	0.3610646
H	5.3444675	2.7510232	0.7175146
H	5.9617745	0.4629252	1.3917786
P	-0.6648785	-2.1616628	-0.2122214
P	0.4205825	1.7048142	0.6215926
O	1.6242665	-0.8524278	1.1741966
Cu	-0.5301915	0.0864302	-0.7107384
C	-1.3298135	1.3723902	-4.0963804
H	-1.6126205	0.6341122	-4.8601194
C	-1.4011905	2.7811782	-4.6785514
H	-0.7394355	2.8602702	-5.5488574
H	-2.4148615	3.0538292	-5.0012804
H	-1.0693505	3.5286962	-3.9488634
C	-2.1190645	1.1685882	-2.9405504
C	-2.6766875	0.9335342	-1.8659344
C	-3.7537405	0.7231652	-0.9412564
C	-4.7837335	-0.1844738	-1.2504684
C	-3.8206715	1.4190422	0.2756436
C	-5.8311795	-0.3946418	-0.3611914

H	-4.7527835	-0.7186048	-2.1951784
C	-4.8730265	1.2080572	1.1602816
H	-3.0386215	2.1279832	0.5183576
C	-5.8805285	0.2960122	0.8504806
H	-6.6169025	-1.1007178	-0.6174864
H	-4.9022765	1.7574492	2.0980086
H	-6.7007945	0.1285932	1.5431986
C	5.6495425	-2.1135548	1.6871316
H	6.3126355	-1.4442468	2.2436256
H	5.8146475	-3.1198918	2.0833106
H	5.9477895	-2.1004698	0.6329796
C	3.8439235	-1.7342108	3.3712936
H	3.9852975	-2.7474418	3.7648496
H	4.4919325	-1.0480768	3.9286426
H	2.8029695	-1.4408278	3.5459466
C	0.3715055	3.4681802	0.0570656
C	0.7893955	3.5359702	-1.4161874
C	-1.0088205	4.1089302	0.2242466
H	1.0829865	4.0417262	0.6675096
C	0.8094185	4.9746522	-1.9247814
H	0.0604775	2.9571552	-1.9995784
H	1.7636865	3.0615352	-1.5776644
C	-0.9876295	5.5587502	-0.2580794
H	-1.7409485	3.5418292	-0.3665354
H	-1.3335355	4.0717682	1.2693986
C	-0.5390285	5.6578292	-1.7130994
H	1.0803645	4.9821442	-2.9881184

H	1.5926705	5.5355732	-1.3935894
H	-1.9831775	6.0033972	-0.1346734
H	-0.3008835	6.1368842	0.3779256
H	-0.4894145	6.7080932	-2.0271124
H	-1.2916695	5.1715622	-2.3505134
C	0.0410285	1.6864142	2.4533946
C	0.6928195	2.8256612	3.2384436
C	-1.4443665	1.5638612	2.7909396
H	0.5144075	0.7440432	2.7682996
C	0.4974495	2.6214242	4.7406936
H	0.2382445	3.7814312	2.9427026
H	1.7617635	2.8914352	3.0026606
C	-1.6336035	1.3564262	4.2924726
H	-1.9762705	2.4744722	2.4879386
H	-1.8901305	0.7343642	2.2304376
C	-0.9801975	2.4780602	5.0959266
H	0.9448165	3.4580822	5.2921846
H	1.0356395	1.7132712	5.0501326
H	-2.7036895	1.2889272	4.5269376
H	-1.1843215	0.3943262	4.5805866
H	-1.0977135	2.2948862	6.1713946
H	-1.4964705	3.4246972	4.8774416
C	-1.1881005	-3.2125948	-1.6641324
C	-2.5465705	-2.7632028	-2.2149994
C	-0.1349925	-3.1199838	-2.7772354
H	-1.2779505	-4.2556758	-1.3262924
C	-2.9497375	-3.5650468	-3.4513314

H	-2.4854625	-1.6975758	-2.4738394
H	-3.3264275	-2.8607898	-1.4558614
C	-0.5357855	-3.9262098	-4.0097294
H	-0.0236635	-2.0654708	-3.0614474
H	0.8448285	-3.4522128	-2.4205354
C	-1.8920365	-3.4816988	-4.5451734
H	-3.9167065	-3.2004038	-3.8212844
H	-3.0968935	-4.6178568	-3.1675064
H	0.2370695	-3.8171728	-4.7809044
H	-0.5775675	-4.9946378	-3.7498324
H	-2.1843295	-4.0904128	-5.4101844
H	-1.8159415	-2.4421768	-4.8968324
C	-1.6701525	-2.7468248	1.2565876
C	-0.9573825	-2.3771228	2.5649506
C	-3.0913135	-2.1776858	1.2598426
H	-1.7170875	-3.8442498	1.1790556
C	-1.7556115	-2.8159818	3.7911086
H	-0.8114845	-1.2886168	2.5952146
H	0.0383165	-2.8310568	2.5988566
C	-3.8825905	-2.6331418	2.4840496
H	-3.0357915	-1.0807678	1.2531206
H	-3.6311775	-2.4660518	0.3556346
C	-3.1724315	-2.2534248	3.7770086
H	-1.2251895	-2.5051178	4.7004196
H	-1.8032345	-3.9147608	3.8138676
H	-4.8861465	-2.1913468	2.4495926
H	-4.0137205	-3.7248478	2.4467296

H	-3.7357925	-2.6123068	4.6476436
H	-3.1301325	-1.1574418	3.8546536
C	2.5191565	0.4993832	-3.1306744
C	3.5679885	1.3265782	-3.5523804
C	4.8956725	1.0699072	-3.2044724
C	5.2058825	-0.0435138	-2.4262574
C	4.1800025	-0.8824018	-1.9897814
C	2.8587095	-0.5982568	-2.3274334
H	3.3393935	2.1959362	-4.1706244
H	5.6877025	1.7369432	-3.5396274
H	6.2376035	-0.2524008	-2.1525304
H	4.4094895	-1.7503078	-1.3756494
H	2.0679785	-1.2493198	-1.9535394
Si	0.6868855	0.7980022	-3.7306534
H	1.0843605	2.1055672	-4.4191644
H	0.6221555	-0.3174848	-4.7459614
H	0.1643145	0.2461812	-2.3359864

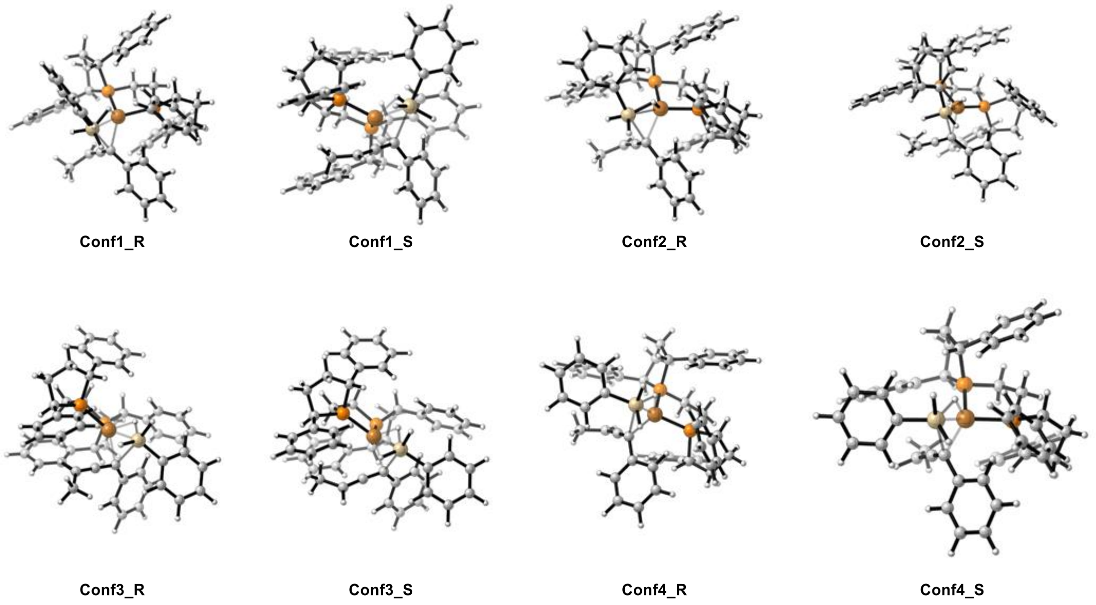

Supplementary Fig 4. Conformers conf1-conf4.

Supplementary Tab 4. Thermodynamic data of TS conformers. Energy unit: Hartree.

**Geometry**	**Gcr**	**E_{sol}(dlpno-ccsd(t))**	**G_{sol}[dlpno-ccsd(t)]**	**G_{rel}/kcal•mol^{-1}**
**Conf1_*R***	0.833882	-4544.319564	-4543.485682	0
**Conf1_*S***	0.835919	-4544.318848	-4543.482929	1.73
**Conf2_*R***	0.835293	-4544.320169	-4543.484876	0.51
**Conf2_*S***	0.837075	-4544.31994	-4543.482865	1.77
**Conf3_*R***	0.83558	-4544.320169	-4543.484589	0.69
**Conf3_*S***	0.835742	-4544.318351	-4543.482609	1.93
**Conf4_*R***	0.834988	-4544.318402	-4543.483414	1.42
**Conf4_*S***	0.837434	-4544.317549	-4543.480115	3.49

Supplementary Tab 5. Boltzmann distribution of the metathesis TS conformers responsible for enantiocontrol.

Temperature:	223.15	$Q_{(Relat)}$: 1.619989		
**Entry**	**G_{rel}(kcal/mol)**	**$Q_{i(Relat)}$**	**Percent/%**	**structure**
1	0	1	61.73	**Conf1_*R***
2	1.72	0.020625	1.27	**Conf1_*S***
3	0.51	0.31637	19.53	**Conf2_*R***
4	1.77	0.018424	1.14	**Conf2_*S***
5	0.69	0.210763	13.01	**Conf3_*R***
6	1.93	0.012841	0.79	**Conf3_*S***

7	1.42	0.040587	2.51	**Conf4_*R***
8	3.49	0.00038	0.02	**Conf4_*S***

Cartesian coordinates of the metathesis conformers **conf1-conf4**.

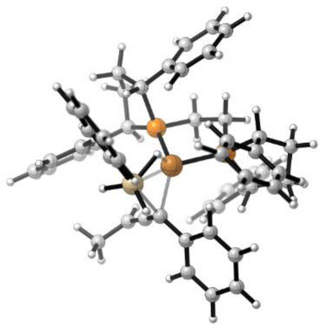

Conf1_*R*

C	-2.44271	-2.33783	0.16894
C	-3.81908	-2.39598	0.86811
C	-3.81619	-1.52793	2.13206
C	-2.98320	-0.26257	1.88971
H	-2.57967	-2.01326	-0.86730
H	-4.56681	-2.02165	0.16163
H	-4.10037	-3.42702	1.10579
H	-4.83483	-1.28516	2.45754
H	-3.34803	-2.08172	2.95433
H	-2.59931	0.12788	2.84014
P	-1.49914	-0.92102	0.96407
C	-0.52208	-1.65032	2.36246
H	-0.55408	-0.89418	3.15382
H	-1.00498	-2.55116	2.75500
C	0.92897	-1.96302	1.99969
H	0.97014	-2.85787	1.36539
H	1.49737	-2.18157	2.91003
C	2.39480	0.66285	2.18572
C	3.43266	-1.41764	0.74441
C	3.85611	0.27706	2.50793
H	2.38374	1.59182	1.60502
C	4.11666	-1.17396	2.10608
H	3.28859	-2.49154	0.57795
H	4.51696	0.93115	1.92877
H	4.08688	0.44655	3.56471
H	5.19044	-1.38712	2.04523
H	3.69040	-1.85891	2.84852
P	1.75404	-0.64475	0.98401
C	-3.63884	0.88004	1.15032
C	-4.92699	0.81116	0.61583
C	-2.91967	2.07421	0.99303
C	-5.47629	1.89890	-0.06552
H	-5.51801	-0.09271	0.72700

C	-3.46160	3.15807	0.31189
H	-1.91602	2.14608	1.40421
C	-4.74554	3.07201	-0.22799
H	-6.48105	1.82160	-0.47346
H	-2.87772	4.06799	0.19905
H	-5.17236	3.91442	-0.76554
C	-1.58657	-3.57350	0.14831
C	-1.60909	-4.52748	1.17092
C	-0.66307	-3.73518	-0.89367
C	-0.72328	-5.60405	1.15983
H	-2.31961	-4.43066	1.98838
C	0.22406	-4.80763	-0.90528
H	-0.63907	-3.00338	-1.69809
C	0.20134	-5.74549	0.12681
H	-0.75643	-6.33419	1.96460
H	0.93091	-4.91127	-1.72488
H	0.89163	-6.58490	0.12112
C	1.44847	0.84639	3.33779
C	1.55008	0.10220	4.51790
C	0.37883	1.74056	3.20174
C	0.59560	0.23336	5.52492
H	2.37419	-0.59460	4.64863
C	-0.57707	1.87221	4.20552
H	0.29819	2.33360	2.29271
C	-0.47584	1.11180	5.37032
H	0.68863	-0.35625	6.43338
H	-1.40023	2.57068	4.07771
H	-1.22026	1.21107	6.15573
C	4.23856	-0.85018	-0.40058
C	4.06072	0.44697	-0.89040
C	5.24641	-1.63946	-0.96748
C	4.86973	0.94648	-1.90887
H	3.26817	1.07659	-0.49435
C	6.06020	-1.14423	-1.98280
H	5.39346	-2.65421	-0.60310
C	5.87668	0.15483	-2.45578
H	4.70460	1.95564	-2.27738
H	6.83589	-1.77565	-2.40857
H	6.50810	0.54318	-3.25044
Cu	0.05884	0.04287	-0.36618
C	1.82834	-1.56306	-2.94359
H	2.15081	-2.32709	-2.23466
C	2.66922	-1.38039	-4.17720
H	3.70397	-1.12966	-3.91122

H	2.70397	-2.30036	-4.77680
H	2.27669	-0.57730	-4.80916
C	0.75065	-0.85466	-2.67003
C	-0.27221	-0.05909	-2.44039
C	-1.61579	-0.36379	-2.97488
C	-1.79144	-1.20405	-4.08872
C	-2.76329	0.19890	-2.39341
C	-3.05856	-1.49170	-4.58278
H	-0.91295	-1.63876	-4.56086
C	-4.03402	-0.08853	-2.88802
H	-2.65481	0.85327	-1.53254
C	-4.18966	-0.93641	-3.98258
H	-3.16520	-2.15190	-5.44046
H	-4.90426	0.35515	-2.41212
H	-5.18110	-1.15853	-4.36892
C	-0.33411	4.97751	-1.43982
C	0.60811	3.95378	-1.27010
C	1.79891	4.28232	-0.60358
C	2.03793	5.56693	-0.11838
C	1.07955	6.56585	-0.29533
C	-0.10836	6.26933	-0.96139
H	-1.26820	4.76176	-1.95970
H	2.56219	3.51524	-0.45961
H	2.97034	5.79346	0.39468
H	1.26045	7.56996	0.08063
H	-0.85692	7.04505	-1.10895
Si	0.24243	2.13233	-1.82019
H	-0.94462	2.42381	-2.68442
H	1.60208	1.80476	-2.35352
H	-0.08401	1.76868	-0.33529

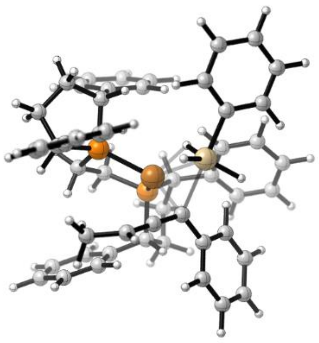

Conf1_*S*

C	-2.58559	-2.13823	0.04086
C	-3.97349	-2.00341	0.70219
C	-3.84504	-1.28007	2.04398
C	-2.88243	-0.09812	1.87976

H	-2.64808	-1.81500	-1.00258
H	-4.60926	-1.41920	0.02863
H	-4.45004	-2.98221	0.82187
H	-4.82002	-0.95989	2.43016
H	-3.41947	-1.96293	2.78930
H	-2.43502	0.16727	2.84531
P	-1.49346	-0.84164	0.86436
C	-0.53920	-1.70043	2.20505
H	-0.50776	-0.98292	3.03116
H	-1.07416	-2.58547	2.56429
C	0.88315	-2.08857	1.80363
H	0.85718	-2.95177	1.12572
H	1.44824	-2.38874	2.69271
C	2.47908	0.47163	2.06864
C	3.42517	-1.62077	0.58694
C	3.93378	0.03491	2.35069
H	2.48072	1.41854	1.51799
C	4.12173	-1.42503	1.94789
H	3.23631	-2.68517	0.40421
H	4.60283	0.65935	1.74848
H	4.19754	0.20084	3.40041
H	5.18301	-1.69294	1.88472
H	3.66077	-2.08859	2.68922
P	1.77955	-0.78069	0.83759
C	-3.43786	1.16773	1.26800
C	-4.76101	1.29789	0.84031
C	-2.59081	2.27789	1.13944
C	-5.21977	2.49650	0.29084
H	-5.45094	0.46448	0.93053
C	-3.04247	3.47265	0.59093
H	-1.55872	2.19566	1.46685
C	-4.36356	3.58528	0.15643
H	-6.25358	2.57243	-0.03686
H	-2.35893	4.31269	0.49811
H	-4.72135	4.51478	-0.27811
C	-1.92729	-3.48866	0.06266
C	-2.12645	-4.40457	1.10163
C	-1.03453	-3.82731	-0.96254
C	-1.44748	-5.62142	1.11896
H	-2.81863	-4.16662	1.90586
C	-0.35401	-5.04202	-0.94639
H	-0.87653	-3.12600	-1.77949
C	-0.55558	-5.94380	0.09779
H	-1.61735	-6.31991	1.93444

H	0.33197	-5.28523	-1.75371
H	-0.02723	-6.89335	0.11153
C	1.57484	0.64027	3.25533
C	1.66125	-0.18385	4.38302
C	0.56561	1.60899	3.21129
C	0.74537	-0.05842	5.42538
H	2.44265	-0.93735	4.44345
C	-0.35332	1.73470	4.25004
H	0.50602	2.26714	2.34687
C	-0.27170	0.89337	5.35914
H	0.82405	-0.71067	6.29141
H	-1.13235	2.49068	4.19192
H	-0.98759	0.98678	6.17132
C	4.25405	-1.07001	-0.55148
C	4.01478	0.17105	-1.14520
C	5.33514	-1.83022	-1.01660
C	4.82576	0.64114	-2.17732
H	3.17160	0.77674	-0.82353
C	6.14680	-1.36639	-2.04707
H	5.53310	-2.80163	-0.56769
C	5.89377	-0.12564	-2.63315
H	4.61028	1.60631	-2.62860
H	6.97530	-1.97663	-2.39770
H	6.52326	0.23671	-3.44154
Cu	0.11621	0.04715	-0.47384
C	1.52579	-1.47680	-3.36138
C	0.55877	-0.71044	-2.89952
C	-0.36841	0.15260	-2.53431
C	-1.77852	-0.04278	-2.94584
C	-2.11941	-0.86659	-4.03251
C	-2.82006	0.60898	-2.26766
C	-3.44459	-1.05304	-4.40903
H	-1.32441	-1.37144	-4.57702
C	-4.14898	0.42490	-2.64544
H	-2.58599	1.25057	-1.42304
C	-4.46941	-0.40853	-3.71479
H	-3.67981	-1.70323	-5.24848
H	-4.93440	0.93768	-2.09730
H	-5.50612	-0.55118	-4.00870
C	0.06453	5.05038	-1.31335
C	0.90790	3.93347	-1.24140
C	2.15060	4.11048	-0.61289
C	2.53319	5.33700	-0.07225
C	1.67027	6.43108	-0.15242

C	0.43348	6.28631	-0.77853
H	-0.90668	4.95365	-1.80038
H	2.84139	3.26776	-0.54409
H	3.50329	5.44455	0.40888
H	1.96310	7.39053	0.26722
H	-0.24173	7.13660	-0.85083
Si	0.34532	2.18139	-1.87355
H	-0.85755	2.66112	-2.63316
H	1.65126	1.80132	-2.50997
H	0.09309	1.78003	-0.37891
C	2.09156	-2.69332	-2.68925
H	3.18633	-2.65244	-2.65948
H	1.72160	-2.78688	-1.66423
H	1.81748	-3.60782	-3.23406
H	1.93270	-1.22529	-4.34412

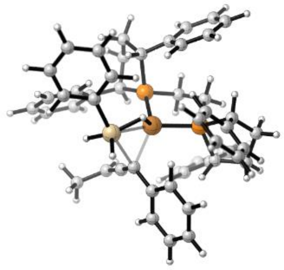

Conf2_*R*

C	-3.07846	-1.43205	0.31190
C	-4.46727	-0.91089	0.74205
C	-4.31683	0.20612	1.77881
C	-3.14298	1.11011	1.38282
H	-3.01140	-1.42586	-0.78017
H	-4.96860	-0.52270	-0.15018
H	-5.09119	-1.72382	1.12785
H	-5.24555	0.77664	1.89861
H	-4.08841	-0.23172	2.75796
H	-2.73689	1.61645	2.26705
P	-1.83972	-0.12483	0.85818
C	-1.18810	-0.65564	2.51108
H	-1.07719	0.26910	3.08625
H	-1.91476	-1.28171	3.03875
C	0.15210	-1.38156	2.42407
H	0.00445	-2.38682	2.00839
H	0.57590	-1.50060	3.42699
C	2.24071	0.78351	2.22861
C	2.78575	-1.78342	1.42772
C	3.53063	0.15281	2.79939
H	2.50185	1.52876	1.46893

C	3.42605	-1.37114	2.77023
H	2.35994	-2.79014	1.50646
H	4.37197	0.46653	2.17243
H	3.73486	0.51868	3.81099
H	4.40722	-1.84440	2.89366
H	2.78877	-1.72313	3.59017
P	1.36837	-0.58602	1.26521
C	-3.39493	2.15985	0.32583
C	-4.61536	2.30582	-0.33687
C	-2.35669	3.04540	0.00242
C	-4.79074	3.30090	-1.29992
H	-5.44621	1.64623	-0.10675
C	-2.52441	4.03232	-0.96201
H	-1.40182	2.94886	0.51283
C	-3.74629	4.16185	-1.62337
H	-5.75057	3.39580	-1.80150
H	-1.70009	4.70042	-1.19880
H	-3.88220	4.93052	-2.37934
C	-2.65474	-2.79404	0.78705
C	-3.06835	-3.32675	2.01266
C	-1.75642	-3.53518	0.00815
C	-2.58773	-4.55911	2.45239
H	-3.77107	-2.77551	2.63306
C	-1.27353	-4.76517	0.44594
H	-1.43093	-3.13489	-0.94916
C	-1.68425	-5.28098	1.67475
H	-2.92181	-4.95534	3.40798
H	-0.57925	-5.32283	-0.17791
H	-1.31058	-6.24149	2.01948
C	1.29780	1.43928	3.19732
C	1.13192	0.98283	4.50921
C	0.49554	2.49762	2.75279
C	0.17523	1.55576	5.34508
H	1.74621	0.16603	4.87977
C	-0.46226	3.07099	3.58483
H	0.62559	2.86590	1.73769
C	-0.63119	2.59584	4.88510
H	0.05797	1.18473	6.36007
H	-1.07590	3.88967	3.21717
H	-1.37839	3.03901	5.53786
C	3.77365	-1.76786	0.28771
C	4.10462	-0.60801	-0.41988
C	4.43528	-2.95722	-0.04139
C	5.07261	-0.63460	-1.42138

H	3.59633	0.32995	-0.21031
C	5.40407	-2.98765	-1.04059
H	4.18494	-3.86899	0.49723
C	5.72876	-1.82222	-1.73410
H	5.30786	0.28014	-1.95841
H	5.90386	-3.92305	-1.27950
H	6.48267	-1.84190	-2.51662
Cu	0.00876	0.08711	-0.42052
C	1.29263	-2.88140	-2.21069
H	1.29889	-3.49755	-1.30950
C	2.22823	-3.27974	-3.31956
H	3.26891	-3.27244	-2.97186
H	2.01297	-4.29567	-3.67785
H	2.14927	-2.59377	-4.16876
C	0.48115	-1.84612	-2.26804
C	-0.21915	-0.73788	-2.36364
C	-1.57134	-0.71807	-2.96318
C	-2.07417	-1.81357	-3.68791
C	-2.39686	0.40866	-2.82490
C	-3.35267	-1.79218	-4.23262
H	-1.44536	-2.69243	-3.81321
C	-3.67691	0.43319	-3.37534
H	-2.03447	1.26723	-2.26661
C	-4.16390	-0.66690	-4.07768
H	-3.71871	-2.65678	-4.78149
H	-4.29420	1.31813	-3.24951
H	-5.16331	-0.64767	-4.50463
C	3.59061	2.66577	-2.51263
C	2.33592	2.59491	-1.88888
C	2.02827	3.59522	-0.95696
C	2.92238	4.62365	-0.65810
C	4.16112	4.67189	-1.29602
C	4.49389	3.68978	-2.22914
H	3.87151	1.89562	-3.23157
H	1.06476	3.56526	-0.44622
H	2.65680	5.38393	0.07348
H	4.86536	5.46722	-1.06422
H	5.45990	3.72004	-2.72865
Si	1.11747	1.14458	-2.29043
H	2.19772	0.16969	-2.67506
H	0.20075	1.64537	-1.06004
H	0.35300	1.62936	-3.48185

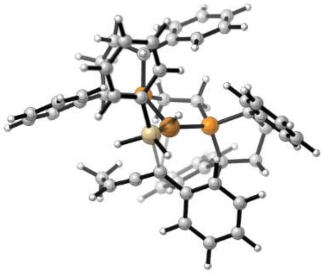

Conf2_*S*

C	-3.21834	-1.13511	-0.02115
C	-4.51231	-0.48131	0.50886
C	-4.21615	0.29756	1.79129
C	-2.91717	1.08726	1.59758
H	-3.12475	-0.93941	-1.09325
H	-4.88147	0.20303	-0.26164
H	-5.29290	-1.23237	0.67002
H	-5.05022	0.95054	2.07452
H	-4.06745	-0.40412	2.62111
H	-2.44795	1.29875	2.56607
P	-1.80137	-0.16340	0.75681
C	-1.20867	-1.13557	2.22255
H	-1.02674	-0.39467	3.00719
H	-1.98847	-1.81093	2.58895
C	0.06872	-1.92783	1.94625
H	-0.15705	-2.78162	1.29355
H	0.46507	-2.33110	2.88436
C	2.30145	0.08418	2.26931
C	2.70941	-2.25470	0.90424
C	3.55111	-0.73265	2.66696
H	2.60765	0.97142	1.70429
C	3.34501	-2.20190	2.30833
H	2.23371	-3.22778	0.73791
H	4.40839	-0.34457	2.10647
H	3.78101	-0.60662	3.73003
H	4.28856	-2.75978	2.32924
H	2.66683	-2.67754	3.02690
P	1.36187	-0.97264	1.01340
C	-3.01208	2.39042	0.83703
C	-4.20255	2.87604	0.29095
C	-1.85466	3.16749	0.68913
C	-4.23215	4.09254	-0.39261
H	-5.12397	2.31168	0.39458
C	-1.87726	4.37620	0.00291
H	-0.92139	2.80839	1.11282

C	-3.07077	4.84354	-0.54797
H	-5.17128	4.44862	-0.80863
H	-0.96134	4.95216	-0.10372
H	-3.09408	5.78615	-1.08804
C	-3.06234	-2.61517	0.18881
C	-3.60778	-3.27702	1.29504
C	-2.31507	-3.36137	-0.73112
C	-3.40874	-4.64387	1.47830
H	-4.19720	-2.72130	2.02041
C	-2.11494	-4.72759	-0.54977
H	-1.89099	-2.86011	-1.59871
C	-2.65925	-5.37470	0.55848
H	-3.84328	-5.13860	2.34326
H	-1.53563	-5.28767	-1.27920
H	-2.50635	-6.44123	0.70047
C	1.40642	0.54381	3.38435
C	1.19783	-0.21935	4.53861
C	0.70419	1.74655	3.24447
C	0.29082	0.19718	5.51077
H	1.73998	-1.15177	4.67591
C	-0.20466	2.16477	4.21283
H	0.87702	2.35554	2.36019
C	-0.42148	1.38487	5.34825
H	0.13786	-0.41115	6.39854
H	-0.74148	3.10081	4.08017
H	-1.13069	1.70600	6.10628
C	3.72764	-2.01971	-0.18567
C	4.06079	-0.74871	-0.66287
C	4.41859	-3.12323	-0.70207
C	5.05619	-0.58447	-1.62443
H	3.53913	0.13315	-0.29862
C	5.40805	-2.96427	-1.66784
H	4.17275	-4.11916	-0.33892
C	5.73231	-1.69006	-2.13205
H	5.29367	0.41517	-1.97756
H	5.92529	-3.83672	-2.05899
H	6.50408	-1.56151	-2.88634
Cu	0.07008	0.13662	-0.48521
C	1.01395	-2.41333	-3.05400
C	0.31848	-1.32870	-2.78529
C	-0.26972	-0.17572	-2.55644
C	-1.63838	0.10881	-3.04542
C	-2.29773	-0.75287	-3.93993
C	-2.32390	1.25656	-2.61832

C	-3.59337	-0.49193	-4.37007
H	-1.77897	-1.64303	-4.28904
C	-3.62065	1.52279	-3.05412
H	-1.84057	1.93746	-1.92389
C	-4.26491	0.64860	-3.92670
H	-4.08259	-1.18113	-5.05447
H	-4.12719	2.41758	-2.70349
H	-5.27786	0.85480	-4.26269
C	3.79509	2.88206	-2.12637
C	2.58257	2.71387	-1.44034
C	2.41723	3.43509	-0.25046
C	3.40751	4.28808	0.23844
C	4.60043	4.44003	-0.46638
C	4.79208	3.73569	-1.65546
H	3.96777	2.32459	-3.04757
H	1.49051	3.32073	0.31360
H	3.25162	4.83024	1.16884
H	5.37879	5.09917	-0.08962
H	5.72180	3.84787	-2.20924
Si	1.22826	1.50050	-2.11147
H	2.21192	0.55355	-2.75017
H	0.39590	1.78009	-0.76034
H	0.49438	2.31549	-3.12976
C	1.08232	-3.64378	-2.19518
H	0.59635	-4.50004	-2.68359
H	2.12479	-3.92886	-2.01046
H	0.59257	-3.48117	-1.23084
H	1.58020	-2.43192	-3.98854

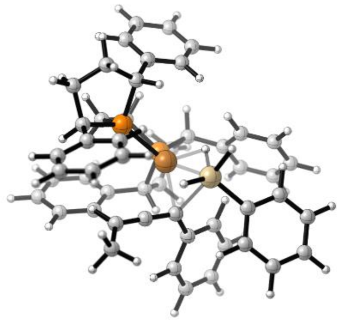

Conf3_R

C	-3.07846	-1.43205	0.31190
C	-4.46727	-0.91089	0.74205
C	-4.31683	0.20612	1.77881
C	-3.14298	1.11011	1.38282
H	-3.01140	-1.42586	-0.78017
H	-4.96860	-0.52270	-0.15018
H	-5.09119	-1.72382	1.12785

H	-5.24555	0.77664	1.89861
H	-4.08841	-0.23172	2.75796
H	-2.73689	1.61645	2.26705
P	-1.83972	-0.12483	0.85818
C	-1.18810	-0.65564	2.51108
H	-1.07719	0.26910	3.08625
H	-1.91476	-1.28171	3.03875
C	0.15210	-1.38156	2.42407
H	0.00445	-2.38682	2.00839
H	0.57590	-1.50060	3.42699
C	2.24071	0.78351	2.22861
C	2.78575	-1.78342	1.42772
C	3.53063	0.15281	2.79939
H	2.50185	1.52876	1.46893
C	3.42605	-1.37114	2.77023
H	2.35994	-2.79014	1.50646
H	4.37197	0.46653	2.17243
H	3.73486	0.51868	3.81099
H	4.40722	-1.84440	2.89366
H	2.78877	-1.72313	3.59017
P	1.36837	-0.58602	1.26521
C	-3.39493	2.15985	0.32583
C	-4.61536	2.30582	-0.33687
C	-2.35669	3.04540	0.00242
C	-4.79074	3.30090	-1.29992
H	-5.44621	1.64623	-0.10675
C	-2.52441	4.03232	-0.96201
H	-1.40182	2.94886	0.51283
C	-3.74629	4.16185	-1.62337
H	-5.75057	3.39580	-1.80150
H	-1.70009	4.70042	-1.19880
H	-3.88220	4.93052	-2.37934
C	-2.65474	-2.79404	0.78705
C	-3.06835	-3.32675	2.01266
C	-1.75642	-3.53518	0.00815
C	-2.58773	-4.55911	2.45239
H	-3.77107	-2.77551	2.63306
C	-1.27353	-4.76517	0.44594
H	-1.43093	-3.13489	-0.94916
C	-1.68425	-5.28098	1.67475
H	-2.92181	-4.95534	3.40798
H	-0.57925	-5.32283	-0.17791
H	-1.31058	-6.24149	2.01948
C	1.29780	1.43928	3.19732

C	1.13192	0.98283	4.50921
C	0.49554	2.49762	2.75279
C	0.17523	1.55576	5.34508
H	1.74621	0.16603	4.87977
C	-0.46226	3.07099	3.58483
H	0.62559	2.86590	1.73769
C	-0.63119	2.59584	4.88510
H	0.05797	1.18473	6.36007
H	-1.07590	3.88967	3.21717
H	-1.37839	3.03901	5.53786
C	3.77365	-1.76786	0.28771
C	4.10462	-0.60801	-0.41988
C	4.43528	-2.95722	-0.04139
C	5.07261	-0.63460	-1.42138
H	3.59633	0.32995	-0.21031
C	5.40407	-2.98765	-1.04059
H	4.18494	-3.86899	0.49723
C	5.72876	-1.82222	-1.73410
H	5.30786	0.28014	-1.95841
H	5.90386	-3.92305	-1.27950
H	6.48267	-1.84190	-2.51662
Cu	0.00876	0.08711	-0.42052
C	1.29263	-2.88140	-2.21069
H	1.29889	-3.49755	-1.30950
C	2.22823	-3.27974	-3.31956
H	3.26891	-3.27244	-2.97186
H	2.01297	-4.29567	-3.67785
H	2.14927	-2.59377	-4.16876
C	0.48115	-1.84612	-2.26804
C	-0.21915	-0.73788	-2.36364
C	-1.57134	-0.71807	-2.96318
C	-2.07417	-1.81357	-3.68791
C	-2.39686	0.40866	-2.82490
C	-3.35267	-1.79218	-4.23262
H	-1.44536	-2.69243	-3.81321
C	-3.67691	0.43319	-3.37534
H	-2.03447	1.26723	-2.26661
C	-4.16390	-0.66690	-4.07768
H	-3.71871	-2.65678	-4.78149
H	-4.29420	1.31813	-3.24951
H	-5.16331	-0.64767	-4.50463
C	3.59061	2.66577	-2.51263
C	2.33592	2.59491	-1.88888
C	2.02827	3.59522	-0.95696

C	2.92238	4.62365	-0.65810
C	4.16112	4.67189	-1.29602
C	4.49389	3.68978	-2.22914
H	3.87151	1.89562	-3.23157
H	1.06476	3.56526	-0.44622
H	2.65680	5.38393	0.07348
H	4.86536	5.46722	-1.06422
H	5.45990	3.72004	-2.72865
Si	1.11747	1.14458	-2.29043
H	2.19772	0.16969	-2.67506
H	0.20075	1.64537	-1.06004
H	0.35300	1.62936	-3.48185

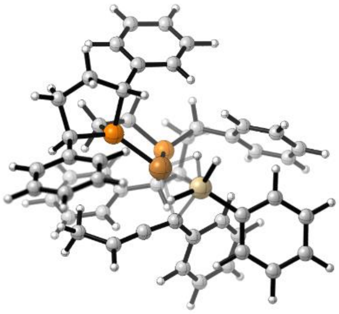

Conf3_*S*

C	0.96533	-1.99226	2.10020
C	1.94436	-3.18214	2.01640
C	1.67358	-3.99376	0.74894
C	1.44412	-3.02637	-0.41800
H	1.52205	-1.07951	2.33037
H	2.96064	-2.77618	1.98335
H	1.87942	-3.80855	2.91246
H	2.48370	-4.70137	0.53643
H	0.76237	-4.58948	0.88078
H	0.84337	-3.50758	-1.19914
P	0.32981	-1.72442	0.34485
C	-1.30314	-2.60005	0.22891
H	-1.31498	-3.04846	-0.76926
H	-1.36187	-3.41892	0.95291
C	-2.51197	-1.68210	0.39845
H	-2.61080	-1.38648	1.45120
H	-3.42525	-2.22119	0.12407
C	-2.86308	-0.39092	-2.31392
C	-4.02697	0.68279	-0.08108
C	-4.36196	-0.03428	-2.42363
H	-2.27177	0.33144	-2.88715
C	-4.99648	-0.04181	-1.03595

H	-4.25153	0.42203	0.95969
H	-4.44615	0.97203	-2.84821
H	-4.87900	-0.71545	-3.10755
H	-5.97788	0.44699	-1.03774
H	-5.14429	-1.07162	-0.68933
P	-2.37568	-0.07042	-0.51436
C	2.67020	-2.44153	-1.08058
C	3.97386	-2.72829	-0.67014
C	2.49790	-1.59063	-2.18162
C	5.07070	-2.17031	-1.32908
H	4.15044	-3.39113	0.17117
C	3.58736	-1.03401	-2.84133
H	1.49126	-1.35803	-2.51707
C	4.88417	-1.31552	-2.41084
H	6.07551	-2.40545	-0.98708
H	3.42331	-0.37713	-3.69132
H	5.73851	-0.87445	-2.91716
C	-0.17089	-2.09378	3.07815
C	-0.75604	-3.31651	3.42584
C	-0.70443	-0.92013	3.62649
C	-1.84776	-3.36418	4.29065
H	-0.35653	-4.24218	3.01835
C	-1.79540	-0.96558	4.49053
H	-0.25370	0.03634	3.36926
C	-2.37478	-2.18928	4.82346
H	-2.28713	-4.32449	4.54861
H	-2.19121	-0.04278	4.90667
H	-3.22520	-2.22738	5.49904
C	-2.45706	-1.76610	-2.76113
C	-3.28113	-2.88334	-2.58461
C	-1.18680	-1.95600	-3.31764
C	-2.83491	-4.15685	-2.93140
H	-4.27544	-2.76106	-2.16260
C	-0.73632	-3.22777	-3.66217
H	-0.54731	-1.09020	-3.47586
C	-1.55809	-4.33643	-3.46231
H	-3.48742	-5.01341	-2.78265
H	0.25641	-3.35130	-4.08763
H	-1.21091	-5.33109	-3.72863
C	-4.10697	2.18361	-0.23565
C	-3.23348	2.91791	-1.04084
C	-5.13275	2.86922	0.42797
C	-3.37316	4.29848	-1.17611
H	-2.41642	2.41956	-1.55625

C	-5.27657	4.24685	0.29611
H	-5.82056	2.31180	1.06090
C	-4.39364	4.96831	-0.50780
H	-2.67339	4.84793	-1.80044
H	-6.07541	4.75934	0.82612
H	-4.50012	6.04513	-0.60870
Cu	-0.17434	0.42739	-0.21041
C	-0.99762	2.98329	2.17819
C	-0.02985	2.37436	1.52464
C	0.98262	1.89620	0.82873
C	2.18027	1.37193	1.53346
C	2.42503	1.66915	2.88471
C	3.11983	0.57745	0.85980
C	3.54852	1.17855	3.54146
H	1.71389	2.29310	3.42168
C	4.24945	0.09111	1.51323
H	2.96103	0.33839	-0.18715
C	4.46882	0.38369	2.85812
H	3.70798	1.41993	4.58988
H	4.96058	-0.51841	0.96343
H	5.35098	0.00301	3.36650
C	3.22402	3.77767	-0.27242
C	2.69834	2.99479	-1.30913
C	3.55489	2.63744	-2.35911
C	4.89863	3.00771	-2.35616
C	5.40190	3.78433	-1.31411
C	4.55705	4.18175	-0.27841
H	2.58473	4.06456	0.55926
H	3.16598	2.05702	-3.19362
H	5.55009	2.69750	-3.16994
H	6.44728	4.08315	-1.31099
H	4.94099	4.79677	0.53206
Si	0.89105	2.43355	-1.36499
H	0.63604	0.91039	-1.67993
H	-0.19815	3.38170	-0.96020
H	0.73186	2.69044	-2.90099
C	-2.25264	2.34897	2.70128
H	-2.26328	2.34641	3.80027
H	-3.13941	2.90250	2.37192
H	-2.34547	1.31423	2.35981
H	-0.86064	4.04640	2.39166

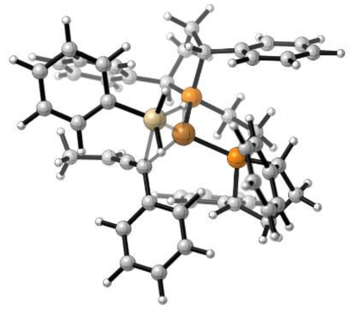

Conf4_*R*

C	-2.69698	0.27990	1.94500
C	-4.23245	0.42037	1.85981
C	-4.62839	1.22363	0.61605
C	-3.70503	0.85186	-0.55129
H	-2.43649	-0.77941	2.03543
H	-4.65605	-0.58736	1.80045
H	-4.63934	0.87990	2.76667
H	-5.68391	1.07475	0.35849
H	-4.50330	2.29406	0.81622
H	-3.65356	1.67589	-1.27374
P	-2.02142	0.77386	0.26149
C	-1.63334	2.58380	0.36357
H	-1.83522	2.96917	-0.64145
H	-2.31523	3.09487	1.05122
C	-0.18939	2.88214	0.75511
H	-0.04832	2.70054	1.82759
H	0.03150	3.93891	0.57138
C	1.43698	2.53876	-1.75260
C	2.61040	2.45389	0.72682
C	2.67452	3.44113	-1.55198
H	1.70580	1.68164	-2.38012
C	2.85783	3.75450	-0.06804
H	2.37200	2.68765	1.77089
H	3.55535	2.89912	-1.91290
H	2.59802	4.35660	-2.14781
H	3.85953	4.14937	0.13866
H	2.13571	4.51417	0.25327
P	1.06159	1.78173	-0.06364
C	-4.01799	-0.41483	-1.31197
C	-5.05921	-1.27926	-0.96656
C	-3.22509	-0.74151	-2.42192
C	-5.29251	-2.44388	-1.69992
H	-5.70099	-1.05170	-0.12094
C	-3.45469	-1.90078	-3.15392
H	-2.41209	-0.07702	-2.70547

C	-4.48872	-2.76379	-2.78980
H	-6.10716	-3.10322	-1.41113
H	-2.82122	-2.13349	-4.00591
H	-4.66698	-3.67486	-3.35445
C	-1.96293	1.02273	3.02618
C	-2.40339	2.24985	3.53249
C	-0.73971	0.51321	3.48269
C	-1.63415	2.95596	4.45623
H	-3.35226	2.66386	3.20001
C	0.03134	1.21778	4.40280
H	-0.38783	-0.44235	3.09996
C	-0.41089	2.44777	4.88971
H	-1.99317	3.90899	4.83657
H	0.97708	0.80214	4.74184
H	0.18778	3.00081	5.60861
C	0.20571	3.16608	-2.34334
C	-0.13035	4.50542	-2.11914
C	-0.68114	2.37029	-3.08047
C	-1.32998	5.02778	-2.59905
H	0.54475	5.14508	-1.55599
C	-1.88097	2.88924	-3.55983
H	-0.42637	1.32942	-3.26928
C	-2.21414	4.22100	-3.31362
H	-1.57515	6.06997	-2.41098
H	-2.55513	2.25268	-4.12735
H	-3.15018	4.62934	-3.68493
C	3.81140	1.54081	0.69772
C	4.02722	0.59034	-0.30482
C	4.78855	1.69418	1.68877
C	5.19468	-0.16965	-0.32712
H	3.27544	0.41990	-1.07201
C	5.95567	0.93556	1.67113
H	4.63005	2.42366	2.48063
C	6.16592	0.00339	0.65610
H	5.33218	-0.90999	-1.10967
H	6.69975	1.07168	2.45191
H	7.07438	-0.59297	0.63842
Cu	-0.01480	-0.21996	-0.18915
C	2.24730	-1.38894	2.05502
H	2.24484	-0.39895	2.51222
C	3.44062	-2.26031	2.33435
H	4.36176	-1.80847	1.94414
H	3.58204	-2.40044	3.41478
H	3.33159	-3.24820	1.87713

C	1.23431	-1.73737	1.28152
C	0.27043	-2.16615	0.49323
C	-0.79213	-3.03284	1.03601
C	-0.49969	-4.00128	2.01419
C	-2.11416	-2.96222	0.56585
C	-1.48715	-4.84048	2.51844
H	0.52177	-4.08460	2.37969
C	-3.10561	-3.79704	1.07660
H	-2.36542	-2.23337	-0.19949
C	-2.79971	-4.74121	2.05484
H	-1.23187	-5.57602	3.27797
H	-4.12161	-3.70871	0.70033
H	-3.57246	-5.39711	2.44745
C	2.67608	-3.65089	-1.02498
C	2.16977	-2.69524	-1.91554
C	3.03731	-2.19088	-2.89701
C	4.36570	-2.60554	-2.97377
C	4.84729	-3.56194	-2.08056
C	3.99692	-4.08812	-1.11005
H	2.02797	-4.06048	-0.25561
H	2.66594	-1.46961	-3.62353
H	5.02091	-2.19211	-3.73690
H	5.87914	-3.89847	-2.14399
H	4.36352	-4.84006	-0.41534
Si	0.36082	-2.15258	-1.96374
H	-0.74187	-3.12098	-1.71846
H	0.08358	-0.63529	-1.90484
H	0.24966	-2.11144	-3.50737

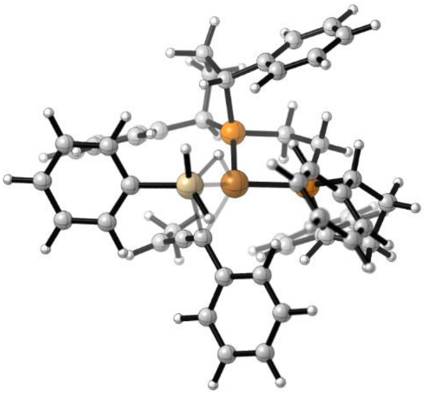

Conf4_*S*

C	2.67063	-0.58007	1.90899
C	4.19837	-0.71054	1.73522
C	4.51729	-1.44702	0.43232
C	3.58328	-0.93763	-0.67239

H	2.41940	0.45775	2.15006
H	4.61870	0.29986	1.70047
H	4.65114	-1.21387	2.59614
H	5.57193	-1.34045	0.15188
H	4.33477	-2.52004	0.56405
H	3.46739	-1.69778	-1.45464
P	1.92519	-0.85460	0.20106
C	1.44854	-2.64550	0.10464
H	1.62506	-2.92106	-0.94014
H	2.11242	-3.26263	0.71867
C	-0.00409	-2.92915	0.47506
H	-0.13108	-2.87184	1.56356
H	-0.27010	-3.94638	0.16736
C	-1.58487	-2.16152	-1.98266
C	-2.79870	-2.42503	0.46301
C	-2.87882	-3.00333	-1.94122
H	-1.78329	-1.20561	-2.47958
C	-3.08598	-3.56288	-0.53659
H	-2.58535	-2.83769	1.45618
H	-3.72224	-2.35031	-2.19134
H	-2.85463	-3.79888	-2.69339
H	-4.10305	-3.94959	-0.40209
H	-2.39370	-4.39194	-0.34736
P	-1.21319	-1.68843	-0.18897
C	3.95700	0.36181	-1.34647
C	5.07749	1.11538	-0.98809
C	3.15192	0.83102	-2.39427
C	5.37674	2.30804	-1.64840
H	5.73118	0.77975	-0.18888
C	3.44726	2.01836	-3.05418
H	2.27604	0.25751	-2.68593
C	4.56179	2.76809	-2.67817
H	6.25239	2.87890	-1.34972
H	2.80126	2.36121	-3.85817
H	4.79247	3.70053	-3.18614
C	1.99935	-1.46328	2.92248
C	2.47002	-2.74440	3.23024
C	0.81656	-1.02358	3.53089
C	1.77011	-3.56771	4.11035
H	3.38997	-3.10571	2.77670
C	0.11483	-1.84477	4.40933
H	0.44454	-0.02659	3.30430
C	0.58709	-3.12438	4.69902
H	2.15173	-4.56033	4.33579

H	-0.80096	-1.48243	4.86943
H	0.04231	-3.76767	5.38491
C	-0.38977	-2.78344	-2.64838
C	-0.13440	-4.15805	-2.58693
C	0.53811	-1.96206	-3.30044
C	1.02912	-4.69076	-3.13821
H	-0.84541	-4.81913	-2.09809
C	1.70260	-2.49132	-3.85094
H	0.34196	-0.89418	-3.36892
C	1.95727	-3.85954	-3.76385
H	1.21189	-5.76054	-3.07630
H	2.41037	-1.83352	-4.34902
H	2.86546	-4.27637	-4.19076
C	-3.95902	-1.46609	0.57884
C	-4.04591	-0.27450	-0.14416
C	-5.01259	-1.80081	1.43910
C	-5.14447	0.57141	0.00179
H	-3.23884	0.02460	-0.80867
C	-6.11362	-0.96276	1.58432
H	-4.95845	-2.72573	2.00996
C	-6.18033	0.23229	0.86732
H	-5.17732	1.50191	-0.55761
H	-6.91621	-1.23792	2.26399
H	-7.03345	0.89525	0.98556
Cu	-0.05413	0.27057	-0.07382
C	-2.20869	1.71498	2.15713
C	-1.15482	1.89788	1.38456
C	-0.15822	2.26190	0.59934
C	0.98501	2.99154	1.19432
C	0.78239	3.90744	2.24136
C	2.29245	2.83209	0.71041
C	1.84350	4.61137	2.80039
H	-0.22634	4.05456	2.62146
C	3.35783	3.53033	1.27533
H	2.47472	2.14385	-0.10928
C	3.14099	4.42309	2.32286
H	1.65768	5.30965	3.61328
H	4.36095	3.37648	0.88626
H	3.97198	4.97142	2.75908
C	-2.52916	3.96429	-0.64934
C	-2.10597	3.02311	-1.59802
C	-3.03106	2.62077	-2.57351
C	-4.33345	3.11736	-2.59028
C	-4.72975	4.05957	-1.64182

C	-3.82122	4.48682	-0.67483
H	-1.83773	4.29722	0.12015
H	-2.72431	1.91375	-3.34296
H	-5.03476	2.77818	-3.34901
H	-5.74007	4.46086	-1.65869
H	-4.11976	5.22813	0.06265
Si	-0.33702	2.34888	-1.70593
H	0.85186	3.24004	-1.55140
H	-0.16988	0.80714	-1.75499
H	-0.31351	2.34423	-3.26919
C	-2.54528	0.48425	2.94473
H	-2.34730	0.63294	4.01625
H	-3.60683	0.23266	2.84544
H	-1.95545	-0.37413	2.61017
H	-2.89567	2.56032	2.24990

5. References

1. Wang, Y., Wang, Z.-L., Ma, W.-W., & Xu, Y.-H. Copper-Catalyzed Markovnikov Selective 3,4-Hydrosilylation of 2-Substituted 1,3-Dienes. *Org. Lett.* **24**, 4081–4086 (2022).

2 Frisch, M. J., Trucks, G. W., Schlegel, H. B., Scuseria, G. E., Robb, M. A., Cheeseman, J. R., Scalmani, G., Barone, V., Petersson, G. A., Nakatsuji, H., Li, X., Caricato, M., Marenich, A. V., Bloino, J., Janesko, B. G., Gomperts, R., Mennucci, B., Hratchian, H. P., Ortiz, J. V., Izmaylov, A. F., Sonnenberg, J. L., Williams, Ding, F., Lipparini, F., Egidi, F., Goings, J., Peng, B., Petrone, A., Henderson, T., Ranasinghe, D., Zakrzewski, V. G., Gao, J., Rega, N., Zheng, G., Liang, W., Hada, M., Ehara, M., Toyota, K., Fukuda, R., Hasegawa, J., Ishida, M., Nakajima, T., Honda, Y., Kitao, O., Nakai, H., Vreven, T., Throssell, K., Montgomery Jr., J. A., Peralta, J. E., Ogliaro, F., Bearpark, M. J., Heyd, J. J., Brothers, E. N., Kudin, K. N., Staroverov, V. N., Keith, T. A., Kobayashi, R., Normand, J., Raghavachari, K., Rendell, A. P., Burant, J. C., Iyengar, S. S., Tomasi, J., Cossi, M., Millam, J. M., Klene, M., Adamo, C., Cammi, R., Ochterski, J. W., Martin, R. L., Morokuma, K., Farkas, O., Foresman, J. B., & Fox, D. J. Gaussian 16 Rev. A.03, Wallingford, CT, (2016).

3 Adamo, C., & Barone, V., Toward reliable density functional methods without adjustable parameters: The PBE0 model, *J. Chem. Phys.* **110**, 6158–6169 (1999).

4 Grimme, S., Ehrlich, S., & Goerigk. L., Effect of the damping function in dispersion corrected density functional theory, *J. Comp. Chem.* **32**, 1456–1465 (2011).

5 Ditchfifield, R., Hehre, W. J., & Pople. J. A., Self-Consistent Molecular Orbital Methods. 9. Extended Gaussian-type basis for molecular-orbital studies of organic molecules, *J. Chem. Phys.* **54**, 724–728 (1971).

6. Hehre, W. J., Ditchfifield, R., & Pople. J. A., Self-Consistent Molecular Orbital Methods. 12. Further extensions of Gaussian-type basis sets for use in molecular-orbital studies of organic-molecules, *J. Chem. Phys.* **56**, 2257–2261 (1972).

7. Fuentealba, P., Preuss, H., Stoll, H., & Szentpály. L. v., A Proper Account of Core-polarization with Pseudopotentials - Single Valence-Electron Alkali Compounds, *Chem. Phys. Lett.* **89**, 418–422 (1982).

8. Weigend, F., & Ahlrichs, R., Balanced basis sets of split valence, triple zeta valence and quadruple zeta valence quality for H to Rn: Design and assessment of accuracy, *Phys. Chem. Chem. Phys.* **7**, 3297–3305 (2005).

9. Yu, H. S., He, X., Li, S. L., & Truhlar, D. G., MN15: A Kohn-Sham Global-Hybrid Exchange-Correlation Density Functional with Broad Accuracy for Multi-Reference and Single-Reference Systems and Noncovalent Interactions". *Chem. Sci.* **7**, 5032–5051 (2016).

10. Marenich, A. V., Cramer, C. J., & Truhlar. D. G., Universal solvation model based on solute electron density and a continuum model of the solvent defined by the bulk dielectric constant and atomic surface tensions, *J. Phys. Chem. B* **113**, 6378–6396 (2009).

11. Lu, T., & Chen, Q., Interaction Region Indicator (IRI): A Simple Real Space Function Clearly Revealing Both Chemical Bonds and Weak Interactions, *Chem. Methods* **1**, 231–239 (2021).

12. Lu, T., & Chen, F., Multiwfn: A Multifunctional Wavefunction Analyzer, *J. Comput. Chem*. **33**, 580–592 (2012).

13. Humphrey, W., Dalke, A., & Schulten, K., VMD–Visual Molecular Dynamics, *J. Molec. Graphics* **14**, 33–38 (1996).

14. CYLview, 1.0b, Legault, C. Y., Université de Sherbrooke, (http://www.cylview.org). (2009)
